# A General Photoredox Platform for the Hydroarylation of Olefins

**DOI:** 10.1021/jacs.6c14333

**Published:** 2026-08-31

**Authors:** Yi-Lun Li, Johnny Z. Wang, David W. C. MacMillan

**Affiliations:** 6740 Merck Center for Catalysis at Princeton University, Princeton, New Jersey 08544, United States

## Abstract

Alkene hydroarylation provides a direct strategy for merging aryl groups with alkene-derived C­(sp^3^)-rich fragments, yet general methods that employ abundant aryl bromides across diverse alkene classes remain elusive. Herein, we report XAT-Heck, a radical hydroarylation platform that couples aryl bromides with alkenes under mild photoredox conditions through halogen-atom transfer (XAT)-enabled aryl radical generation. The method accommodates terminal, internal, and gem-disubstituted alkenes, as well as a broad range of heteroaryl and aryl bromides. Chemical-space mapping further supports the broad coverage of commercially available olefin and aryl bromide space. The versatility of this platform is showcased through deuterium incorporation, medium-ring formation, late-stage functionalization, and proteolysis-targeting chimera (PROTAC) library construction.

## Graphical abstract

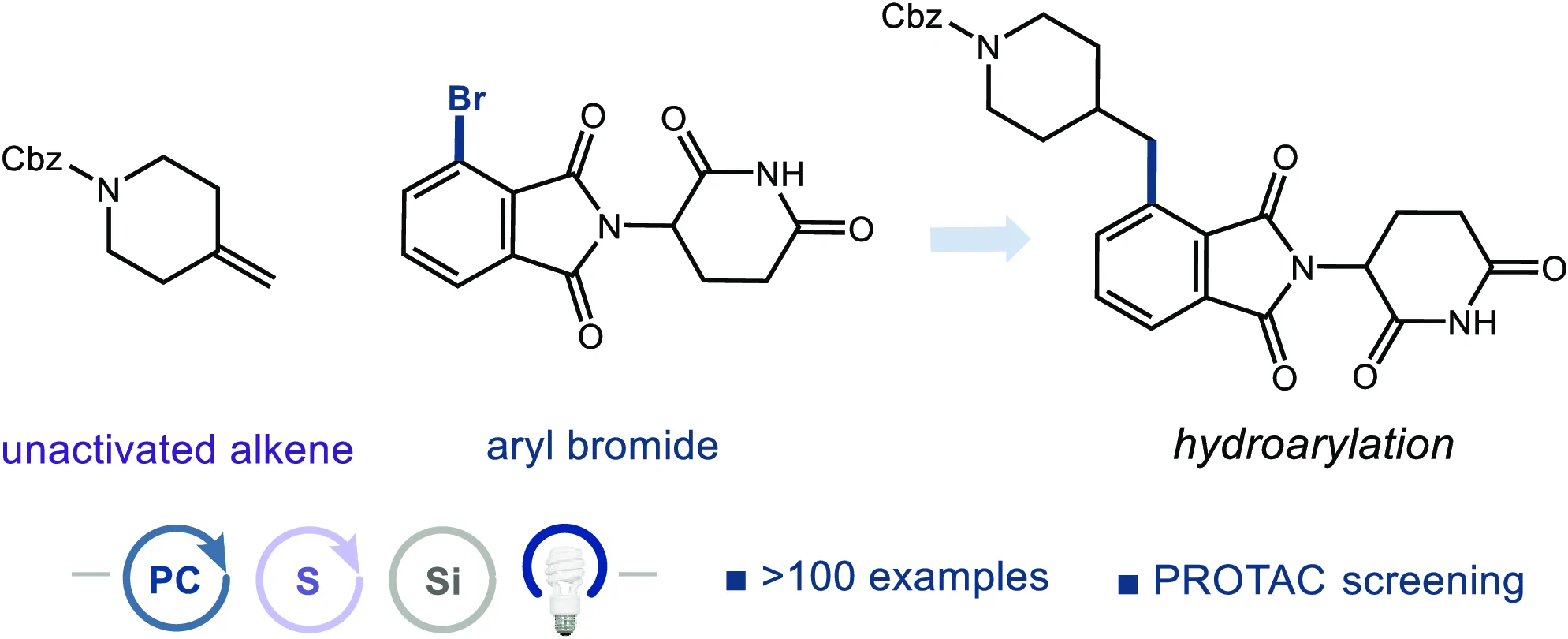

Increasing molecular saturation is a central goal in small-molecule drug discovery, as C­(sp^3^)-rich scaffolds can impart improved physicochemical properties and provide access to more three-dimensional chemical space.^1,2^ However, methods for the rapid construction of C­(sp^2^)–C­(sp^3^) bonds remain limited compared to the well-established repertoire of C­(sp^2^)–C­(sp^2^) cross-coupling reactions.^3,4^ This limitation is particularly relevant in early stage discovery programs, where efficient analogue synthesis and structural diversification are essential.^5,6^ In this context, direct alkene hydroarylation represents an attractive strategy for merging aryl fragments with alkene-derived C­(sp^3^)-rich fragments.^7–11^ The most established approach to alkene hydroarylation is the transition-metal-catalyzed reductive Heck reaction, which has been developed over several decades and has found broad application in modern organic synthesis.^9,12–14^ In this two-electron manifold, oxidative addition of an aryl halide to a transition-metal catalyst is followed by alkene coordination and migratory insertion to forge the key C­(sp^2^)–C­(sp^3^) bond (Figure 1a).^9^ Despite the power of reductive Heck chemistry, the requirements for alkene coordination and migratory insertion can impose substrate-dependent limitations, making general and selective hydroarylation difficult.^15–20^

**Figure 1:**
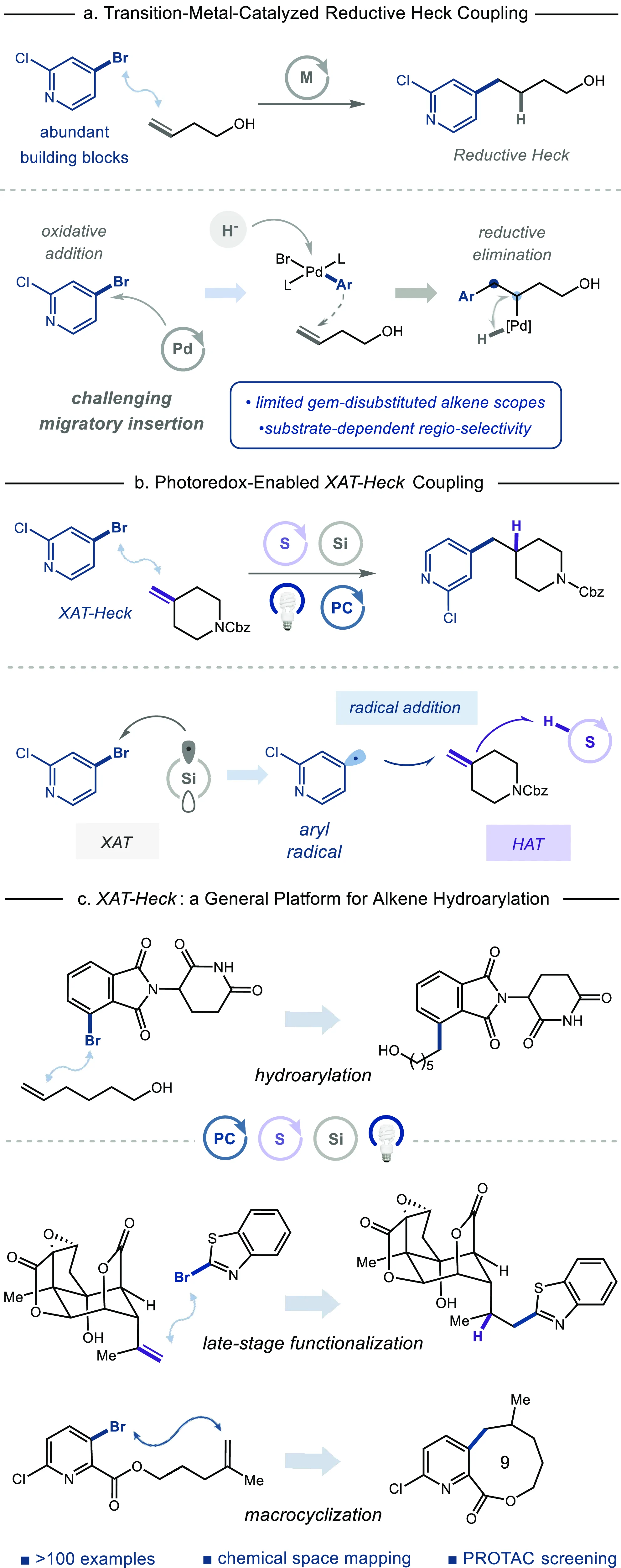
Design of the XAT-Heck strategy for alkene hydroarylation. PC, photocatalyst; Cbz, benzyloxycarbonyl.

Photoredox catalysis has enabled a wide range of open-shell bond constructions under mild conditions and has become a central platform for accessing reactivity patterns that are challenging in traditional two-electron manifolds.^21–23^ In the context of alkene hydroarylation, photoredox strategies offer a mechanistically distinct approach by replacing migratory insertion with direct aryl radical addition.^24,25^ In this pathway, an aryl radical adds to an alkene to generate a new alkyl radical, which can then be reduced or intercepted by a hydrogen atom donor to furnish the hydroarylated product. However, the generality of this approach is often dictated by the aryl radical generation step. As a result, many radical hydroarylation methods rely on readily reduced aryl electrophiles or are largely restricted to aryl iodides, limiting the direct use of abundant aryl bromides.^24–27^ Although more reducing conditions can expand the range of aryl halide coupling partners,^28–30^ such environments may limit functional-group tolerance, especially for substrates bearing reducible motifs. Accordingly, the development of complementary strategies that directly engage aryl bromides could further broaden the available synthetic toolkit, particularly in the context of complex molecule diversification.

We recently introduced a halogen-atom transfer (XAT) platform for generating aryl radicals from aryl bromides,^31,32^ and wondered whether this strategy could overcome the limitations of current radical hydroarylation methods by directly engaging aryl bromide feedstocks. In this design, single-electron oxidation of an adamantylaminosilane under mild photoredox conditions triggers a 1,2-aza-Brook rearrangement to generate a reactive silyl radical.^31^ This silyl radical abstracts bromine from an aryl bromide to generate an aryl radical, which then undergoes regioselective addition to an alkene. The resulting alkyl radical is intercepted by thiol-catalyzed, polarity-matched hydrogen atom transfer (HAT) to furnish the hydroarylated product (Figure 1b). Herein, we report a radical hydroarylation platform that couples aryl bromides with diverse alkene classes and enables late-stage hydroarylation, macrocyclization, and PROTAC library construction (Figure 1c).

Under optimized conditions (Table S3), aryl bromide (1.0 equiv) was combined with alkene (3.0 equiv) in MeCN in the presence of Ir­[dF­(CF_3_)­ppy]_2_(dtbbpy)­PF_6_ (1 mol %) as photocatalyst, adamantylaminosilane (AdNHSi­(TMS)_3_, 1.5 equiv) as the XAT reagent, Ph_3_SiSH (20 mol %) as hydrogen atom donor, and water (1.0 equiv) as an additive. Irradiation with 450 nm blue LEDs for 1 h furnished the desired hydroarylated product in 82% assay yield. Control experiments confirmed that light, photocatalyst, and adamantylamino-silane were all required for the reaction (Table S3). The reaction also proceeded efficiently when the alkene was used as the limiting reagent and the aryl bromide in excess (Table S5).

With optimized conditions in hand, we evaluated the alkene scope on a 0.5 mmol scale (Figure 2). The reaction tolerated both monosubstituted (**1**, 61% yield) and 1,2-disubstituted alkenes (**2**, 62% yield). Unsymmetrical 1,2-disubstituted activated alkenes were also compatible, providing the desired products in synthetically useful yields (see Section 2 of the Supporting Information). Six-membered cyclic 1,1-disubstituted alkenes underwent efficient hydroarylation (**3–5**, 52–76% yield). Notably, by switching to a deuterated solvent, we obtained **3** in 76% yield with 95% deuterium incorporation. The reaction proved compatible with various cyclic alkenes, including three- (**6**, 54% yield), four- (**7–9**, 52–78% yield), five- (**10–12**, 66–86% yield), and seven-membered ring systems (**13**, 59% yield). Linear gem-disubstituted alkenes bearing diverse functional groups were competent substrates (**14–16**, 65–79% yield). The platform further accommodated alkenes with varied electronic properties, including styrene (**17**, 45% yield), vinyl boronate (**18**, 65% yield), and Michael acceptor (**19**, 71% yield). α-Heteroatom-substituted alkenes containing oxygen (**20–22**, 53–82% yield), sulfur (**23**, 71% yield), or nitrogen (**24**, 84% yield) also furnished the desired products. To contextualize the breadth of the olefin scope, we projected the experimentally evaluated substrates onto a representative chemical-space map generated from 18,152 commercially available Enamine olefins^33^ using Morgan fingerprints and dimensionality reduction.^34–36^ The resulting map was partitioned into 16 clusters, and the tested olefins showed broad coverage across this space, occupying 9 clusters and displaying a Vendi score^37^ of 32.18 across 44 examples (see Supporting Information Section 6 for details), reflecting broad chemical diversity.

**Figure 2:**
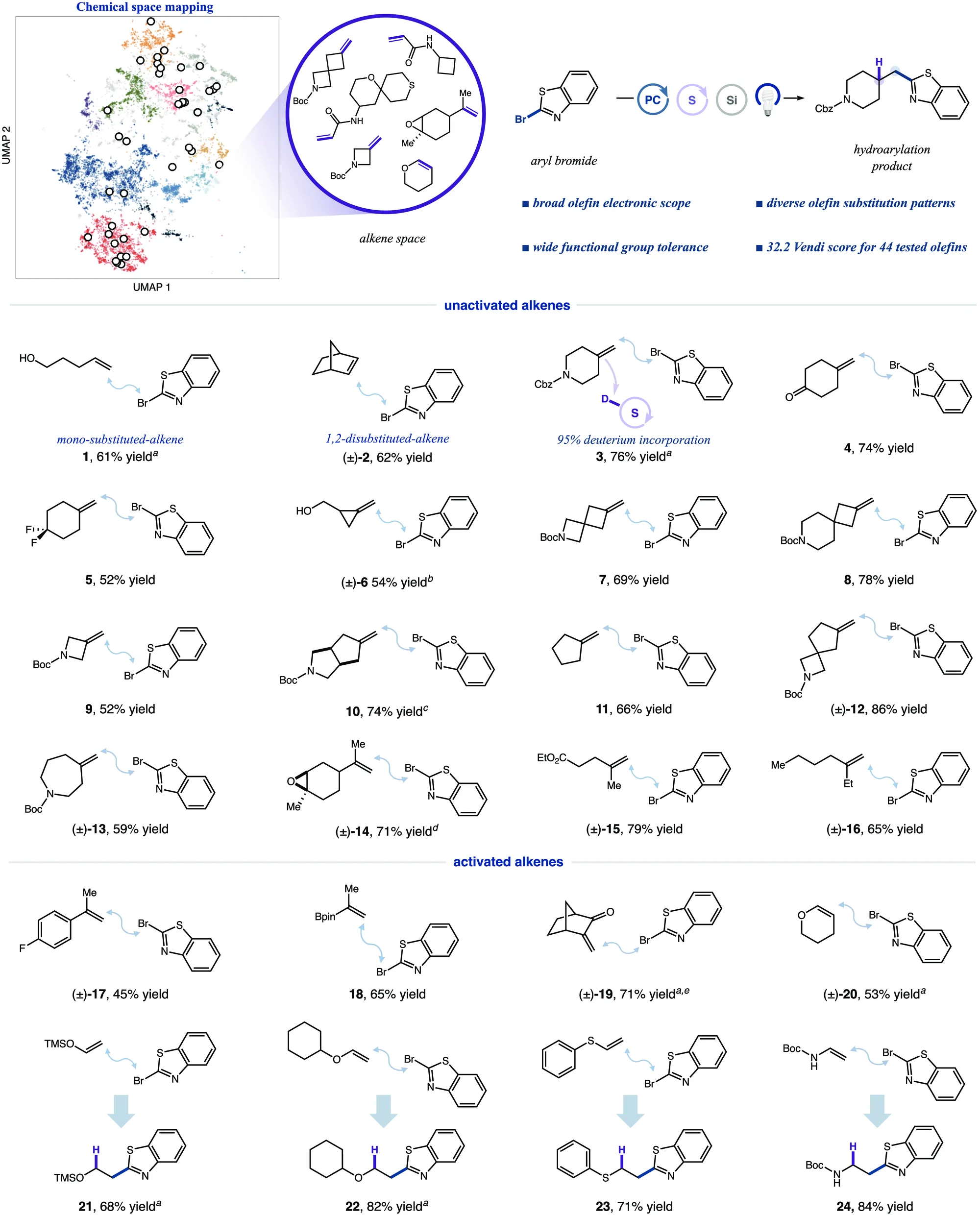
Alkene Scope for XAT-Heck Coupling. Reactions performed with alkene (0.5 mmol, 1.0 equiv), aryl bromide (2.0 equiv), AdNHSi­(TMS)_3_ (2.5 equiv), (TripS)_2_ (10 mol %), (Ir­[dF­(CF_3_)­ppy]_2_(dtbpy))­PF_6_ (1 mol %), MeCN/water (10:1, 0.09 M), integrated photoreactor (450 nm, M1 plate, 100% light intensity), 1.0 h. Yields are isolated. ^*a*^See SI for experimental details. ^*b*^2.7:1 d.r. unassigned. ^*c*^6.1:1 d.r. unassigned. ^*d*^1.3:1 d.r. unassigned. ^*e*^1:1 d.r. Boc, *tert*-butoxycarbonyl; Bpin, pinacol boronate; TMS, trimethylsilyl.

The aryl bromide scope was next evaluated on a 0.5 mmol scale (Figure 3). Heteroaryl bromides of particular importance in medicinal chemistry^38^ were broadly accommodated. Substituted bromopyridines spanning varied electronic environments furnished the desired hydroarylation products in good yields (**25–29**, 62–79% yield). Other six-membered heteroaryl bromides were also viable, including an N,N-dimethyl pyrimidinone derivative (**30**, 38% yield) and a pyrimidine substrate (**31**, 48% yield). Five-membered heteroaryl bromides were similarly competent, including thiazoles (**32–34**, 67–75% yield), imidazoles (**35–36**, 41–47% yield), isothiazole (**37**, 51% yield), thiadiazoles (**38–39**, 66–76% yield), and triazole substrates (**40**, 62% yield). More complex fused and bicyclic heteroaryl scaffolds were also incorporated, including a thiazolopyridine derivative (**41**, 77% yield), a thiazole bearing an aliphatic six-membered ring (**42**, 55% yield), and a benzimidazole (**43**, 62% yield). The method also proved amenable to simple aryl bromides, as a variety of electron-deficient aryl bromides afforded the desired products in good yields (**44–46**, 62–70% yield). Notably, an aryl bromide containing an aryl triflate was tolerated (**47**, 53% yield), highlighting the orthogonal activation of the C–Br bond under XAT conditions relative to conventional transition-metal oxidative addition pathways. An electron-rich aryl bromide bearing a methoxy substituent also underwent hydroarylation, albeit in diminished yield (**48**, 41% yield). Additional aryl bromide examples, including more than 40 substrates examined in smaller-scale screening, are provided in the Supporting Information. To visualize this substrate set within commercially available aryl bromide space, the evaluated examples were mapped against 35,491 Enamine aryl bromides^33^ using Morgan fingerprints and dimensionality reduction. The resulting aryl bromide chemical space was partitioned into 29 clusters, 21 of which were represented by the tested substrates.^34–36^ Together with a Vendi score^37^ of 42.20 across 66 examples, this analysis supports a broad sampling of both heteroaryl and aryl bromide chemical space (see Supporting Information Section 6 for details).

**Figure 3:**
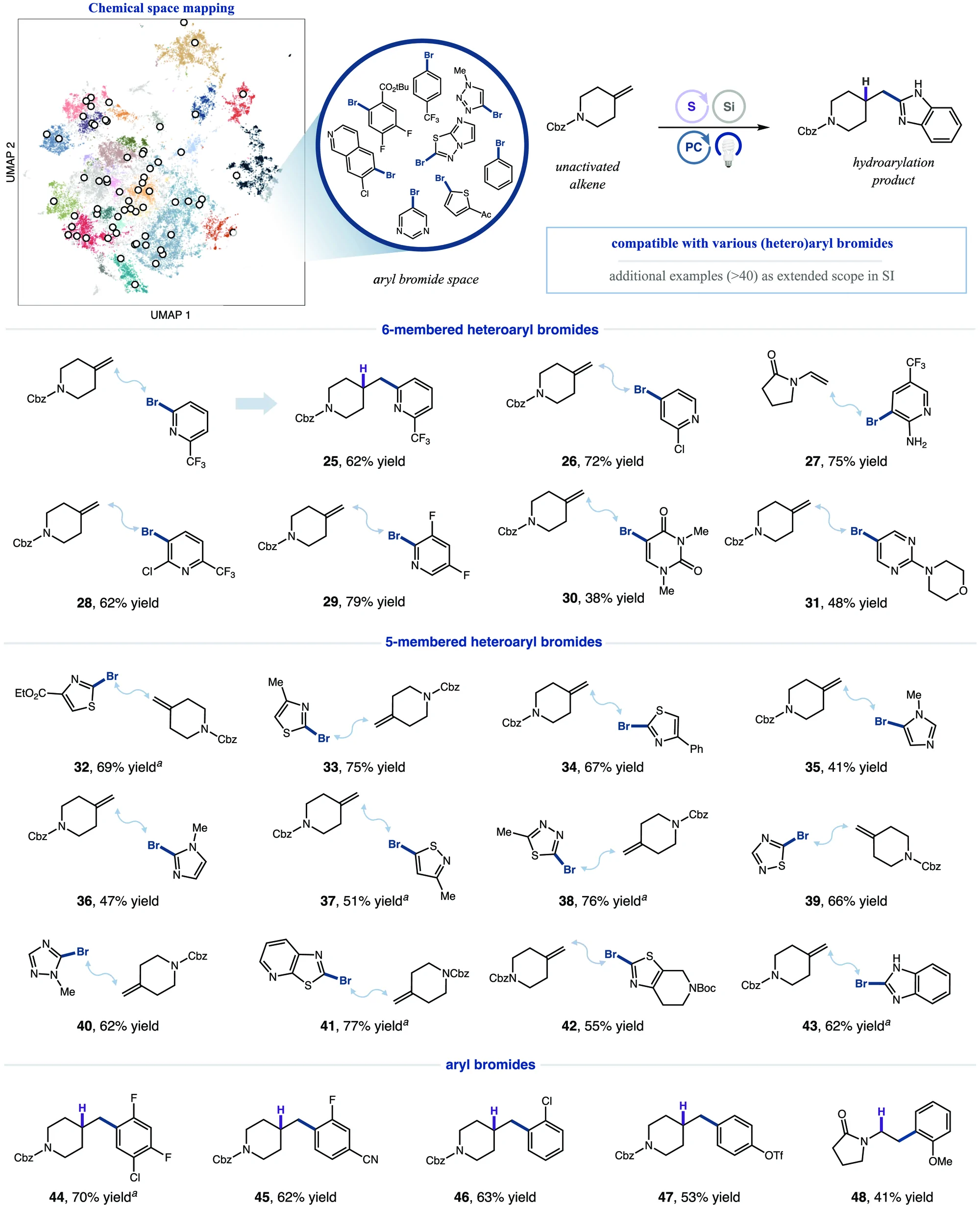
Aryl bromide scope for XAT-Heck coupling. Reactions performed with aryl bromide (0.5 mmol, 1.0 equiv), olefin (3.0 equiv), AdNHSi­(TMS)_3_ (1.5 equiv), Ph_3_SiSH (20 mol %), (Ir­[dF­(CF_3_)­ppy]_2_(dtbpy))­PF_6_ (1 mol %), MeCN/water (10:1, 0.09 M), integrated photoreactor (450 nm, M1plate, 100% light intensity), 1.0 h. Yields are isolated. ^*a*^(TripS)_2_ (10 mol %) was used instead of Ph_3_SiSH.

Beyond intermolecular fragment coupling, we sought to leverage the hydroarylation platform for the rapid assembly of semisaturated fused heterocycles (Figure 4). In analogy to our recently developed couple-close logic,^39,40^ we envisioned using XAT-Heck coupling to install the C­(sp^2^)–C­(sp^3^) bond and position a pendant nucleophile for subsequent annulation. Specifically, hydroarylation of an aryl bromide bearing an embedded leaving group with an olefin-containing alcohol or Michael acceptor would generate an intermediate poised for intramolecular S_N_Ar cyclization. As illustrated in Figure 4, this couple-close sequence provided access to a seven-membered spirocyclic product (**49**) in 52% yield over two steps. This strategy was also used to synthesize a bridged fused heterocycle (**50**), with the hydroarylation and cyclization steps proceeding in 69% and 53% yield, respectively. Importantly, the XAT-Heck platform could also be applied to the construction of medium-sized rings, which are often challenging to access by cyclization. Indeed, intramolecular hydroarylation furnished a series of nine-membered rings in good yields (**51–54**, 55–68% yield), demonstrating compatibility with varied substitution patterns. Beyond small-molecule building blocks, this platform was also suitable for the late-stage functionalization^41^ of complex molecules. Natural product and bioactive molecule derivatives, including pleuromutilin (**55**, 54% yield), betulinic acid (**56**, 52% yield), gibberellic acid (**57**, 42% yield), bromocaffeine (**58**, 52% yield), and picrotoxinin (**59**, 57% yield), underwent hydroarylation under standard conditions. Notably, the gibberellic acid derivative exhibited preferential functionalization of the gem-disubstituted alkene in the presence of a 1,2-disubstituted olefin. The method was further applied to drug-derived substrates, including an etoricoxib-derived aryl bromide that delivered spirocyclic product **60** in 57% yield and a lumacaftor derivative that furnished product **61** in 70% yield.

**Figure 4:**
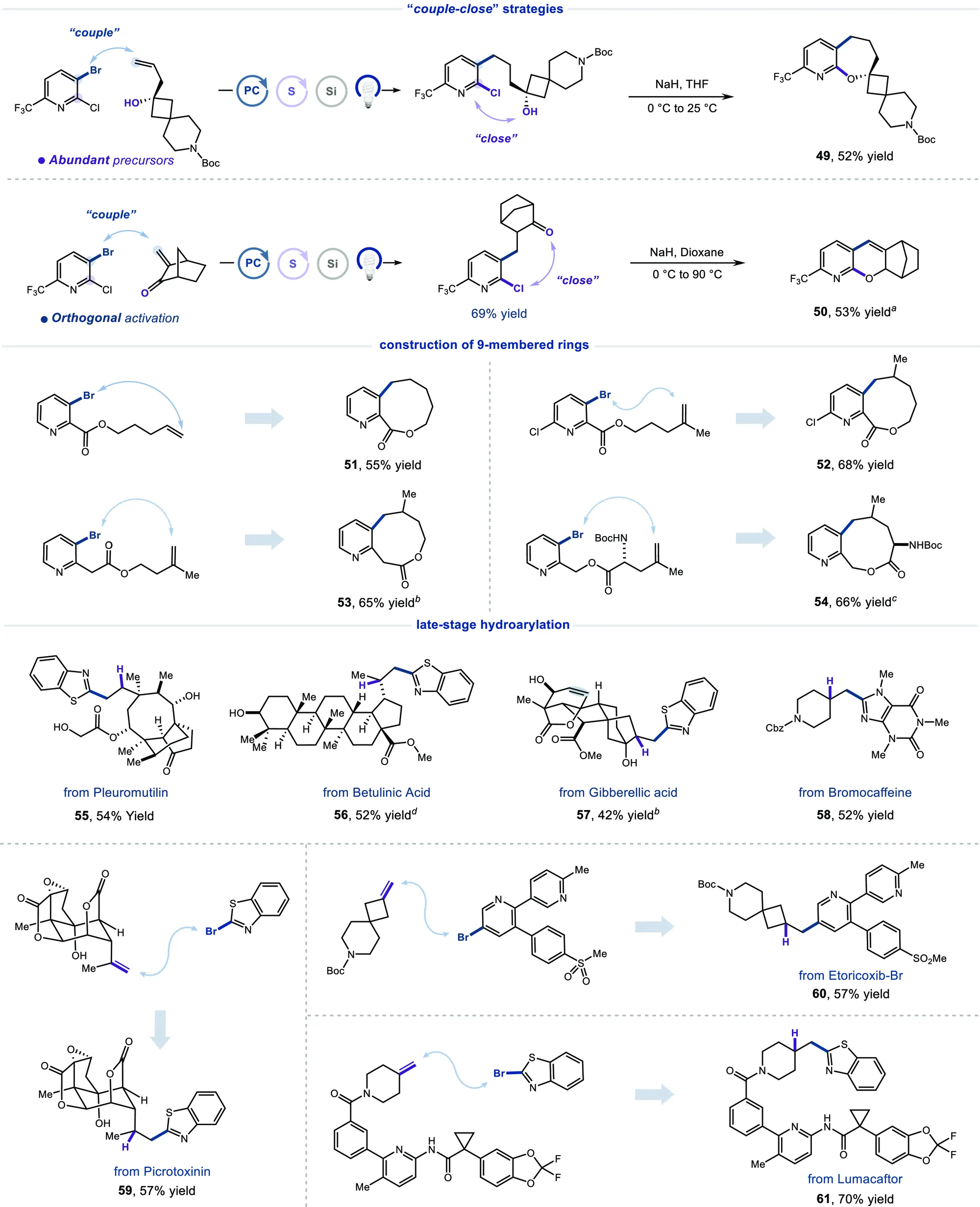
Synthetic applications. See Supporting Information for experimental details. ^*a*^1:1 d.r. ^*b*^2.2:1 d.r. unassigned. ^*c*^3.5:1 d.r. unassigned. THF, tetrahydrofuran.

Finally, we sought to apply XAT-Heck hydroarylation in a medicinally relevant diversification setting through construction of a PROTAC ligand–linker (Figure 5). PROTACs are heterobifunctional degraders that recruit an E3 ligase to a target protein and are composed of three modular elements: an E3 ligase ligand, a linker, and a target-protein ligand.^42^ Because linker identity can profoundly impact ternary complex formation, cellular permeability, and degradation efficiency,^43^ methods that enable rapid linker diversification are highly valuable in PROTAC optimization. We first examined whether this platform could directly append olefin-containing linkers to a thalidomide-derived cereblon (CRBN) ligand. Following brief reoptimization, DMSO was identified as a suitable cosolvent to improve substrate solubility. Under these modified conditions, a series of linkers bearing different chain lengths and terminal functional handles were coupled to the thalidomide scaffold in good yields (**62–64**, 54–73% yield). Encouraged by these results, we next applied the method to the synthesis of a fully elaborated PROTAC. Coupling of a JQ1-derived aryl bromide with a spirocyclic olefin linker furnished the corresponding JQ1–CRBN PROTAC (**65**) in 44% yield. The successful incorporation of a spirocyclic linker is notable, as conformationally restricted linkers provide access to linker geometries and physicochemical profiles that are difficult to achieve with conventional linear linker architectures. To further explore the utility of this approach for library synthesis, we developed a two-stage, one-pot workflow for a combinatorial PROTAC library. Five bifunctional olefin linkers bearing alcohol or amine handles were first coupled with three carboxylic acid-containing drug fragments through standard esterification or amidation protocols. Following acid–base workup, the crude mixtures were directly subjected to XAT-Heck coupling with the thalidomide-derived aryl bromide. This sequence delivered a 15-member PROTAC library without intermediate purification. Ultraperformance liquid chromatography–mass spectrometry (UPLC–MS) analysis showed detectable formation of all 15 products, with product peak areas ranging from 12% to 109% relative to the internal standard (ethyl benzoate). Additionally, a broader range of CRBN ligands was evaluated, with the corresponding hydroarylation products obtained in moderate to good yields in most cases. Selected CRBN ligands were subsequently applied to the synthesis of JQ1-derived PROTACs, and relatively intense product peaks were detected in all cases by UPLC–MS (see Section 8 of the Supporting Information for details). These results highlight the potential of XAT-Heck hydroarylation as a rapid fragment-coupling platform for PROTAC linker diversification and medicinal chemistry library generation.

**Figure 5:**
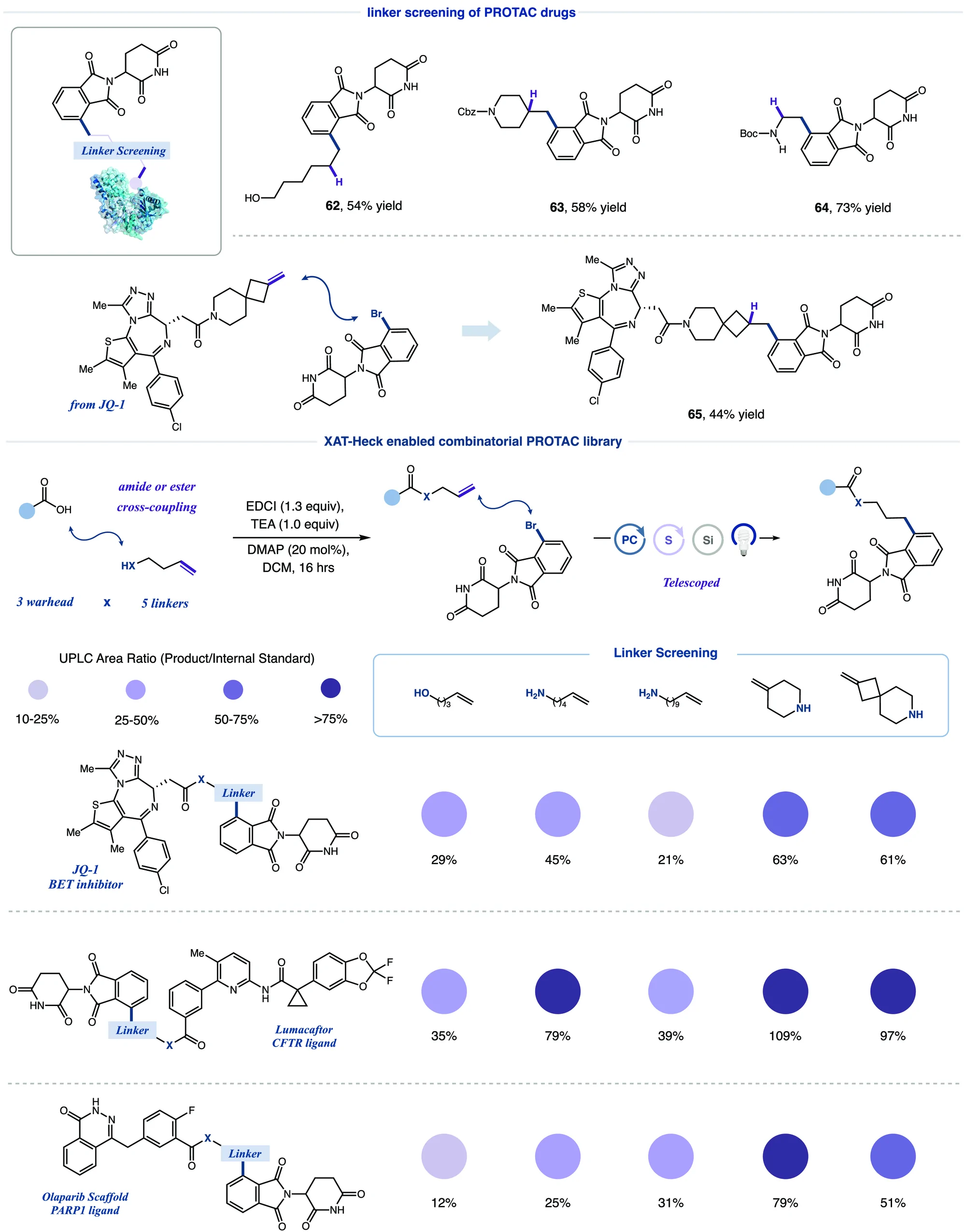
Drug-bioactive conjugation. See Supporting Information for experimental details. EDCI, 1-ethyl-3-(3-(dimethyl­amino)­propyl)­carbo­diimide; TEA, triethylamine; DMAP, 4-dimethyl­aminopyridine; DCM, dichloromethane; BET, bromodomain and extraterminal; CFTR, cystic fibrosis transmembrane conductance regulator; PARP1, poly­(ADP-ribose) polymerase 1 (PARP1).

In summary, we have developed XAT-Heck, a radical hydroarylation platform for the direct coupling of aryl bromides with diverse alkene classes. A wide range of terminal, internal, and gem-disubstituted alkenes, as well as heteroaryl and aryl bromides, served as competent coupling partners, enabling the synthesis of a broad collection of C­(sp^3^)-rich alkyl arenes. Notably, complex aryl bromides and alkene-containing natural product and drug derivatives underwent efficient hydroarylation, suggesting that this protocol could be applied to late-stage diversification. Additionally, medium-ring formation, couple-close annulation, and PROTAC diversification workflows were demonstrated, highlighting the utility of this strategy in medicinally relevant settings. We envision that the modular nature of this XAT-enabled hydroarylation platform, together with its broad substrate scope and compatibility with complex molecules, should offer accelerated access to pharmaceutically relevant C­(sp^2^)–C­(sp^3^) chemical space.

## Supplementary Material



## Notes

Additional experimental details, optimization studies, control studies, compound characterization, and spectra (PDF)
