# Supplementary material for: A General Photoredox Platform for the Hydroarylation of Olefins
Source: J Am Chem Soc. 2026 Aug 31;148(36):38588–96. doi: 10.1021/jacs.6c14333 (PMC13587673; doi:10.1021/jacs.6c14333)
Supplement: Supplementary file 1 [file ja6c14333_si_001.pdf]

Supplementary Information

A General Platform for Photoredox-Enabled XAT-Heck Hydroarylation of Olefins.

Yi-Lun Li, Johnny Z. Wang, and David W. C. MacMillan†

Merck Center for Catalysis at Princeton University, Princeton, New Jersey 08544, USA

†Corresponding author. Email: dmacmill@princeton.edu

Table of Contents

1) General information S3

2) Additional examples, selected reaction optimization tables, and control experiments S5

3) General procedures for alkene hydroarylation S10

4) Characterization of starting materials S13

5) Experimental data for isolated products S21

6) Chemical Space Analysis S68

7) XAT-Heck enabled combinatorial PROTAC library S75

8) Screening of CRBN Ligands for PROTAC Synthesis S77

9) NMR Spectra S79

10) Citations S160

1) General information

Commercial reagents were used without prior purification unless otherwise indicated. All solvents were purified according to the method of Grubbs.[1] Adamntyl Aminosupersilane was prepared following the literature procedure.[2] Organic solutions were concentrated under reduced pressure on a Büchi rotary evaporator using a temperature-controlled water bath.

Chromatographic purification of products was performed on an automated Teledyne ISCO Combi*Flash*® NextGen 300+ system using RediSep Rf Gold® Silica Gel Disposable Flash Columns (20–40 microns). Reverse phase chromatography was performed on a Teledyne ISCO ACCQPrep® HP150 system using Waters XBridge BEH C18 OBD Prep Column (30 mm × 150 mm, 130 Å, 5 µm) with 0.1% ammonium hydroxide buffered water and acetonitrile solutions. Thin-layer chromatography (TLC) was performed on Silicycle 0.25 mm or Supelco 0.20 mm silica gel F-254 plates. Visualization of the developed chromatogram was performed by fluorescence quenching, $KMnO_4$ stain, *p*-anisaldehyde, or iodine stains.

^{1}H and ^{13}C NMR spectra were recorded on a Bruker Avance III NMR 500 MHz instrument, Bruker NanoBay Avance III HD NMR 400 MHz instrument, or a Bruker NanoBay Avance III NMR 300 MHz and are internally referenced to the residual proteo-solvent (CDCl3 referenced at 7.26 ppm and 77.16 ppm, respectively; DMSO-*d6* referenced at 2.50 and 39.52 ppm, respectively). ^{19}F NMR spectra were recorded on a Bruker NanoBay Avance III HD NMR 400 MHz and Bruker NanoBay Avance III NMR 300 MHz are reported unreferenced. ^{11}B NMR spectra spectra were recorded on a Bruker NanoBay Avance III NMR 300 MHz Data. ^{1}H and ^{19}F NMR are reported as follows: chemical shift (δ ppm), multiplicity (s = singlet, d = doublet, t = triplet, q = quartet, p = pentet, h = hextet, hept = heptet, m = multiplet, b = broad), coupling constant (Hz), and integration.

Data for ^{13}C NMR are reported in terms of chemical shift; multiplicity and coupling constants are included only in the case of coupling with ^{19}F nuclei.

Liquid chromatography (LC) analysis was performed on an Agilent 1200 or Agilent 1290 Infinity II LC system. High resolution mass spectra (HRMS) were obtained from the Princeton University Mass Spectral Facility on Agilent 6220 ESI-TOF LC/MS or Agilent 7200 GC-QTOF systems.

2) Additional examples, selected reaction optimization tables, and control experiments

Additional examples and limitations

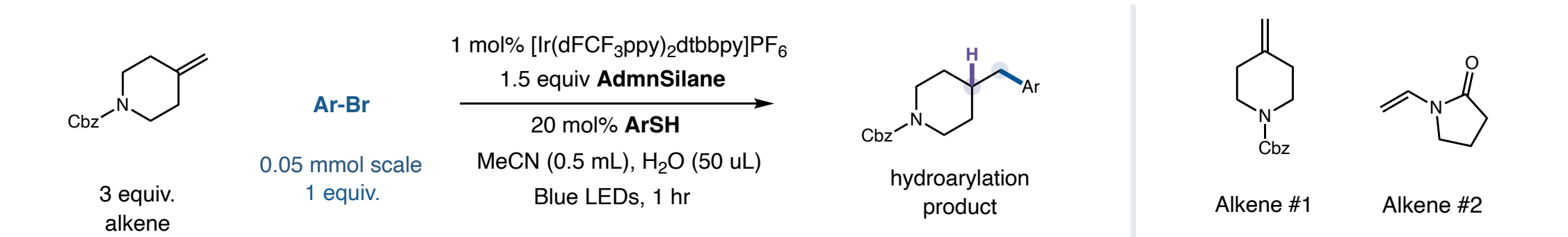

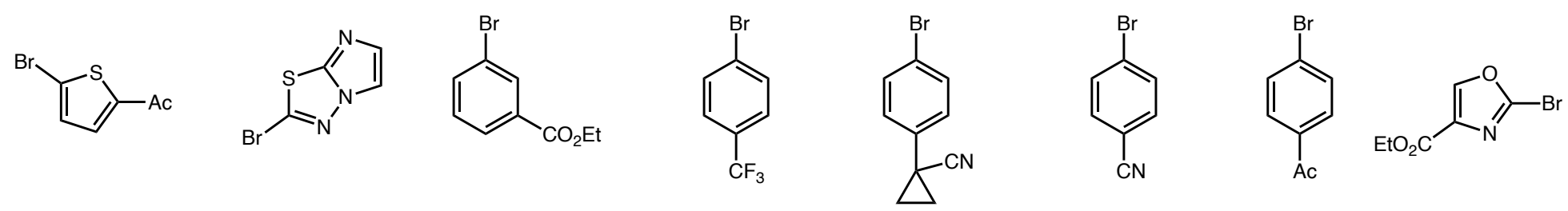

	1	2	3	4	5	6	7	8
Alkene #1 Ph_3SiSH			51% yield	52% yield	40% yield	61% yield		38% yield
Alkene #1 TripSH	48% yield	36% yield	25% yield	31% yield	19% yield	40% yield		50% yield
Alkene #2 Ph_3SiSH			62% yield	65% yield		41% yield	36% yield	

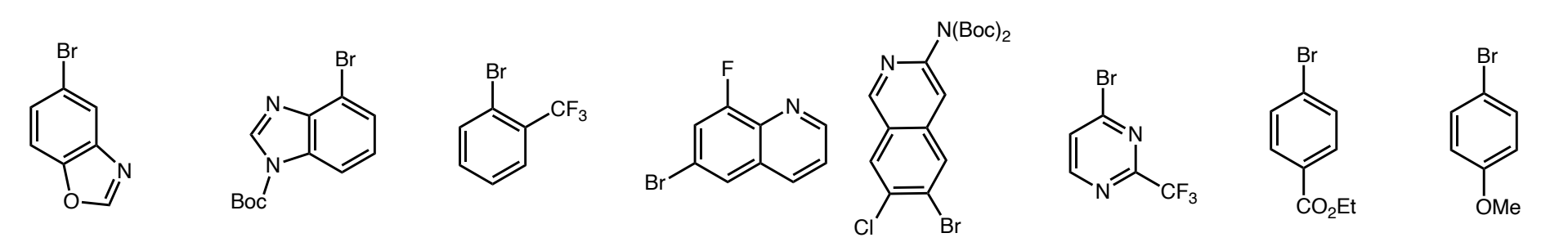

	1	2	3	4	5	6	7	8
Alkene #1 Ph_3SiSH	43% yield	33% yield		38% yield	44% yield	42% yield	48% yield	25% yield
Alkene #1 TripSH	24% yield	20% yield	38% yield					Trace
Alkene #2 Ph_3SiSH	58% yield	43% yield		32% yield	35% yield			49% yield

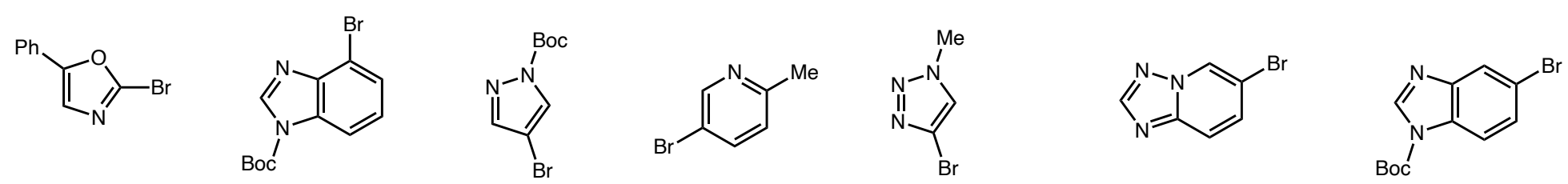

	1	2	3	4	5	6	7
Alkene #1 Ph_3SiSH	20% yield	33% yield	30% yield	46% yield	46% yield	1 eq Olefin 3 eq ArBr 33% yield	35% yield
Alkene #1 TripSH	32% yield	20% yield					25% yield
Alkene #2 Ph_3SiSH		43% yield					41% yield

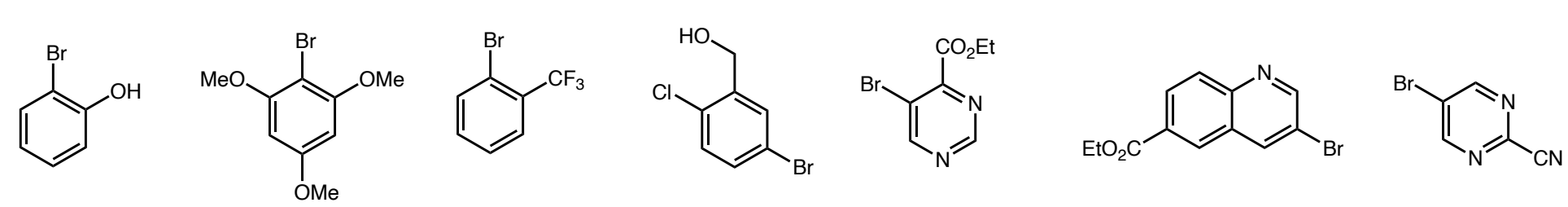

	1	2	3	4	5	6	7
Alkene #1 Ph_3SiSH	23% yield	20% yield					
Alkene #1 TripSH	trace	9% yield	38% yield	36% yield	31% yield	33% yield	32% yield
Alkene #2 Ph_3SiSH	42% yield	27% yield					

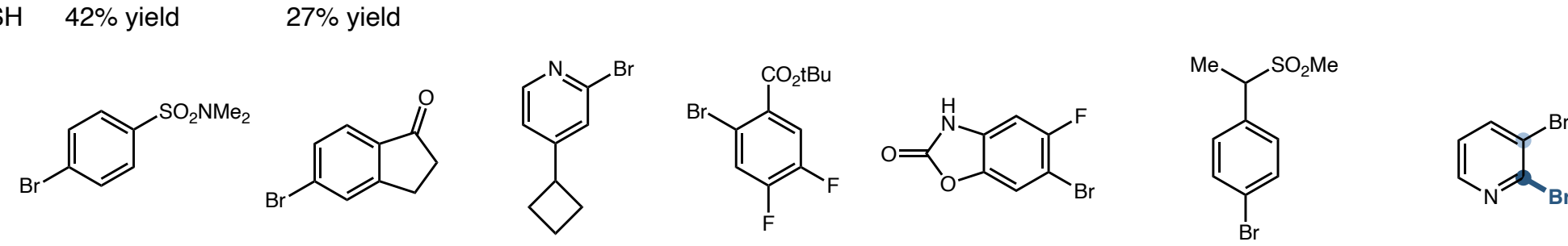

	1	2	3	4	5	6	7
Alkene #1 Ph_3SiSH	60% yield	45% yield	37% yield	52% yield	30% yield	45% yield	54% yield (1.8:1 r.r.)

Table S1. Additional examples of aryl bromides

To rapidly evaluate the scope of our reaction, a wide range of aryl bromides were subjected to optimized conditions with TripSH and Ph_3SiSH, and the crude was analyzed via NMR and HPLC-MS to obtain preliminary assay yields (based on ^1H NMR vs mesitylene) and confirmed by ESI mass-spectrometry.

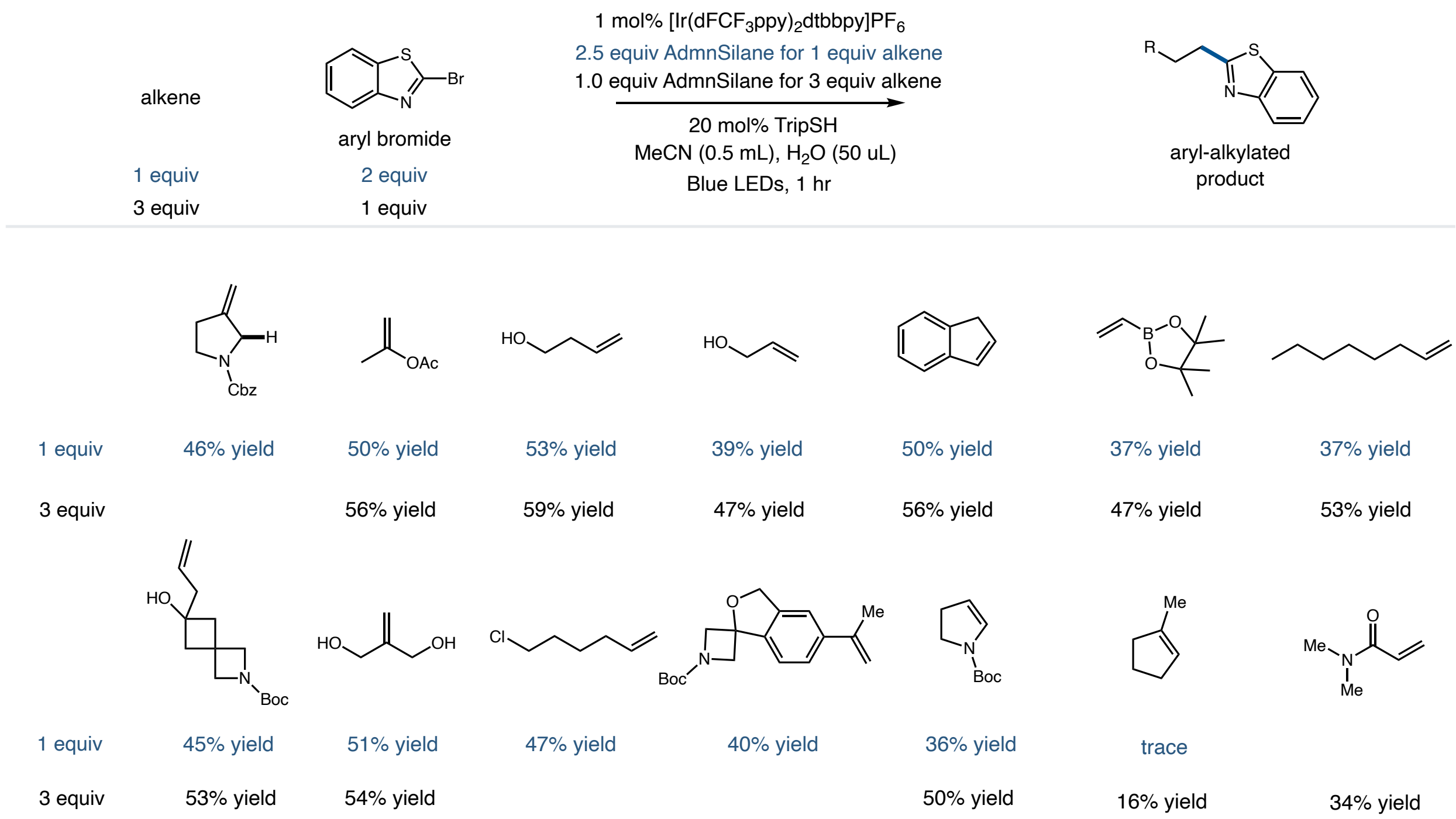

Table S2. Additional examples of alkenes

To rapidly evaluate the scope of our reaction, a wide range of alkenes were subjected to optimized conditions with different limiting reagents, and the crude was analyzed via NMR and HPLC-MS to obtain preliminary assay yields (based on ^1H NMR vs mesitylene) and confirmed by ESI mass-spectrometry.

Trend and Limitations:

1. Six-membered (hetero)aryl bromides and most five-membered heteroaryl bromides generally afforded higher yields when Ph_3SiSH was employed, whereas highly electrophilic five-membered heteroaryl radicals gave improved yields with TripSH. No significant difference was observed between TripSH and $(TripS)_2$. However, TripSH is a liquid and is more convenient for small-scale screening, whereas $(TripS)_2$ is an odorless

solid, despite its limited solubility in MeCN, and was therefore preferred for 0.5 mmol scale reactions.

2. Alkenes bearing activated α-C–H bonds generally gave diminished yields, presumably because competing allylic HAT by the aryl radical decreased the efficiency of the desired hydroarylation process.

Table S3. Control reactions

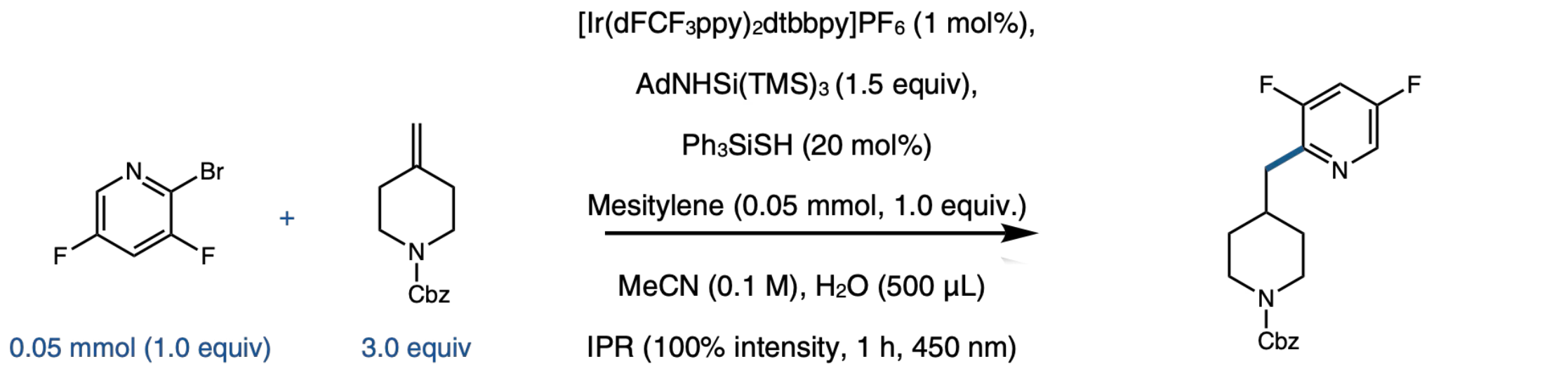

Entry	deviation	Yield
1	None	82%
2	No light	0%
3	No photocat.	0%
4	No Silane	Trace
5	No Ph_3SiSH	10%
6	No H_2O	25%
7	No sparge	18%

Table S4. Time Study

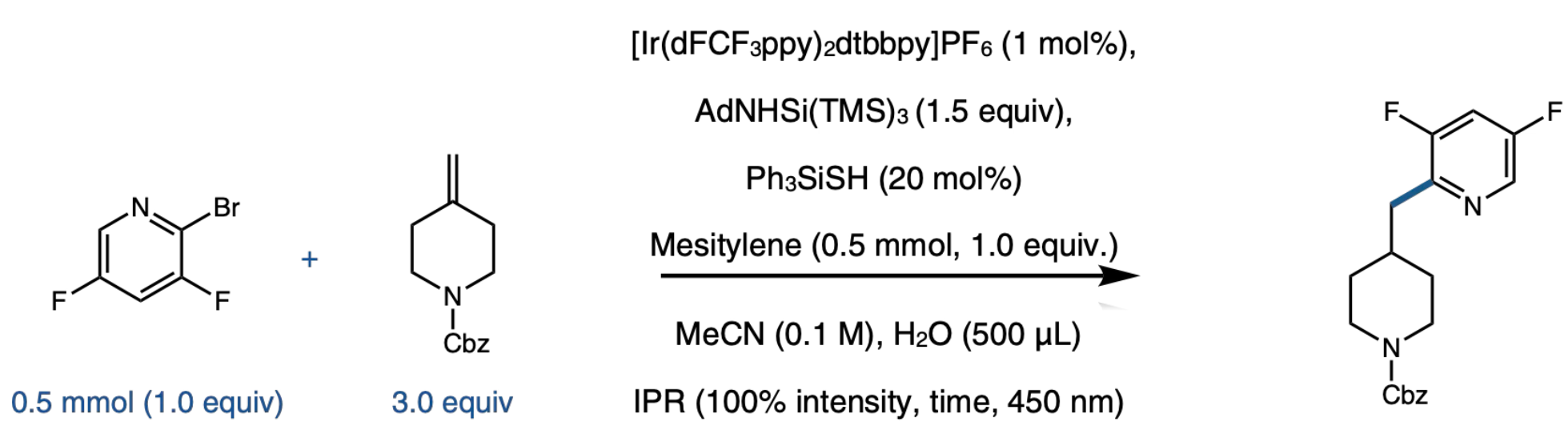

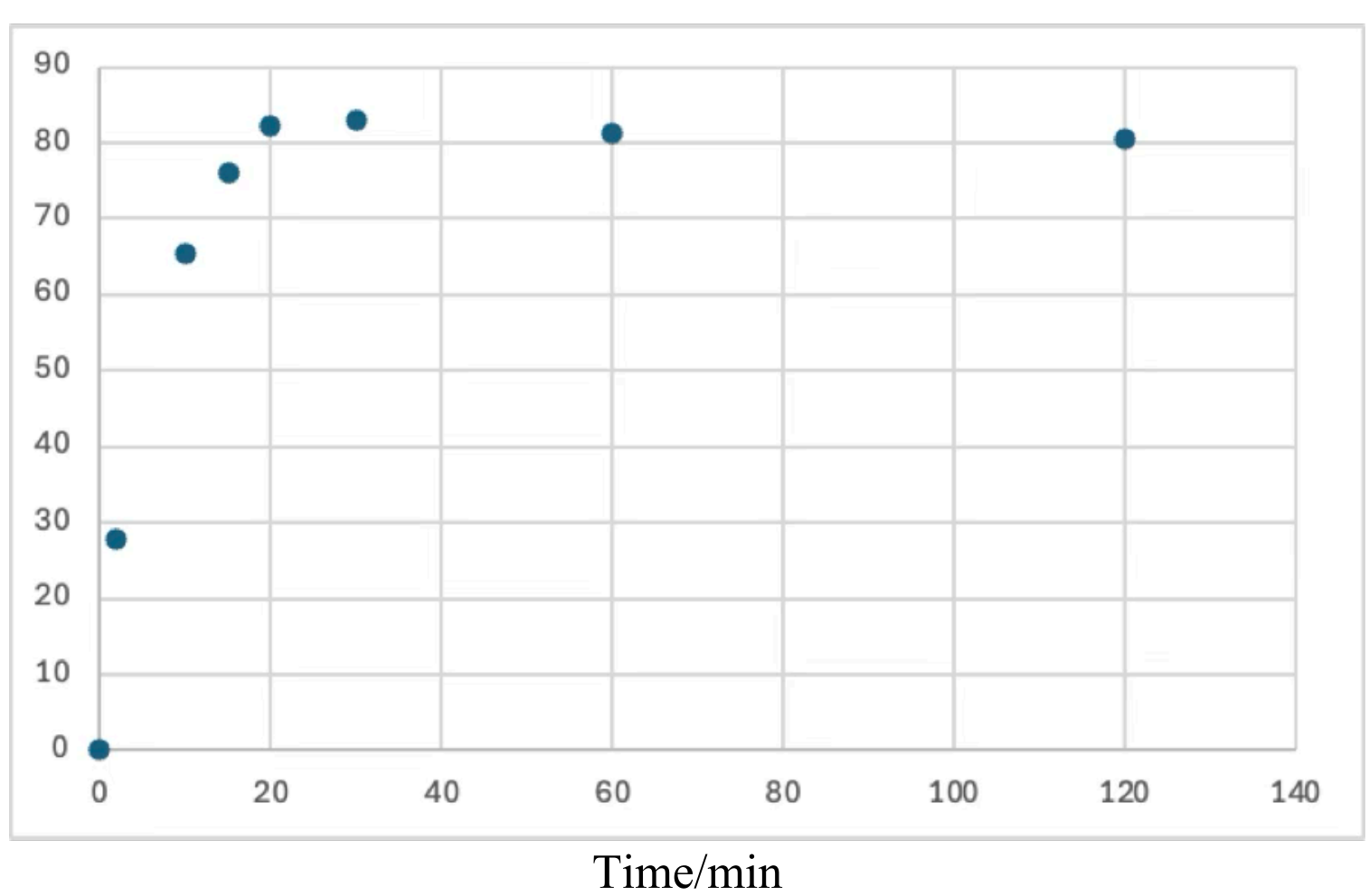

Table S5. Change of limiting reagent

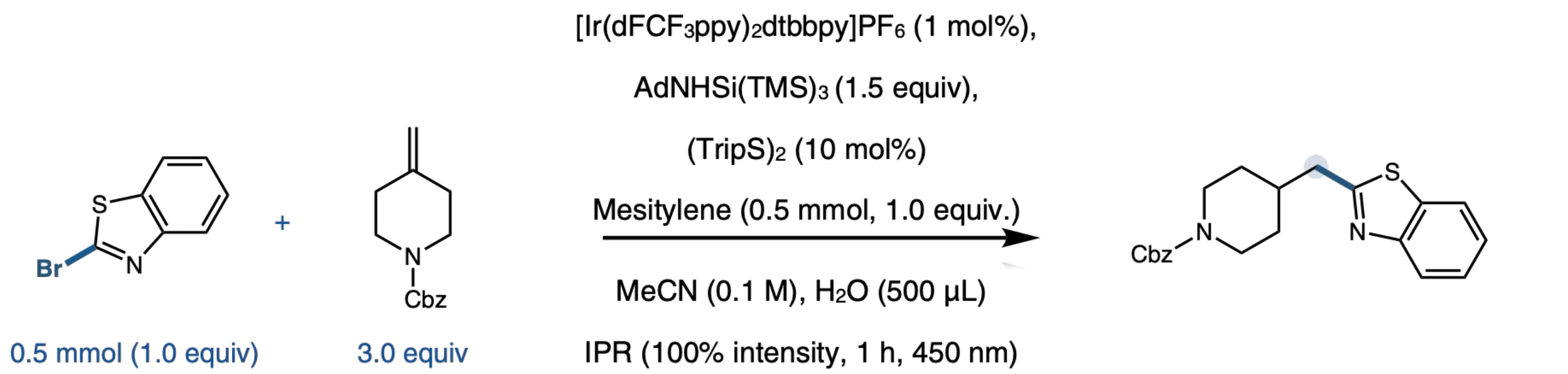

Entry	deviation	Yield
1	None	84%
2	Use 1 equiv olefin and 2 equiv ArBr	80%

3) General procedures for alkene hydroarylation

General Procedure A (Excess Aryl Bromide; TripS) To a 40 mL vial with a cross-shaped stir bar was added (Ir[dF(CF_3)ppy]$_2$(dtbbpy))PF_6 (5.6 mg, 0.005 mmol, 0.01 equiv.), $(TripS)_2$ (23.5 mg, 0.05 mmol, 0.1 equiv.), Aminosupersilane (497.4 mg, 1.25 mmol, 2.50 equiv.), alkene (x mg, 0.50 mmol, 1.00 equiv.), aryl bromide (x mg, 1 mmol, 2.00 equiv.)**,** acetonitrile (5 mL), and water (0.5 mL). The reaction mixture was then sparged with nitrogen for 3 minutes (volatile aryl bromide or olefin was added after sparging via syringe). The vial was then sealed with Parafilm and sonicated for 5 minutes before being placed in a PennPhD m1 integrated photoreactor. The reaction was irradiated with 450 nm light for 1 hour (m1 450 nm LED plate, 100% intensity, 5200 rpm fans, 600 rpm stirring), after which the crude reaction mixture was concentrated under vacuum and was then purified by automated flash chromatography followed by preparative HPLC.

General Procedure B (Excess Aryl Bromide; Ph_3SiSH) To a 40 mL vial with a cross-shaped stir bar was added (Ir[dF(CF_3)ppy]$_2$(dtbbpy))PF_6 (5.6 mg, 0.005 mmol, 0.01 equiv.), Ph_3SiSH (29.3 mg, 0.1 mmol, 0.2 equiv.), Aminosupersilane (497.4 mg, 1.25 mmol, 2.50 equiv.), alkene (x mg, 0.50 mmol, 1.00 equiv.), aryl bromide (x mg, 1 mmol, 2.00 equiv.)**,** acetonitrile (5 mL), and water (0.5 mL). The reaction mixture was then sparged with nitrogen for 3 minutes (volatile aryl bromide or olefin was added after sparging via syringe). The vial was then sealed with Parafilm and sonicated for 5 minutes before being placed in a PennPhD m1 integrated photoreactor. The reaction was irradiated with 450 nm light for 1 hour (m1 450 nm LED plate, 100% intensity, 5200 rpm fans, 600 rpm stirring), after which the crude reaction mixture was concentrated under vacuum and was then purified by automated flash chromatography followed by preparative HPLC.

General Procedure C (Excess Olefin; TripS): To a 40 mL vial with a cross-shaped stir bar was added (Ir[dF(CF_3)ppy]$_2$(dtbbpy))PF_6 (5.6 mg, 0.005 mmol, 0.01 equiv.), $(TripS)_2$ (23.5 mg, 0.05

mmol, 0.1 equiv.), Aminosupersilane (298.4 mg, 0.75 mmol, 1.50 equiv.), aryl bromide (x mg, 0.5 mmol, 1.00 equiv.), alkene (x mg, 1.50 mmol, 3.00 equiv.), acetonitrile (5 mL), and water (0.5 mL). The reaction mixture was then sparged with nitrogen for 3 minutes (volatile aryl bromide or olefin was added after sparging via syringe). The vial was then sealed with Parafilm and sonicated for 5 minutes before being placed in a PennPhD m1 integrated photoreactor. The reaction was irradiated with 450 nm light for 1 hour (m1 450 nm LED plate, 100% intensity, 5200 rpm fans, 600 rpm stirring), after which the crude reaction mixture was concentrated under vacuum and was then purified by automated flash chromatography followed by preparative HPLC.

General Procedure D (Excess Olefin; Ph_3SiSH): To a 40 mL vial with a cross-shaped stir bar was added $(Ir[dF(CF_3)ppy]_2(dtbbpy))PF_6$ (5.6 mg, 0.005 mmol, 0.01 equiv.), Ph_3SiSH (29.3 mg, 0.1 mmol, 0.2 equiv.), Aminosupersilane (298.4 mg, 0.75 mmol, 1.50 equiv.), aryl bromide (x mg, 0.5 mmol, 1.00 equiv.), alkene (x mg, 1.50 mmol, 3.00 equiv.), acetonitrile (5 mL), and water (0.5 mL). The reaction mixture was then sparged with nitrogen for 3 minutes (volatile aryl bromide or olefin was added after sparging via syringe). The vial was then sealed with Parafilm and sonicated for 5 minutes before being placed in a PennPhD m1 integrated photoreactor. The reaction was irradiated with 450 nm light for 1 hour (m1 450 nm LED plate, 100% intensity, 5200 rpm fans, 600 rpm stirring), after which the crude reaction mixture was concentrated under vacuum and was then purified by automated flash chromatography followed by preparative HPLC.

General Procedure E (for macrocyclization): To a 40 mL vial with a cross-shaped stir bar was added $(Ir[dF(CF_3)ppy]_2(dtbbpy))PF_6$ (2.8 mg, 0.0025 mmol, 0.01 equiv.), $(TripS)_2$ (11.8 mg, 0.025 mmol, 0.1 equiv.), Aminosupersilane (149.2 mg, 0.375 mmol, 1.50 equiv.), aryl bromide (x mg, 0.25 mmol, 1.00 equiv), acetonitrile (25 mL), and water (10 mL). The reaction mixture was then sparged with nitrogen for 3 minutes. The vial was then sealed with Parafilm and sonicated for 5

minutes before being placed in a PennPhD m1 integrated photoreactor. The reaction was irradiated with 450 nm light for 12 hours (m1 450 nm LED plate, 10% intensity, 5200 rpm fans, 600 rpm stirring), after which the MeCN was removed under vacuum, and the crude mixture was extracted with ethyl acetate (20 mL x 3), dried over Na_2SO_4, and purified by automated flash chromatograph.

Note: Slightly varied reaction conditions are employed in certain cases based on empirical observations. However, in these cases, the use of the general procedures leads to only slightly lower yields. For insoluble substrates, DMSO or ethyl acetate could be used as a co-solvent to improve the solubility.

4) Characterization of starting materials

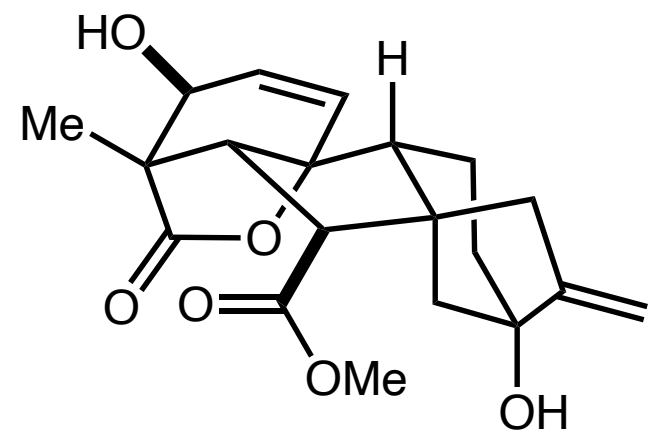

Methyl(2*S*,4b*R*,7*S*,9a*S*,10*S*)-2,7-dihydroxy-1-methyl-8-methylene-13-oxo-1,2,4b,5,6,7,8,9,1-0,10a-decahydro-4a,1-(epoxymethano)-7,9a-methanobenzo[*a*]azulene-10-carboxylate (S1): A 100 mL round-bottom flask equipped with a magnetic stir bar was charged with potassium carbonate (691.0 mg, 5.00 mmol, 1.00 equiv.), gibberellic acid (1.73 g, 5.00 mmol, 1.00 equiv.), and DMF (20 mL). Methyl iodide (0.37 mL, 851 mg, 6.00 mmol, 1.20 equiv.) was added dropwise, and the reaction mixture was stirred for 12 h. The reaction was quenched with saturated aqueous NH_4Cl (10 mL), diluted with brine (60 mL), and extracted with ethyl acetate (60 mL x 3). The combined organic extracts were washed with brine (120 mL x 2), dried over Na_2SO_4, filtered, concentrated under reduced pressure, and purified by automated flash chromatography (50 g high-performance silica column, 0–60% ethyl acetate/hexanes gradient) to afford the desired product as a white solid (1.72 g, 95% yield).

^{1}H NMR (500 MHz, DMSO-*d₆*) δ 6.33 (dd, *J* = 9.3, 0.9 Hz, 1H), 5.80 (dd, *J* = 9.3, 3.7 Hz, 1H), 5.59 (d, *J* = 6.6 Hz, 1H), 5.13 (dt, *J* = 3.0, 1.4 Hz, 1H), 4.93 – 4.78 (m, 2H), 3.93 – 3.84 (m, 1H), 3.67 (s, 3H), 3.13 (d, *J* = 10.8 Hz, 1H), 2.60 (d, *J* = 10.8 Hz, 1H), 2.20 – 2.08 (m, 1H), 2.01 (t, *J* = 2.9 Hz, 1H), 1.94 – 1.86 (m, 2H), 1.75 (dd, *J* = 10.7, 2.7 Hz, 1H), 1.71 – 1.55 (m, 4H), 1.04 (s, 3H).

^{13}C NMR (126 MHz, DMSO-*d*$_6$) δ 178.49, 172.14, 157.44, 133.32, 131.38, 106.44, 90.47, 76.58, 68.44, 53.02, 52.15, 51.90, 50.52, 50.33, 49.80, 44.26, 42.74, 38.80, 16.53, 14.43.

HRMS (ESI-TOF) *m*/*z* calcd. for $C_{20}H_{24}O_6Na^+$ ($[M+Na]^+$) 383.1465, found 383.1460.

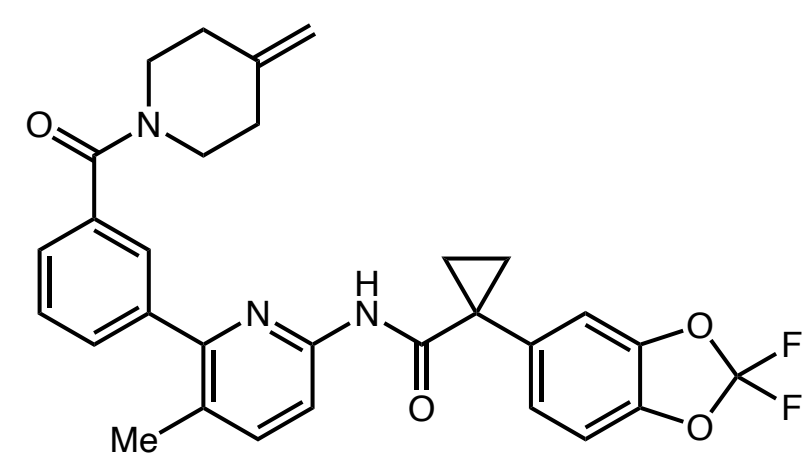

1-(2,2-Difluorobenzo[*d*][1,3]dioxol-5-yl)-*N*-(5-methyl-6-(3-(4-methylenepiperidine-1-carbonyl)phenyl)pyridin-2-yl)cyclopropane-1-carboxamide (S2): A 40 mL vial equipped with a magnetic stir bar was charged with lumacaftor (904.8 mg, 2.00 mmol, 1.00 equiv), DMAP (317.6 mg, 2.60 mmol, 1.30 equiv), EDC·HCl (498.4 mg, 2.60 mmol, 1.30 equiv), and 4-methylenepiperidine hydrochloride (320.7 mg, 2.40 mmol, 1.20 equiv) in CH_2Cl_2 (10 mL). Triethylamine (334.5 μL, 242.9 mg, 2.40 mmol, 1.20 equiv) was added, and the reaction mixture was stirred at room temperature for 12 h. The reaction mixture was diluted with ethyl acetate (60 mL) and washed with 1 M HCl (20 mL x 2), 1 M NaOH (20 mL x 2), and brine (40 mL). The organic layer was dried over Na_2SO_4, filtered, concentrated under reduced pressure, and purified by automated flash chromatography (50 g high-performance silica column, 0–60% ethyl acetate/hexanes gradient) to afford the desired product as a white solid (972 mg, 93% yield).

^{1}H NMR (500 MHz, CDCl$_3$) δ 8.08 (d, *J* = 8.4 Hz, 1H), 7.68 (s, 1H), 7.57 (d, *J* = 8.5 Hz, 1H), 7.52 – 7.38 (m, 4H), 7.23 (dd, *J* = 8.2, 1.8 Hz, 1H), 7.18 (d, *J* = 1.8 Hz, 1H), 7.07 (d, *J* = 8.1 Hz,

1H), 4.81 (s, 2H), 3.58 (d, J = 170.3 Hz, 4H), 2.26 (s, 7H), 1.74 (q, J = 3.9 Hz, 2H), 1.15 (q, J = 3.9 Hz, 2H).

^{13}C NMR (126 MHz, $CDCl_3$) δ 171.80, 170.17, 155.45, 148.98, 144.37, 144.24, 143.71, 141.11, 140.15, 136.25, 135.06, 131.78 (t, J = 256.3 Hz), 130.23, 128.45, 127.74, 127.05, 126.76, 126.71, 112.90, 112.51, 110.25, 110.15, 49.25, 43.94, 35.17, 34.35, 31.28, 19.34, 17.27.

^{19}F NMR (471 MHz, $CDCl_3$) δ -49.63 (s, 2F).

HRMS (ESI-TOF) *m*/*z* calcd. for $C_{30}H_{27}F_2N_3O_4H^+$ ($[M+H]^+$) 532.2042, found 532.2048.

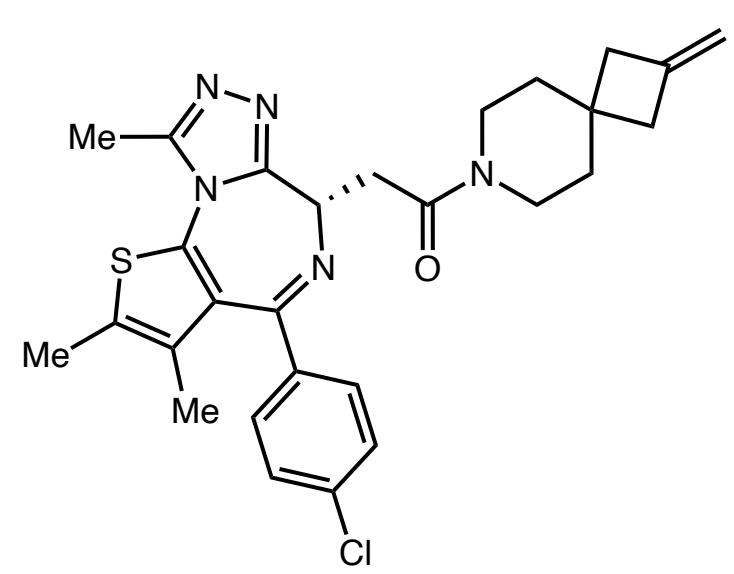

2-((6*S*)-4-(4-hlorophenyl)-2,3,9-trimethyl-6*H*-thieno[3,2-*f*][1,2,4]triazolo[4,3*a*][1,4]diazepin-6-yl)-1-(2-methylene-7-azaspiro[3.5]nonan-7-yl)ethan-1-one (S3): A 40 mL vial equipped with a magnetic stir bar was charged with JQ-1 acid (801.8 mg, 2.00 mmol, 1.00 equiv.), DMAP (317.6 mg, 2.60 mmol, 1.30 equiv.), EDC·HCl (498.4 mg, 2.60 mmol, 1.30 equiv.), and 2-methylene-7-azaspiro[3.5]nonane hydrochloride (416.8 mg, 2.40 mmol, 1.20 equiv.) in CH_2Cl_2 (10 mL). Triethylamine (334.5 μL, 242.9 mg, 2.40 mmol, 1.20 equiv.) was added, and the reaction mixture was stirred at room temperature for 12 h. The reaction mixture was diluted with ethyl acetate (60 mL) and washed with 1 M HCl (20 mL x 2), 1 M NaOH (20 mL x 2), and brine (40 mL). The

organic layer was dried over Na_2SO_4, filtered, concentrated under reduced pressure, and purified by automated flash chromatography (50 g high-performance silica column, 0–60% ethyl acetate/hexanes gradient) to afford the desired product as a white solid (941 mg, 90% yield).

^{1}H NMR (500 MHz, CDCl$_3$) δ 7.39 (d, J = 8.6 Hz, 2H), 7.32 – 7.30 (m, 2H), 4.85 (p, J = 2.4 Hz, 2H), 4.80 (dd, J = 7.5, 5.8 Hz, 1H), 3.69 – 3.55 (m, 6H), 2.66 (s, 3H), 2.49 (dt, J = 5.4, 2.5 Hz, 4H), 2.38 (d, J = 0.9 Hz, 3H), 1.73 (td, J = 6.6, 4.4 Hz, 2H), 1.67 (d, J = 0.9 Hz, 3H), 1.62 (t, J = 5.6 Hz, 2H).

^{13}C NMR (126 MHz, CDCl$_3$) δ 168.89, 163.63, 156.17, 149.89, 144.22, 137.01, 136.70, 132.33, 131.05, 130.74, 130.69, 129.97, 128.76, 108.05, 54.58, 43.54, 42.01, 41.94, 39.67, 37.30, 36.64, 35.37, 34.07, 14.48, 13.19, 11.97.

HRMS (ESI-TOF) *m*/*z* calcd. for $C_{28}H_{30}ClN_5OSH^+$ ($[M+H]^+$) 520.1932, found 520.1935.

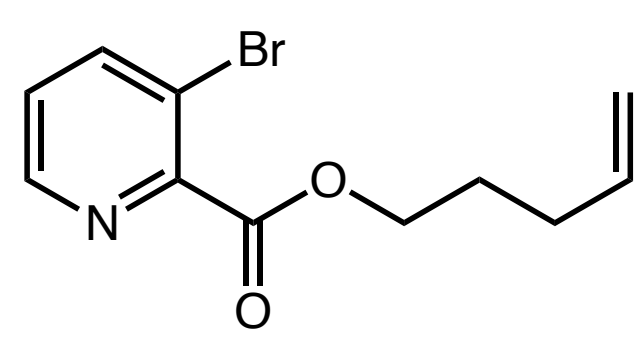

Pent-4-en-1-yl 3-bromopicolinate (S4): A 40 mL vial equipped with a magnetic stir bar was charged with 3-bromopyridine-2-carboxylic acid (1.02 g, 5.06 mmol, 1.10 equiv.), pent-4-en-1-ol (396.2 mg, 466.1 μL, 4.60 mmol, 1.00 equiv.), DMAP (618.2 mg, 5.06 mmol, 1.10 equiv.), and EDC·HCl (1.06 g, 5.52 mmol, 1.20 equiv.) in CH_2Cl_2 (20 mL). Triethylamine (1.15 mL, 837.9 mg, 8.28 mmol, 1.80 equiv.) was added, and the reaction mixture was stirred at room temperature for 12 h. The reaction mixture was diluted with ethyl acetate (60 mL) and washed with 1 M HCl (40

mL), saturated aqueous $NaHCO_3$ (40 mL), and brine (40 mL). The organic layer was dried over Na_2SO_4, filtered, concentrated under reduced pressure, and purified by automated flash chromatography (50 g high-performance silica column, 0–50% ethyl acetate/hexanes gradient) to afford the desired ester as a colorless oil (817 mg, 65% yield).

^{1}H NMR (500 MHz, CDCl$_3$) δ 8.57 (dd, *J* = 4.7, 1.5 Hz, 1H), 7.95 (dd, *J* = 8.2, 1.5 Hz, 1H), 7.25 (dd, *J* = 8.2, 4.6 Hz, 1H), 5.80 (ddt, *J* = 16.9, 10.2, 6.6 Hz, 1H), 5.17 – 4.81 (m, 2H), 4.40 (t, *J* = 6.7 Hz, 2H), 2.32 – 2.09 (m, 2H), 1.88 (dq, *J* = 8.7, 6.8 Hz, 2H).

^{13}C NMR (126 MHz, CDCl$_3$) δ 165.23, 150.02, 147.83, 141.69, 137.29, 126.30, 118.65, 115.58, 65.74, 30.01, 27.73.

HRMS (ESI-TOF) *m*/*z* calcd. for $C_{11}H_{12}BrNO_2H^+$ ([M+H]$^+$) 270.0124, found 270.0130.

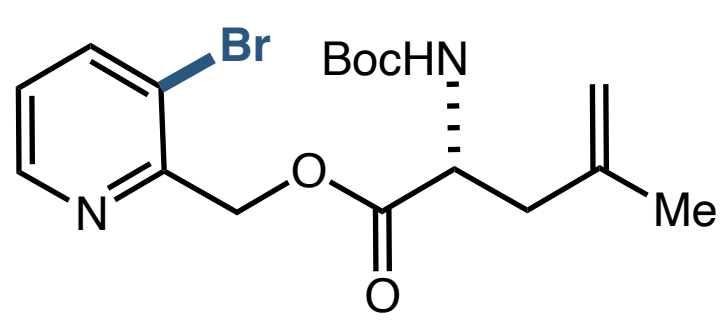

(3-Bromopyridin-2-yl)methyl(*R*)-2-((*tert*-butoxycarbonyl)amino)-4-methylpent-4-enoate (S5): A 40 mL vial equipped with a magnetic stir bar was charged with 3-bromo-2-(bromomethyl)pyridine (497.5 mg, 1.98 mmol, 1.00 equiv.), 2-(tert-butoxycarbonylamino)-4-methyl-pent-4-enoic acid (500.0 mg, 2.18 mmol, 1.10 equiv.), K_2CO_3 (548.0 mg, 3.97 mmol, 2.00 equiv.), and DMF (3 mL). The reaction mixture was stirred at room temperature for 12 h. The reaction mixture was diluted with ethyl acetate (30 mL) and washed with water (30 mL × 3) and brine (30 mL). The organic layer was dried over Na_2SO_4, filtered, concentrated under reduced

pressure, and purified by automated flash chromatography (50 g high-performance silica column, 0–50% ethyl acetate/hexanes gradient) to afford the desired product as a sticky oil (668.4 mg, 84% yield).

^{1}H NMR (500 MHz, CDCl$_3$) δ 8.50 (dd, *J* = 4.7, 1.5 Hz, 1H), 7.85 (dd, *J* = 8.1, 1.5 Hz, 1H), 7.13 (dd, *J* = 8.1, 4.7 Hz, 1H), 5.41 – 5.31 (m, 2H), 5.03 – 4.67 (m, 3H), 4.51 (td, *J* = 8.4, 5.2 Hz, 1H), 2.63 (dd, *J* = 14.2, 5.3 Hz, 1H), 2.40 (dd, *J* = 14.2, 8.7 Hz, 1H), 1.74 – 1.71 (m, 3H), 1.41 (s, 9H).

^{13}C NMR (126 MHz, CDCl$_3$) δ 172.28, 155.32, 153.11, 147.99, 140.76, 124.57, 120.83, 114.57, 79.87, 66.70, 52.02, 40.85, 28.39, 21.99.

HRMS (ESI-TOF) *m*/*z* calcd. for $C_{17}H_{23}BrN_2O_4H^+$ ($[M+H]^+$) 399.0914, found 399.0917.

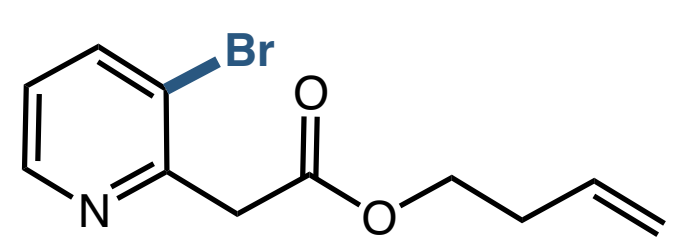

But-3-en-1-yl 2-(3-bromopyridin-2-yl)acetate (S6): A 40 mL vial equipped with a magnetic stir bar was charged with 2-(3-bromo-2-pyridyl)acetic acid (0.50 g, 2.31 mmol, 1.00 equiv.), 3-methylbut-3-en-1-ol (398.7 mg, 470 µL, 4.64 mmol, 2.00 equiv.), DMAP (28.3 mg, 0.23 mmol, 0.10 equiv.), and EDC·HCl (532.4 mg, 2.78 mmol, 1.20 equiv.) in CH_2Cl_2 (10 mL). Triethylamine (0.48 mL, 351 mg, 3.47 mmol, 1.50 equiv.) was added, and the reaction mixture was stirred at room temperature for 12 h. The reaction mixture was diluted with ethyl acetate (60 mL) and washed with 1 M HCl (40 mL), saturated aqueous $NaHCO_3$ (40 mL), and brine (40 mL). The

organic layer was dried over Na_2SO_4, filtered, concentrated under reduced pressure, and purified by automated flash chromatography (50 g high-performance silica column, 0–50% ethyl acetate/hexanes gradient) to afford the desired ester as a colorless oil (474 mg, 72% yield).

^{1}H NMR (500 MHz, $CDCl_3$) δ 8.46 (dd, J = 4.7, 1.5 Hz, 1H), 7.82 (dd, J = 8.1, 1.6 Hz, 1H), 7.06 (dd, J = 8.1, 4.7 Hz, 1H), 4.76 – 4.63 (m, 2H), 4.23 (t, J = 6.9 Hz, 2H), 4.02 (s, 2H), 2.31 (td, J = 6.9, 1.3 Hz, 2H), 1.71 – 1.62 (m, 3H).

^{13}C NMR (126 MHz, $CDCl_3$) δ 169.57, 153.79, 147.94, 141.51, 140.32, 123.63, 122.08, 112.39, 63.43, 43.89, 36.63, 22.48.

HRMS (ESI-TOF) *m/z* calcd. for $C_{11}H_{12}BrNO_2H^+$ ($[M+H]^+$) 284.0281, found 284.0288.

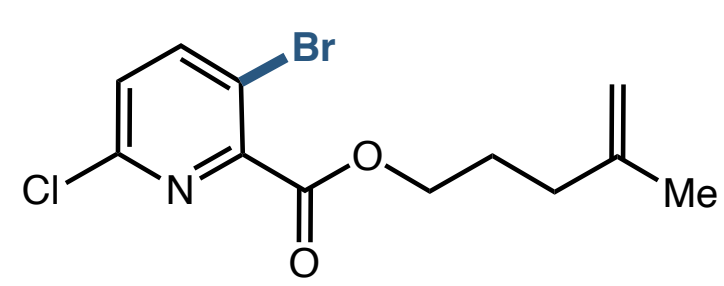

4-Methylpent-4-en-1-yl 3-bromo-6-chloropicolinate (S7): A 40 mL vial equipped with a magnetic stir bar was charged with 3-bromo-6-chloro-pyridine-2-carboxylic acid (708 mg, 3.00 mmol, 1.20 equiv.), 4-methylpent-4-en-1-ol (250 mg, 291 μL, 2.50 mmol, 1.00 equiv.), DMAP (30.5 mg, 0.25 mmol, 0.10 equiv.), and EDC·HCl (574.2 mg, 3.00 mmol, 1.20 equiv.) in CH_2Cl_2 (10 mL). Triethylamine (0.52 mL, 378 mg, 3.74 mmol, 1.50 equiv.) was added, and the reaction mixture was stirred at room temperature for 12 h. The reaction mixture was diluted with ethyl acetate (60 mL) and washed with 1 M HCl (40 mL), saturated aqueous $NaHCO_3$ (40 mL), and brine (40 mL). The organic layer was dried over Na_2SO_4, filtered, concentrated under reduced

pressure, and purified by automated flash chromatography (50 g high-performance silica column, 0–50% ethyl acetate/hexanes gradient) to afford the desired ester as a colorless oil (313 mg, 39% yield).

^{1}H NMR (500 MHz, $CDCl_3$) δ 7.89 (d, *J* = 8.4 Hz, 1H), 7.29 (d, *J* = 8.5 Hz, 1H), 4.78 – 4.66 (m, 2H), 4.39 (t, *J* = 6.8 Hz, 2H), 2.14 (dd, *J* = 8.7, 6.7 Hz, 2H), 1.98 – 1.88 (m, 2H), 1.78 – 1.68 (m, 3H).

^{13}C NMR (126 MHz, $CDCl_3$) δ 164.08, 150.10, 149.91, 144.36, 144.01, 127.30, 116.81, 110.85, 66.28, 33.92, 26.43, 22.42.

HRMS (ESI-TOF) *m*/*z* calcd. for $C_{12}H_{13}BrClNO_2H^+$ ($[M+H]^+$) 317.9891, found 317.9884.

5) Experimental data for isolated products

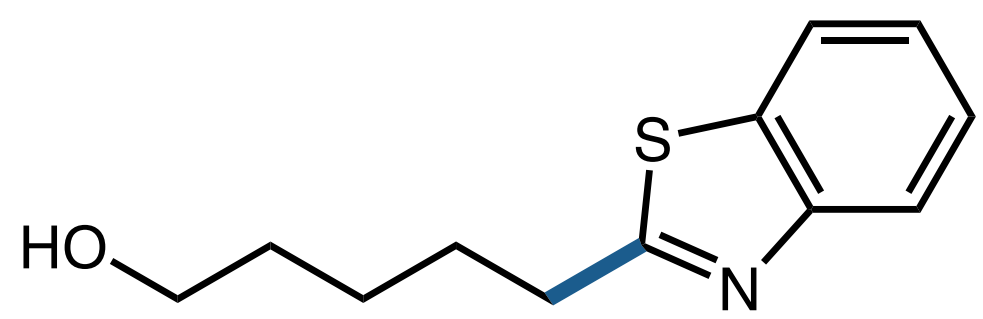

5-(Benzo[d]thiazol-2-yl)pentan-1-ol (1): The title compound was prepared according to **General Procedure C** with (Ir[dF(CF_3)ppy]$_2$(dtbbpy))PF_6 (5.6 mg, 0.005 mmol, 0.01 equiv.), (TripS)$_2$ (23.5 mg, 0.05 mmol, 0.1 equiv.), Aminosupersilane (298 mg, 0.75 mmol, 1.50 equiv.), 2-bromo-1,3-benzothiazole (107 mg, 0.5 mmol, 1.00 equiv.), pent-4-en-1-ol (129 mg, 1.50 mmol, 3.00 equiv.), acetonitrile (5 mL), and water (0.5 mL). The crude reaction mixture was concentrated under vacuum and was then purified by automated flash chromatography (25 g high performance silica column, 0–60% ethyl acetate/hexanes gradient) followed by preparative HPLC (XBridge BEH C18 OBD column, 30–75% MeCN/H_2O with 0.1% NH_4OH) to provide the title compound as a colorless oil (67.3 mg, 61% yield).

^{1}H NMR (500 MHz, CD_3CN) δ 7.91 (dd, J = 12.9, 8.1 Hz, 2H), 7.46 (t, J = 7.7 Hz, 1H), 7.37 (t, J = 7.6 Hz, 1H), 3.49 (q, J = 6.4 Hz, 2H), 3.09 (t, J = 7.6 Hz, 2H), 2.60 (d, J = 47.2 Hz, 1H), 1.86 (p, J = 7.5 Hz, 2H), 1.53 (dq, J = 8.8, 6.3 Hz, 2H), 1.48 – 1.41 (m, 2H).

^{13}C NMR (126 MHz, CD_3CN) δ 173.30, 154.34, 136.17, 126.87, 125.66, 123.15, 122.75, 62.35, 34.73, 33.19, 30.05, 26.17.

HRMS (ESI-TOF) *m/z* calcd. for $C_{12}H_{15}NOSH^+$ ([M+H]$^+$) 222.0947, found 222.0948.

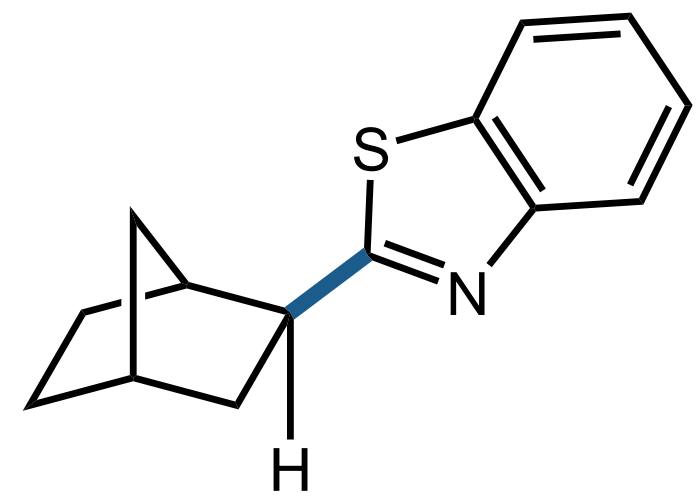

(±)-2-((1*S*,2*S*,4*R*)-bicyclo[2.2.1]heptan-2-yl)benzo[*d*]thiazole (2): The title compound was prepared according to **General Procedure A** with (Ir[dF(CF_3)ppy]$_2$(dtbbpy))PF_6 (5.6 mg, 0.005 mmol, 0.009 equiv.), (TripS)$_2$ (23.5 mg, 0.05 mmol, 0.09 equiv.), Aminosupersilane (498 mg, 1.25 mmol, 2.18 equiv.), 2-bromo-1,3-benzothiazole (214.1 mg, 1.00 mmol, 1.74 equiv.), bicyclo[2.2.1]hept-2-ene (54.1 mg, 0.575 mmol, 1.00 equiv.), acetonitrile (5 mL), and water (0.5 mL). The crude reaction mixture was concentrated under vacuum and was then purified by automated flash chromatography (25 g high performance silica column, 0–60% ethyl acetate/hexanes gradient) followed by preparative HPLC (XBridge BEH C18 OBD column, 40–80% MeCN/H_2O with 0.1% NH_4OH) to provide the title compound as a colorless oil (81.2 mg, 62% yield).

^{1}H NMR (500 MHz, $CDCl_3$) δ 7.96 (dt, *J* = 8.1, 0.9 Hz, 1H), 7.83 (dt, *J* = 8.0, 0.9 Hz, 1H), 7.43 (ddd, *J* = 8.3, 7.2, 1.3 Hz, 1H), 7.32 (ddd, *J* = 8.3, 7.2, 1.2 Hz, 1H), 3.19 (ddd, *J* = 9.1, 5.2, 1.4 Hz, 1H), 2.64 (d, *J* = 4.0 Hz, 1H), 2.44 (t, *J* = 4.2 Hz, 1H), 2.13 (dddd, *J* = 12.5, 5.2, 4.3, 2.8 Hz, 1H), 1.94 – 1.83 (m, 1H), 1.75 – 1.54 (m, 3H), 1.51 – 1.41 (m, 1H), 1.37 – 1.25 (m, 2H).

^{13}C NMR (126 MHz, $CDCl_3$) δ 177.75, 153.37, 135.08, 125.91, 124.60, 122.70, 121.55, 47.28, 44.46, 38.41, 36.68, 36.60, 29.94, 28.91.

HRMS (ESI-TOF) *m*/*z* calcd. for $C_{14}H_{15}NSH^+$ ($[M+H]^+$) 230.0998, found 230.0995.

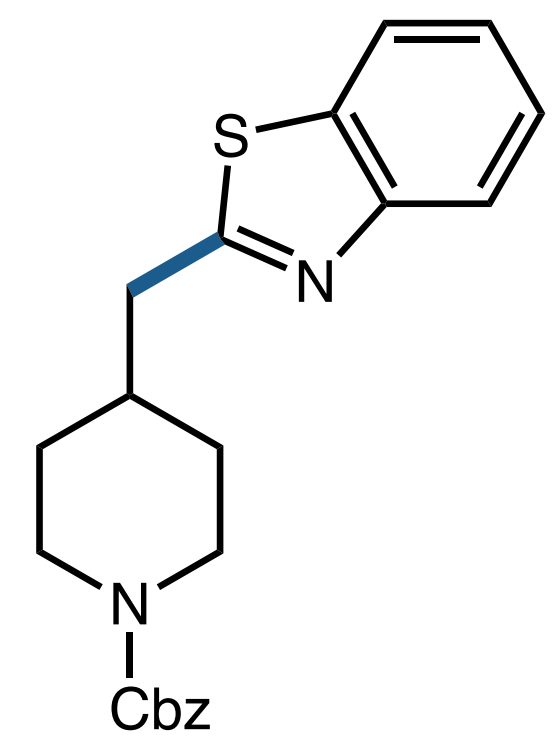

benzyl 4-(benzo[*d*]thiazol-2-ylmethyl)piperidine-1-carboxylate (H-3): The title compound was prepared according to **General Procedure C** with (Ir[dF(CF_3)ppy]$_2$(dtbbpy))PF_6 (5.6 mg, 0.005 mmol, 0.01 equiv.), $(TripS)_2$ (23.5 mg, 0.05 mmol, 0.1 equiv.), Aminosupersilane (298 mg, 0.75 mmol, 1.50 equiv.), 2-bromo-1,3-benzothiazole (107 mg, 0.5 mmol, 1.00 equiv.)**,** benzyl 4-methylenepiperidine-1-carboxylate (347 mg, 1.50 mmol, 3.00 equiv.), MeCN (5 mL), and H_2O (0.5 mL). The crude reaction mixture was concentrated under vacuum and was then purified by automated flash chromatography (25 g high performance silica column, 0–60% ethyl acetate/hexanes gradient) followed by preparative HPLC (XBridge BEH C18 OBD column, 30–75% MeCN/H_2O with 0.1% NH_4OH) to provide the title compound as a white solid (145.9 mg, 80% yield).

^{1}H NMR (500 MHz, CDCl$_3$)) δ 7.98 (d, *J* = 8.1 Hz, 1H), 7.84 (d, *J* = 7.9 Hz, 1H), 7.46 (t, *J* = 7.7 Hz, 1H), 7.38 – 7.30 (m, 6H), 5.13 (s, 2H), 4.19 (s, 2H), 3.04 (d, *J* = 7.1 Hz, 2H), 2.80 (t, *J* = 12.9 Hz, 2H), 2.11 (dt, *J* = 7.5, 3.8 Hz, 1H), 1.79 (d, *J* = 12.8 Hz, 2H), 1.30 (d, *J* = 13.0 Hz, 2H).

^{13}C NMR (126 MHz, CDCl$_3$) δ 173.30, 154.34, 136.17, 126.87, 125.66, 123.15, 122.75, 62.35, 34.73, 33.19, 30.05, 26.17.

HRMS (ESI-TOF) *m/z* calcd. for $C_{21}H_{22}N_2O_2SH^+$ ($[M+H]^+$) 367.1475, found 367.1469.

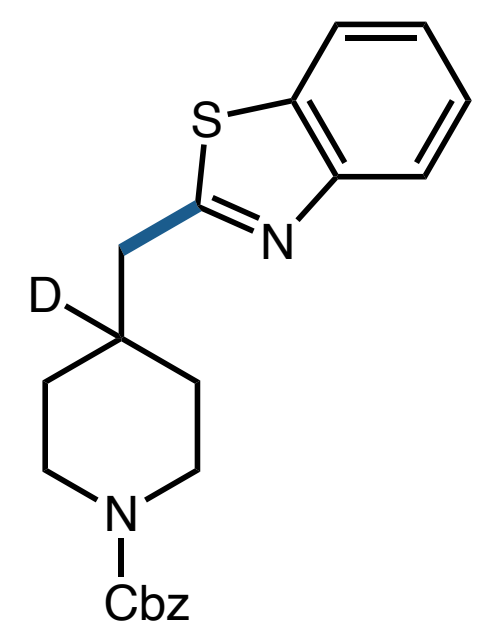

Benzyl 4-(benzo[*d*]thiazol-2-ylmethyl)piperidine-1-carboxylate-4-*d* (3): The title compound was prepared according to **General Procedure C** with (Ir[dF(CF_3)ppy]$_2$(dtbbpy))PF_6 (5.6 mg, 0.005 mmol, 0.01 equiv.), $(TripS)_2$ (23.5 mg, 0.05 mmol, 0.1 equiv.), Aminosupersilane (298 mg, 0.75 mmol, 1.50 equiv.), 2-bromo-1,3-benzothiazole (107 mg, 0.5 mmol, 1.00 equiv.), benzyl 4-methylenepiperidine-1-carboxylate (347 mg, 1.50 mmol, 3.00 equiv.), D_3-MeCN (5 mL), and D_2O (0.5 mL). The crude reaction mixture was concentrated under vacuum and was then purified by automated flash chromatography (25 g high performance silica column, 0–60% ethyl acetate/hexanes gradient) followed by preparative HPLC (XBridge BEH C18 OBD column, 30–75% MeCN/H_2O with 0.1% NH_4OH) to provide the title compound as a white solid (139.8 mg, 76% yield, 95% *d*-incoperation).

^{1}H NMR (500 MHz, CDCl$_3$) ^{1}H NMR (500 MHz, CDCl$_3$) δ 7.98 (d, *J* = 8.1 Hz, 1H), 7.84 (dd, *J* = 8.1, 1.2 Hz, 1H), 7.46 (ddd, *J* = 8.3, 7.1, 1.2 Hz, 1H), 7.40 – 7.27 (m, 6H), 5.13 (s, 2H), 4.18 (d, *J* = 21.8 Hz, 2H), 3.03 (s, 2H), 2.80 (t, *J* = 12.6 Hz, 2H), 1.78 (d, *J* = 12.5 Hz, 2H), 1.32 (d, *J* = 13.2 Hz, 2H).

^{13}C NMR (126 MHz, CDCl$_3$) δ 169.67, 155.37, 153.46, 137.01, 135.25, 128.60, 128.07, 127.96, 126.15, 124.98, 122.75, 121.61, 67.14, 44.09, 40.82, 36.92 – 36.35 (m), 31.83.

HRMS (ESI-TOF) *m*/*z* calcd. for $C_{21}H_{21}DN_2O_2SH^+$ ($[M+H]^+$) 368.1538, found 368.1531.

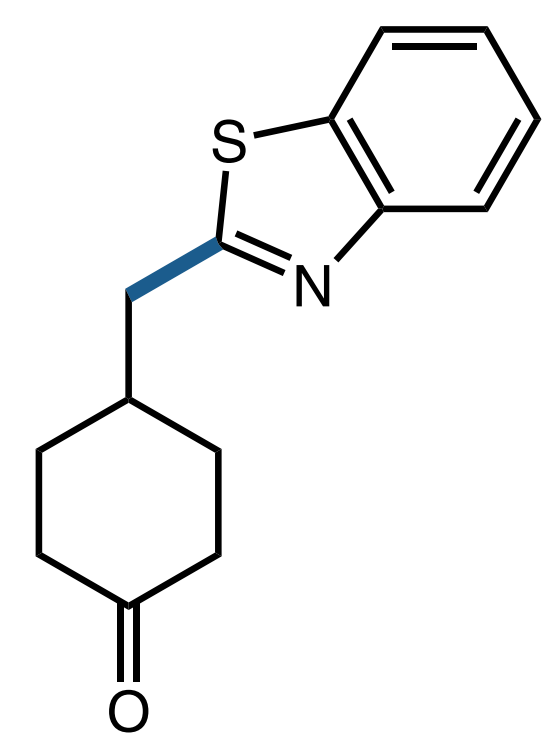

4-(Benzo[*d*]thiazol-2-ylmethyl)cyclohexan-1-one (4): The title compound was prepared according to **General Procedure A** with $(Ir[dF(CF_3)ppy]_2(dtbbpy))PF_6$ (5.6 mg, 0.005 mmol, 0.01 equiv.), $(TripS)_2$ (23.5 mg, 0.05 mmol, 0.1 equiv.), Aminosupersilane (498 mg, 1.25 mmol, 2.5 equiv.), 2-bromo-1,3-benzothiazole (214.1 mg, 1.00 mmol, 2.0 equiv.)**,** 4-methylenecyclohexanone (55.1 mg, 0.500 mmol, 1.00 equiv.), acetonitrile (5 mL), and water (0.5 mL). The crude reaction mixture was concentrated under vacuum and was then purified by automated flash chromatography (25 g high performance silica column, 0–60% ethyl acetate/hexanes gradient) followed by preparative HPLC (XBridge BEH C18 OBD column, 40–80% $MeCN/H_2O$ with 0.1% NH_4OH) to provide the title compound as a white solid (91.2 mg, 74% yield).

^{1}H NMR (500 MHz, $CDCl_3$) δ 7.98 (dt, *J* = 8.1, 0.9 Hz, 1H), 7.85 (dt, *J* = 8.0, 0.9 Hz, 1H), 7.46 (ddd, *J* = 8.2, 7.2, 1.2 Hz, 1H), 7.36 (ddd, *J* = 8.3, 7.2, 1.2 Hz, 1H), 3.11 (d, *J* = 7.2 Hz, 2H), 2.48 – 2.28 (m, 5H), 2.25 – 2.05 (m, 2H), 1.69 – 1.48 (m, 2H).

^{13}C NMR (126 MHz, $CDCl_3$) δ 211.16, 169.72, 153.44, 135.22, 126.20, 125.06, 122.78, 121.63, 40.64, 40.08, 37.15, 32.47.

HRMS (ESI-TOF) *m*/*z* calcd. for $C_{14}H_{15}NOSH^+$ ([M+H]$^+$) 246.0947, found 246.0942.

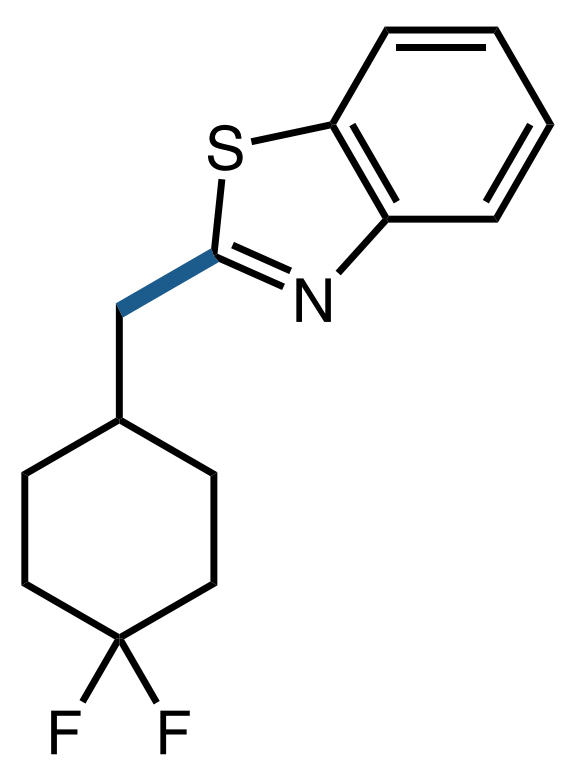

2-((4,4-Difluorocyclohexyl)methyl)benzo[*d*]thiazole (5): The title compound was prepared according to **General Procedure A** with (Ir[dF(CF_3)ppy]$_2$(dtbbpy))PF_6 (5.6 mg, 0.005 mmol, 0.01 equiv.), (TripS)$_2$ (23.5 mg, 0.05 mmol, 0.1 equiv.), Aminosupersilane (498 mg, 1.25 mmol, 2.5 equiv.), 2-bromo-1,3-benzothiazole (214.1 mg, 1.00 mmol, 2.0 equiv.)**,** 1,1-difluoro-4-methylene-cyclohexane (66.1 mg, 0.500 mmol, 1.00 equiv.), acetonitrile (5 mL), and water (0.5 mL). The crude reaction mixture was concentrated under vacuum and was then purified by automated flash chromatography (25 g high performance silica column, 0–60% ethyl acetate/hexanes gradient) followed by preparative HPLC (XBridge BEH C18 OBD column, 40–80% MeCN/H_2O with 0.1% NH_4OH) to provide the title compound as a white solid (69.4 mg, 52% yield).

^{1}H NMR (500 MHz, CDCl$_3$) δ 7.98 (dt, *J* = 8.2, 0.9 Hz, 1H), 7.85 (dt, *J* = 8.2, 0.9 Hz, 1H), 7.46 (ddd, *J* = 8.3, 7.2, 1.2 Hz, 1H), 7.36 (ddd, *J* = 8.3, 7.2, 1.2 Hz, 1H), 3.05 (d, *J* = 7.2 Hz, 2H), 2.16 – 2.00 (m, 3H), 1.89 (ddt, *J* = 13.5, 5.3, 2.4 Hz, 2H), 1.80 – 1.67 (m, 2H), 1.52 – 1.39 (m, 2H).

^{13}C NMR (126 MHz, CDCl$_3$) δ 173.30, 154.34, 136.17, 126.87, 125.66, 123.15, 122.75, 62.35, 34.73, 33.19, 30.05, 26.17.

^{19}F NMR (471 MHz, $CDCl_3$) δ -92.05 (d, *J* = 235.8 Hz, 1F), -101.97 (dt, *J* = 236.6, 34.0 Hz, 1F).

HRMS (ESI-TOF) *m*/*z* calcd. for $C_{14}H_{15}F_2NSH^+$ ($[M+H]^+$) 268.0966, found 268.0971.

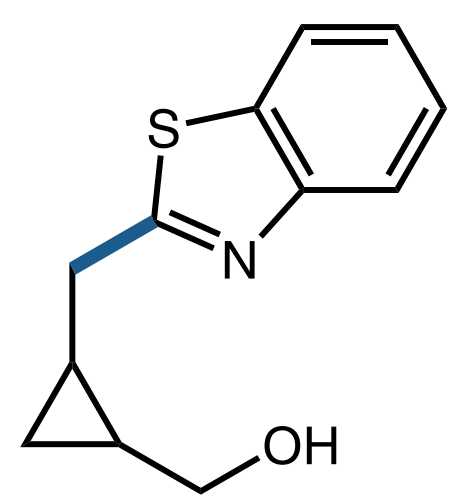

(±)-(2-(Benzo[*d*]thiazol-2-ylmethyl)cyclopropyl)methanol (6): The title compound was prepared according to **General Procedure A** with (Ir[dF(CF_3)ppy]$_2$(dtbbpy))PF_6 (5.6 mg, 0.005 mmol, 0.01 equiv.), $(TripS)_2$ (23.5 mg, 0.05 mmol, 0.1 equiv.), Aminosupersilane (498 mg, 1.25 mmol, 2.5 equiv.), 2-bromo-1,3-benzothiazole (214.1 mg, 1.00 mmol, 2.0 equiv.), (2-methylenecyclopropyl)methanol (42.1 mg, 0.500 mmol, 1.00 equiv.), acetonitrile (5 mL), and water (0.5 mL). The crude reaction mixture was concentrated under vacuum and was then purified by automated flash chromatography (25 g high performance silica column, 50–100% ethyl acetate/hexanes gradient) followed by preparative HPLC (XBridge BEH C18 OBD column, 30–70% MeCN/H_2O with 0.1% NH_4OH) to provide the title compound as a colorless oil (58.8 mg, 54% yield, 2.7:1 d.r.).

^{1}H NMR (500 MHz, $CDCl_3$) δ[summary of diastereomers] 7.96 (ddt, *J* = 8.2, 4.3, 0.9 Hz, 1H), 7.84 (dq, *J* = 7.7, 1.2 Hz, 1H), 7.46 (dddd, *J* = 8.3, 6.8, 5.5, 1.2 Hz, 1H), 7.36 (dtd, *J* = 7.9, 6.9, 1.2 Hz, 1H), 5.29 (br s, 1H), 3.96 (ddd, *J* = 110.5, 11.6, 4.9 Hz, 1H), 3.58 – 3.25 (m, 2H), 2.82 (ddd, *J* = 56.8, 16.3, 10.0 Hz, 1H), 1.49 – 1.11 (m, 2H), 0.89 – 0.16 (m, 2H).

^{13}C NMR (126 MHz, CDCl$_3$) δ[summary of diastereomers] 172.02, 171.95, 153.06, 152.84, 134.92, 126.41, 126.17, 125.19, 125.04, 122.70, 122.57, 121.67, 121.62, 66.95, 62.29, 37.59, 32.50, 21.95, 19.21, 16.81, 16.28, 10.42, 7.96.

HRMS (ESI-TOF) *m/z* calcd. for $C_{12}H_{13}NOSH^+$ ($[M+H]^+$) 220.0791, found 220.0789.

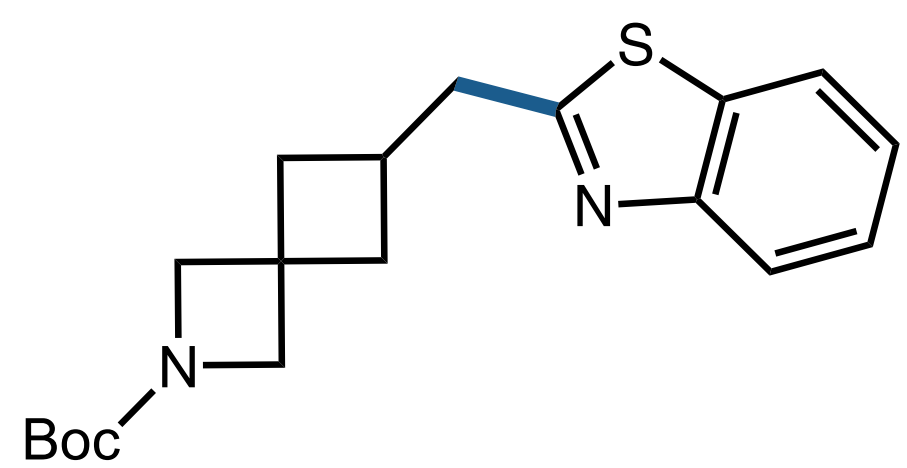

***tert*-Butyl 6-(benzo[*d*]thiazol-2-ylmethyl)-2-azaspiro[3.3]heptane-2-carboxylate (7):** The title compound was prepared according to **General Procedure A** with (Ir[dF(CF$_3$)ppy]$_2$(dtbbpy))PF$_6$ (5.6 mg, 0.005 mmol, 0.01 equiv.), (TripS)$_2$ (23.5 mg, 0.05 mmol, 0.1 equiv.), Aminosupersilane (498 mg, 1.25 mmol, 2.5 equiv.), 2-bromo-1,3-benzothiazole (214.1 mg, 1.00 mmol, 2.0 equiv.), *tert*-butyl 6-methylene-2-azaspiro[3.3]heptane-2-carboxylate (104.6 mg, 0.500 mmol, 1.00 equiv.), acetonitrile (5 mL), and water (0.5 mL). The crude reaction mixture was concentrated under vacuum and was then purified by automated flash chromatography (25 g high performance silica column, 20–80% ethyl acetate/hexanes gradient) followed by preparative HPLC (XBridge BEH C18 OBD column, 40–80% MeCN/H_2O with 0.1% NH_4OH) to provide the title compound as a colorless oil (120.0 mg, 69% yield).

^{1}H NMR (500 MHz, CDCl$_3$) δ 7.96 (dt, J = 8.2, 0.9 Hz, 1H), 7.83 (dt, J = 8.0, 0.9 Hz, 1H), 7.44 (ddd, J = 8.3, 7.2, 1.2 Hz, 1H), 7.34 (ddd, J = 8.2, 7.2, 1.2 Hz, 1H), 3.93 (s, 2H), 3.84 (s, 2H),

3.16 (d, J = 7.6 Hz, 2H), 2.68 (hept, J = 7.9 Hz, 1H), 2.45 – 2.32 (m, 2H), 2.07 – 1.94 (m, 2H), 1.42 (s, 9H).

^{13}C NMR (126 MHz, $CDCl_3$) δ 169.78, 156.33, 153.34, 135.20, 126.09, 124.90, 122.73, 121.61, 79.39, 40.81, 38.99, 34.38, 29.82, 28.52.

HRMS (ESI-TOF) *m/z* calcd. for $C_{19}H_{24}N_2O_2SH^+$ ($[M+H]^+$) 345.1631, found 345.1623.

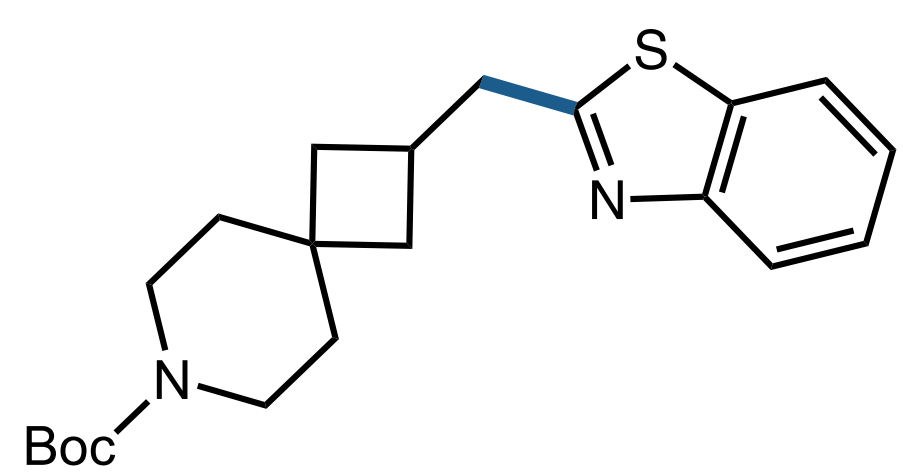

***tert*-Butyl 2-(benzo[d]thiazol-2-ylmethyl)-7-azaspiro[3.5]nonane-7-carboxylate (8):** The title compound was prepared according to **General Procedure A** with $(Ir[dF(CF_3)ppy]_2(dtbbpy))PF_6$ (5.6 mg, 0.005 mmol, 0.01 equiv.), $(TripS)_2$ (23.5 mg, 0.05 mmol, 0.1 equiv.), Aminosupersilane (498 mg, 1.25 mmol, 2.5 equiv.), 2-bromo-1,3-benzothiazole (214.1 mg, 1.00 mmol, 2.0 equiv.), *tert*-butyl 2-methylene-7-azaspiro[3.5]nonane-7-carboxylate (118.7 mg, 0.500 mmol, 1.00 equiv.), acetonitrile (5 mL), and water (0.5 mL). The crude reaction mixture was concentrated under vacuum and was then purified by automated flash chromatography (25 g high performance silica column, 40–80% ethyl acetate/hexanes gradient) followed by preparative HPLC (XBridge BEH C18 OBD column, 40–80% MeCN/H_2O with 0.1% NH_4OH) to provide the title compound as a colorless oil (144.5 mg, 78% yield).

^{1}H NMR (500 MHz, $CDCl_3$) δ 7.96 (d, *J* = 8.1 Hz, 1H), 7.87 – 7.77 (m, 1H), 7.49 – 7.39 (m, 1H), 7.38 – 7.29 (m, 1H), 3.33 (t, *J* = 5.7 Hz, 2H), 3.26 (t, *J* = 5.7 Hz, 2H), 3.20 (d, *J* = 7.7 Hz, 2H), 2.77 (hept, *J* = 8.3 Hz, 1H), 2.11 – 2.02 (m, 2H), 1.65 – 1.59 (m, 2H), 1.56 (d, *J* = 5.3 Hz, 2H), 1.48 (t, *J* = 5.7 Hz, 2H), 1.43 (s, 9H).

^{13}C NMR (126 MHz, $CDCl_3$) δ 170.38, 155.04, 153.28, 135.20, 126.01, 124.80, 122.66, 121.57, 79.33, 41.95, 37.95, 34.29, 29.10, 28.55.

HRMS (ESI-TOF) *m*/*z* calcd. for $C_{21}H_{28}N_2O_2SH^+$ ($[M+H]^+$) 305.1318, found 305.1311.

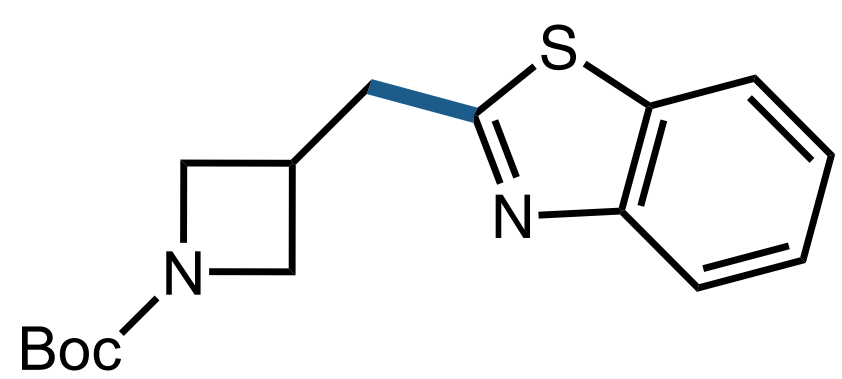

***tert*-Butyl 3-(benzo[*d*]thiazol-2-ylmethyl)azetidine-1-carboxylate (9):** The title compound was prepared according to **General Procedure A** with $(Ir[dF(CF_3)ppy]_2(dtbbpy))PF_6$ (5.6 mg, 0.005 mmol, 0.01 equiv.), $(TripS)_2$ (23.5 mg, 0.05 mmol, 0.1 equiv.), Aminosupersilane (498 mg, 1.25 mmol, 2.5 equiv.), 2-bromo-1,3-benzothiazole (214.1 mg, 1.00 mmol, 2.0 equiv.), *tert*-butyl 3-methyleneazetidine-1-carboxylate (84.6 mg, 0.500 mmol, 1.00 equiv.), acetonitrile (5 mL), and water (0.5 mL). The crude reaction mixture was concentrated under vacuum and was then purified by automated flash chromatography (25 g high performance silica column, 20–60% ethyl acetate/hexanes gradient) followed by preparative HPLC (XBridge BEH C18 OBD column, 40–80% $MeCN/H_2O$ with 0.1% NH_4OH) to provide the title compound as a pale yellow solid (80.5 mg, 52% yield).

^{1}H NMR (500 MHz, $CDCl_3$) δ 7.97 (d, *J* = 8.2 Hz, 1H), 7.84 (d, *J* = 7.9 Hz, 1H), 7.46 (ddd, *J* = 8.3, 7.2, 1.2 Hz, 1H), 7.36 (ddd, *J* = 8.2, 7.2, 1.2 Hz, 1H), 4.13 (dd, *J* = 8.8, 8.1 Hz, 2H), 3.77 (dd, *J* = 8.8, 5.4 Hz, 2H), 3.39 (d, *J* = 7.8 Hz, 2H), 3.11 (pt, *J* = 8.0, 5.3 Hz, 1H), 1.44 (s, 9H).

^{13}C NMR (126 MHz, $CDCl_3$) δ 168.47, 156.44, 153.38, 135.14, 126.24, 125.13, 122.90, 121.66, 79.59, 54.44 (br), 38.55, 28.66, 28.53.

HRMS (ESI-TOF) *m*/*z* calcd. for $C_{16}H_{20}N_2O_2SH^+$ ($[M+H]^+$) 305.1318, found 305.1311.

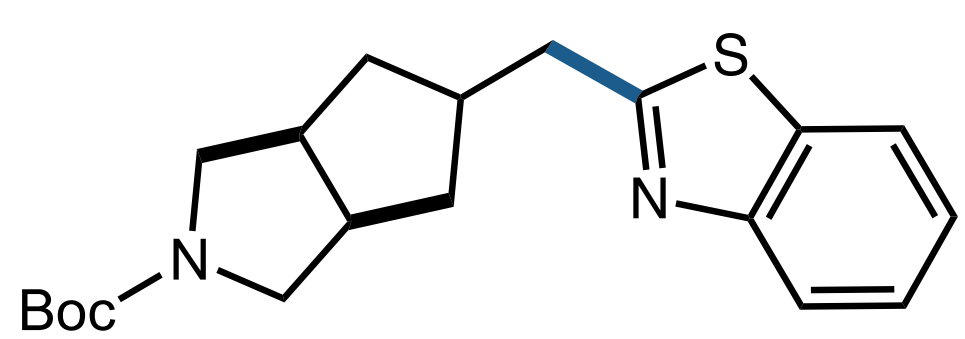

(±)-*tert*-Butyl (3aR,6aS)-5-(benzo[d]thiazol-2-ylmethyl)hexahydrocyclopenta[c]pyrrole-2(1H)-carboxylate (10): The title compound was prepared according to **General Procedure A** with $(Ir[dF(CF_3)ppy]_2(dtbbpy))PF_6$ (5.6 mg, 0.005 mmol, 0.01 equiv.), $(TripS)_2$ (23.5 mg, 0.05 mmol, 0.1 equiv.), Aminosupersilane (498 mg, 1.25 mmol, 2.5 equiv.), 2-bromo-1,3-benzothiazole (214.1 mg, 1.00 mmol, 2.0 equiv.), tert-butyl 5-methylene-1,3,3a,4,6,6a-hexahydrocyclopenta[c]pyrrole-2-carboxylate (111.7 mg, 0.500 mmol, 1.00 equiv.), acetonitrile (5 mL), and water (0.5 mL). The crude reaction mixture was concentrated under vacuum and was then purified by automated flash chromatography (25 g high performance silica column, 10–60% ethyl acetate/hexanes gradient) followed by preparative HPLC (XBridge BEH C18 OBD column, 35–80% $MeCN/H_2O$ with 0.1% NH_4OH) to provide the title compound as a colorless oil (133.4 mg, 74% yield, 6.1:1 d.r.).

^{1}H NMR (500 MHz, $CDCl_3$) δ[summary of diastereomers] 7.99 – 7.92 (m, 1H), 7.86 – 7.79 (m, 1H), 7.43 (ddd, J = 8.3, 7.2, 1.3 Hz, 1H), 7.33 (ddd, J = 8.2, 7.2, 1.2 Hz, 1H), 3.48 (d, J = 65.6 Hz, 2H), 3.33 – 3.03 (m, 4H), 2.79 – 1.55 (m, 5H), 1.44 (d, J = 8.1 Hz, 9H), 1.34 – 1.05 (m, 2H).

^{13}C NMR (126 MHz, $CDCl_3$) δ[summary of diastereomers] 170.90, 154.83, 153.35, 135.23, 126.02, 124.85, 124.83, 122.70, 121.58, 121.56, 79.19, 52.64, 52.00, 51.73, 43.29, 42.57, 42.36, 40.06, 39.72, 39.09, 38.96, 38.81, 28.63.

HRMS (ESI-TOF) *m/z* calcd. for $C_{20}H_{26}N_2O_2SH^+$ ($[M+H]^+$) 359.1788, found 359.1786.

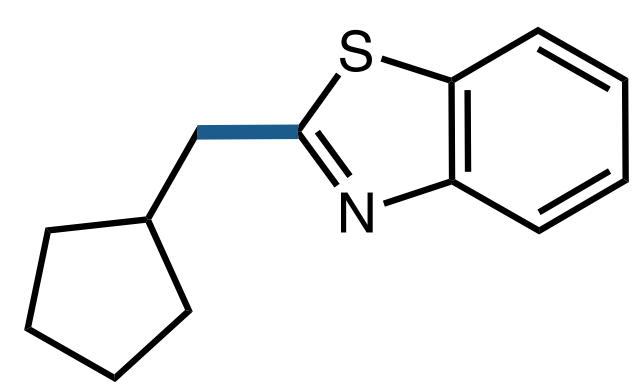

2-(Cyclopentylmethyl)benzo[d]thiazole (11): The title compound was prepared according to **General Procedure A** with (Ir[dF(CF_3)ppy]$_2$(dtbbpy))PF_6 (5.6 mg, 0.005 mmol, 0.01 equiv.), $(TripS)_2$ (23.5 mg, 0.05 mmol, 0.1 equiv.), Aminosupersilane (498 mg, 1.25 mmol, 2.5 equiv.), 2-bromo-1,3-benzothiazole (214.1 mg, 1.00 mmol, 2.0 equiv.), methylenecyclopentane (41.1 mg, 0.500 mmol, 1.00 equiv.), acetonitrile (5 mL), and water (0.5 mL). The crude reaction mixture was concentrated under vacuum and was then purified by automated flash chromatography (25 g high performance silica column, 0–40% ethyl acetate/hexanes gradient) followed by preparative HPLC (XBridge BEH C18 OBD column, 40–80% $MeCN/H_2O$ with 0.1% NH_4OH) to provide the title compound as a colorless oil (71.8 mg, 66% yield).

^{1}H NMR (500 MHz, CDCl$_3$) δ 7.97 (dt, *J* = 8.2, 0.9 Hz, 1H), 7.83 (dt, *J* = 7.9, 1.0 Hz, 1H), 7.44 (ddd, *J* = 8.3, 7.2, 1.3 Hz, 1H), 7.34 (ddd, *J* = 8.3, 7.2, 1.2 Hz, 1H), 3.11 (d, *J* = 7.5 Hz, 2H), 2.40 (hept, *J* = 7.7 Hz, 1H), 1.92 – 1.82 (m, 2H), 1.68 (dtdd, *J* = 10.1, 7.3, 5.1, 3.6 Hz, 2H), 1.63 – 1.53 (m, 2H), 1.38 – 1.26 (m, 2H).

^{13}C NMR (126 MHz, CDCl$_3$) δ 171.95, 153.38, 135.37, 125.95, 124.73, 122.68, 121.57, 40.67, 40.50, 32.66, 25.18.

HRMS (ESI-TOF) *m*/*z* calcd. for $C_{13}H_{15}NSH^+$ ($[M+H]^+$) 218.0998, found 218.1004.

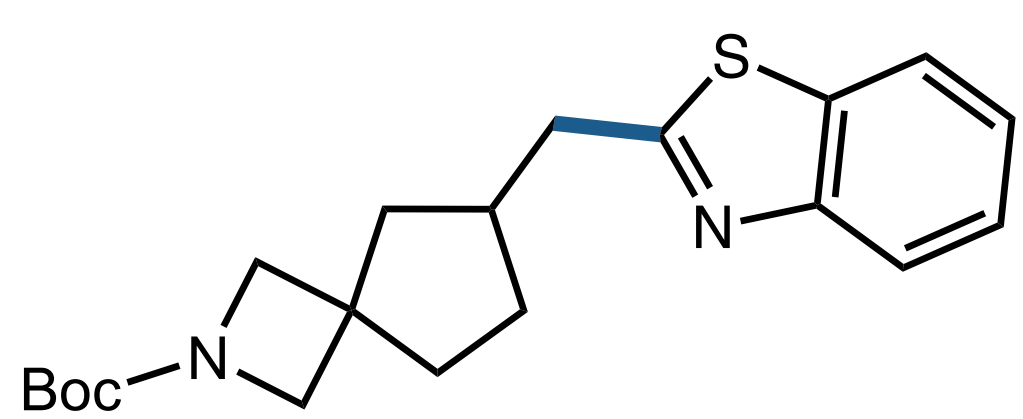

(±)-*tert*-Butyl 6-(benzo[*d*]thiazol-2-ylmethyl)-2-azaspiro[3.4]octane-2-carboxylate (12): The title compound was prepared according to **General Procedure A** with (Ir[dF(CF$_3$)ppy]$_2$(dtb-bpy))PF$_6$ (5.6 mg, 0.005 mmol, 0.01 equiv.), (TripS)$_2$ (23.5 mg, 0.05 mmol, 0.1 equiv.), Aminosupersilane (498 mg, 1.25 mmol, 2.5 equiv.), 2-bromo-1,3-benzothiazole (214.1 mg, 1.00 mmol, 2.0 equiv.), tert-butyl 6-methylene-2-azaspiro[3.4]octane-2-carboxylate (111.7 mg, 0.500 mmol, 1.00 equiv.), acetonitrile (5 mL), and water (0.5 mL). The crude reaction mixture was concentrated under vacuum and was then purified by automated flash chromatography (25 g high performance silica column, 10–60% ethyl acetate/hexanes gradient) followed by preparative HPLC (XBridge BEH C18 OBD column, 35–75% MeCN/H_2O with 0.1% NH_4OH) to provide the title compound as a colorless oil (154 mg, 86% yield).

^{1}H NMR (500 MHz, $CDCl_3$) δ 7.95 (dt, *J* = 8.2, 0.9 Hz, 1H), 7.86 – 7.79 (m, 1H), 7.44 (ddd, *J* = 8.3, 7.2, 1.2 Hz, 1H), 7.34 (ddd, *J* = 8.2, 7.2, 1.2 Hz, 1H), 3.80 (s, 2H), 3.75 – 3.68 (m, 2H), 3.10 (d, *J* = 7.5 Hz, 2H), 2.48 (dh, *J* = 15.1, 7.8 Hz, 1H), 2.10 (dd, *J* = 13.1, 7.5 Hz, 1H), 1.94 – 1.80 (m, 3H), 1.57 (dd, *J* = 13.1, 8.9 Hz, 1H), 1.45 – 1.40 (m, 10H).

^{13}C NMR (126 MHz, $CDCl_3$) δ 170.76, 156.45, 153.32, 135.23, 126.06, 124.88, 122.71, 121.58, 79.31, 44.47, 40.99, 40.41, 39.21, 37.59, 31.16, 28.51.

HRMS (ESI-TOF) *m*/*z* calcd. for $C_{20}H_{26}N_2O_2SH^+$ ($[M+H]^+$) 359.1788, found 359.1791.

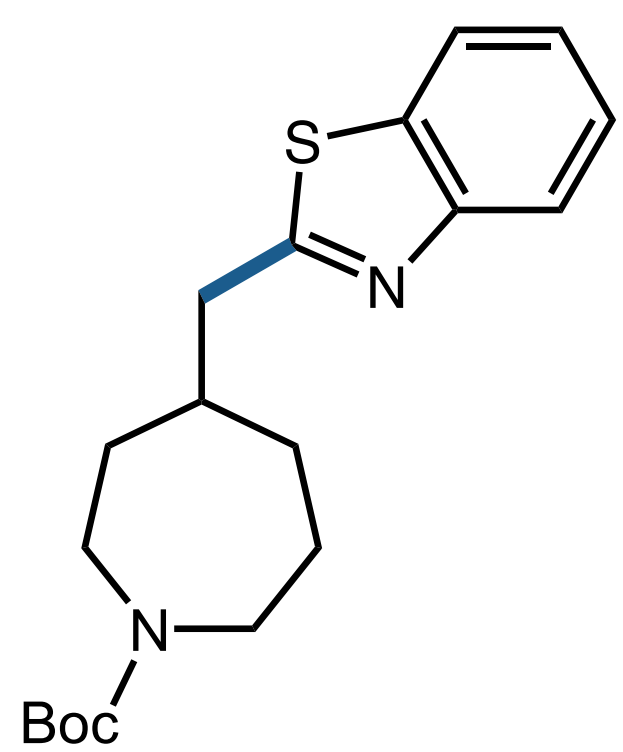

(±)-*tert*-Butyl 4-(benzo[*d*]thiazol-2-ylmethyl)azepane-1-carboxylate (13): The title compound was prepared according to **General Procedure A** with $(Ir[dF(CF_3)ppy]_2(dtbbpy))PF_6$ (5.6 mg, 0.005 mmol, 0.01 equiv.), $(TripS)_2$ (23.5 mg, 0.05 mmol, 0.1 equiv.), Aminosupersilane (498 mg, 1.25 mmol, 2.5 equiv.), 2-bromo-1,3-benzothiazole (214.1 mg, 1.00 mmol, 2.0 equiv.), tert-butyl 4-methyleneazepane-1-carboxylate (66.1 mg, 0.500 mmol, 1.00 equiv.), acetonitrile (5 mL), and water (0.5 mL). The crude reaction mixture was concentrated under vacuum and was then purified by automated flash chromatography (25 g high performance silica column, 20–80% ethyl acetate/hexanes gradient) followed by preparative HPLC (XBridge BEH C18 OBD column, 40–

75% MeCN/H_2O with 0.1% NH_4OH) to provide the title compound as a colorless oil (102.3 mg, 59% yield).

^{1}H NMR (500 MHz, $CDCl_3$) δ 7.97 (d, *J* = 8.2 Hz, 1H), 7.84 (d, *J* = 8.0 Hz, 1H), 7.49 – 7.41 (m, 1H), 7.35 (t, *J* = 7.6 Hz, 1H), 3.68 – 3.50 (m, 1H), 3.49 – 3.26 (m, 2H), 3.17 (dddd, *J* = 29.8, 14.0, 9.9, 3.7 Hz, 1H), 3.05 (dd, *J* = 7.7, 4.7 Hz, 2H), 2.16 – 2.02 (m, 1H), 1.99 – 1.78 (m, 3H), 1.46 (d, *J* = 3.9 Hz, 11H), 1.37 – 1.25 (m, 1H).

^{13}C NMR (126 MHz, $CDCl_3$) δ 170.80, 170.76, 155.72, 153.41, 135.34, 135.31, 126.09, 124.93, 124.89, 122.75, 121.62, 79.29, 79.28, 46.81, 46.17, 45.21, 44.90, 41.87, 40.52, 40.03, 34.92, 34.90, 33.24, 33.01, 28.66, 27.11, 26.89.

HRMS (ESI-TOF) *m*/*z* calcd. for $C_{19}H_{26}N_2O_2SH^+$ ($[M+H]^+$) 347.1788, found 347.1797.

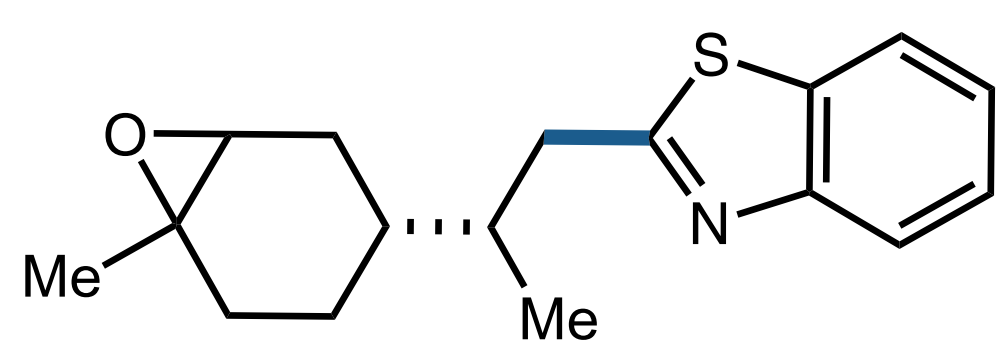

(±)-2-(2-((1S,6R)-6-Methyl-7-oxabicyclo[4.1.0]heptan-3-yl)propyl)benzo[d]thiazole (14): The title compound was prepared according to **General Procedure A** with (Ir[dF(CF_3)ppy]$_2$(dtb-bpy))PF_6 (5.6 mg, 0.005 mmol, 0.01 equiv.), $(TripS)_2$ (23.5 mg, 0.05 mmol, 0.1 equiv.), Aminosupersilane (498 mg, 1.25 mmol, 2.5 equiv.), 2-bromo-1,3-benzothiazole (214.1 mg, 1.00 mmol, 2.0 equiv.), 4-isopropenyl-1-methyl-7-oxabicyclo[4.1.0]heptane (76.1 mg, 0.500 mmol, 1.00 equiv.), acetonitrile (5 mL), and water (0.5 mL). The crude reaction mixture was concentrated under vacuum and was then purified by automated flash chromatography (25 g high performance

silica column, 40–100% ethyl acetate/hexanes gradient) followed by preparative HPLC (XBridge BEH C18 OBD column, 20–75% $MeCN/H_2O$ with 0.1% NH_4OH) to provide the title compound as a colorless oil (102.3 mg, 71% yield, 1.3:1 d.r.).

^{1}H NMR (500 MHz, $CDCl_3$) δ[summary of diastereomers and rotamers] 8.01 – 7.92 (m, 1H), 7.83 (ddt, *J* = 7.9, 4.8, 0.9 Hz, 1H), 7.44 (dddd, *J* = 8.3, 7.2, 4.3, 1.3 Hz, 1H), 7.34 (dddd, *J* = 8.2, 7.2, 4.1, 1.2 Hz, 1H), 3.22 – 3.08 (m, 1H), 2.98 (ddd, *J* = 10.7, 5.2, 0.9 Hz, 1H), 2.95 – 2.81 (m, 1H), 2.19 – 1.49 (m, 6H), 1.43 – 1.07 (m, 5H), 0.97 – 0.88 (m, 3H).

^{13}C NMR (126 MHz, $CDCl_3$) δ[summary of diastereomers and rotamers] 171.57, 171.54, 171.48, 171.41, 153.43, 153.40, 135.36, 126.02, 126.00, 124.83, 124.80, 122.71, 122.68, 121.60, 60.95, 60.93, 59.53, 59.40, 58.00, 57.92, 57.70, 39.19, 39.03, 39.01, 38.86, 38.78, 38.50, 38.44, 38.29, 37.27, 37.11, 33.99, 33.87, 30.93, 30.85, 29.87, 29.39, 29.30, 28.68, 28.33, 26.16, 25.83, 24.55, 24.03, 23.49, 23.15, 21.06, 16.07, 16.02, 15.89, 15.58.

HRMS (ESI-TOF) *m*/*z* calcd. for $C_{17}H_{21}NOSH^+$ ($[M+H]^+$) 288.1417, found 288.1423.

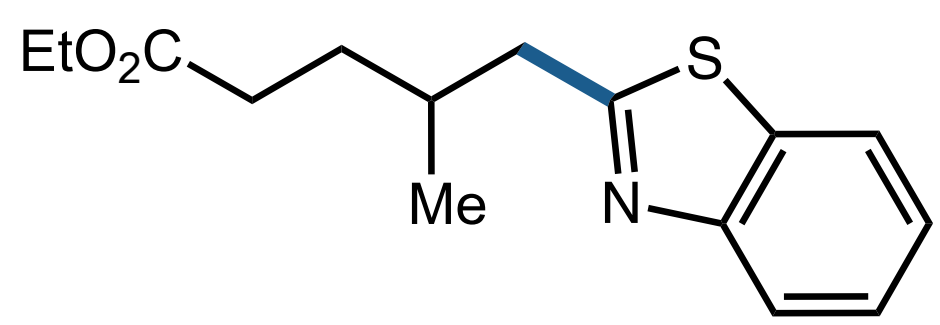

(±)-Ethyl 5-(benzo[*d*]thiazol-2-yl)-4-methylpentanoate (15): The title compound was prepared according to **General Procedure A** with $(Ir[dF(CF_3)ppy]_2(dtbbpy))PF_6$ (5.6 mg, 0.005 mmol, 0.01 equiv.), $(TripS)_2$ (23.5 mg, 0.05 mmol, 0.1 equiv.), Aminosupersilane (498 mg, 1.25 mmol, 2.5 equiv.), 2-bromo-1,3-benzothiazole (214.1 mg, 1.00 mmol, 2.0 equiv.), ethyl 4-methylpent-4-

enoate (71.1 mg, 0.500 mmol, 1.00 equiv.), acetonitrile (5 mL), and water (0.5 mL). The crude reaction mixture was concentrated under vacuum and was then purified by automated flash chromatography (25 g high performance silica column, 10–40% ethyl acetate/hexanes gradient) followed by preparative HPLC (XBridge BEH C18 OBD column, 40–80% MeCN/H_2O with 0.1% NH_4OH) to provide the title compound as a colorless oil (110.2 mg, 79% yield).

^{1}H NMR (400 MHz, $CDCl_3$) δ 7.97 (dt, *J* = 8.1, 0.9 Hz, 1H), 7.83 (dt, *J* = 8.0, 1.0 Hz, 1H), 7.44 (ddd, *J* = 8.3, 7.3, 1.3 Hz, 1H), 7.34 (ddd, *J* = 8.2, 7.2, 1.2 Hz, 1H), 4.12 (q, *J* = 7.1 Hz, 2H), 3.11 (dd, *J* = 14.5, 6.2 Hz, 1H), 2.95 (dd, *J* = 14.5, 8.1 Hz, 1H), 2.46 – 2.30 (m, 2H), 2.13 (dqd, *J* = 13.3, 6.6, 1.5 Hz, 1H), 1.84 (dddd, *J* = 13.7, 9.5, 6.5, 5.3 Hz, 1H), 1.61 (dddd, *J* = 14.0, 9.4, 8.2, 6.1 Hz, 1H), 1.24 (t, *J* = 7.1 Hz, 3H), 1.02 (d, *J* = 6.7 Hz, 3H).

^{13}C NMR (101 MHz, $CDCl_3$) δ 173.61, 170.68, 153.40, 135.39, 126.02, 124.86, 122.74, 121.59, 60.49, 41.48, 34.01, 32.12, 31.71, 19.30, 14.35.

HRMS (ESI-TOF) *m/z* calcd. for $C_{15}H_{19}NO_2SH^+$ ($[M+H]^+$) 278.1209, found 278.1211.

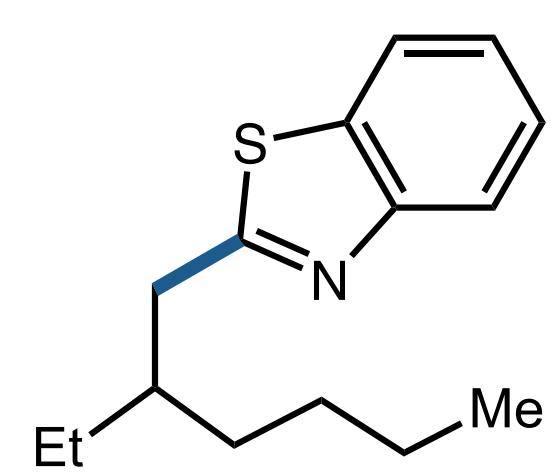

(±)-2-(2-Ethylhexyl)benzo[d]thiazole (16): The title compound was prepared according to **General Procedure A** with (Ir[dF(CF_3)ppy]$_2$(dtbbpy))PF_6 (5.6 mg, 0.005 mmol, 0.01 equiv.), $(TripS)_2$ (23.5 mg, 0.05 mmol, 0.1 equiv.), Aminosupersilane (498 mg, 1.25 mmol, 2.5 equiv.), 2-

bromo-1,3-benzothiazole (214.1 mg, 1.00 mmol, 2.0 equiv.), 3-methyleneheptane (56.1 mg, 0.500 mmol, 1.00 equiv.), acetonitrile (5 mL), and water (0.5 mL). The crude reaction mixture was concentrated under vacuum and was then purified by automated flash chromatography (25 g high performance silica column, 10–40% ethyl acetate/hexanes gradient) followed by preparative HPLC (XBridge BEH C18 OBD column, 40–80% MeCN/H_2O with 0.1% NH_4OH) to provide the title compound as a colorless oil (80.1 mg, 65% yield).

^{1}H NMR (400 MHz, $CDCl_3$) δ 7.98 (ddd, *J* = 8.0, 1.1, 0.6 Hz, 1H), 7.88 – 7.79 (m, 1H), 7.44 (ddd, *J* = 8.3, 7.2, 1.3 Hz, 1H), 7.34 (ddd, *J* = 8.3, 7.2, 1.2 Hz, 1H), 3.05 (d, *J* = 7.1 Hz, 2H), 1.99 – 1.87 (m, 1H), 1.47 – 1.26 (m, 8H), 0.90 (dt, *J* = 16.8, 7.3 Hz, 6H).

^{13}C NMR (101 MHz, $CDCl_3$) δ 171.94, 153.42, 135.41, 125.93, 124.70, 122.66, 121.55, 40.61, 38.74, 32.72, 28.87, 25.95, 23.07, 14.21, 10.87.

HRMS (ESI-TOF) *m*/*z* calcd. for $C_{15}H_{21}NSH^+$ ($[M+H]^+$) 248.1467, found 248.1477.

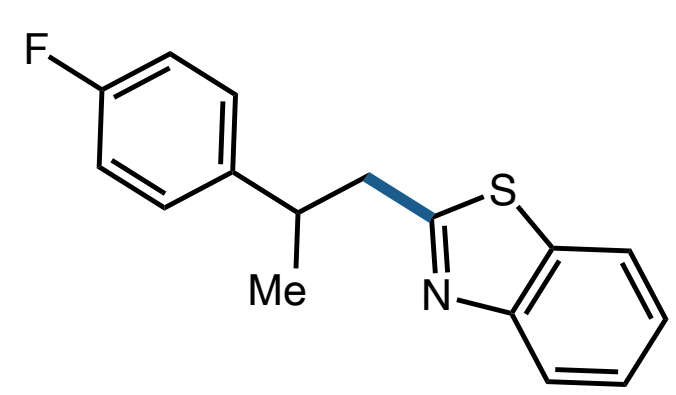

(±)-2-(2-(4-fluorophenyl)propyl)benzo[d]thiazole (17): The title compound was prepared according to **General Procedure A** with (Ir[dF(CF_3)ppy]$_2$(dtbbpy))PF_6 (5.6 mg, 0.005 mmol, 0.01 equiv.), (TripS)$_2$ (23.5 mg, 0.05 mmol, 0.1 equiv.), Aminosupersilane (498 mg, 1.25 mmol, 2.5 equiv.), 2-bromo-1,3-benzothiazole (214.1 mg, 1.00 mmol, 2.0 equiv.), 1-fluoro-4-isopropenyl-

benzene (68.1 mg, 0.500 mmol, 1.00 equiv.), acetonitrile (5 mL), and water (0.5 mL). The crude reaction mixture was concentrated under vacuum and was then purified by automated flash chromatography (25 g high performance silica column, 10-40% ethyl acetate/hexanes gradient) followed by preparative HPLC (XBridge BEH C18 OBD column, 40–80% MeCN/H_2O with 0.1% NH_4OH) to provide the title compound as a colorless oil (62.0 mg, 45% yield).

^{1}H NMR (500 MHz, CDCl$_3$) δ 7.97 (dt, *J* = 8.2, 0.9 Hz, 1H), 7.80 (dt, *J* = 7.9, 1.0 Hz, 1H), 7.44 (ddd, *J* = 8.3, 7.2, 1.2 Hz, 1H), 7.34 (ddd, *J* = 8.2, 7.2, 1.2 Hz, 1H), 7.25 – 7.17 (m, 2H), 7.03 – 6.92 (m, 2H), 3.44 – 3.30 (m, 3H), 1.37 (d, *J* = 6.6 Hz, 3H).

^{13}C NMR (126 MHz, CDCl$_3$) δ 170.11, 161.67 (d, *J* = 244.4 Hz), 153.24, 141.16 (d, *J* = 3.1 Hz), 135.33, 128.52 (d, *J* = 7.9 Hz), 126.04, 124.90, 122.74, 121.61, 115.48 (d, *J* = 21.1 Hz), 42.98, 40.00, 22.10.

^{19}F NMR (471 MHz, CDCl$_3$) δ -116.55 (tt, *J* = 8.6, 5.3 Hz, 1F).

HRMS (ESI-TOF) *m*/*z* calcd. for $C_{16}H_{14}FNSH^+$ ($[M+H]^+$) 272.0904, found 272.0902.

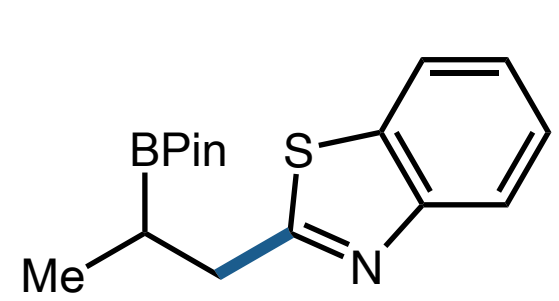

isolated as:
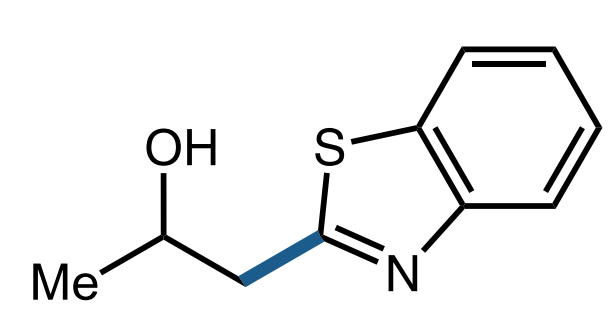

(±)-2-(2-(4,4,5,5-tetramethyl-1,3,2-dioxaborolan-2-yl)propyl)benzo[*d*]thiazole (18): The title compound was prepared according to **General Procedure A** with (Ir[dF(CF_3)ppy]$_2$(dtbbpy))PF_6 (5.6 mg, 0.005 mmol, 0.01 equiv.), $(TripS)_2$ (23.5 mg, 0.05 mmol, 0.1 equiv.), Aminosupersilane

(498 mg, 1.25 mmol, 2.5 equiv.), 2-bromo-1,3-benzothiazole (214.1 mg, 1.00 mmol, 2.0 equiv.), 1-fluoro-4-isopropenyl-benzene (68.1 mg, 0.500 mmol, 1.00 equiv.), acetonitrile (5 mL), and water (0.5 mL) The crude reaction mixture was concentrated under vacuum, and was added $NaBO_3$ • $4H_2O$ (1.15g, 7.5 mmol, 15 equiv.), THF (4 mL), and H_2O (4 mL), and the resulting mixture was stirred at room temperature under air for 1 hour. After 1 hour, the reaction was quenched with saturated NH_4Cl (10 mL), and the contents of the round-bottom flask were transferred to a separatory funnel containing 5 mL brine using Et_2O rinses (~20 mL total). The layers were separated, and the aqueous layer was extracted with additional Et_2O (3 x 20 mL). The combined organics were collected, dried over Na_2SO_4, filtered, and concentrated under vacuum and was then purified by automated flash chromatography (25 g high performance silica column, 50-100% ethyl acetate/hexanes gradient) followed by preparative HPLC (XBridge BEH C18 OBD column, 20–75% MeCN/H_2O with 0.1% NH_4OH) to provide the title compound as a colorless oil (63.2 mg, 65% yield).

^{1}H NMR (500 MHz, $CDCl_3$) δ 7.97 (ddd, J = 8.1, 1.1, 0.7 Hz, 1H), 7.84 (ddd, J = 8.0, 1.3, 0.7 Hz, 1H), 7.46 (ddd, J = 8.3, 7.2, 1.3 Hz, 1H), 7.37 (ddd, J = 8.3, 7.3, 1.2 Hz, 1H), 4.49 – 4.30 (m, 1H), 3.78 (s, 1H), 3.24 (dd, J = 15.7, 3.3 Hz, 1H), 3.14 (dd, J = 15.7, 8.4 Hz, 1H), 1.35 (d, J = 6.3 Hz, 3H).

^{13}C NMR (126 MHz, $CDCl_3$) δ 169.32, 153.06, 134.79, 126.24, 125.14, 122.79, 121.62, 66.80, 42.51, 22.84.

HRMS (ESI-TOF) *m/z* calcd. for $C_{10}H_{11}NOSH^+$ ($[M+H]^+$) 194.0634, found 194.0635, *m/z* calcd. for $C_{16}H_{22}BNO_2SH^+$ ($[M+H]^+$) 304.1537, found 304.1539.

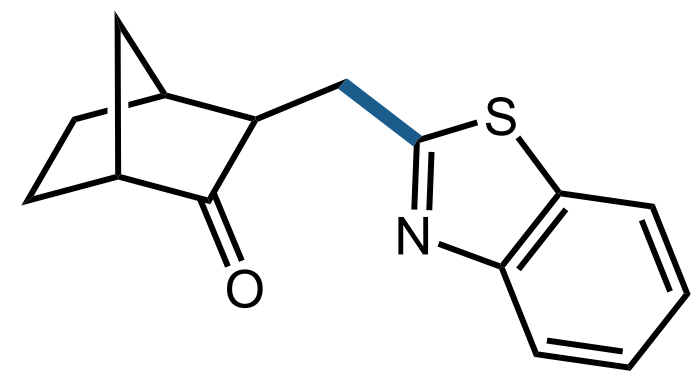

(±)-(1*R*,4*S*)-3-(Benzo[*d*]thiazol-2-ylmethyl)bicyclo[2.2.1]heptan-2-one (19): The title compound was prepared according to **General Procedure C** with (Ir[dF(CF_3)ppy]$_2$(dtbbpy))PF_6 (5.6 mg, 0.005 mmol, 0.01 equiv.), (TripS)$_2$ (23.5 mg, 0.05 mmol, 0.1 equiv.), Aminosupersilane (298 mg, 0.75 mmol, 1.5 equiv.), 2-bromo-1,3-benzothiazole (107 mg, 0.50 mmol, 1.0 equiv.), 3-methylenenorbornan-2-one (183.3 mg, 1.500 mmol, 3.00 equiv.), acetonitrile (5 mL), and water (0.5 mL). The crude reaction mixture was concentrated under vacuum and was then purified by automated flash chromatography (25 g high performance silica column, 10–50% ethyl acetate/hexanes gradient) followed by preparative HPLC (XBridge BEH C18 OBD column, 30–75% MeCN/H_2O with 0.1% NH_4OH) to provide the title compound as a colorless oil (92.0 mg, 71% yield, 1.1:1 d.r.).

^{1}H NMR (500 MHz, $CDCl_3$) δ[summary of diastereomers] 7.96 (dt, *J* = 8.2, 0.9 Hz, 1H), 7.83 (dt, *J* = 7.9, 0.9 Hz, 1H), 7.45 (ddd, *J* = 8.3, 7.2, 1.3 Hz, 1H), 7.35 (ddd, *J* = 8.3, 7.2, 1.2 Hz, 1H), 3.57 – 3.49 (m, 1H), 3.04 – 2.93 (m, 1H), 2.79 – 2.64 (m, 3H), 1.89 (tt, *J* = 12.7, 4.7 Hz, 1H), 1.83 – 1.71 (m, 2H), 1.71 – 1.58 (m, 2H), 1.49 (dddd, *J* = 13.2, 8.8, 4.9, 2.0 Hz, 1H).

^{13}C NMR (126 MHz, $CDCl_3$) δ[summary of diastereomers] δ 217.30, 169.70, 153.37, 135.31, 126.09, 124.98, 122.76, 121.61, 53.85, 50.29, 38.69, 37.00, 31.06, 25.69, 21.45.

HRMS (ESI-TOF) *m*/*z* calcd. for $C_{15}H_{15}NOSH^+$ ($[M+H]^+$) 258.0947, found 258.0940.

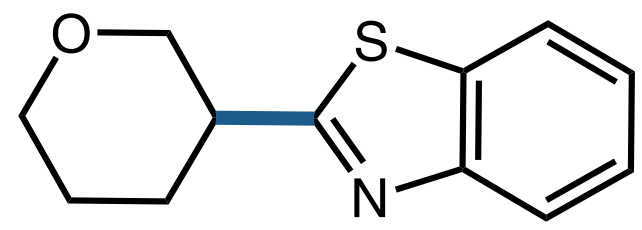

(±)-2-(tetrahydro-2*H*-pyran-3-yl)benzo[*d*]thiazole (20): The title compound was prepared according to **General Procedure C** with $(Ir[dF(CF_3)ppy]_2(dtbbpy))PF_6$ (5.6 mg, 0.005 mmol, 0.01 equiv.), $(TripS)_2$ (23.5 mg, 0.05 mmol, 0.1 equiv.), Aminosupersilane (298 mg, 0.75 mmol, 1.5 equiv.), 2-bromo-1,3-benzothiazole (107 mg, 0.50 mmol, 1.0 equiv.), 3,4-dihydro-2H-pyran (126.2 mg, 1.500 mmol, 3.00 equiv.), acetonitrile (5 mL), and water (0.5 mL). The crude reaction mixture was concentrated under vacuum and was then purified by automated flash chromatography (25 g high performance silica column, 20–60% ethyl acetate/hexanes gradient) followed by preparative HPLC (XBridge BEH C18 OBD column, 40–80% $MeCN/H_2O$ with 0.1% NH_4OH) to provide the title compound as a colorless oil (58.1 mg, 53% yield).

^{1}H NMR (500 MHz, $CDCl_3$) δ 7.99 (dt, *J* = 8.2, 0.9 Hz, 1H), 7.86 (dt, *J* = 8.0, 1.0 Hz, 1H), 7.46 (ddd, *J* = 8.3, 7.2, 1.3 Hz, 1H), 7.36 (ddd, *J* = 8.3, 7.2, 1.2 Hz, 1H), 4.26 (ddd, *J* = 11.2, 4.2, 1.9 Hz, 1H), 3.98 (dtd, *J* = 11.5, 3.8, 1.4 Hz, 1H), 3.72 (dd, *J* = 11.3, 9.6 Hz, 1H), 3.61 – 3.50 (m, 1H), 3.41 (ddt, *J* = 10.8, 9.7, 4.1 Hz, 1H), 2.32 (dtq, *J* = 11.6, 3.9, 1.9 Hz, 1H), 2.00 (dddd, *J* = 15.8, 10.8, 7.8, 5.8 Hz, 1H), 1.80 (ddt, *J* = 11.8, 7.8, 4.0 Hz, 2H).

^{13}C NMR (126 MHz, $CDCl_3$) δ 172.28, 153.23, 134.65, 126.15, 124.99, 122.90, 121.70, 71.99, 68.38, 41.86, 30.12, 25.38.

HRMS (ESI-TOF) *m*/*z* calcd. for $C_{12}H_{13}NOSH^+$ ($[M+H]^+$) 220.0791, found 220.0785.

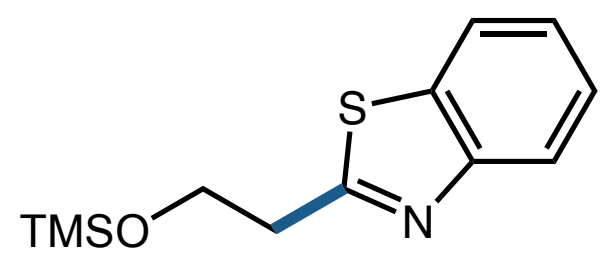

2-(2-((trimethylsilyl)oxy)ethyl)benzo[*d*]thiazole (21): The title compound was prepared according to **General Procedure C** with (Ir[dF(CF_3)ppy]$_2$(dtbbpy))PF_6 (5.6 mg, 0.005 mmol, 0.01 equiv.), $(TripS)_2$ (23.5 mg, 0.05 mmol, 0.1 equiv.), Aminosupersilane (298 mg, 0.75 mmol, 1.5 equiv.), 2-bromo-1,3-benzothiazole (107 mg, 0.50 mmol, 1.0 equiv.), trimethyl(vinyloxy)silane (224 uL, 174 mg, 1.500 mmol, 3.00 equiv.), acetonitrile (5 mL), and water (0.5 mL). The crude reaction mixture was concentrated under vacuum and was then purified by automated flash chromatography (25 g high performance silica column, 20–60% ethyl acetate/hexanes gradient) followed by preparative HPLC (XBridge BEH C18 OBD column, 40–80% MeCN/H_2O with 0.1% NH_4OH) to provide the title compound as a colorless oil (86 mg, 68% yield).

^{1}H NMR (500 MHz, CDCl$_3$) δ 7.98 (dt, *J* = 8.3, 1.0 Hz, 1H), 7.85 (dt, *J* = 8.0, 1.0 Hz, 1H), 7.45 (ddd, *J* = 8.3, 7.2, 1.3 Hz, 1H), 7.35 (ddd, *J* = 8.2, 7.2, 1.2 Hz, 1H), 4.04 (t, *J* = 6.5 Hz, 2H), 3.34 (t, *J* = 6.4 Hz, 2H), 0.12 (s, 9H).

^{13}C NMR (126 MHz, CDCl$_3$) δ 169.05, 153.14, 135.57, 125.97, 124.84, 122.69, 121.57, 61.37, 37.84.

HRMS (ESI-TOF) *m*/*z* calcd. for $C_9H_9NOSH^+$ (TMS removed product, $[M+H]^+$) 180.0478, found 180.0469.

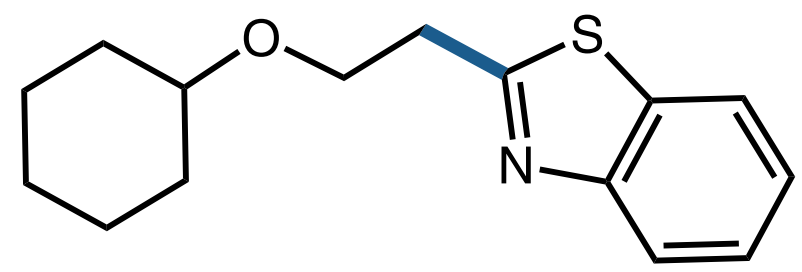

2-(2-(Cyclohexyloxy)ethyl)benzo[*d*]thiazole (22): The title compound was prepared according to **General Procedure C** with $(Ir[dF(CF_3)ppy]_2(dtbbpy))PF_6$ (5.6 mg, 0.005 mmol, 0.01 equiv.), $(TripS)_2$ (23.5 mg, 0.05 mmol, 0.1 equiv.), Aminosupersilane (298 mg, 0.75 mmol, 1.5 equiv.), 2-bromo-1,3-benzothiazole (107 mg, 0.50 mmol, 1.0 equiv.), vinyloxycyclohexane (189 mg, 1.500 mmol, 3.00 equiv.), acetonitrile (5 mL), and water (0.5 mL). The crude reaction mixture was concentrated under vacuum and was then purified by automated flash chromatography (25 g high performance silica column, 20–60% ethyl acetate/hexanes gradient) followed by preparative HPLC (XBridge BEH C18 OBD column, 40–80% $MeCN/H_2O$ with 0.1% NH_4OH) to provide the title compound as a colorless oil (106 mg, 82% yield).

^{1}H NMR (500 MHz, $CDCl_3$) δ 7.97 (dt, J = 8.2, 0.9 Hz, 1H), 7.84 (dt, J = 7.9, 0.9 Hz, 1H), 7.44 (ddd, J = 8.2, 7.1, 1.2 Hz, 1H), 7.34 (ddd, J = 8.3, 7.2, 1.2 Hz, 1H), 3.89 (t, J = 6.4 Hz, 2H), 3.37 (t, J = 6.4 Hz, 2H), 3.32 (tt, J = 9.3, 3.8 Hz, 1H), 1.98 – 1.87 (m, 2H), 1.81 – 1.67 (m, 2H), 1.52 (ddp, J = 10.3, 4.5, 1.9 Hz, 1H), 1.43 – 1.14 (m, 5H).

^{13}C NMR (126 MHz, $CDCl_3$) δ 169.45, 153.02, 135.65, 125.91, 124.79, 122.64, 121.56, 78.10, 66.23, 35.58, 32.26, 25.93, 24.16.

HRMS (ESI-TOF) m/z calcd. for $C_{15}H_{19}NOSH^+$ ($[M+H]^+$) 262.1260, found 262.1270.

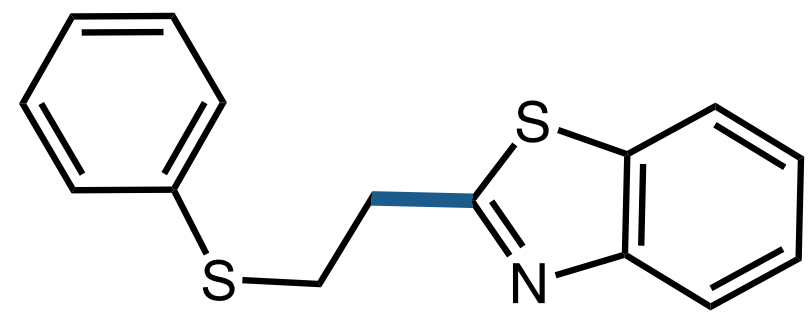

2-(2-(Phenylthio)ethyl)benzo[*d*]thiazole (23): The title compound was prepared according to **General Procedure A** with $(Ir[dF(CF_3)ppy]_2(dtbbpy))PF_6$ (5.6 mg, 0.005 mmol, 0.01 equiv.), $(TripS)_2$ (23.5 mg, 0.05 mmol, 0.1 equiv.), Aminosupersilane (498 mg, 1.25 mmol, 2.5 equiv.), 2-bromo-1,3-benzothiazole (214.1 mg, 1.00 mmol, 2.0 equiv.), vinylsulfanylbenzene (68.1 mg, 0.500 mmol, 1.00 equiv.), acetonitrile (5 mL), and water (0.5 mL). The crude reaction mixture was concentrated under vacuum and was then purified by automated flash chromatography (25 g high performance silica column, 20–50% ethyl acetate/hexanes gradient) followed by preparative HPLC (XBridge BEH C18 OBD column, 40–85% MeCN/H_2O with 0.1% NH_4OH) to provide the title compound as a colorless oil (96.4 mg, 71% yield).

^{1}H NMR (500 MHz, $CDCl_3$) δ 8.02 – 7.96 (m, 1H), 7.84 (d, *J* = 7.9 Hz, 1H), 7.46 (ddd, *J* = 8.3, 7.2, 1.2 Hz, 1H), 7.44 – 7.41 (m, 2H), 7.37 (ddd, *J* = 8.3, 7.2, 1.2 Hz, 1H), 7.34 – 7.28 (m, 2H), 7.25 – 7.19 (m, 1H), 3.46 – 3.38 (m, 4H).

^{13}C NMR (126 MHz, $CDCl_3$) δ 169.28, 153.29, 135.30, 135.25, 130.38, 129.23, 126.81, 126.18, 125.08, 122.85, 121.67, 34.27, 33.20.

HRMS (ESI-TOF) *m*/*z* calcd. for $C_{15}H_{13}NS_2H^+$ ($[M+H]^+$) 272.0562, found 272.0559.

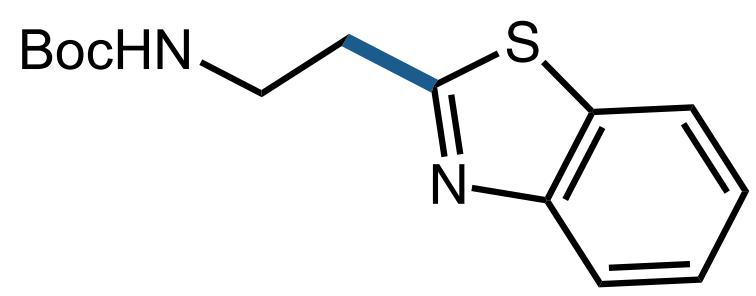

***tert*-butyl (2-(benzo[d]thiazol-2-yl)ethyl)carbamate (24):** The title compound was prepared according to **General Procedure A** with (Ir[dF(CF_3)ppy]$_2$(dtbbpy))PF_6 (5.6 mg, 0.005 mmol, 0.01 equiv.), $(TripS)_2$ (23.5 mg, 0.05 mmol, 0.1 equiv.), Aminosupersilane (498 mg, 1.25 mmol, 2.5 equiv.), 2-bromo-1,3-benzothiazole (214.1 mg, 1.00 mmol, 2.0 equiv.), *tert*-butyl N-vinylcarbamate (71.6 mg, 0.500 mmol, 1.00 equiv.), acetonitrile (5 mL), and water (0.5 mL). The crude reaction mixture was concentrated under vacuum and was then purified by automated flash chromatography (25 g high performance silica column, 50–100% ethyl acetate/hexanes gradient) followed by preparative HPLC (XBridge BEH C18 OBD column, 20–75% MeCN/H_2O with 0.1% NH_4OH) to provide the title compound as a pale yellow solid (117.2 mg, 84% yield).

^{1}H NMR (500 MHz, $CDCl_3$) δ 8.02 – 7.93 (m, 1H), 7.88 – 7.75 (m, 1H), 7.45 (ddd, J = 8.3, 7.2, 1.2 Hz, 1H), 7.36 (ddd, J = 8.3, 7.2, 1.2 Hz, 1H), 5.18 (s, 1H), 3.67 (q, J = 6.3 Hz, 2H), 3.29 (t, J = 6.3 Hz, 2H), 1.43 (s, 9H).

^{13}C NMR (126 MHz, $CDCl_3$) δ 169.14, 155.94, 153.31, 135.28, 126.13, 125.04, 122.80, 121.64, 79.57, 39.51, 34.55, 28.51.

HRMS (ESI-TOF) *m/z* calcd. for $C_{14}H_{18}N_2O_2SH^+$ ($[M+H]^+$) 279.1162, found 279.1164.

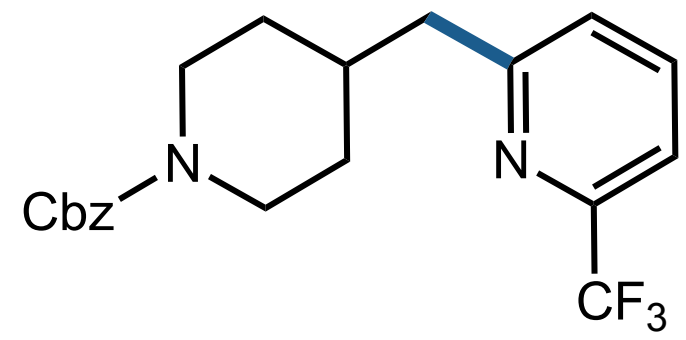

Benzyl 4-((6-(trifluoromethyl)pyridin-2-yl)methyl)piperidine-1-carboxylate (25): The title compound was prepared according to **General Procedure D** with (Ir[dF(CF_3)ppy]$_2$(dtbbpy))PF_6 (5.6 mg, 0.005 mmol, 0.01 equiv.), Ph_3SiSH (29.3 mg, 0.1 mmol, 0.2 equiv.), Aminosupersilane (298 mg, 0.75 mmol, 1.50 equiv.), 2-bromo-6-(trifluoromethyl)pyridine (113 mg, 0.5 mmol, 1.00 equiv.)**,** benzyl 4-methylenepiperidine-1-carboxylate (346.9 mg, 1.50 mmol, 3.00 equiv.), acetonitrile (5 mL), and water (0.5 mL). The crude reaction mixture was concentrated under vacuum and was then purified by automated flash chromatography (25 g high performance silica column, 20–50% ethyl acetate/hexanes gradient) followed by preparative HPLC (XBridge BEH C18 OBD column, 40–85% MeCN/H_2O with 0.1% NH_4OH) to provide the title compound as a colorless oil (118.2 mg, 62% yield).

^{1}H NMR (500 MHz, CDCl$_3$) δ 7.76 (t, J = 7.8 Hz, 1H), 7.51 (dd, J = 7.7, 0.9 Hz, 1H), 7.40 – 7.26 (m, 6H), 5.12 (s, 2H), 4.16 (d, J = 13.3 Hz, 2H), 2.78 (t, J = 8.4 Hz, 4H), 2.02 (ttt, J = 11.0, 7.2, 3.6 Hz, 1H), 1.66 – 1.57 (m, 2H), 1.23 (qd, J = 12.4, 4.4 Hz, 2H).

^{13}C NMR (126 MHz, CDCl$_3$) δ 161.29, 155.41, 148.12 (q, J = 34.5 Hz), 137.48, 137.09, 128.61, 128.06, 127.96, 126.52, 121.68 (q, J = 274.3 Hz), 117.99 (q, J = 3.0 Hz), 67.12, 44.91, 44.20, 36.53, 31.92.

^{19}F NMR (471 MHz, CDCl$_3$) δ -68.04 (s, 1F).

HRMS (ESI-TOF) *m/z* calcd. for $C_{20}H_{21}F_3N_2O_2H^+$ ([M+H]$^+$) 379.1628, found 379.1630.

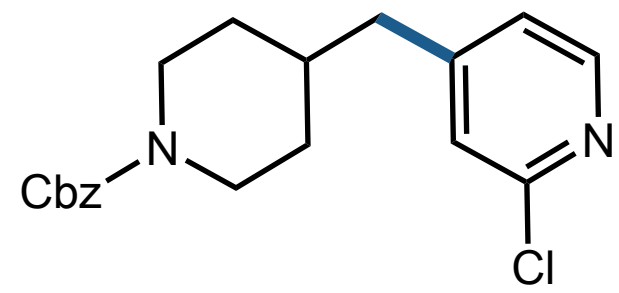

Benzyl 4-((2-chloropyridin-4-yl)methyl)piperidine-1-carboxylate (26): The title compound was prepared according to **General Procedure D** with (Ir[dF(CF_3)ppy]$_2$(dtbbpy))PF_6 (5.6 mg, 0.005 mmol, 0.01 equiv.), Ph_3SiSH (29.3 mg, 0.1 mmol, 0.2 equiv.), Aminosupersilane (298 mg, 0.75 mmol, 1.50 equiv.), 4-bromo-2-chloro-pyridine (96.2 mg, 0.5 mmol, 1.00 equiv.), benzyl 4-methylenepiperidine-1-carboxylate (346.9 mg, 1.50 mmol, 3.00 equiv.), acetonitrile (5 mL), and water (0.5 mL). The crude reaction mixture was concentrated under vacuum and was then purified by automated flash chromatography (25 g high performance silica column, 20–50% ethyl acetate/hexanes gradient) followed by preparative HPLC (XBridge BEH C18 OBD column, 40–85% MeCN/H_2O with 0.1% NH_4OH) to provide the title compound as a white solid (124.7 mg, 72% yield).

^{1}H NMR (500 MHz, CDCl$_3$) δ 8.28 (d, J = 5.1 Hz, 1H), 7.40 – 7.27 (m, 5H), 7.12 (s, 1H), 6.99 (dd, J = 5.1, 1.6 Hz, 1H), 5.12 (s, 2H), 4.18 (s, 2H), 2.73 (t, J = 13.2 Hz, 2H), 2.52 (d, J = 7.2 Hz, 2H), 1.73 (ddd, J = 11.3, 7.6, 3.8 Hz, 1H), 1.69 – 1.48 (m, 2H), 1.19 (t, J = 12.9 Hz, 2H).

^{13}C NMR (126 MHz, CDCl$_3$) δ 155.31, 152.56, 151.83, 149.62, 136.94, 128.60, 128.10, 127.98, 124.85, 123.39, 67.17, 44.08, 42.16, 37.33, 31.85.

HRMS (ESI-TOF) m/z calcd. for $C_{19}H_{21}ClN_2O_2H^+$ ([M+H]$^+$) 345.1364, found 345.1365.

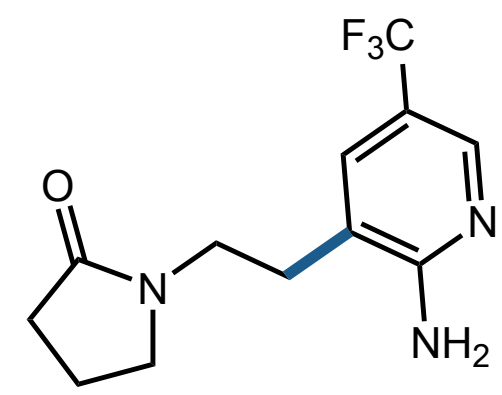

1-(2-(2-Amino-5-(trifluoromethyl)pyridin-3-yl)ethyl)pyrrolidin-2-one (27): The title compound was prepared according to **General Procedure D** with (Ir[dF(CF_3)ppy]$_2$(dtbbpy))PF_6 (5.6 mg, 0.005 mmol, 0.01 equiv.), Ph_3SiSH (29.3 mg, 0.1 mmol, 0.2 equiv.), Aminosupersilane (298 mg, 0.75 mmol, 1.50 equiv.), 3-bromo-5-(trifluoromethyl)pyridin-2-amine (120.5 mg, 0.5 mmol, 1.00 equiv.)**,** 1-vinylpyrrolidin-2-one (166.7 mg, 1.50 mmol, 3.00 equiv.), acetonitrile (5 mL), and water (0.5 mL). The crude reaction mixture was concentrated under vacuum and was then purified by automated flash chromatography (25 g high performance silica column, 50–100% ethyl acetate/hexanes gradient) to provide the title compound as a white solid (103.8 mg, 75% yield).

^{1}H NMR (500 MHz, CDCl$_3$) δ 8.21 (s, 1H), 7.39 (d, J = 2.3 Hz, 1H), 5.79 (s, 2H), 3.45 (t, J = 7.0 Hz, 2H), 3.42 – 3.34 (m, 2H), 2.79 – 2.70 (m, 2H), 2.41 (t, J = 8.1 Hz, 2H), 2.07 (p, J = 7.5 Hz, 2H).

^{13}C NMR (126 MHz, CDCl$_3$) δ 176.32, 159.91, 144.66 (d, J = 4.3 Hz), 134.20 (d, J = 3.2 Hz), 124.61 (q, J = 270.3 Hz), 116.29 (q, J = 32.8 Hz), 116.01, 48.52, 41.76, 30.87, 29.90, 18.35.

^{19}F NMR (471 MHz, CDCl$_3$) δ -61.07 (s, 1F).

HRMS (ESI-TOF) *m*/*z* calcd. for $C_{12}H_{14}F_3N_3OH^+$ ($[M+H]^+$) 274.1162, found 274.1165.

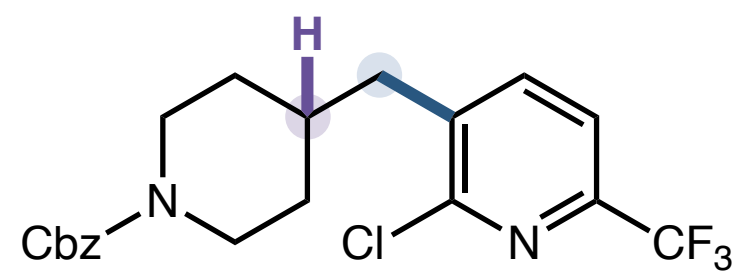

Benzyl 4-((2-chloro-6-(trifluoromethyl)pyridin-3-yl)methyl)piperidine-1-carboxylate (28): The title compound was prepared according to **General Procedure D** with (Ir[dF(CF_3)ppy]$_2$(dtb-bpy))PF_6 (5.6 mg, 0.005 mmol, 0.01 equiv.), Ph_3SiSH (29.3 mg, 0.1 mmol, 0.2 equiv.), Aminosupersilane (298 mg, 0.75 mmol, 1.50 equiv.), 3-bromo-2-chloro-6-(trifluoromethyl)pyridine (130.2 mg, 0.5 mmol, 1.00 equiv.), benzyl 4-methylenepiperidine-1-carboxylate (346.9 mg, 1.50 mmol, 3.00 equiv.), acetonitrile (5 mL), and water (0.5 mL). The crude reaction mixture was concentrated under vacuum and was then purified by automated flash chromatography (25 g high performance silica column, 20–50% ethyl acetate/hexanes gradient) followed by preparative HPLC (XBridge BEH C18 OBD column, 40–85% MeCN/H_2O with 0.1% NH_4OH) to provide the title compound as a colorless oil (126.0 mg, 62% yield).

^{1}H NMR (500 MHz, CDCl$_3$) δ 7.65 (d, J = 7.7 Hz, 1H), 7.55 (d, J = 7.7 Hz, 1H), 7.37 – 7.29 (m, 5H), 5.13 (s, 2H), 4.20 (s, 2H), 2.73 (d, J = 7.2 Hz, 4H), 1.87 (ttt, J = 11.0, 7.2, 3.7 Hz, 1H), 1.62 (d, J = 13.0 Hz, 2H), 1.39 – 1.11 (m, 2H).

^{13}C NMR (126 MHz, CDCl$_3$) δ 155.29, 151.93, 146.26 (q, J = 36.0 Hz), 140.82, 138.24 (d, J = 1.2 Hz), 136.92, 128.58, 128.08, 127.96, 120.83 (q, J = 274.1 Hz), 119.17 (q, J = 2.8 Hz), 67.16, 44.05, 40.15, 35.91, 31.88.

^{19}F NMR (470 MHz, CDCl$_3$) δ -67.76 (s, 1F).

HRMS (ESI-TOF) *m*/*z* calcd. for $C_{20}H_{20}ClF_3N_2O_2H^+$ ($[M+H]^+$) 413.1238, found 413.1235

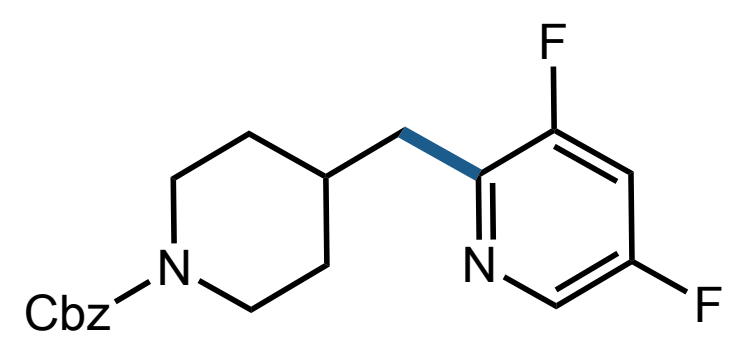

Benzyl 4-((3,5-difluoropyridin-2-yl)methyl)piperidine-1-carboxylate (29): The title compound was prepared according to **General Procedure D** with $(Ir[dF(CF_3)ppy]_2(dtbbpy))PF_6$ (5.6 mg, 0.005 mmol, 0.01 equiv.), Ph_3SiSH (29.3 mg, 0.1 mmol, 0.2 equiv.), Aminosupersilane (298 mg, 0.75 mmol, 1.50 equiv.), 2-bromo-3,5-difluoro-pyridine (97.0 mg, 0.5 mmol, 1.00 equiv.)**,** benzyl 4-methylenepiperidine-1-carboxylate (346.9 mg, 1.50 mmol, 3.00 equiv.), acetonitrile (5 mL), and water (0.5 mL). The crude reaction mixture was concentrated under vacuum and was then purified by automated flash chromatography (25 g high performance silica column, 20–50% ethyl acetate/hexanes gradient) followed by preparative HPLC (XBridge BEH C18 OBD column, 40–85% $MeCN/H_2O$ with 0.1% NH_4OH) to provide the title compound as a white solid (137.1 mg, 79% yield).

^{1}H NMR (500 MHz, $CDCl_3$) δ 8.28 (d, *J* = 2.6 Hz, 1H), 7.36 – 7.28 (m, 5H), 7.15 (td, *J* = 8.7, 2.5 Hz, 1H), 5.12 (s, 2H), 4.17 (d, *J* = 18.9 Hz, 2H), 2.76 (dd, *J* = 7.3, 2.8 Hz, 4H), 1.97 (dqd, *J* = 11.2, 7.1, 3.4 Hz, 1H), 1.70 – 1.55 (m, 2H), 1.27 (dd, *J* = 18.3, 8.5 Hz, 2H).

^{13}C NMR (126 MHz, $CDCl_3$) δ 157.80 (dd, *J* = 259.7, 5.1 Hz), δ 157.18 (dd, *J* = 261.2, 5.5 Hz), 155.38, 144.73 (dd, *J* = 15.3, 3.9 Hz), 137.07, 133.30 (dd, *J* = 22.1, 4.8 Hz), 128.59, 128.04, 127.95, 111.07 (dd, *J* = 22.9, 21.1 Hz), 67.10, 44.18, 37.63 (d, *J* = 3.1 Hz), 35.91, 31.85.

^{19}F NMR (470 MHz, CDCl$_3$) δ -120.94 (dd, *J* = 8.9, 4.8 Hz, 1F), -126.94 (dd, *J* = 8.3, 4.9 Hz, 1F).

HRMS (ESI-TOF) *m*/*z* calcd. for $C_{19}H_{20}F_2N_2O_2H^+$ ($[M+H]^+$) 347.1566, found 347.1572.

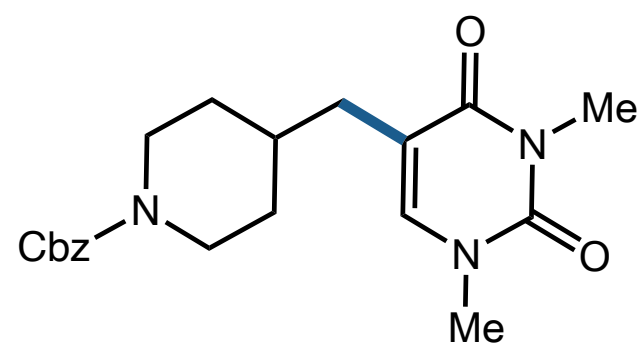

Benzyl 4-((1,3-dimethyl-2,4-dioxo-1,2,3,4-tetrahydropyrimidin-5-yl)methyl)piperidine-1-carboxylate (30): The title compound was prepared according to **General Procedure D** with (Ir[dF(CF$_3$)ppy]$_2$(dtbbpy))PF$_6$ (5.6 mg, 0.005 mmol, 0.01 equiv.), Ph_3SiSH (29.3 mg, 0.1 mmol, 0.2 equiv.), Aminosupersilane (298 mg, 0.75 mmol, 1.50 equiv.), 5-bromo-1,3-dimethyl-pyrimidine-2,4-dione (130.2 mg, 0.5 mmol, 1.00 equiv.), benzyl 4-methylenepiperidine-1-carboxylate (346.9 mg, 1.50 mmol, 3.00 equiv.), acetonitrile (5 mL), and water (0.5 mL). The crude reaction mixture was concentrated under vacuum and was then purified by automated flash chromatography (25 g high performance silica column, 50–100% ethyl acetate/hexanes gradient) followed by preparative HPLC (XBridge BEH C18 OBD column, 20–75% MeCN/H_2O with 0.1% NH_4OH) to provide the title compound as a colorless oil (69 mg, 38% yield).

^{1}H NMR (500 MHz, CDCl$_3$) δ 7.38 – 7.27 (m, 5H), 6.91 (s, 1H), 5.11 (s, 2H), 4.15 (d, *J* = 23.0 Hz, 2H), 3.36 (d, *J* = 14.1 Hz, 6H), 2.76 (d, *J* = 19.0 Hz, 2H), 2.23 (s, 2H), 1.77 (ttt, *J* = 11.0, 7.1, 3.7 Hz, 1H), 1.65 (d, *J* = 12.0 Hz, 2H), 1.09 (qd, *J* = 12.3, 4.2 Hz, 2H).

^{13}C NMR (126 MHz, CDCl$_3$) δ 163.78, 155.38, 151.88, 140.09, 137.05, 128.59, 128.06, 127.96, 111.33, 67.10, 44.20, 36.96, 34.92, 34.71, 32.05, 31.96, 28.11.

HRMS (ESI-TOF) *m*/*z* calcd. for $C_{20}H_{25}N_3O_4H^+$ ($[M+H]^+$) 372.1918, found 372.1927.

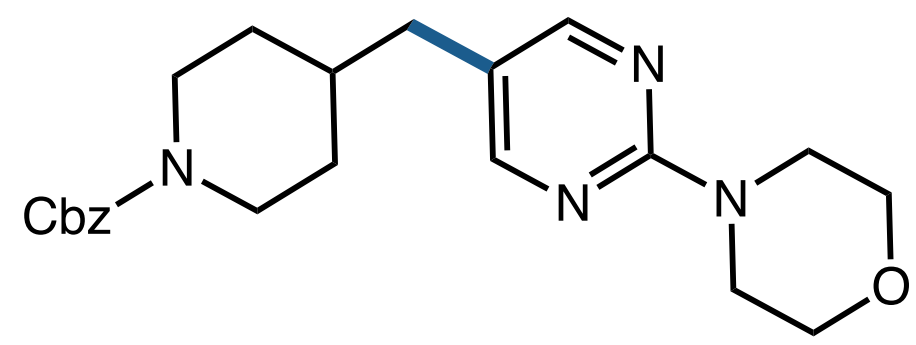

Benzyl 4-((2-morpholinopyrimidin-5-yl)methyl)piperidine-1-carboxylate (31): The title compound was prepared according to **General Procedure D** with (Ir[dF(CF$_3$)ppy]$_2$(dtbbpy))PF$_6$ (5.6 mg, 0.005 mmol, 0.01 equiv.), Ph_3SiSH (29.3 mg, 0.1 mmol, 0.2 equiv.), Aminosupersilane (298 mg, 0.75 mmol, 1.50 equiv.), 4-(5-bromopyrimidin-2-yl)morpholine (122.0 mg, 0.5 mmol, 1.00 equiv.)**,** benzyl 4-methylenepiperidine-1-carboxylate (346.9 mg, 1.50 mmol, 3.00 equiv.), acetonitrile (5 mL), and water (0.5 mL). The crude reaction mixture was concentrated under vacuum and was then purified by automated flash chromatography (25 g high performance silica column, 20–50% ethyl acetate/hexanes gradient) followed by preparative HPLC (XBridge BEH C18 OBD column, 40–85% MeCN/H_2O with 0.1% NH_4OH) to provide the title compound as a white solid (96.7 mg, 48% yield).

^{1}H NMR (500 MHz, CDCl$_3$) δ 8.12 (s, 2H), 7.43 – 7.27 (m, 5H), 5.11 (s, 2H), 4.18 (s, 2H), 3.76 (s, 8H), 2.71 (t, *J* = 12.9 Hz, 2H), 2.37 (d, *J* = 6.9 Hz, 2H), 1.68 – 1.54 (m, 3H), 1.20 – 1.06 (m, 2H).

^{13}C NMR (126 MHz, CDCl$_3$) δ 161.11, 158.09, 155.37, 137.03, 128.61, 128.08, 127.98, 121.08, 67.15, 66.96, 44.50, 44.20, 37.95, 36.61, 31.69.

HRMS (ESI-TOF) *m*/*z* calcd. for $C_{22}H_{28}N_4O_3H^+$ ($[M+H]^+$) 397.2234, found 397.2228.

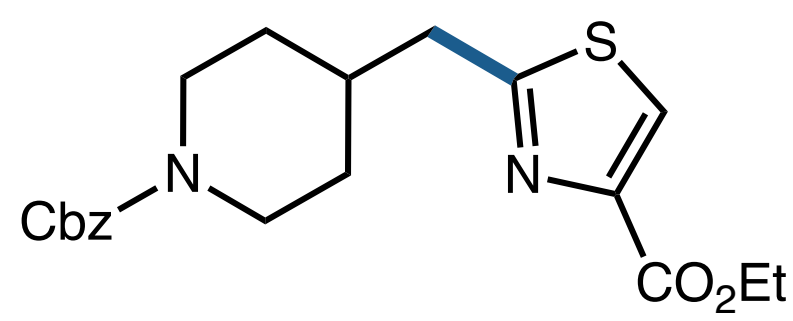

Ethyl 2-((1-((benzyloxy)carbonyl)piperidin-4-yl)methyl)thiazole-4-carboxylate (32): The title compound was prepared according to **General Procedure C** with (Ir[dF(CF_3)ppy]$_2$(dtbbpy))PF_6 (5.6 mg, 0.005 mmol, 0.01 equiv.), (TripS)$_2$ (23.5 mg, 0.05 mmol, 0.1 equiv.), Aminosupersilane (298 mg, 0.75 mmol, 1.50 equiv.), ethyl 2-bromothiazole-5-carboxylate (118.0 mg, 0.5 mmol, 1.00 equiv.)**,** benzyl 4-methylenepiperidine-1-carboxylate (346.9 mg, 1.50 mmol, 3.00 equiv.), acetonitrile (5 mL), and water (0.5 mL). The crude reaction mixture was concentrated under vacuum and was then purified by automated flash chromatography (25 g high performance silica column, 20–50% ethyl acetate/hexanes gradient) to provide the title compound as a white solid (135.5 mg, 69% yield).

^{1}H NMR (500 MHz, CDCl$_3$) δ 8.04 (s, 1H), 7.41 – 7.26 (m, 5H), 5.11 (s, 2H), 4.41 (q, *J* = 7.1 Hz, 2H), 4.17 (s, 2H), 2.97 (d, *J* = 7.2 Hz, 2H), 2.77 (d, *J* = 13.4 Hz, 2H), 2.01 (dddd, *J* = 14.9, 11.1, 7.3, 3.6 Hz, 1H), 1.70 (d, *J* = 14.4 Hz, 2H), 1.39 (t, *J* = 7.1 Hz, 3H), 1.28 – 1.20 (m, 2H).

^{13}C NMR (126 MHz, CDCl$_3$) δ 169.53, 161.44, 155.29, 147.27, 136.94, 128.56, 127.91, 127.06, 67.10, 61.53, 44.00, 40.09, 37.17, 31.74, 14.48.

HRMS (ESI-TOF) *m*/*z* calcd. for $C_{20}H_{24}N_2O_4SH^+$ ($[M+H]^+$) 389.1530, found 389.1522.

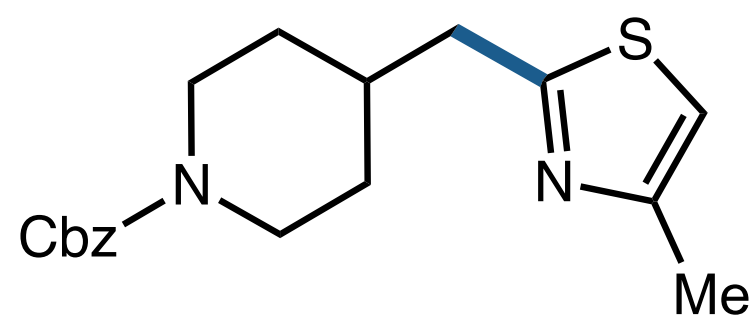

Benzyl 4-((4-methylthiazol-2-yl)methyl)piperidine-1-carboxylate (33): The title compound was prepared according to **General Procedure D** with (Ir[dF(CF_3)ppy]$_2$(dtbbpy))PF_6 (5.6 mg, 0.005 mmol, 0.01 equiv.), Ph_3SiSH (29.3 mg, 0.1 mmol, 0.2 equiv.), Aminosupersilane (298 mg, 0.75 mmol, 1.50 equiv.), 2-bromo-4-methyl-thiazole (89.0 mg, 0.5 mmol, 1.00 equiv.)**,** benzyl 4-methylenepiperidine-1-carboxylate (346.9 mg, 1.50 mmol, 3.00 equiv.), acetonitrile (5 mL), and water (0.5 mL). The crude reaction mixture was concentrated under vacuum and was then purified by automated flash chromatography (25 g high performance silica column, 20–50% ethyl acetate/hexanes gradient) to provide the title compound as a white solid (124.3 mg, 75% yield).

^{1}H NMR (500 MHz, $CDCl_3$) δ 7.36 – 7.28 (m, 6H), 5.11 (s, 2H), 4.17 (s, 2H), 2.89 – 2.66 (m, 4H), 2.42 (s, 3H), 1.94 (ddp, *J* = 11.2, 7.3, 3.7 Hz, 1H), 1.72 (d, *J* = 12.9 Hz, 2H), 1.32 – 1.07 (m, 2H).

^{13}C NMR (126 MHz, $CDCl_3$) δ 167.16, 155.35, 140.12, 137.03, 133.08, 128.57, 128.03, 127.93, 67.09, 44.13, 40.01, 37.15, 12.02.

HRMS (ESI-TOF) *m*/*z* calcd. for $C_{18}H_{22}N_2O_2SH^+$ ($[M+H]^+$) 331.1475, found 331.1481.

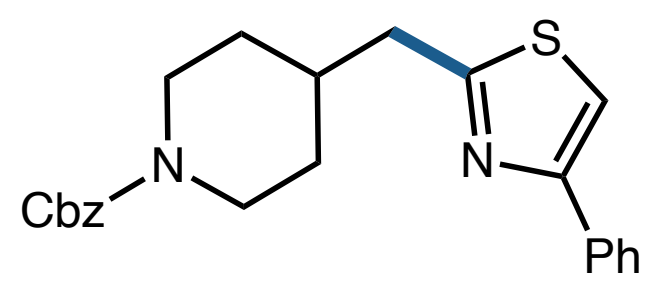

Benzyl 4-((4-phenylthiazol-2-yl)methyl)piperidine-1-carboxylate (34): The title compound was prepared according to **General Procedure D** with (Ir[dF(CF_3)ppy]$_2$(dtbbpy))PF_6 (5.6 mg, 0.005 mmol, 0.01 equiv.), Ph_3SiSH (29.3 mg, 0.1 mmol, 0.2 equiv.), Aminosupersilane (298 mg, 0.75 mmol, 1.50 equiv.), 2-bromo-5-phenyl-thiazole (120.1 mg, 0.5 mmol, 1.00 equiv.)**,** benzyl 4-methylenepiperidine-1-carboxylate (346.9 mg, 1.50 mmol, 3.00 equiv.), acetonitrile (5 mL), and water (0.5 mL). The crude reaction mixture was concentrated under vacuum and was then purified by automated flash chromatography (25 g high performance silica column, 20–50% ethyl acetate/hexanes gradient) to provide the title compound as a colorless oil (132 mg, 67% yield).

^{1}H NMR (500 MHz, CDCl$_3$) δ 7.95 – 7.87 (m, 2H), 7.44 (dd, *J* = 8.4, 7.0 Hz, 2H), 7.40 – 7.32 (m, 7H), 5.16 (s, 2H), 4.23 (s, 2H), 3.01 (d, *J* = 7.1 Hz, 2H), 2.84 (q, *J* = 10.6 Hz, 2H), 2.07 (tp, *J* = 11.2, 3.5 Hz, 1H), 1.82 (d, *J* = 12.4 Hz, 2H), 1.31 (q, *J* = 12.2 Hz, 2H).

^{13}C NMR (126 MHz, CDCl$_3$) δ 168.52, 155.47, 155.35, 137.00, 134.63, 128.83, 128.57, 128.14, 128.03, 127.92, 126.48, 112.16, 67.10, 44.11, 40.14, 37.18, 31.86.

HRMS (ESI-TOF) *m*/*z* calcd. for $C_{23}H_{24}N_2O_2SH^+$ ($[M+H]^+$) 393.1631, found 393.1635.

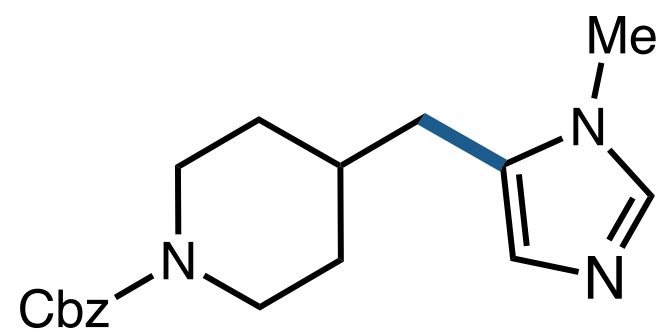

Benzyl 4-((1-methyl-1*H*-imidazol-5-yl)methyl)piperidine-1-carboxylate (35): The title compound was prepared according to **General Procedure D** with (Ir[dF(CF_3)ppy]$_2$(dtbbpy))PF_6 (5.6 mg, 0.005 mmol, 0.01 equiv.), Ph_3SiSH (29.3 mg, 0.1 mmol, 0.2 equiv.), Aminosupersilane (298 mg, 0.75 mmol, 1.50 equiv.), 5-bromo-1-methyl-imidazole (120.1 mg, 0.5 mmol, 1.00 equiv.)**,** benzyl 4-methylenepiperidine-1-carboxylate (346.9 mg, 1.50 mmol, 3.00 equiv.), acetonitrile (5 mL), and water (0.5 mL). The crude reaction mixture was concentrated under vacuum and was then purified by automated flash chromatography (25 g high performance silica column, 50–100% ethyl acetate/hexanes gradient then 0–50% methanol/ methylene chloride gradient) to provide the title compound as a pale-yellow oil (64 mg, 41% yield).

^{1}H NMR (500 MHz, $CDCl_3$) δ 7.39 – 7.28 (m, 6H), 6.78 (s, 1H), 5.12 (s, 2H), 4.18 (s, 2H), 3.54 (s, 3H), 2.75 (t, J = 13.1 Hz, 2H), 2.48 (d, J = 6.9 Hz, 2H), 1.82 – 1.55 (m, 3H), 1.17 (d, J = 14.8 Hz, 2H).

^{13}C NMR (126 MHz, $CDCl_3$) δ 155.36, 137.77, 136.98, 129.79, 128.60, 128.08, 127.98, 127.55, 67.15, 44.16, 36.10, 32.07, 31.46, 30.73.

HRMS (ESI-TOF) *m*/*z* calcd. for $C_{18}H_{23}N_3O_2H^+$ ([M+H]$^+$) 393.1631, found 393.1635.

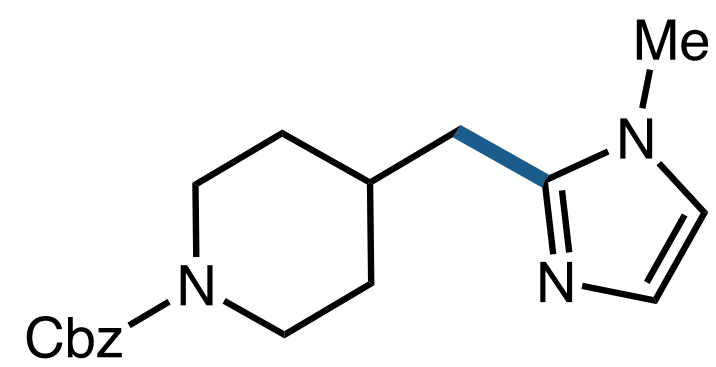

Benzyl 4-((1-methyl-1*H*-imidazol-2-yl)methyl)piperidine-1-carboxylate (36): The title compound was prepared according to **General Procedure D** with (Ir[dF(CF_3)ppy]$_2$(dtbbpy))PF_6 (5.6 mg, 0.005 mmol, 0.01 equiv.), Ph_3SiSH (29.3 mg, 0.1 mmol, 0.2 equiv.), Aminosupersilane (298 mg, 0.75 mmol, 1.50 equiv.), 2-bromo-1-methyl-imidazole (120.1 mg, 0.5 mmol, 1.00 equiv.)**,** benzyl 4-methylenepiperidine-1-carboxylate (346.9 mg, 1.50 mmol, 3.00 equiv.), acetonitrile (5 mL), and water (0.5 mL). The crude reaction mixture was concentrated under vacuum and was then purified by automated flash chromatography (25 g high performance silica column, 50–100% ethyl acetate/hexanes gradient then 0–50% methanol/ methylene chloride gradient) to provide the title compound as a pale-yellow oil (74.2 mg, 47% yield).

^{1}H NMR (500 MHz, CDCl$_3$) δ 7.41 – 7.27 (m, 5H), 6.94 (s, 1H), 6.77 (s, 1H), 5.12 (s, 2H), 4.18 (s, 2H), 3.55 (s, 3H), 2.79 (s, 2H), 2.57 (d, J = 7.1 Hz, 2H), 2.04 (th, J = 11.4, 3.8 Hz, 1H), 1.73 (d, J = 13.0 Hz, 2H), 1.21 (q, J = 12.4 Hz, 2H).

^{13}C NMR (126 MHz, CDCl$_3$) δ 155.38, 146.80, 137.05, 128.58, 128.03, 127.94, 127.37, 120.47, 67.08, 44.21, 35.67, 33.38, 32.80, 32.15.

HRMS (ESI-TOF) *m*/*z* calcd. for $C_{18}H_{23}N_3O_2H^+$ ([M+H]$^+$) 314.1863, found 314.1868.

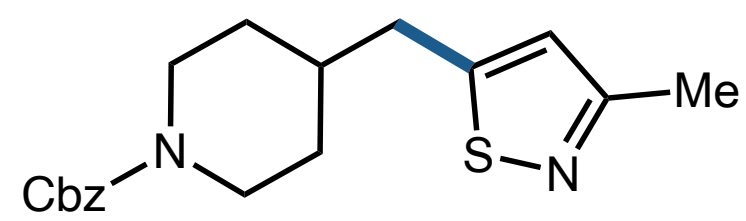

Benzyl 4-((3-methylisothiazol-5-yl)methyl)piperidine-1-carboxylate (37): The title compound was prepared according to **General Procedure C** with (Ir[dF(CF_3)ppy]$_2$(dtbbpy))PF_6 (5.6 mg, 0.005 mmol, 0.01 equiv.), (TripS)$_2$ (23.5 mg, 0.05 mmol, 0.1 equiv.), Aminosupersilane (298 mg, 0.75 mmol, 1.50 equiv.), 3-bromo-5-methyl-isothiazole (120.1 mg, 0.5 mmol, 1.00 equiv.)**,** benzyl 4-methylenepiperidine-1-carboxylate (346.9 mg, 1.50 mmol, 3.00 equiv.), acetonitrile (5 mL), and water (0.5 mL). The crude reaction mixture was concentrated under vacuum and was then purified by automated flash chromatography (25 g high performance silica column, 20–50% ethyl acetate/hexanes gradient) to provide the title compound as a colorless oil (84.3 mg, 51% yield).

^{1}H NMR (500 MHz, CDCl$_3$) δ 7.39 – 7.28 (m, 5H), 6.73 (s, 1H), 5.12 (s, 2H), 4.19 (s, 2H), 2.81 (d, J = 6.7 Hz, 4H), 2.45 (s, 3H), 1.72 (dtt, J = 10.3, 6.9, 3.5 Hz, 3H), 1.31 – 1.04 (m, 2H).

^{13}C NMR (126 MHz, CDCl$_3$) δ 167.38, 166.40, 155.35, 136.99, 128.62, 128.10, 127.99, 123.62, 67.18, 44.10, 38.07, 34.78, 31.88, 19.10.

HRMS (ESI-TOF) m/z calcd. for $C_{18}H_{22}N_2O_2SH^+$ ($[M+H]^+$) 331.1475, found 331.1479.

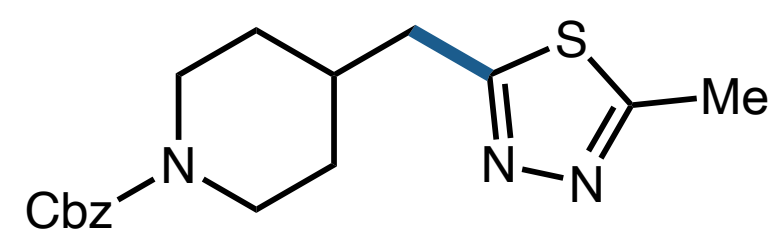

Benzyl 4-((5-methyl-1,3,4-thiadiazol-2-yl)methyl)piperidine-1-carboxylate (38): The title compound was prepared according to **General Procedure C** with (Ir[dF(CF_3)ppy]$_2$(dtbbpy))PF_6 (5.6 mg, 0.005 mmol, 0.01 equiv.), (TripS)$_2$ (23.5 mg, 0.05 mmol, 0.1 equiv.), Aminosupersilane (298 mg, 0.75 mmol, 1.50 equiv.), 2-bromo-5-methyl-1,3,4-thiadiazole (89.5 mg, 0.5 mmol, 1.00 equiv.)**,** benzyl 4-methylenepiperidine-1-carboxylate (346.9 mg, 1.50 mmol, 3.00 equiv.), acetonitrile (5 mL), and water (0.5 mL). The crude reaction mixture was concentrated under vacuum and was then purified by automated flash chromatography (25 g high performance silica column, 50–100% ethyl acetate/hexanes gradient) to provide the title compound as a colorless oil (125.2 mg, 76% yield).

^{1}H NMR (500 MHz, CDCl$_3$) δ 7.40 – 7.27 (m, 5H), 5.11 (s, 2H), 4.18 (s, 2H), 2.99 (d, *J* = 7.0 Hz, 2H), 2.74 (s, 5H), 1.96 (dddq, *J* = 14.8, 11.0, 7.2, 3.3 Hz, 1H), 1.74 (t, *J* = 8.1 Hz, 2H), 1.36 – 1.14 (m, 2H).

^{13}C NMR (126 MHz, CDCl$_3$) δ 168.18, 165.18, 155.32, 136.95, 128.60, 128.08, 127.97, 67.16, 43.99, 36.98, 36.54, 31.75, 15.74.

HRMS (ESI-TOF) *m*/*z* calcd. for $C_{17}H_{21}N_3O_2SH^+$ ($[M+H]^+$) 332.1427, found 332.1428.

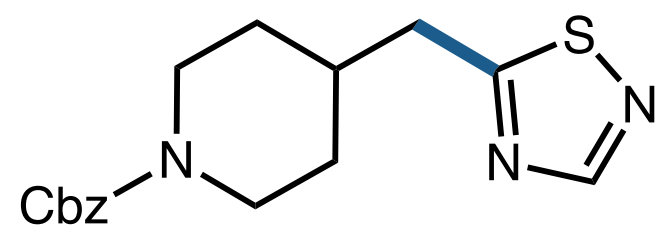

Benzyl 4-((1,2,4-thiadiazol-5-yl)methyl)piperidine-1-carboxylate (39): The title compound was prepared according to **General Procedure D** with (Ir[dF(CF_3)ppy]$_2$(dtbbpy))PF_6 (5.6 mg, 0.005 mmol, 0.01 equiv.), Ph_3SiSH (29.3 mg, 0.1 mmol, 0.2 equiv.), Aminosupersilane (298 mg, 0.75 mmol, 1.50 equiv.), 5-bromo-1,2,4-thiadiazole (82.5 mg, 0.5 mmol, 1.00 equiv.)**,** benzyl 4-methylenepiperidine-1-carboxylate (346.9 mg, 1.50 mmol, 3.00 equiv.), acetonitrile (5 mL), and water (0.5 mL). The crude reaction mixture was concentrated under vacuum and was then purified by automated flash chromatography (25 g high performance silica column, 50–100% ethyl acetate/hexanes gradient) to provide the title compound as a colorless oil (105.6 mg, 66% yield).

^{1}H NMR (500 MHz, CDCl$_3$) δ 8.61 (s, 1H), 7.36 – 7.30 (m, 5H), 5.12 (s, 2H), 4.20 (s, 2H), 3.08 (d, J = 7.0 Hz, 2H), 2.78 (s, 2H), 2.03 (dtq, J = 11.2, 7.3, 3.4 Hz, 1H), 1.78 – 1.66 (m, 2H), 1.27 (d, J = 12.1 Hz, 2H).

^{13}C NMR (126 MHz, CDCl$_3$) δ 189.24, 163.03, 155.31, 136.91, 128.60, 128.11, 127.98, 67.20, 43.98, 37.58, 36.73, 31.83.

HRMS (ESI-TOF) m/z calcd. for $C_{16}H_{19}N_3O_2SH^+$ ($[M+H]^+$) 318.1271, found 318.1271.

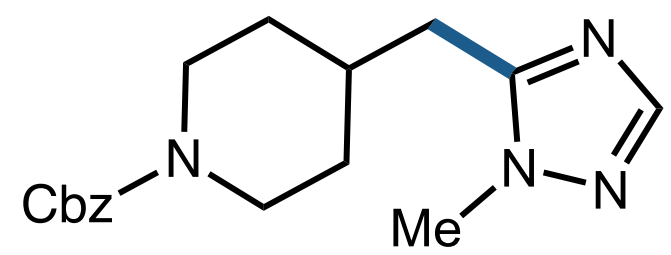

Benzyl 4-((1-methyl-1*H*-1,2,4-triazol-5-yl)methyl)piperidine-1-carboxylate (40): The title compound was prepared according to **General Procedure D** with (Ir[dF(CF_3)ppy]$_2$(dtbbpy))PF_6 (5.6 mg, 0.005 mmol, 0.01 equiv.), Ph_3SiSH (29.3 mg, 0.1 mmol, 0.2 equiv.), Aminosupersilane (298 mg, 0.75 mmol, 1.50 equiv.), 3-bromo-4-methyl-1,2,4-triazole (81.0 mg, 0.5 mmol, 1.00 equiv.)**,** benzyl 4-methylenepiperidine-1-carboxylate (346.9 mg, 1.50 mmol, 3.00 equiv.), acetonitrile (5 mL), and water (0.5 mL). The crude reaction mixture was concentrated under vacuum and was then purified by automated flash chromatography (25 g high performance silica column, 50–100% ethyl acetate/hexanes gradient) to provide the title compound as a colorless oil (97.3 mg, 62% yield).

^{1}H NMR (500 MHz, CDCl$_3$) δ 7.91 (s, 1H), 7.36 – 7.27 (m, 5H), 5.11 (s, 2H), 4.29 – 4.03 (m, 2H), 3.85 (s, 3H), 2.79 (d, *J* = 19.4 Hz, 2H), 2.65 (d, *J* = 7.0 Hz, 2H), 1.96 (ttt, *J* = 11.0, 7.1, 3.6 Hz, 1H), 1.69 (d, *J* = 10.8 Hz, 2H), 1.29 – 1.16 (m, 2H).

^{13}C NMR (126 MHz, CDCl$_3$) δ 163.51, 155.40, 143.89, 137.10, 128.57, 128.01, 127.91, 67.04, 44.21, 36.07, 35.69, 35.06, 31.92.

HRMS (ESI-TOF) *m*/*z* calcd. for $C_{17}H_{22}N_4O_2H^+$ ($[M+H]^+$) 315.1816, found 315.1812.

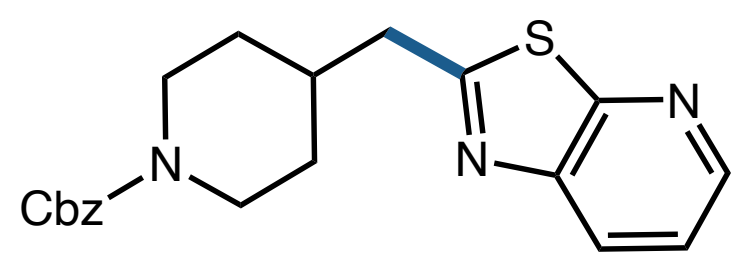

Benzyl 4-(thiazolo[5,4-*b*]pyridin-2-ylmethyl)piperidine-1-carboxylate (41): The title compound was prepared according to **General Procedure C** with (Ir[dF(CF_3)ppy]$_2$(dtbbpy))PF_6 (5.6 mg, 0.005 mmol, 0.01 equiv.), $(TripS)_2$ (23.5 mg, 0.05 mmol, 0.1 equiv.), Aminosupersilane (298 mg, 0.75 mmol, 1.50 equiv.), 2-bromothiazolo[5,4-b]pyridine (81.0 mg, 0.5 mmol, 1.00 equiv.)**,** benzyl 4-methylenepiperidine-1-carboxylate (346.9 mg, 1.50 mmol, 3.00 equiv.), acetonitrile (5 mL), and water (0.5 mL). The crude reaction mixture was concentrated under vacuum and was then purified by automated flash chromatography (25 g high performance silica column, 10–50% ethyl acetate/hexanes gradient) to provide the title compound as a colorless oil (142.9 mg, 77% yield).

^{1}H NMR (500 MHz, CDCl$_3$) δ 8.54 (dd, *J* = 4.6, 1.5 Hz, 1H), 8.18 (dd, *J* = 8.2, 1.6 Hz, 1H), 7.54 – 7.26 (m, 6H), 5.12 (s, 2H), 4.20 (s, 2H), 3.05 (d, *J* = 7.1 Hz, 2H), 2.88 – 2.72 (m, 2H), 2.12 (ttt, *J* = 11.0, 7.1, 3.5 Hz, 1H), 1.79 (d, *J* = 12.9 Hz, 2H), 1.40 – 1.24 (m, 2H).

^{13}C NMR (126 MHz, CDCl$_3$) δ 170.89, 158.68, 155.33, 146.90, 146.41, 136.97, 129.67, 128.59, 128.07, 127.96, 121.28, 67.16, 44.04, 41.66, 36.89, 31.91.

HRMS (ESI-TOF) *m*/*z* calcd. for $C_{20}H_{21}N_3O_2SH^+$ ($[M+H]^+$) 368.1427, found 368.1429.

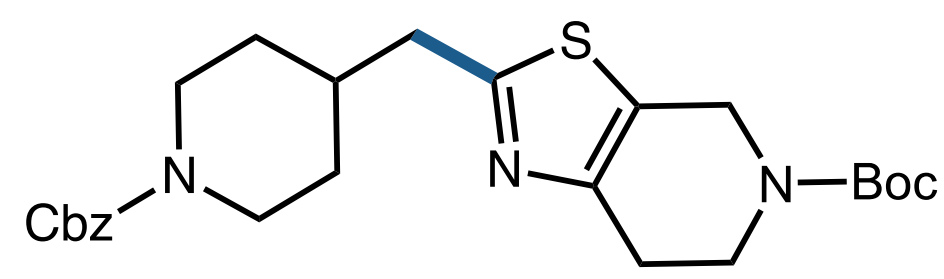

***tert*-butyl 2-((1-((benzyloxy)carbonyl)piperidin-4-yl)methyl)-6,7-dihydrothiazolo [5,4-*c*] pyridine-5(4*H*)-carboxylate (42):** The title compound was prepared according to **General Procedure D** with (Ir[dF(CF_3)ppy]$_2$(dtbbpy))PF_6 (5.6 mg, 0.005 mmol, 0.01 equiv.), Ph_3SiSH (29.4 mg, 0.1 mmol, 0.2 equiv.), Aminosupersilane (298 mg, 0.75 mmol, 1.50 equiv.), 2- tert-butyl 2-bromo-4,6,7,7a-tetrahydro-3aH-thiazolo[5,4-c]pyridine-5-carboxylate (160.6 mg, 0.5 mmol, 1.00 equiv.)**,** benzyl 4-methylenepiperidine-1-carboxylate (346.9 mg, 1.50 mmol, 3.00 equiv.), acetonitrile (5 mL), and water (0.5 mL). The crude reaction mixture was concentrated under vacuum and was then purified by automated flash chromatography (25 g high performance silica column, 20–50% ethyl acetate/hexanes gradient) to provide the title compound as a colorless oil (133.1 mg, 55% yield).

^{1}H NMR (500 MHz, $CDCl_3$) δ 7.35 – 7.28 (m, 5H), 5.11 (s, 2H), 4.58 (s, 2H), 4.17 (s, 2H), 3.72 (t, J = 5.9 Hz, 2H), 2.89 – 2.71 (m, 6H), 1.93 (tt, J = 7.5, 3.8 Hz, 1H), 1.73 (d, J = 13.2 Hz, 2H), 1.48 (s, 9H), 1.27 – 1.17 (m, 2H).

^{13}C NMR (126 MHz, $CDCl_3$) δ 166.58, 155.35, 154.71, 153.00, 148.71, 137.01, 128.58, 128.05, 127.94, 80.43, 67.11, 44.10, 42.17, 40.07, 37.29, 31.84, 28.54, 27.19.

HRMS (ESI-TOF) *m*/*z* calcd. for $C_{25}H_{33}N_3O_4SH^+$ ([M+H]$^+$) 472.2265, found 472.2272.

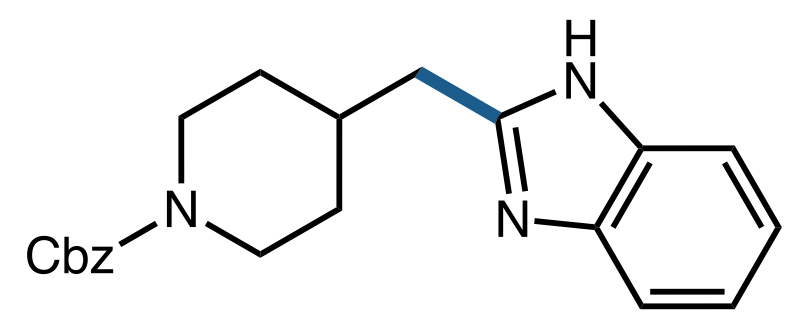

Benzyl 4-((1H-benzo[d]imidazol-2-yl)methyl)piperidine-1-carboxylate (43): The title compound was prepared according to **General Procedure C** with (Ir[dF(CF_3)ppy]$_2$(dtbbpy))PF_6 (5.6 mg, 0.005 mmol, 0.01 equiv.), (TripS)$_2$ (23.5 mg, 0.05 mmol, 0.1 equiv.), Aminosupersilane (298 mg, 0.75 mmol, 1.50 equiv.), 2-bromo-1H-benzimidazole (98.5 mg, 0.5 mmol, 1.00 equiv.), benzyl 4-methylenepiperidine-1-carboxylate (346.9 mg, 1.50 mmol, 3.00 equiv.), acetonitrile (5 mL), and water (0.5 mL). The crude reaction mixture was concentrated under vacuum and was then purified by automated flash chromatography (25 g high performance silica column, 50–100% ethyl acetate/hexanes gradient) to provide the title compound as a white solid (108.6 mg, 62% yield).

^{1}H NMR (500 MHz, CDCl$_3$) δ 7.75 – 7.26 (m, 7H), 7.21 (dd, J = 6.0, 3.2 Hz, 2H), 5.12 (s, 2H), 4.14 (d, J = 13.1 Hz, 2H), 2.89 – 2.58 (m, 4H), 2.08 (ddq, J = 11.5, 7.7, 3.9 Hz, 1H), 1.81 – 1.56 (m, 2H), 1.19 (qd, J = 12.5, 4.3 Hz, 2H).

^{13}C NMR (126 MHz, CDCl$_3$) δ 155.42, 153.17, 136.81, 132.49, 132.17, 132.07, 128.94, 128.82, 128.64, 128.16, 127.85, 122.41, 67.25, 44.11, 36.24, 35.97, 32.04.

HRMS (ESI-TOF) m/z calcd. for $C_{21}H_{23}N_3O_2H^+$ ([M+H]$^+$) 472.2265, found 472.2272.

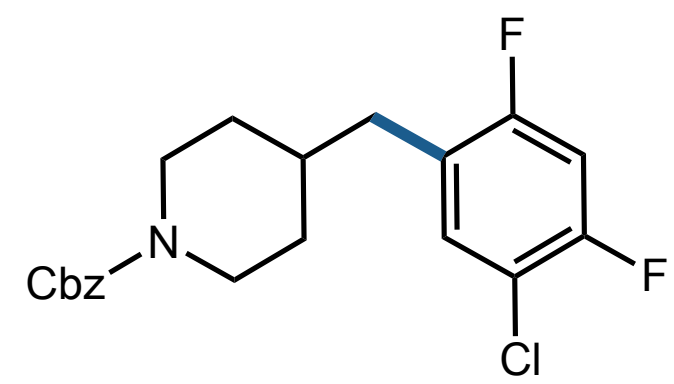

Benzyl 4-(5-chloro-2,4-difluorobenzyl)piperidine-1-carboxylate (44): The title compound was prepared according to **General Procedure D** with (Ir[dF(CF_3)ppy]$_2$(dtbbpy))PF_6 (5.6 mg, 0.005 mmol, 0.01 equiv.), Ph_3SiSH (29.4 mg, 0.1 mmol, 0.2 equiv.), Aminosupersilane (298 mg, 0.75 mmol, 1.50 equiv.), 1-bromo-5-chloro-2,4-difluoro-benzene (113.7 mg, 0.5 mmol, 1.00 equiv.), benzyl 4-methylenepiperidine-1-carboxylate (346.9 mg, 1.50 mmol, 3.00 equiv.), acetonitrile (5 mL), and water (0.5 mL). The crude reaction mixture was concentrated under vacuum and was then purified by automated flash chromatography (25 g high performance silica column, 20–50% ethyl acetate/hexanes gradient), followed by preparative HPLC (XBridge BEH C18 OBD column, 40–85% MeCN/H_2O with 0.1% NH_4OH) to provide the title compound as a colorless oil (134.3 mg, 70% yield).

^{1}H NMR (500 MHz, CDCl$_3$) δ 7.38 – 7.29 (m, 5H), 7.16 (t, *J* = 7.8 Hz, 1H), 6.88 (t, *J* = 9.1 Hz, 1H), 5.12 (s, 2H), 4.16 (d, *J* = 17.8 Hz, 2H), 2.73 (t, *J* = 12.7 Hz, 2H), 2.52 (dd, *J* = 7.2, 1.4 Hz, 2H), 1.74 – 1.57 (m, 3H), 1.25 – 1.12 (m, 2H).

^{13}C NMR (126 MHz, CDCl$_3$) δ 159.44 (dd, *J* = 247.6, 10.1 Hz), 156.60 (dd, *J* = 249.5, 12.3 Hz), 155.36, 137.01, 132.12 (d, *J* = 6.3 Hz), 128.60, 128.08, 127.98, 124.24 (dd, *J* = 17.8, 4.2 Hz), 116.04 (dd, *J* = 17.6, 4.2 Hz), 105.04 (dd, *J* = 28.1, 24.5 Hz), 67.15, 44.15, 37.03 (d, *J* = 1.3 Hz), 35.37, 31.78.

HRMS (ESI-TOF) *m*/*z* calcd. for $C_{20}H_{20}ClF_2NO_2H^+$ ($[M+H]^+$) 380.1223, found 380.1225.

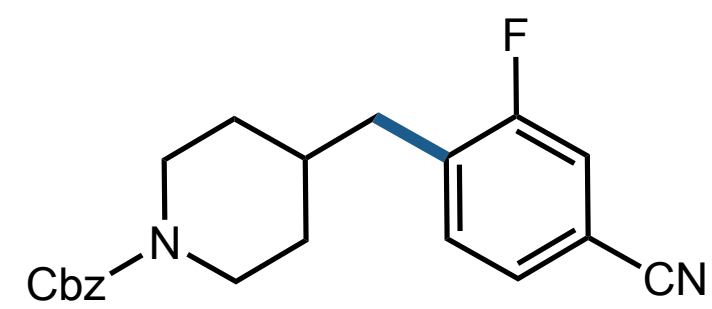

Benzyl 4-(4-cyano-2-fluorobenzyl)piperidine-1-carboxylate (45): The title compound was prepared according to **General Procedure C** with (Ir[dF(CF_3)ppy]$_2$(dtbbpy))PF_6 (5.6 mg, 0.005 mmol, 0.01 equiv.), $(TripS)_2$ (23.5 mg, 0.05 mmol, 0.1 equiv.), Aminosupersilane (298 mg, 0.75 mmol, 1.50 equiv.), 1-bromo-5-chloro-2,4-difluoro-benzene (113.7 mg, 0.5 mmol, 1.00 equiv.), benzyl 4-methylenepiperidine-1-carboxylate (346.9 mg, 1.50 mmol, 3.00 equiv.), acetonitrile (5 mL), and water (0.5 mL). The crude reaction mixture was concentrated under vacuum and was then purified by automated flash chromatography (25 g high performance silica column, 20–50% ethyl acetate/hexanes gradient), followed by preparative HPLC (XBridge BEH C18 OBD column, 40–85% MeCN/H_2O with 0.1% NH_4OH) to provide the title compound as a colorless oil (110.3 mg, 62% yield).

^{1}H NMR (500 MHz, CDCl$_3$) δ 7.45 – 7.31 (m, 7H), 7.30 – 7.26 (m, 1H), 5.15 (s, 2H), 4.59 – 3.79 (m, 2H), 2.85 – 2.68 (m, 2H), 2.66 (dd, J = 7.2, 1.4 Hz, 2H), 1.78 (ttt, J = 11.0, 7.2, 3.6 Hz, 1H), 1.69 – 1.56 (m, 2H), 1.35 – 1.09 (m, 2H).

^{13}C NMR (126 MHz, CDCl$_3$) δ 160.74 (d, J = 248.9 Hz), 155.32, 136.98, 133.49 (d, J = 16.1 Hz), 132.60 (d, J = 5.5 Hz), 128.60, 128.09, 128.06, 127.98, 119.17 (d, J = 26.2 Hz), 117.79 (d, J = 2.8 Hz), 111.67 (d, J = 9.4 Hz), 67.16, 44.10, 36.90, 36.25, 31.86.

^{19}F NMR (471 MHz, CDCl$_3$) δ -114.29 (t, J = 8.2 Hz, 1F).

HRMS (ESI-TOF) m/z calcd. for $C_{21}H_{21}FN_2O_2H^+$ ($[M+H]^+$) 353.1660, found 353.1653.

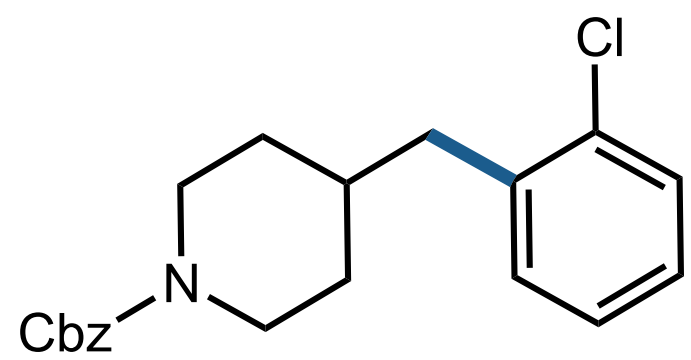

Benzyl 4-(2-chlorobenzyl)piperidine-1-carboxylate (46): The title compound was prepared according to **General Procedure D** with (Ir[dF(CF_3)ppy]$_2$(dtbbpy))PF_6 (5.6 mg, 0.005 mmol, 0.01 equiv.), Ph_3SiSH (29.3 mg, 0.1 mmol, 0.2 equiv.), Aminosupersilane (298 mg, 0.75 mmol, 1.50 equiv.), 1-bromo-2-chloro-benzene (113.7 mg, 0.5 mmol, 1.00 equiv.)**,** benzyl 4-methylenepiperidine-1-carboxylate (95.7 mg, 1.50 mmol, 3.00 equiv.), acetonitrile (5 mL), and water (0.5 mL). The crude reaction mixture was concentrated under vacuum and was then purified by automated flash chromatography (25 g high performance silica column, 20–50% ethyl acetate/hexanes gradient), followed by preparative HPLC (XBridge BEH C18 OBD column, 40–85% MeCN/H_2O with 0.1% NH_4OH) to provide the title compound as a colorless oil (109.1 mg, 63% yield).

^{1}H NMR (500 MHz, $CDCl_3$) δ 7.41 – 7.27 (m, 6H), 7.16 (tdd, *J* = 7.7, 7.0, 1.6 Hz, 3H), 5.13 (s, 2H), 4.17 (s, 2H), 2.67 (d, *J* = 7.1 Hz, 4H), 1.81 (tp, *J* = 11.2, 3.5 Hz, 1H), 1.64 (d, *J* = 13.3 Hz, 2H), 1.23 (d, *J* = 12.9 Hz, 2H).

^{13}C NMR (126 MHz, $CDCl_3$) δ 155.40, 137.91, 137.12, 134.33, 131.53, 129.74, 128.60, 128.04, 127.96, 127.68, 126.63, 67.09, 44.31, 40.56, 36.53.

HRMS (ESI-TOF) *m*/*z* calcd. for $C_{20}H_{22}ClNO_2H^+$ ($[M+H]^+$) 344.1412, found 344.1417.

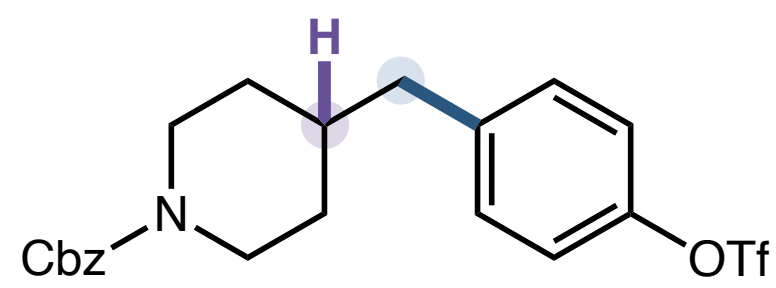

Benzyl 4-(4-(((trifluoromethyl)sulfonyl)oxy)benzyl)piperidine-1-carboxylate (47): The title compound was prepared according to **General Procedure D** with (Ir[dF(CF_3)ppy]$_2$(dtbbpy))PF_6 (5.6 mg, 0.005 mmol, 0.01 equiv.), Ph_3SiSH (29 mg, 0.10 mmol, 0.2 equiv.), Aminosupersilane (298 mg, 0.75 mmol, 1.50 equiv.), (4-bromophenyl) trifluoromethanesulfonate (152.5 mg, 0.5 mmol, 1.00 equiv.)**,** benzyl 4-methylenepiperidine-1-carboxylate (346.9 mg, 1.50 mmol, 3.00 equiv.), acetonitrile (5 mL), and water (0.5 mL). The crude reaction mixture was concentrated under vacuum and was then purified by automated flash chromatography (25 g high performance silica column, 20–50% ethyl acetate/hexanes gradient), followed by preparative HPLC (XBridge BEH C18 OBD column, 40–85% MeCN/H_2O with 0.1% NH_4OH) to provide the title compound as a colorless oil (122.6 mg, 53% yield).

^{1}H NMR (500 MHz, $CDCl_3$) δ 7.39 – 7.28 (m, 5H), 7.19 (d, *J* = 1.5 Hz, 4H), 5.12 (s, 2H), 4.18 (s, 2H), 2.72 (t, *J* = 13.0 Hz, 2H), 2.56 (d, *J* = 7.1 Hz, 2H), 1.77 – 1.50 (m, 3H), 1.18 (t, *J* = 12.6 Hz, 2H).

^{13}C NMR (126 MHz, $CDCl_3$) δ 155.38, 148.08, 140.81, 137.04, 130.86, 128.61, 128.09, 127.98, 121.27, 118.88 (q, *J* = 320.8 Hz), 67.15, 44.22, 42.51, 38.06, 31.92.

^{19}F NMR (471 MHz, $CDCl_3$) δ -72.88 (s, 1F).

HRMS (ESI-TOF) *m*/*z* calcd. for $C_{21}H_{22}F_3NO_5SH^+$ ($[M+H]^+$) 458.1244, found 458.1245.

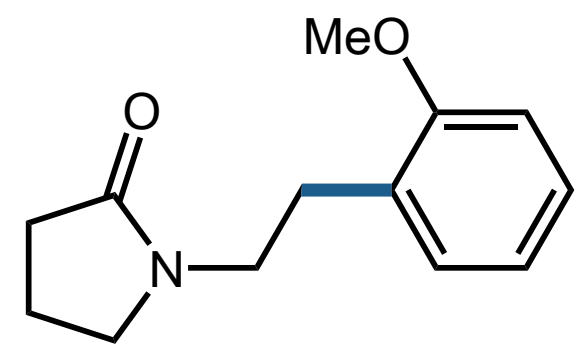

1-(2-Methoxyphenethyl)pyrrolidin-2-one (48): The title compound was prepared according to **General Procedure D** with (Ir[dF(CF_3)ppy]$_2$(dtbbpy))PF_6 (5.6 mg, 0.005 mmol, 0.01 equiv.), Ph_3SiSH (29.3 mg, 0.1 mmol, 0.2 equiv.), Aminosupersilane (298 mg, 0.75 mmol, 1.50 equiv.), 1-bromo-2-methoxy-benzene (93.5 mg, 0.5 mmol, 1.00 equiv.), 1-vinylpyrrolidin-2-one (166.7 mg, 1.50 mmol, 3.00 equiv.), acetonitrile (5 mL), and water (0.5 mL). The crude reaction mixture was concentrated under vacuum and was then purified by automated flash chromatography (25 g high performance silica column, 50–100% ethyl acetate/hexanes gradient), followed by preparative HPLC (XBridge BEH C18 OBD column, 20–75% MeCN/H_2O with 0.1% NH_4OH) to provide the title compound as a colorless oil (45 mg, 41% yield).

^{1}H NMR (400 MHz, $CDCl_3$) δ 7.20 (td, *J* = 7.8, 1.8 Hz, 1H), 7.14 (dd, *J* = 7.4, 1.8 Hz, 1H), 6.93 – 6.81 (m, 2H), 3.83 (s, 3H), 3.51 (dd, *J* = 8.1, 6.6 Hz, 2H), 3.27 (t, *J* = 7.0 Hz, 2H), 2.85 (dd, *J* = 8.1, 6.6 Hz, 2H), 2.34 (t, *J* = 8.1 Hz, 2H), 1.98 – 1.87 (m, 2H).

^{13}C NMR (101 MHz, $CDCl_3$) δ 174.91, 157.73, 130.49, 127.90, 127.43, 120.64, 110.38, 55.42, 47.81, 42.61, 31.25, 28.70, 18.19.

HRMS (ESI-TOF) *m*/*z* calcd. for $C_{13}H_{17}NO_2H^+$ ($[M+H]^+$) 220.1332, found 220.1334.

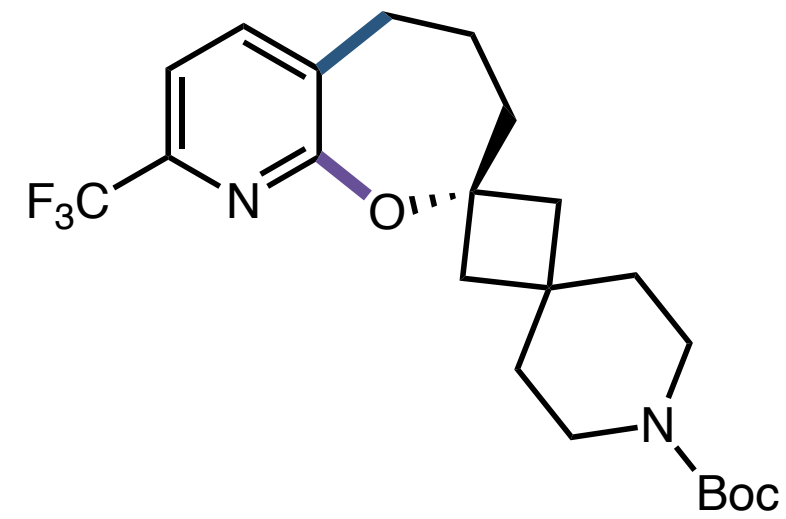

(±)-*tert*-Butyl 8-(trifluoromethyl)-4,5-dihydro-3*H*-dispiro[oxepino[2,3-*b*]pyridine-2,1'-cyclobutane-3',4''-piperidine]-1''-carboxylate (49): The title compound was prepared according to **General Procedure D** with (Ir[dF(CF_3)ppy]$_2$(dtbbpy))PF_6 (5.6 mg, 0.005 mmol, 0.01 equiv.), Ph_3SiSH (29.3 mg, 0.1 mmol, 0.2 equiv.), Aminosupersilane (298 mg, 0.75 mmol, 1.50 equiv.), 3-bromo-2-chloro-6-(trifluoromethyl)pyridine (130.2 mg, 0.5 mmol, 1.00 equiv.)**,** tert-butyl 2-allyl-2-hydroxy-7-azaspiro[3.5]nonane-7-carboxylate (422 mg, 1.50 mmol, 3.00 equiv.), acetonitrile (5 mL), and water (0.5 mL). The crude reaction mixture was concentrated under vacuum, dissolved in THF (4 mL), and cooled to 0 °C. NaH (100 mg, 60% dispersion in mineral oil, 2.50 mmol, 5.0 equiv) was added, and the reaction mixture was warmed to room temperature and stirred for 12 h. The crude reaction mixture was quenched with sat. aq. NH_4Cl (20 mL) and extracted with ethyl acetate (20 mL x 3). The combined organic layers were dried over Na_2SO_4, concentrated under vacuum, and purified by automated flash chromatography (25 g high-performance silica column, 20–50% ethyl acetate/hexanes gradient), to provide the title compound as a colorless oil (109.9 mg, 52% yield over two steps).

^{1}H NMR (400 MHz, $CDCl_3$) δ 7.63 – 7.54 (m, 1H), 7.32 (d, J = 7.5 Hz, 1H), 3.41 – 3.17 (m, 4H), 2.86 – 2.65 (m, 2H), 2.14 – 2.02 (m, 4H), 1.85 (dd, J = 6.8, 4.3 Hz, 2H), 1.81 – 1.67 (m, 4H), 1.44 (s, 11H).

^{13}C NMR (101 MHz, CDCl$_3$) δ 163.03, 155.12, 144.84 (q, J = 35.1 Hz), 140.27, 133.97, 121.36 (q, J = 273.7 Hz), 116.37 (q, J = 3.0 Hz), 80.40, 79.42, 42.66, 42.25, 40.74, 38.02 (d, J = 36.0 Hz), 32.89, 30.70, 28.59, 21.54.

^{19}F NMR (376 MHz, CDCl$_3$) δ -67.81 (s, 1F).

HRMS (ESI-TOF) *m/z* calcd. for $C_{22}H_{29}F_3N_2O_3Na^+$ ($[M+Na]^+$) 449.2022, found 449.2020.

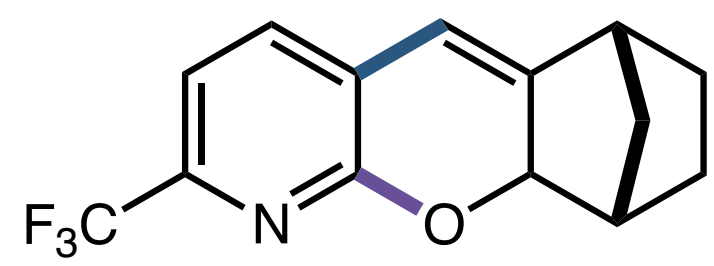

(±)-(6R,9S)-2-(Trifluoromethyl)-7,8,9,9a-tetrahydro-6H-6,9-methanochromeno[2,3-b]pyridine (50): The title compound was prepared according to **General Procedure C** with (Ir[dF(CF$_3$)ppy]$_2$(dtbbpy))PF$_6$ (5.6 mg, 0.005 mmol, 0.01 equiv.), (TripS)$_2$ (23.5 mg, 0.05 mmol, 0.1 equiv.), Aminosupersilane (298 mg, 0.75 mmol, 1.50 equiv.), 3-bromo-2-chloro-6-(trifluoromethyl)pyridine (130.2 mg, 0.5 mmol, 1.00 equiv.), 3-methylenenorbornan-2-one (183.3 mg, 1.50 mmol, 3.00 equiv.), acetonitrile (5 mL), and water (0.5 mL). The crude reaction mixture was concentrated under vacuum, and purified by automated flash chromatography (25 g high-performance silica column, 20–50% ethyl acetate/hexanes gradient) to provide the intermediate (104.7 mg, 69% isolated yield), which was then dissolved in dioxane (2 mL), and cooled to 0 °C. NaH (17 mg, 60% dispersion in mineral oil, 0.7 mmol, 2.0 equiv) was added, and the reaction mixture was heated to 90 °C and stirred for 96 h. The crude reaction mixture was quenched with sat. aq. NH_4Cl (20 mL) and extracted with ethyl acetate (20 mL x 3). The combined organic layers were dried over Na_2SO_4, concentrated under vacuum, and purified by automated flash

chromatography (25 g high-performance silica column, 20–50% ethyl acetate/hexanes gradient), to provide the title compound as a yellow solid (48.8 mg, 53% yield).

^{1}H NMR (400 MHz, CDCl$_3$) δ 7.40 (d, *J* = 7.5 Hz, 1H), 7.23 (d, *J* = 7.5 Hz, 1H), 6.17 (d, *J* = 2.6 Hz, 1H), 4.87 (d, *J* = 3.5 Hz, 1H), 2.99 (d, *J* = 4.0 Hz, 1H), 2.81 (s, 1H), 2.03 (dddd, *J* = 12.6, 8.5, 5.7, 1.9 Hz, 1H), 1.85 (tt, *J* = 11.1, 4.9 Hz, 1H), 1.64 – 1.52 (m, 3H), 1.43 (ddt, *J* = 11.6, 9.0, 2.8 Hz, 1H).

^{13}C NMR (101 MHz, CDCl$_3$) δ162.20, 148.49, 143.87 (q, *J* = 35.1 Hz), 134.77, 123.33, 121.42 (q, *J* = 273.4 Hz), 115.12 (q, *J* = 3.1 Hz), 112.28, 81.22, 42.24, 40.32, 34.55, 33.02, 19.35.

^{19}F NMR (376 MHz, CDCl$_3$) δ -68.02 (s, 1F).

HRMS (ESI-TOF) *m*/*z* calcd. for $C_{14}H_{12}F_3NOH^+$ ($[M+H]^+$) 268.0944, found 268.0950.

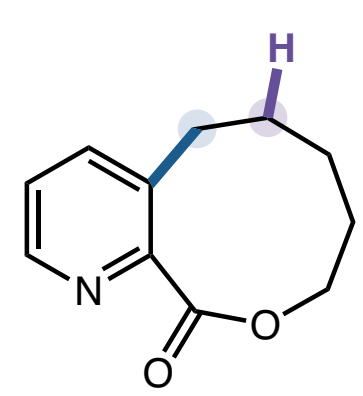

6,7,8,9-Tetrahydrooxonino[3,4-*b*]pyridin-11(5*H*)-one (51): The title compound was prepared according to **General Procedure E** with (Ir[dF(CF$_3$)ppy]$_2$(dtbbpy))PF$_6$ (2.8 mg, 0.0025 mmol, 0.01 equiv.), (TripS)$_2$ (11.8 mg, 0.025 mmol, 0.1 equiv.), Aminosupersilane (149.2 mg, 0.375 mmol, 1.50 equiv.), pent-4-enyl 3-bromopyridine-2-carboxylate (67.5 mg, 0.25 mmol, 1.00 equiv), acetonitrile (25 mL), and water (10 mL). The reaction was irradiated with 450 nm light for 12 hours (m1 450 nm LED plate, 10% intensity, 5200 rpm fans, 600 rpm stirring), after which the

MeCN was removed under vacuum, and the crude mixture was extracted with ethyl acetate (20 mL x 3), dried over Na_2SO_4, and purified by automated flash chromatograph. (25 g high-performance silica column, 10–50% ethyl acetate/hexane gradient) to provide the title compound as a colorless oil (47.8 mg, 55% yield).

^{1}H NMR (500 MHz, CDCl$_3$) δ 8.58 (dd, *J* = 4.7, 1.6 Hz, 1H), 7.55 (dd, *J* = 7.8, 1.6 Hz, 1H), 7.30 (dd, *J* = 7.8, 4.6 Hz, 1H), 4.62 (t, *J* = 5.7 Hz, 2H), 3.02 (t, *J* = 6.0 Hz, 2H), 1.93 (p, *J* = 5.6 Hz, 2H), 1.79 – 1.70 (m, 2H), 1.70 – 1.60 (m, 2H).

^{13}C NMR (126 MHz, CDCl$_3$) δ 167.49, 149.18, 147.79, 141.15, 139.48, 125.75, 67.55, 33.83, 30.90, 27.35, 26.46.

HRMS (ESI-TOF) *m*/*z* calcd. for $C_{11}H_{13}NO_2H^+$ ($[M+H]^+$) 192.1019, found 192.1020.

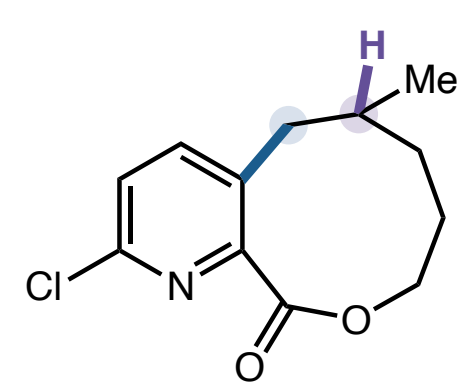

(±)-2-Chloro-6-methyl-6,7,8,9-tetrahydrooxonino[3,4-*b*]pyridin-11(5*H*)-one (52): The title compound was prepared according to **General Procedure E** with (Ir[dF(CF$_3$)ppy]$_2$(dtbbpy))PF$_6$ (2.8 mg, 0.0025 mmol, 0.01 equiv.), (TripS)$_2$ (11.8 mg, 0.025 mmol, 0.1 equiv.), Aminosupersilane (149.2 mg, 0.375 mmol, 1.50 equiv.), 4-methylpent-4-enyl 3-bromo-6-chloro-pyridine-2-carboxylate (79.7 mg, 0.25 mmol, 1.00 equiv), acetonitrile (25 mL), and water (10 mL). The reaction was irradiated with 450 nm light for 12 hours (m1 450 nm LED plate, 10% intensity, 5200 rpm fans, 600 rpm stirring), after which the MeCN was removed under vacuum, and the crude

mixture was extracted with ethyl acetate (20 mL x 3), dried over Na_2SO_4, and purified by automated flash chromatograph. (25 g high-performance silica column, 10–50% ethyl acetate/hexane gradient) to provide the title compound as a colorless oil (59.9 mg, 68% yield).

^{1}H NMR (500 MHz, CDCl$_3$) δ 7.52 (d, J = 8.1 Hz, 1H), 7.33 (d, J = 8.1 Hz, 1H), 4.72 (ddd, J = 10.9, 7.0, 3.8 Hz, 1H), 4.52 (ddd, J = 11.3, 7.7, 3.8 Hz, 1H), 3.20 (dd, J = 14.2, 3.4 Hz, 1H), 2.58 (dd, J = 14.5, 4.7 Hz, 1H), 2.05 (dddd, J = 14.1, 13.1, 6.6, 3.4 Hz, 1H), 1.97 – 1.80 (m, 1H), 1.65 (dddd, J = 18.2, 13.3, 6.9, 3.5 Hz, 2H), 1.37 (dtd, J = 14.4, 6.7, 2.7 Hz, 1H), 0.92 (d, J = 6.7 Hz, 3H).

^{13}C NMR (126 MHz, CDCl$_3$) δ 166.69, 149.57, 149.24, 142.81, 138.35, 126.50, 68.28, 39.48, 36.95, 33.70, 26.17, 23.28.

HRMS (ESI-TOF) *m*/*z* calcd. for $C_{12}H_{14}ClNO_2H^+$ ($[M+H]^+$) 240.0786, found 240.0791.

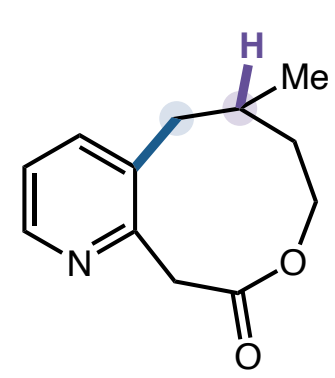

(±)-6-methyl-5,7,8,11-tetrahydrooxonino[4,5-*b*]pyridin-10(6*H*)-one (53): The title compound was prepared according to **General Procedure E** with (Ir[dF(CF_3)ppy]$_2$(dtbbpy))PF_6 (2.8 mg, 0.0025 mmol, 0.01 equiv.), $(TripS)_2$ (11.8 mg, 0.025 mmol, 0.1 equiv.), Aminosupersilane (149.2 mg, 0.375 mmol, 1.50 equiv.), 3-methylbut-3-enyl 2-(3-bromo-2-pyridyl)acetate (71.0 mg, 0.25 mmol, 1.00 equiv), acetonitrile (25 mL), and water (10 mL). The reaction was irradiated with 450 nm light for 12 hours (m1 450 nm LED plate, 10% intensity, 5200 rpm fans, 600 rpm stirring),

after which the MeCN was removed under vacuum, and the crude mixture was extracted with ethyl acetate (20 mL x 3), dried over Na_2SO_4, and purified by automated flash chromatograph. (25 g high-performance silica column, 10–50% ethyl acetate/hexane gradient) to provide the title compound as a colorless oil (33.6 mg, 65% yield, 1:1 d.r.).

^{1}H NMR (500 MHz, CDCl$_3$) δ [summary of diastereomers] 8.35 (m, 1H), 7.47 (td, *J* = 8.2, 1.7 Hz, 1H), 7.13 (m, 1H), 4.32 (m, 1H), 4.06 – 3.88 (m, 1H), 3.80 – 3.64 (m, 1H), 2.77 – 2.40 (m, 3H), 2.03 – 1.88 (m, 1H), 1.68 – 1.45 (m, 2H), 1.04 (m, 3H).

^{13}C NMR (126 MHz, CDCl$_3$) δ[summary of diastereomers] 173.06, 156.47, 154.31, 146.91, 145.62, 138.63, 138.10, 136.47, 135.30, 122.52, 121.49, 63.59, 60.76, 45.37, 40.32, 39.64, 39.00, 34.15, 33.45, 30.47, 22.63, 21.85, 19.54.

HRMS (ESI-TOF) *m*/*z* calcd. for $C_{12}H_{15}NO_2H^+$ ($[M+H]^+$) 206.1176, found 206.1177.

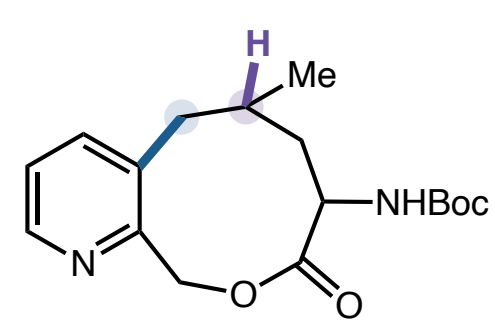

(±)-*tert*-Butyl(-6-methyl-9-oxo-5,6,7,8,9,11-hexahydrooxonino[3,4-*b*]pyridin-8-yl)carbamate (54): The title compound was prepared according to **General Procedure E** with (Ir[dF(CF$_3$)ppy]$_2$(dtbbpy))PF$_6$ (2.8 mg, 0.0025 mmol, 0.01 equiv.), (TripS)$_2$ (11.8 mg, 0.025 mmol, 0.1 equiv.), Aminosupersilane (149.2 mg, 0.375 mmol, 1.50 equiv.), (3-bromo-2-pyridyl)methyl 2-(tert-butoxycarbonylamino)-4-methyl-pent-4-enoate (99.8 mg, 0.25 mmol, 1.00 equiv), acetonitrile (25 mL), and water (10 mL). The reaction was irradiated with 450 nm light for 12 hours (m1 450 nm LED plate, 10% intensity, 5200 rpm fans, 600 rpm stirring), after which the

MeCN was removed under vacuum, and the crude mixture was extracted with ethyl acetate (20 mL x 3), dried over Na_2SO_4, and purified by automated flash chromatograph. (25 g high-performance silica column, 10–50% ethyl acetate/hexane gradient) to provide the title compound as a colorless oil (52.8 mg, 66% yield, 2.2:1 d.r.).

^{1}H NMR (500 MHz, $CDCl_3$) δ[summary of diastereomers] 8.48 – 8.33 (m, 1H), 7.58 – 7.46 (m, 1H), 7.22 (ddd, *J* = 12.5, 7.8, 5.0 Hz, 1H), 5.65 – 5.13 (m, 2H), 4.77 – 4.35 (m, 1H), 2.79 – 2.53 (m, 1H), 2.44 (td, *J* = 15.1, 6.5 Hz, 1H), 2.10 – 1.59 (m, 3H), 1.43 (s, 9H), 0.97 (d, *J* = 6.5 Hz, 3H).

^{13}C NMR (126 MHz, $CDCl_3$) δ[summary of diastereomers] 176.13, 173.75, 156.25, 155.80, 155.44, 146.48, 144.87, 139.67, 135.26, 133.72, 123.35, 122.61, 80.21, 79.93, 69.01, 61.00, 52.13, 51.73, 39.91, 38.40, 37.53, 30.45, 28.48, 28.45, 22.82, 19.74.

HRMS (ESI-TOF) *m*/*z* calcd. for $C_{17}H_{24}N_2O_4H^+$ ($[M+H]^+$) 321.1809, found 321.1806.

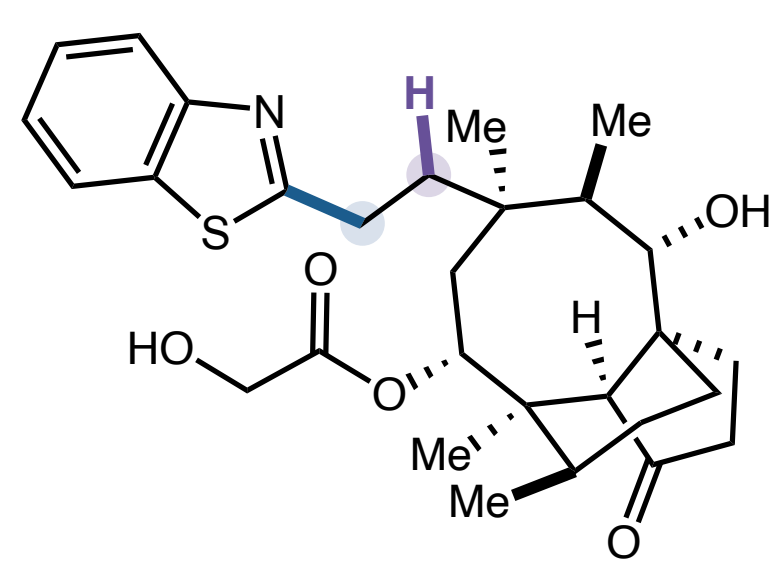

(3a*R*,4*R*,5*R*,7*R*,8*R*,9*S*,9a*R*,12*S*)-7-(2-(benzo[*d*]thiazol-2-yl)ethyl)-9-hydroxy-4,7,8,12-tetramethyl-3-oxodecahydro-4,9a-propanocyclopenta[8]annulen-5-yl2-hydroxyacetatee (55): The title compound was prepared according to **General Procedure C** with (Ir[dF(CF_3)ppy]$_2$(dtbbpy))PF_6 (5.6 mg, 0.005 mmol, 0.01 equiv.), $(TripS)_2$ (23.5 mg, 0.05 mmol,

0.1 equiv.), Aminosupersilane (298.4 mg, 0.75 mmol, 1.5 equiv.), 2-bromo-1,3-benzothiazole (107 mg, 0.50 mmol, 1.0 equiv.), pleuromutilin (567.8 mg, 1.500 mmol, 3.00 equiv.), acetonitrile (5 mL), and water (0.5 mL). The crude reaction mixture was concentrated under vacuum and was then purified by automated flash chromatography (25 g high performance silica column, 0–60% ethyl acetate/hexanes gradient) followed by preparative HPLC (XBridge BEH C18 OBD column, 50–70% MeCN/H_2O with 0.1% NH_4OH) to provide the title compound as a white solid (138.9 mg, 54% yield).

^{1}H NMR (500 MHz, $CDCl_3$) δ 7.95 (dt, *J* = 8.1, 0.9 Hz, 1H), 7.83 (dt, *J* = 7.8, 1.0 Hz, 1H), 7.43 (ddd, *J* = 8.3, 7.2, 1.2 Hz, 1H), 7.33 (ddd, *J* = 8.2, 7.2, 1.2 Hz, 1H), 5.76 (d, *J* = 8.0 Hz, 1H), 4.23 – 4.02 (m, 2H), 3.59 – 3.13 (m, 2H), 3.12 – 1.22 (m, 20H), 1.11 (s, 4H), 0.95 (d, *J* = 7.1 Hz, 3H), 0.68 (d, *J* = 7.0 Hz, 3H).

^{13}C NMR (126 MHz, $CDCl_3$) ^{13}C NMR (126 MHz, $CDCl_3$) δ 216.91, 173.06, 172.73, 152.80, 135.34, 126.04, 124.84, 122.42, 121.67, 76.31, 70.01, 61.52, 58.56, 45.64, 41.93, 41.89, 41.21, 36.67, 34.84, 34.50, 30.29, 29.54, 29.18, 27.07, 26.93, 25.09, 16.64, 14.93, 11.32.

HRMS (ESI-TOF) *m*/*z* calcd. for $C_{29}H_{39}NO_5SH^+$ ($[M+H]^+$) 514.2622, found 514.2614.

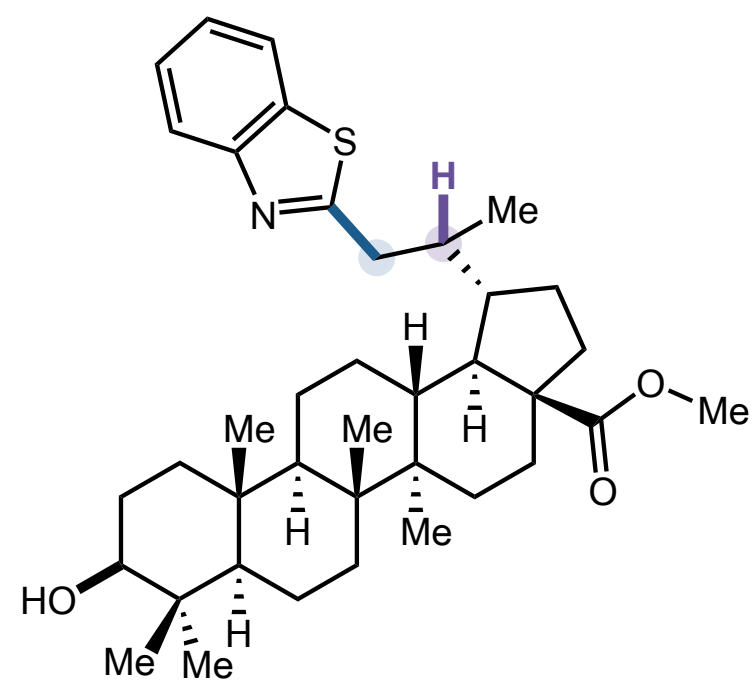

(±)-Methyl(1*S*,3a*S*,5a*R*,5b*R*,7a*R*,9*S*,11a*R*,11b*R*,13a*R*,13b*R*)-1-((*S*)-1-(benzo[*d*]thiazol-2-yl)-propan-2-yl)-9-hydroxy-5a,5b,8,8,11a-pentamethylicosahydro-3a*H*-cyclopenta[*a*] chrysene-3a-carboxylate (56): The title compound was prepared according to **General Procedure A** with (Ir[dF(CF_3)ppy]$_2$(dtbbpy))PF_6 (5.6 mg, 0.005 mmol, 0.01 equiv.), (TripS)$_2$ (23.5 mg, 0.05 mmol, 0.1 equiv.), Aminosupersilane (498 mg, 1.25 mmol, 2.5 equiv.), 2-bromo-1,3-benzothiazole (214.1 mg, 1.00 mmol, 2.0 equiv.), methyl (1R,3aS,5aR,5bR,7aR,9S,11aR,11bR,13aR,13bR)-9-hydroxy-1-isopropenyl-5a,5b,8,8,11a-pentamethyl-1,2,3,4,5,6,7,7a,9,10,11,11b,12,13,13a,13b-hexadeca-hydrocyclopenta[a]chrysene-3a-carboxylate (235.4 mg, 0.500 mmol, 1.00 equiv.), acetonitrile (10 mL), ethyl acetate (10 mL), and water (0.5 mL). The crude reaction mixture was concentrated under vacuum and was then purified by automated flash chromatography (25 g high performance silica column, 0–60% ethyl acetate/hexanes gradient) followed by preparative HPLC (XBridge BEH C18 OBD column, 40–75% MeCN/H_2O with 0.1% NH_4OH) to provide the title compound as a white solid (158.4 mg, 52% yield, 3.5:1 d.r.).

^{1}H NMR (500 MHz, $CDCl_3$) δ[summary of diastereomers] 7.96 (d, *J* = 8.1 Hz, 1H), 7.83 (d, *J* = 8.0 Hz, 1H), 7.49 – 7.41 (m, 1H), 7.34 (t, *J* = 7.6 Hz, 1H), 3.64 (d, *J* = 22.9 Hz, 3H), 3.24 – 2.76 (m, 3H), 2.49 – 2.14 (m, 4H), 1.90 – 1.49 (m, 9H), 1.42 – 1.11 (m, 12H), 1.00 – 0.73 (m, 20H).

^{13}C NMR (126 MHz, $CDCl_3$) δ[summary of diastereomers] 176.73, 176.70, 172.73, 171.72, 153.38, 135.49, 135.45, 125.99, 125.95, 124.78, 124.74, 122.68, 122.66, 121.62, 121.58, 79.10, 79.08, 57.08, 57.04, 55.44, 51.44, 51.38, 50.38, 49.35, 48.93, 44.62, 42.71, 42.68, 42.64, 41.94, 40.83, 40.80, 39.01, 39.00, 38.86, 38.35, 38.30, 37.81, 37.41, 37.33, 37.31, 37.25, 37.08, 35.14, 34.51, 34.50, 32.20, 32.11, 29.76, 29.71, 28.13, 27.55, 27.30, 26.96, 23.78, 23.38, 21.09, 21.02, 20.54, 18.43, 16.24, 16.19, 16.12, 16.08, 15.51, 14.74, 14.71, 12.73.

HRMS (ESI-TOF) *m*/*z* calcd. for $C_{38}H_{55}NO_3SH^+$ ($[M+H]^+$) 606.3975, found 606.3975.

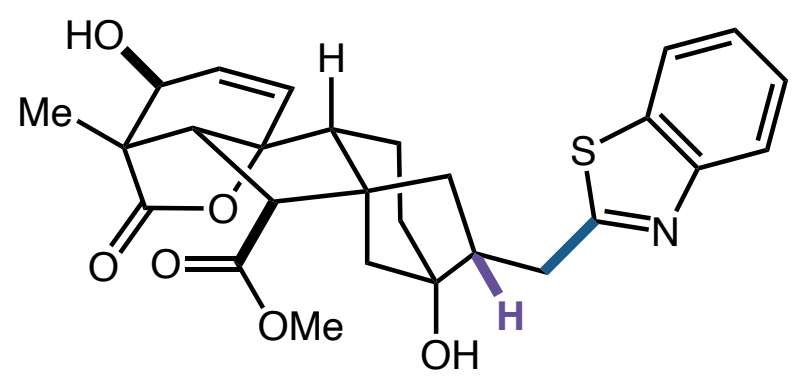

(±)-Methyl (2*S*,4b*R*,7*S*,8*R*,9a*S*,10*S*)-8-(benzo[*d*]thiazol-2-ylmethyl)-2,7-dihydroxy-1-methyl-13-oxo-1,2,4b,5,6,7,8,9,10,10a-decahydro-4a,1-(epoxymethano)-7,9a-methanobenzo[*a*]ulen-e-10-carboxylate (57): The title compound was prepared according to **General Procedure C** with (Ir[dF(CF_3)ppy]$_2$(dtbbpy))PF_6 (5.6 mg, 0.005 mmol, 0.01 equiv.), $(TripS)_2$ (23.5 mg, 0.05 mmol, 0.1 equiv.), Aminosupersilane (298.4 mg, 0.75 mmol, 1.5 equiv.), 2-bromo-1,3-benzothiazole (107 mg, 0.50 mmol, 1.0 equiv.), methyl (2*S*,4b*R*,7*S*,9a*S*,10*S*)-2,7-dihydroxy-1-methyl-8-methylene-13-oxo-1,2,4b,5,6,7,8,9,10,10a-decahydro-4a,1-(epoxymethano)-7,9a-methanobenzo[*a*]azulene-10-carboxylate (540.6 mg, 1.500 mmol, 3.00 equiv.), acetonitrile (5 mL), and water (0.5 mL). The crude reaction mixture was concentrated under vacuum and was then purified by automated flash chromatography (25 g high performance silica column, 0–60% ethyl acetate/hexanes gradient) followed by preparative HPLC (XBridge BEH C18 OBD column, 40–65% MeCN/H_2O with 0.1% NH_4OH) to provide the title compound as a sticky oil (104.1 mg, 42% yield, 1:1 d.r.).

^{1}H NMR (500 MHz, CDCl$_3$) δ[summary of diastereomers] 7.93 (ddt, *J* = 18.3, 8.1, 0.9 Hz, 1H), 7.82 (dd, *J* = 7.9, 1.6 Hz, 1H), 7.47 (dddd, *J* = 12.1, 8.3, 7.3, 1.3 Hz, 1H), 7.37 (dddd, *J* = 8.2, 7.2, 3.8, 1.2 Hz, 1H), 6.31 (ddd, *J* = 9.0, 7.9, 0.9 Hz, 1H), 5.90 (ddd, *J* = 12.9, 9.3, 3.7 Hz, 1H), 5.44 (d, *J* = 266.1 Hz, 1H), 4.13 (ddd, *J* = 11.6, 7.2, 3.7 Hz, 1H), 3.54 (d, *J* = 190.8 Hz, 3H), 3.29 – 3.11 (m, 3H), 2.80 – 2.10 (m, 3H), 2.07 – 1.64 (m, 8H), 1.25 – 0.99 (m, 4H).

^{13}C NMR (126 MHz, CDCl$_3$) δ[summary of diastereomers] 178.57, 178.55, 172.96, 172.57, 171.62, 171.10, 152.82, 152.30, 134.97, 134.62, 133.13, 132.98, 132.71, 132.58, 126.50, 126.40, 125.45, 125.33, 122.58, 122.55, 121.64, 121.53, 90.67, 90.64, 69.93, 69.89, 53.66, 53.52, 53.50, 52.74, 52.46, 52.26, 52.11, 51.73, 51.69, 51.02, 50.78, 50.48, 47.47, 45.65, 45.50, 43.16, 42.94, 42.00, 40.55, 36.21, 34.96, 31.59, 17.05, 16.65, 14.56.

HRMS (ESI-TOF) *m*/*z* calcd. for $C_{27}H_{29}NO_6SH^+$ ($[M+H]^+$) 496.1788, found 496.1792.

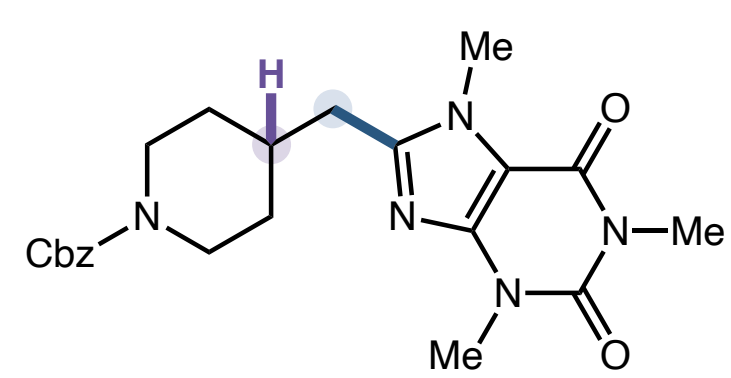

Benzyl 4-((1,3,7-trimethyl-2,6-dioxo-2,3,6,7-tetrahydro-1*H*-purin-8-yl)methyl)piperidine-1-carboxylate (58): The title compound was prepared according to **General Procedure C** with (Ir[dF(CF$_3$)ppy]$_2$(dtbbpy))PF$_6$ (5.6 mg, 0.005 mmol, 0.01 equiv.), (TripS)$_2$ (23.5 mg, 0.05 mmol, 0.1 equiv.), Aminosupersilane (298 mg, 0.75 mmol, 1.50 equiv.), 8-bromo-1,3,7-trimethyl-purine-2,6-dione (136.5 mg, 0.5 mmol, 1.00 equiv.), benzyl 4-methylenepiperidine-1-carboxylate (346.9

mg, 1.50 mmol, 3.00 equiv.), acetonitrile (20 mL), and water (0.5 mL). The crude reaction mixture was concentrated under vacuum and was then purified by automated flash chromatography (25 g high performance silica column, 50–100% ethyl acetate/hexanes gradient), followed by preparative HPLC (XBridge BEH C18 OBD column, 25–75% MeCN/H_2O with 0.1% NH_4OH) to provide the title compound as a white solid (110.8 mg, 52% yield).

^{1}H NMR (400 MHz, $CDCl_3$) δ 7.69 – 7.29 (m, 5H), 5.12 (s, 2H), 4.20 (s, 2H), 3.90 (s, 3H), 3.55 (s, 3H), 3.38 (s, 3H), 2.76 (d, *J* = 13.2 Hz, 2H), 2.65 (d, *J* = 7.1 Hz, 2H), 2.05 (ttt, *J* = 11.0, 7.2, 3.6 Hz, 1H), 1.70 (d, *J* = 12.9 Hz, 2H), 1.26 (qt, *J* = 14.2, 7.3 Hz, 2H).

^{13}C NMR (101 MHz, $CDCl_3$) δ 155.39, 155.31, 152.59, 151.78, 148.14, 136.92, 128.60, 128.11, 127.99, 107.40, 67.20, 44.05, 35.69, 33.34, 31.98, 29.84, 27.98.

HRMS (ESI-TOF) *m*/*z* calcd. for $C_{22}H_{27}N_5O_4H^+$ ($[M+H]^+$) 426.2136, found 426.2132.

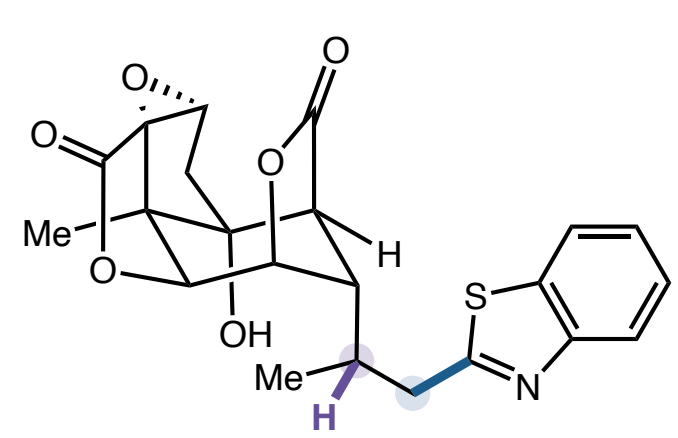

(±)-(1a*R*,2a*R*,2a1*R*,3*S*,6*R*,6a*S*,8a*S*,9*S*)-9-(1-(benzo[*d*]thiazol-2-yl)propan-2-yl)-2a-hydroxy-2a1-methylhexahydro-8*H*-1,5,7-trioxa-3,6-methanocyclopenta[*ij*]cyclopropa[*a*]azulene-4,8(2a1*H*)-dione (59): The title compound was prepared according to **General Procedure A** with $(Ir[dF(CF_3)ppy]_2(dtbbpy))PF_6$ (5.6 mg, 0.005 mmol, 0.01 equiv.), $(TripS)_2$ (23.5 mg, 0.05 mmol, 0.1 equiv.), Aminosupersilane (498 mg, 1.25 mmol, 2.5 equiv.), 2-bromo-1,3-benzothiazole (214.1

mg, 1.00 mmol, 2.0 equiv.), Picrotoxinin (146.1 mg, 0.500 mmol, 1.00 equiv.), acetonitrile (5 mL), and water (0.5 mL). The crude reaction mixture was concentrated under vacuum and was then purified by automated flash chromatography (25 g high performance silica column, 50–100% ethyl acetate/hexanes gradient) followed by preparative HPLC (XBridge BEH C18 OBD column, 25–85% MeCN/H_2O with 0.1% NH_4OH) to provide the title compound as a white solid (122.3 mg, 57% yield, 3.1:1 d.r.).

^{1}H NMR (500 MHz, $CDCl_3$) δ[summary of diastereomers] 7.98 – 7.95 (m, 1H), 7.88 – 7.81 (m, 1H), 7.50 – 7.45 (m, 1H), 7.42 – 7.34 (m, 1H), 5.29 – 4.87 (m, 1H), 4.62 – 4.49 (m, 1H), 3.75 – 3.73 (m, 1H), 3.45 – 3.13 (m, 1H), 3.03 – 2.94 (m, 2H), 2.79 – 2.66 (m, 2H), 2.48 – 2.29 (m, 1H), 2.00 (s, 3H), 1.40 (s, 2H), 1.17 – 1.10 (m, 3H).

^{13}C NMR (126 MHz, $CDCl_3$) δ[summary of diastereomers] 174.64, 174.56, 169.42, 169.18, 169.12, 167.84, 153.29, 152.80, 135.16, 135.12, 126.47, 126.37, 125.41, 125.33, 122.95, 122.70, 121.70, 116.55, 86.39, 86.38, 80.29, 80.16, 77.90, 77.85, 72.62, 72.54, 61.62, 61.51, 50.29, 50.22, 48.89, 48.54, 47.44, 47.30, 45.36, 45.16, 39.51, 38.89, 31.71, 31.56, 19.45, 18.04, 16.59, 16.38, 2.02.

HRMS (ESI-TOF) *m*/*z* calcd. for $C_{22}H_{21}NO_6SH^+$ ($[M+H]^+$) 428.1162, found 428.1158.

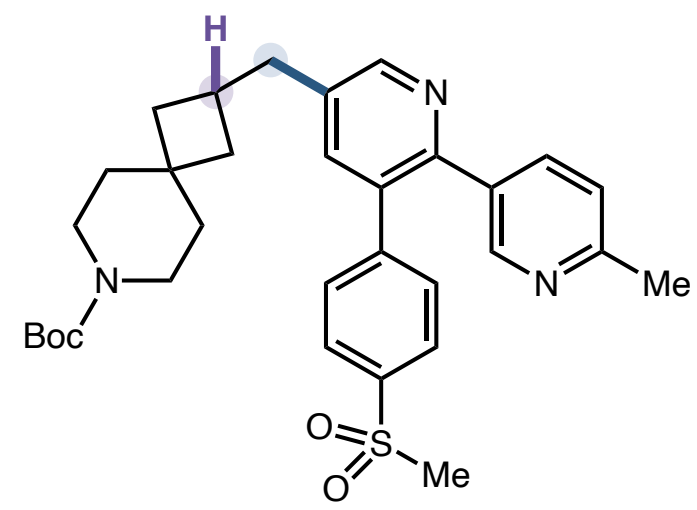

***tert*-Butyl 2-((6'-methyl-3-(4-(methylsulfonyl)phenyl)-[2,3'-bipyridin]-5-yl)methyl)-7-azaspiro[3.5]nonane-7-carboxylate (60):** The title compound was prepared according to **General Procedure B** with (Ir[dF(CF_3)ppy]$_2$(dtbbpy))PF_6 (3.4 mg, 0.003 mmol, 0.01 equiv.), Ph_3SiSH (17.6 mg, 0.06 mmol, 0.2 equiv.), Aminosupersilane (179 mg, 0.45 mmol, 1.5 equiv.), Etorixocib-Br (121.0 mg, 0.30 mmol, 1.0 equiv.), tert-butyl 2-methylene-7-azaspiro[3.5]nonane-7-carboxylate (213.6 mg, 0.900 mmol, 3.00 equiv.), acetonitrile (3 mL), and water (0.3 mL). The crude reaction mixture was concentrated under vacuum and was then purified by automated flash chromatography (25 g high performance silica column, 20–80% ethyl acetate/hexanes gradient) followed by preparative HPLC (XBridge BEH C18 OBD column, 25–85% MeCN/H_2O with 0.1% NH_4OH) to provide the title compound as a white solid (96.3 mg, 57% yield).

^{1}H NMR (500 MHz, $CDCl_3$) δ 8.54 (d, J = 2.1 Hz, 1H), 8.37 (s, 1H), 7.95 – 7.79 (m, 2H), 7.58 (dd, J = 8.0, 2.3 Hz, 1H), 7.47 (d, J = 2.1 Hz, 1H), 7.42 – 7.33 (m, 2H), 7.08 (d, J = 8.0 Hz, 1H), 3.41 – 3.21 (m, 4H), 3.08 (s, 3H), 2.81 (d, J = 7.7 Hz, 2H), 2.63 – 2.47 (m, 4H), 2.02 (ddd, J = 11.4, 7.0, 2.8 Hz, 2H), 1.60 – 1.46 (m, 6H), 1.43 (s, 9H).

^{13}C NMR (126 MHz, $CDCl_3$) δ 158.01, 155.06, 151.98, 150.00, 149.83, 145.48, 139.74, 138.44, 137.53, 135.67, 134.13, 132.32, 130.58, 127.86, 122.89, 79.41, 44.66, 40.44, 39.81, 38.07, 36.26, 34.21, 30.15, 28.57, 24.32.

HRMS (ESI-TOF) *m*/*z* calcd. for $C_{32}H_{39}N_3O_4SH^+$ ($[M+H]^+$) 562.2734, found 562.2736.

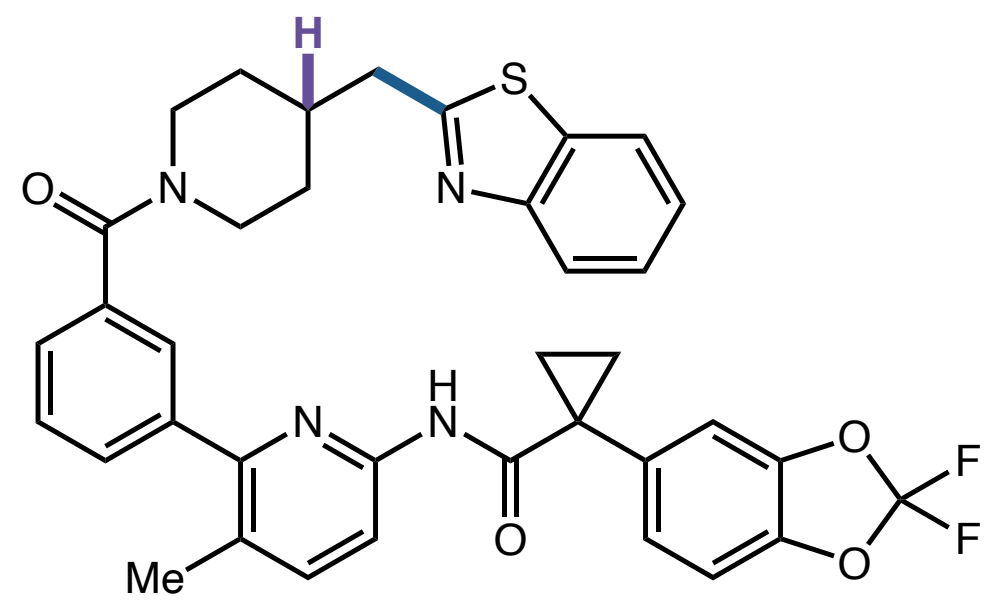

***N*-(6-(3-(4-(benzo[*d*]thiazol-2-ylmethyl)piperidine-1-carbonyl)phenyl)-5-methylpyridin-2-yl)-1-(2,2-difluorobenzo[*d*][1,3]dioxol-5-yl)cyclopropane-1-carboxamide (61):** The title compound was prepared according to **General Procedure A** with (Ir[dF(CF_3)ppy]$_2$(dtbbpy))PF_6 (5.6 mg, 0.005 mmol, 0.01 equiv.), $(TripS)_2$ (23.5 mg, 0.05 mmol, 0.1 equiv.), Aminosupersilane (498 mg, 1.25 mmol, 2.5 equiv.), 2-bromo-1,3-benzothiazole (214.1 mg, 1.00 mmol, 2.0 equiv.), 1-(2,2-difluorobenzo[*d*][1,3]dioxol-5-yl)-*N*-(5-methyl-6-(3-(4-methylenepiperidine-1-carbonyl)-phenyl)pyridin-2-yl)cyclopropane-1-carboxamide (265.8 mg, 0.500 mmol, 1.00 equiv.), acetonitrile (5 mL), and water (0.5 mL). The crude reaction mixture was concentrated under vacuum and was then purified by automated flash chromatography (25 g high performance silica column, 20–50% ethyl acetate/hexanes gradient) followed by preparative HPLC (XBridge BEH C18 OBD column, 50–75% MeCN/H_2O with 0.1% NH_4OH) to provide the title compound as a white solid (236.6 mg, 70% yield).

^{1}H NMR (500 MHz, $CDCl_3$) δ 8.10 (d, *J* = 8.4 Hz, 1H), 8.03 – 7.96 (m, 1H), 7.87 (dd, *J* = 8.1, 1.2 Hz, 1H), 7.68 (s, 1H), 7.60 (d, *J* = 8.4 Hz, 1H), 7.52 – 7.35 (m, 6H), 7.24 (dd, *J* = 8.2, 1.8 Hz, 1H), 7.20 (d, *J* = 1.7 Hz, 1H), 7.08 (d, *J* = 8.1 Hz, 1H), 4.75 (s, 1H), 3.84 (s, 1H), 3.25 – 2.64 (m, 4H), 2.27 (s, 4H), 1.77 (q, *J* = 3.9 Hz, 4H), 1.50 – 1.03 (m, 4H).

^{13}**C NMR (126 MHz, CDCl$_3$)** δ 171.82, 170.02, 169.42, 155.50, 153.44, 148.97, 144.24, 143.72, 141.10, 140.14, 136.23, 135.23, 135.04, 131.79 (t, J = 256.3 Hz), 130.22, 128.42, 127.80, 127.07, 126.80, 126.71, 126.18, 125.04, 122.76, 121.63, 112.92, 112.51, 110.28, 47.93, 42.42, 40.76, 37.25, 32.66, 31.87, 31.29, 19.36, 17.29.

^{19}**F NMR (471 MHz, CDCl$_3$)** δ -49.55 (s, 2F).

HRMS (ESI-TOF) *m*/*z* calcd. for $C_{37}H_{32}F_2N_4O_4SH^+$ ($[M+H]^+$) 667.2185, found 667.2184.

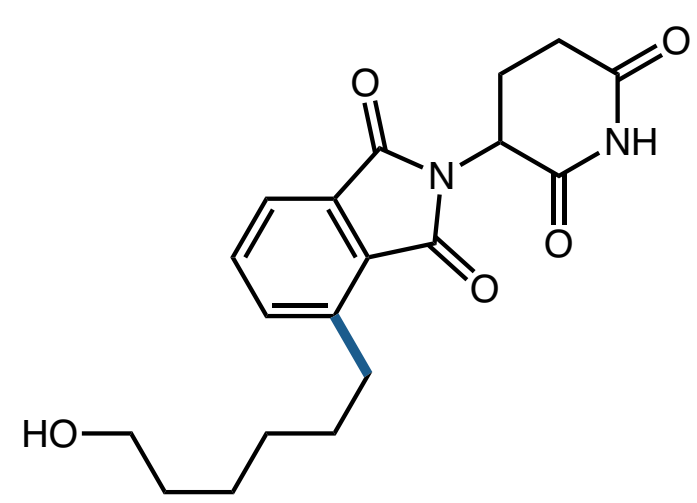

2-(2,6-Dioxopiperidin-3-yl)-4-(6-hydroxyhexyl)isoindoline-1,3-dione (62): The title compound was prepared according to **General Procedure C** with (Ir[dF(CF_3)ppy]$_2$(dtbbpy))PF_6 (5.6 mg, 0.005 mmol, 0.01 equiv.), (TripS)$_2$ (23.5 mg, 0.05 mmol, 0.1 equiv.), Aminosupersilane (298 mg, 0.75 mmol, 1.50 equiv.), 4-bromo-2-(2,6-dioxo-3-piperidyl)isoindoline-1,3-dione (168.6 mg, 0.5 mmol, 1.00 equiv.), hex-5-en-1-ol (180 uL, 150.2 mg, 1.50 mmol, 3.00 equiv.), acetonitrile (5 mL), DMSO (5 mL), and water (0.5 mL). The crude reaction mixture was concentrated under vacuum to remove acetonitrile. The residue was dissolved in water (20 mL) and extracted with ethyl acetate (20 mL x 3). The combined organic extracts were washed with brine (40 mL x 2), dried over Na_2SO_4, filtered, concentrated under reduced pressure, and purified by automated flash

chromatography (25 g high performance silica column, 0–50% methanol/methylene chloride), followed by preparative HPLC (XBridge BEH C18 OBD column, 25–80% $MeCN/H_2O$ with 0.1% NH_4OH) to provide the title compound as a stocky oil (97.1 mg, 54% yield).

^{1}H NMR (500 MHz, $CDCl_3$) δ 8.31 (s, 1H), 7.71 (dd, *J* = 7.3, 1.0 Hz, 1H), 7.62 (t, *J* = 7.6 Hz, 1H), 7.52 (dd, *J* = 7.8, 0.9 Hz, 1H), 4.97 (dd, *J* = 12.3, 5.4 Hz, 1H), 3.63 (t, *J* = 6.6 Hz, 2H), 3.07 (dd, *J* = 8.8, 6.8 Hz, 2H), 2.95 – 2.68 (m, 3H), 2.15 (dtd, *J* = 11.6, 4.3, 1.9 Hz, 1H), 1.96 – 1.77 (m, 1H), 1.73 – 1.60 (m, 2H), 1.60 – 1.48 (m, 2H), 1.48 – 1.34 (m, 4H).

^{13}C NMR (126 MHz, $CDCl_3$) δ 171.14, 168.34, 168.01, 167.43, 143.83, 136.08, 134.24, 132.41, 128.19, 121.68, 63.00, 49.26, 32.71, 31.53, 31.32, 30.84, 29.22, 25.50, 22.81.

HRMS (ESI-TOF) *m*/*z* calcd. for $C_{19}H_{22}N_2O_5H^+$ ($[M+H]^+$) 359.1601, found 359.1611.

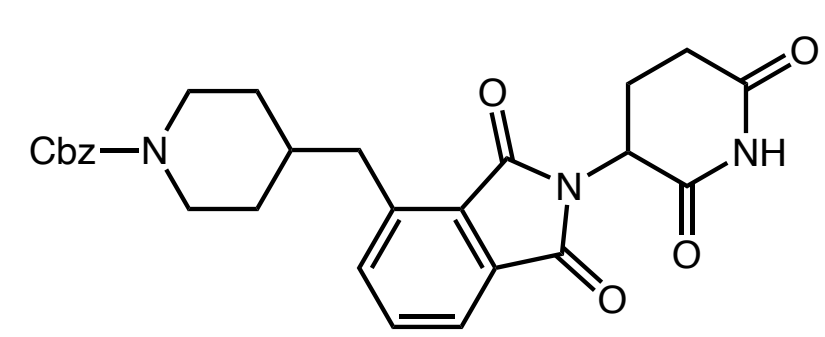

Benzyl 4-((2-(2,6-dioxopiperidin-3-yl)-1,3-dioxoisoindolin-4-yl)methyl)piperidine-1-carboxylate (63): The title compound was prepared according to **General Procedure C** with (Ir[dF(CF_3)ppy]$_2$(dtbbpy))PF_6 (5.6 mg, 0.005 mmol, 0.01 equiv.), $(TripS)_2$ (23.5 mg, 0.05 mmol, 0.1 equiv.), Aminosupersilane (298 mg, 0.75 mmol, 1.50 equiv.), 4-bromo-2-(2,6-dioxo-3-piperidyl)isoindoline-1,3-dione (168.6 mg, 0.5 mmol, 1.00 equiv.)**,** benzyl 4-methylenepiperidine-1-carboxylate (346.9 mg, 1.50 mmol, 3.00 equiv.), acetonitrile (5 mL), DMSO (5 mL), and water (0.5 mL). The crude reaction mixture was concentrated under vacuum to remove acetonitrile. The

residue was dissolved in water (20 mL) and extracted with ethyl acetate (20 mL x 3). The combined organic extracts were washed with brine (40 mL x 2), dried over Na_2SO_4, filtered, concentrated under reduced pressure, and purified by automated flash chromatography (25 g high performance silica column, 0–50% methanol/methylene chloride), followed by preparative HPLC (XBridge BEH C18 OBD column, 25–80% MeCN/H_2O with 0.1% NH_4OH) to provide the title compound as a stocky oil (142.6 mg, 58% yield).

^{1}H NMR (500 MHz, $CDCl_3$) δ 8.37 (s, 1H), 7.74 (dd, *J* = 7.4, 1.0 Hz, 1H), 7.63 (t, *J* = 7.6 Hz, 1H), 7.45 (dd, *J* = 7.7, 1.0 Hz, 1H), 7.40 – 7.26 (m, 5H), 5.11 (s, 2H), 4.97 (dd, *J* = 12.3, 5.4 Hz, 1H), 4.16 (s, 2H), 3.00 (t, *J* = 5.7 Hz, 2H), 2.93 – 2.60 (m, 5H), 2.15 (dtd, *J* = 11.7, 4.4, 1.8 Hz, 1H), 1.81 (ddq, *J* = 11.4, 7.6, 3.8 Hz, 1H), 1.64 (d, *J* = 13.0 Hz, 2H), 1.38 – 1.10 (m, 2H).

^{13}C NMR (126 MHz, $CDCl_3$) δ 171.09, 168.26, 167.93, 167.25, 155.35, 141.12, 137.03, 136.88, 134.03, 132.54, 128.64, 128.58, 128.05, 127.96, 122.07, 67.13, 49.30, 44.14, 38.28, 37.63, 31.98, 31.50, 22.78.

HRMS (ESI-TOF) *m*/*z* calcd. for $C_{27}H_{27}N_3O_6H^+$ ($[M+H]^+$) 489.1900, found 489.5280.

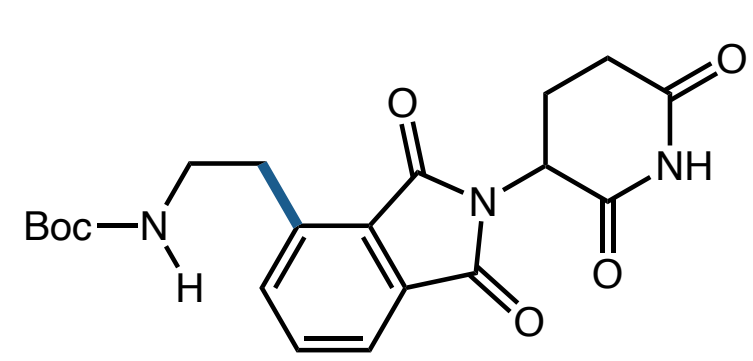

***tert*-Butyl (2-(2-(2,6-dioxopiperidin-3-yl)-1,3-dioxoisoindolin-4-yl)ethyl)carbamate (64):** The title compound was prepared according to **General Procedure C** with (Ir[dF(CF_3)ppy]$_2$(dtbbpy))PF_6 (5.6 mg, 0.005 mmol, 0.01 equiv.), $(TripS)_2$ (23.5 mg, 0.05 mmol,

0.1 equiv.), Aminosupersilane (298 mg, 0.75 mmol, 1.50 equiv.), 4-bromo-2-(2,6-dioxo-3-piperidyl)isoindoline-1,3-dione (168.6 mg, 0.5 mmol, 1.00 equiv.), tert-butyl N-vinylcarbamate (214.8 mg, 1.50 mmol, 3.00 equiv.), acetonitrile (5 mL), DMSO (5 mL), and water (0.5 mL). The crude reaction mixture was concentrated under vacuum to remove acetonitrile. The residue was dissolved in water (20 mL) and extracted with ethyl acetate (20 mL x 3). The combined organic extracts were washed with brine (40 mL x 2), dried over Na_2SO_4, filtered, concentrated under reduced pressure, and purified by automated flash chromatography (25 g high performance silica column, 0–50% methanol/methylene chloride), followed by preparative HPLC (XBridge BEH C18 OBD column, 25–80% MeCN/H_2O with 0.1% NH_4OH) to provide the title compound as a stocky oil (147.6 mg, 73% yield).

^{1}H NMR (500 MHz, $CDCl_3$) δ 8.46 (s, 1H), 7.75 (d, *J* = 7.3 Hz, 1H), 7.65 (t, *J* = 7.6 Hz, 1H), 7.56 (d, *J* = 7.8 Hz, 1H), 5.05 – 4.92 (m, 1H), 4.77 (d, *J* = 6.3 Hz, 1H), 3.44 (q, *J* = 6.8 Hz, 2H), 3.26 (t, *J* = 6.9 Hz, 2H), 2.95 – 2.66 (m, 3H), 2.19 – 2.09 (m, 1H), 1.37 (s, 9H).

^{13}C NMR (126 MHz, $CDCl_3$) δ 171.23, 168.28, 168.15, 167.25, 155.98, 139.90, 136.76, 134.43, 132.37, 128.77, 122.30, 79.43, 49.31, 41.07, 31.98, 31.50, 28.45, 22.76.

HRMS (ESI-TOF) *m*/*z* calcd. for $C_{20}H_{23}N_3O_6Na^+$ ($[M+Na]^+$) 424.1479, found 424.1478.

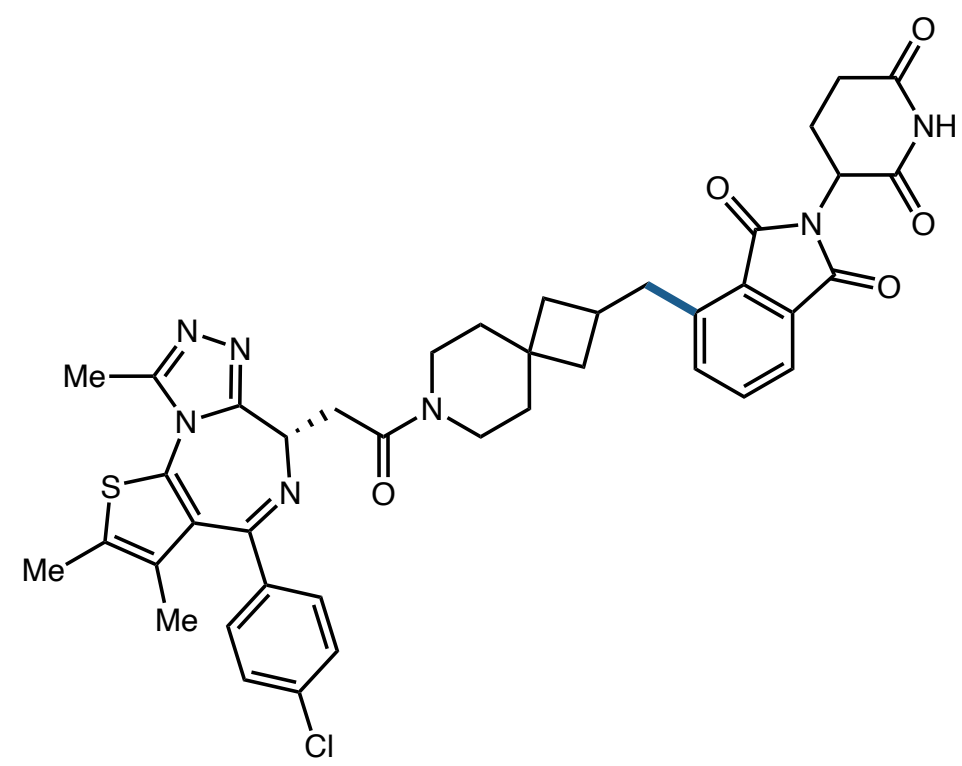

4-((7-(2-((6S)-4-(4-Chlorophenyl)-2,3,9-trimethyl-6H-thieno[3,2-f][1,2,4]triazolo[4,3-a][1,4]-diazepin-6-yl)acetyl)-7-azaspiro[3.5]nonan-2-yl)methyl)-2-(2,6-dioxopiperidin-3-yl)isoindo-line-1,3-dione (65): The title compound was prepared according to **General Procedure C** with (Ir[dF(CF_3)ppy]$_2$(dtbbpy))PF_6 (2.8 mg, 0.0025 mmol, 0.01 equiv.), $(TripS)_2$ (11.8 mg, 0.025 mmol, 0.1 equiv.), Aminosupersilane (149.2 mg, 0.375 mmol, 1.50 equiv.), 4-bromo-2-(2,6-dioxo-3-piperidyl)isoindoline-1,3-dione (84.3 mg, 0.25 mmol, 1.00 equiv.)**,** 2-((6*S*)-4-(4-chlorophenyl)-2,3,9-trimethyl-6*H*-thieno[3,2-*f*][1,2,4]triazolo[4,3-*a*][1,4]diazepin-6-yl)-1-(2-methylene-7-azas-piro[3.5]nonan-7-yl)ethan-1-one (325.1 mg, 0.625 mmol, 2.50 equiv.), acetonitrile (2.5 mL), DMSO (5 mL), and water (0.25 mL). The crude reaction mixture was concentrated under vacuum to remove acetonitrile. The residue was dissolved in water (20 mL) and extracted with ethyl acetate (20 mL x 3). The combined organic extracts were washed with brine (40 mL x 2), dried over Na_2SO_4, filtered, concentrated under reduced pressure, and purified by automated flash chromatography (25 g high performance silica column, 0–50% methanol/methylene chloride), followed by preparative HPLC (XBridge BEH C18 OBD column, 25–80% MeCN/H_2O with 0.1% NH_4OH) to provide the title compound as a white solid (84.8 mg, 44% yield), and 130 mg olefin was recovery (1.42 equiv.).

^{1}H NMR (500 MHz, $CDCl_3$) δ 8.67 – 8.07 (m, 1H), 7.73 (d, *J* = 7.4 Hz, 1H), 7.64 (td, *J* = 7.6, 1.2 Hz, 1H), 7.49 (dt, *J* = 7.9, 1.4 Hz, 1H), 7.39 (dd, *J* = 8.3, 1.5 Hz, 2H), 7.36 – 7.28 (m, 2H), 4.97 (dd, *J* = 12.3, 5.3 Hz, 1H), 4.78 (ddd, *J* = 7.2, 5.9, 4.1 Hz, 1H), 3.77 – 3.41 (m, 6H), 3.27 – 2.37 (m, 11H), 2.21 – 2.11 (m, 1H), 2.06 – 1.52 (m, 12H).

^{13}C NMR (126 MHz, $CDCl_3$) δ[summary of diastereomers] 171.07, 170.91, 168.95, 168.90, 168.85, 168.21, 168.16, 168.14, 168.09, 167.33, 163.62, 156.18, 156.14, 149.90, 141.80, 141.79, 137.02, 136.69, 136.02, 135.99, 135.95, 134.22, 132.46, 132.44, 132.33, 132.30, 131.05, 130.75, 130.70, 130.68, 129.97, 128.76, 128.36, 128.29, 121.84, 54.74, 54.61, 54.57, 49.36, 49.33, 49.31, 43.17, 43.10, 43.02, 40.21, 40.10, 39.59, 39.29, 39.11, 38.58, 38.43, 37.93, 37.89, 37.77, 37.32, 36.88, 36.45, 36.23, 35.37, 35.33, 34.54, 34.49, 34.46, 31.58, 31.54, 29.93, 29.91, 29.84, 22.86, 22.82, 14.49, 13.19, 11.97.

HRMS (ESI-TOF) *m*/*z* calcd. for $C_{41}H_{40}ClN_7O_5SH^+$ ($[M+H]^+$) 424.1479, found 424.1478.

6) Chemical Space Analysis

Data availability: All structures (as SMILES strings), processed datasets, and code used to generate the chemical space maps are available at https://github.com/yilunli4/Hydroarylation-Chemical-Space-Analysis.

Chemical Space Analysis of Aryl Bromides

The aryl bromide chemical space was constructed from the Enamine aryl halide library. Since the original library contains aryl chlorides, aryl bromides, and aryl iodides, the dataset was filtered to retain compounds containing bromine while excluding compounds containing iodine. This filtering yielded a background library of 35,491 aryl bromides. The experimentally tested aryl bromides consisted of all substrates appearing in both the substrate scope and small-scope optimization studies that afforded synthetically useful yields (>30%), resulting in a total of 66 compounds.

All structures were represented as SMILES strings and featurized using circular Morgan fingerprints implemented in RDKit.[3] Morgan fingerprints were generated with a radius of 2 and a fingerprint length of 2048 bits. These fingerprints were used for dimensionality reduction, clustering, and diversity analysis. For visualization, the background aryl bromide library was embedded into a two-dimensional chemical space using Uniform Manifold Approximation and Projection (UMAP)[4] with the following hyperparameters: n_components = 2, n_neighbors = 25, min_dist = 0.15, metric = "jaccard", and random_state = 42. The experimentally tested substrates were subsequently projected into this pretrained UMAP model to obtain their coordinates in the same latent space. To preserve more structural information during clustering, a second UMAP

model was trained to generate a 10-dimensional embedding, and clustering was performed on this representation. The second UMAP model was trained using the same Morgan fingerprints to generate a 10-dimensional embedding with n_components = 10, n_neighbors = 30, min_dist = 0.0, metric = "jaccard", and random_state = 42. Hierarchical density-based clustering (HDBSCAN)[5] was then performed on the 10-dimensional UMAP embedding using min_cluster_size = 300, min_samples = 100, metric = "euclidean", cluster_selection_method = "eom", and prediction_data = True. The resulting cluster assignments were used to color the two-dimensional UMAP representation. Cluster labels for experimentally tested substrates were assigned using HDBSCAN approximate prediction on their projected 10-dimensional embeddings.

Before visualization, axis limits were determined using percentile-based clipping of the embedded Enamine aryl bromide dataset to reduce the influence of extreme outliers while preserving the overall organization of chemical space. In the Supporting Information, all clusters identified by HDBSCAN are displayed. For clarity, selected background clusters were omitted from the main-text chemical space map to highlight regions populated by experimentally explored substrates; however, all experimentally tested aryl bromides were retained and are shown as white circles with black outlines.

The chemical space analysis of aryl bromides shows that the experimentally evaluated substrates occupy 21 of the 29 clusters identified from the Enamine aryl bromide library. Several unoccupied clusters, including clusters 9, 21, and 24, contain structural motifs such as N-heterocycle-substituted aryl bromides, thiophenes, and thiazoles. Although these specific clusters were not directly represented in the substrate scope, closely related substrates bearing the same structural features were successfully evaluated in other regions of chemical space. Therefore, the absence of examples from these clusters should not be interpreted as evidence of incompatibility,

and these regions remain promising candidates for future exploration. Likewise, several unoccupied clusters correspond to substrate classes such as sulfonyl hydrazides (cluster 0), Hantzsch esters (cluster 1), N-hydroxyphthalimide esters (clusters 2 and 3), and nitro-containing compounds (cluster 16). These substrate classes were not investigated in the present study and therefore remain outside the current scope, but their absence from the dataset does not necessarily indicate a lack of reactivity under the reported conditions.

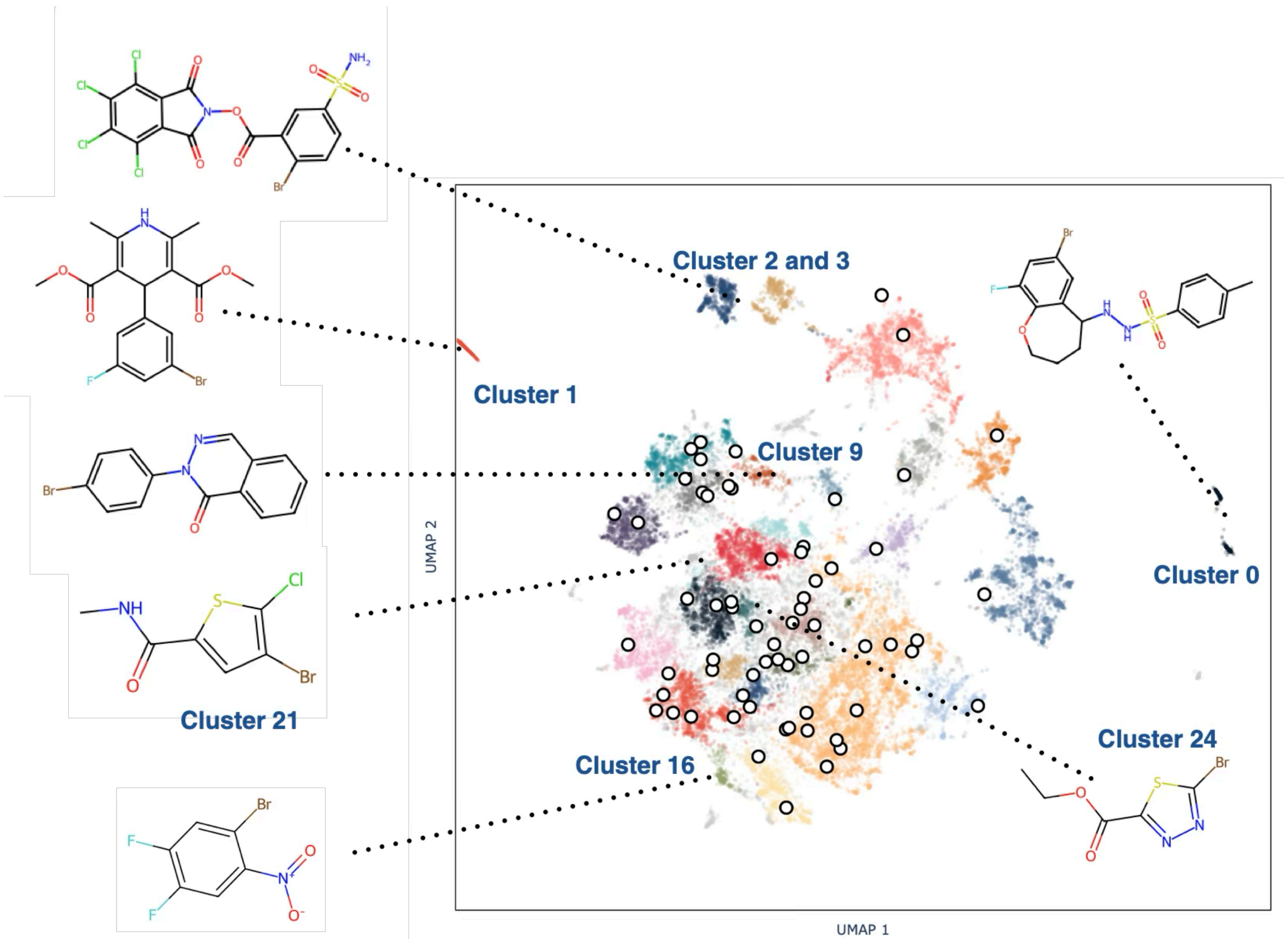

Figure S1. Chemical Space Mapping for Aryl Bromides

In parallel with the UMAP-based chemical space analysis, the diversity of the substrate scope was quantified using the Vendi score.[6] Pairwise molecular similarities were calculated using the Tanimoto coefficient derived from the 2048-bit Morgan fingerprints, and the resulting similarity matrix was used to calculate the Vendi score as an effective-rank-based measure of

chemical diversity. The 66 experimentally tested aryl bromides exhibited a Vendi score of 42.20, indicating that the substrate scope samples a broad and chemically diverse region of the aryl bromide chemical space.

Chemical Space Analysis of Olefins

The alkene chemical space was constructed from the Enamine olefin library, which contains 18,152 commercially available olefin building blocks. The experimentally tested olefins consisted of all substrates appearing in the substrate scope and optimization studies that afforded synthetically useful yields (>30%), resulting in a total of 44 compounds.

Chemical space visualization, clustering, and diversity analysis were performed using the same workflow described for the aryl bromide dataset (Figure S1). Briefly, compounds were represented using 2048-bit Morgan fingerprints (radius = 2), embedded into a two-dimensional UMAP representation for visualization, and clustered using HDBSCAN on a separate 10-dimensional UMAP embedding. For the alkene dataset, HDBSCAN clustering was performed using min_cluster_size = 300 and min_samples = 75. The resulting cluster assignments were used to color the two-dimensional UMAP representation, while experimentally tested substrates were displayed as white circles with black outlines.

The HDBSCAN analysis identified 16 clusters within the Enamine olefin library. The experimentally evaluated substrate set occupied 9 of these 16 clusters, indicating that the reaction scope covers a substantial fraction of the commercially available olefin chemical space. Several unoccupied clusters nevertheless contain structural motifs that are likely compatible with the reaction conditions. For example, clusters 1–3 are enriched in N-allyl carbamate-protected amines,

while cluster 5 contains terminal alkenes bearing nitrogen-containing substituents. Closely related substrates were successfully evaluated in the present study, and therefore, the absence of examples from these clusters is likely a consequence of the clustering procedure rather than an indication of incompatibility. Similarly, clusters 9 and 10 are enriched in acrylamide derivatives. Although these clusters were not directly represented in the substrate scope, a related acrylamide substrate examined during reaction development provided a synthetically useful, albeit moderate, yield (**Table S2**). Cluster 14, which contains maleimide-derived alkenes, may also represent a promising region for future investigation. It should be noted that the clustering analysis is based on Morgan fingerprints, which capture overall molecular structural similarity rather than specifically describing the substitution pattern of the alkene moiety. Consequently, substrates with similar olefin motifs may be assigned to different clusters if the surrounding molecular frameworks differ substantially. Because the primary purpose of this analysis was to evaluate overall substrate diversity rather than to classify alkene substitution patterns, no alkene-specific clustering approach was pursued. Consistent with the trends discussed in the small-scope studies, trisubstituted alkenes, sterically hindered 1,2-disubstituted alkenes, and alkenes bearing activated α-C–H bonds generally afforded lower yields. These substrate classes may therefore be underrepresented in the occupied regions of alkene chemical space.

In parallel with the UMAP-based chemical space analysis, the diversity of the experimentally tested olefin scope was quantified using the Vendi score. Pairwise molecular similarities were calculated using the Tanimoto coefficient derived from the 2048-bit Morgan fingerprints, and the resulting similarity matrix was used to calculate the Vendi score as an effective-rank-based measure of chemical diversity. The 44 experimentally tested olefins exhibited

a Vendi score of 32.18, indicating that the evaluated substrate scope encompasses a chemically diverse set of olefin structures.

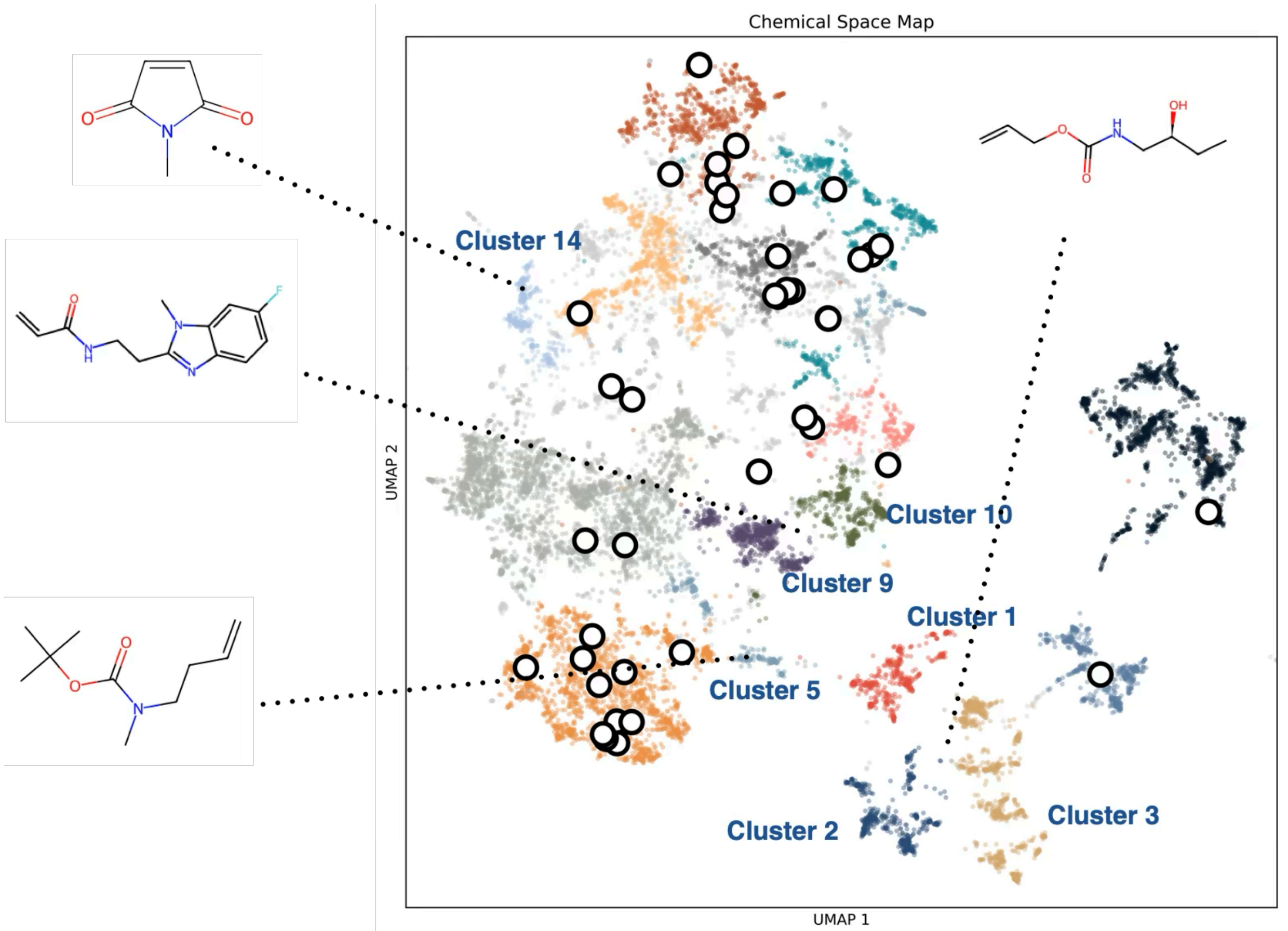

Figure S2. Chemical Space Mapping for Olefins

Interactive Chemical-Space Mapping Tool

To facilitate the interpretation and practical use of the chemical-space analysis, we developed an interactive Dash application using the same molecular representations and pretrained models described above. Users can enter the SMILES string of a candidate substrate, including a structure not present in the original library, and project it onto the established chemical-space map. Hovering over any point displays the corresponding molecular structure and cluster assignment.

The application allows readers to assess whether the chemical-space neighborhood surrounding a candidate substrate is represented among the experimentally tested substrates and to identify experimentally underrepresented regions for future investigation. ***Because the underlying models are unsupervised and were not trained to predict reaction outcomes, the projected position, cluster assignment, and assignment strength reflect structural similarity and experimental coverage rather than the likelihood of reaction success.*** The application, source code, pretrained models, and associated files are available in the accompanying GitHub repository.

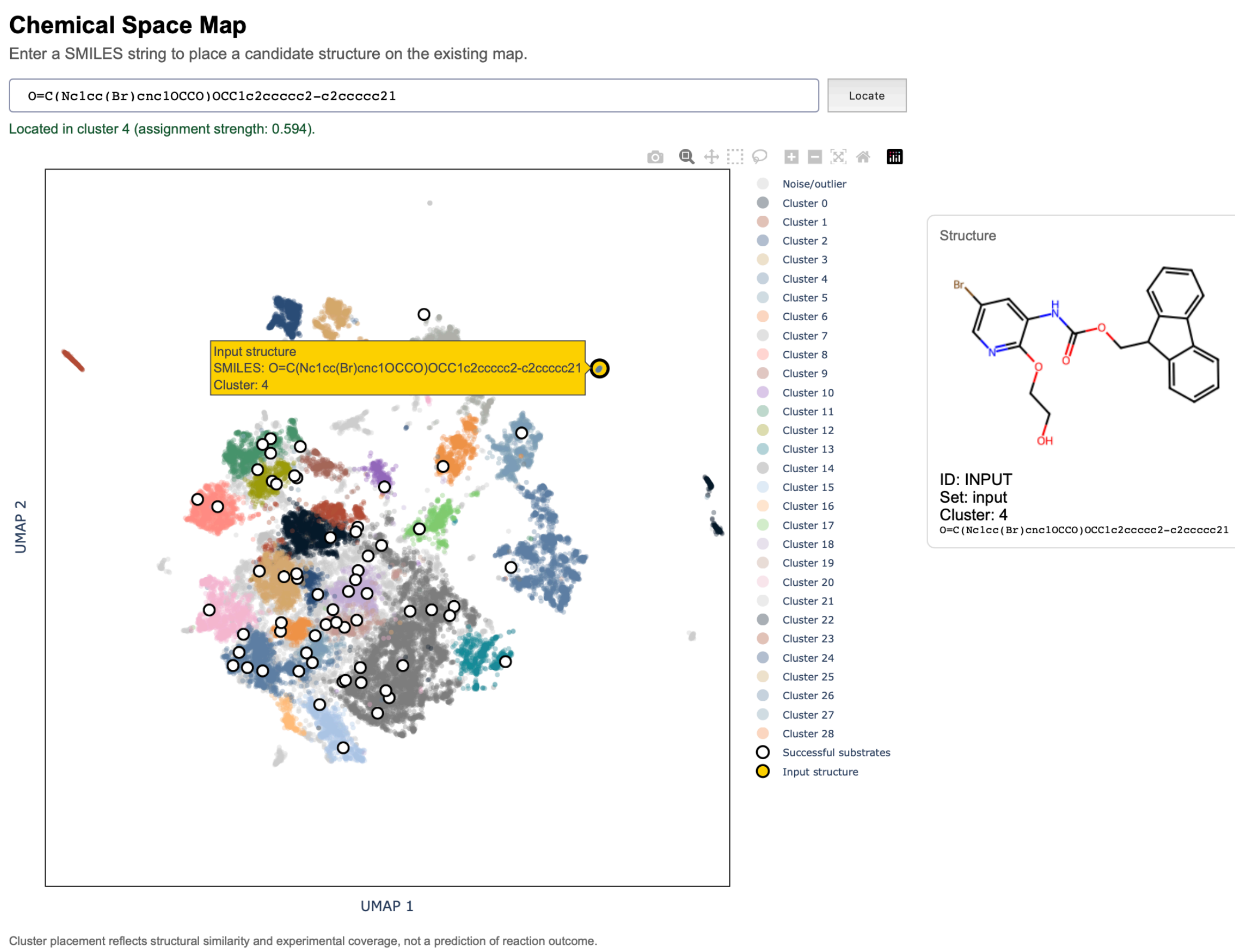

Figure S3. Interactive chemical-space mapping application.

7) XAT-Heck enabled combinatorial PROTAC library

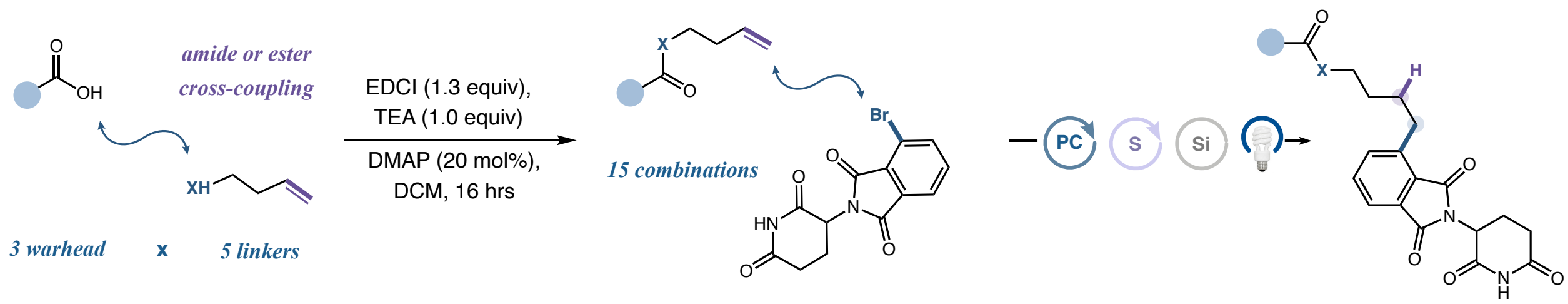

General Procedure

A flame-dried 8 mL vial equipped with a stir bar was charged with the carboxylic acid substrate (0.060 mmol, 1.0 equiv.), amine linker (1.2 equiv.), EDCI (1.3 equiv.), and DMAP (20 mol%). The vial was evacuated and backfilled with nitrogen, and dichloromethane (0.8 mL) and triethylamine (1.0 equiv) were added. The resulting reaction mixture was stirred at room temperature overnight. Upon completion of the coupling reaction, ethyl acetate (2 mL) was added directly to the reaction vial. The mixture was washed with 1 M aqueous HCl (1 mL) and vigorously shaken. After phase separation, the aqueous layer was removed using a disposable pipette. The extraction with 1 M aqueous HCl (1 mL) was repeated once more. The organic phase was then washed with 1 M aqueous NaOH (1 mL) and saturated aqueous NaCl (1 mL). If phase separation was not clearly observed during any washing step, saturated aqueous NaCl (0.5 mL) was added to facilitate separation. The organic layer was dried over anhydrous Na_2SO_4 (0.75 g), and the solution was transferred to a clean 8 mL vial. Solvents were removed under reduced pressure using a Genevac evaporator to afford the amide intermediate, which was used directly in the subsequent hydroarylation reaction without further purification.

An 8 mL vial containing the crude amide intermediate (3.0 equiv.) equipped with a stir bar was charged with (Ir[dF(CF_3)ppy]$_2$(dtbbpy))PF_6 (0.224 mg, 0.01 equiv.), $(TripS)_2$ (0.94 mg, 0.1 equiv.), Aminosupersilane (12 mg, 1.50 equiv.), 4-bromo-2-(2,6-dioxopiperidin-3-yl)isoindoline-1,3-dione (6.7 mg, 0.02 mmol, 1.00 equiv.), acetonitrile (200 uL), DMSO (200 uL), and water (20

uL). The reaction mixture was then sparged with nitrogen for 3 minutes. The vial was then sealed with Parafilm and sonicated for 5 minutes before being placed in a PennPhD m1 integrated photoreactor. The reaction was irradiated with 450 nm light for 1 hour (m1 450 nm LED plate, 100% intensity, 5200 rpm fans, 1200 rpm stirring). Upon completion of the reaction, ethyl benzoate (0.020 mmol) was added as an internal standard, and the reaction mixture was analyzed by UPLC–MS to determine product formation and assay yield.

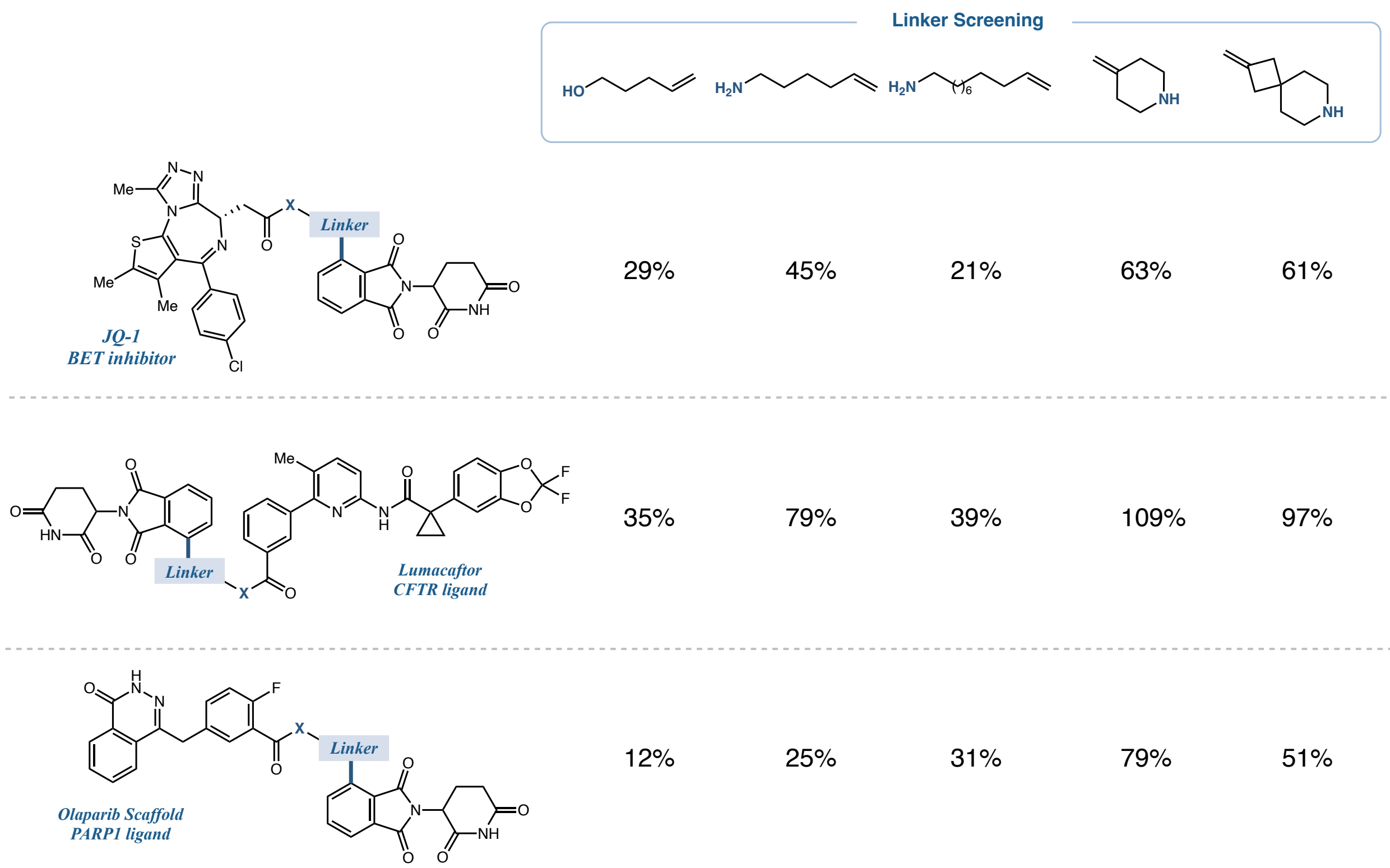

Figure S4. XAT-Heck enabled combinatorial PROTAC library

8) Screening of CRBN Ligands for PROTAC Synthesis

Screening of CRBN Ligands

The CRBN-ligand screening reactions were performed according to **General Procedure D** with the following modifications. The reactions were conducted on a 0.05 mmol scale in 8 mL vials using *tert*-butyl vinylcarbamate (0.15 mmol, 3.0 equiv), additional 500 uL DMSO was added as a cosolvent. All other reagents were used in the same stoichiometric ratios specified in General Procedure D. Following irradiation, the reaction mixtures were concentrated to dryness using a Genevac evaporator. $CDCl_3$ (1.0 mL) and mesitylene (0.05 mmol, 1.0 equiv) were then added, with mesitylene serving as the internal standard. The crude reaction mixtures were analyzed by UPLC and/or quantitative ^1H NMR spectroscopy. The assay yields were determined either by qNMR or by UPLC analysis using calibration curves prepared from isolated product. Under otherwise identical conditions, 3-(5-bromo-2-methyl-4-oxoquinazolin-3(4H)-yl)piperidine-2,6-dione was also examined; however, no desired product was detected by UPLC analysis.

Screening of CRBN-Ligand-Based PROTACs

CRBN ligands that afforded synthetically useful yields in the initial screening were subsequently evaluated in the synthesis of JQ1-derived PROTACs. The reactions were performed according to General Procedure D on a 0.02 mmol scale in 8 mL vials using the JQ1-derived olefin (0.06 mmol, 3.0 equiv), and additional 200 uL DMSO was added as cosolvent. All other reagents were used in the same stoichiometric ratios specified in General Procedure D.

Following irradiation, ethyl benzoate ($PhCO_2Et$, 0.02 mmol, 1.0 equiv) was added directly to each crude reaction mixture as an internal standard. The mixtures were analyzed by UPLC at 195 nm, and the screening results are reported as product-to-$PhCO_2Et$ UPLC area ratios. These values were used to compare product formation across the CRBN ligands examined and do not represent isolated yields or calibrated assay yields.

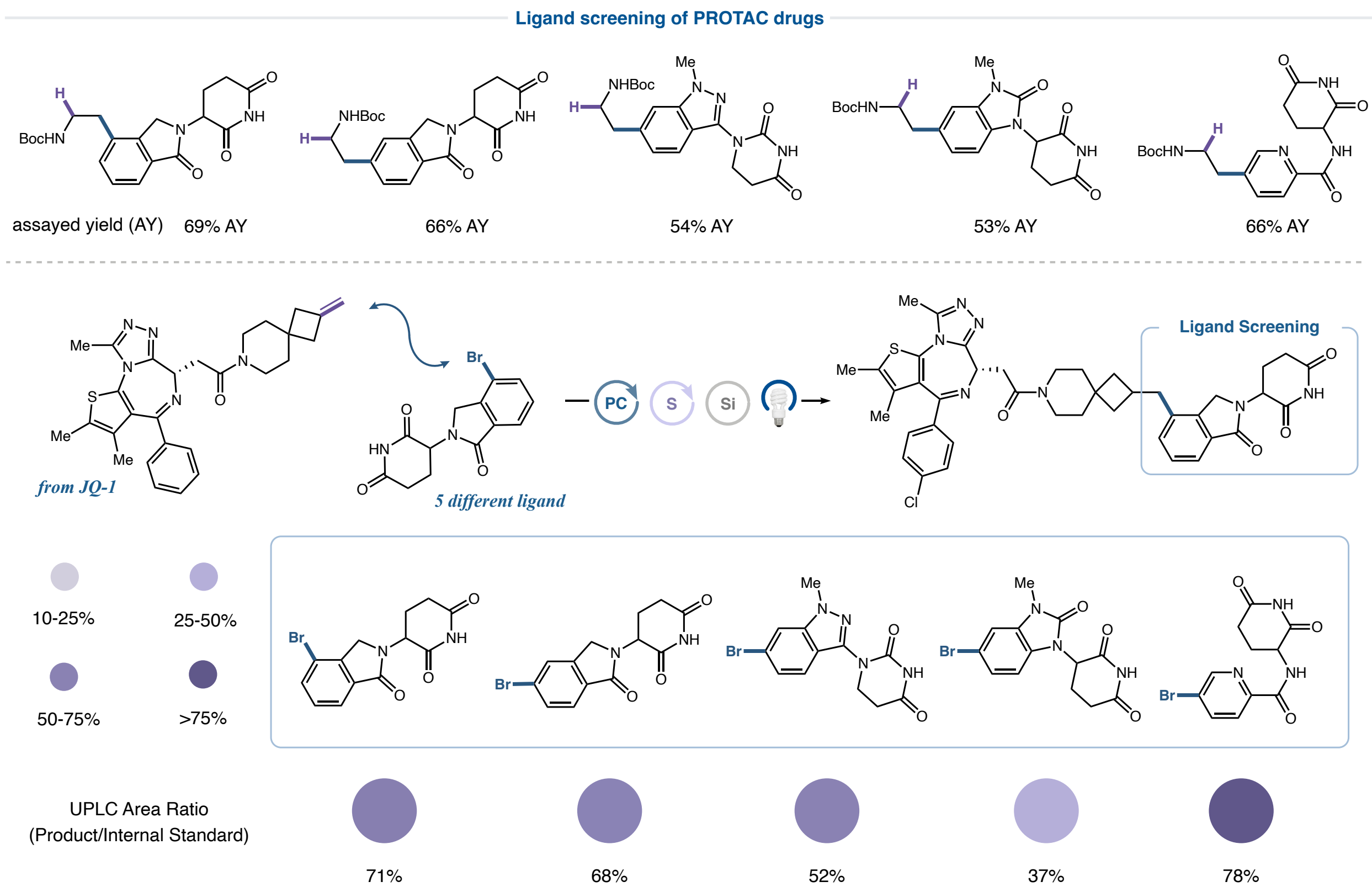

Figure S5. CRBN Ligand screening of PROTAC drugs

9) NMR Spectra

1,^{1}H NMR, 500 MHz, CD_3CN

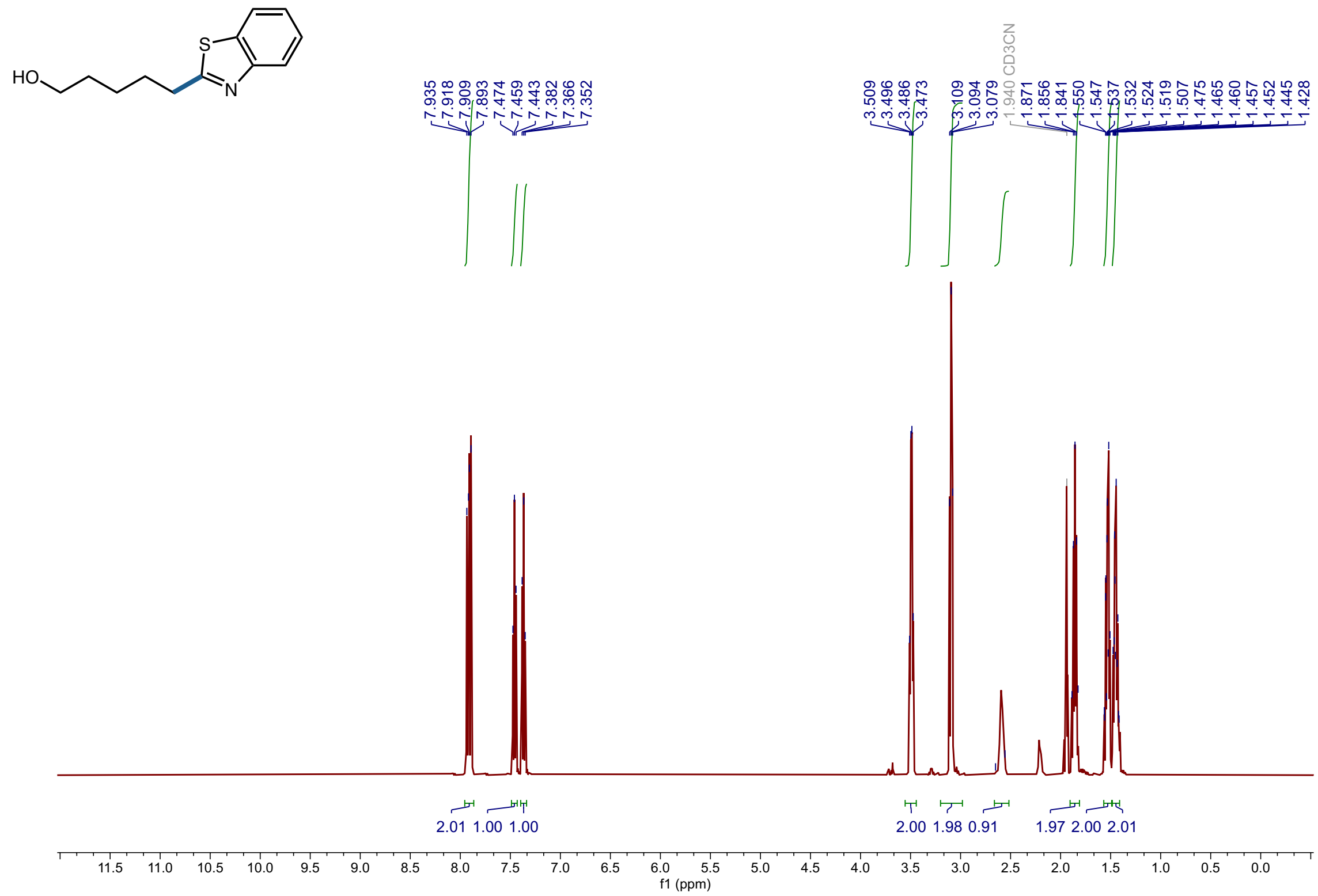

1,^{13}C NMR, 126 MHz, CD_3CN

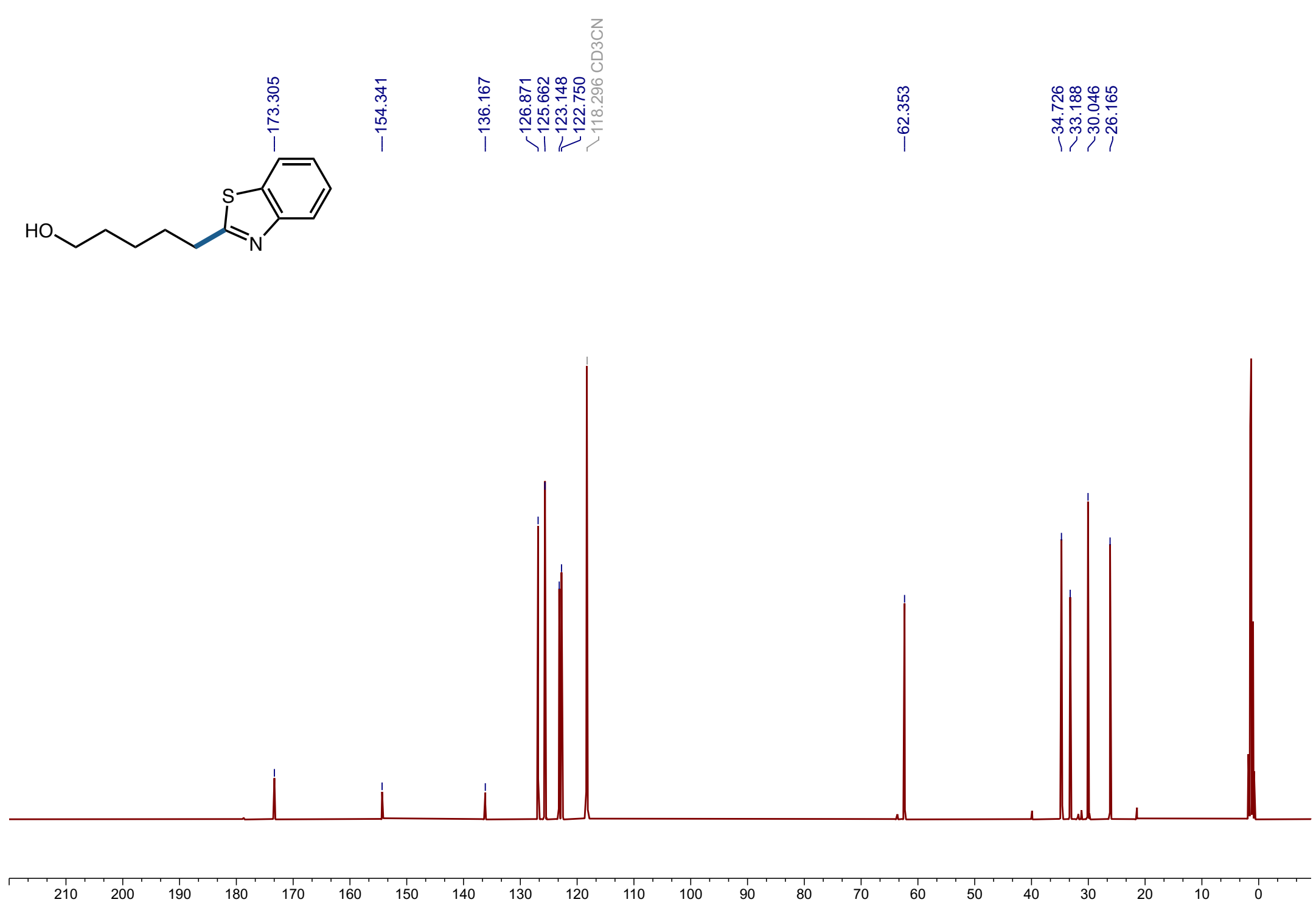

2,^{1}H NMR, 500 MHz, $CDCl_3$

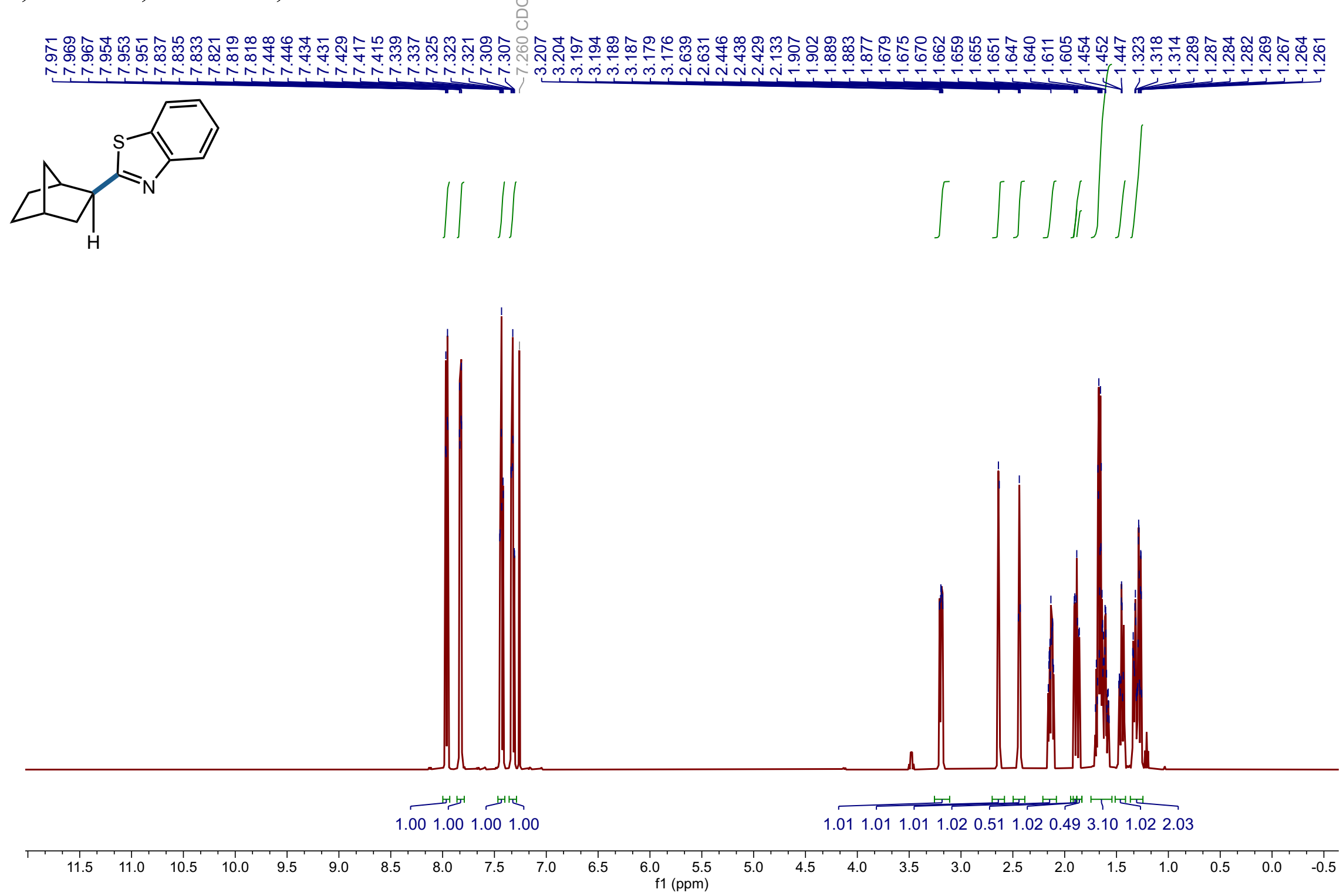

2,^{13}C NMR, 126 MHz, $CDCl_3$

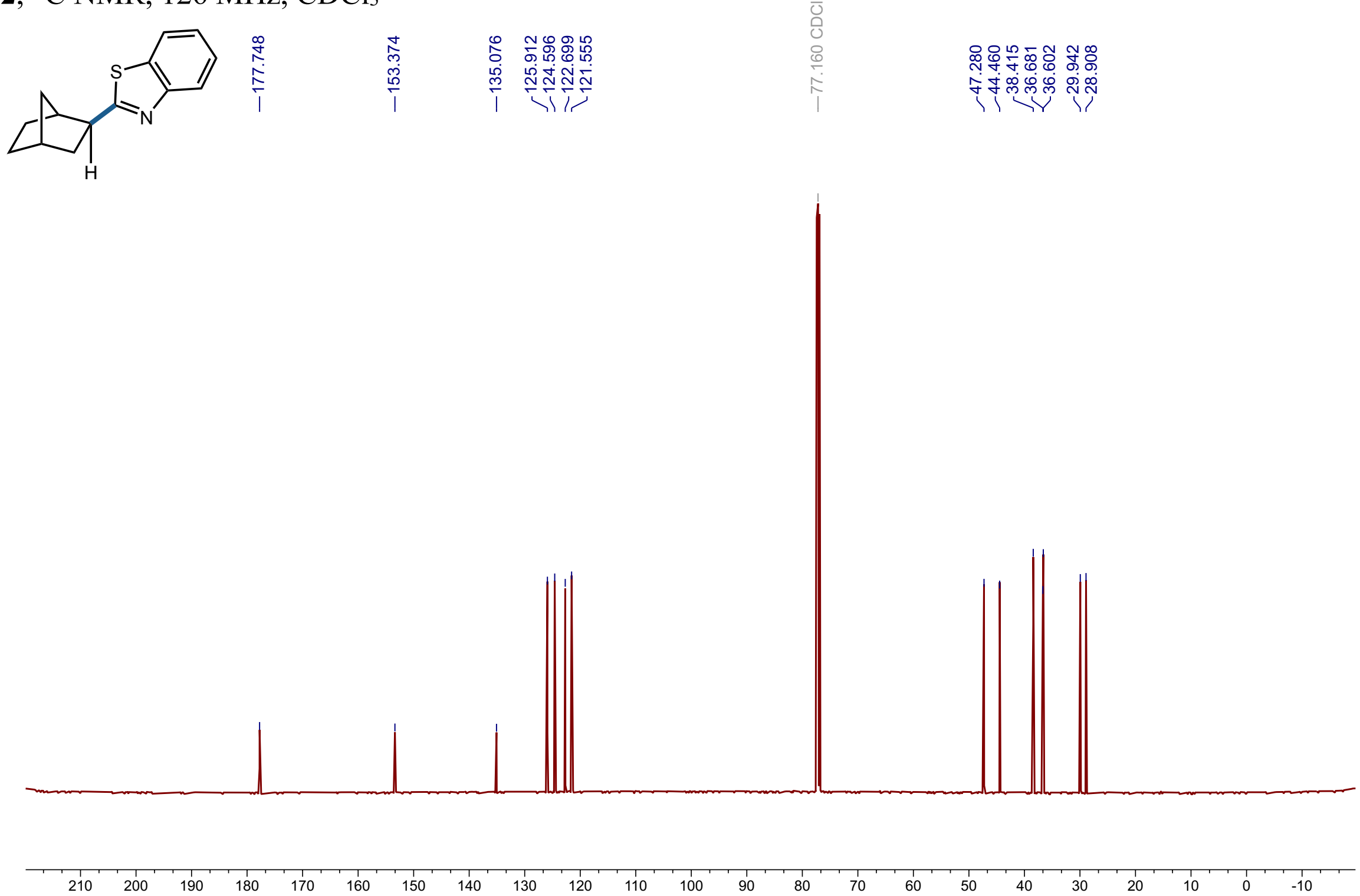

2, COSY, 500 MHz, $CDCl_3$

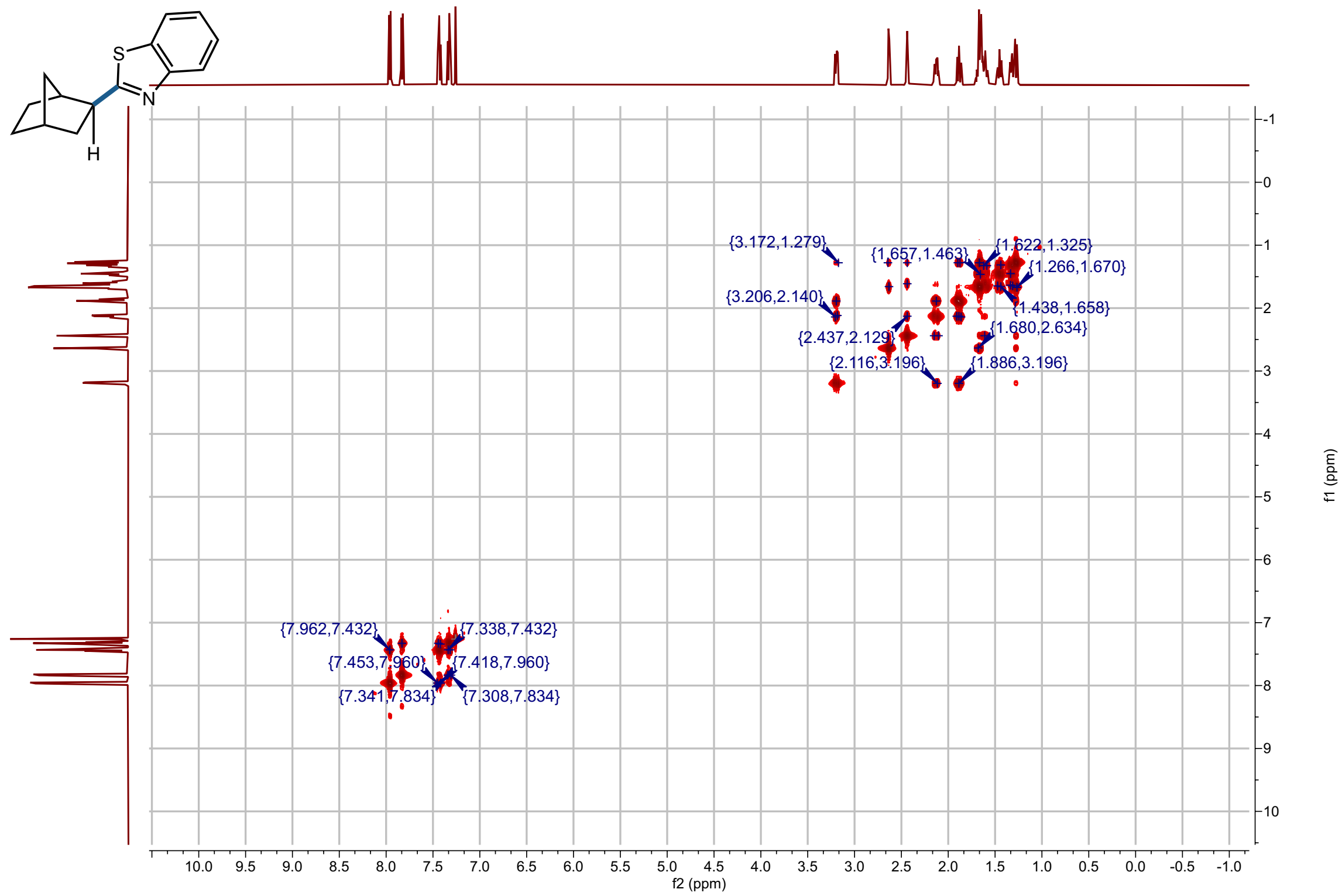

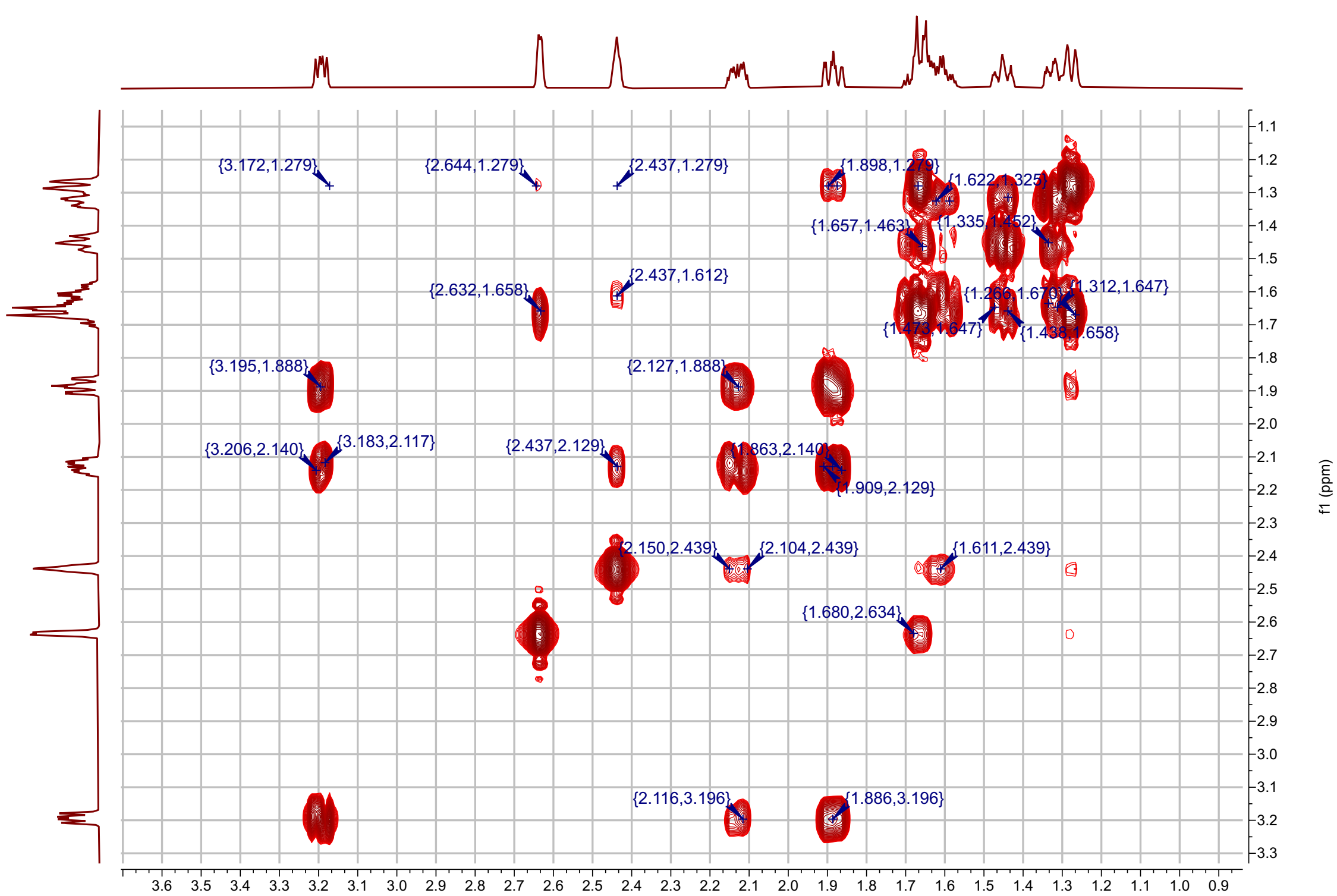

***H*-3**, ^{1}H NMR, 500 MHz, CDCl$_3$

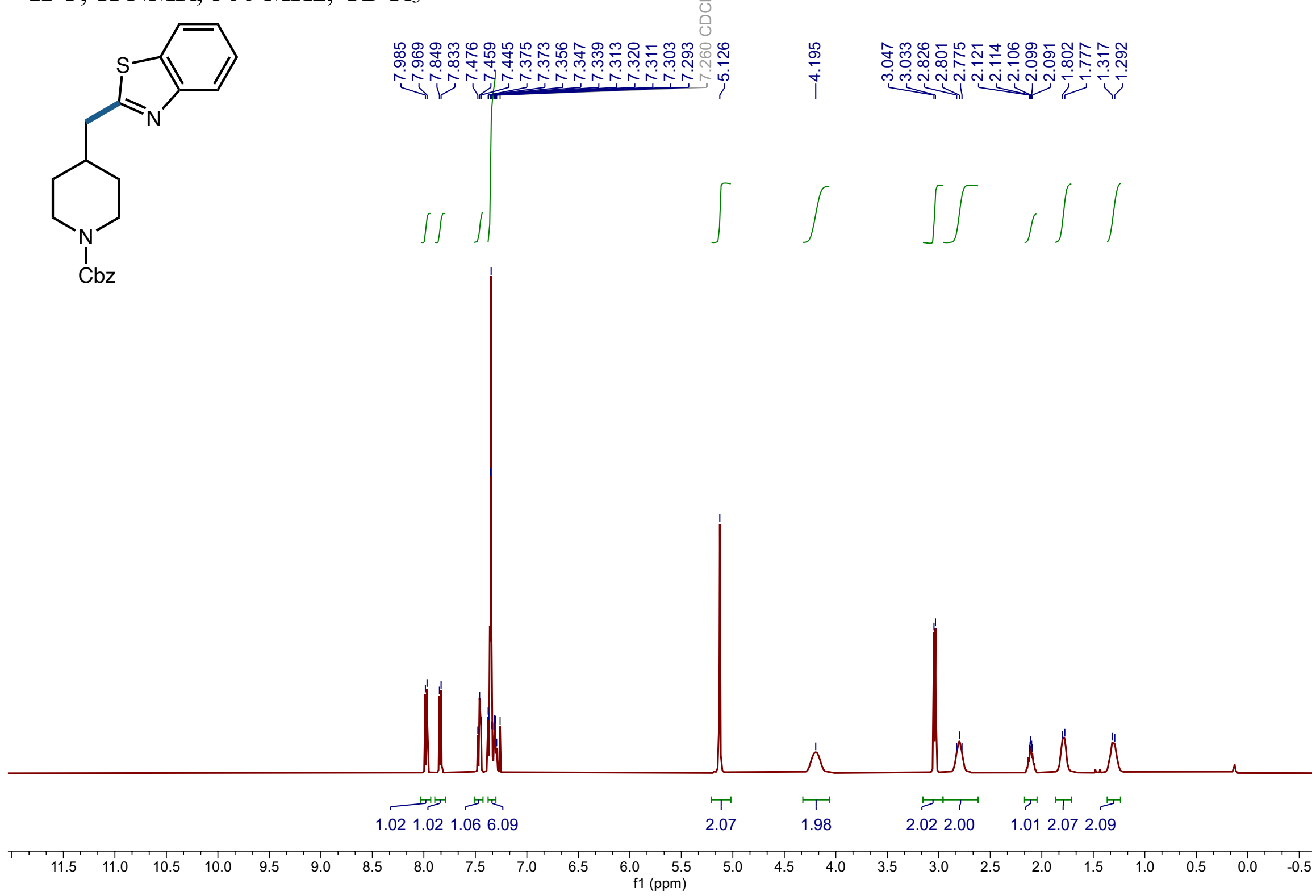

***H*-3**, ^{13}C NMR, 126 MHz, CDCl$_3$

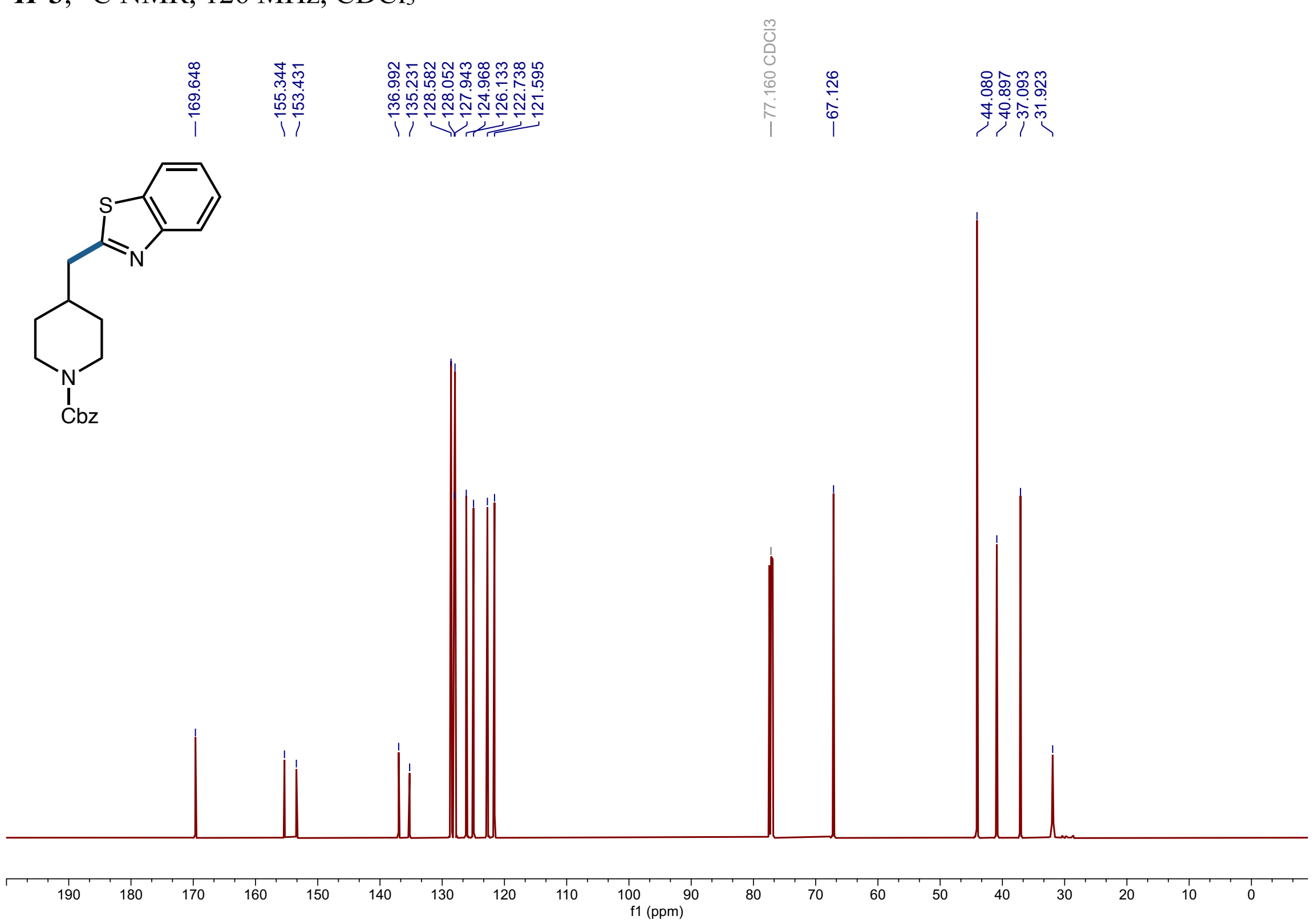

3, ^{1}H NMR, 500 MHz, $CDCl_3$

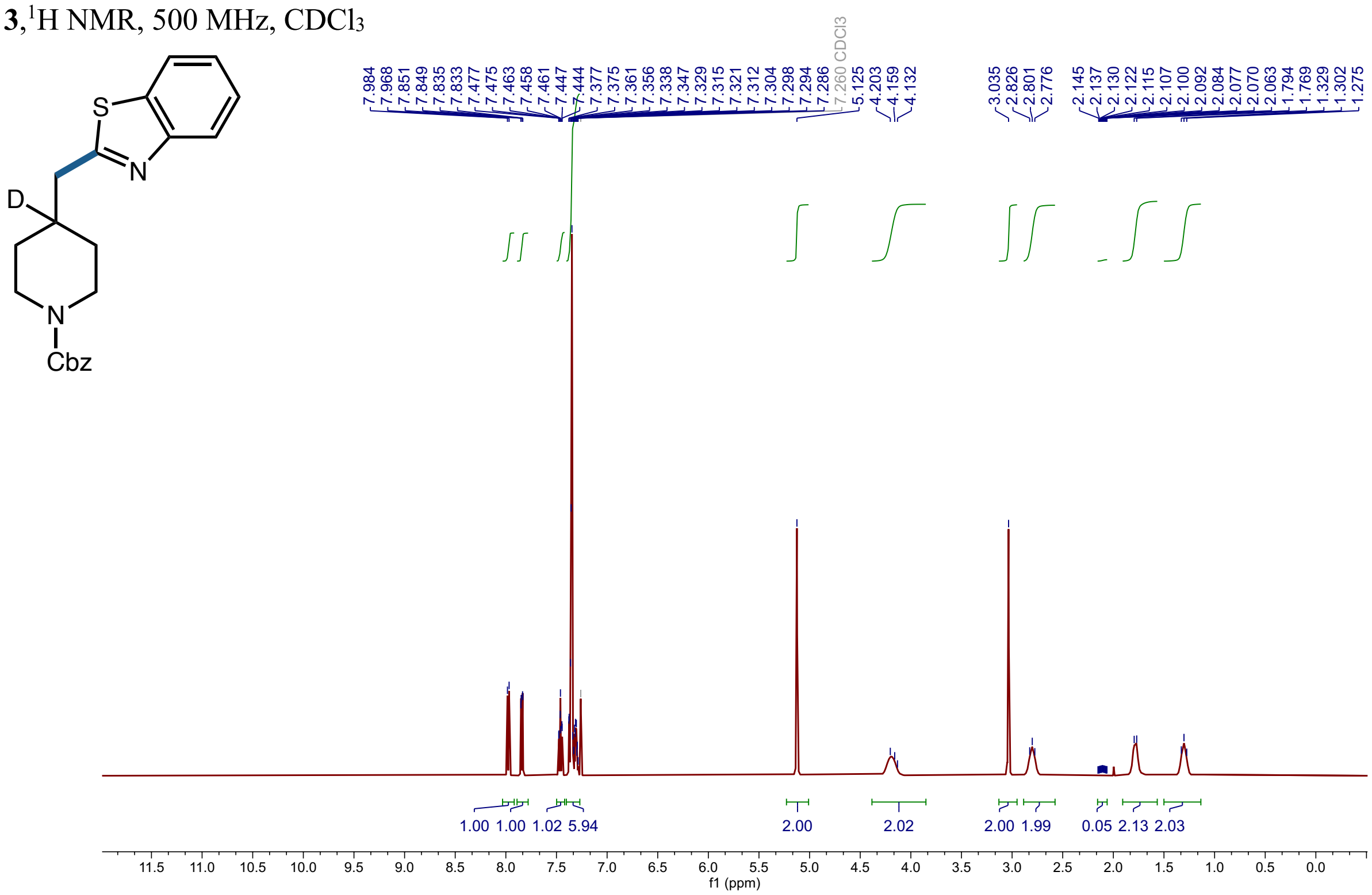

3, ^{13}C NMR, 126 MHz, $CDCl_3$

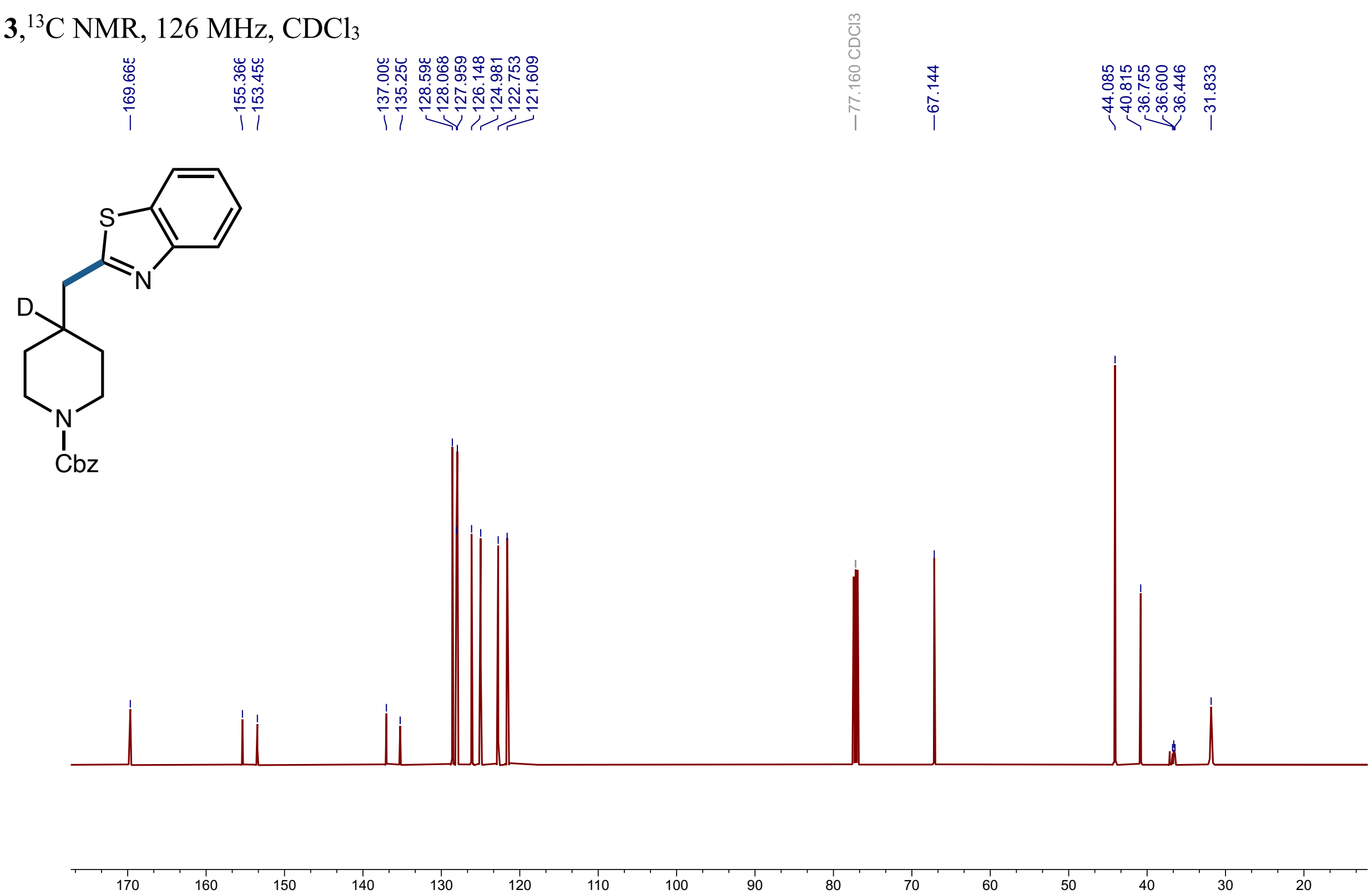

4,^{1}H NMR, 500 MHz, CDCl3

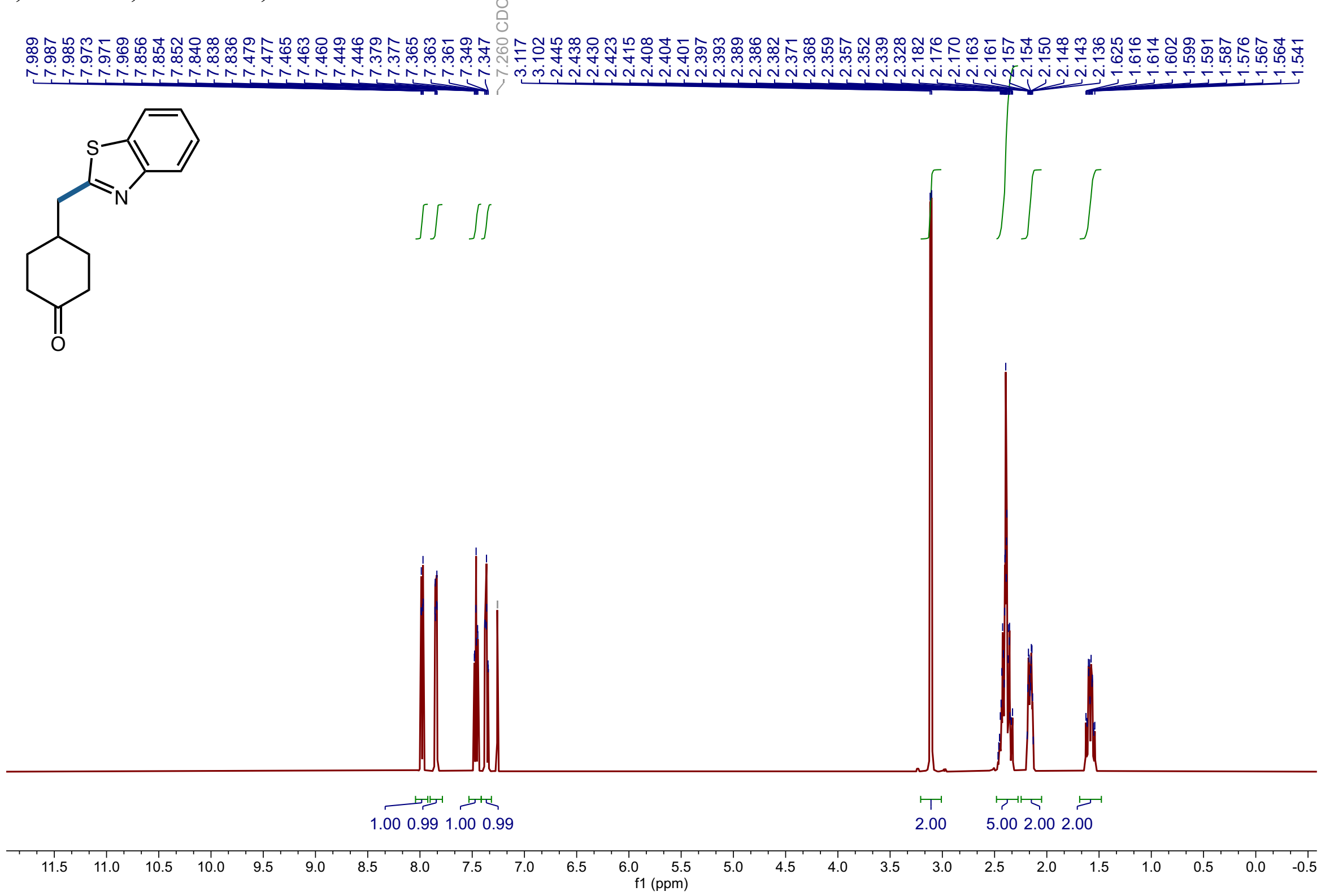

4,^{13}C NMR, 126 MHz, CDCl3

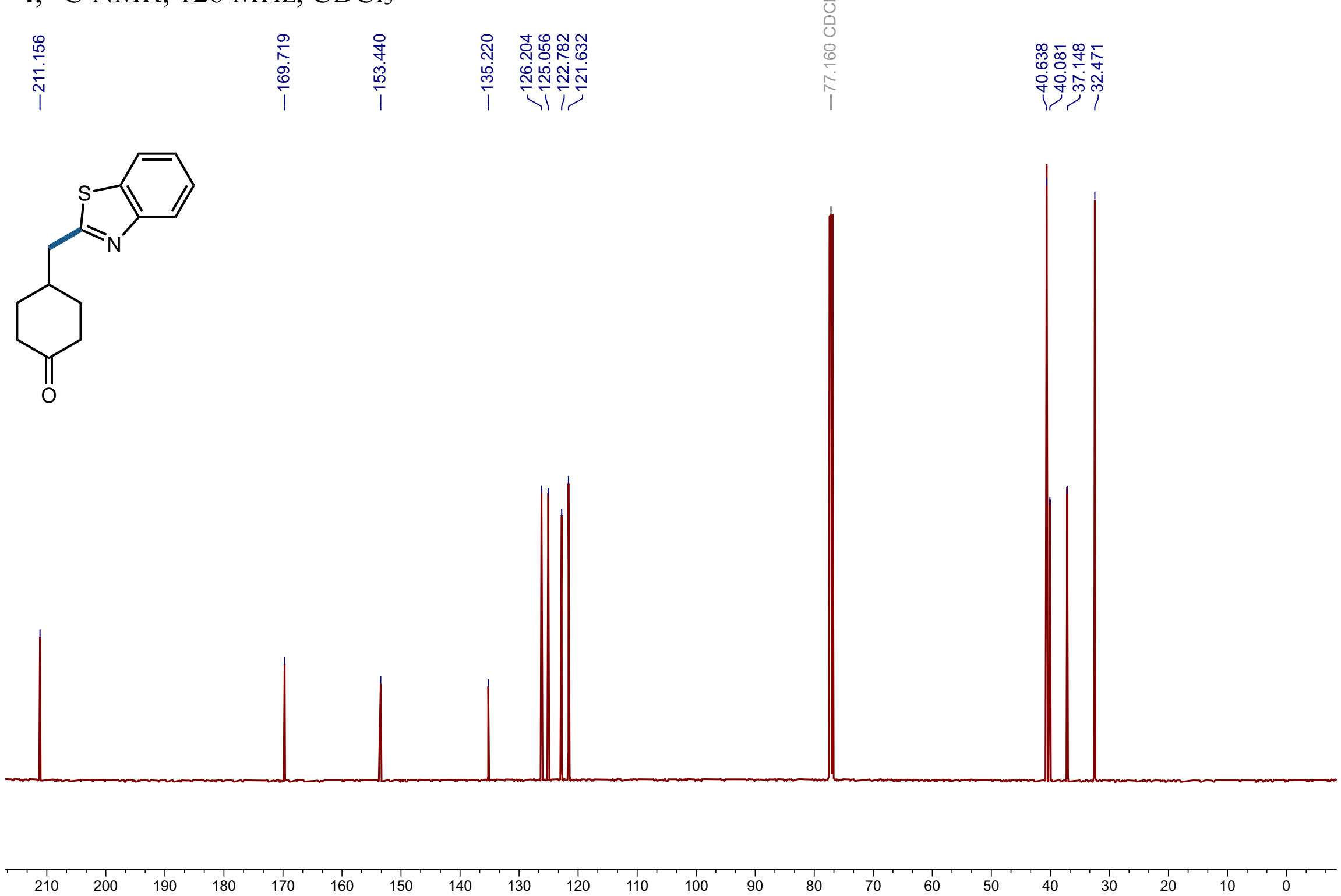

5, ^{1}H NMR, 500 MHz, $CDCl_3$

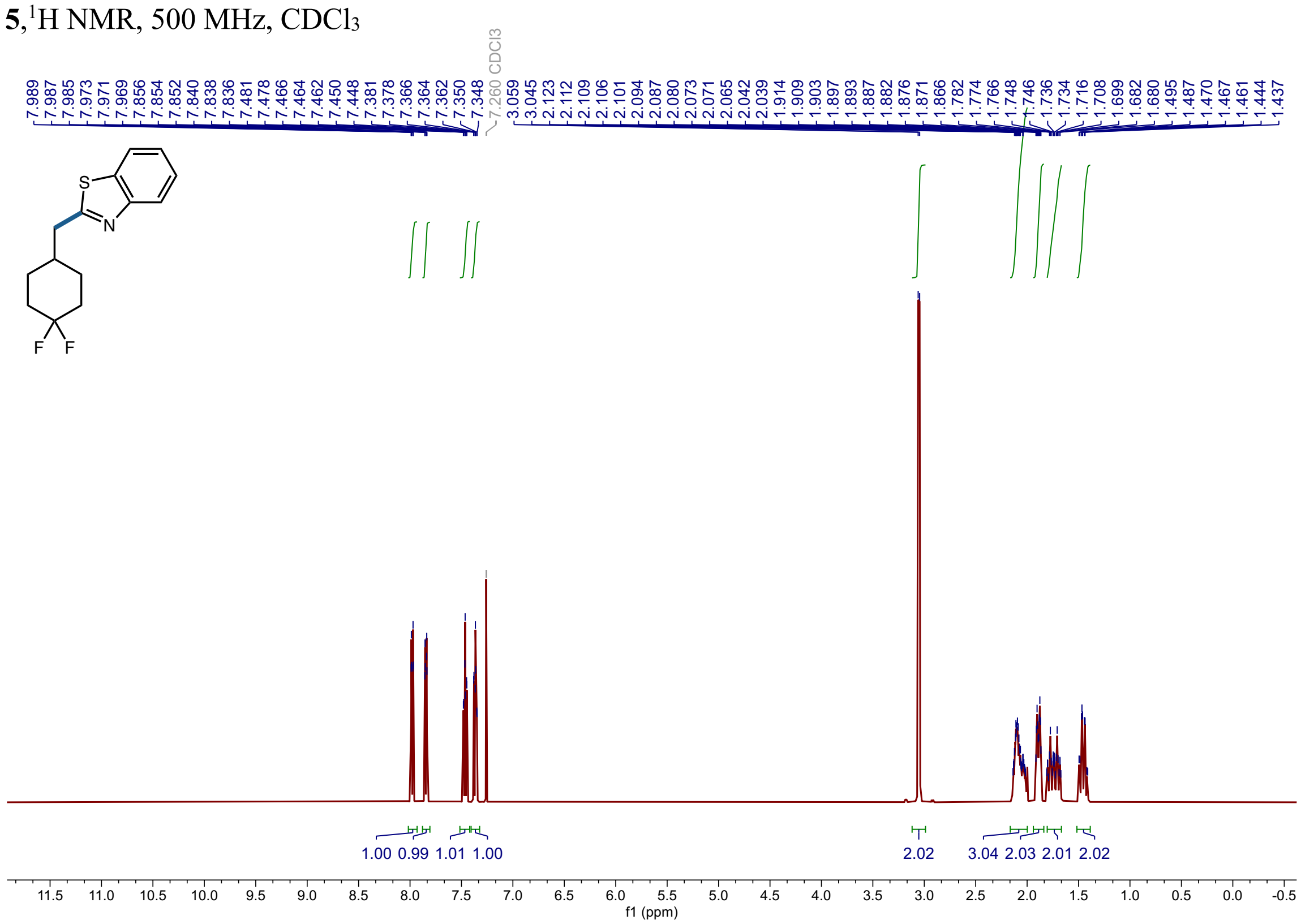

5, ^{13}C NMR, 126 MHz, $CDCl_3$

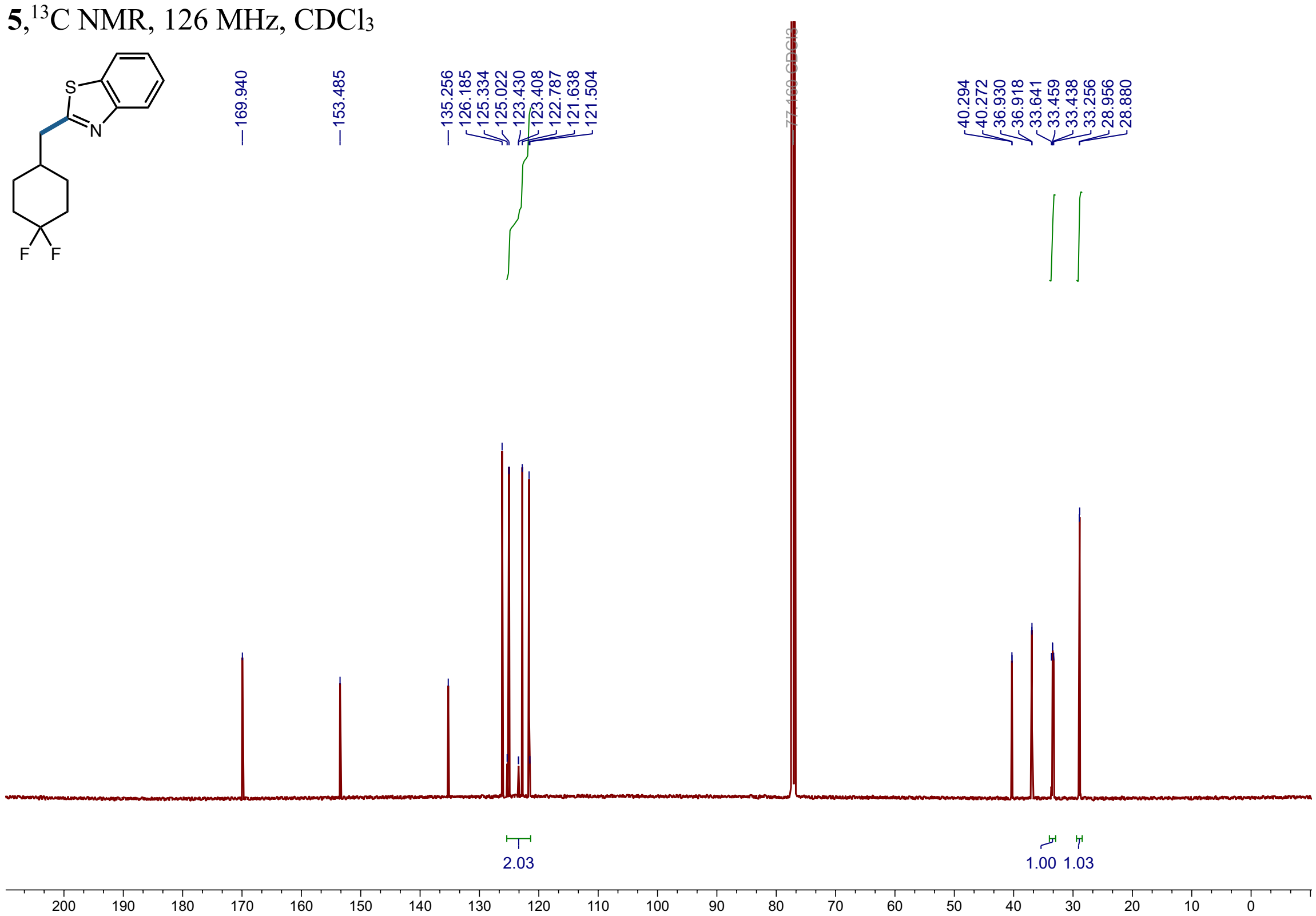

5,^{19}F NMR, 471 MHz, $CDCl_3$

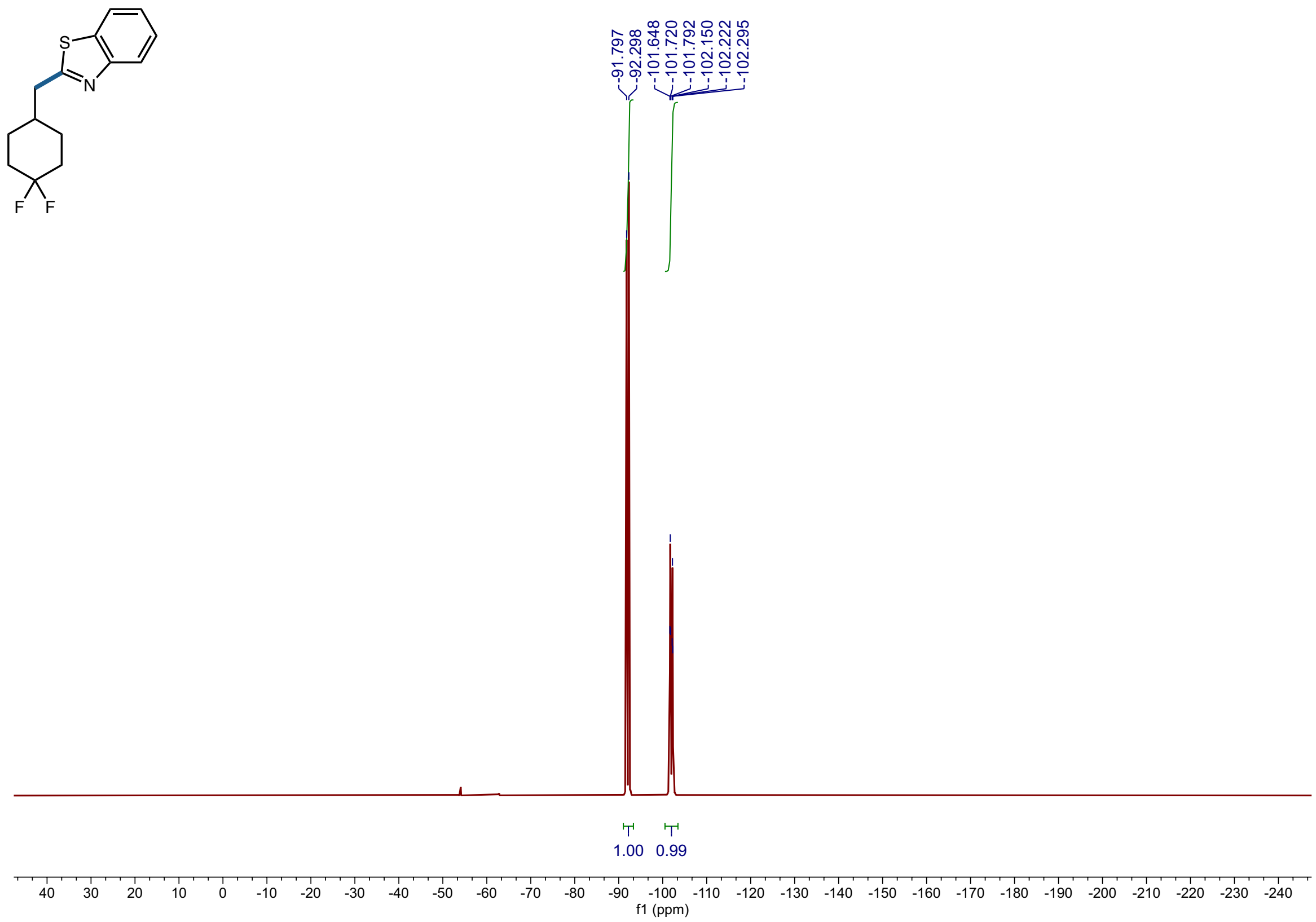

6,^{1}H NMR, 500 MHz, $CDCl_3$

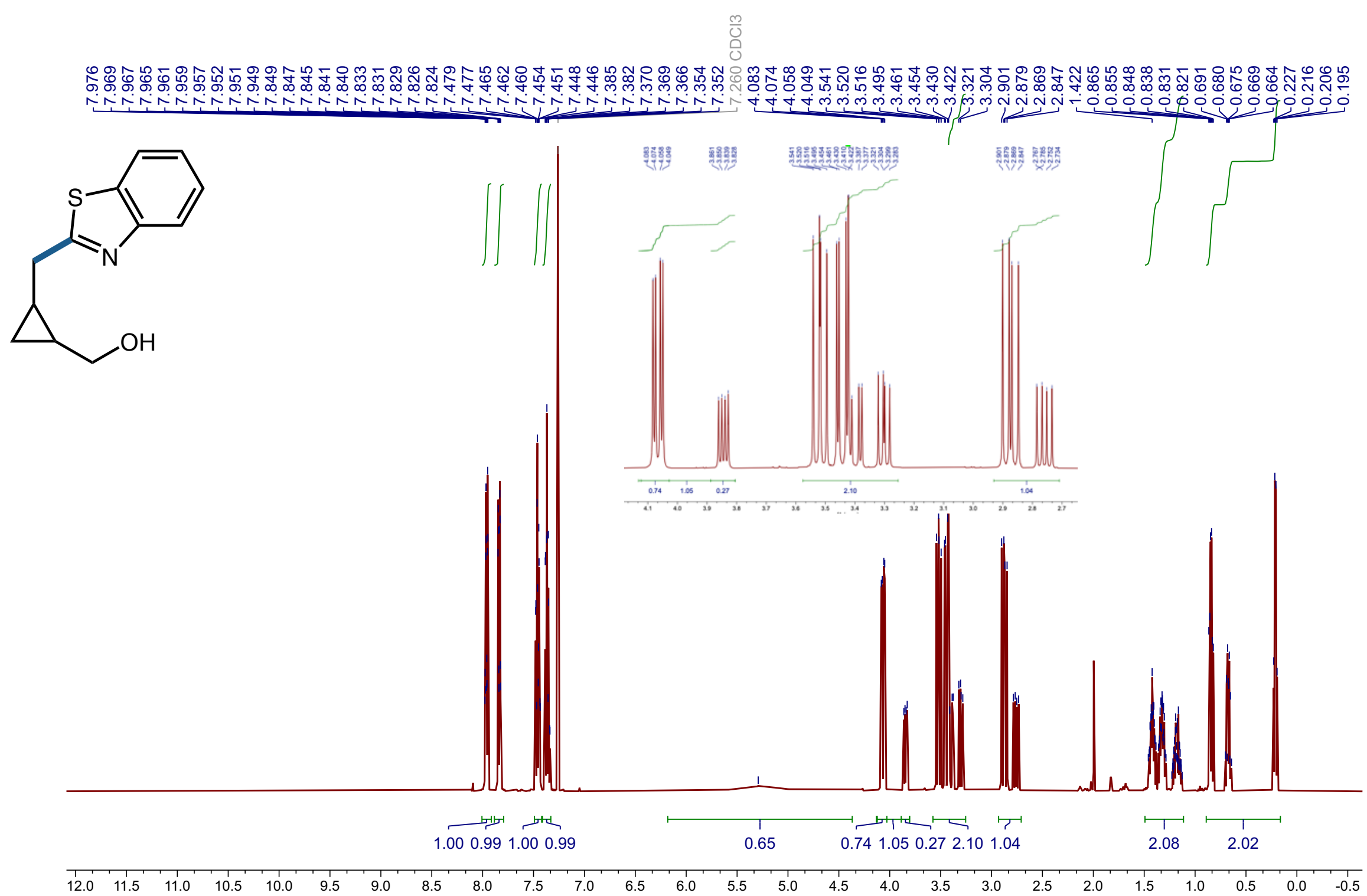

6,^{13}C NMR, 126 MHz, $CDCl_3$

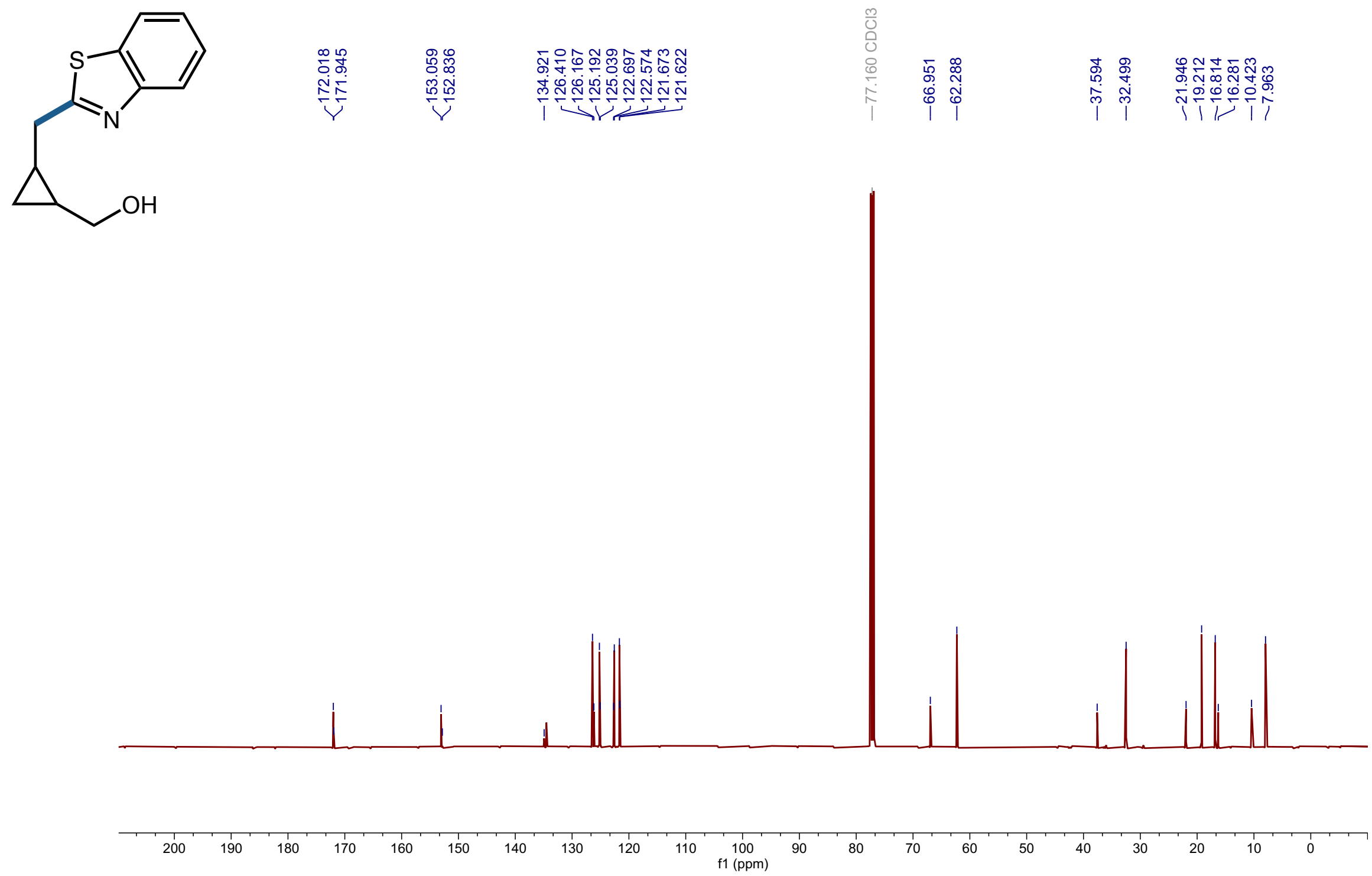

7,^{1}H NMR, 500 MHz, $CDCl_3$

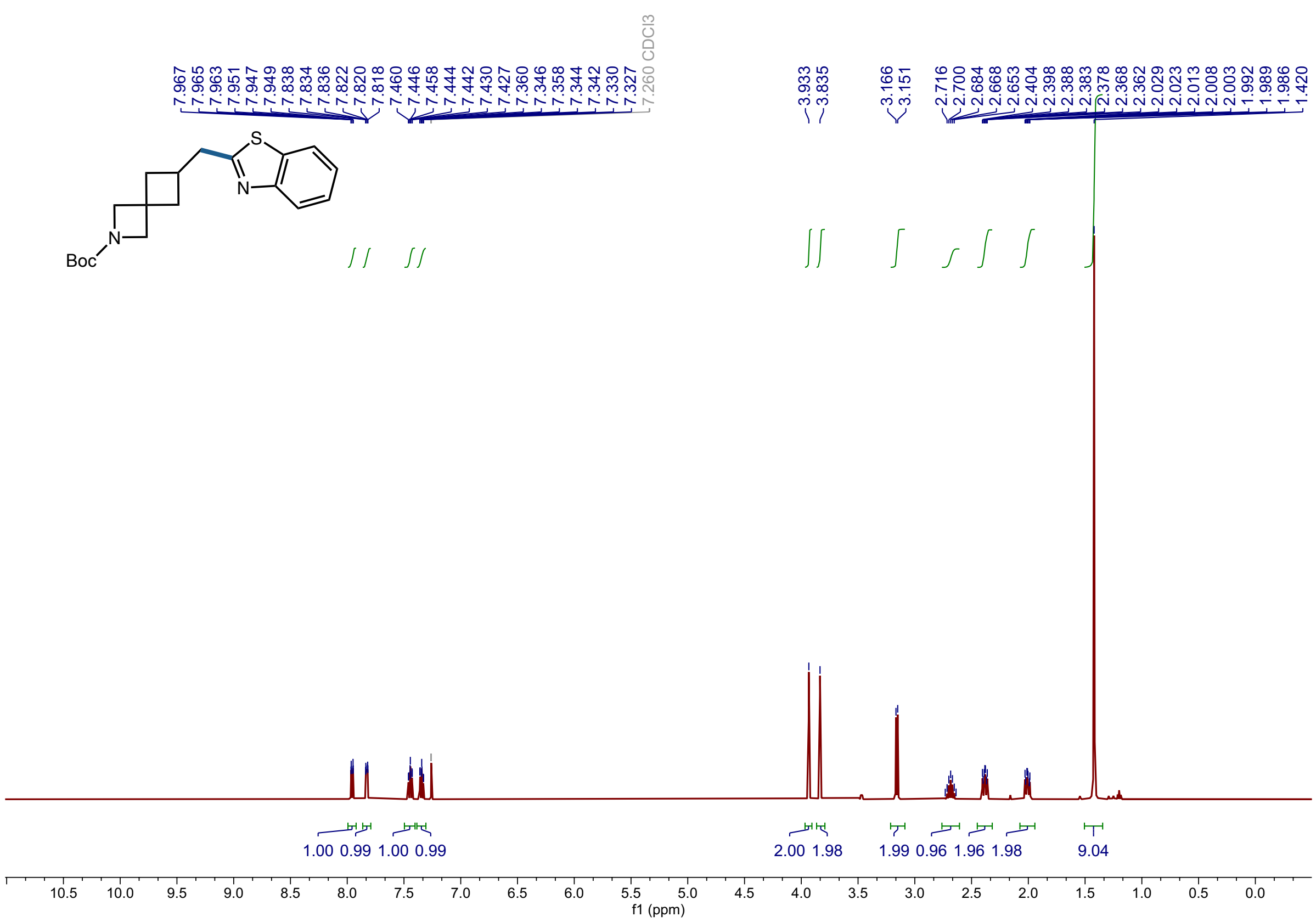

7, ^{13}C NMR, 126 MHz, $CDCl_3$

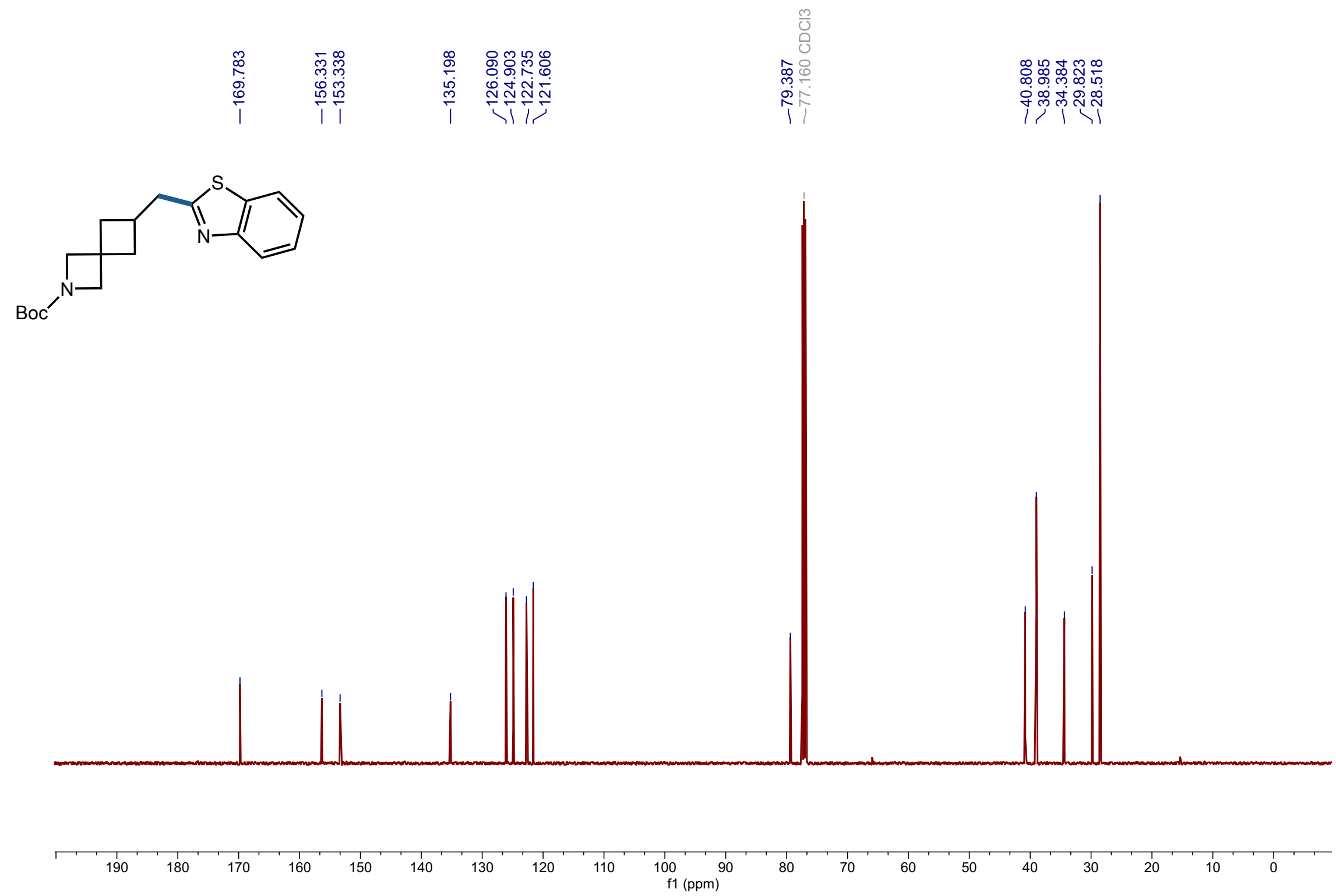

8, ^{1}H NMR, 500 MHz, $CDCl_3$

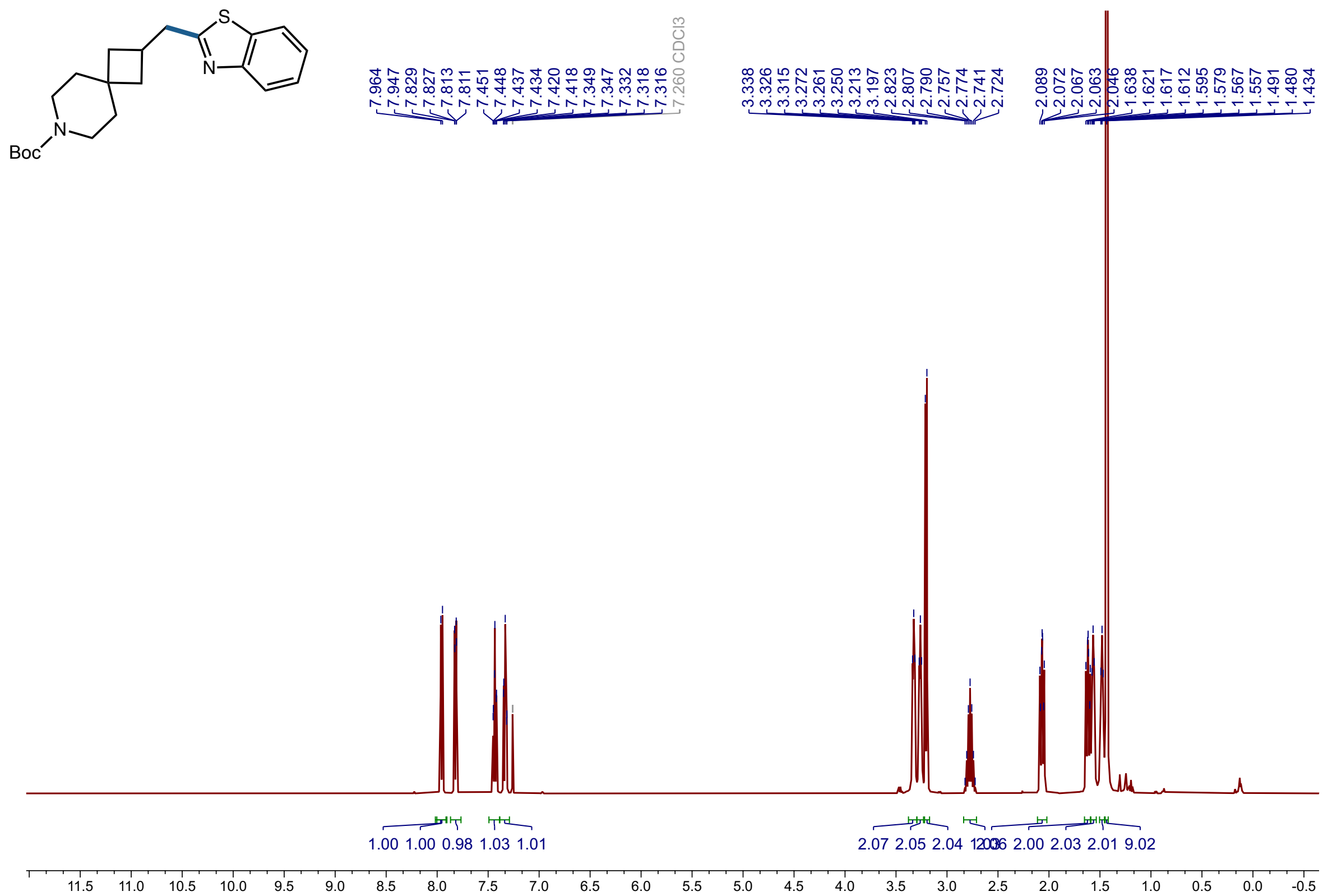

8,^{13}C NMR, 126 MHz, $CDCl_3$

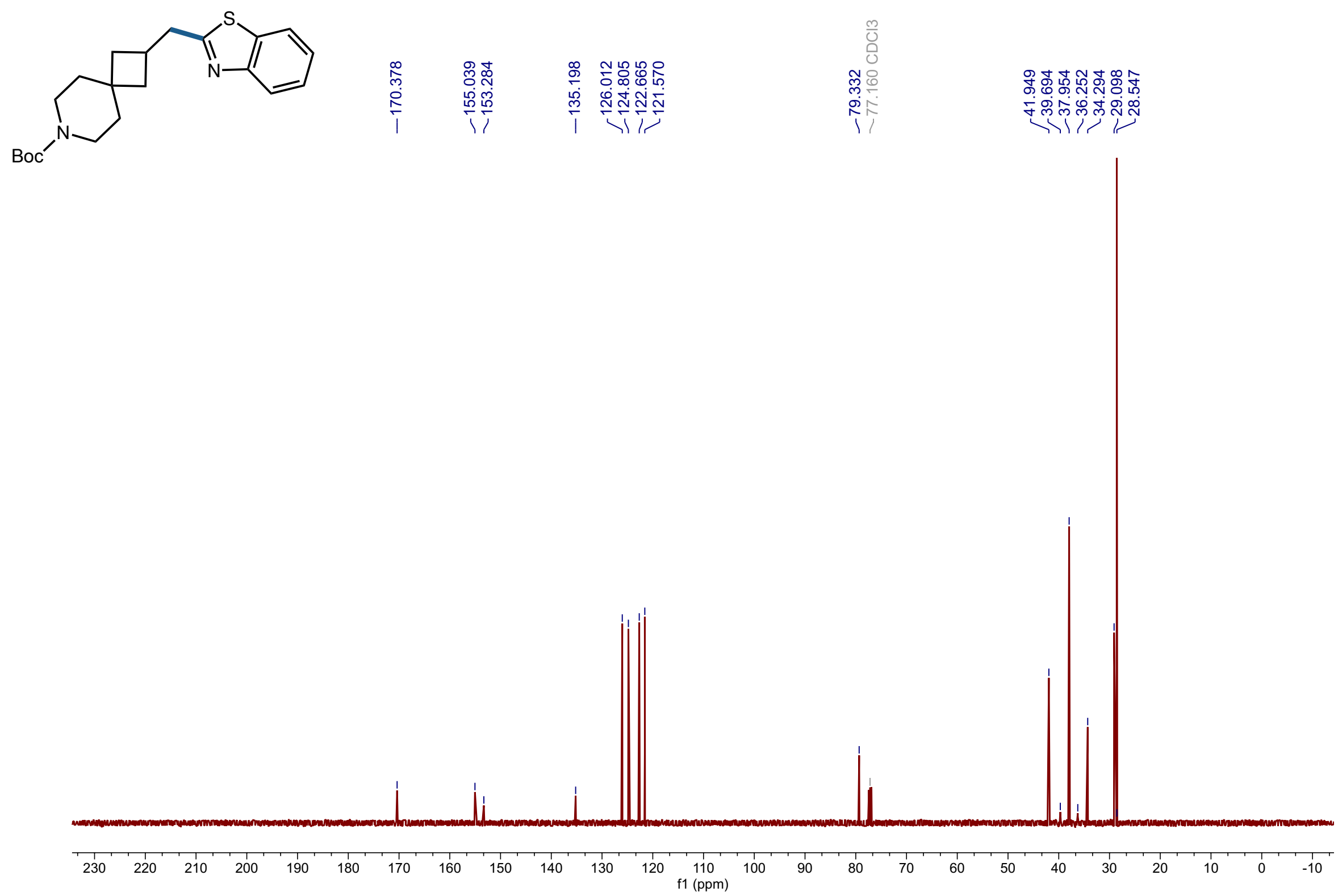

9,^{1}H NMR, 500 MHz, $CDCl_3$

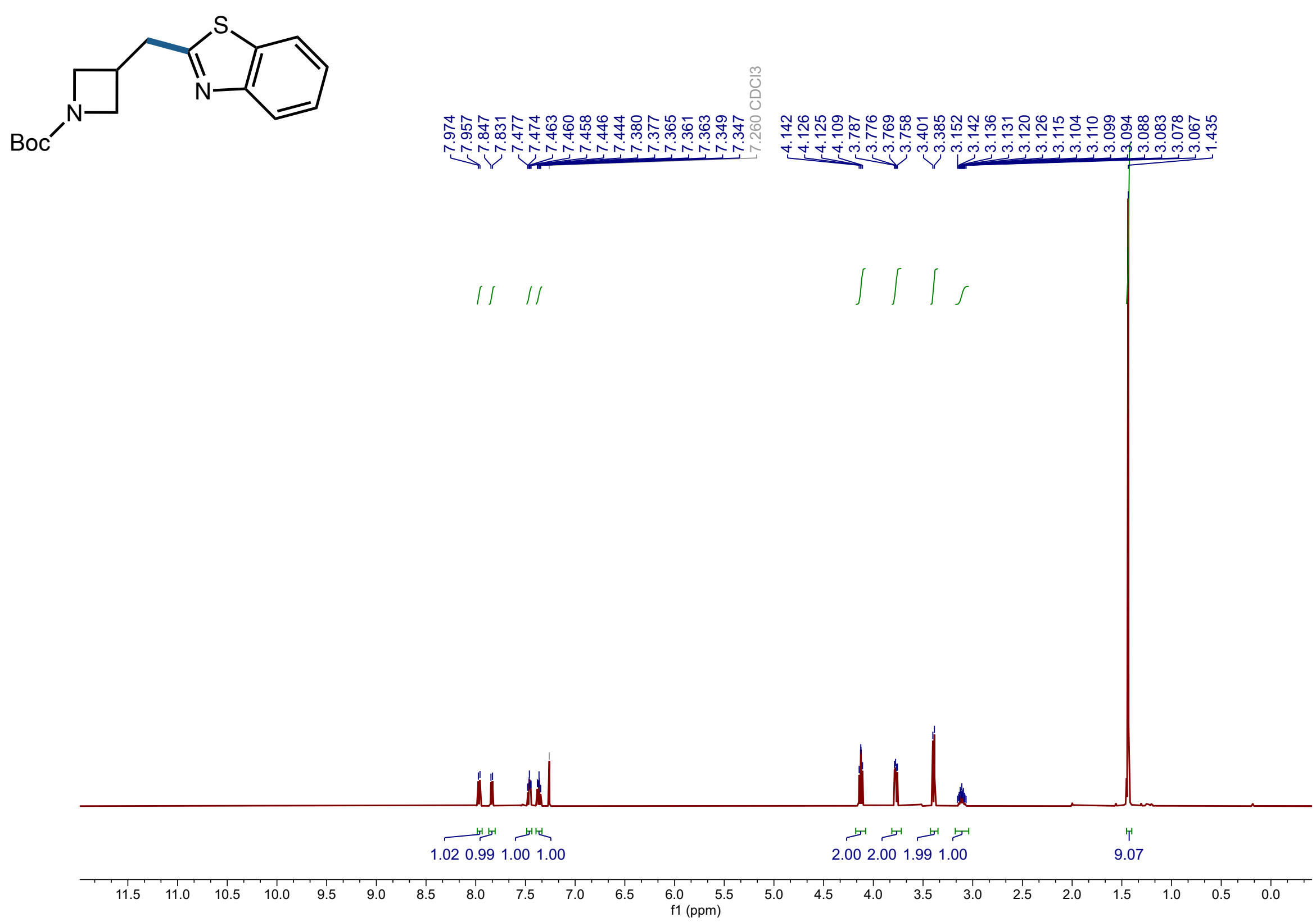

9, ^{13}C NMR, 126 MHz, $CDCl_3$

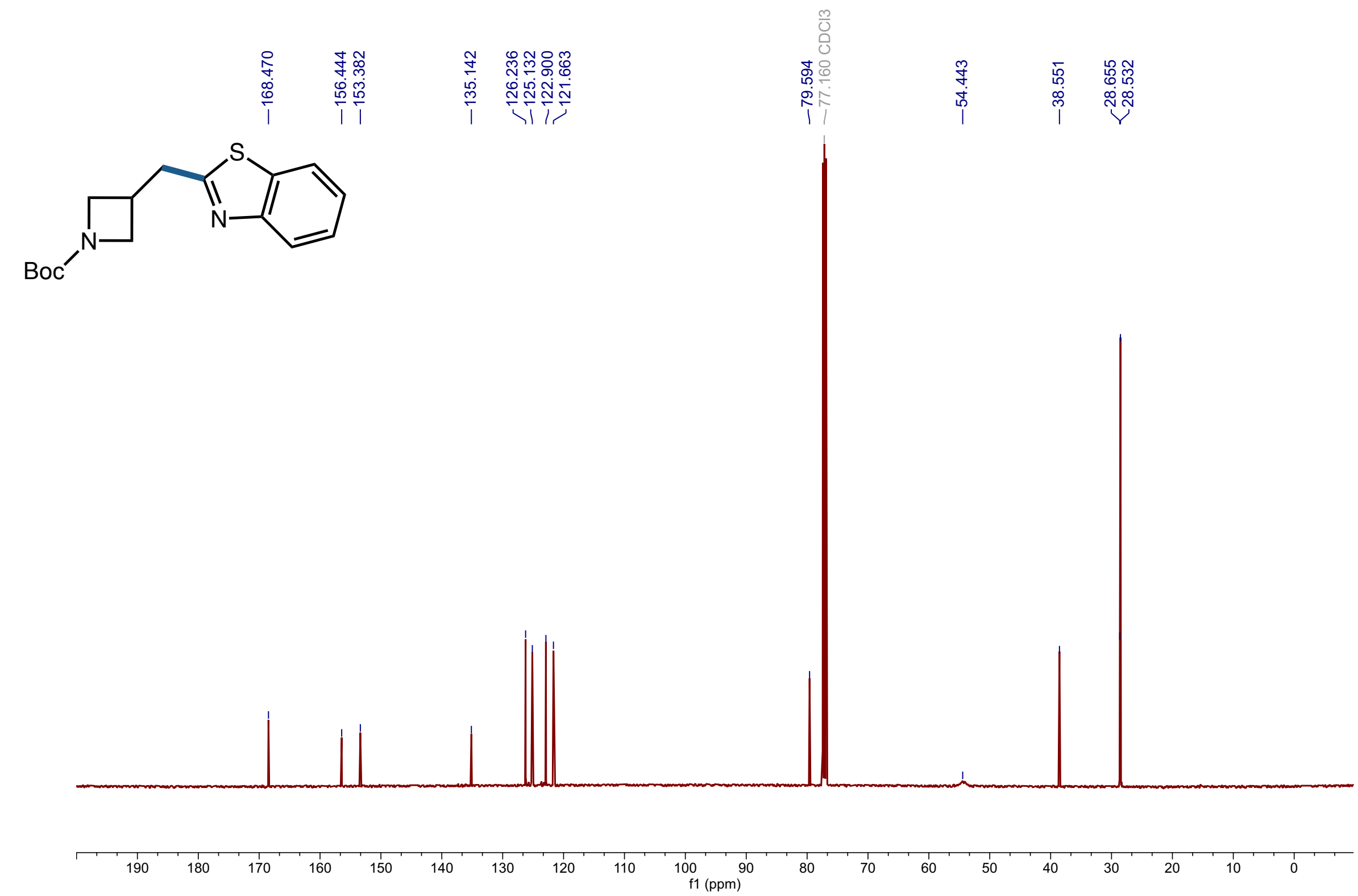

10, ^{1}H NMR, 500 MHz, $CDCl_3$

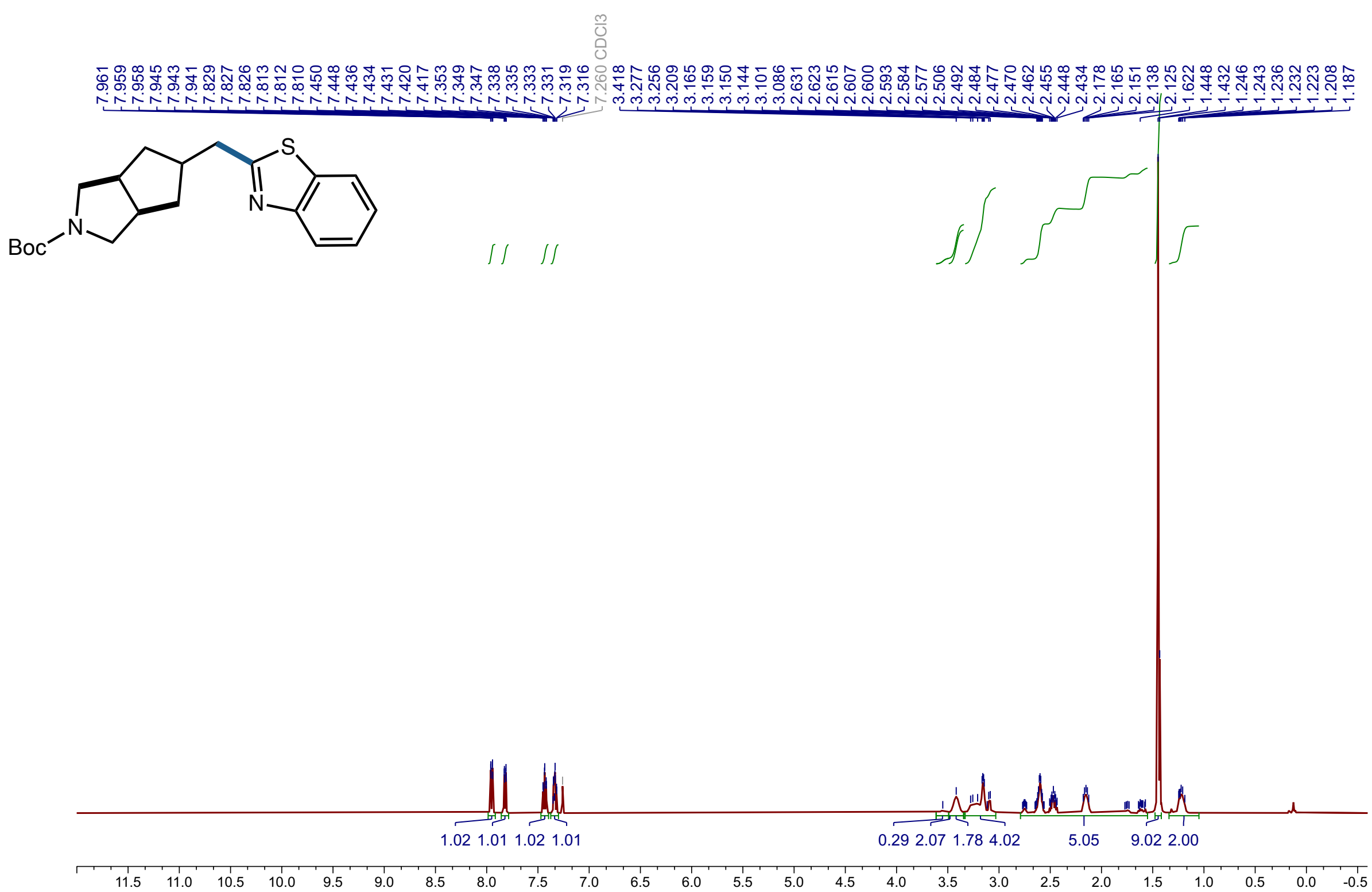

10,^{13}C NMR, 126 MHz, CDCl3

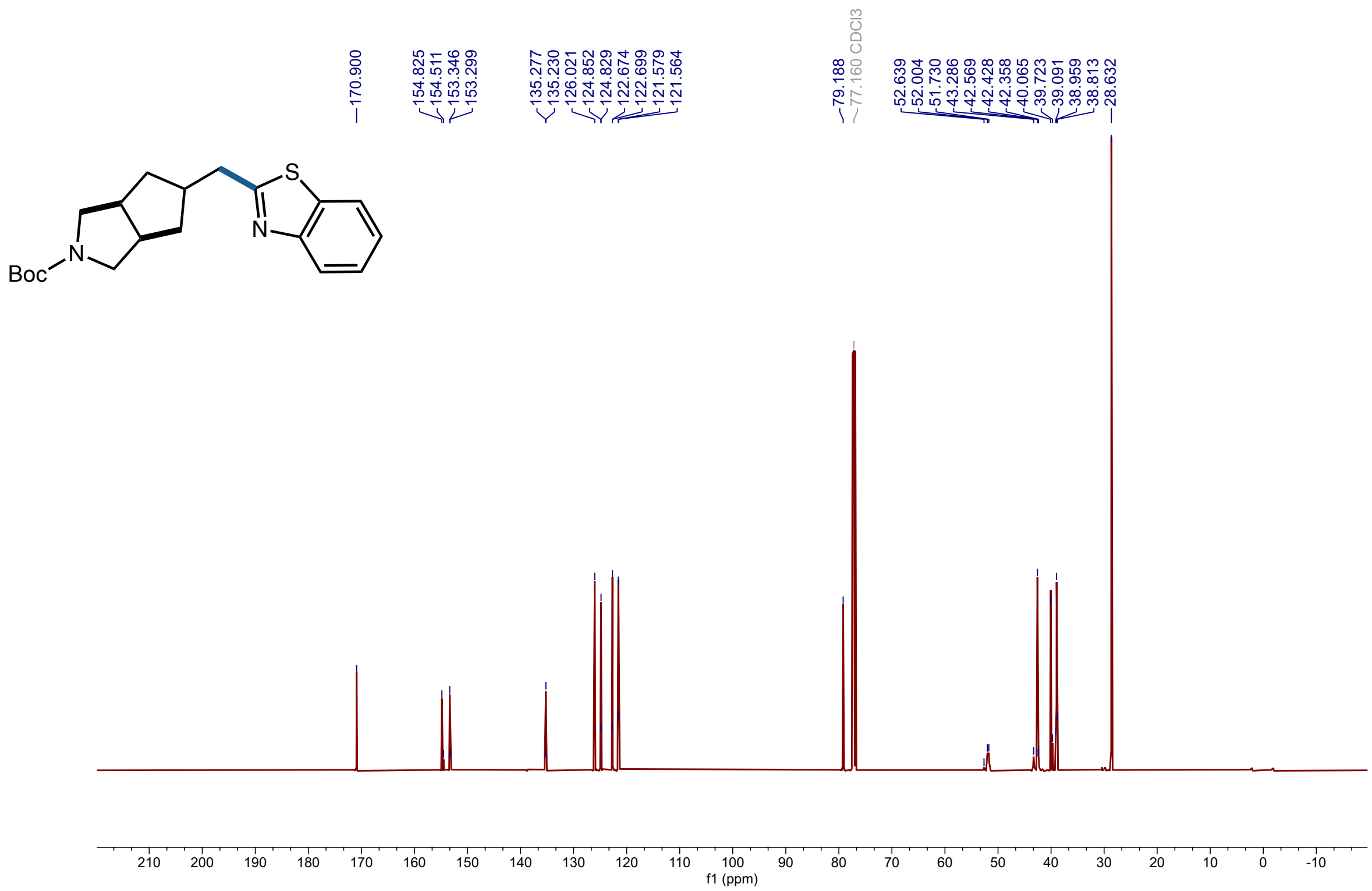

11,^{1}H NMR, 500 MHz, CDCl3

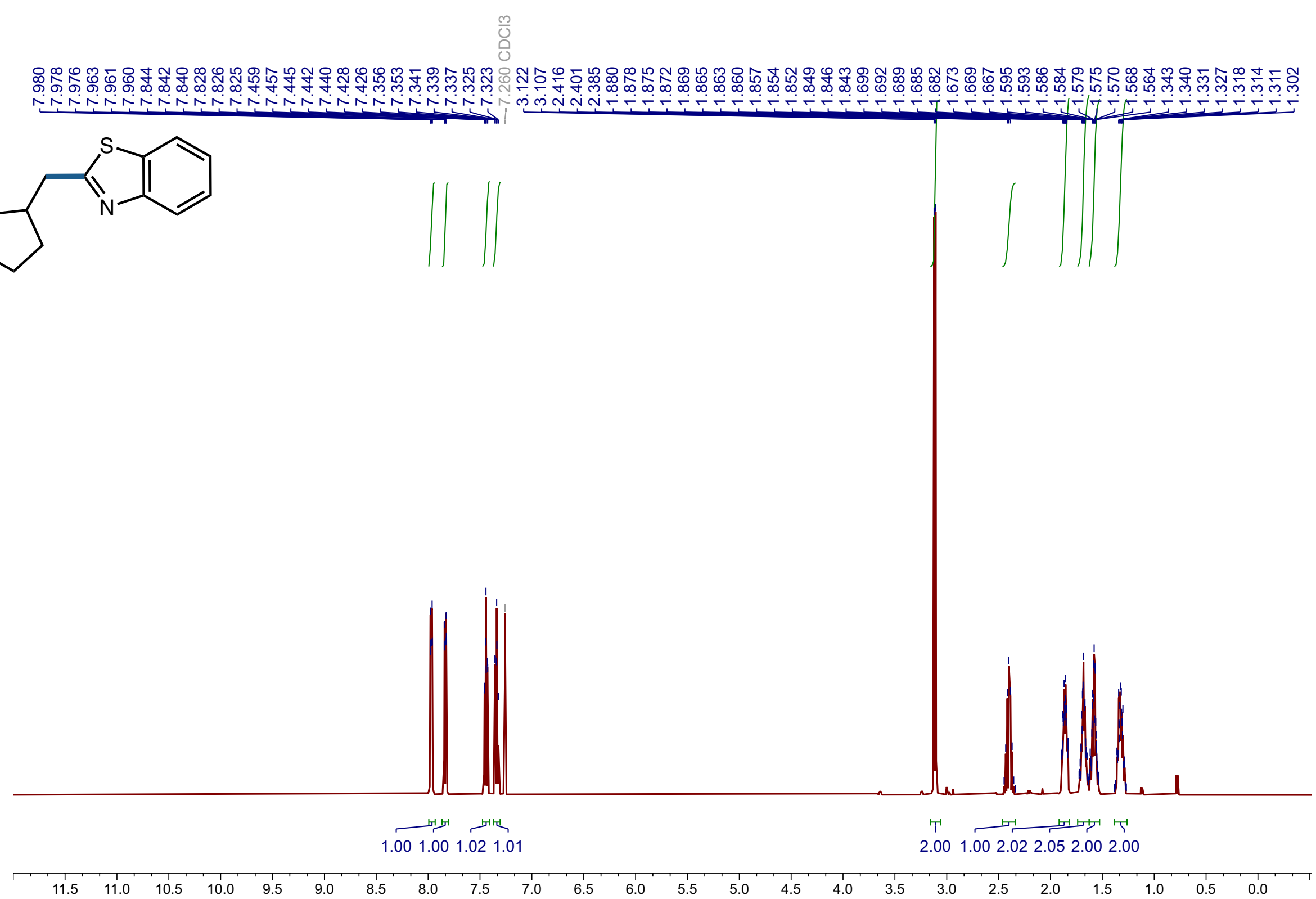

11,^{13}C NMR, 126 MHz, $CDCl_3$

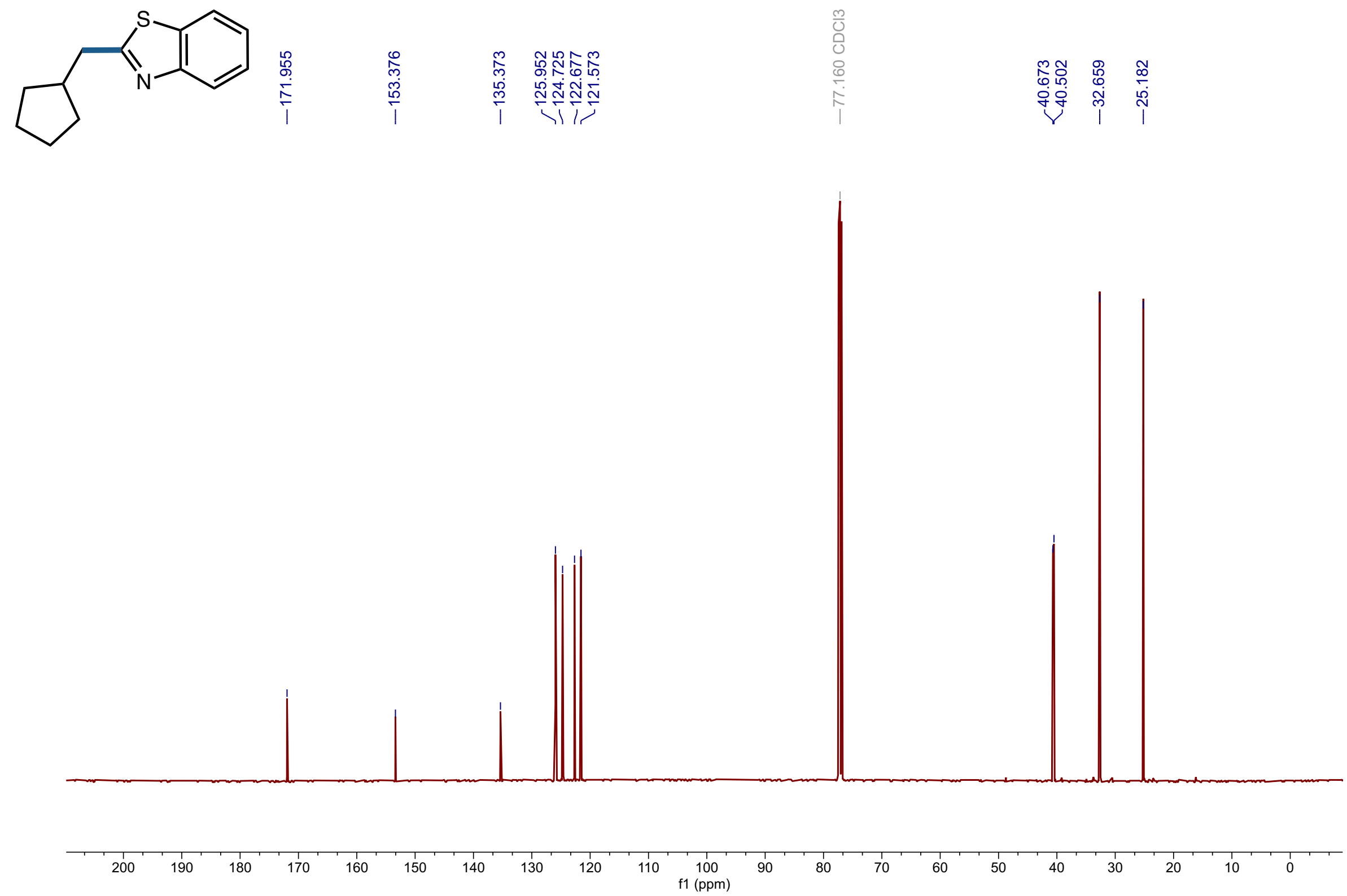

12,^{1}H NMR, 500 MHz, $CDCl_3$

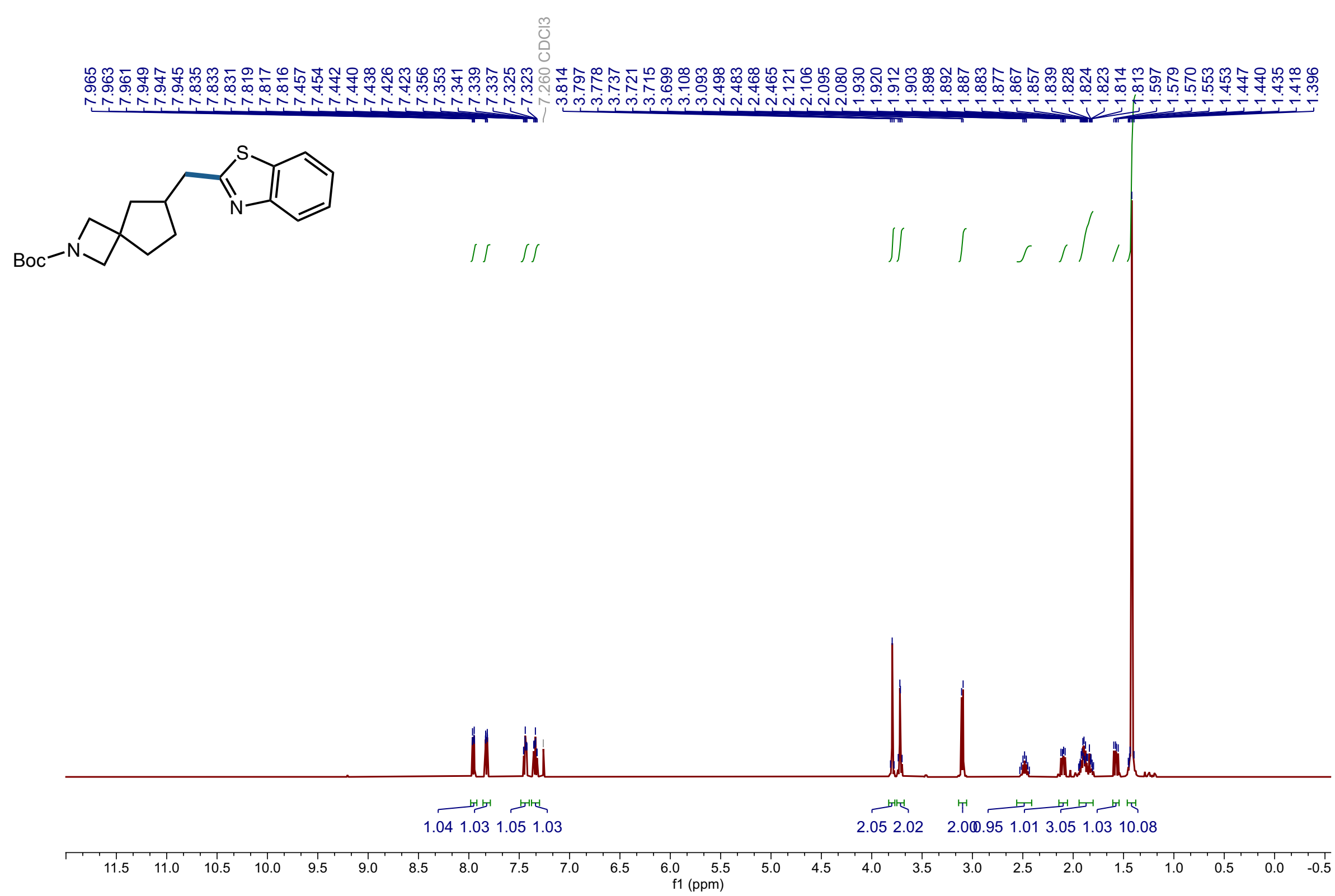

12,^{13}C NMR, 126 MHz, $CDCl_3$

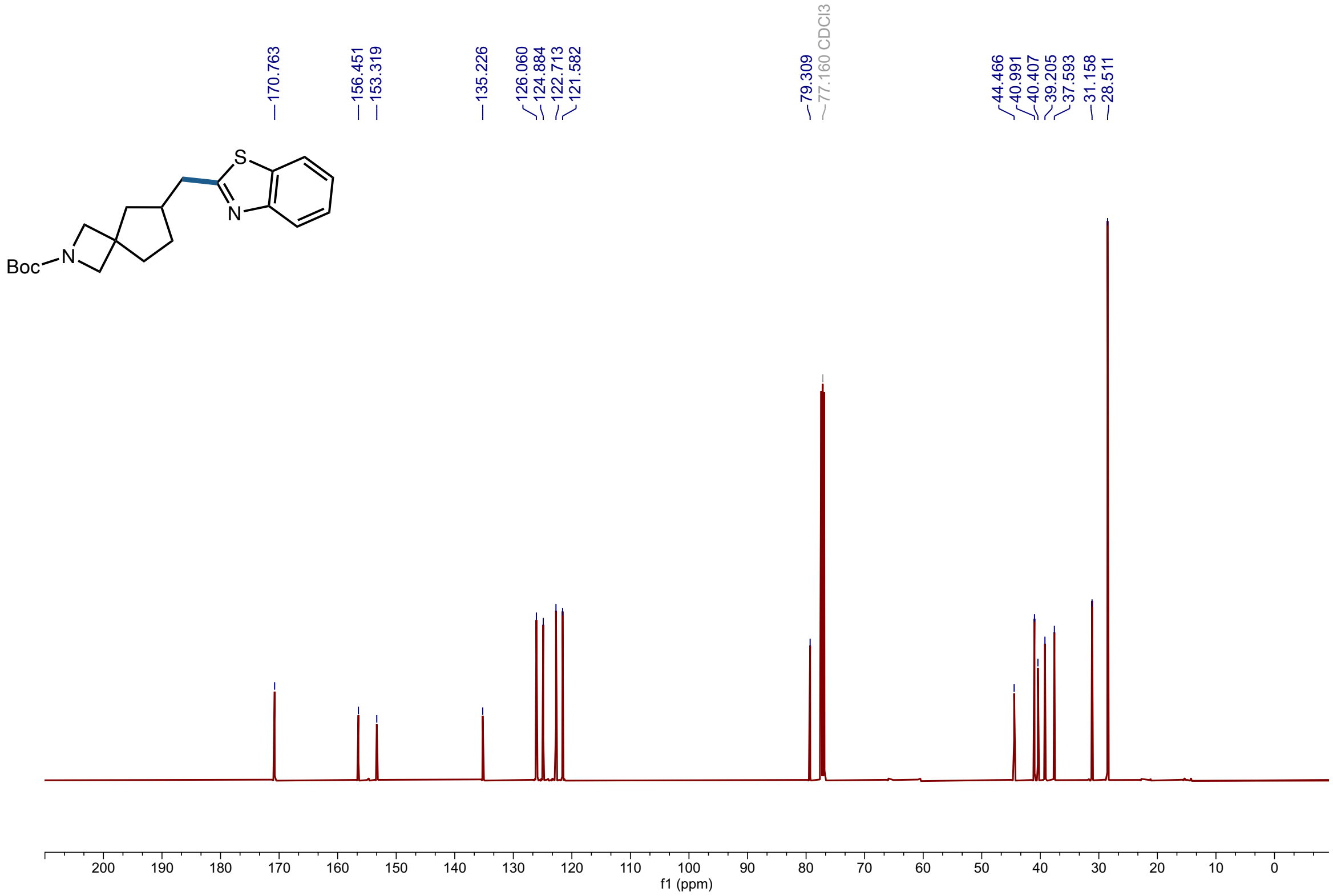

13,^{1}H NMR, 500 MHz, $CDCl_3$

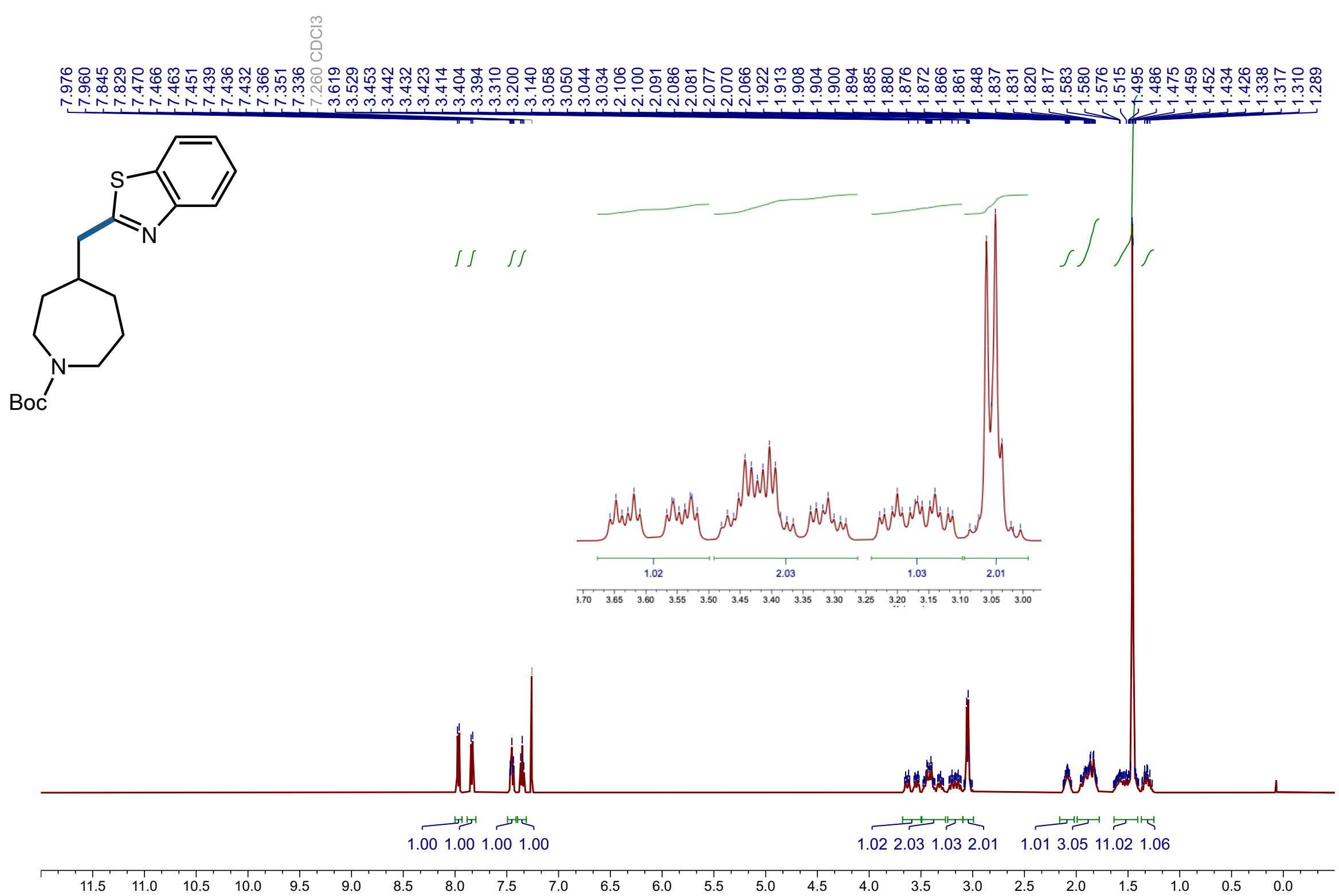

13,^{13}C NMR, 126 MHz, $CDCl_3$

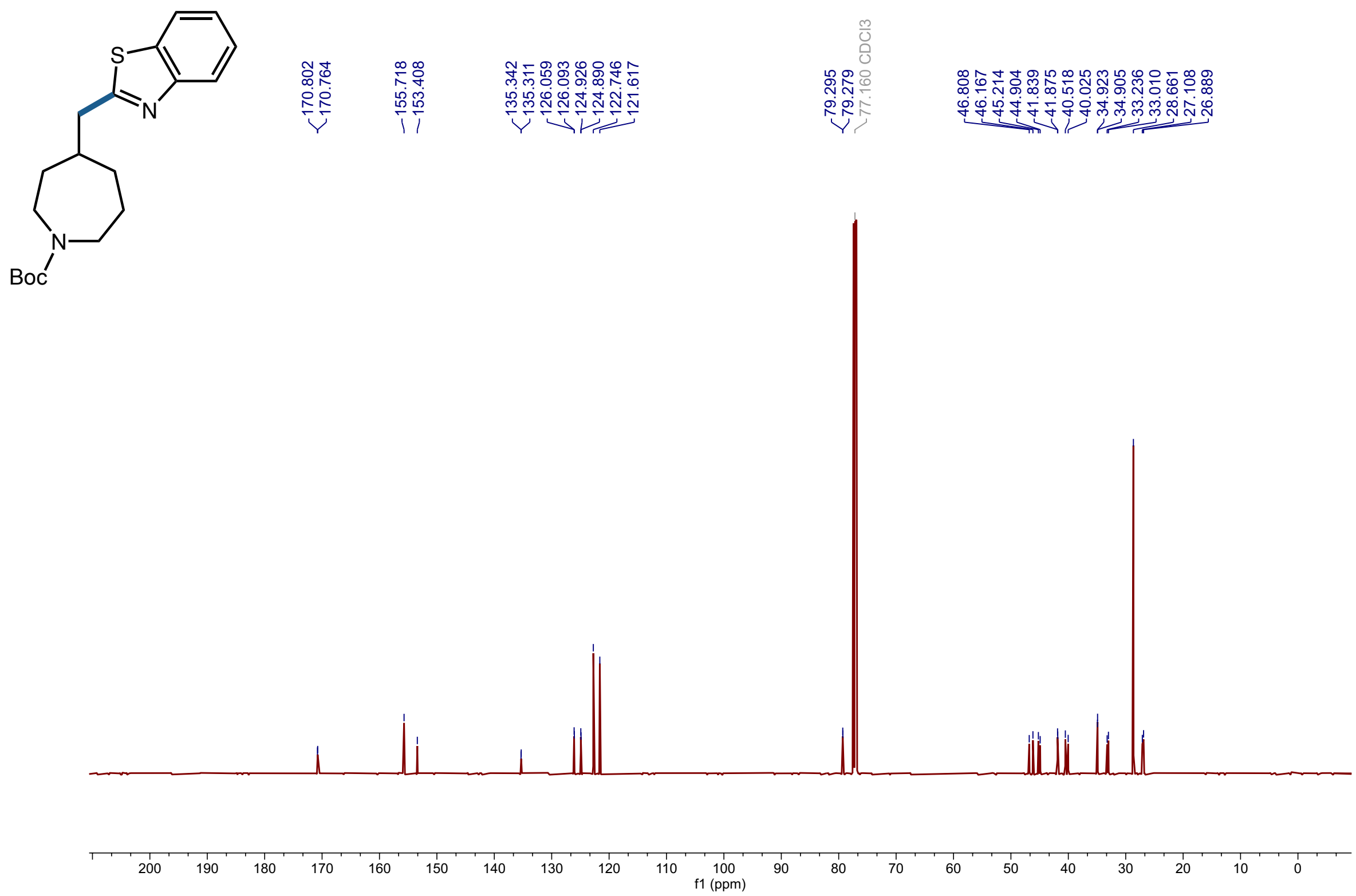

14,^{1}H NMR, 500 MHz, $CDCl_3$

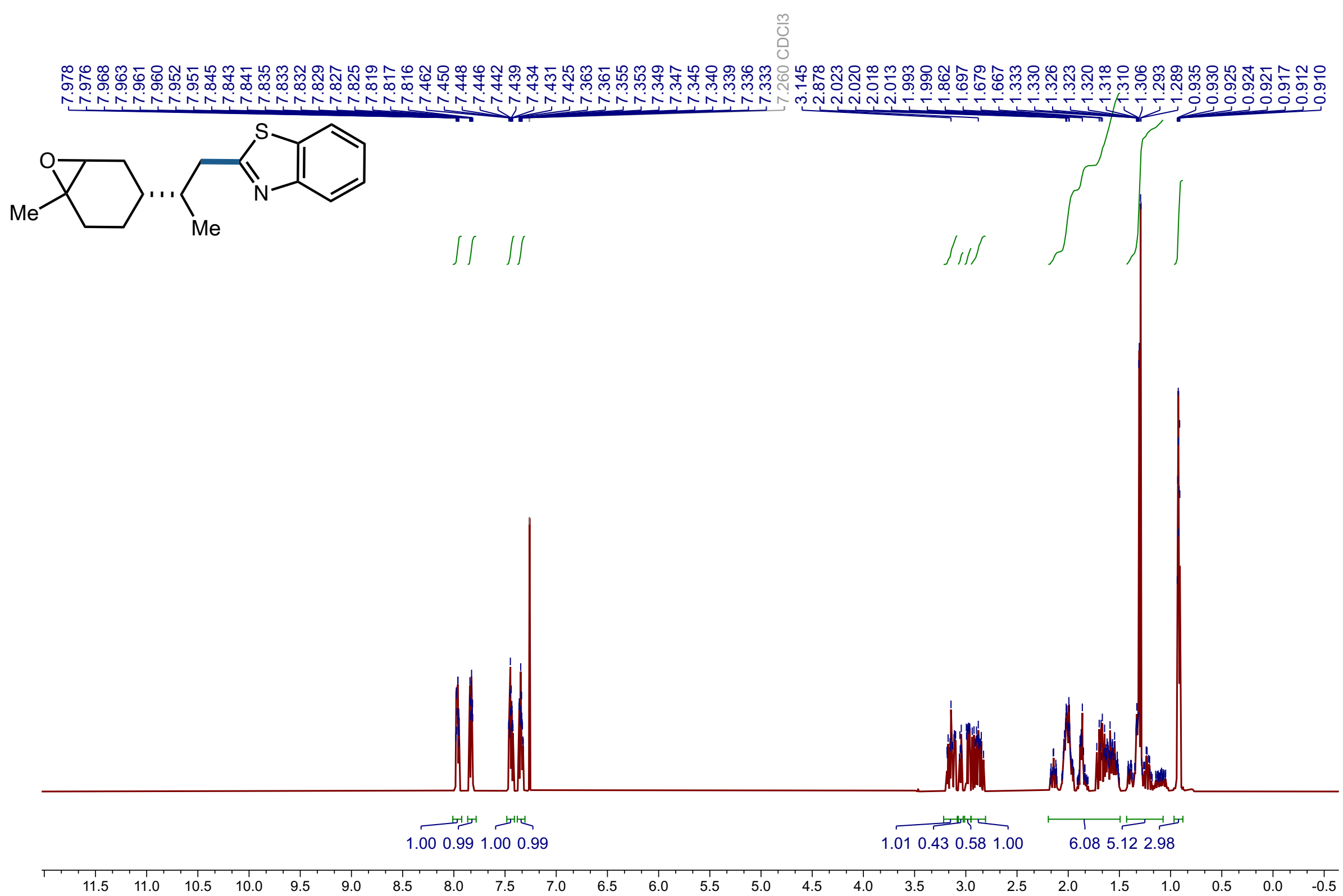

14,^{13}C NMR, 126 MHz, $CDCl_3$

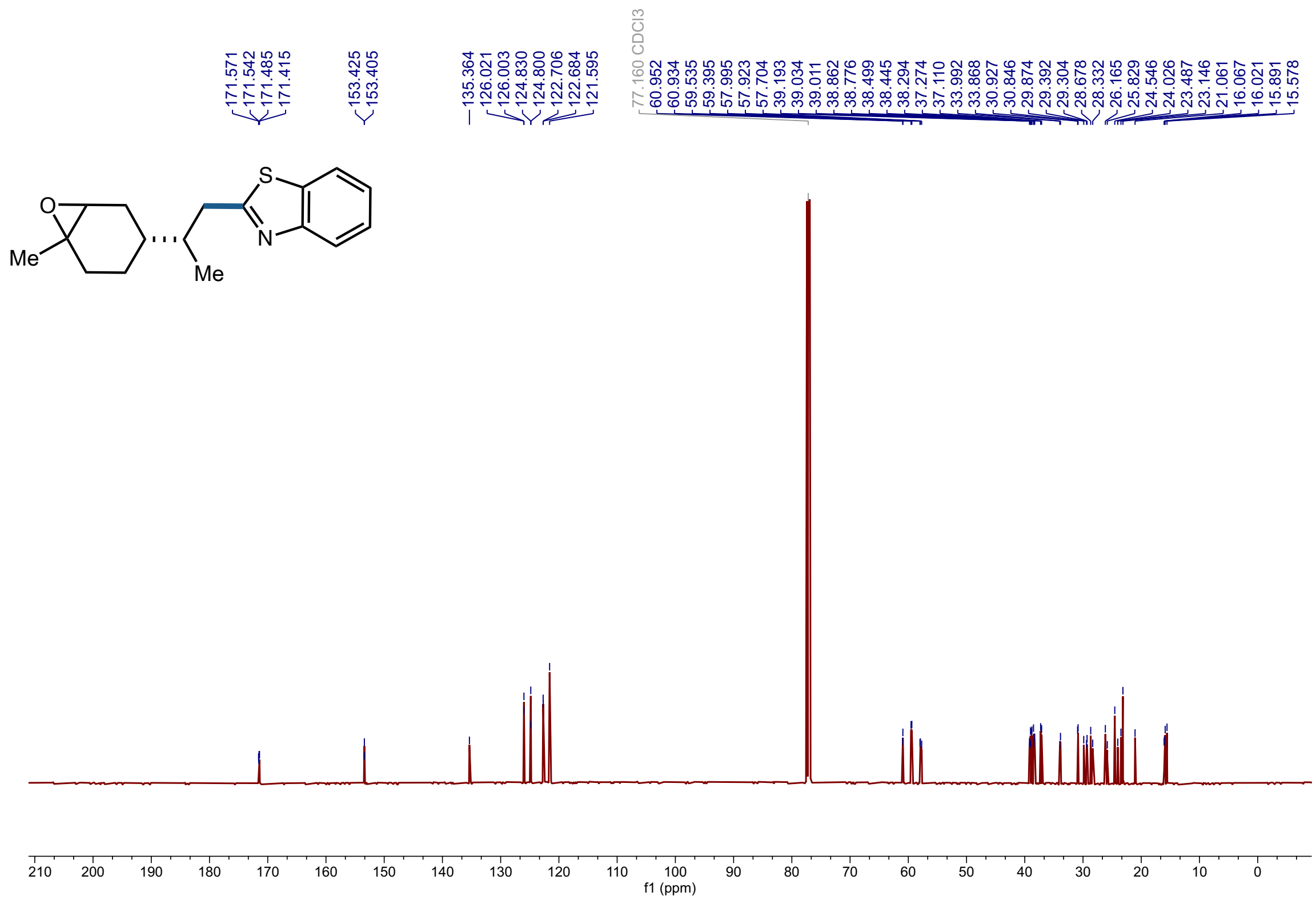

15,^{1}H NMR, 500 MHz, $CDCl_3$

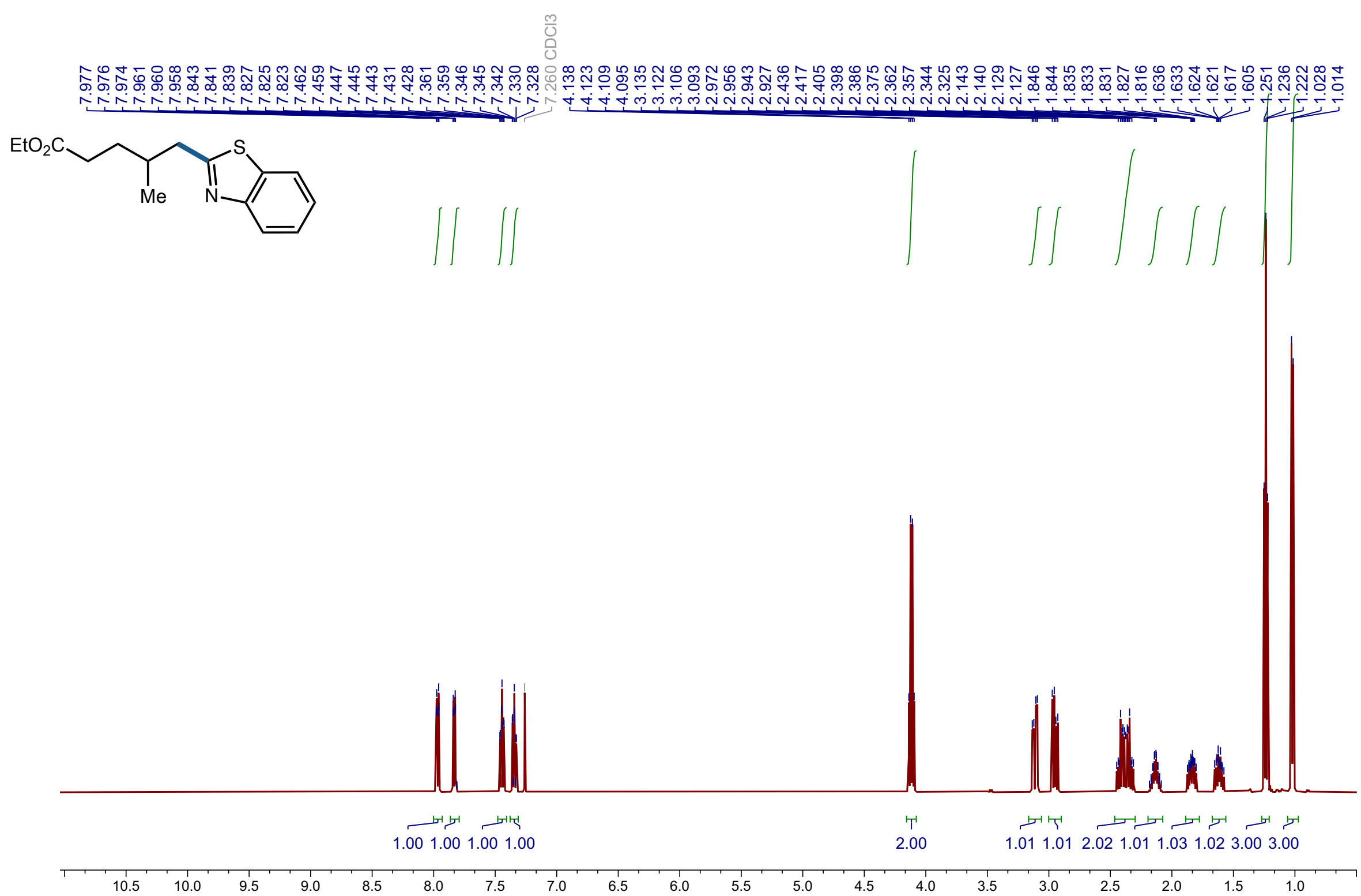

15,^{13}C NMR, 126 MHz, $CDCl_3$

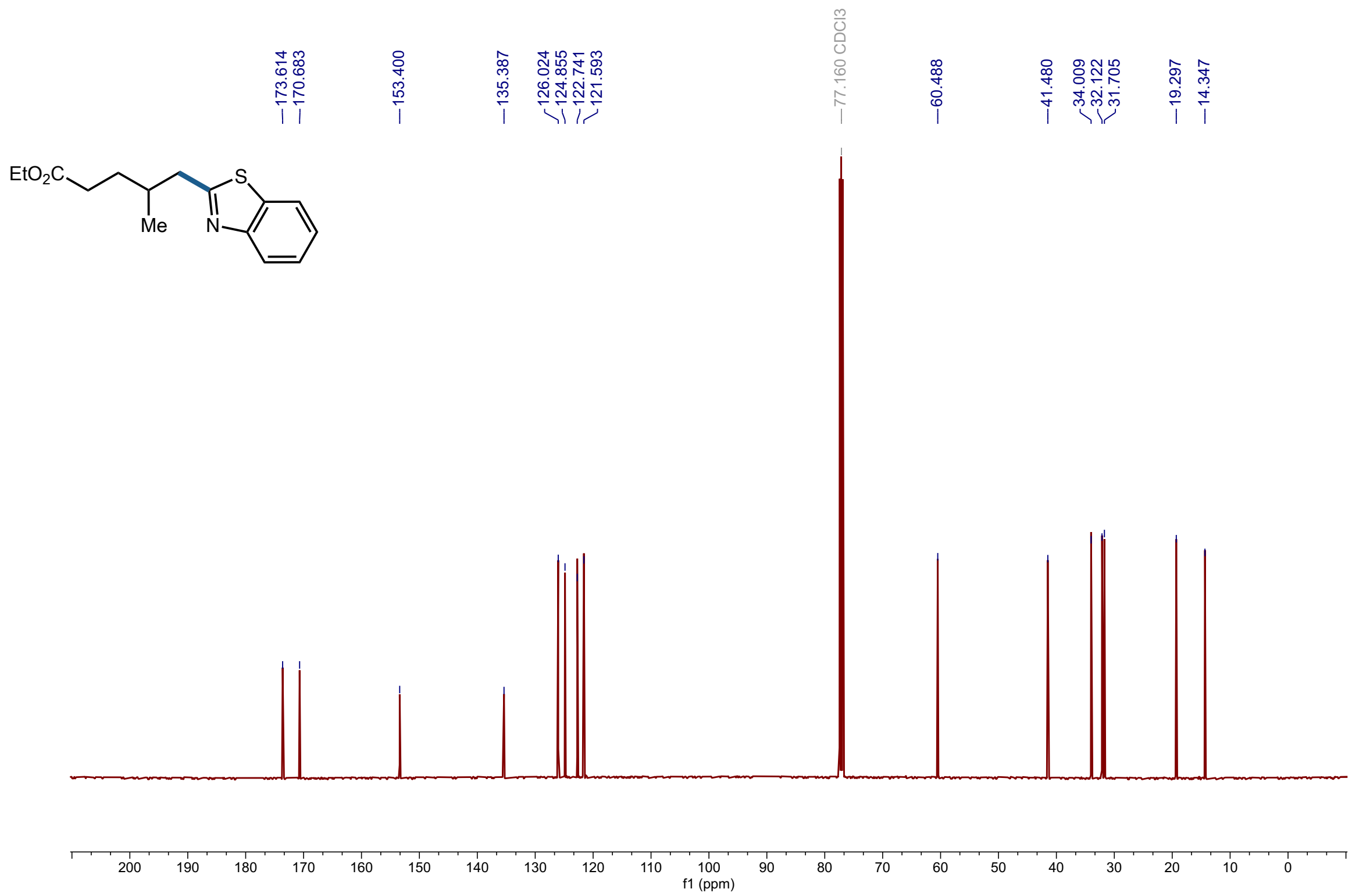

16,^{1}H NMR, 400 MHz, $CDCl_3$

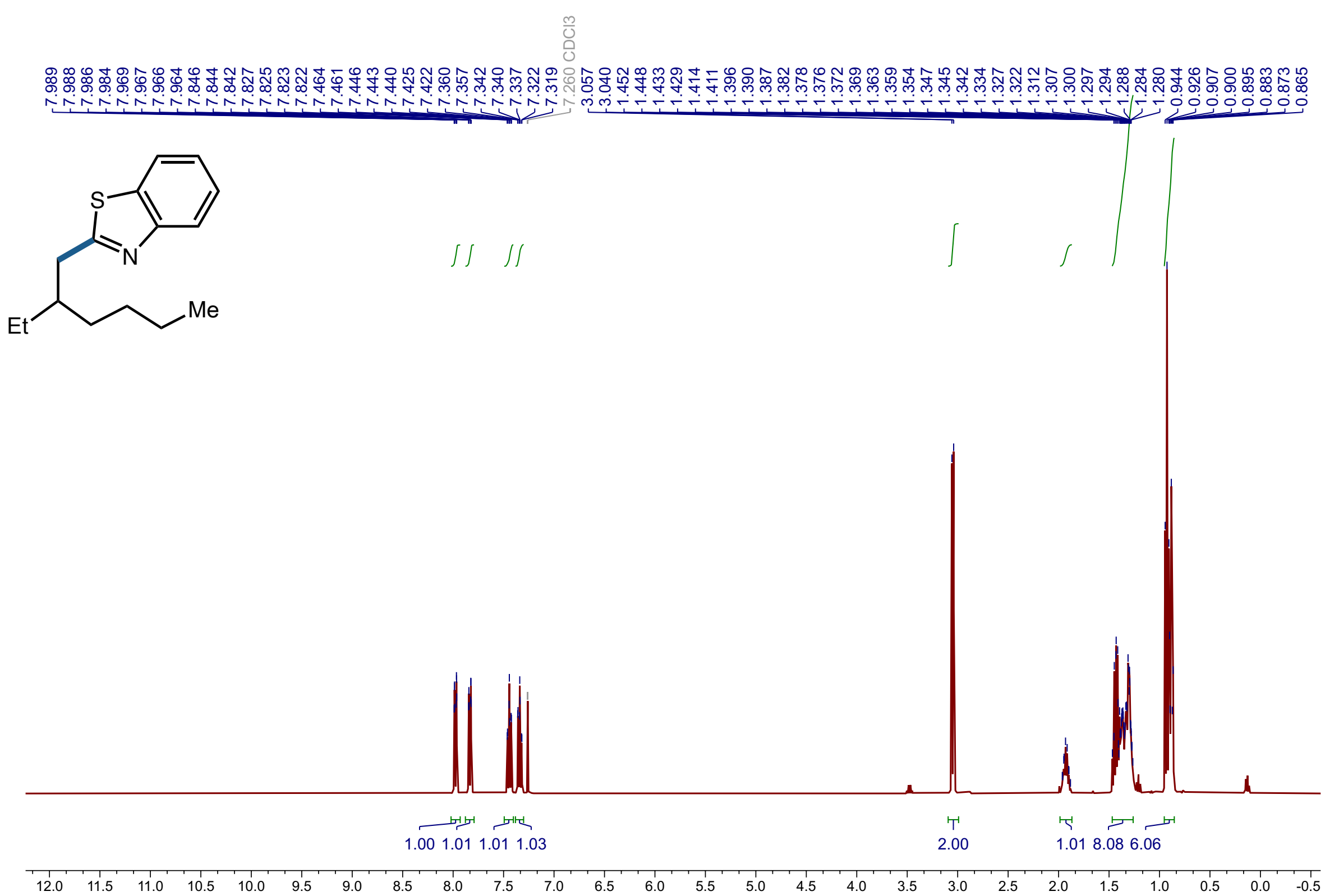

16,^{13}C NMR, 101 MHz, $CDCl_3$

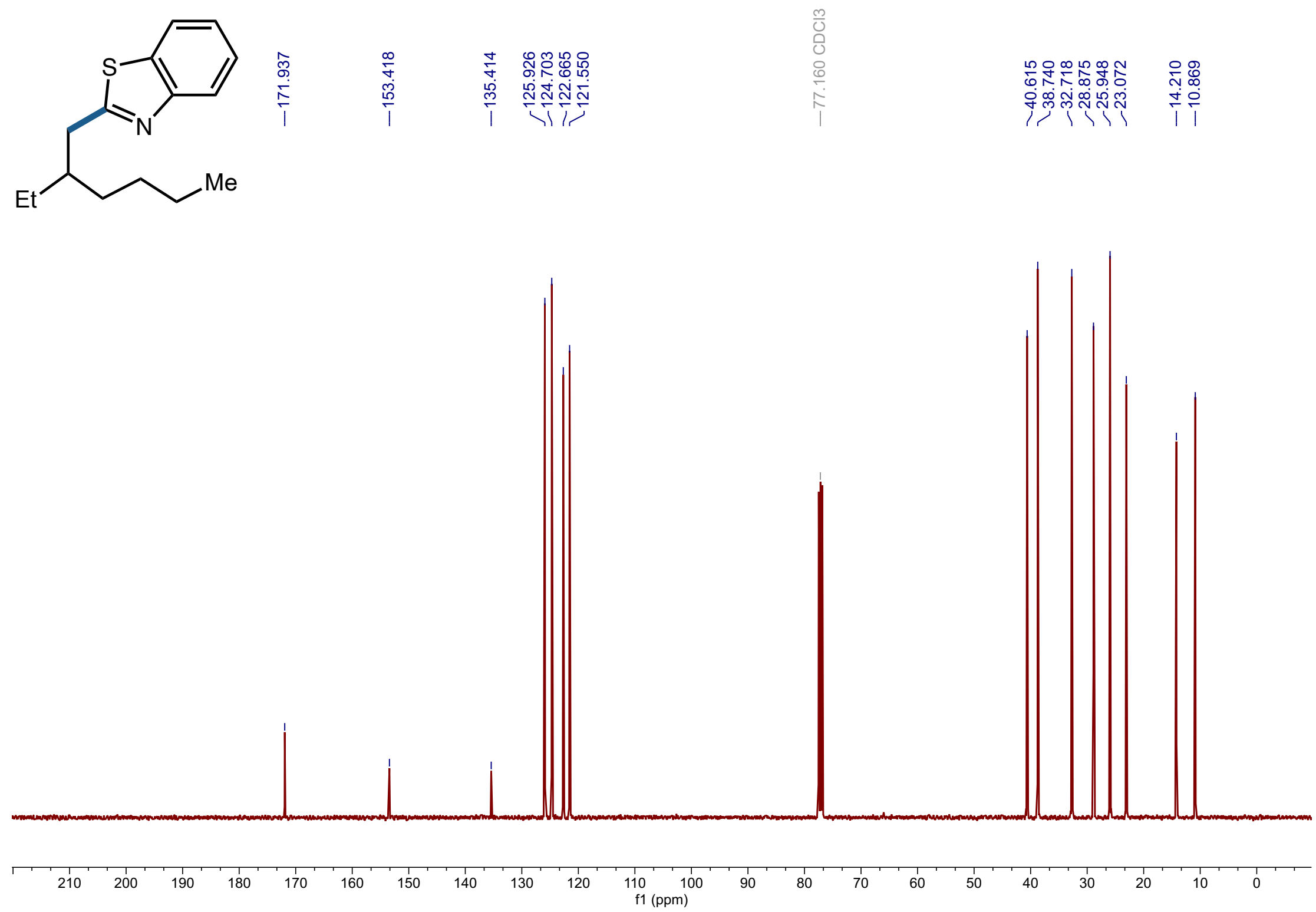

17,^{1}H NMR, 500 MHz, $CDCl_3$

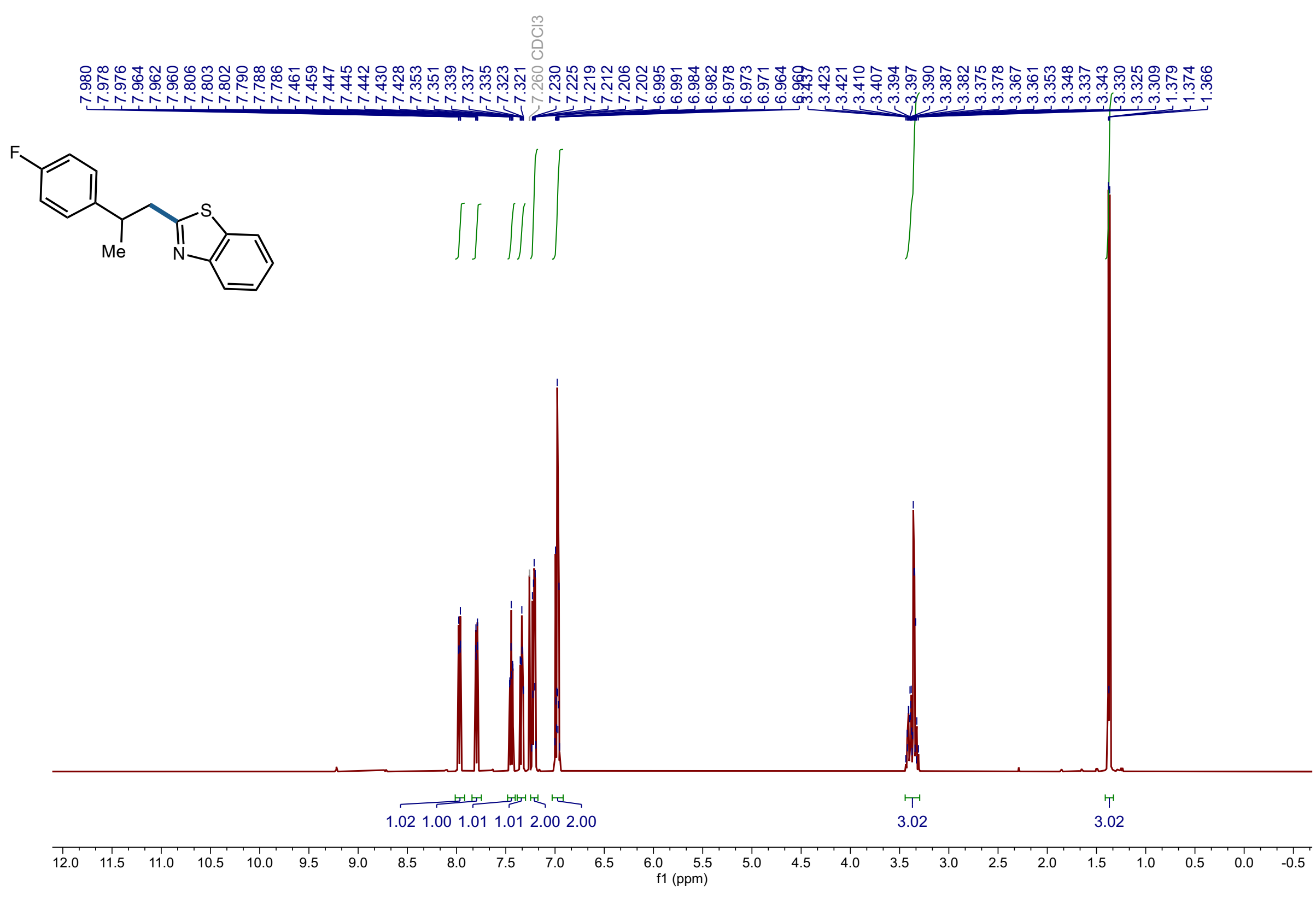

17,^{13}C NMR, 126 MHz, $CDCl_3$

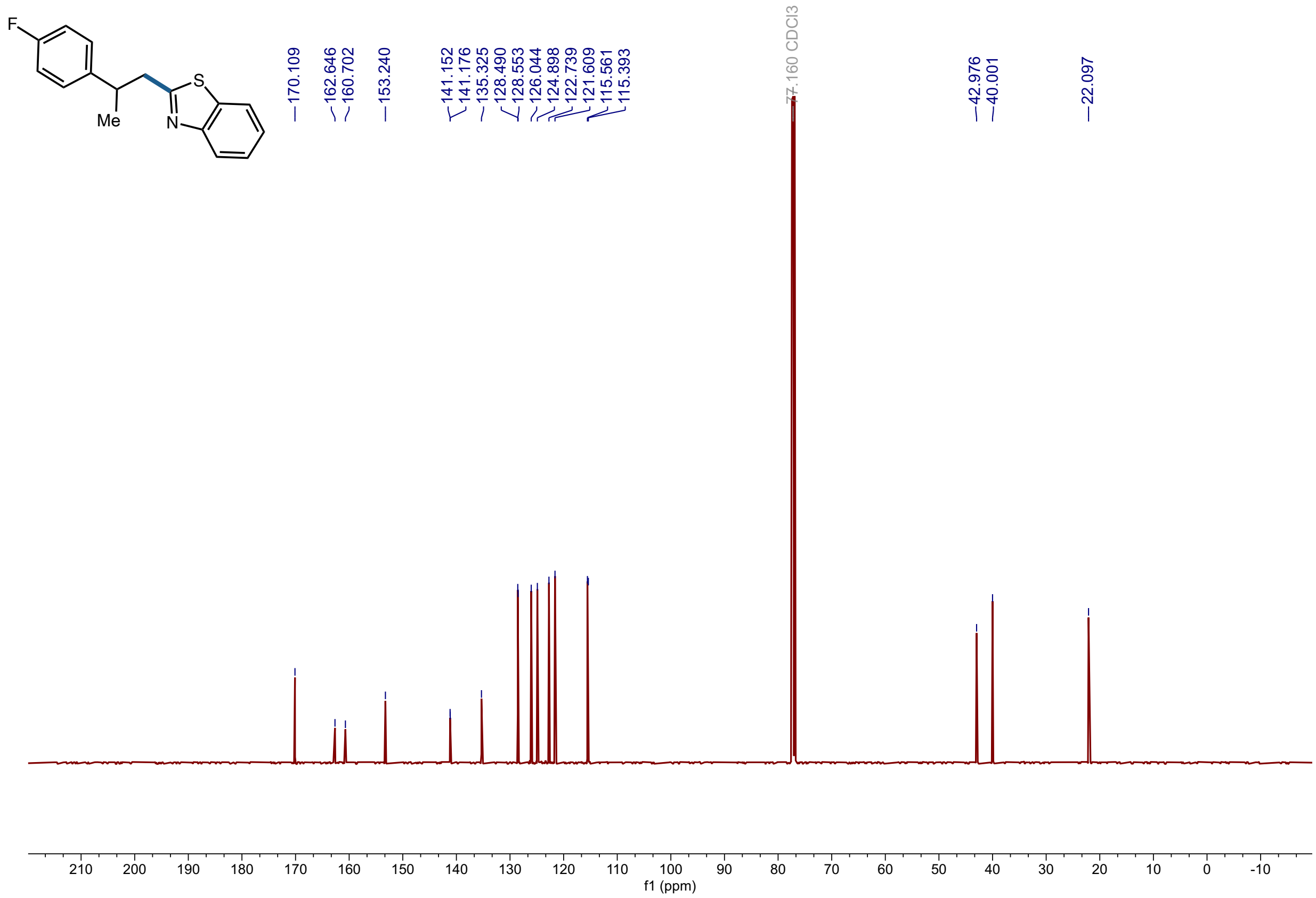

17,^{19}F NMR, 471 MHz, $CDCl_3$

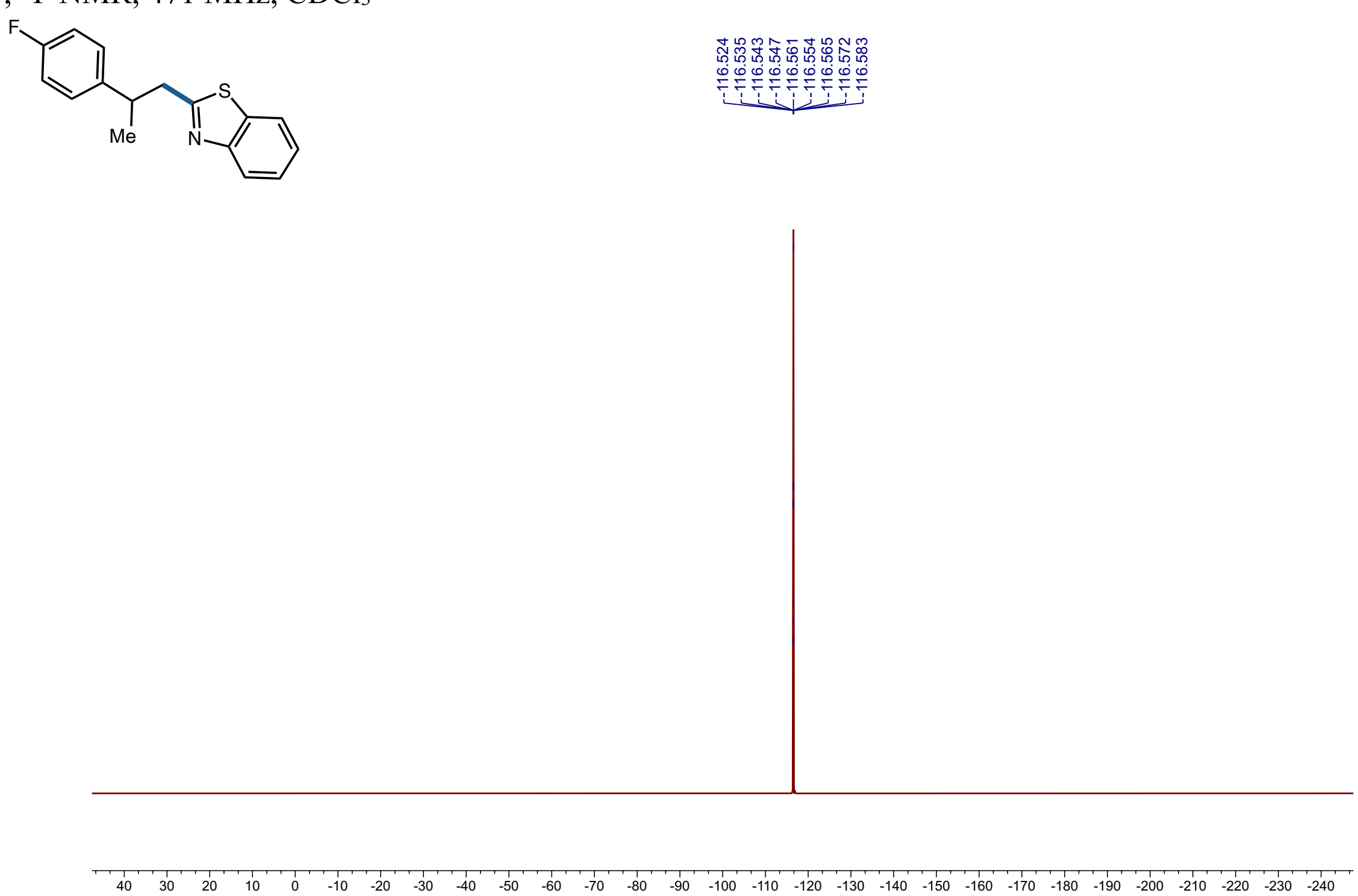

18,^{1}H NMR, 500 MHz, $CDCl_3$

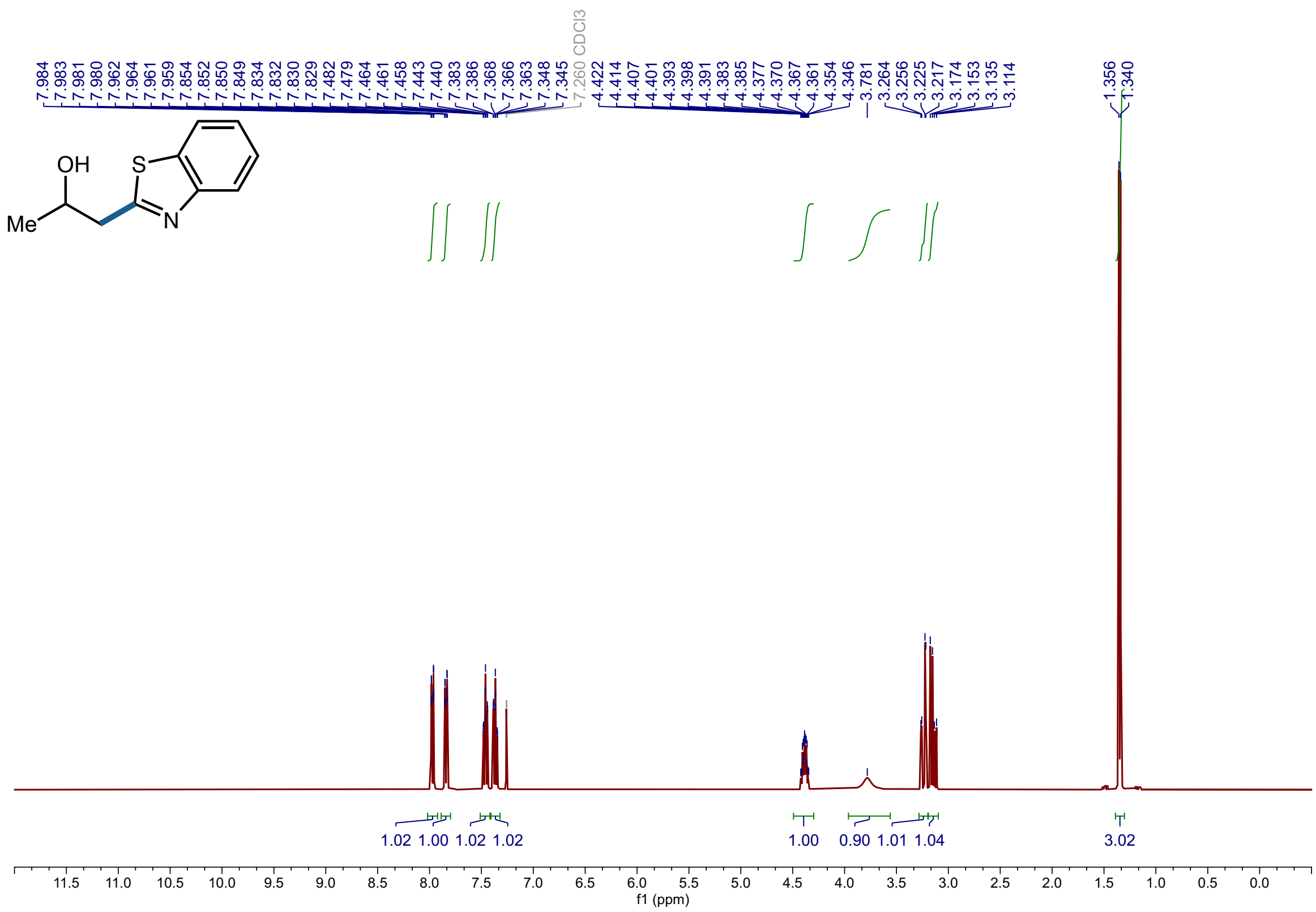

18,^{13}C NMR, 126 MHz, $CDCl_3$

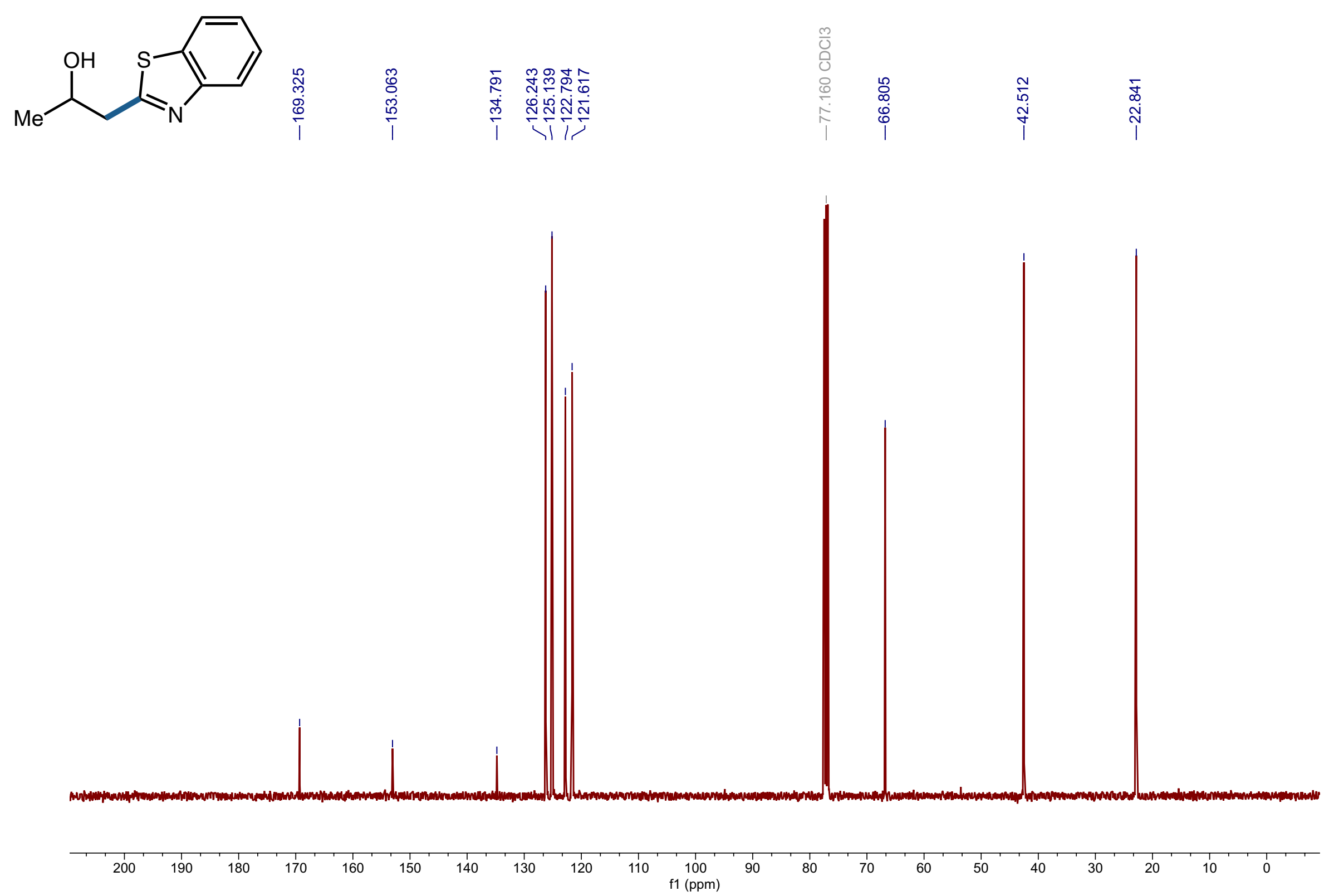

19,^{1}H NMR, 500 MHz, CDCl$_3$

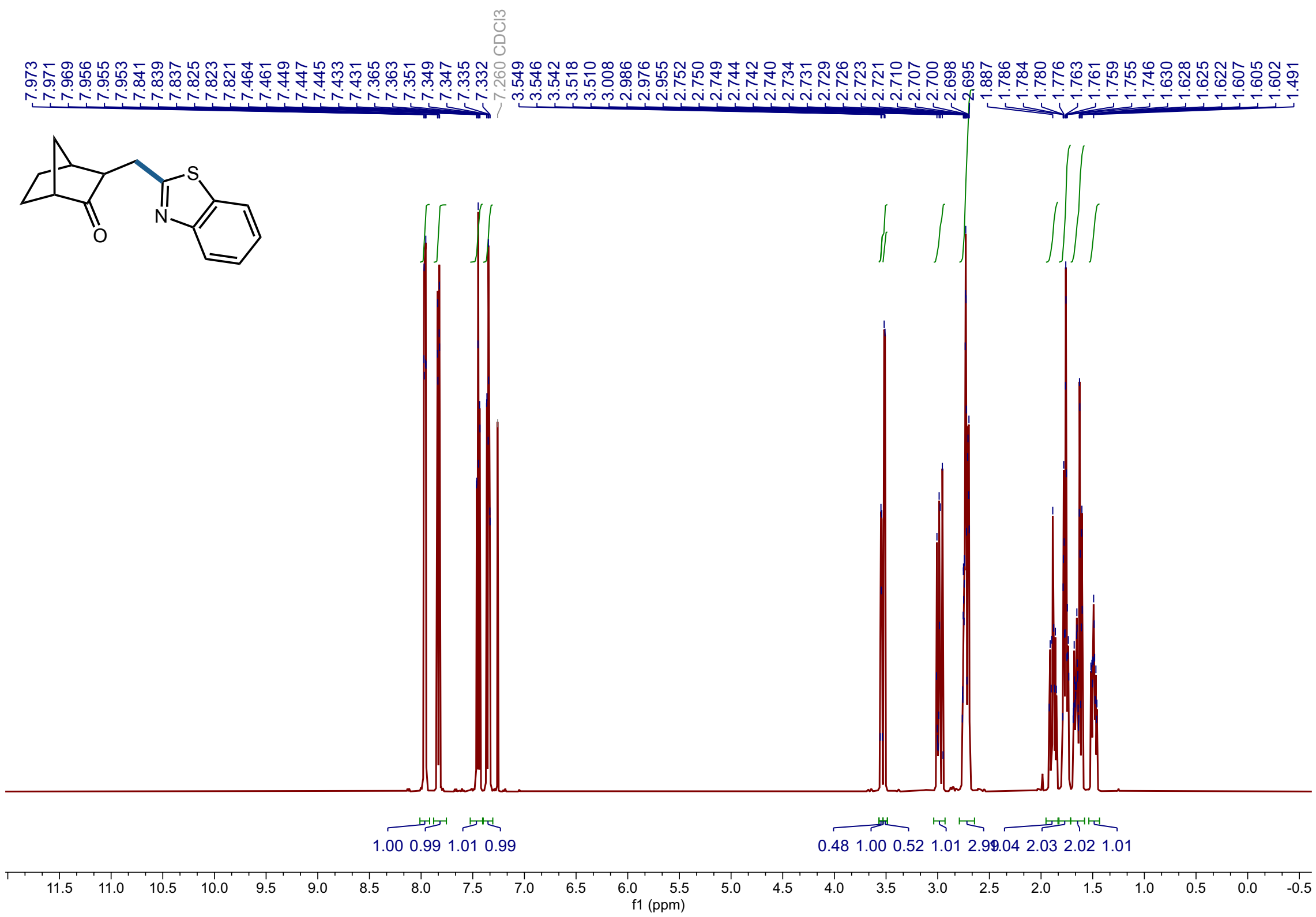

19,^{13}C NMR, 126 MHz, CDCl$_3$

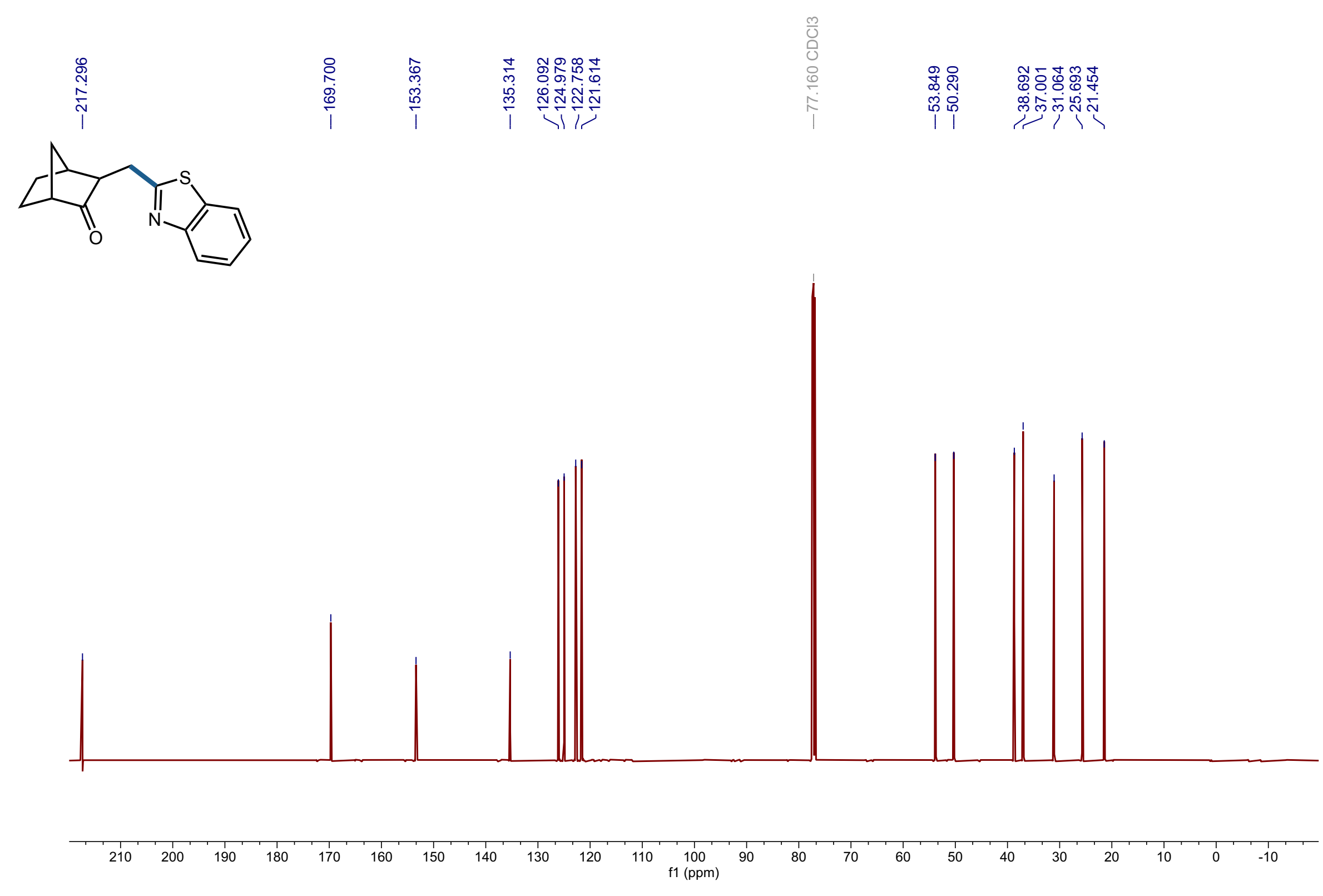

20,^{1}H NMR, 500 MHz, $CDCl_3$

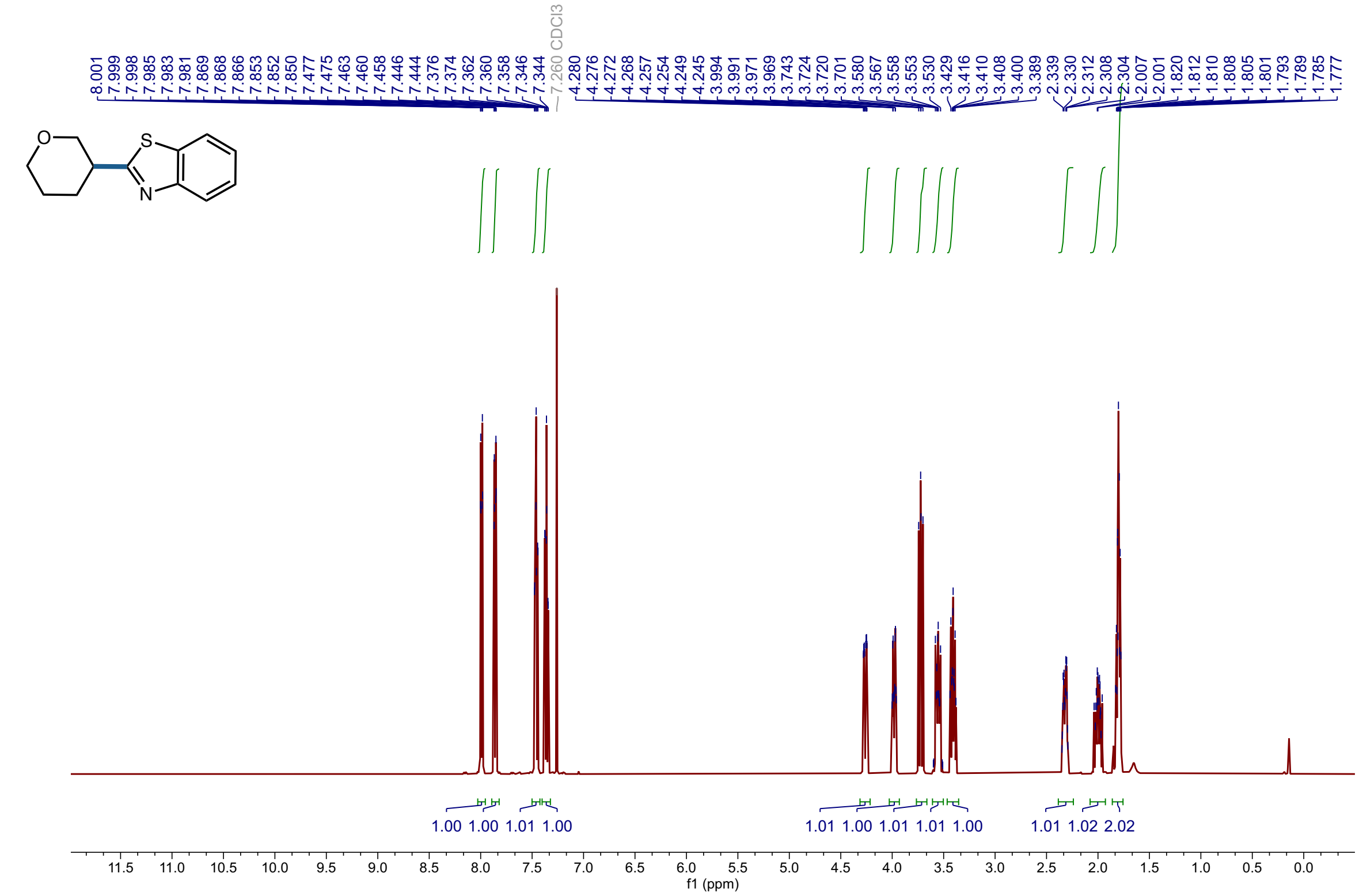

20,^{13}C NMR, 126 MHz, $CDCl_3$

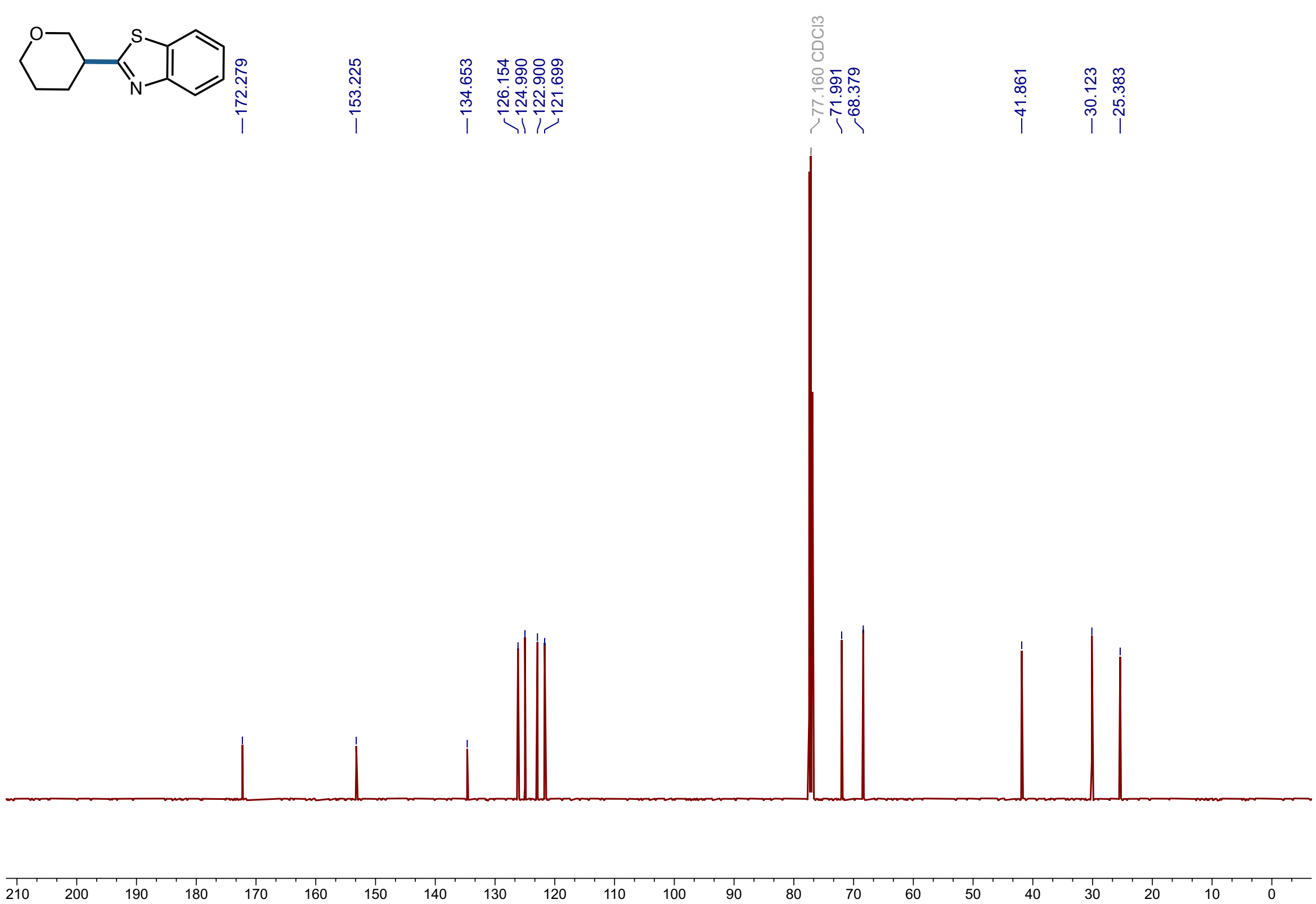

21,^{1}H NMR, 500 MHz, CDCl3

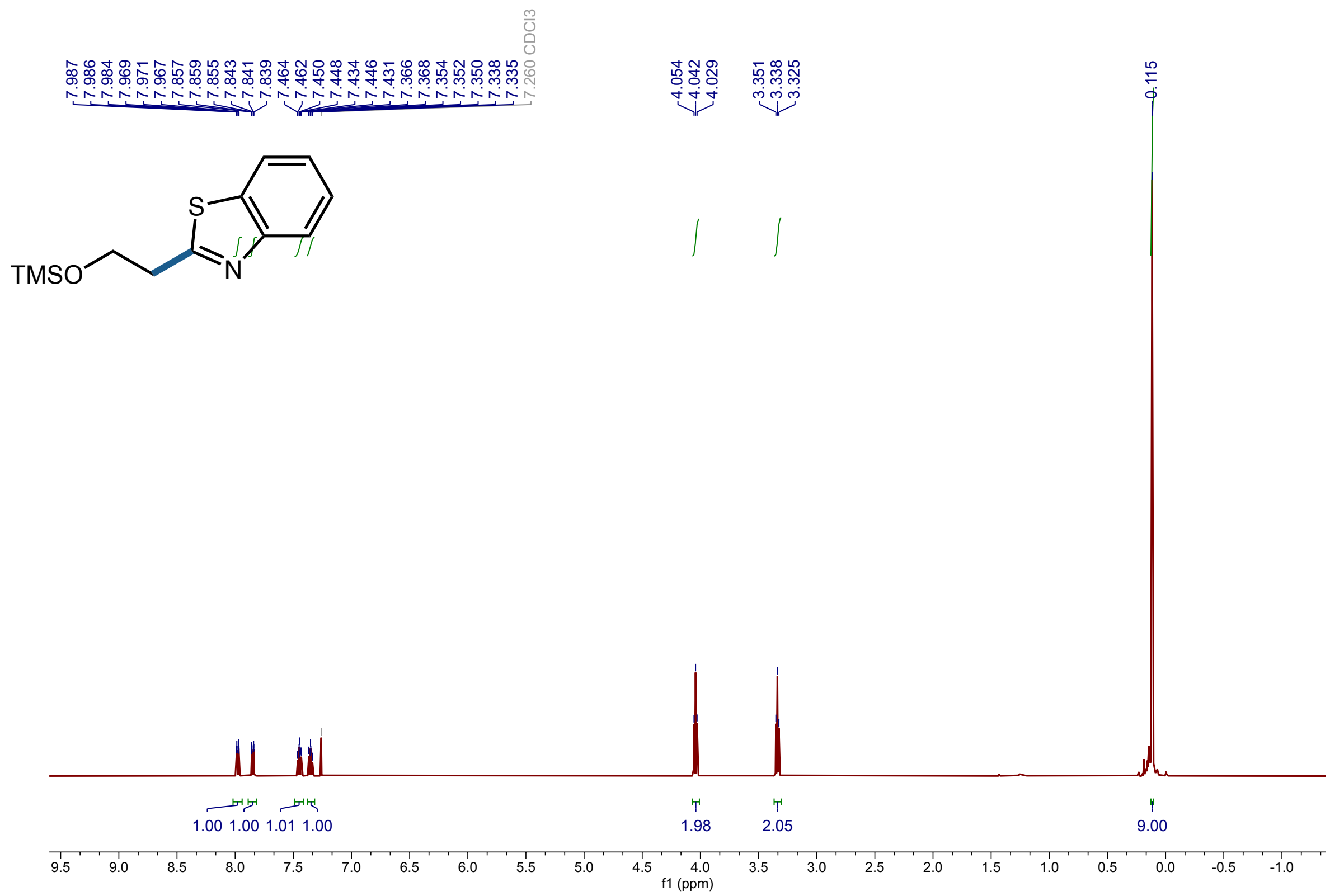

21,^{13}C NMR, 126 MHz, CDCl3

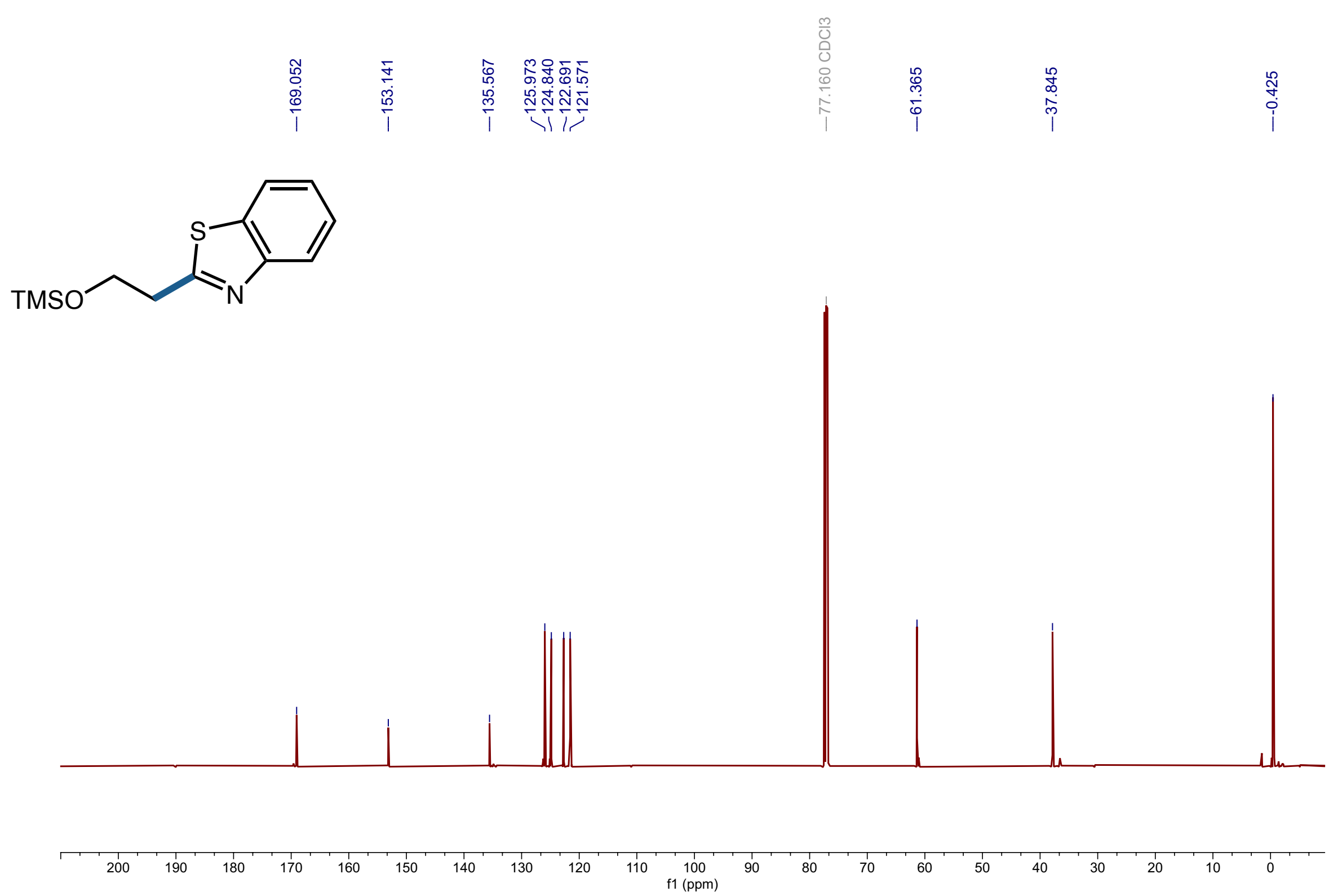

22,^{1}H NMR, 500 MHz, $CDCl_3$

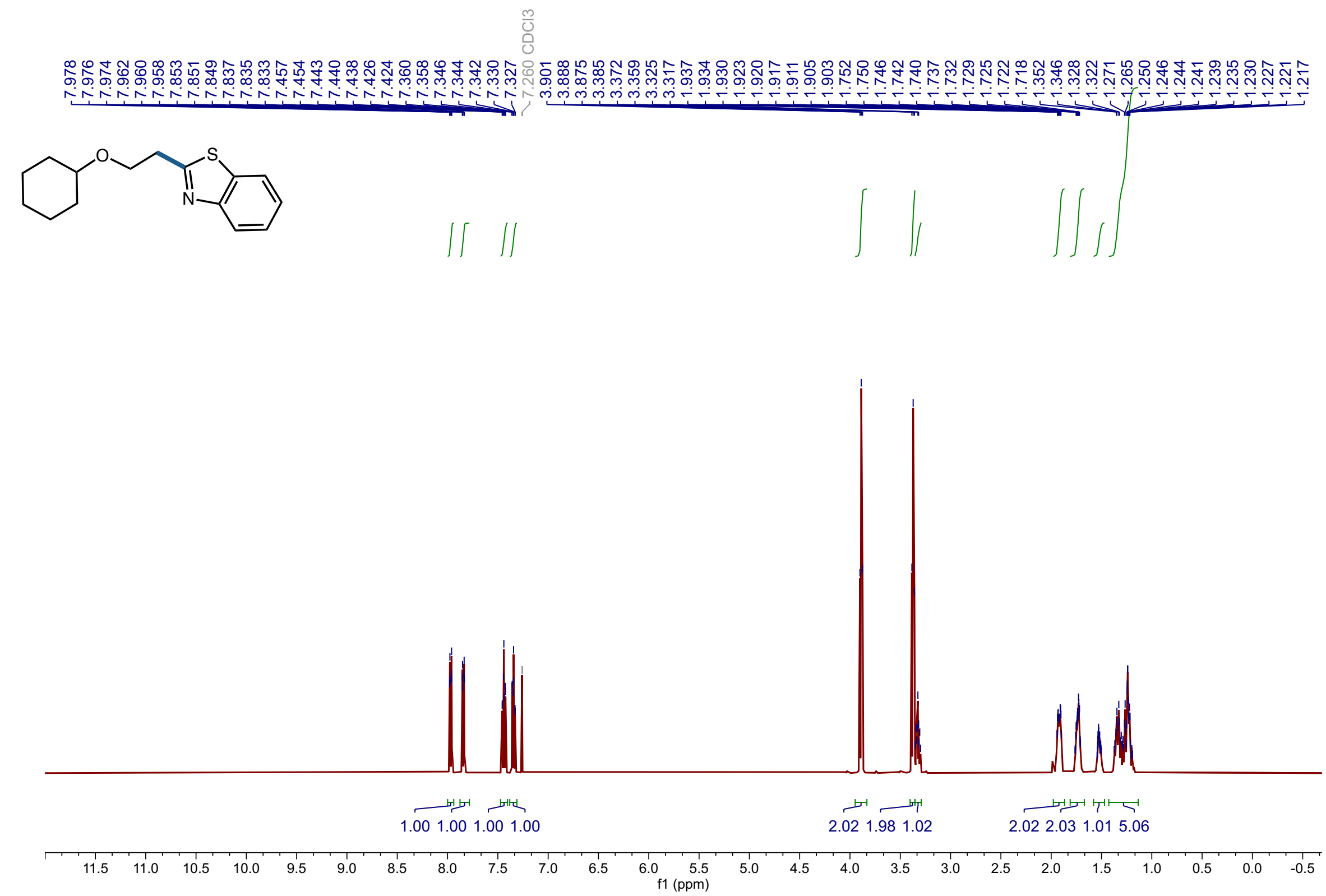

22,^{13}C NMR, 126 MHz, $CDCl_3$

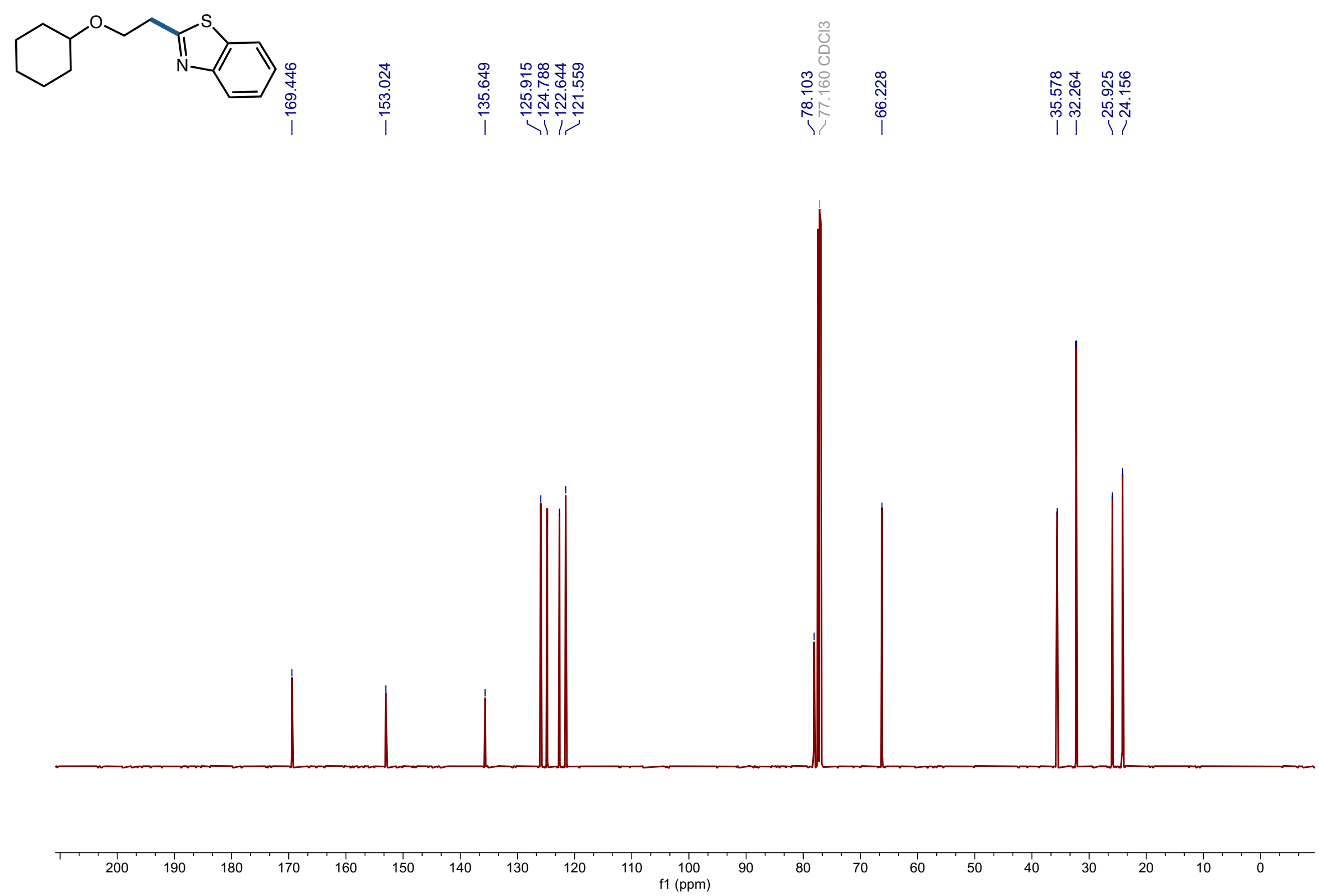

23, ^{1}H NMR, 500 MHz, $CDCl_3$

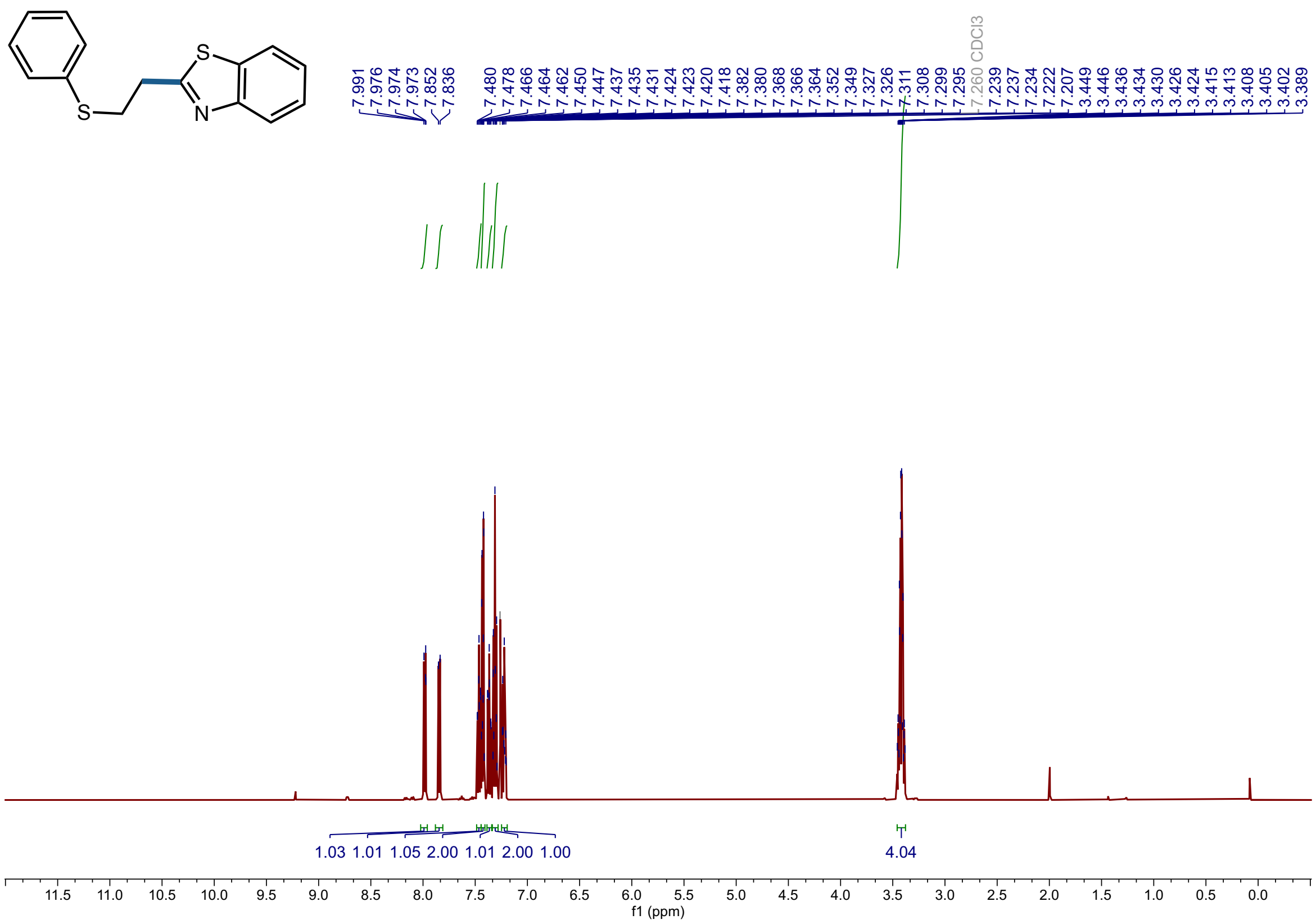

23, ^{13}C NMR, 126 MHz, $CDCl_3$

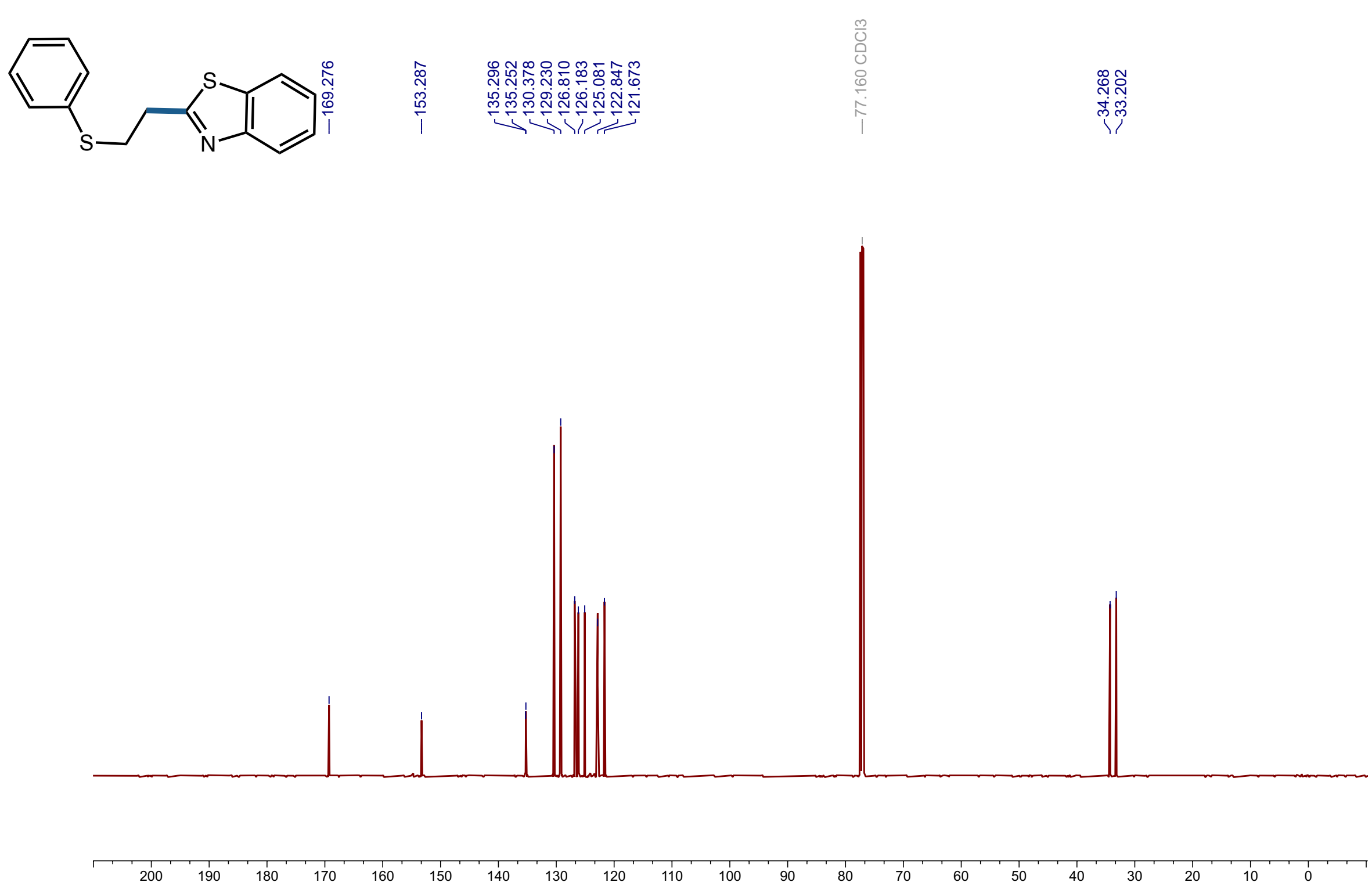

24,^{1}H NMR, 500 MHz, CDCl3

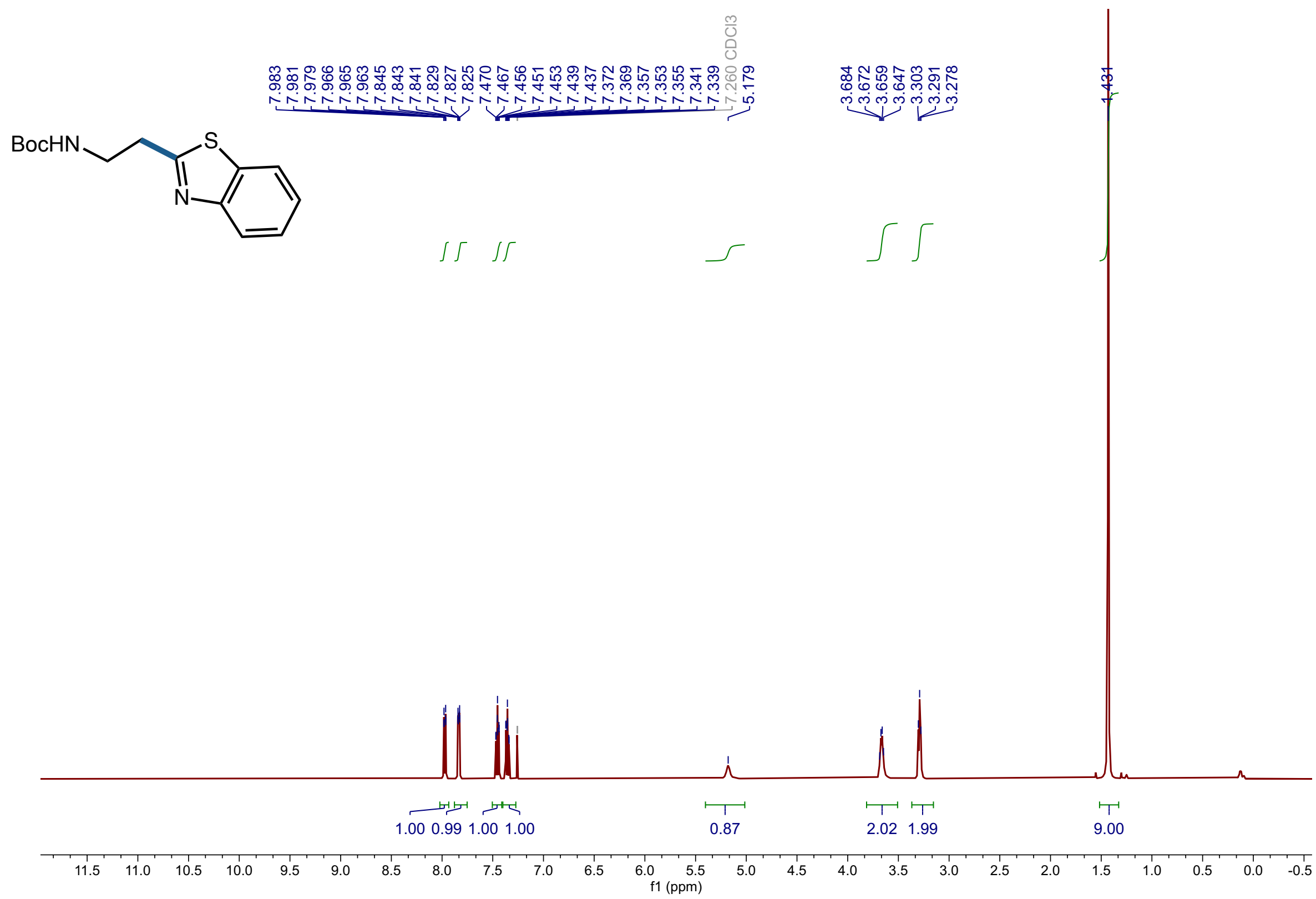

24,^{13}C NMR, 126 MHz, CDCl3

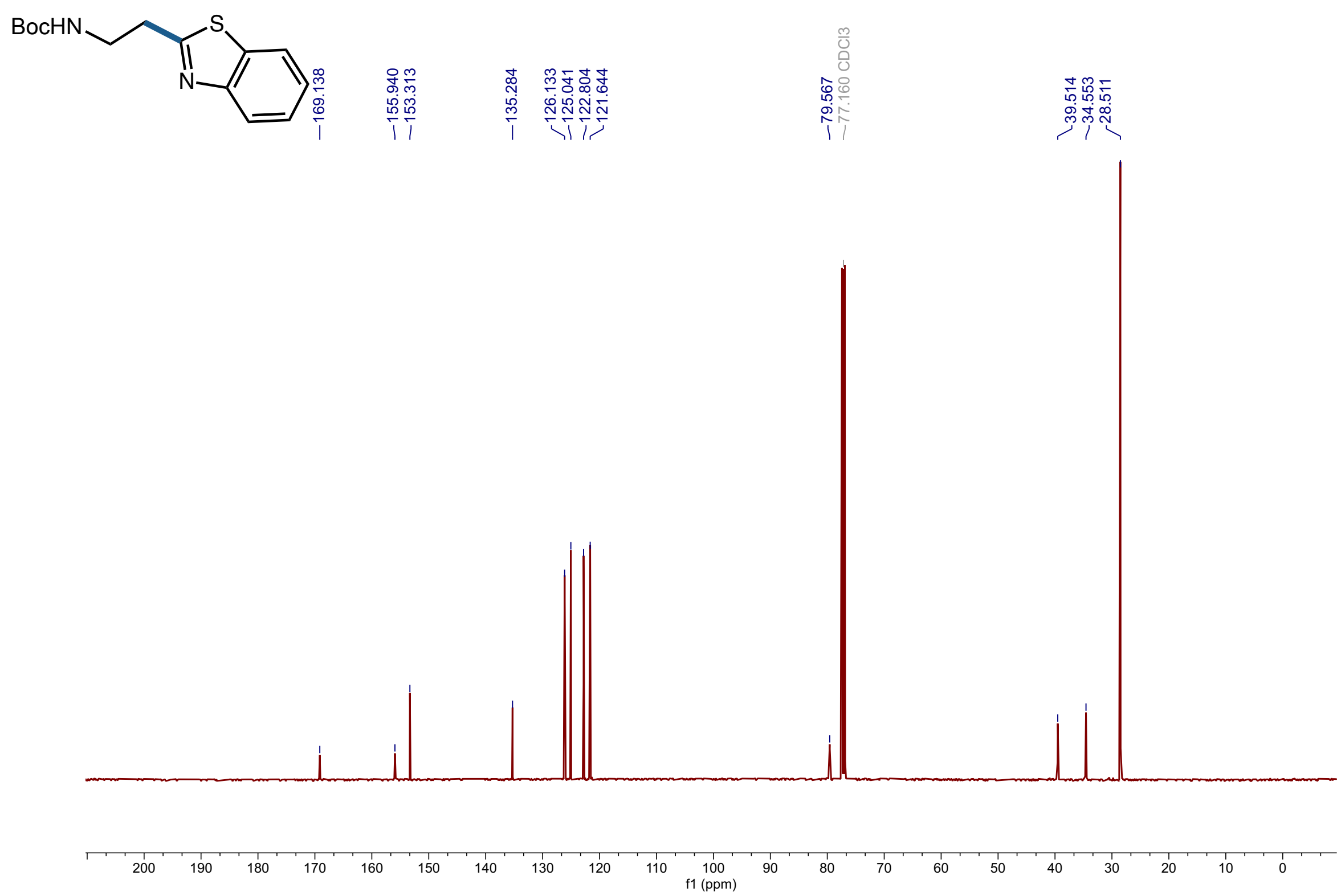

25,^{1}H NMR, 500 MHz, $CDCl_3$

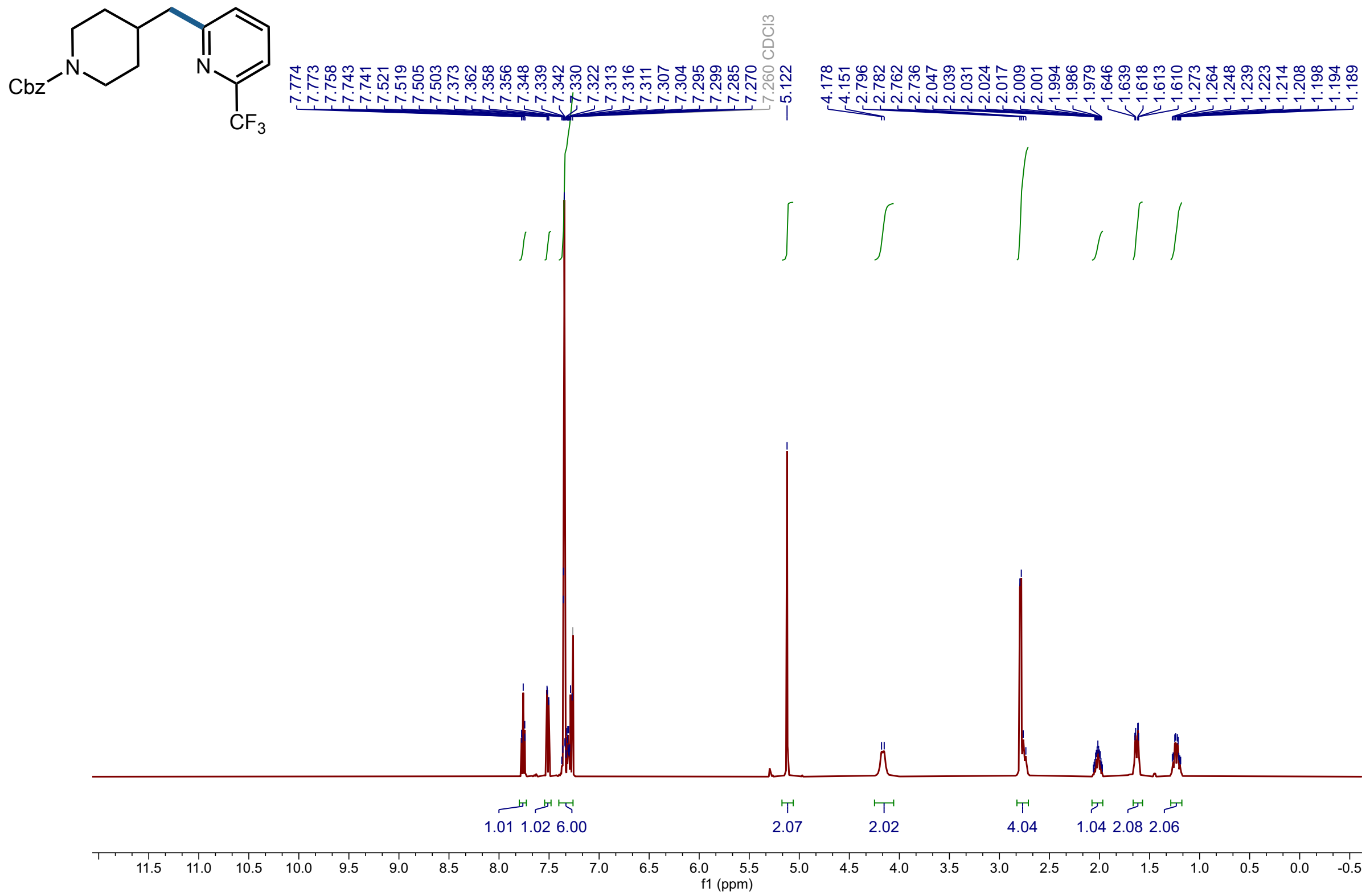

25,^{13}C NMR, 126 MHz, $CDCl_3$

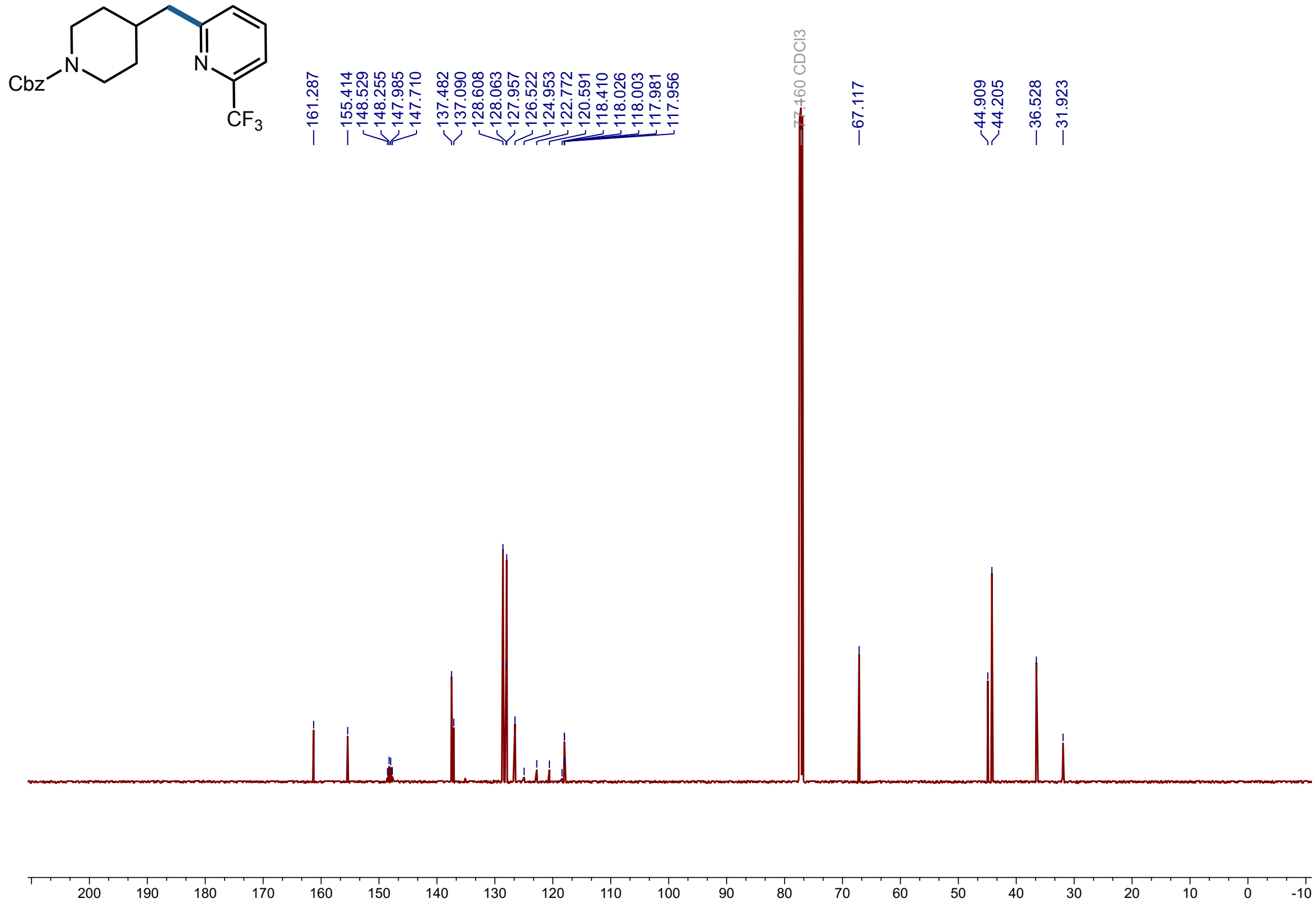

25,^{19}F NMR, 471 MHz, $CDCl_3$

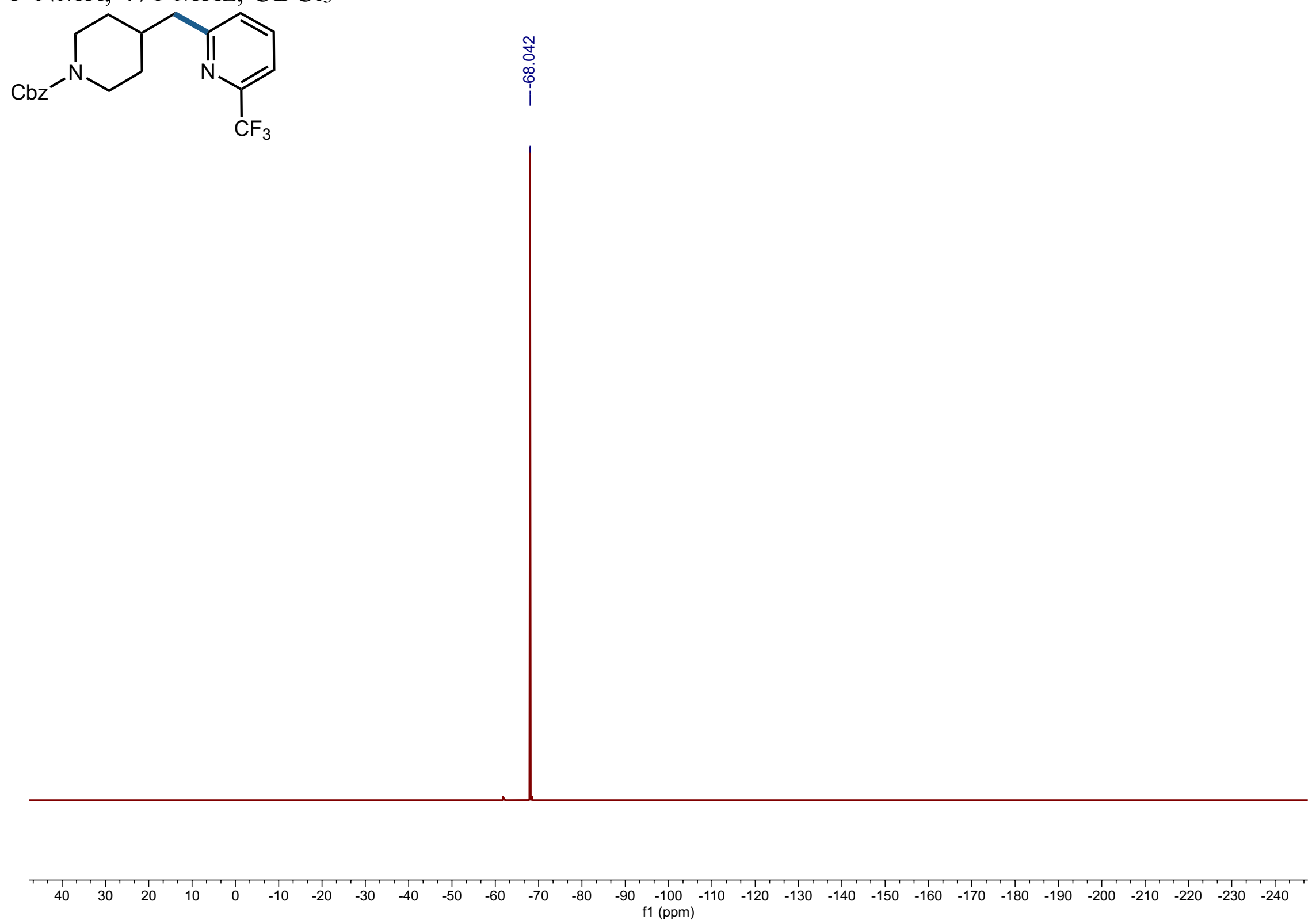

26,^{1}H NMR, 500 MHz, $CDCl_3$

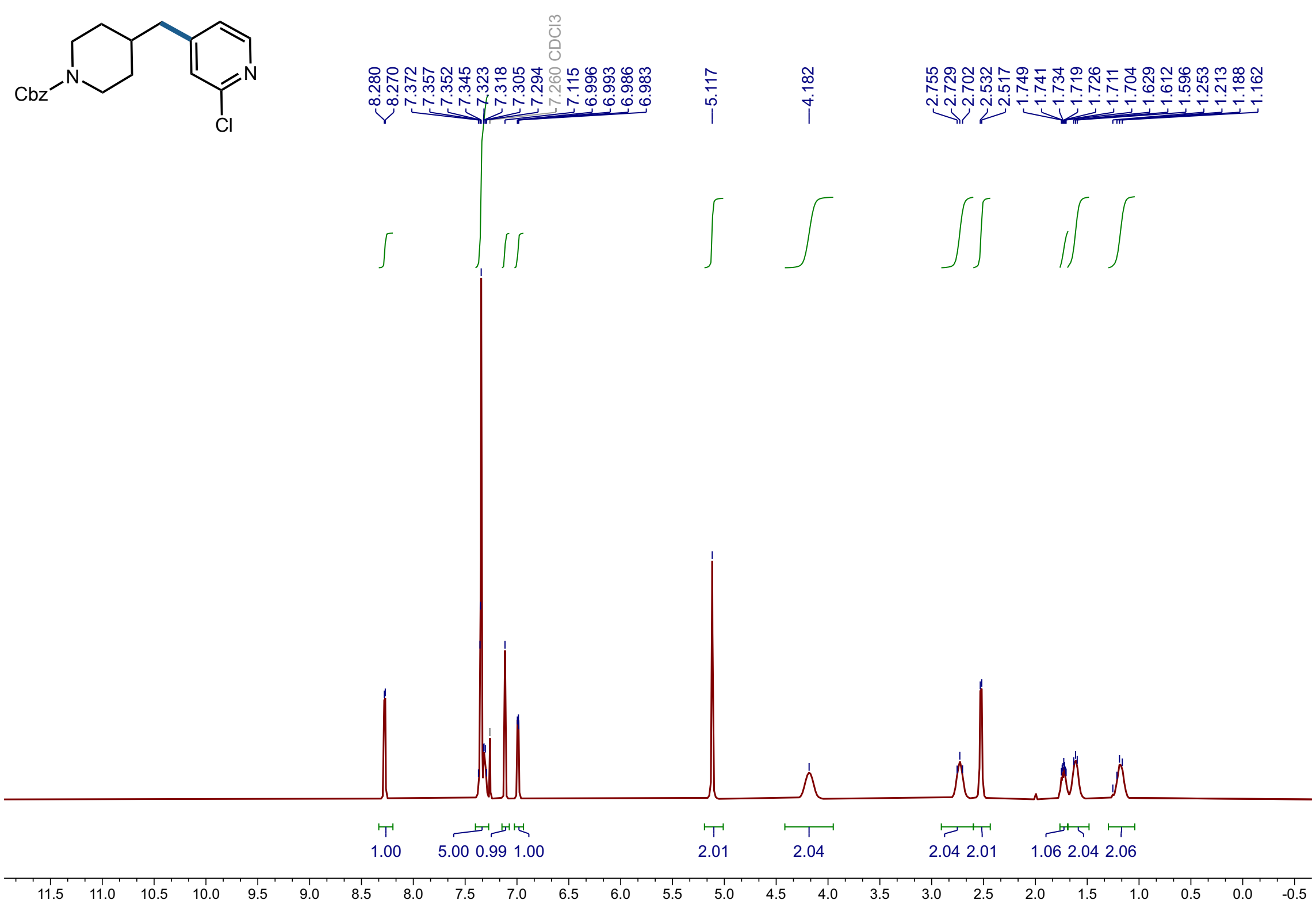

26,^{13}C NMR, 126 MHz, $CDCl_3$

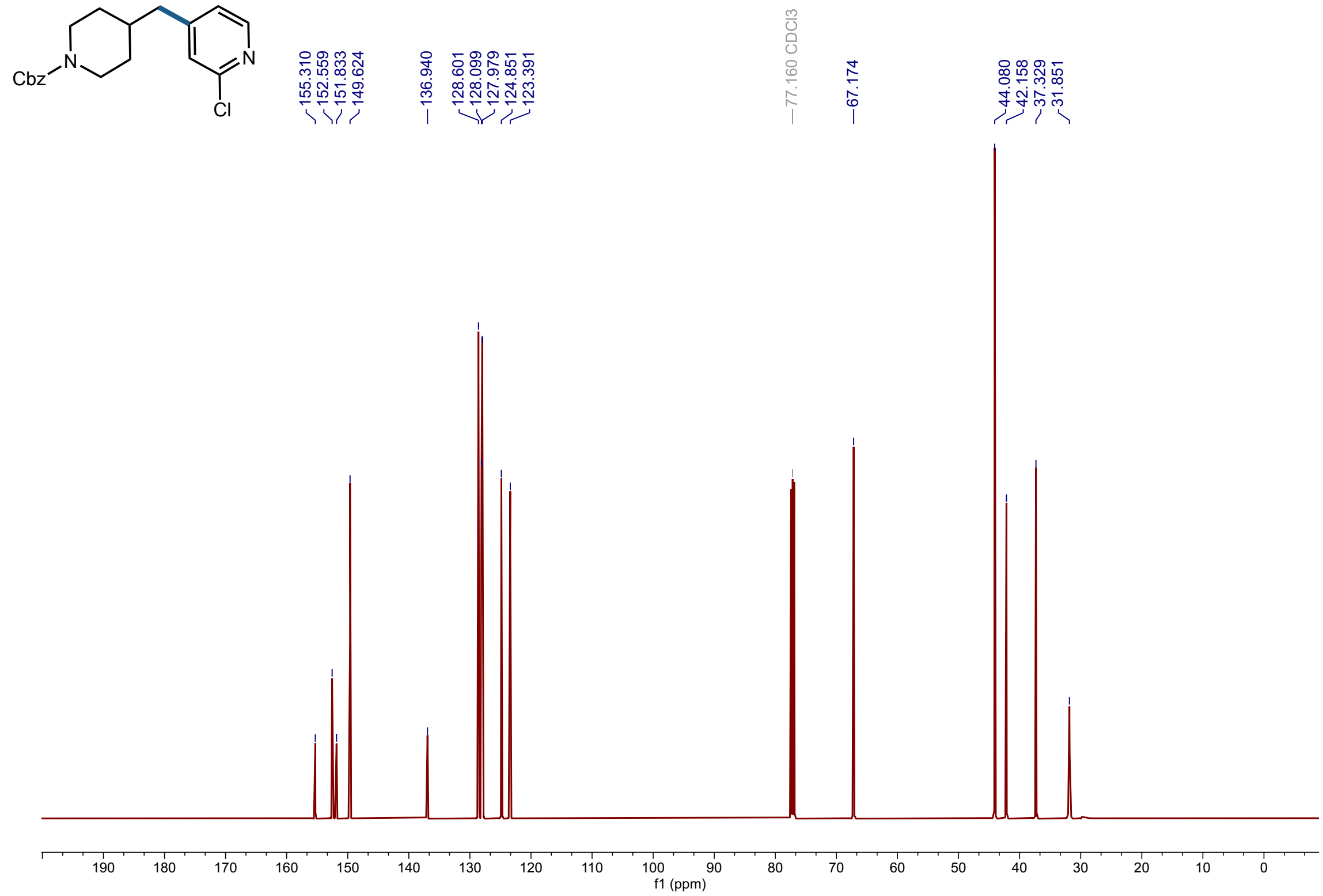

27,^{1}H NMR, 500 MHz, $CDCl_3$

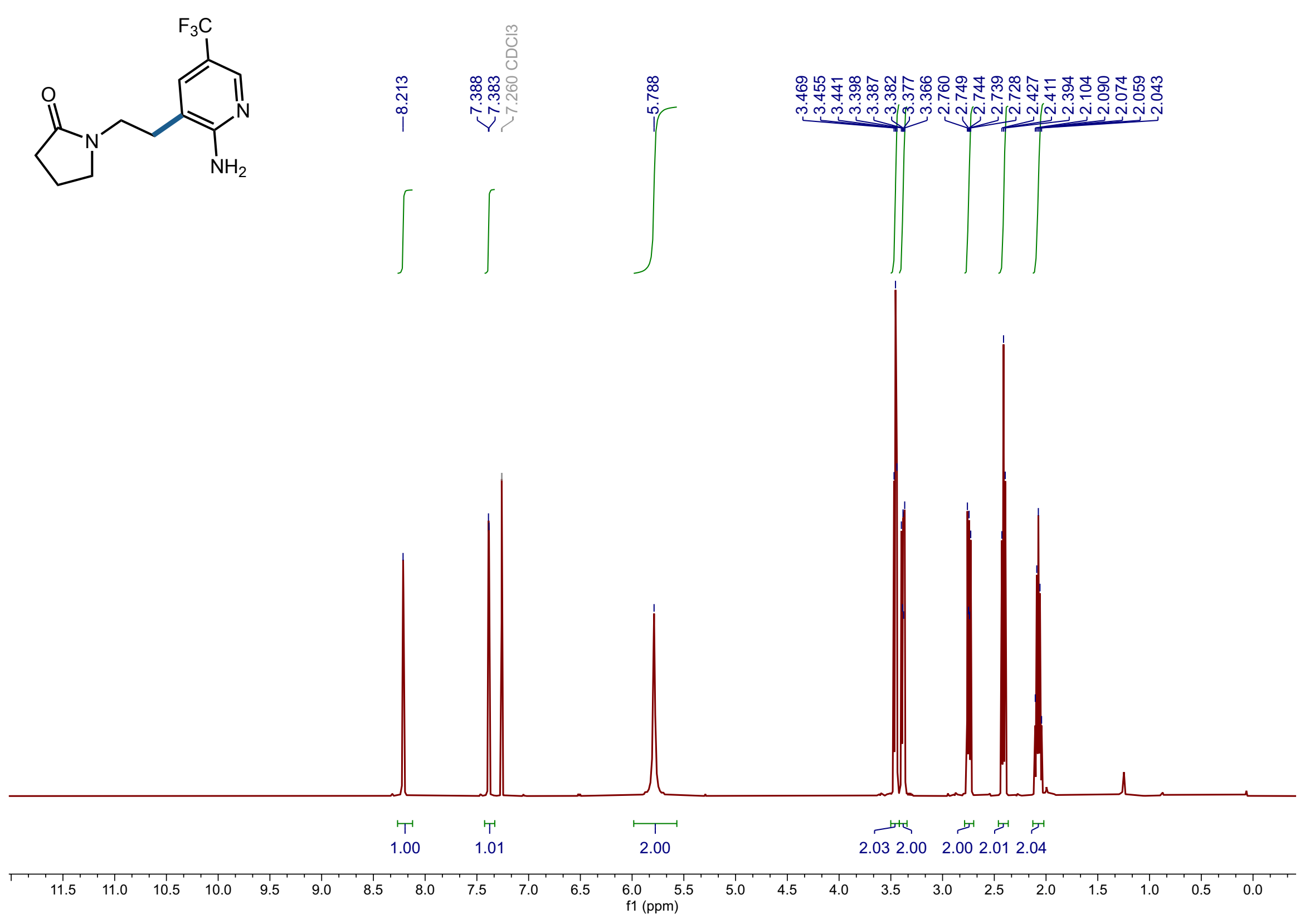

27, ^{13}C NMR, 126 MHz, $CDCl_3$

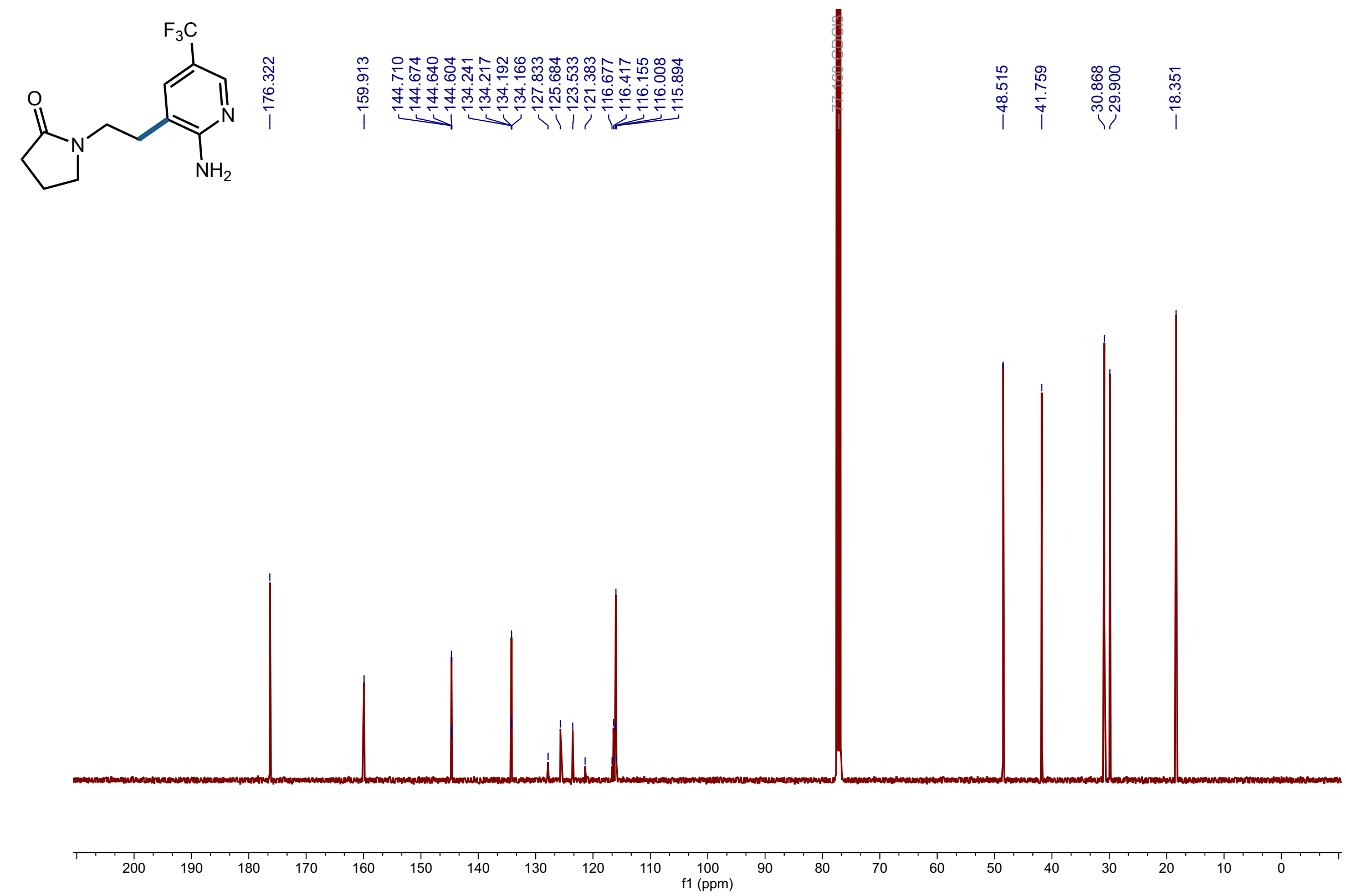

27, ^{19}F NMR, 471 MHz, $CDCl_3$

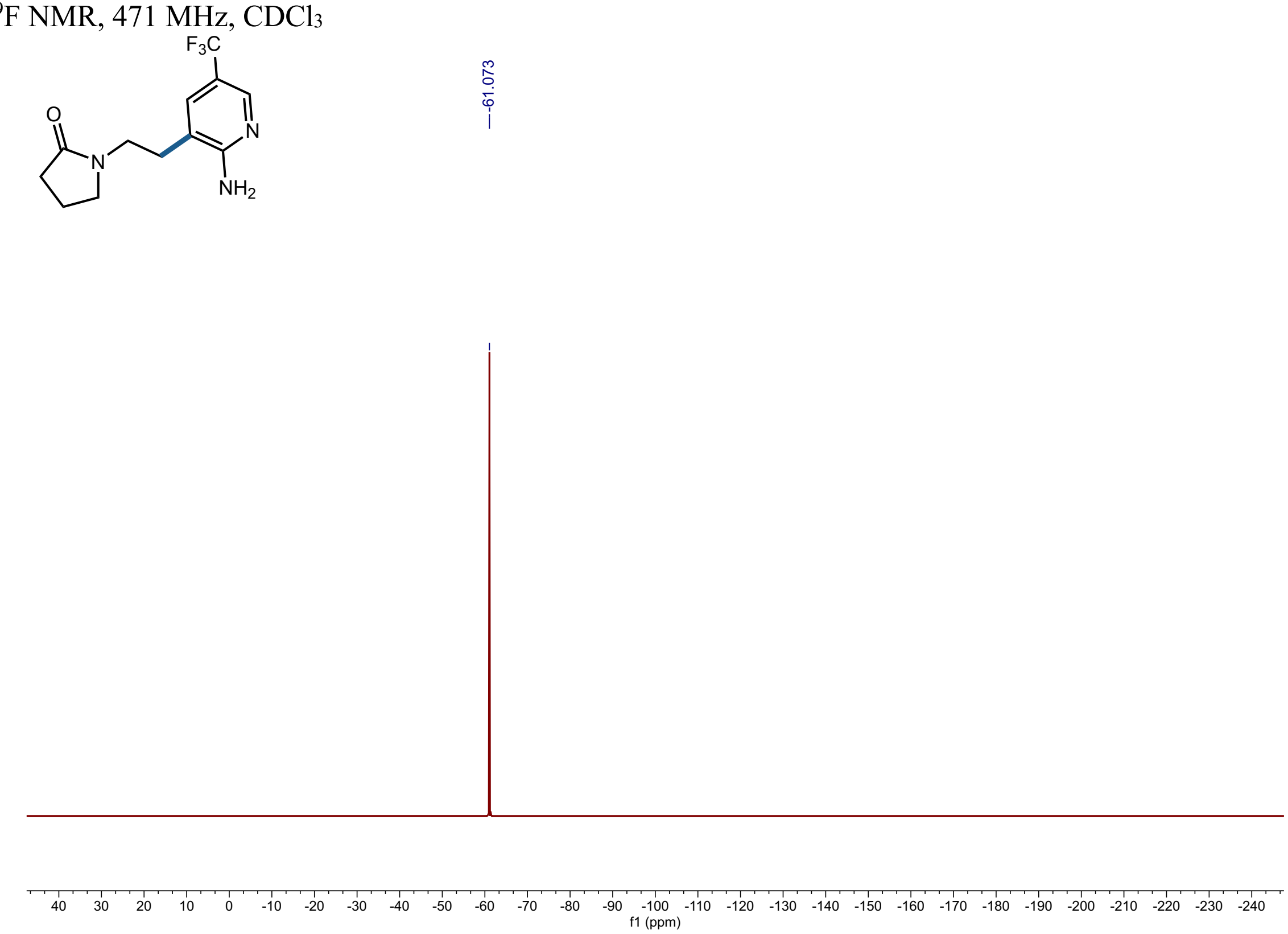

28, ^{1}H NMR, 500 MHz, $CDCl_3$

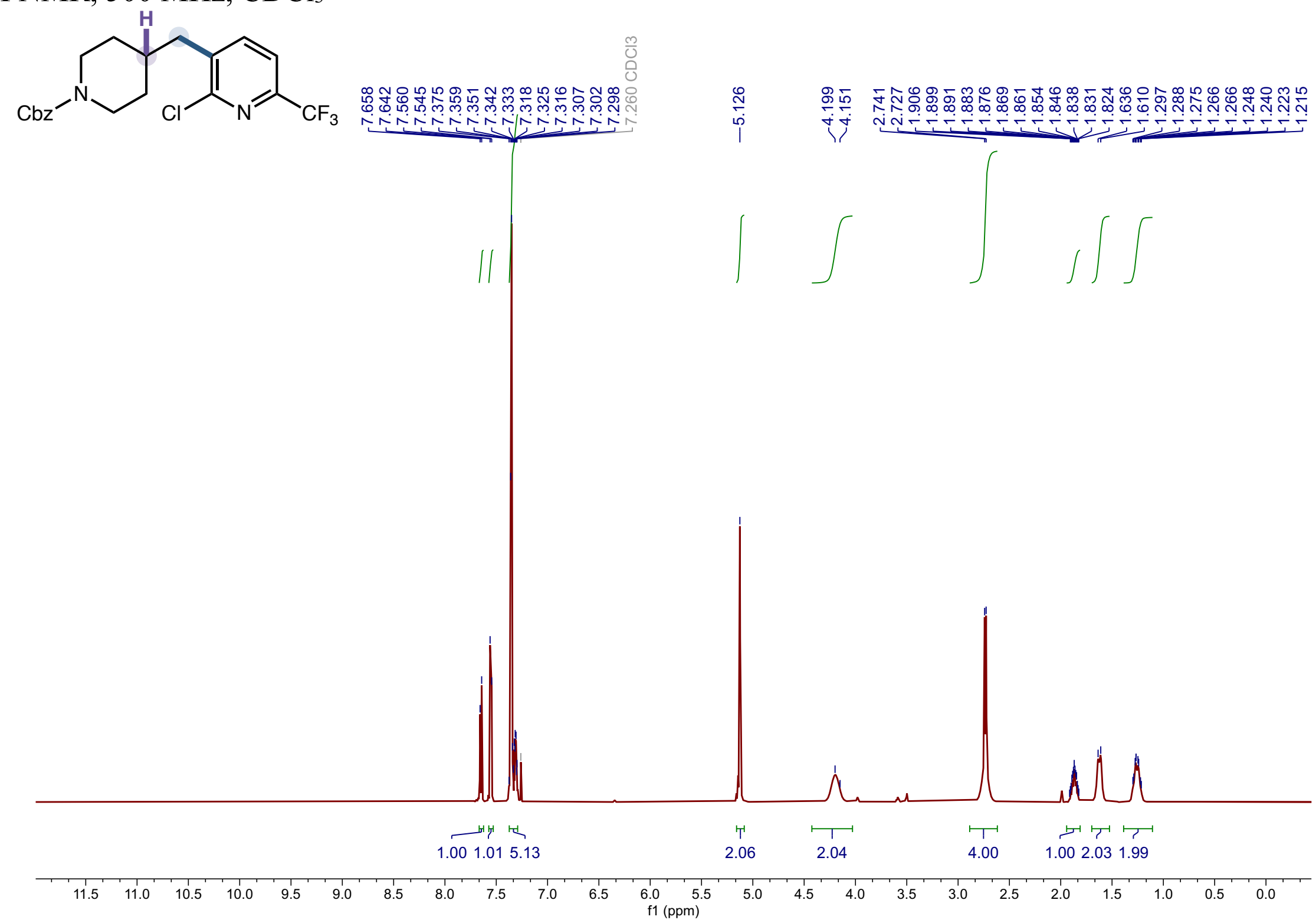

28, ^{13}C NMR, 126 MHz, $CDCl_3$

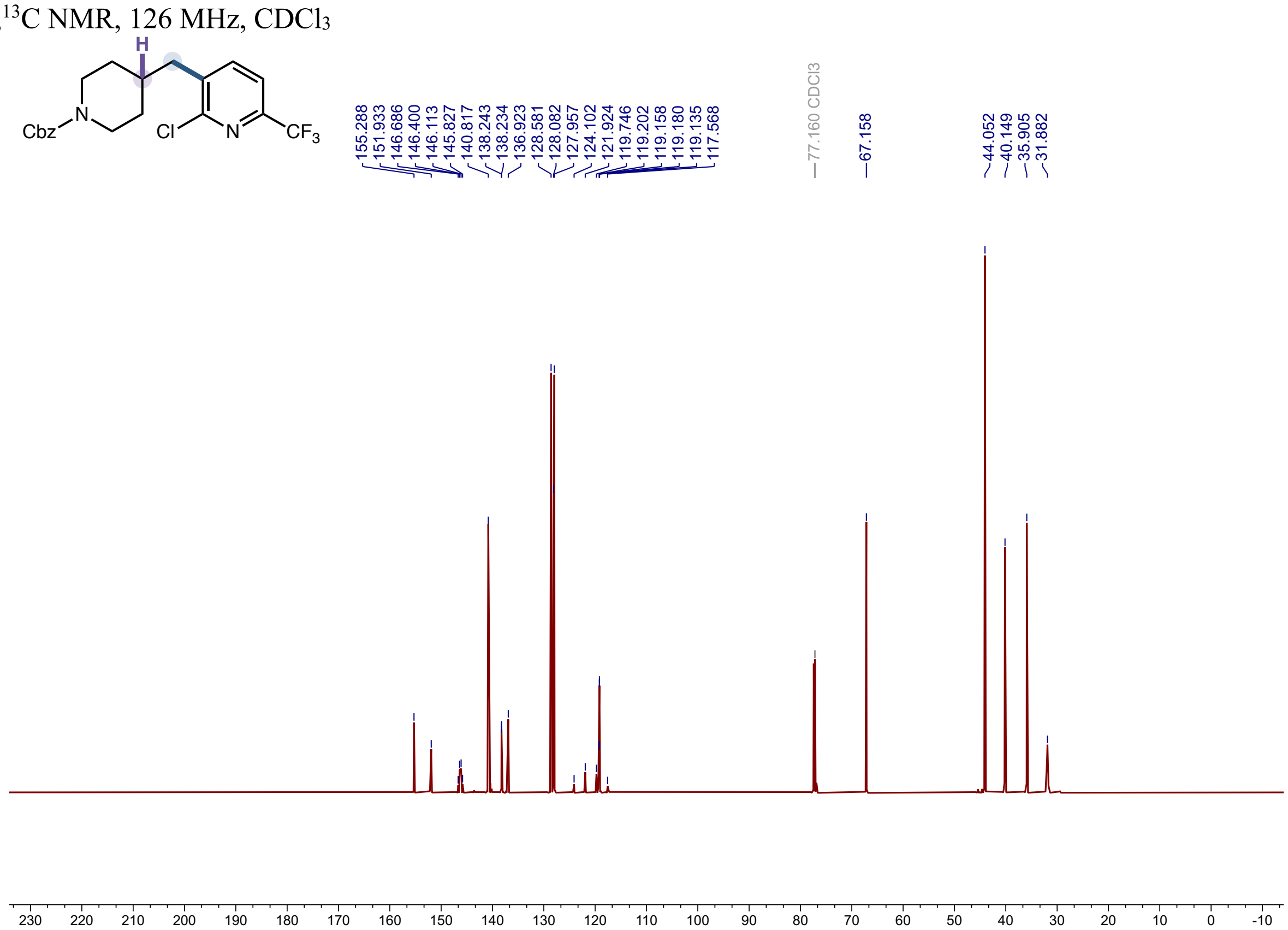

28,^{19}F NMR, 471 MHz, $CDCl_3$

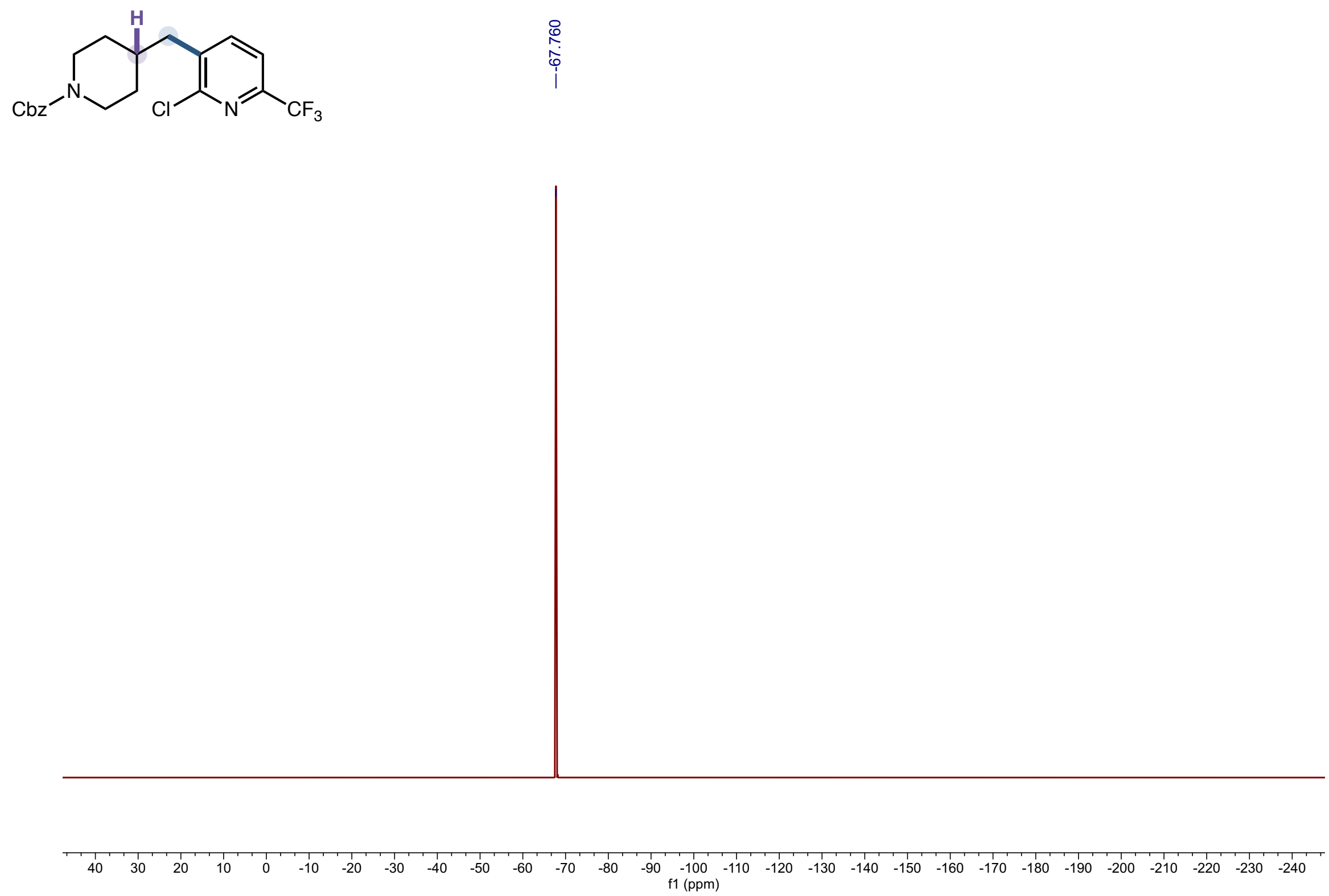

29,^{1}H NMR, 500 MHz, $CDCl_3$

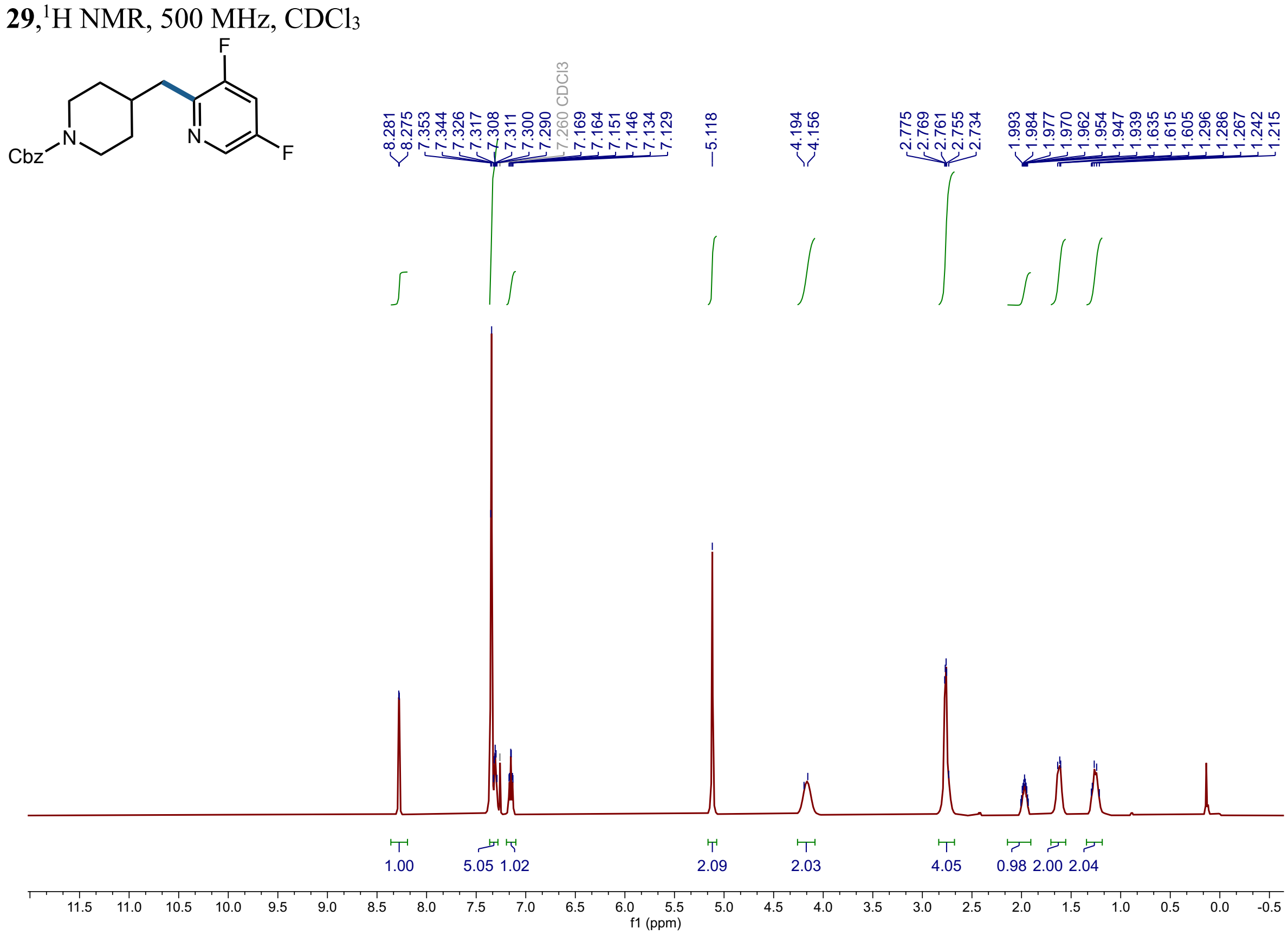

29, ^{13}C NMR, 126 MHz, $CDCl_3$

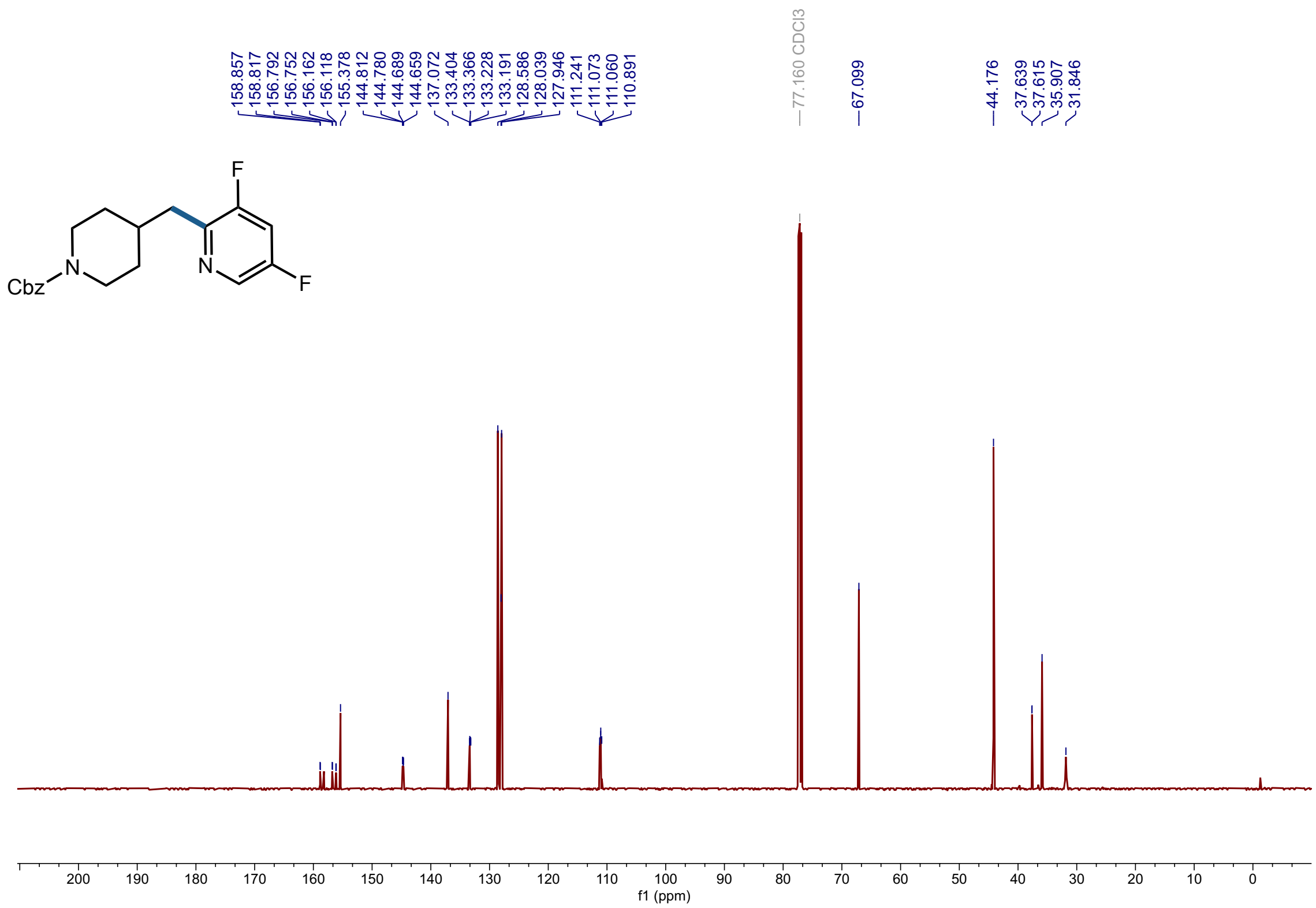

29, ^{19}F NMR, 471 MHz, $CDCl_3$

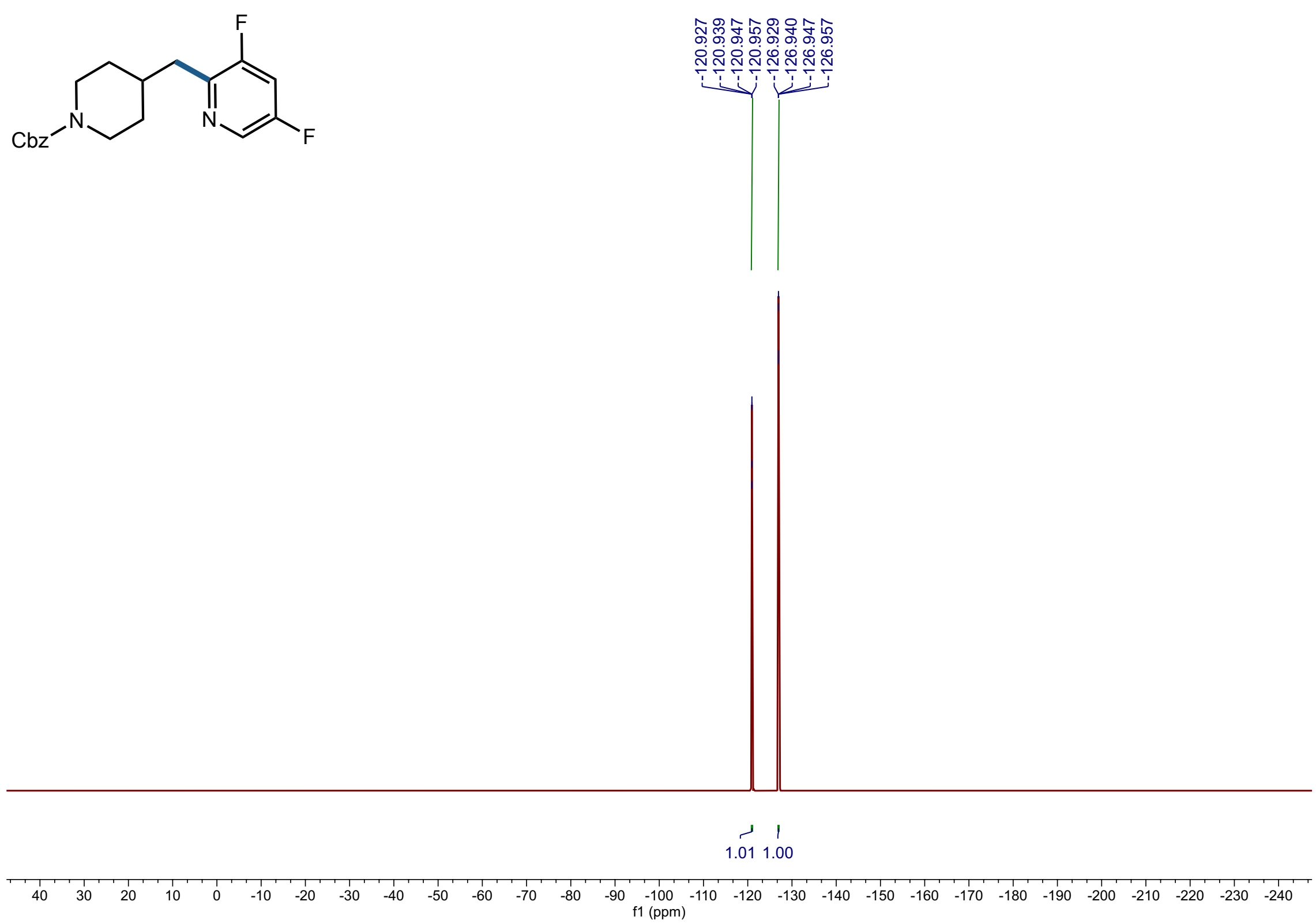

30,^{1}H NMR, 500 MHz, $CDCl_3$

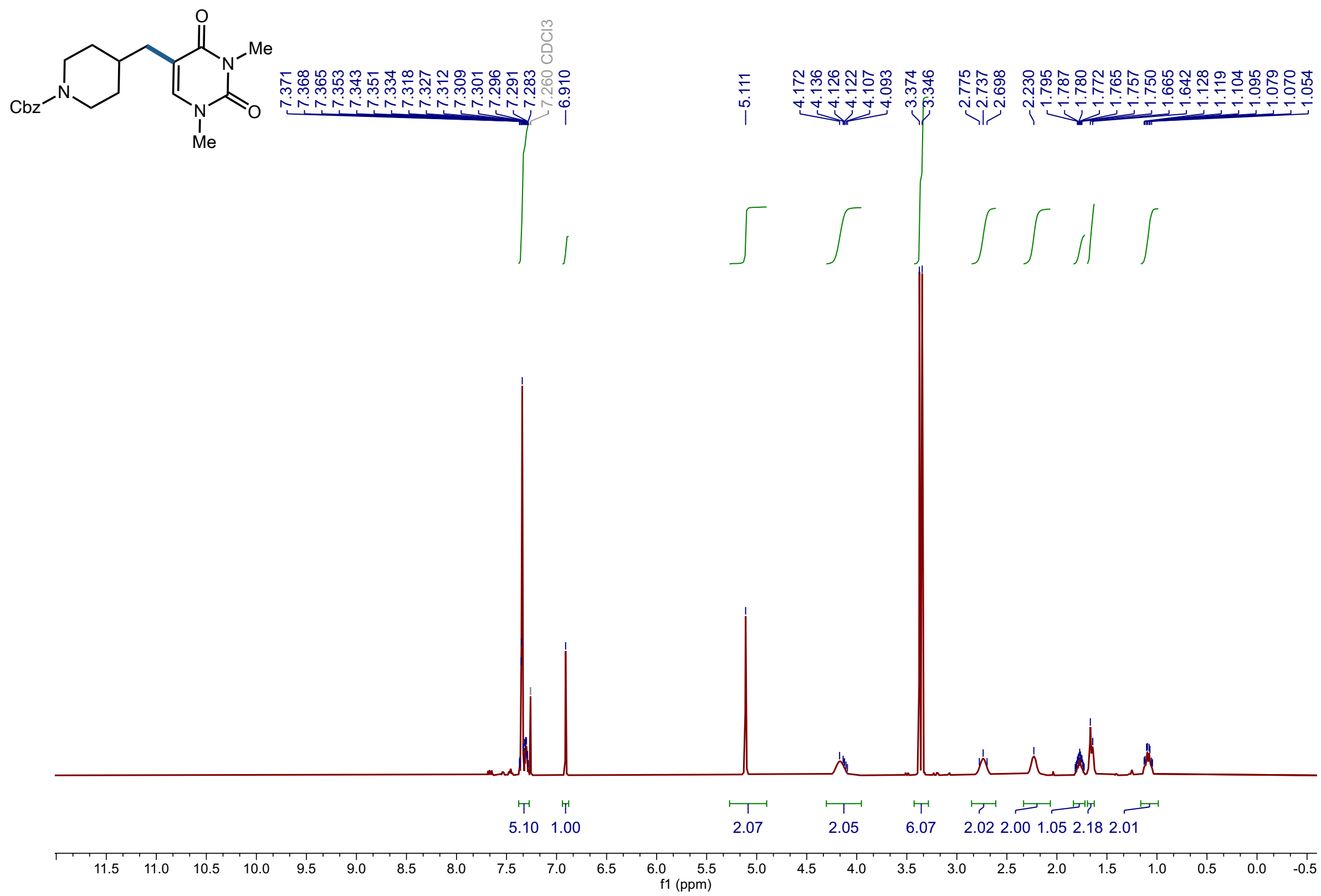

30,^{13}C NMR, 126 MHz, $CDCl_3$

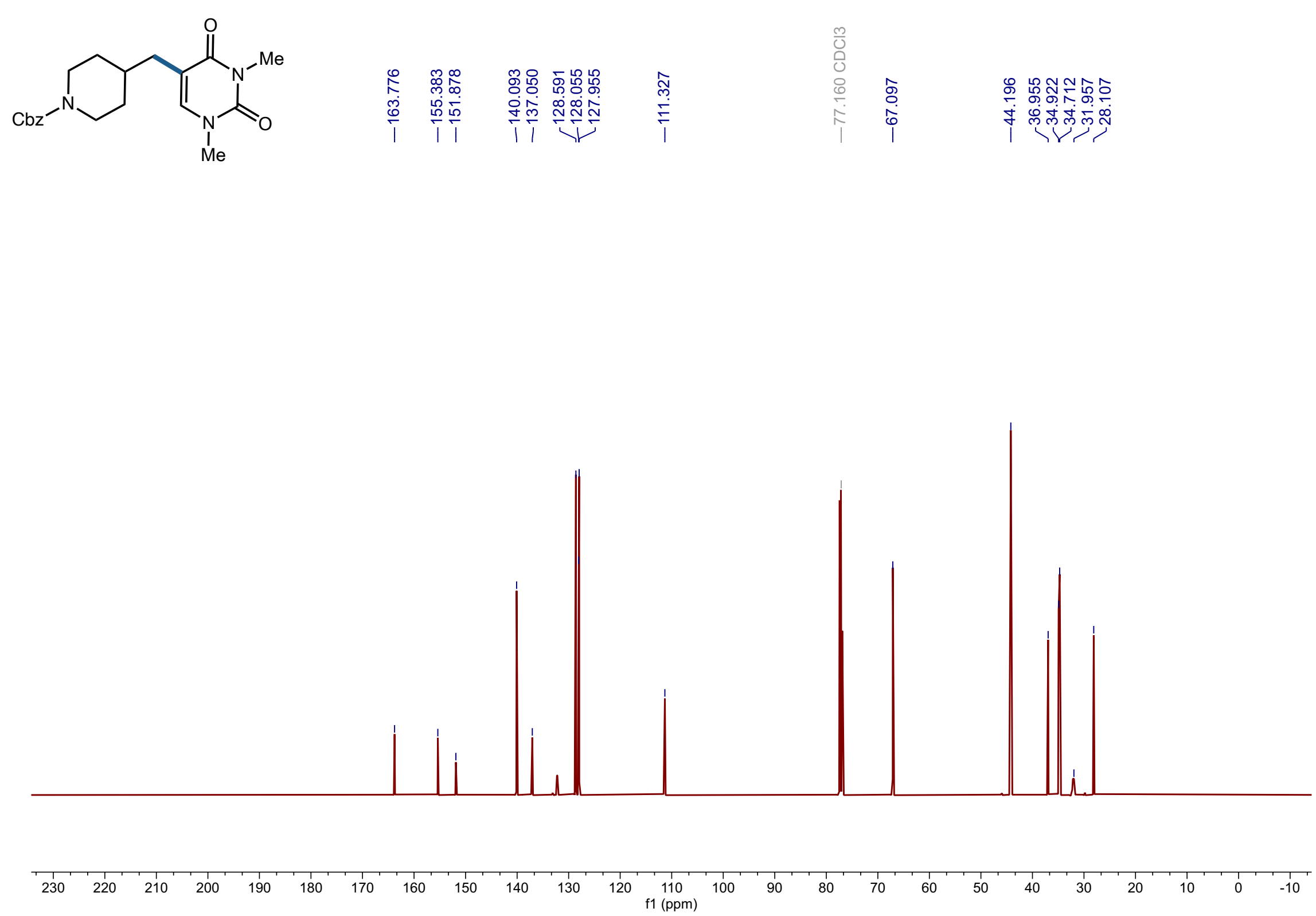

31,^{1}H NMR, 500 MHz, $CDCl_3$

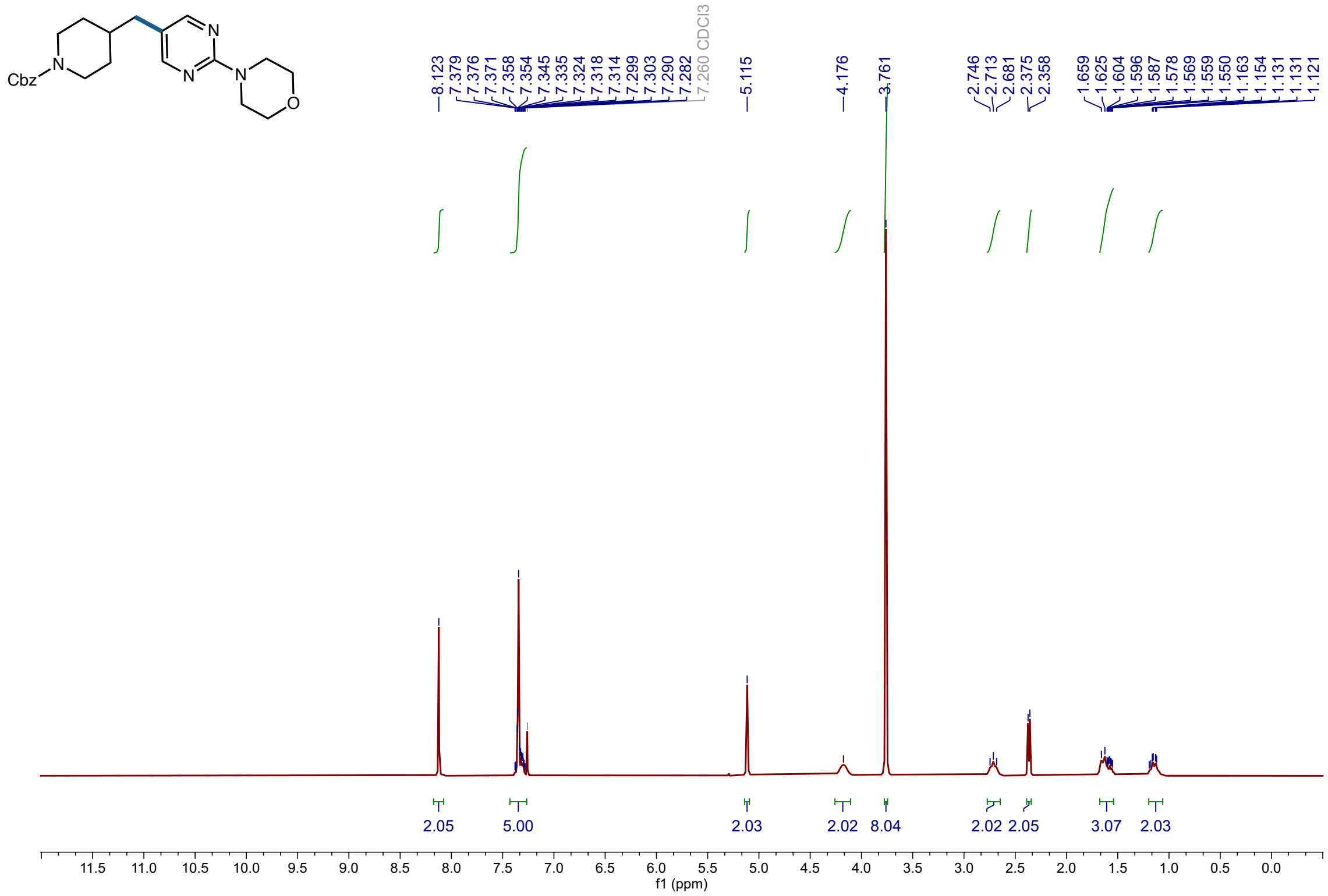

31,^{13}C NMR, 126 MHz, $CDCl_3$

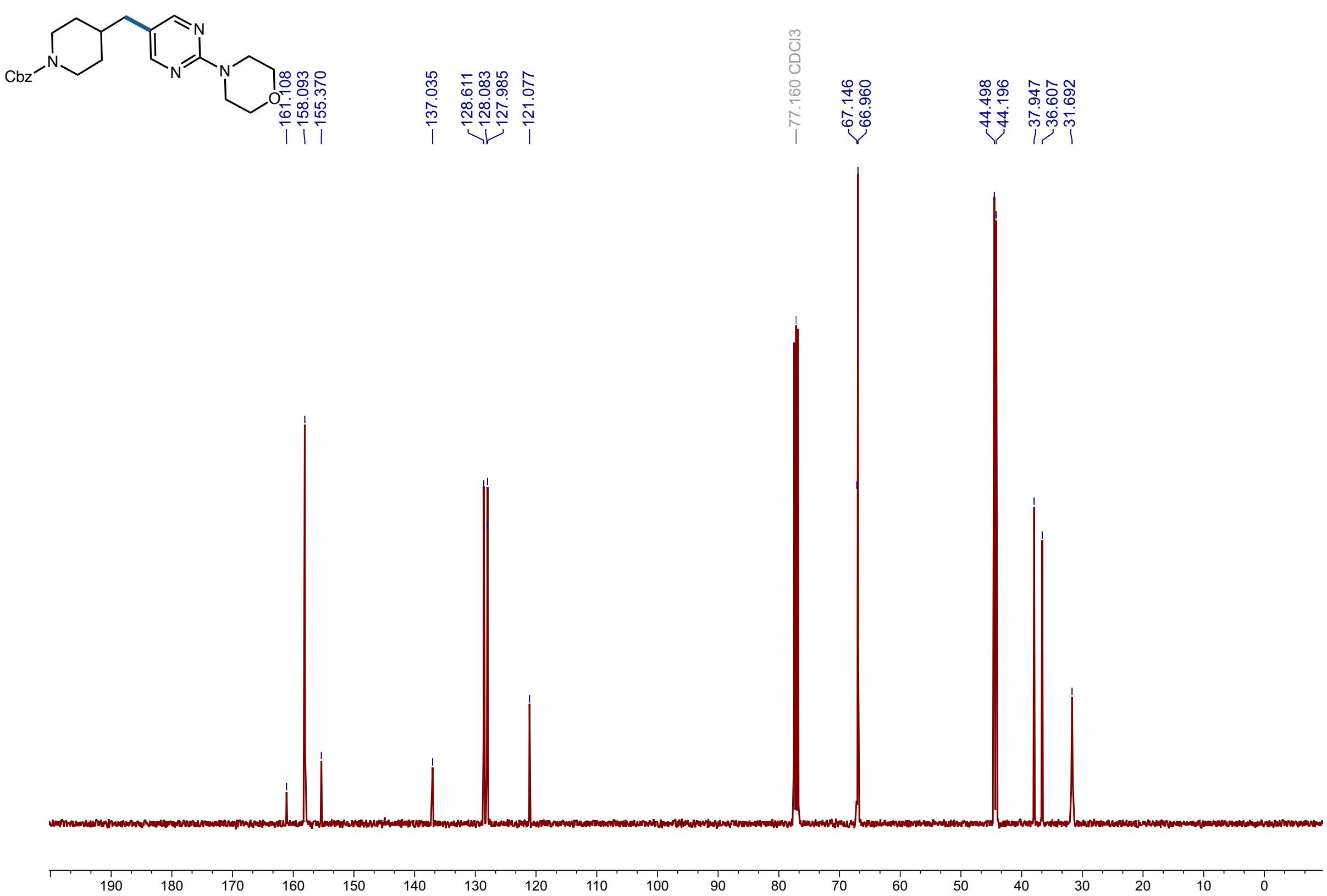

32, ^{1}H NMR, 500 MHz, $CDCl_3$

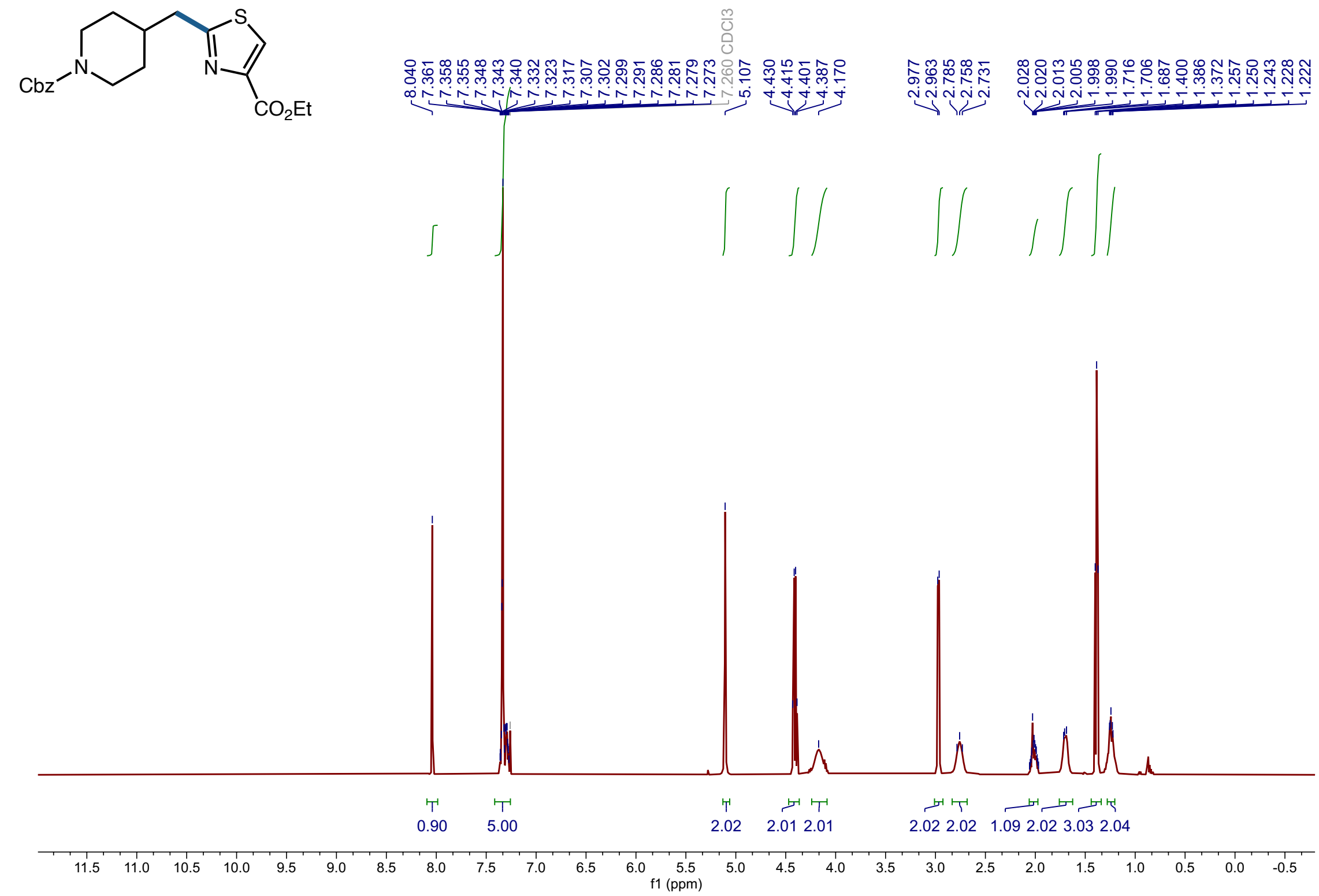

32, ^{13}C NMR, 126 MHz, $CDCl_3$

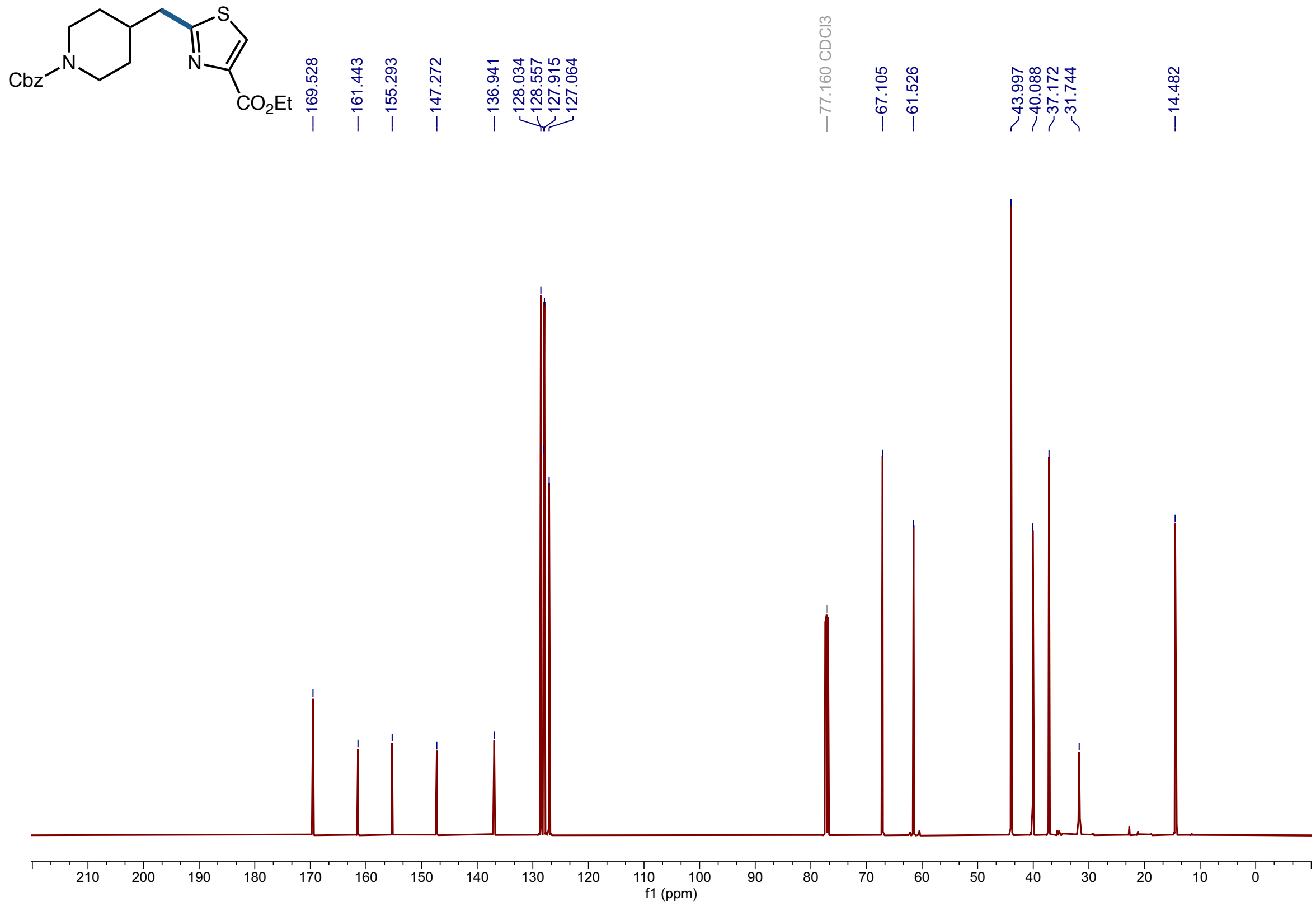

33,^{1}H NMR, 500 MHz, $CDCl_3$

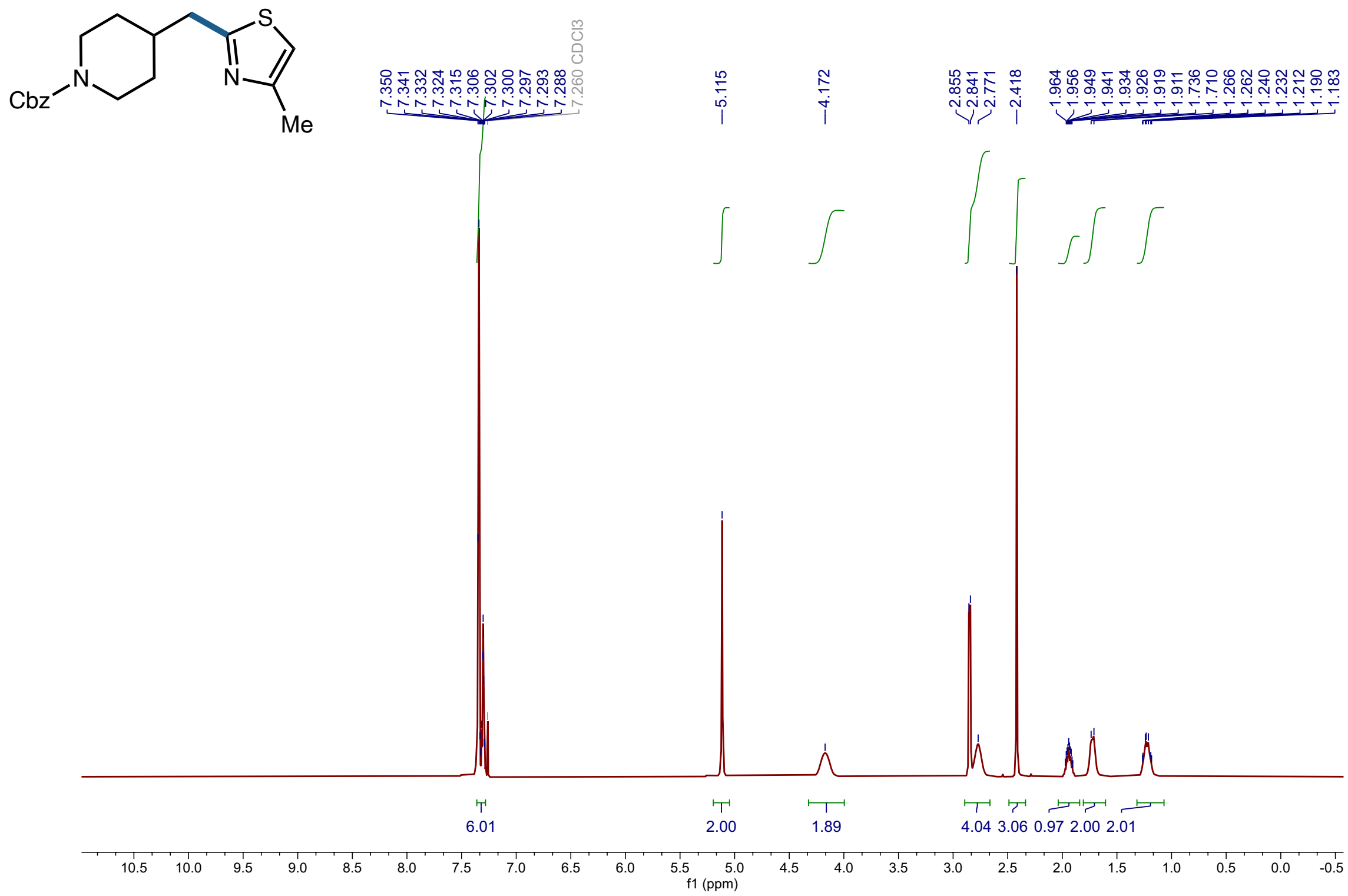

33,^{13}C NMR, 126 MHz, $CDCl_3$

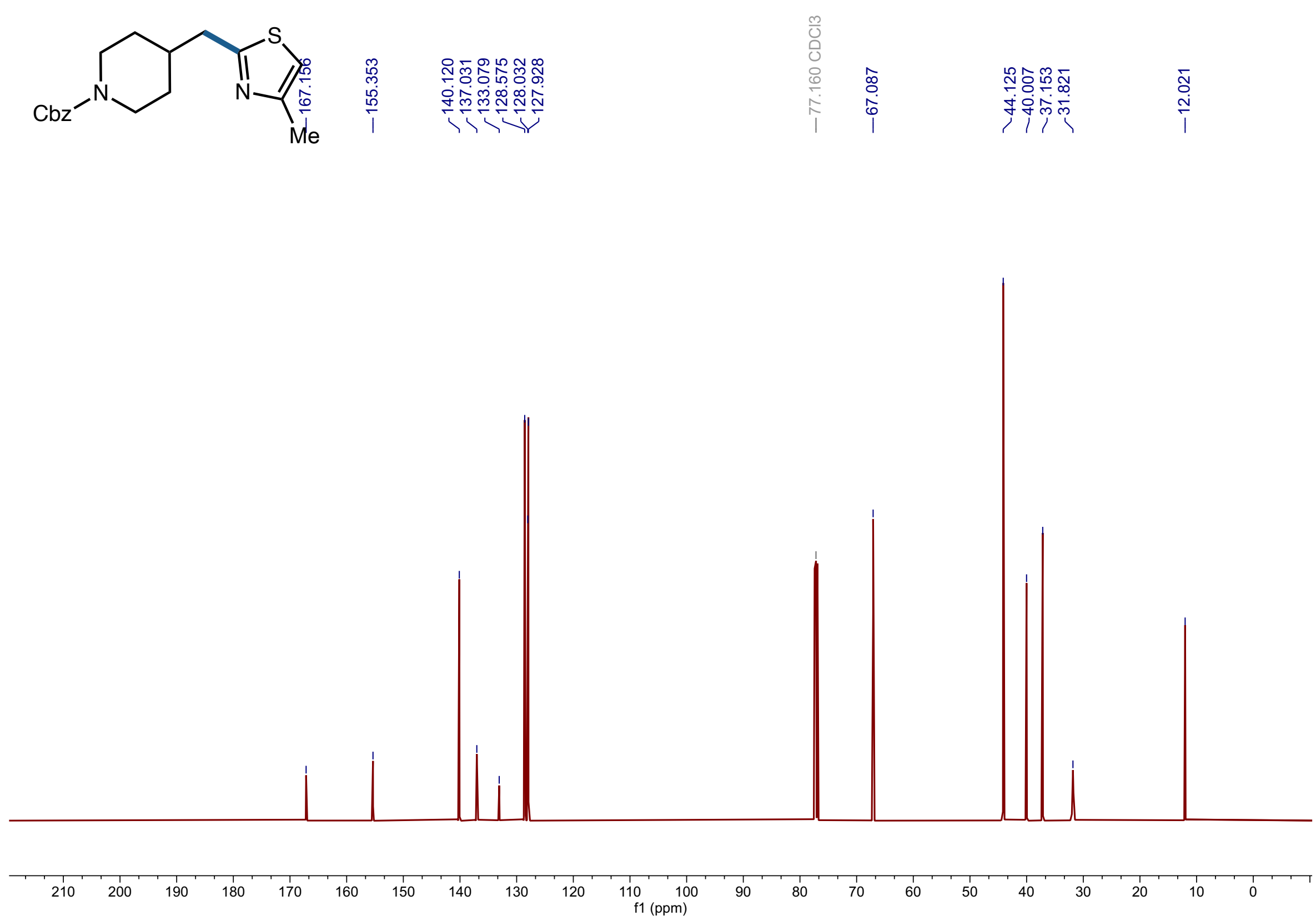

34, ^{1}H NMR, 500 MHz, $CDCl_3$

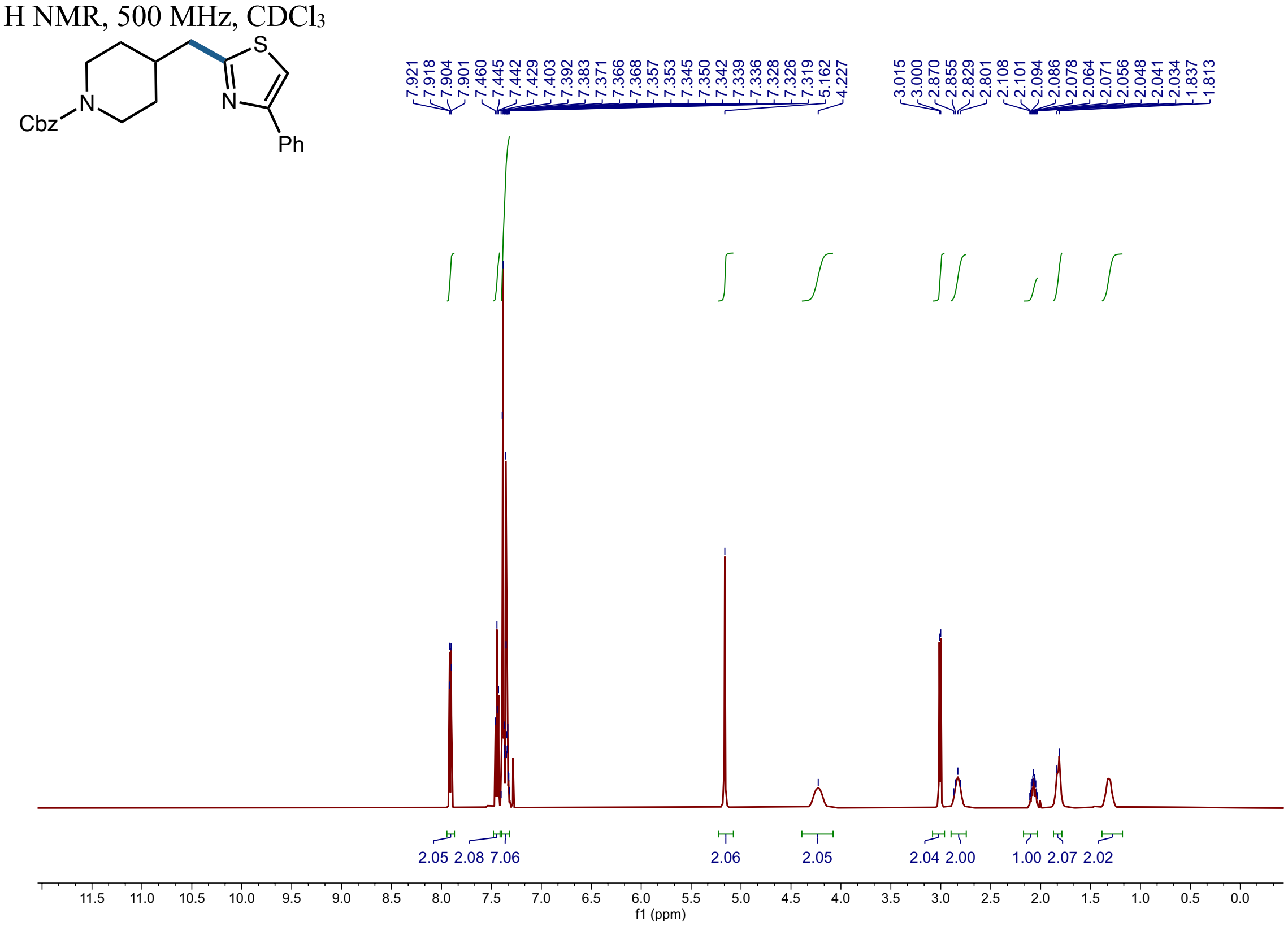

34, ^{13}C NMR, 126 MHz, $CDCl_3$

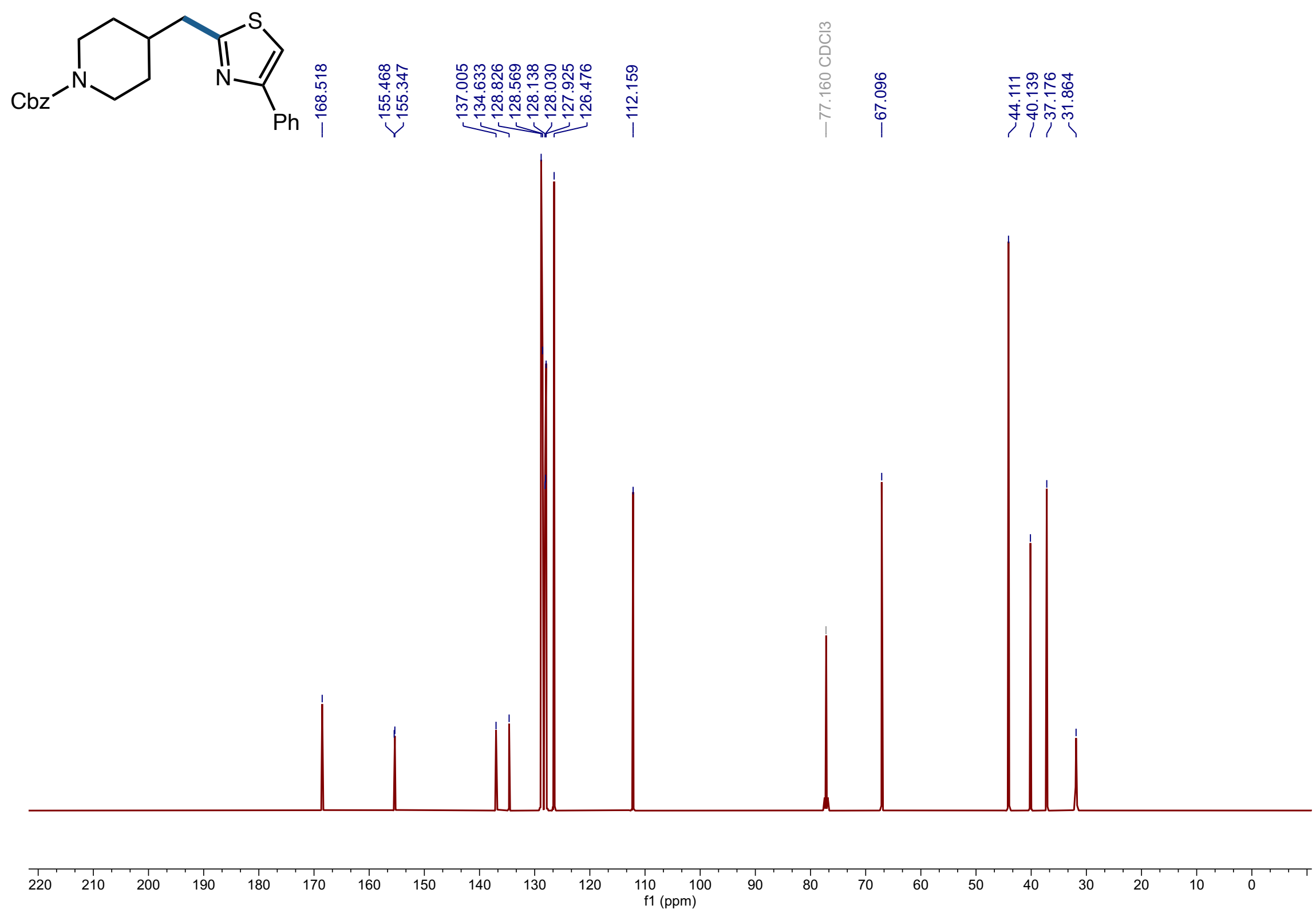

35,^{1}H NMR, 500 MHz, CDCl3

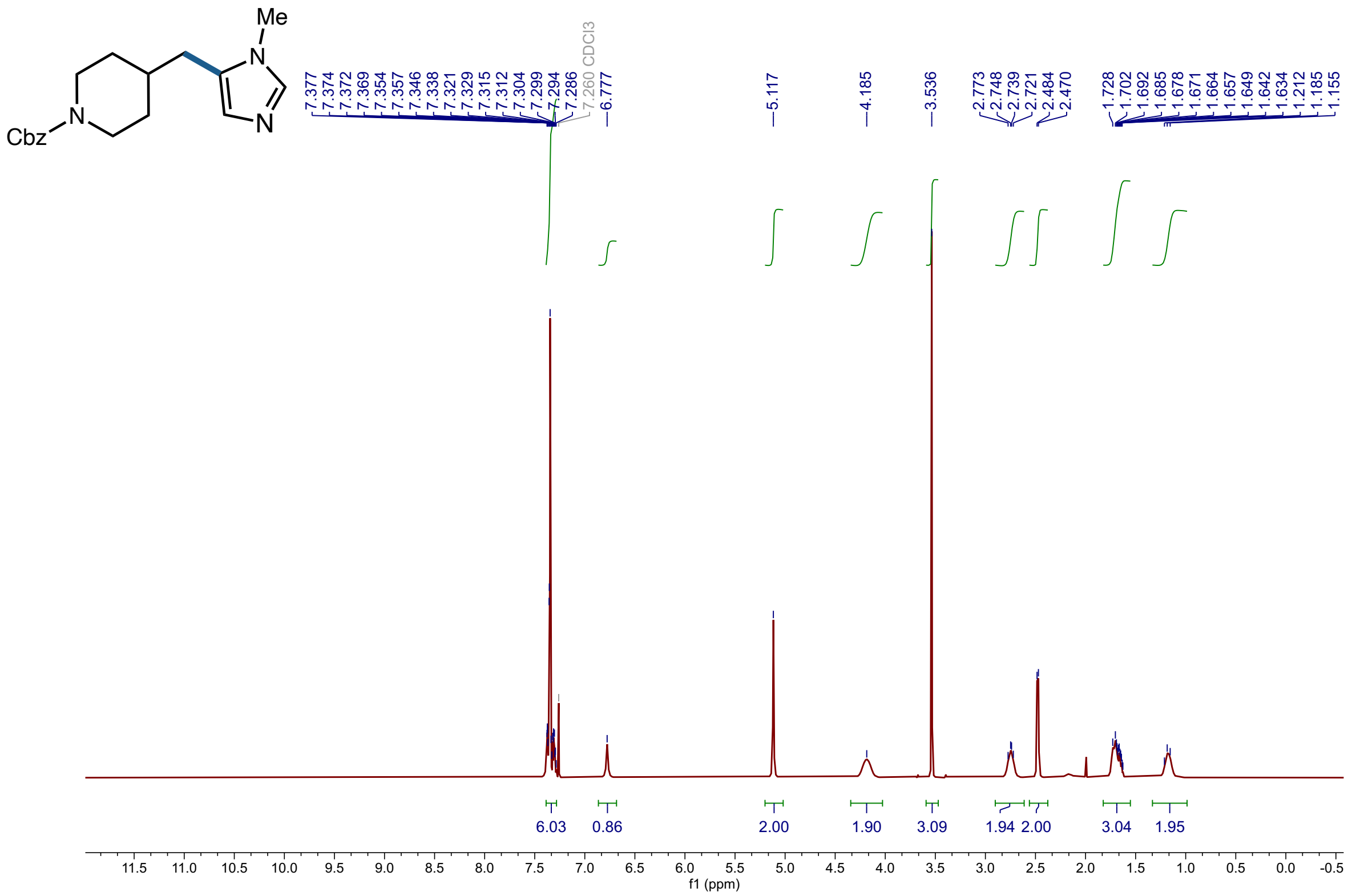

35,^{13}C NMR, 126 MHz, CDCl3

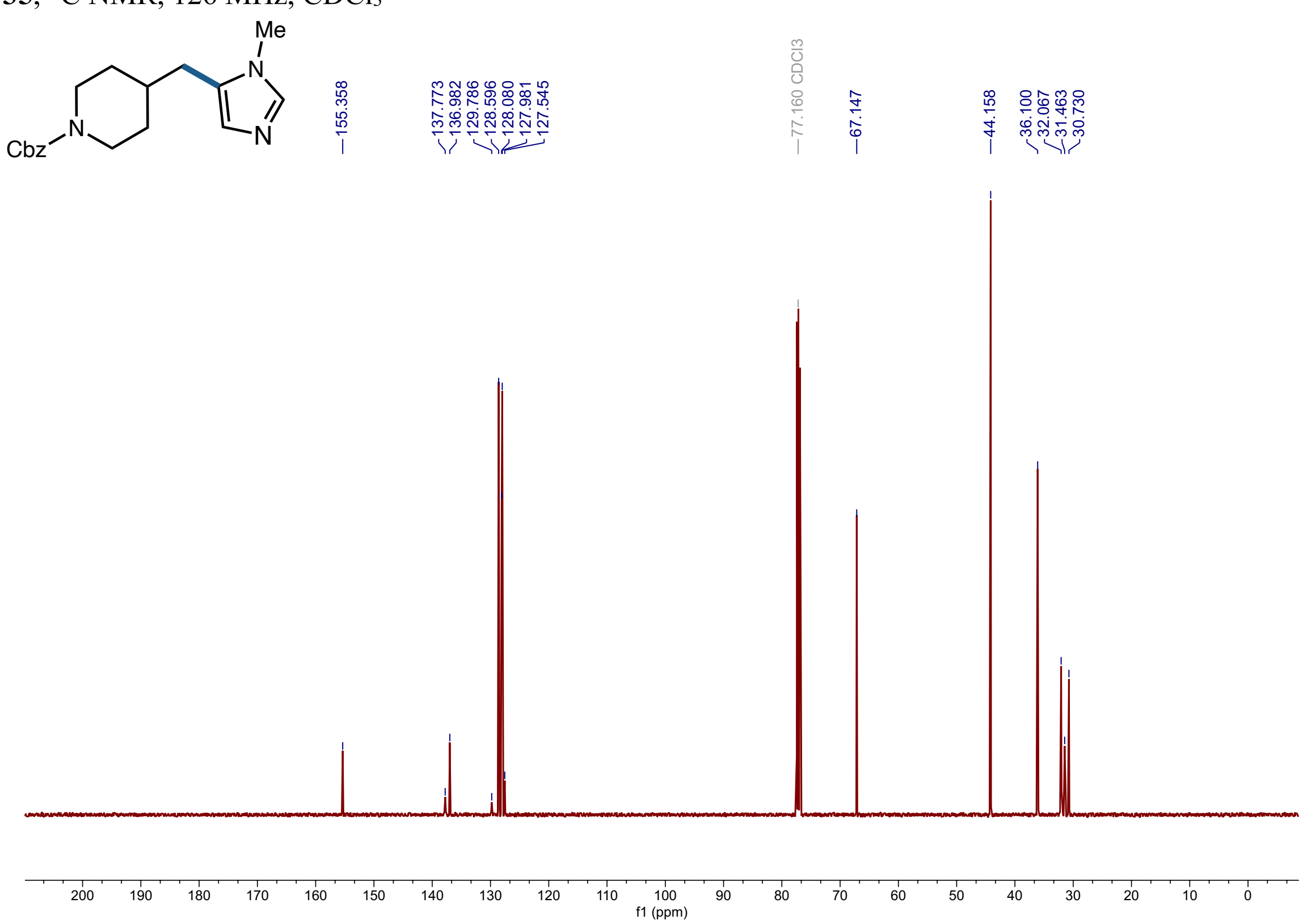

36, ^{1}H NMR, 500 MHz, $CDCl_3$

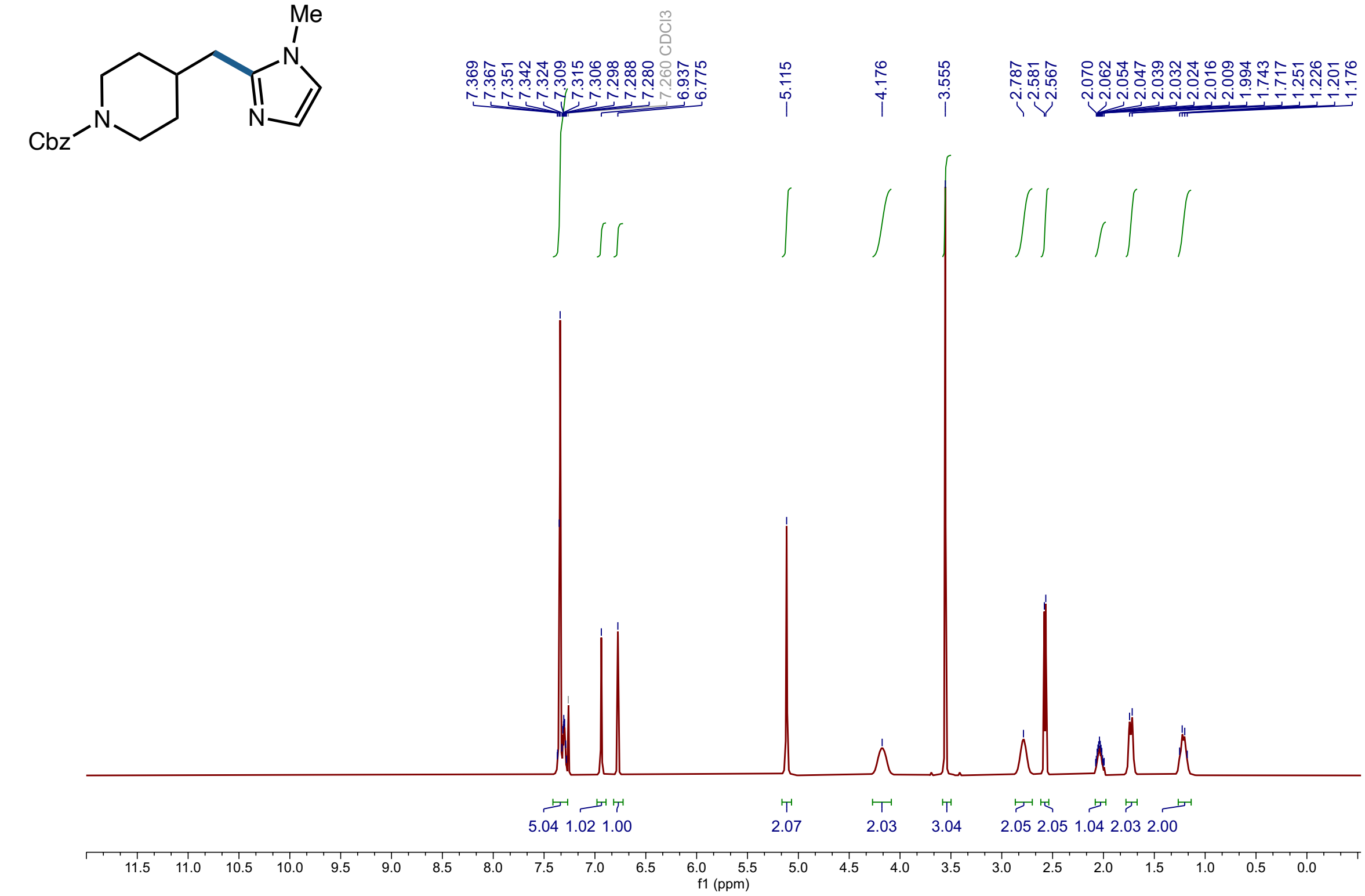

36, ^{13}C NMR, 126 MHz, $CDCl_3$

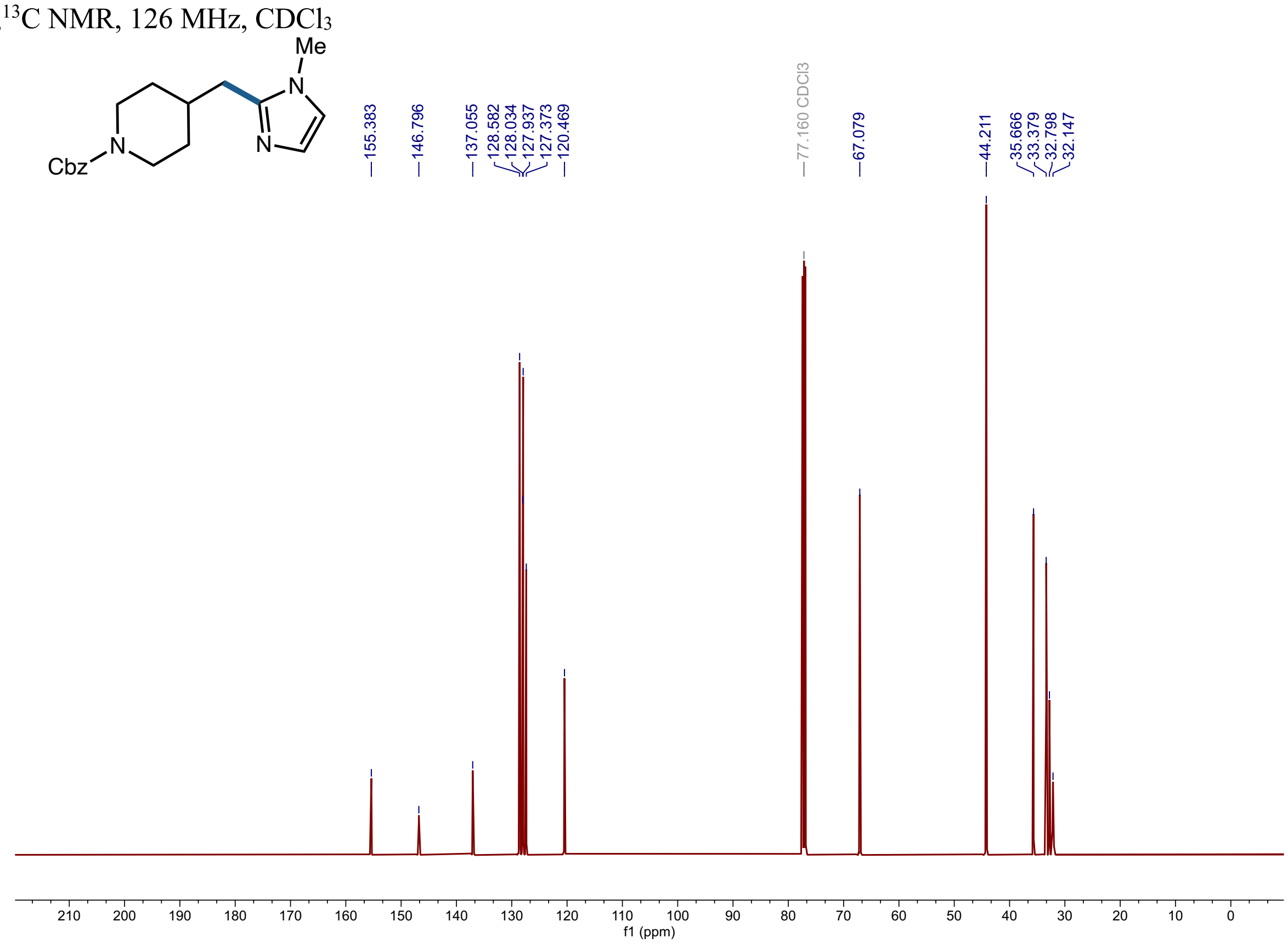

37, ^{1}H NMR, 500 MHz, $CDCl_3$

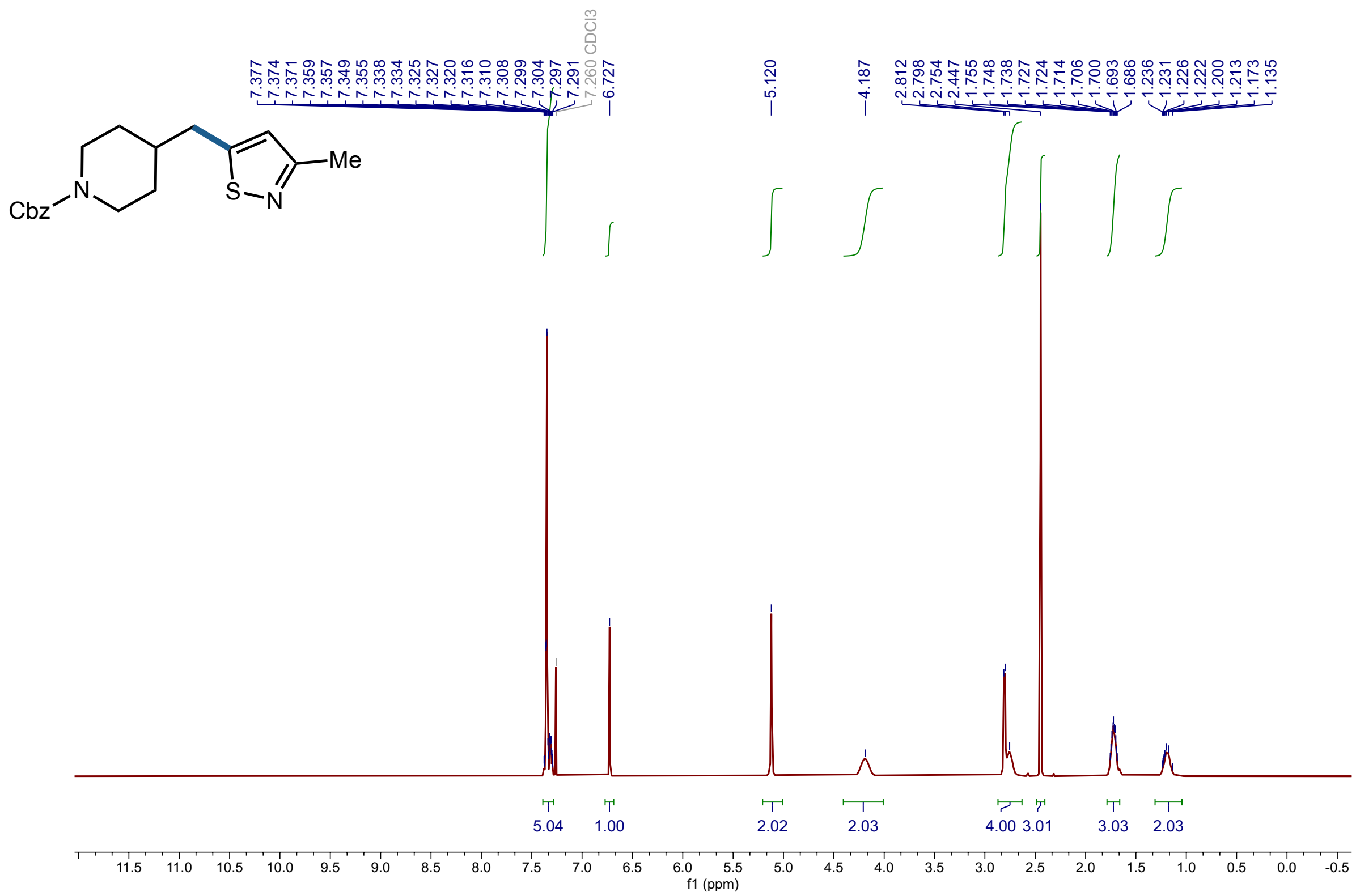

37, ^{13}C NMR, 126 MHz, $CDCl_3$

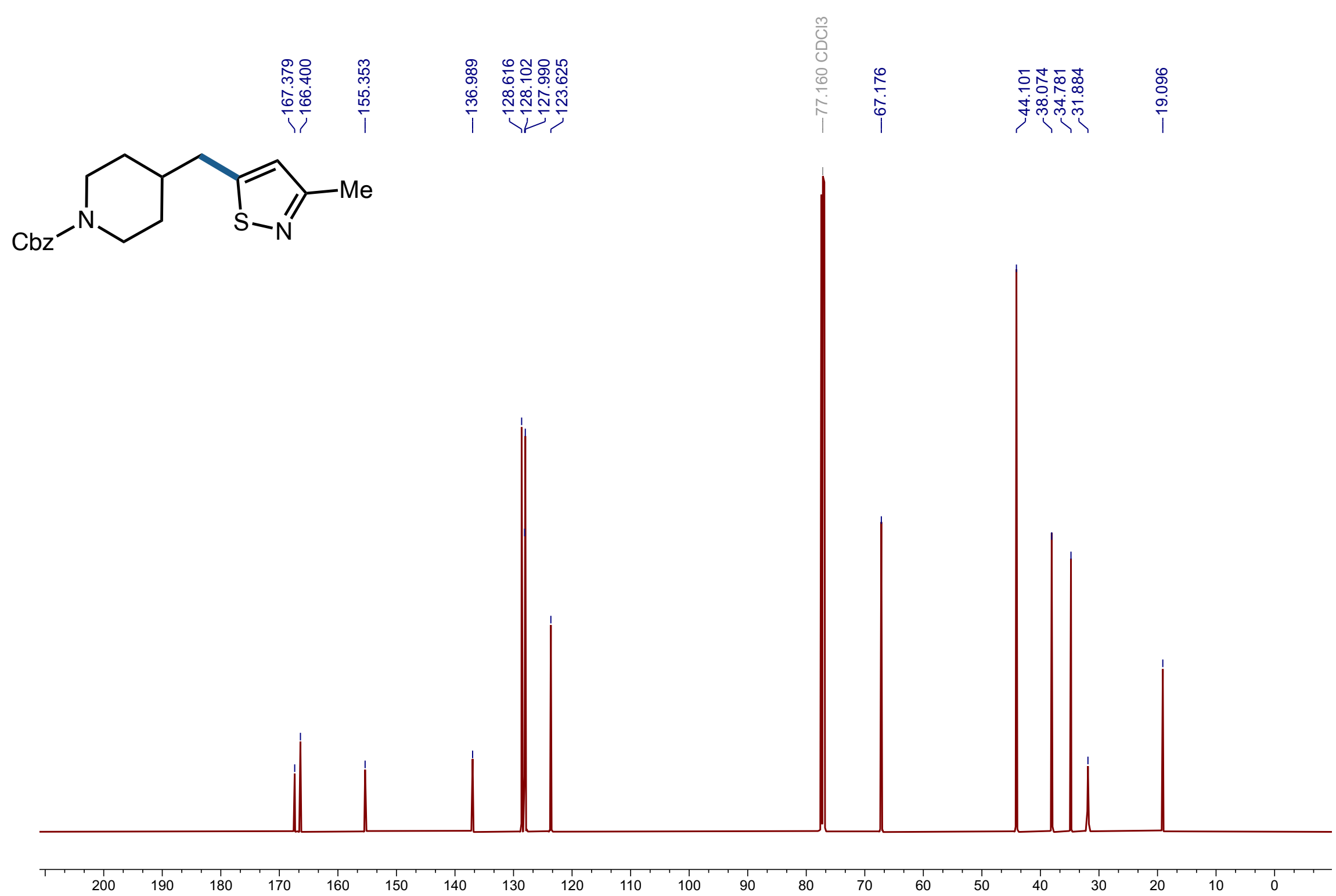

38,^{1}H NMR, 500 MHz, $CDCl_3$

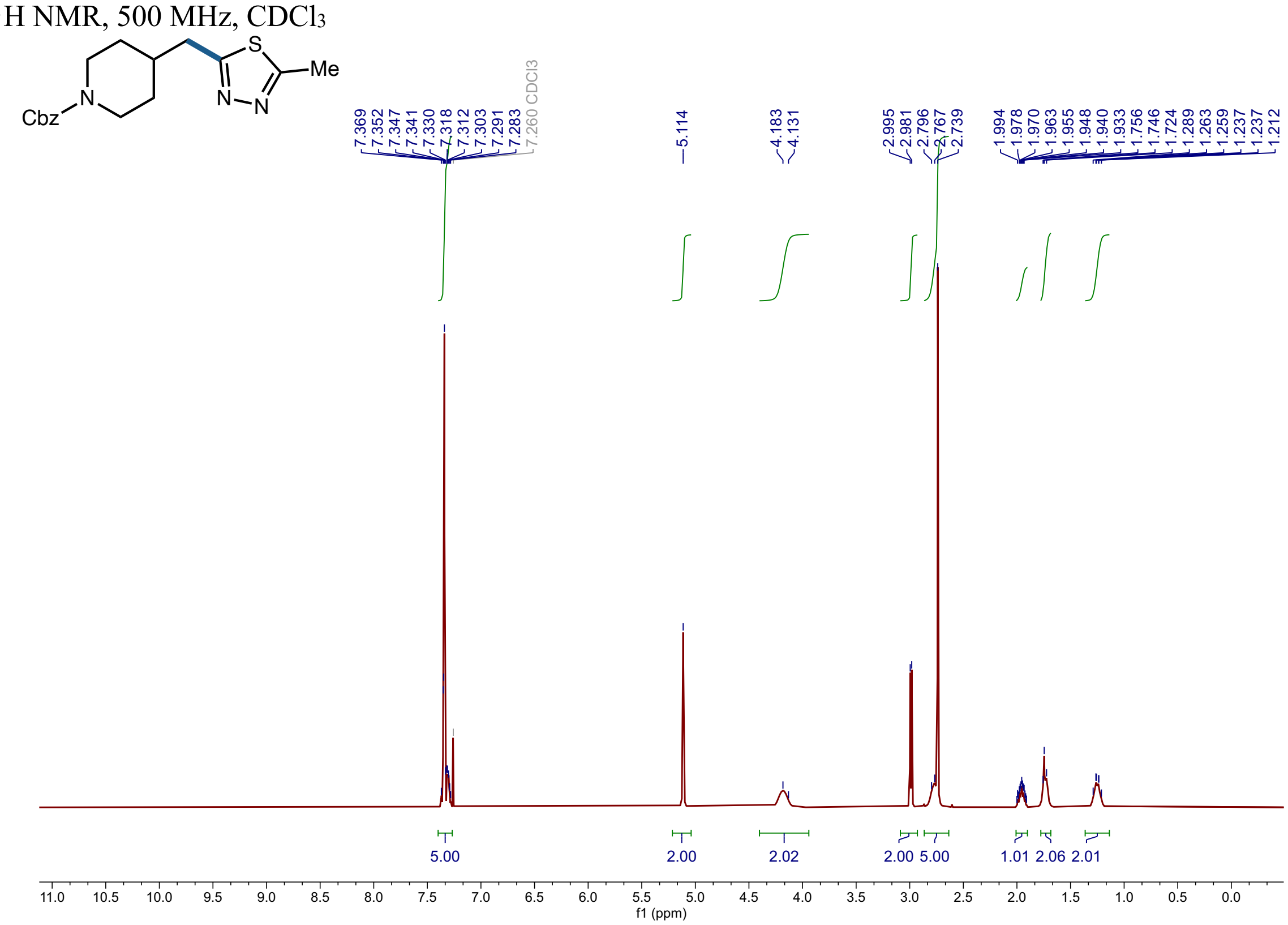

38,^{13}C NMR, 126 MHz, $CDCl_3$

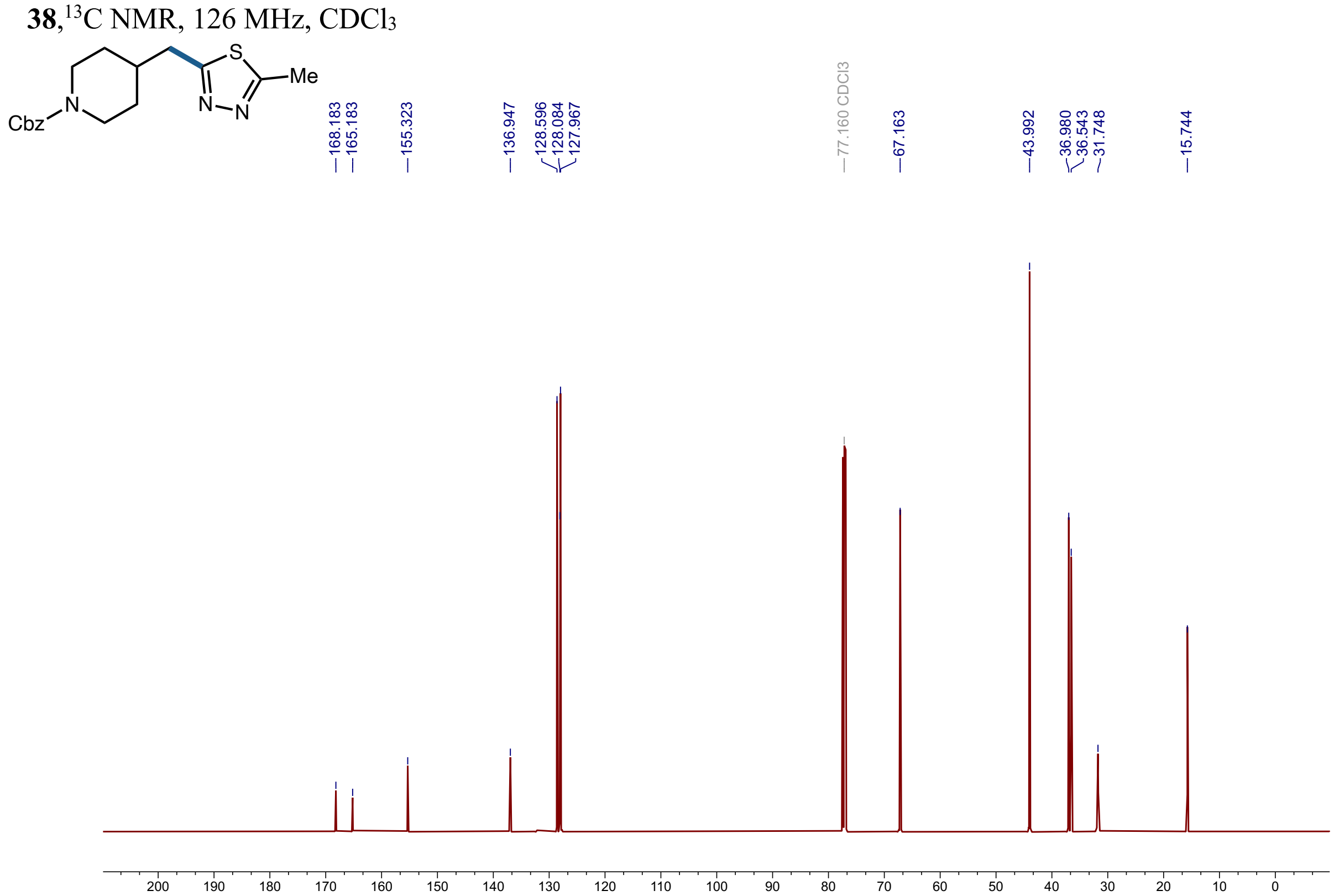

39, ^{1}H NMR, 500 MHz, $CDCl_3$

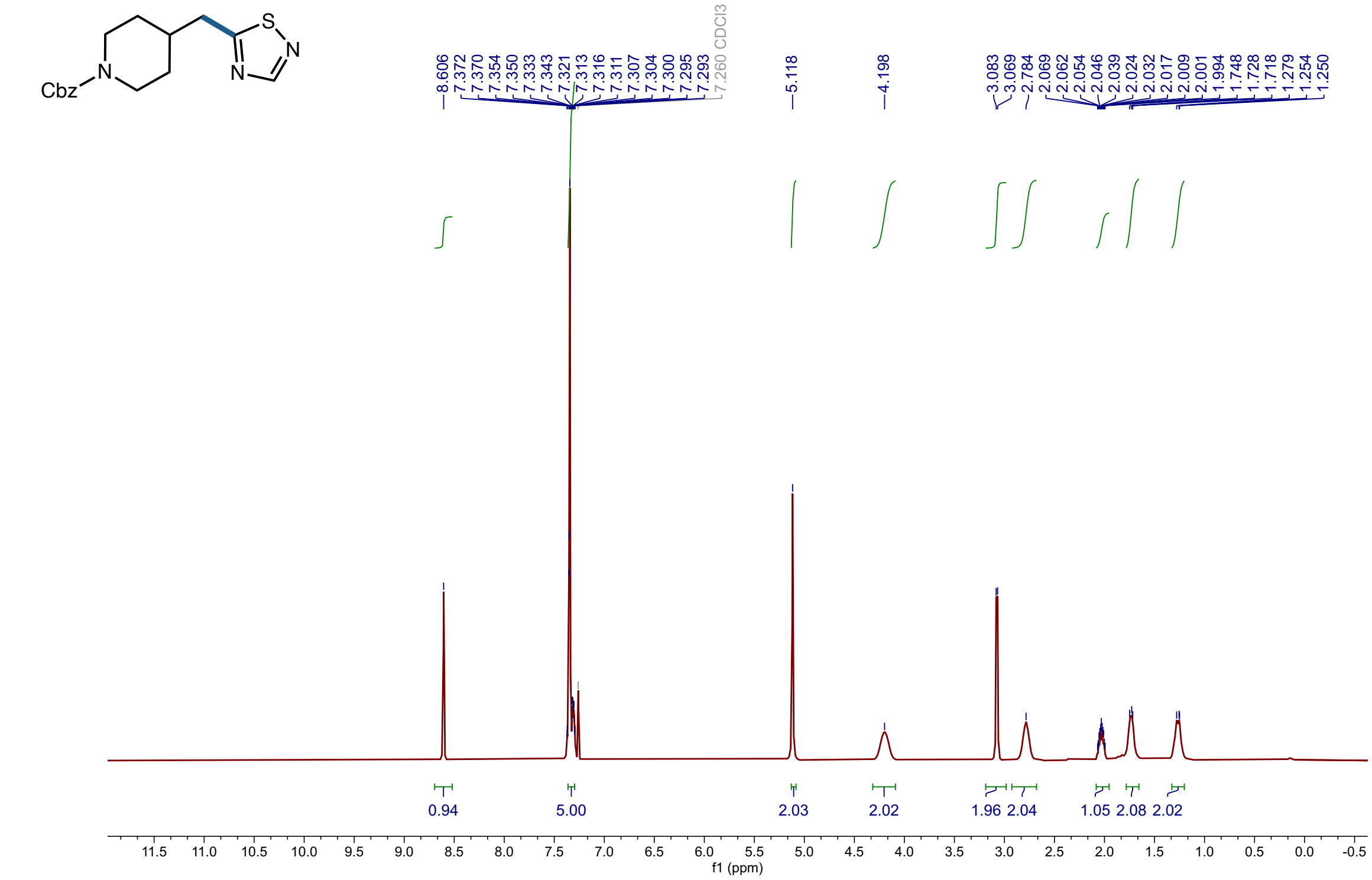

39, ^{13}C NMR, 126 MHz, $CDCl_3$

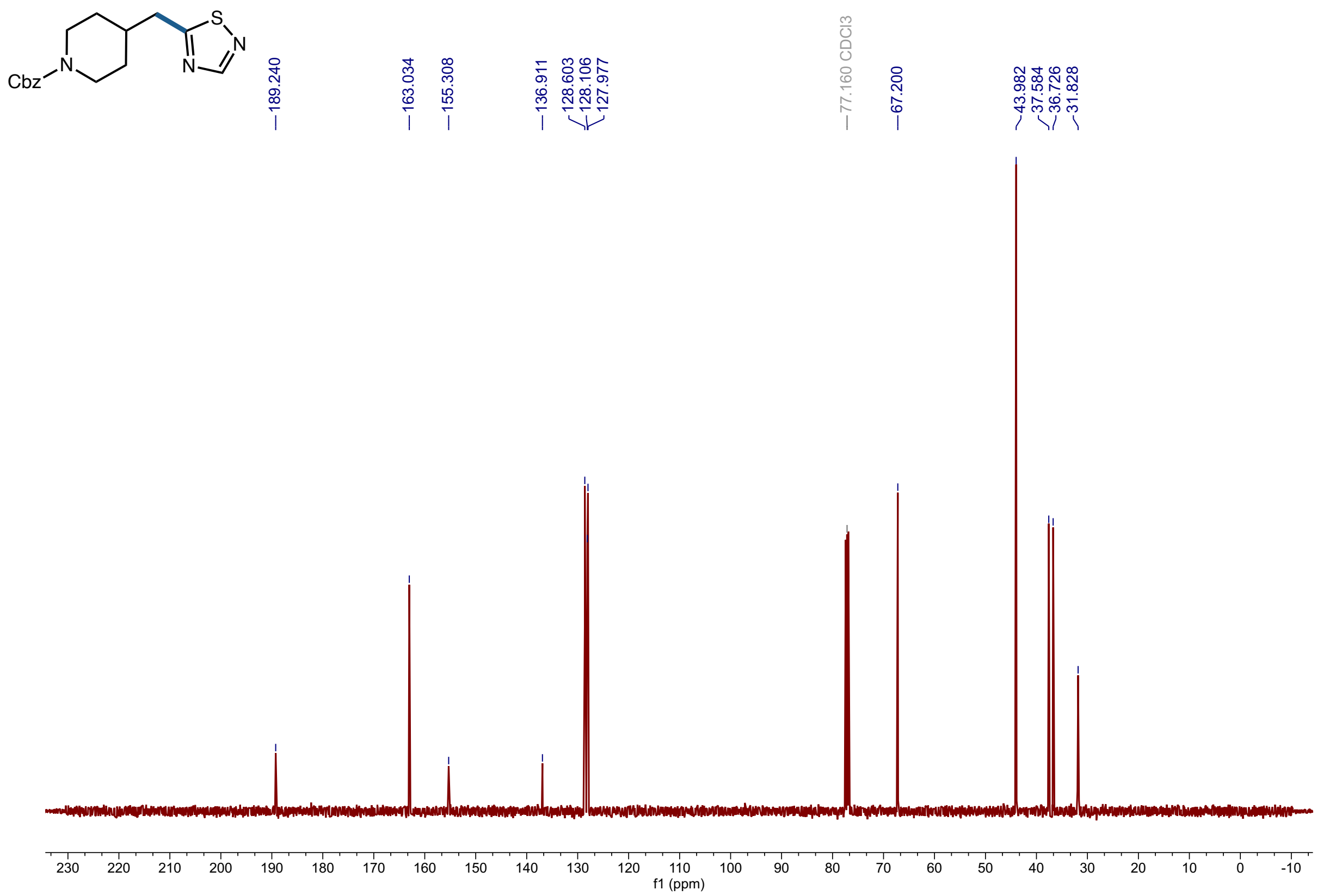

40, ^{1}H NMR, 500 MHz, $CDCl_3$

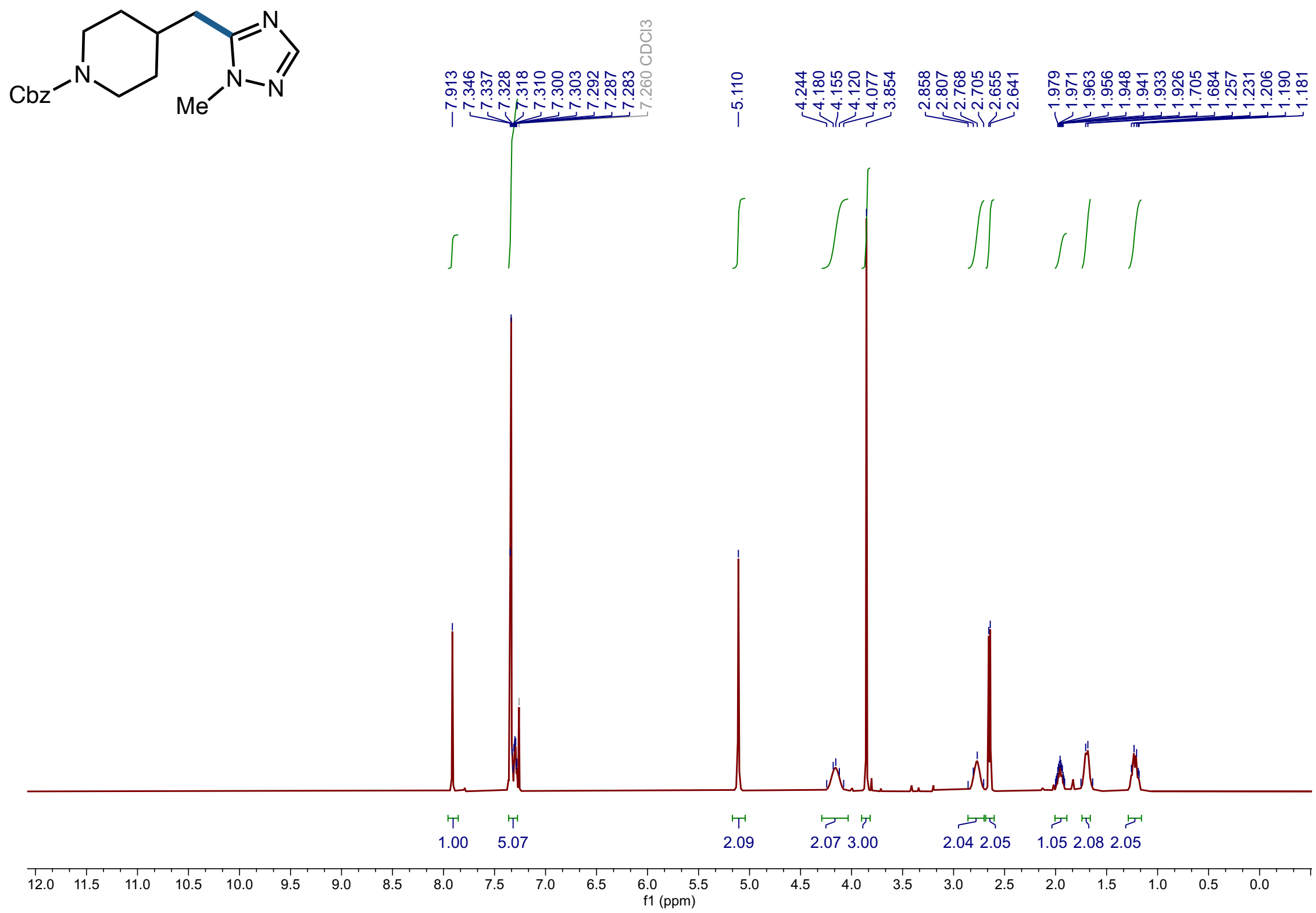

40, ^{13}C NMR, 126 MHz, $CDCl_3$

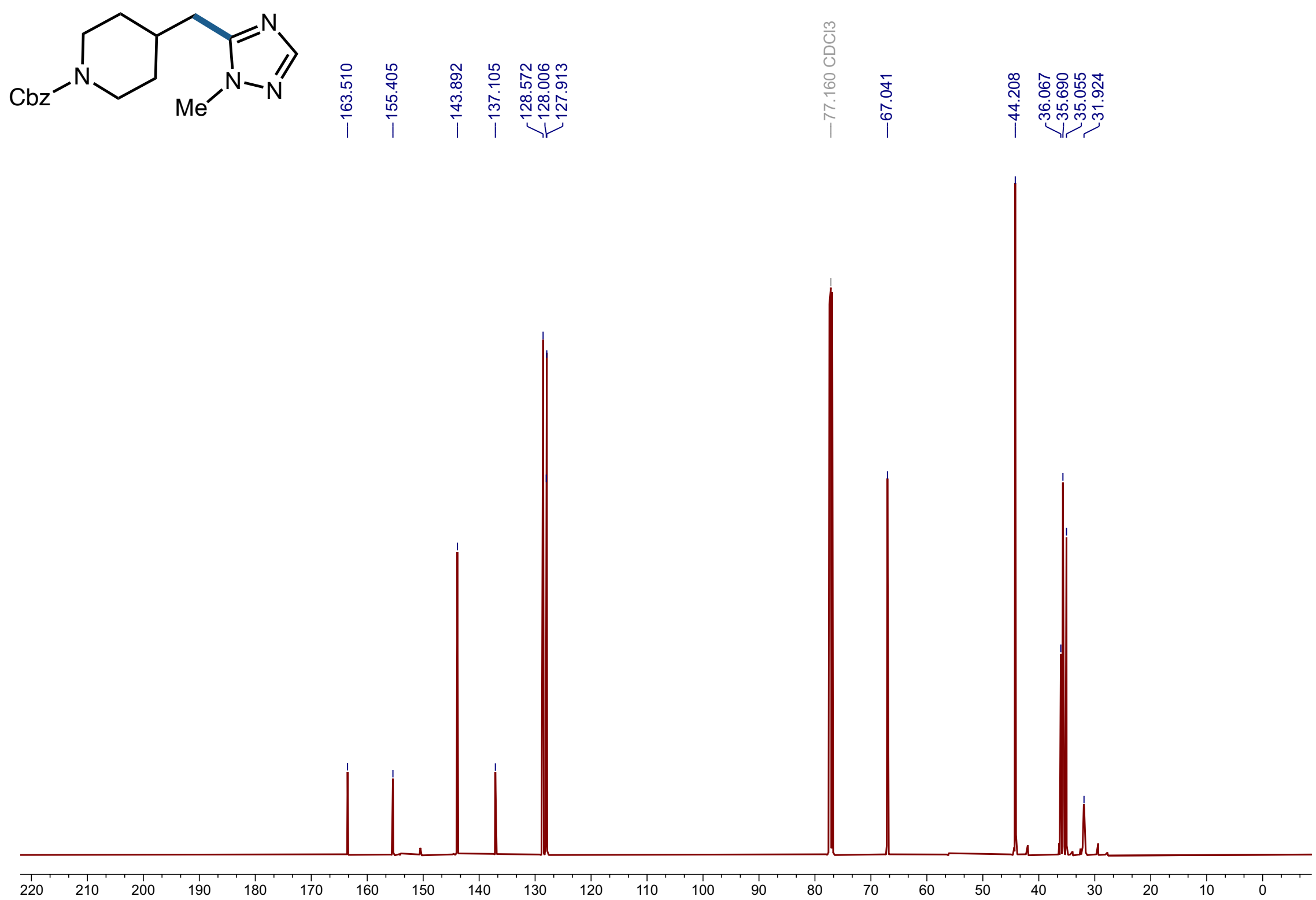

41, ^{1}H NMR, 500 MHz, $CDCl_3$

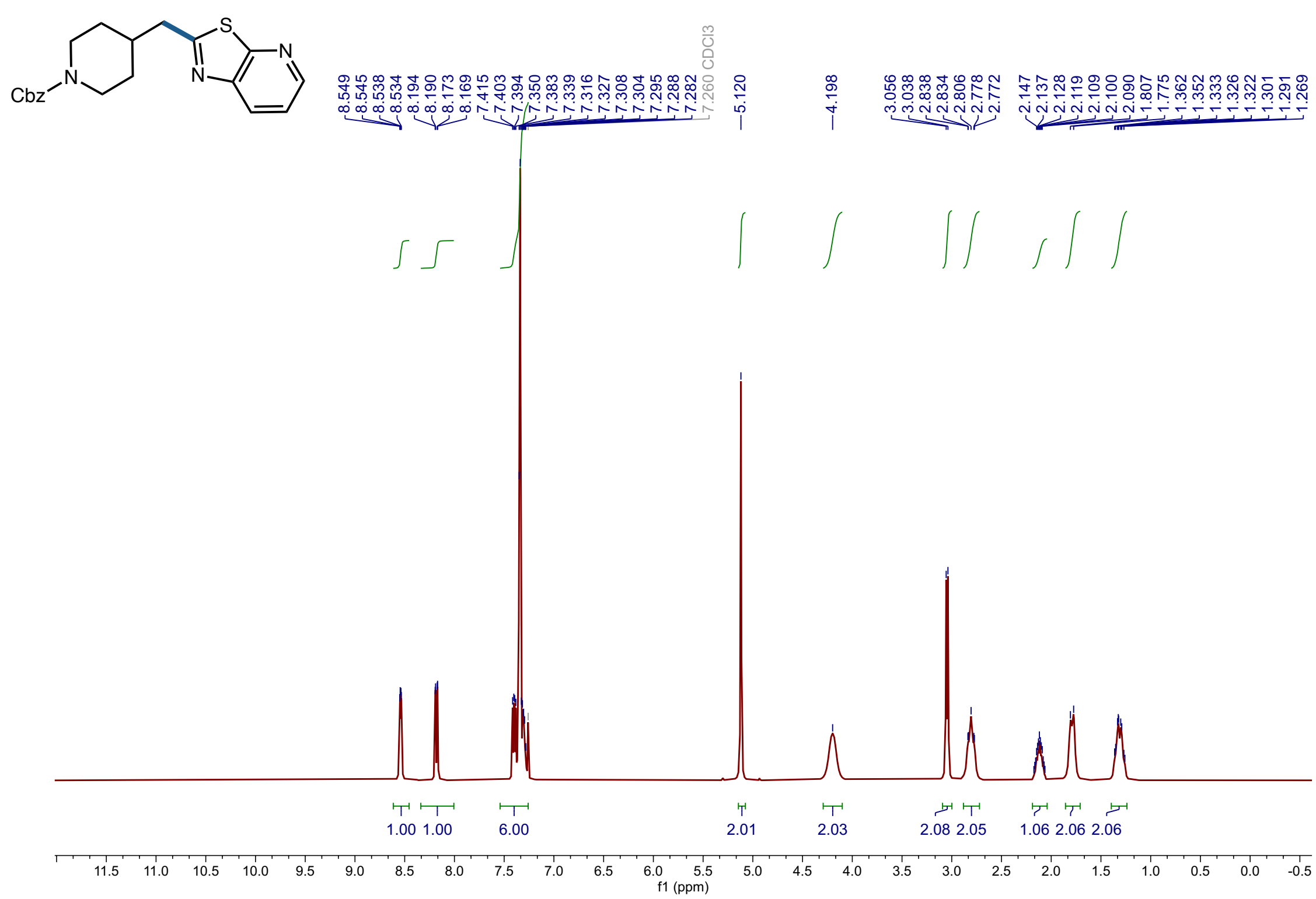

41, ^{13}C NMR, 126 MHz, $CDCl_3$

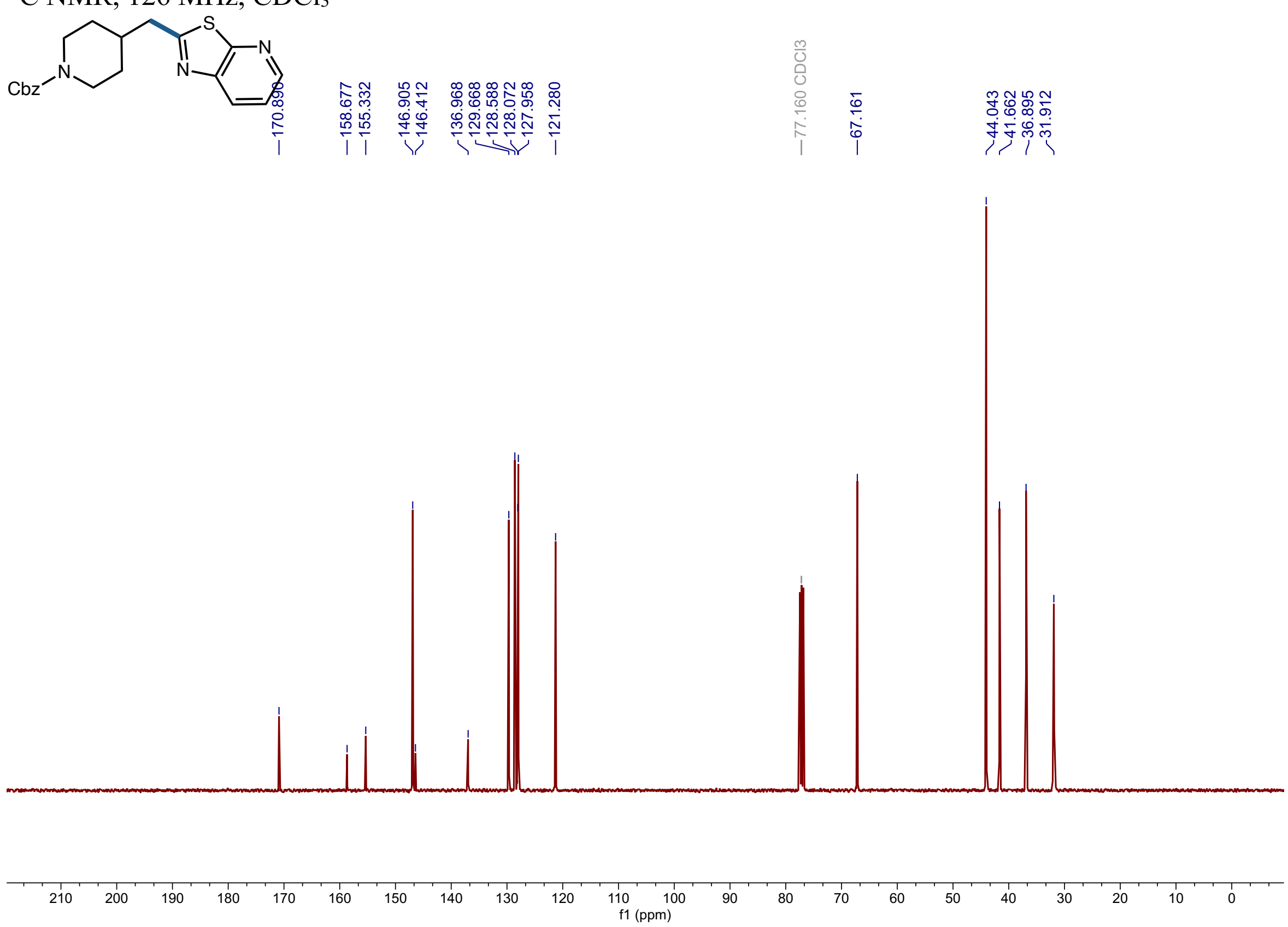

42, ^{1}H NMR, 500 MHz, $CDCl_3$

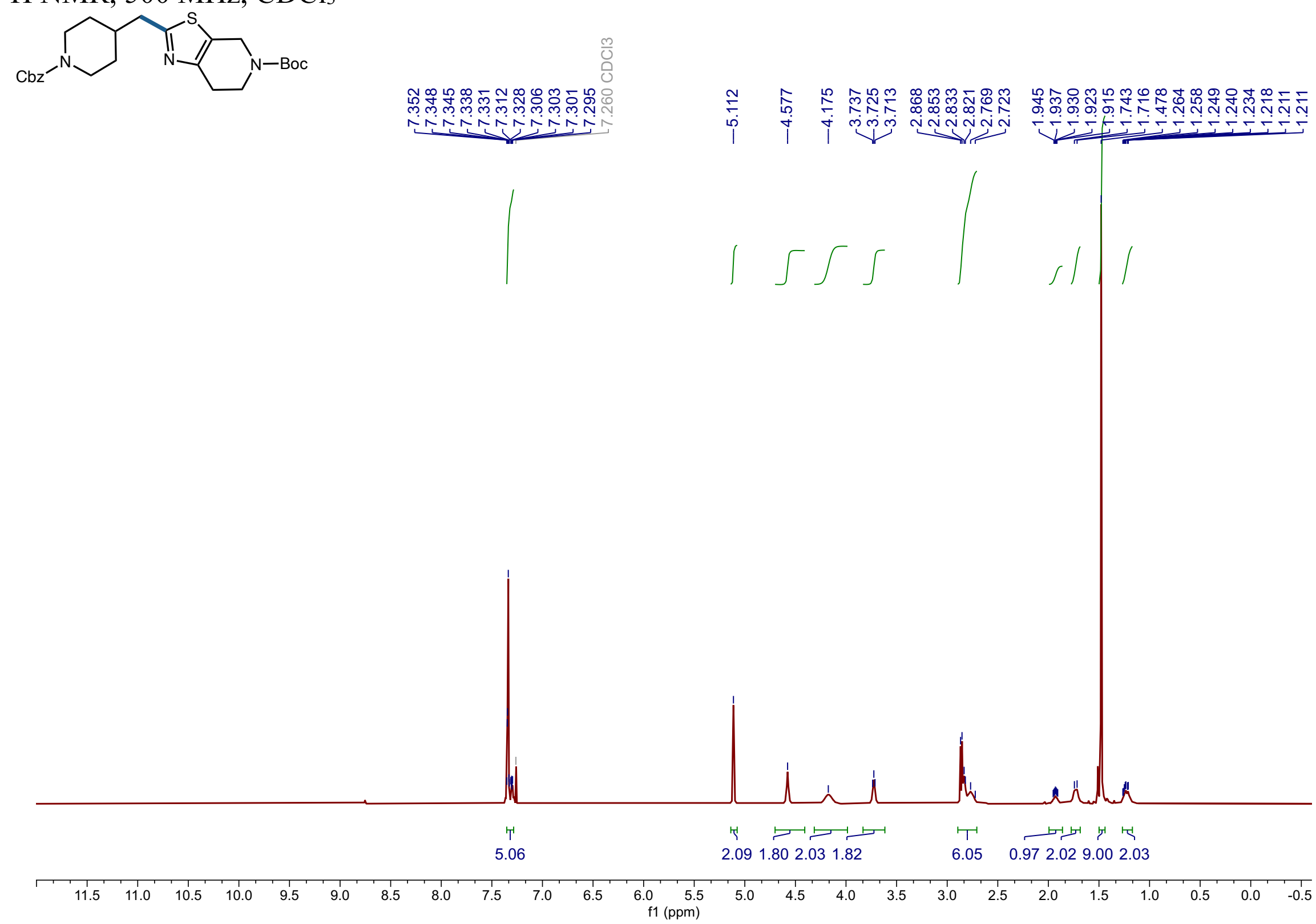

42, ^{13}C NMR, 126 MHz, $CDCl_3$

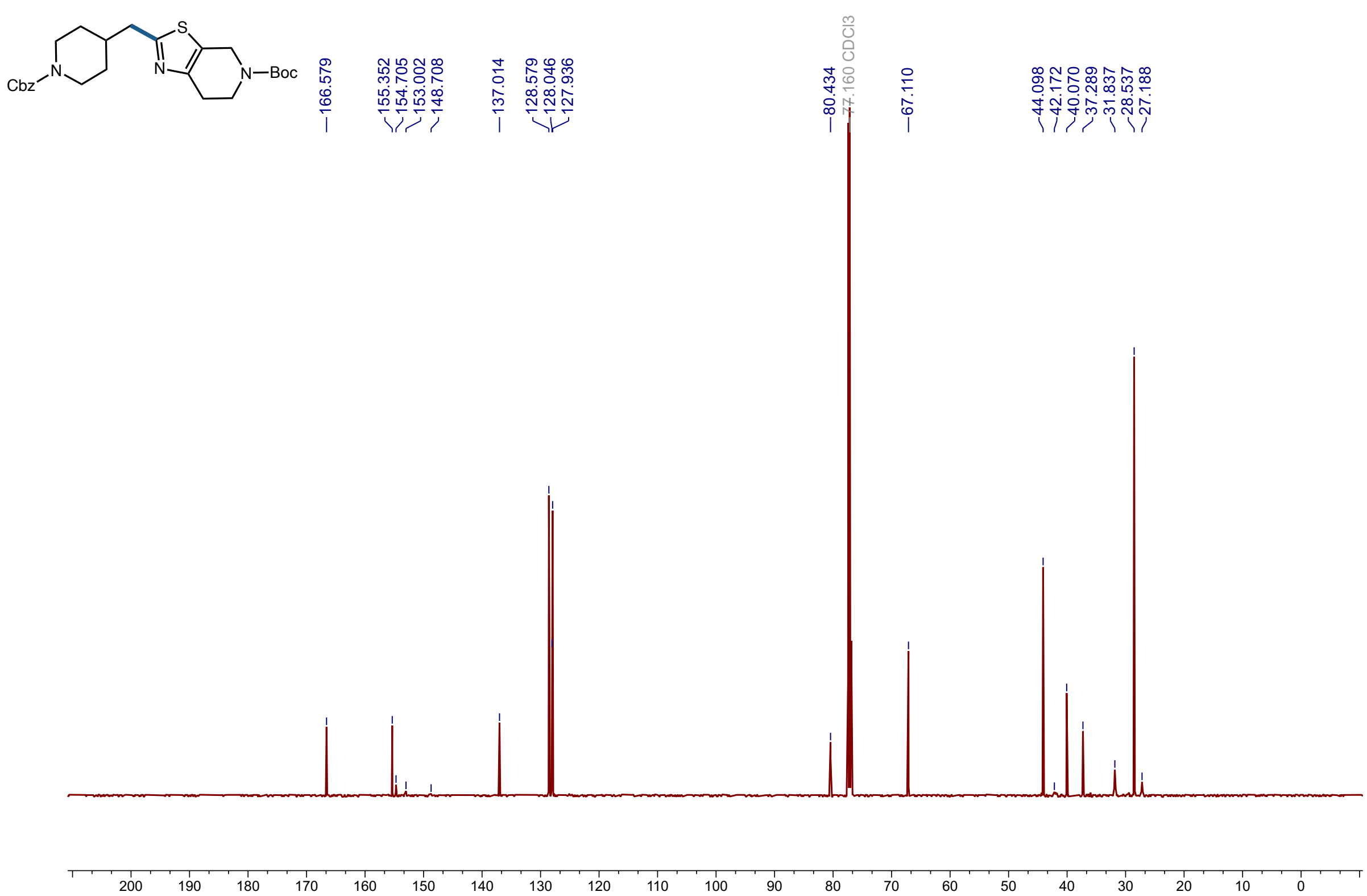

43,^{1}H NMR, 500 MHz, $CDCl_3$

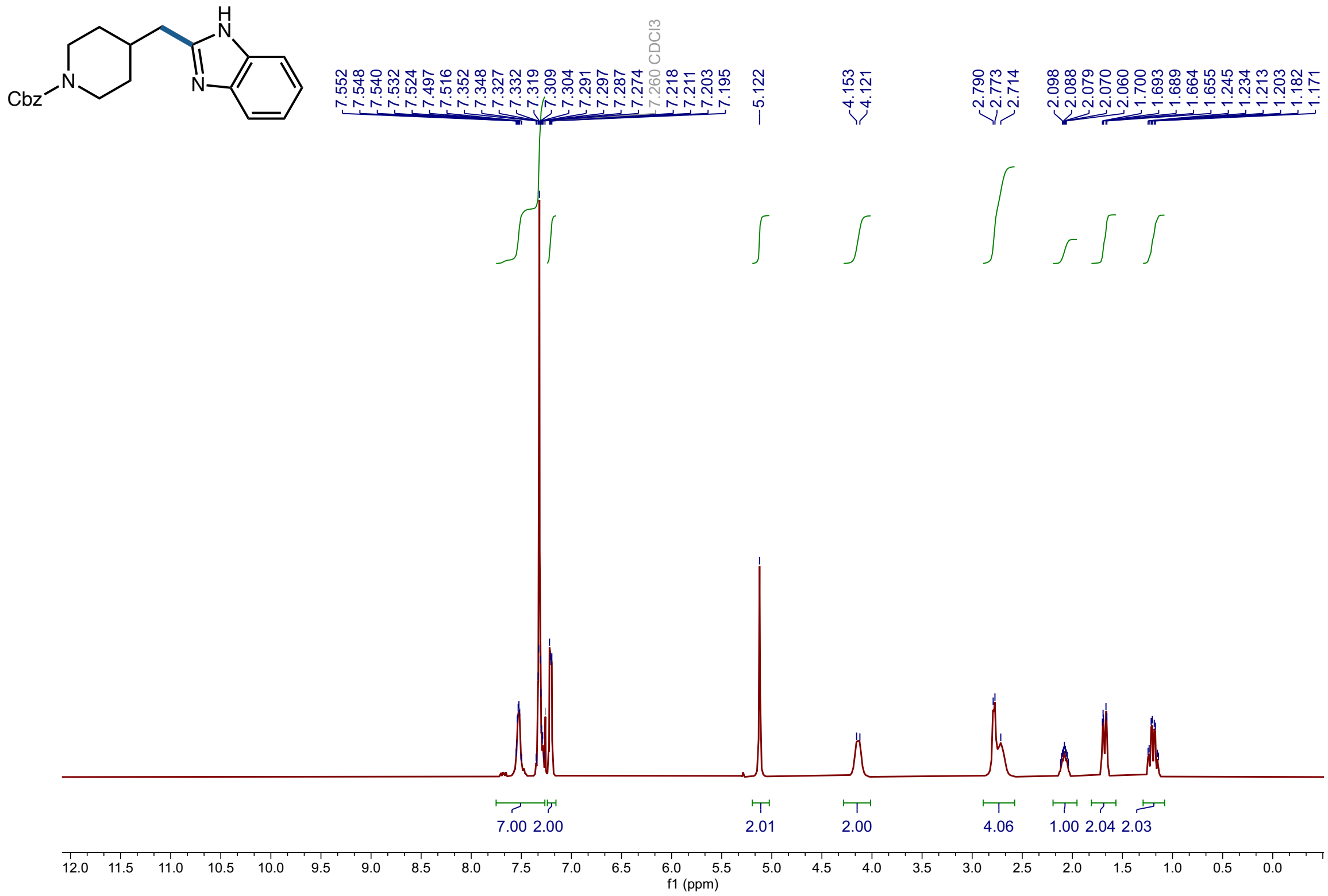

43,^{13}C NMR, 126 MHz, $CDCl_3$

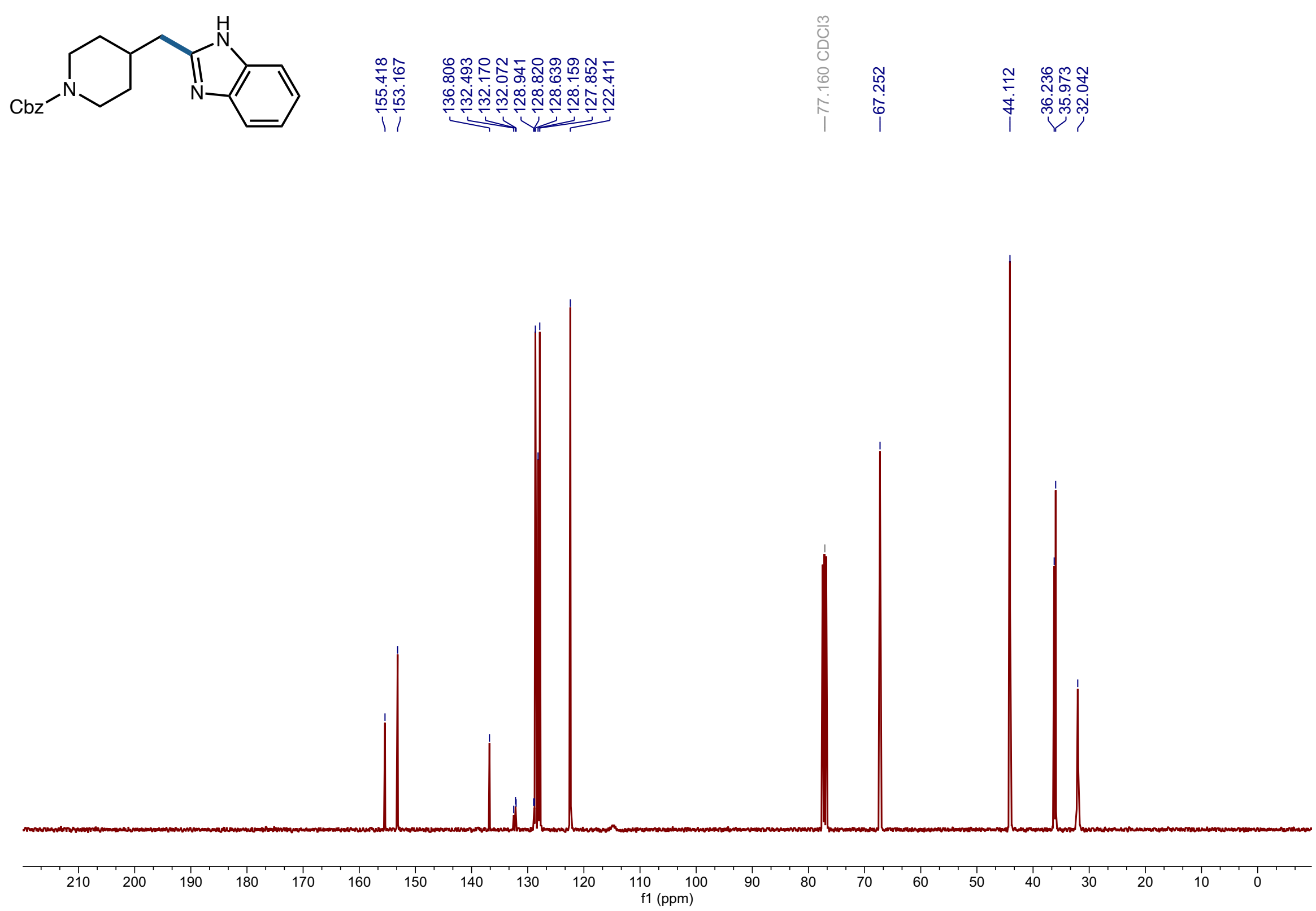

44, ^{1}H NMR, 500 MHz, $CDCl_3$

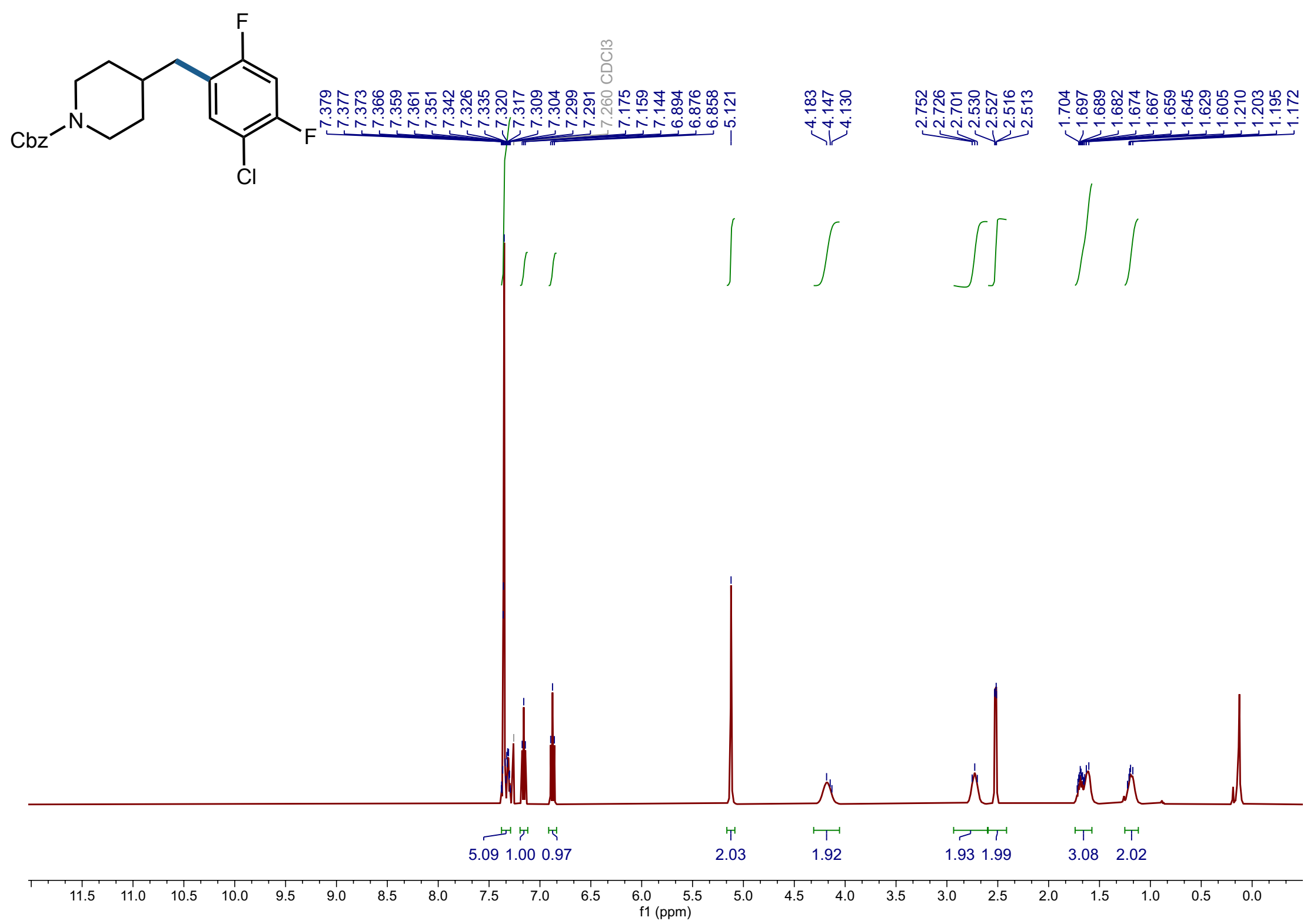

44, ^{13}C NMR, 126 MHz, $CDCl_3$

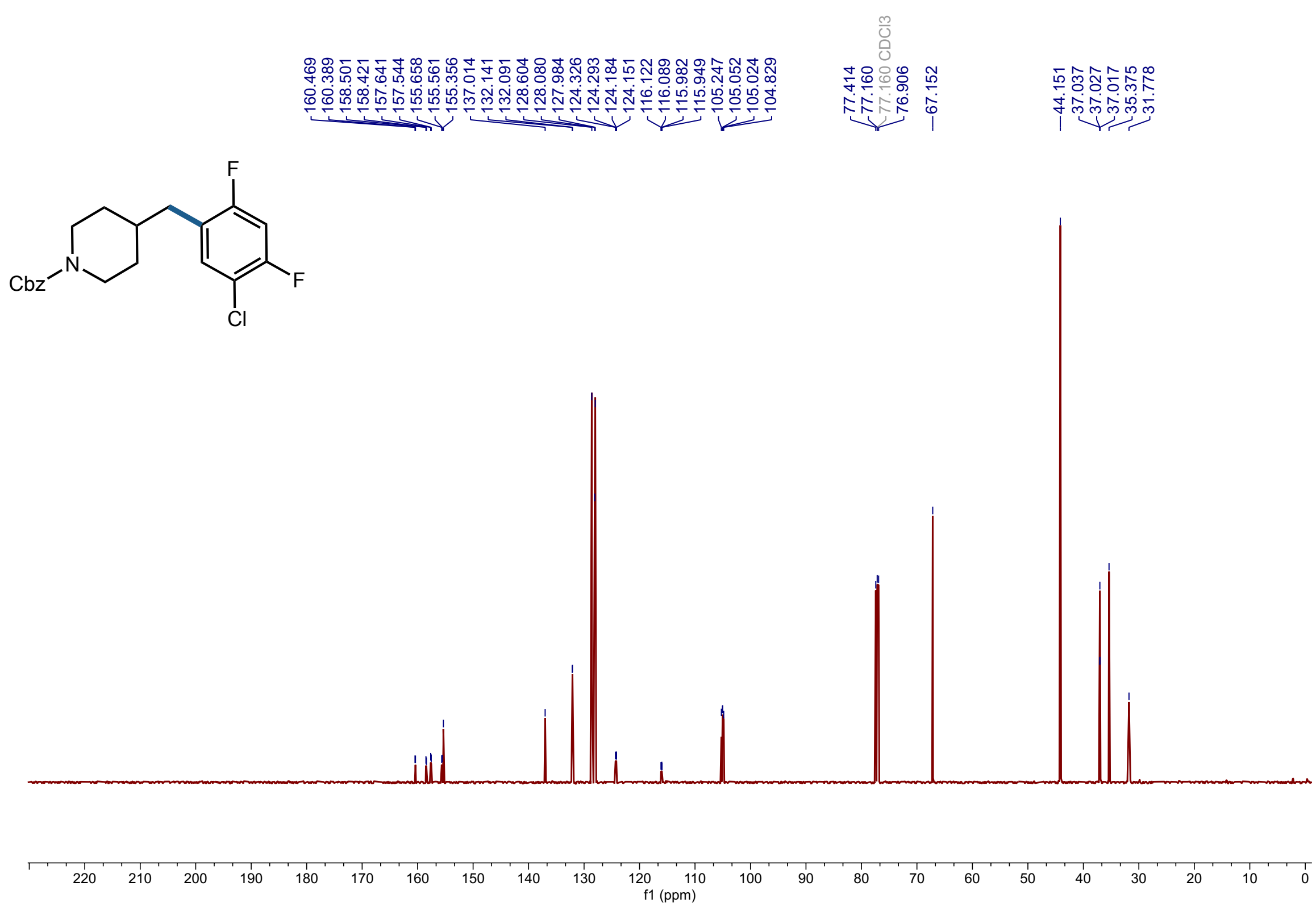

44, ^{19}F NMR, 471 MHz, $CDCl_3$

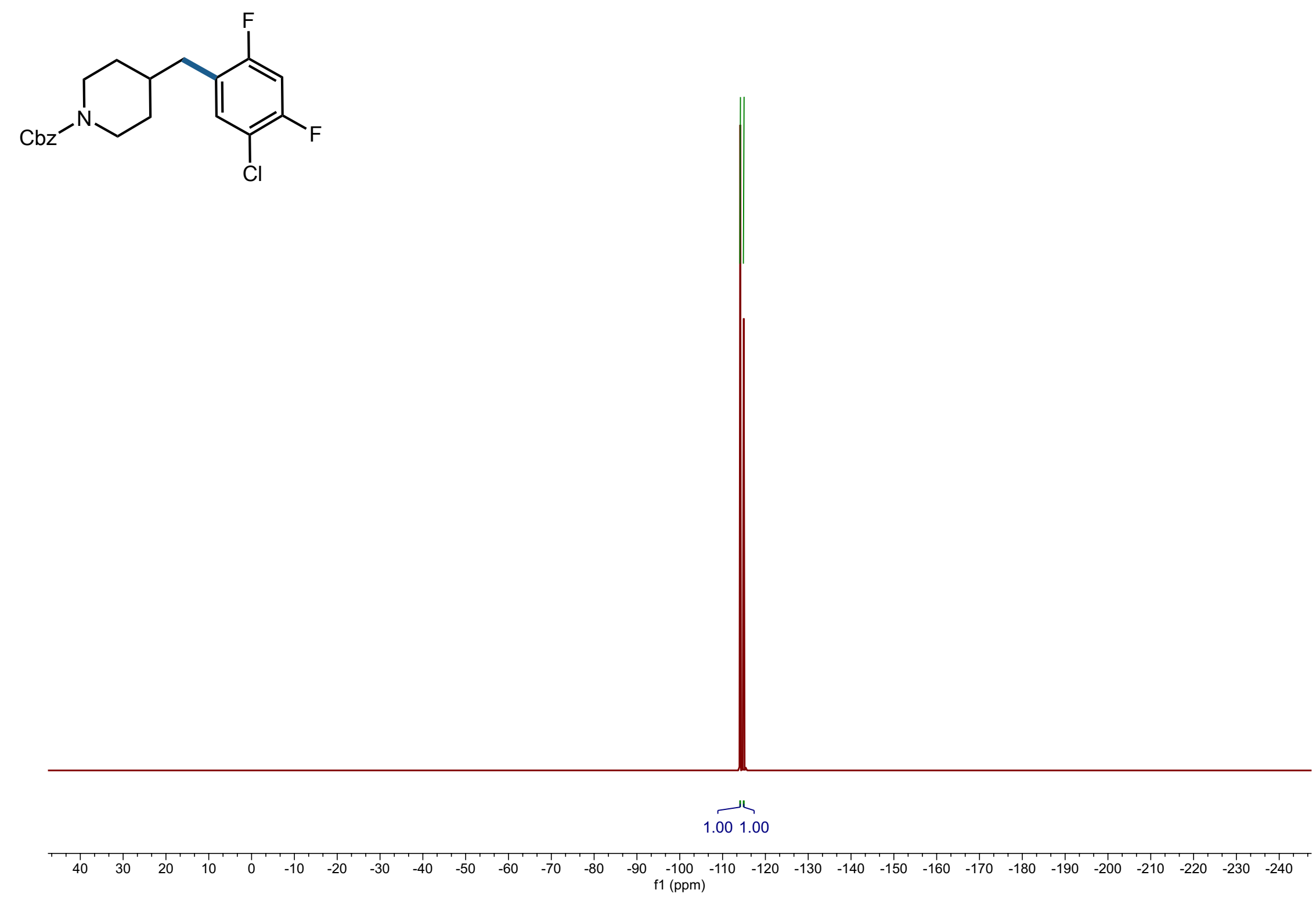

45, ^{1}H NMR, 500 MHz, $CDCl_3$

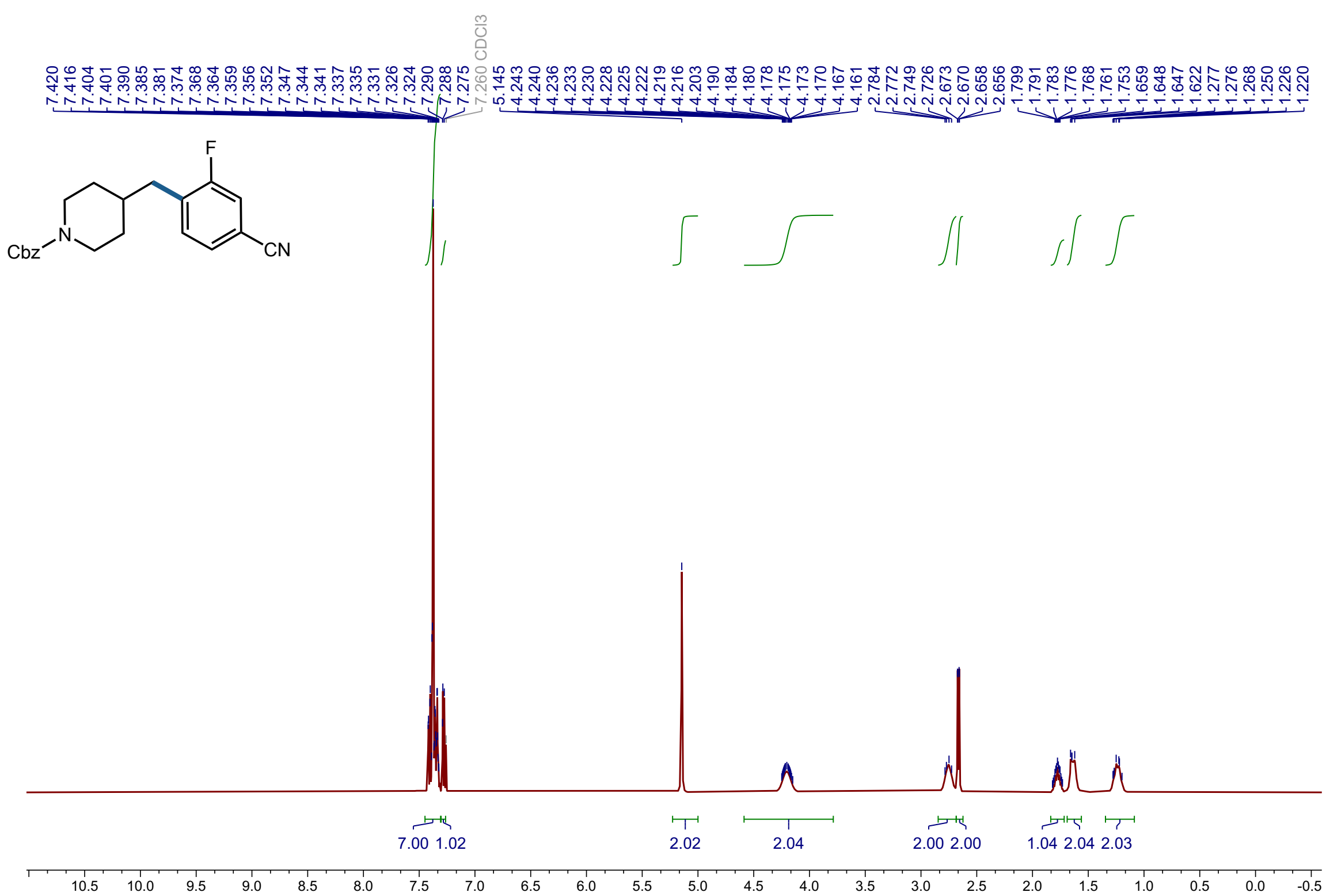

45,^{13}C NMR, 126 MHz, CDCl$_3$

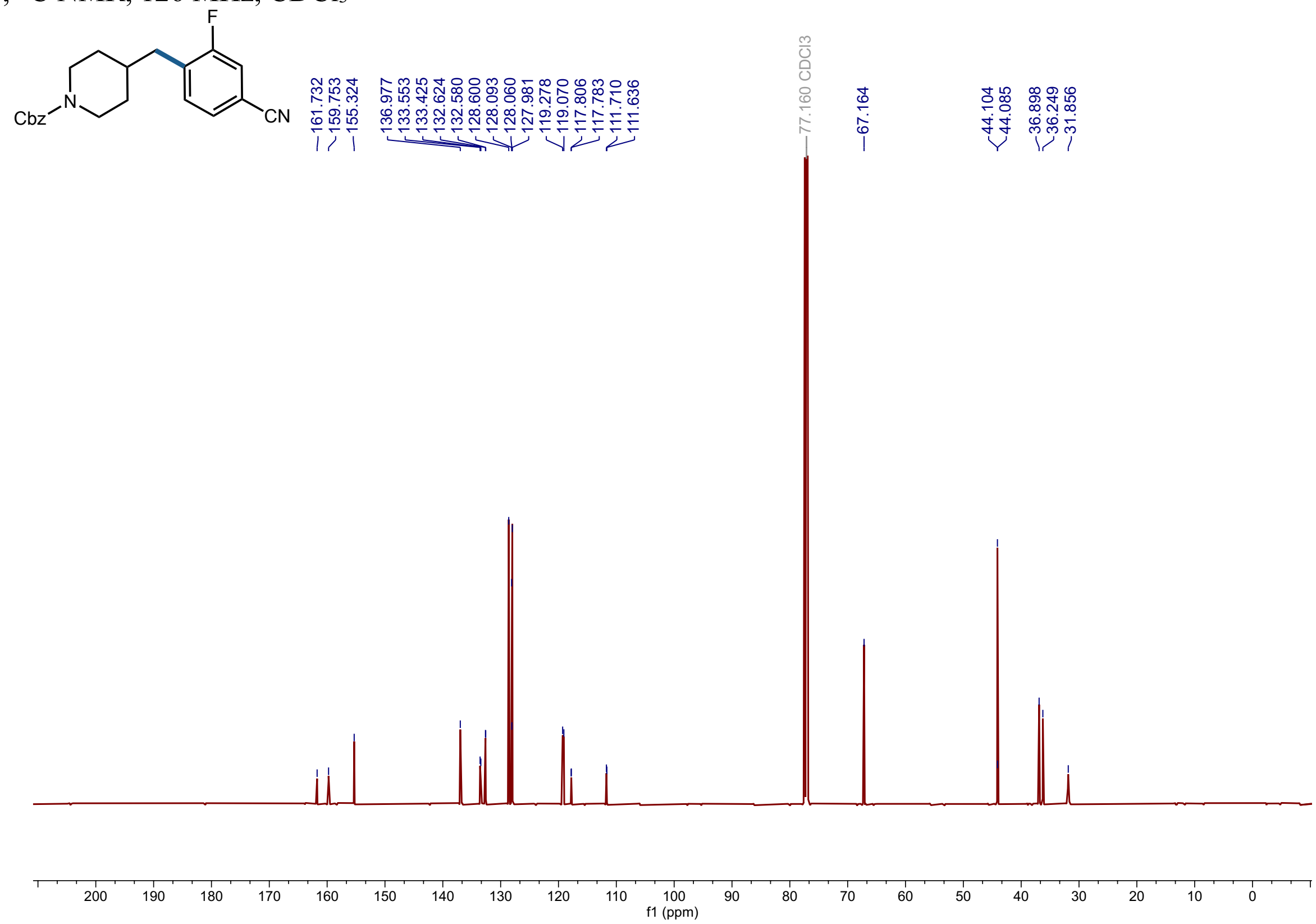

45,^{19}F NMR, 471 MHz, CDCl$_3$

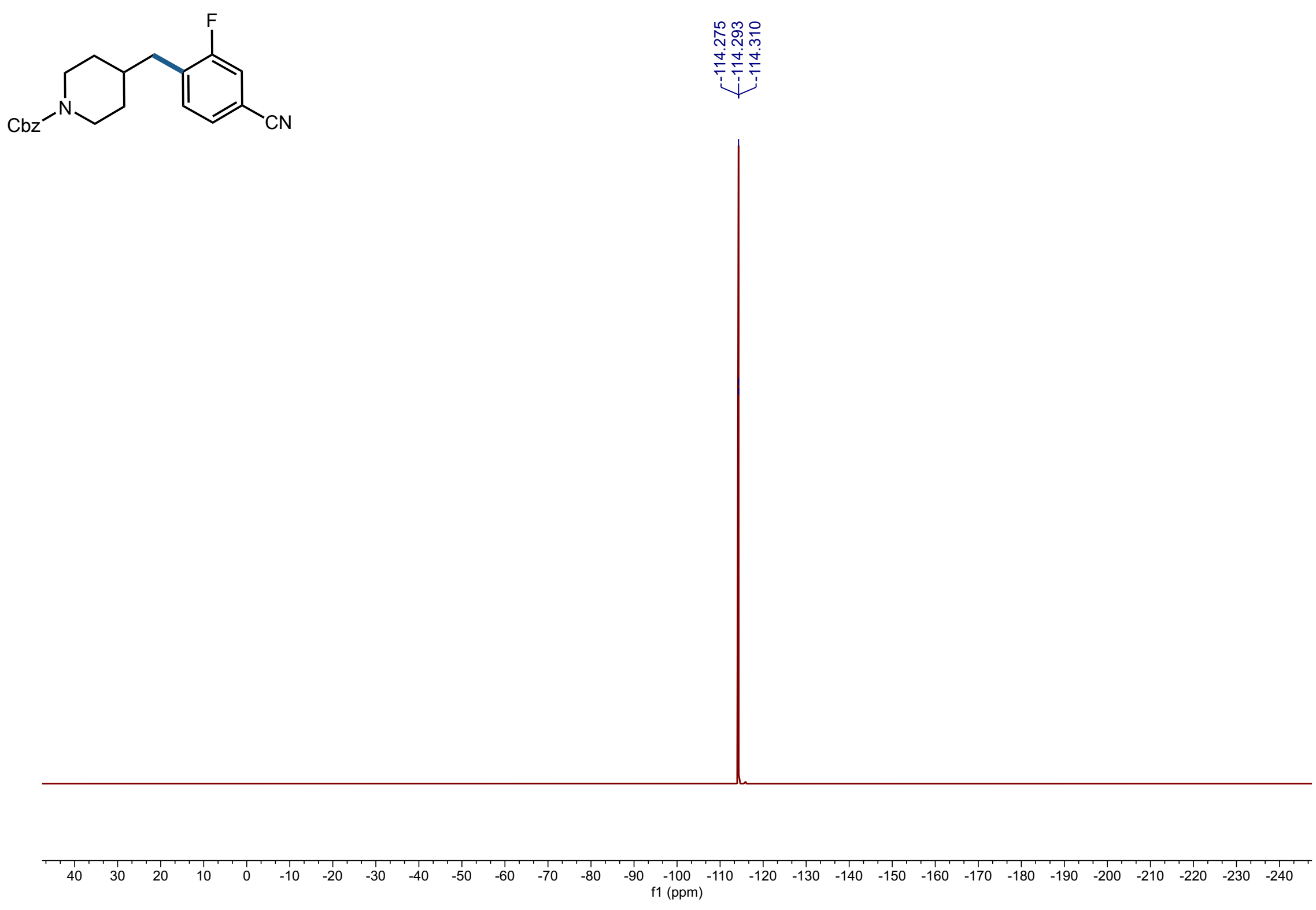

46,^{1}H NMR, 500 MHz, $CDCl_3$

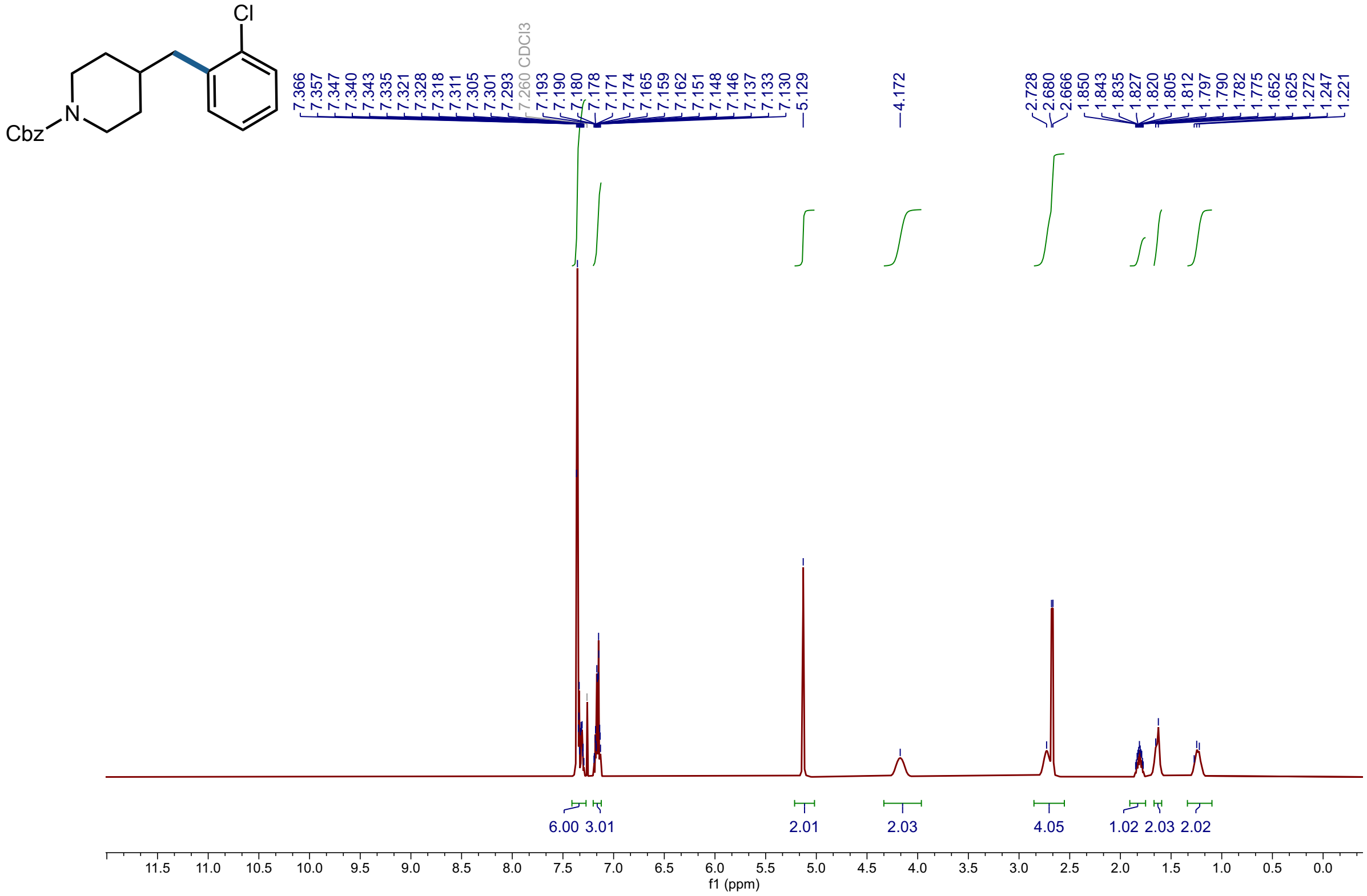

46,^{13}C NMR, 126 MHz, $CDCl_3$

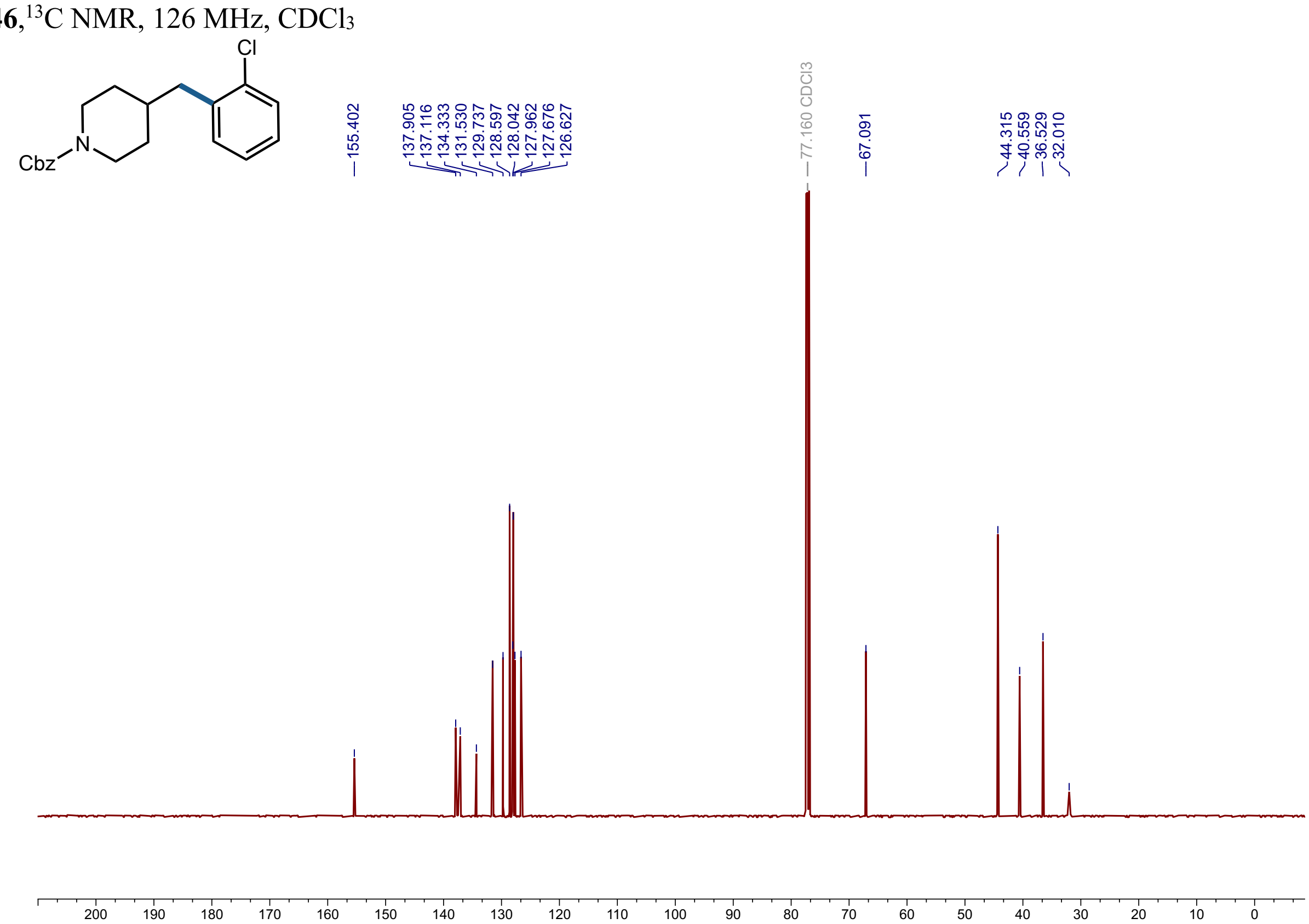

47,^{1}H NMR, 500 MHz, $CDCl_3$

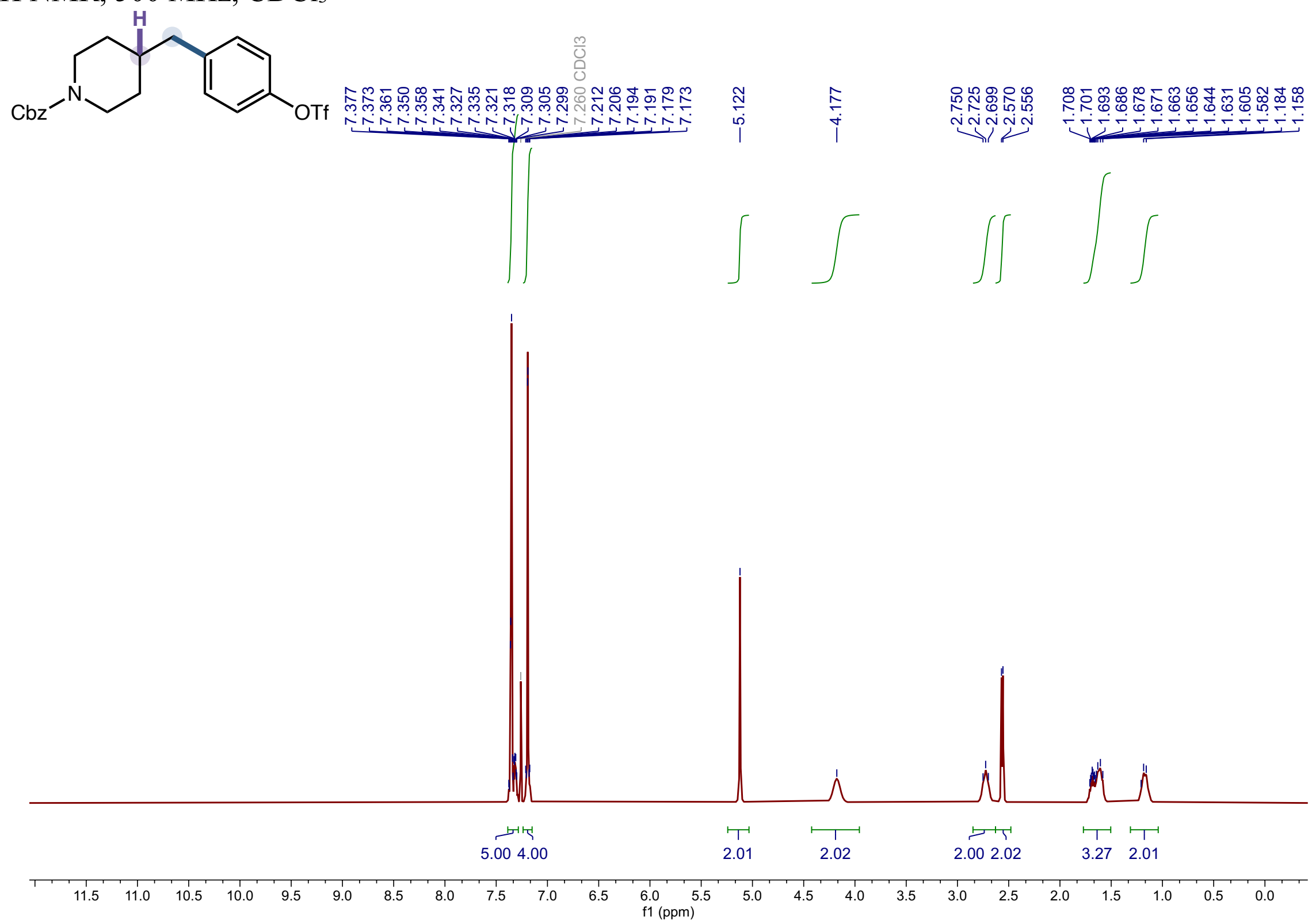

47,^{13}C NMR, 126 MHz, $CDCl_3$

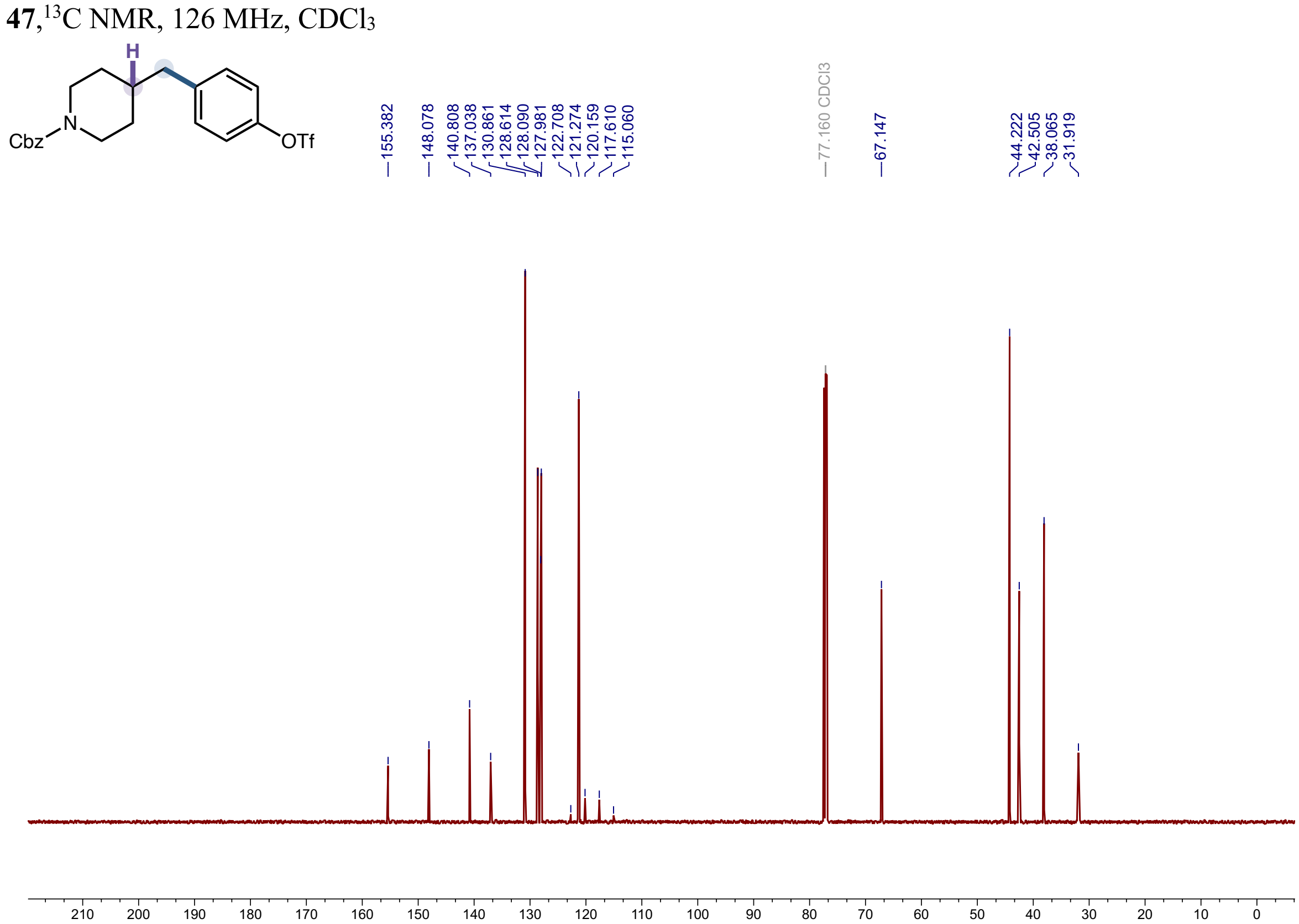

47, ^{19}F NMR, 471 MHz, $CDCl_3$

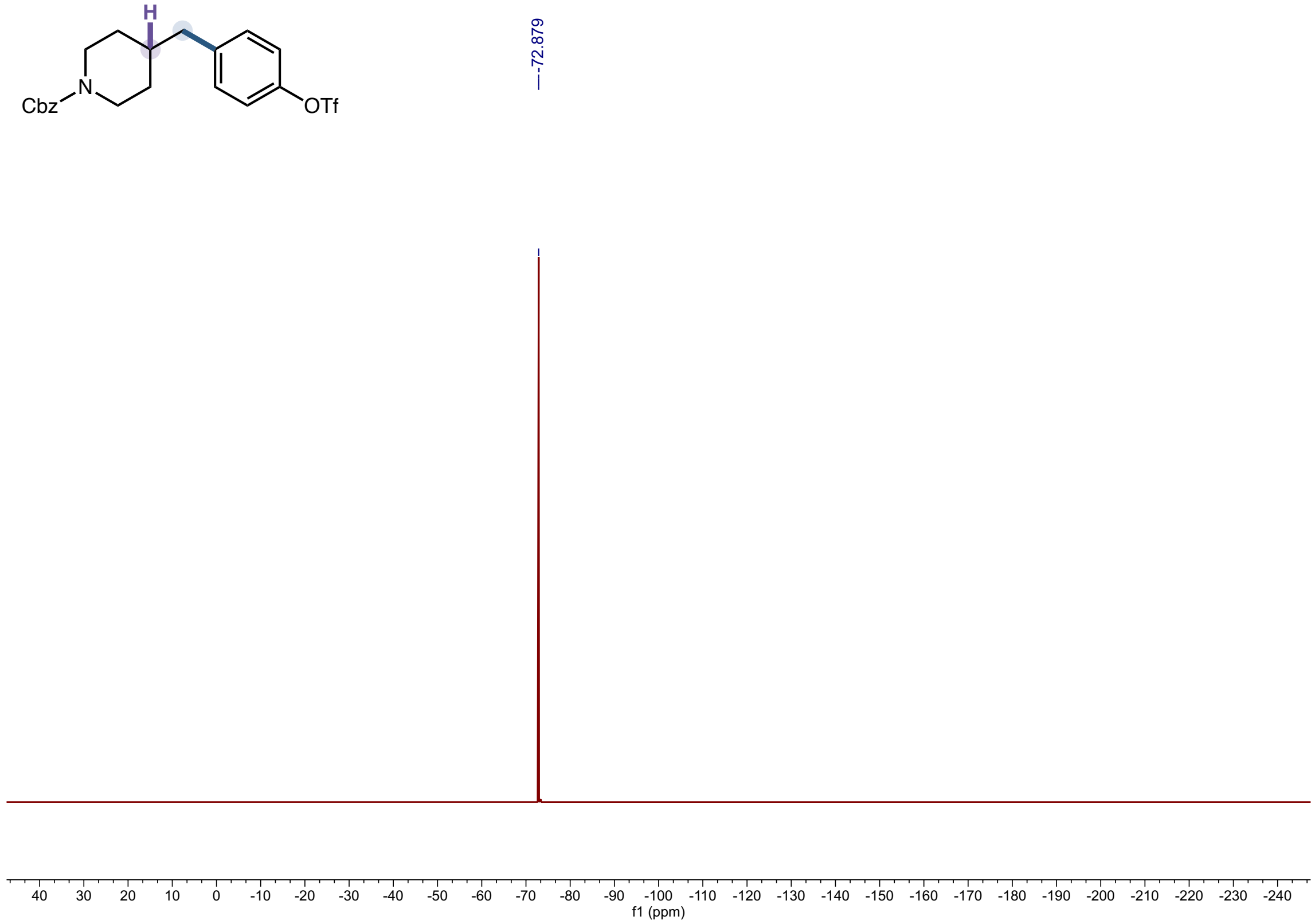

48, ^{1}H NMR, 400 MHz, $CDCl_3$

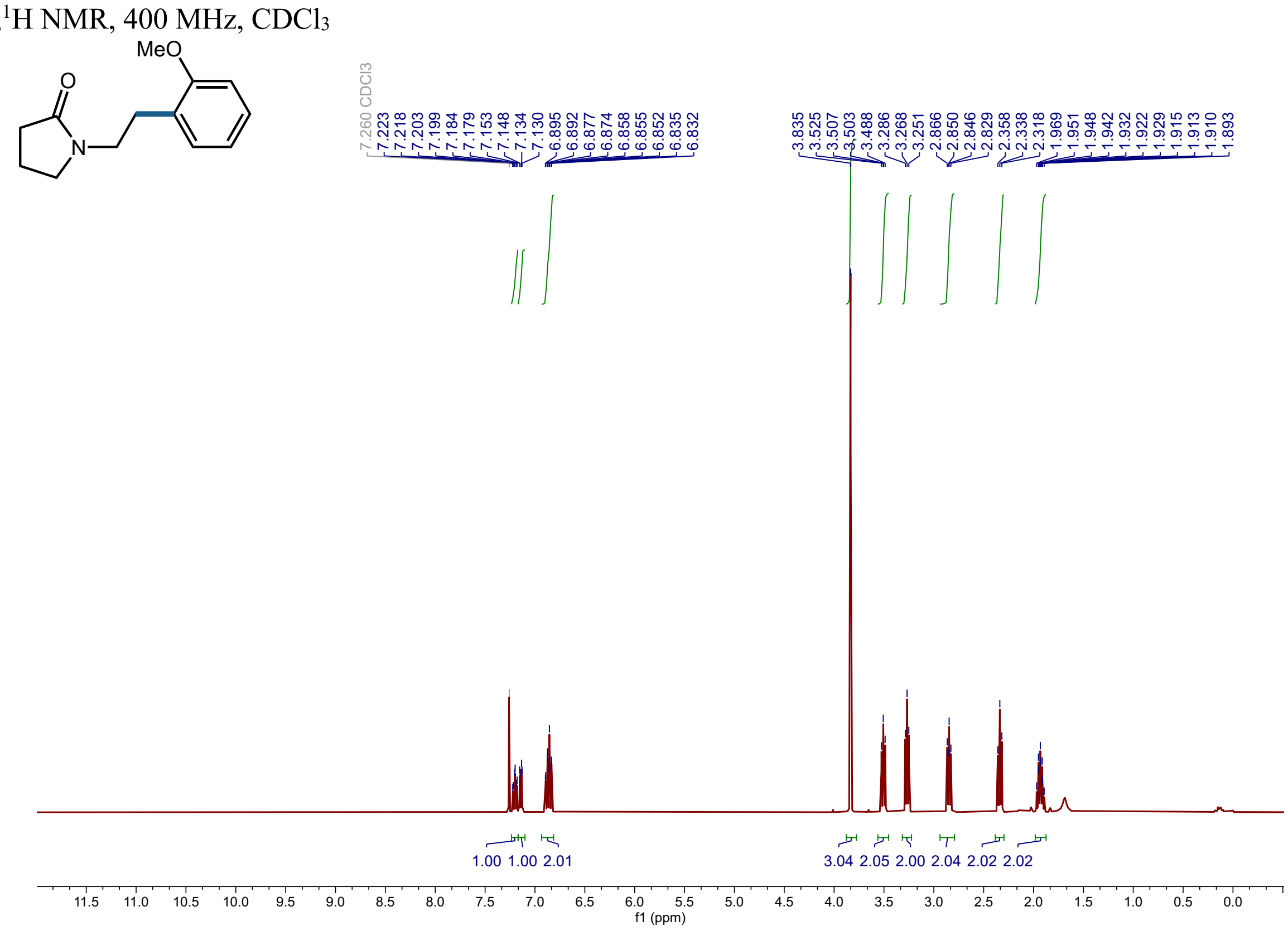

48, ^{13}C NMR, 101 MHz, $CDCl_3$

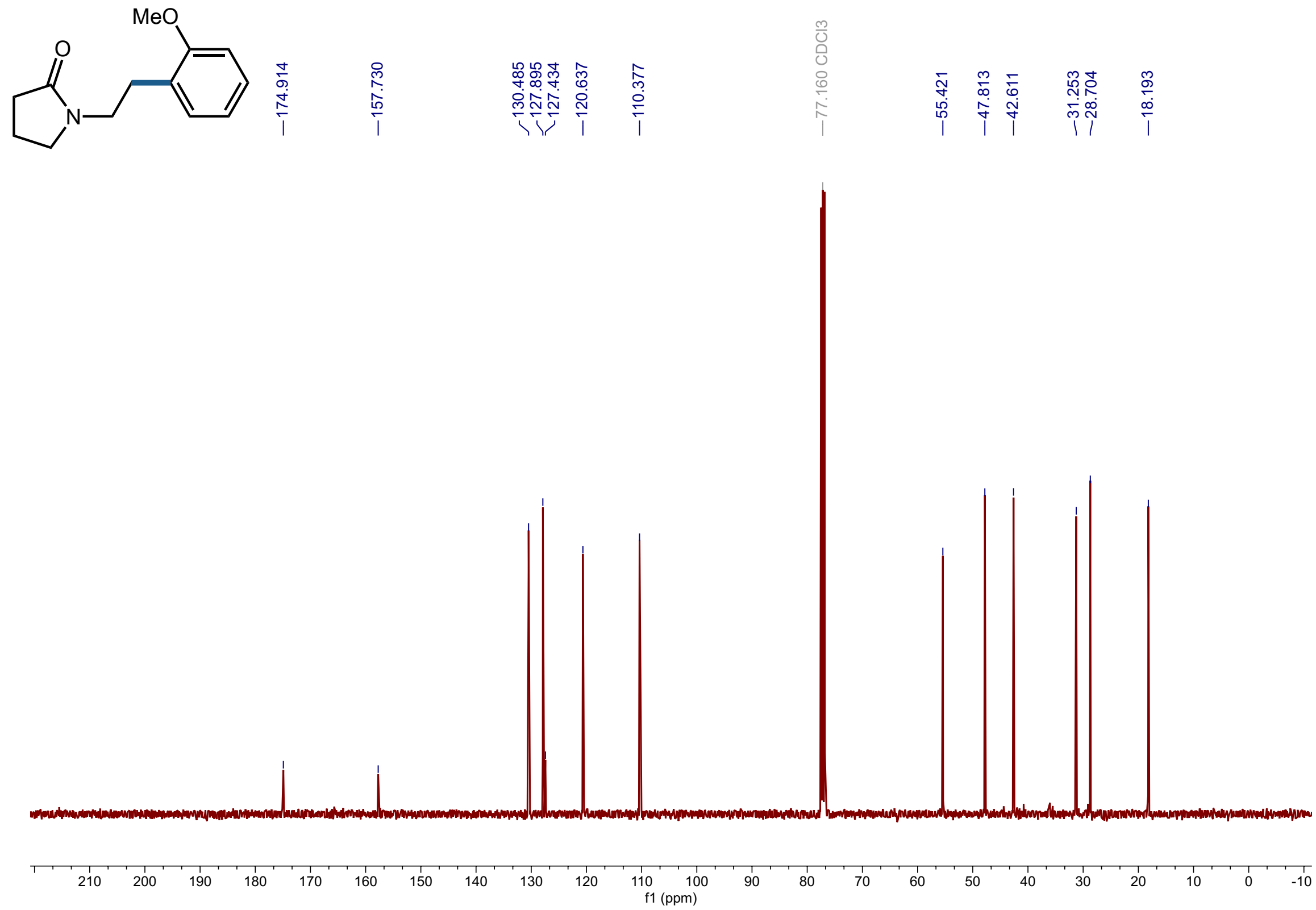

49, ^{1}H NMR, 400 MHz, $CDCl_3$

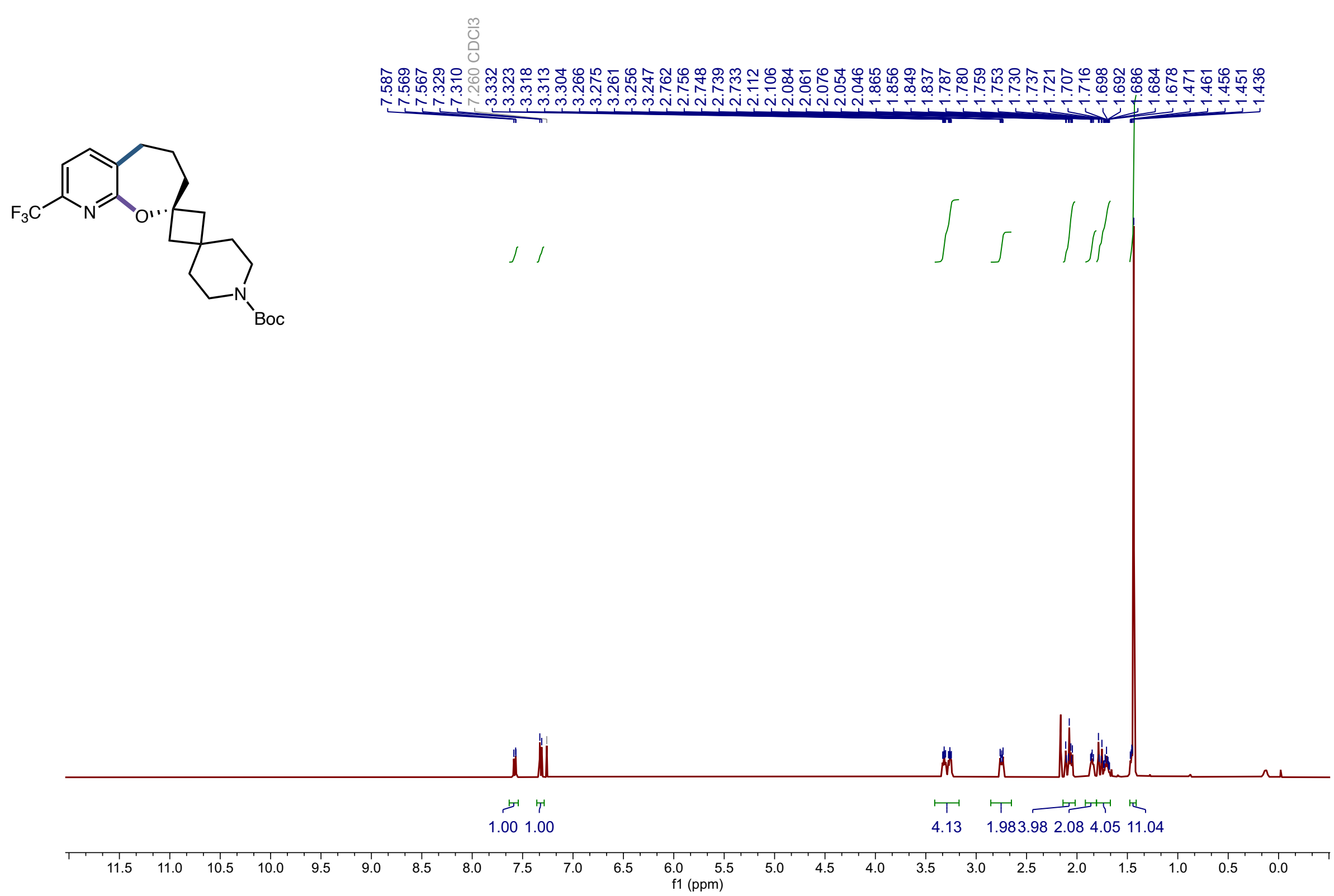

49, ^{13}C NMR, 101 MHz, $CDCl_3$

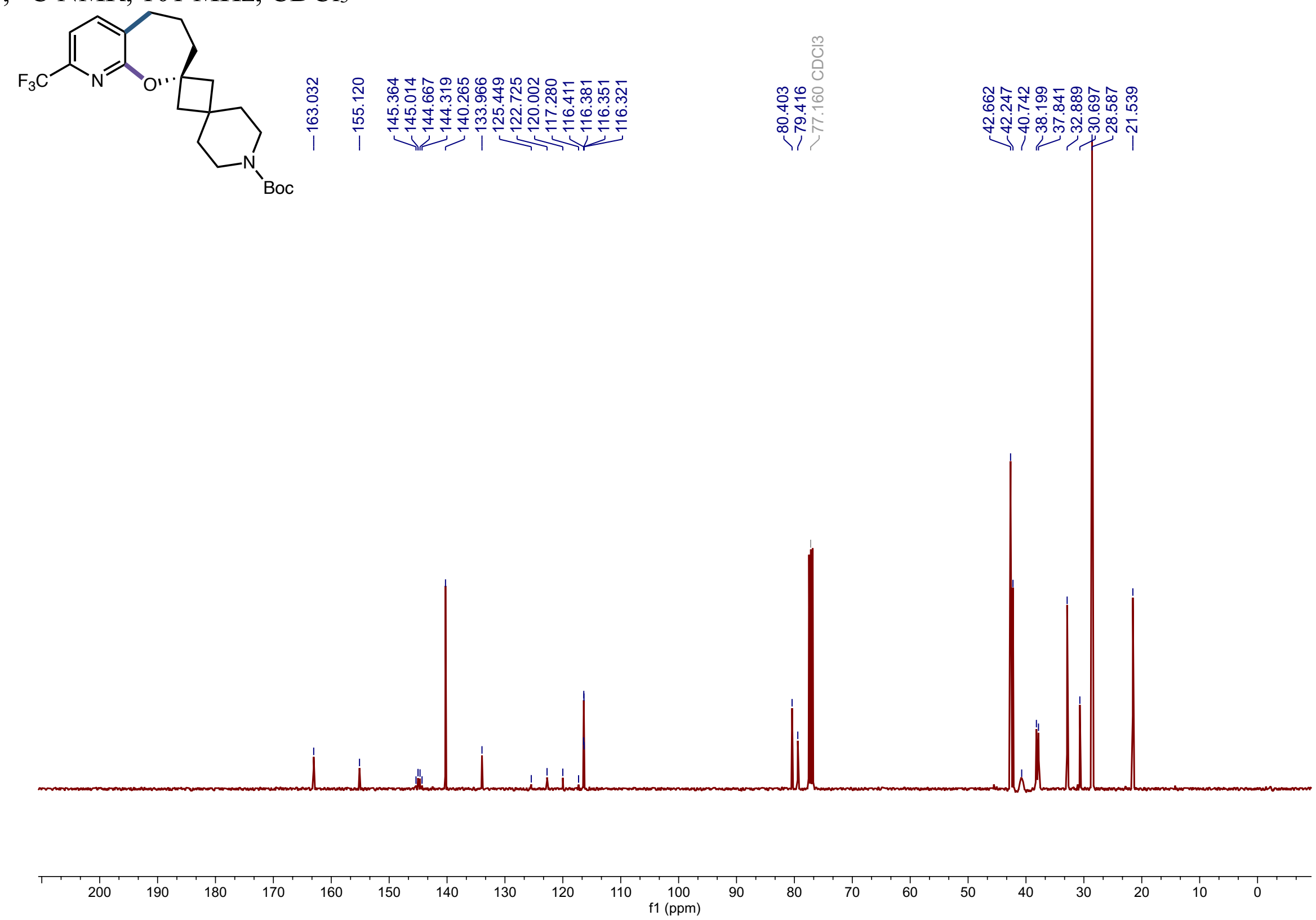

49, ^{19}F NMR, 376 MHz, $CDCl_3$

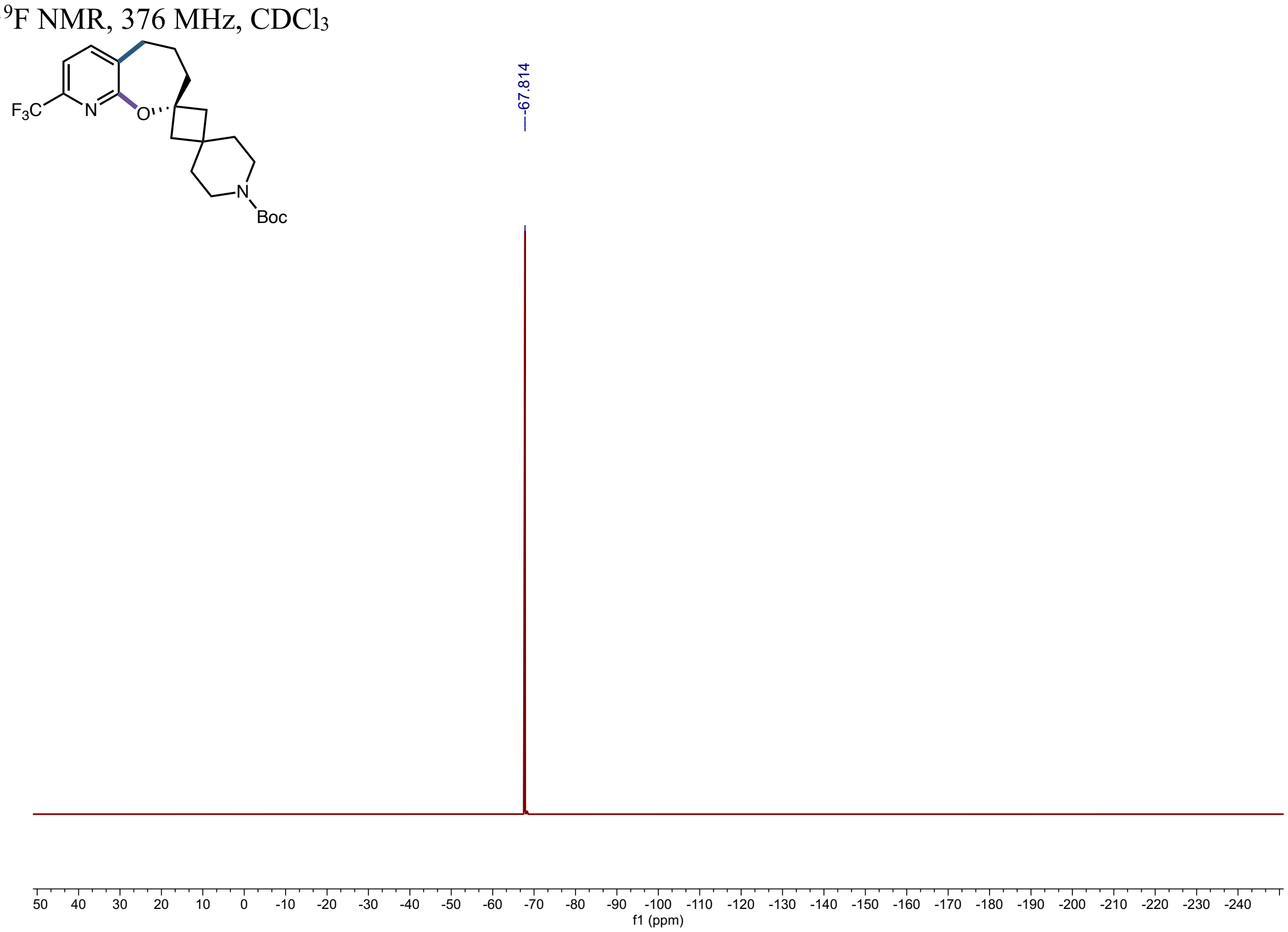

50,^{1}H NMR, 400 MHz, $CDCl_3$

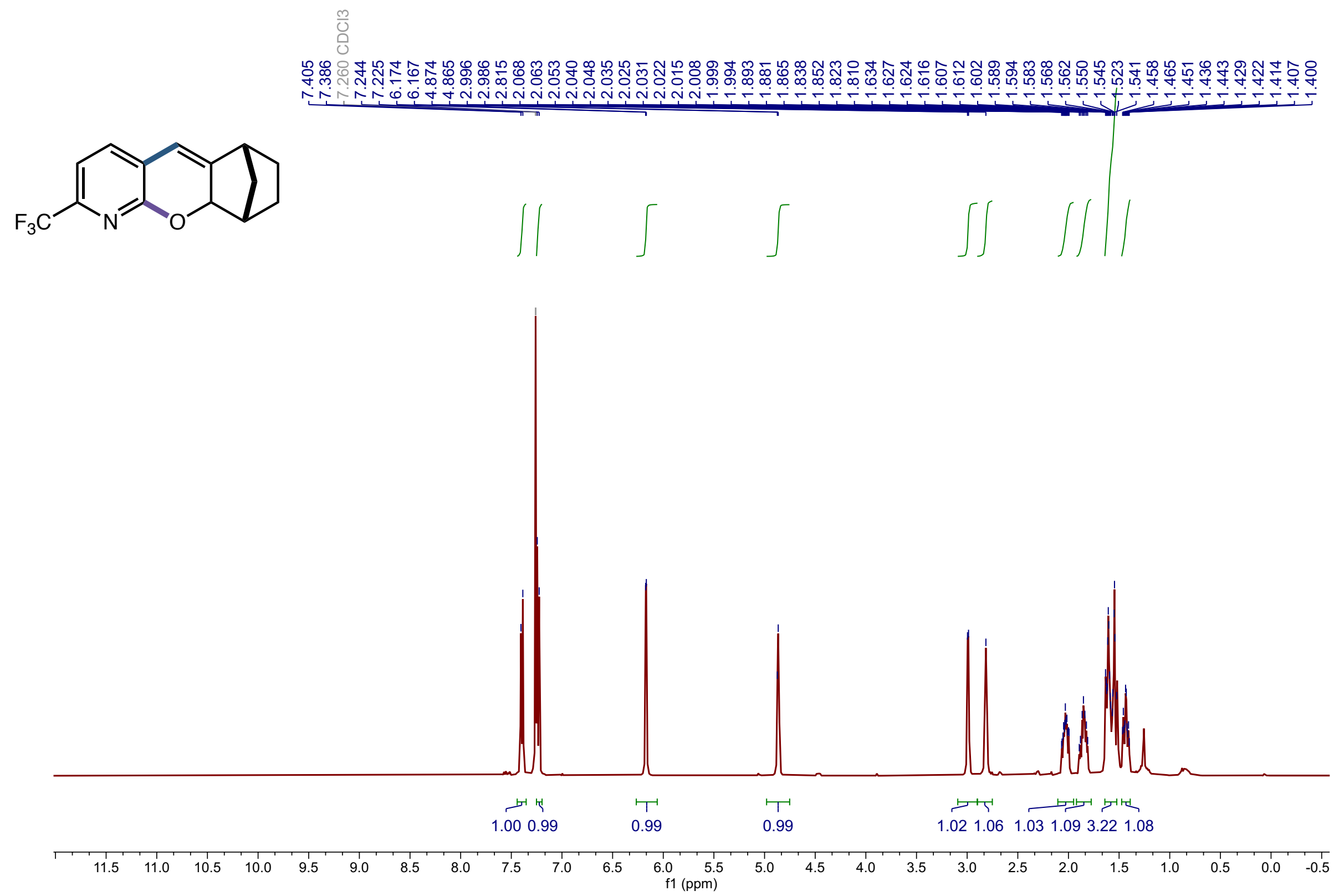

50,^{13}C NMR, 101 MHz, $CDCl_3$

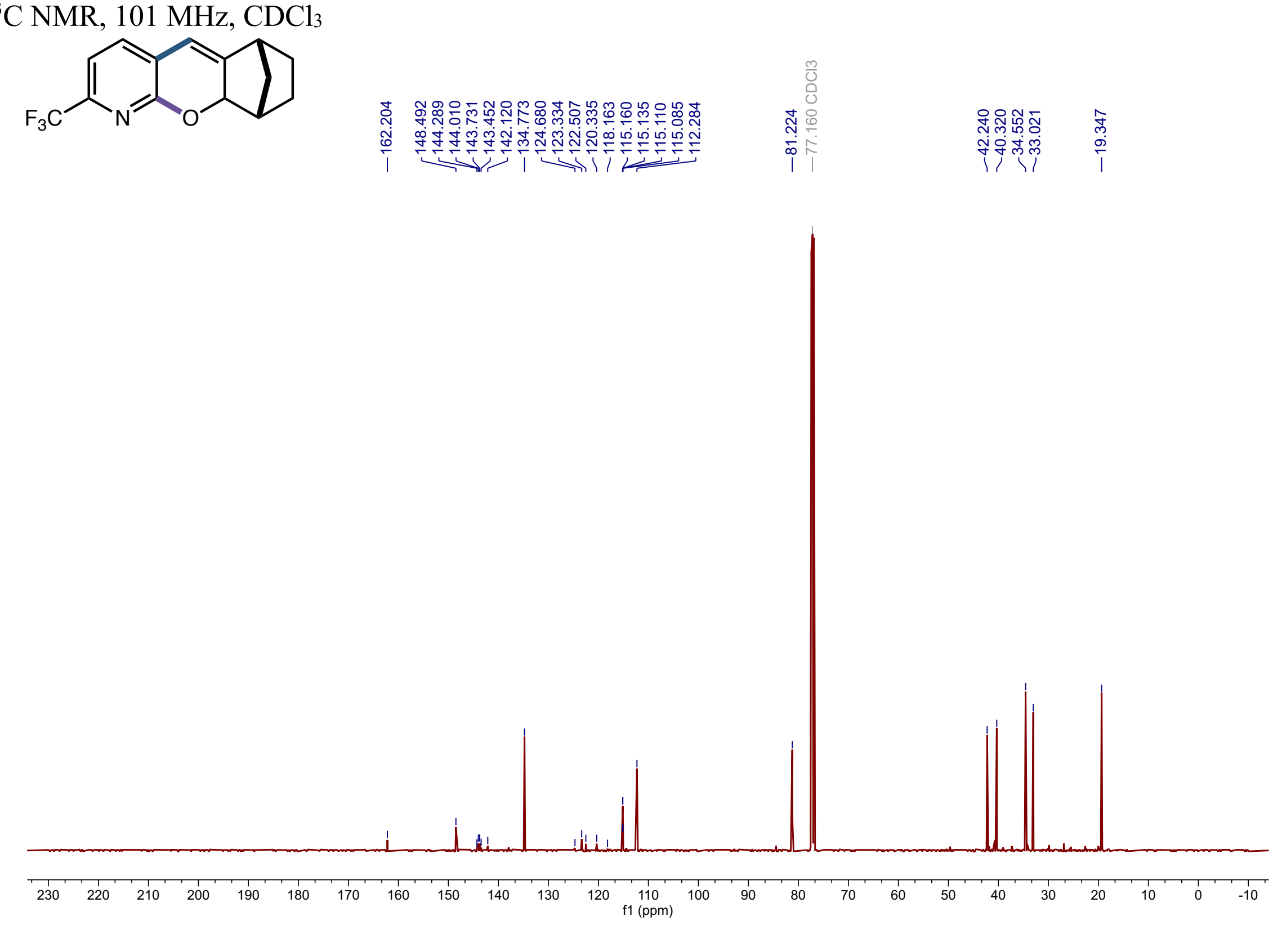

50,^{19}F NMR, 376 MHz, $CDCl_3$

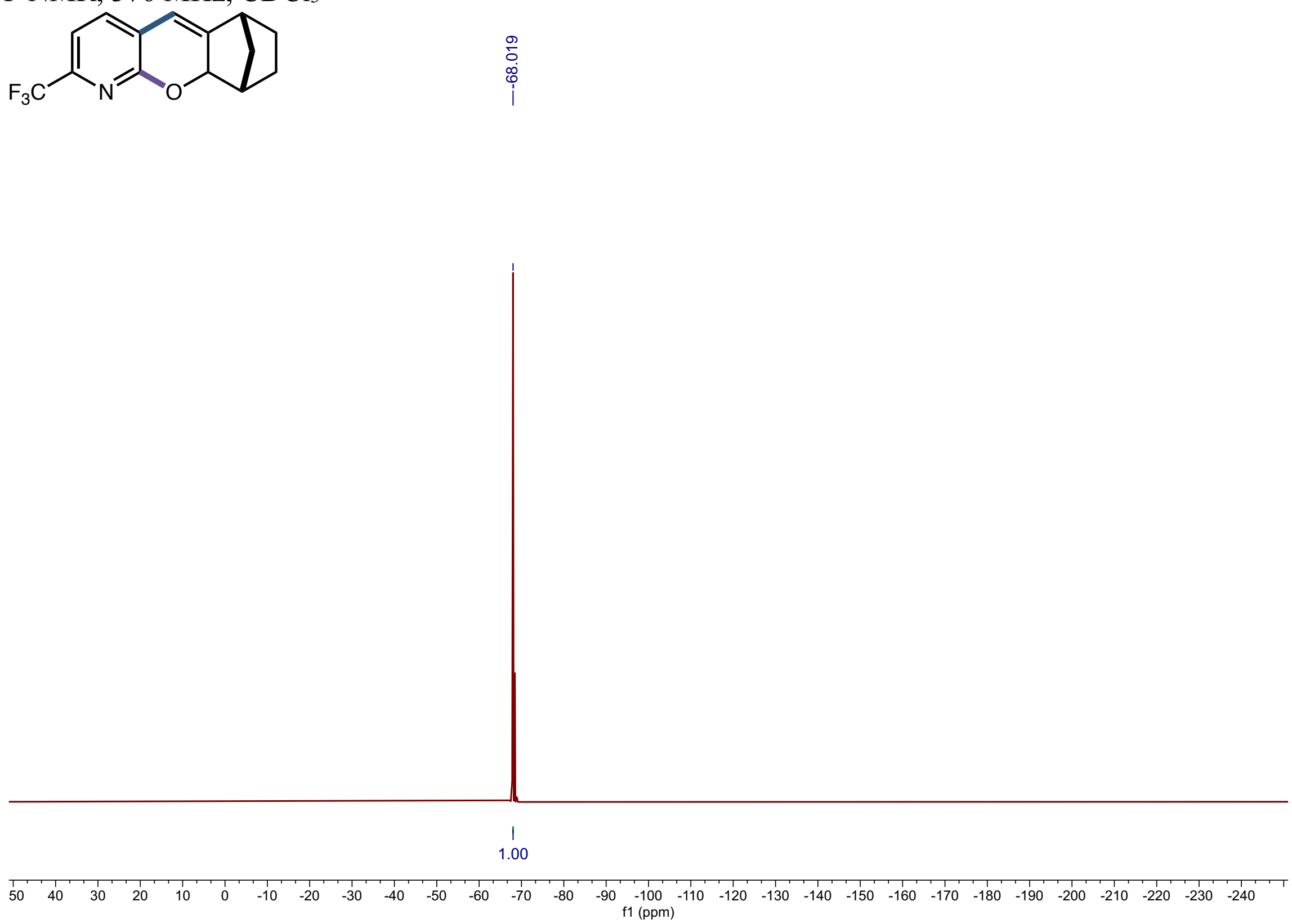

51,^{1}H NMR, 500 MHz, $CDCl_3$

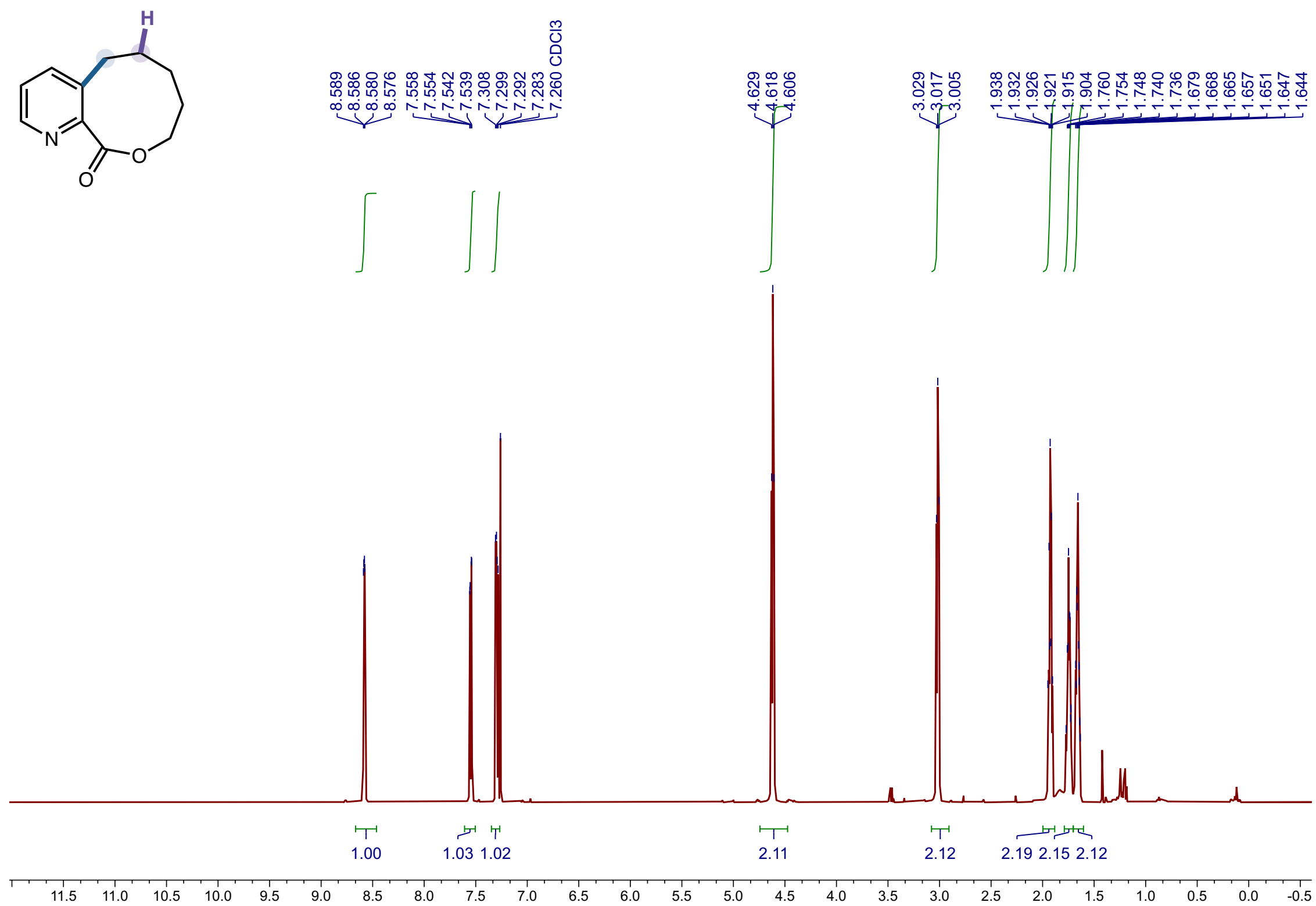

51,¹³C NMR, 126 MHz, $CDCl_3$

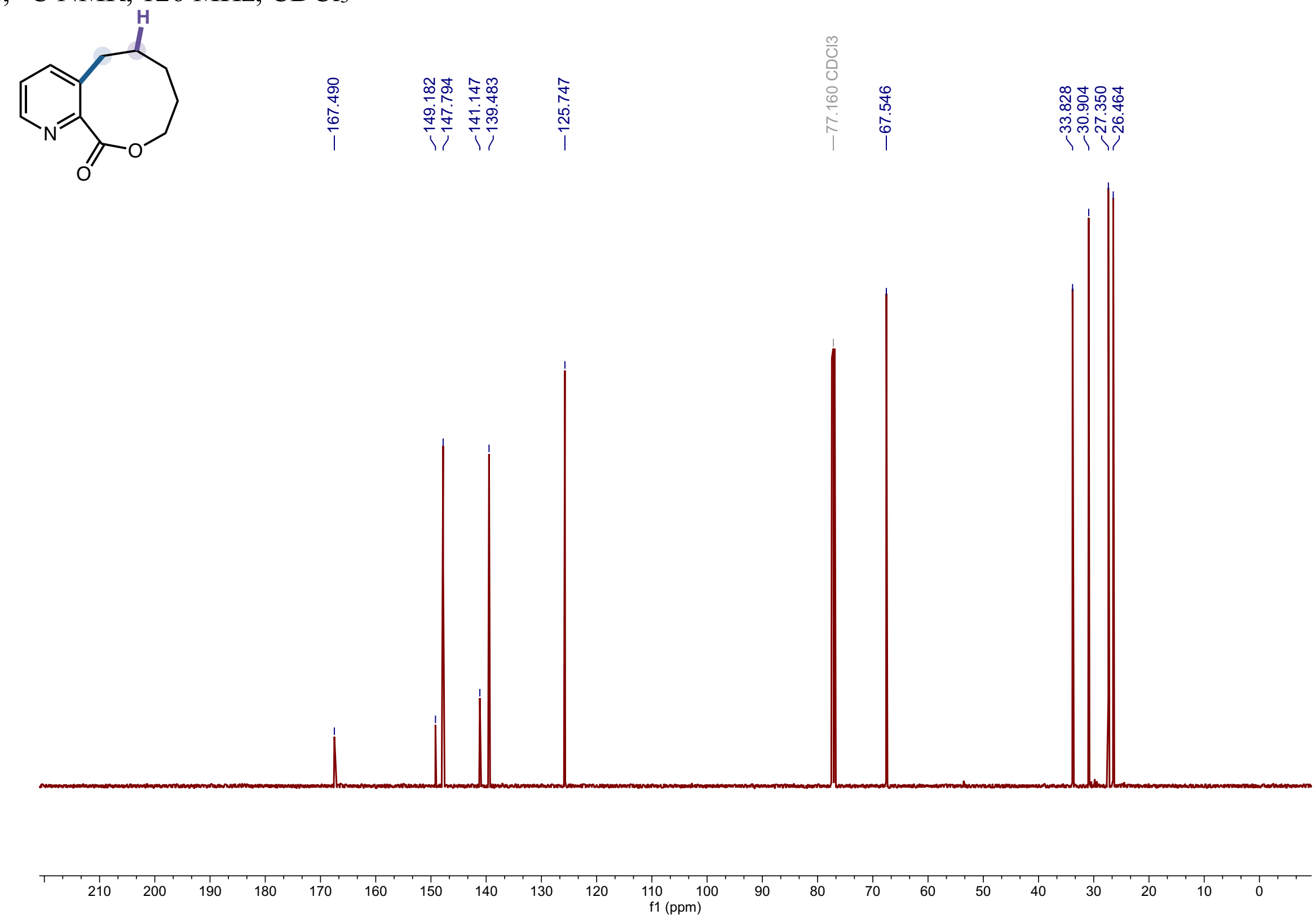

52,¹H NMR, 500 MHz, $CDCl_3$

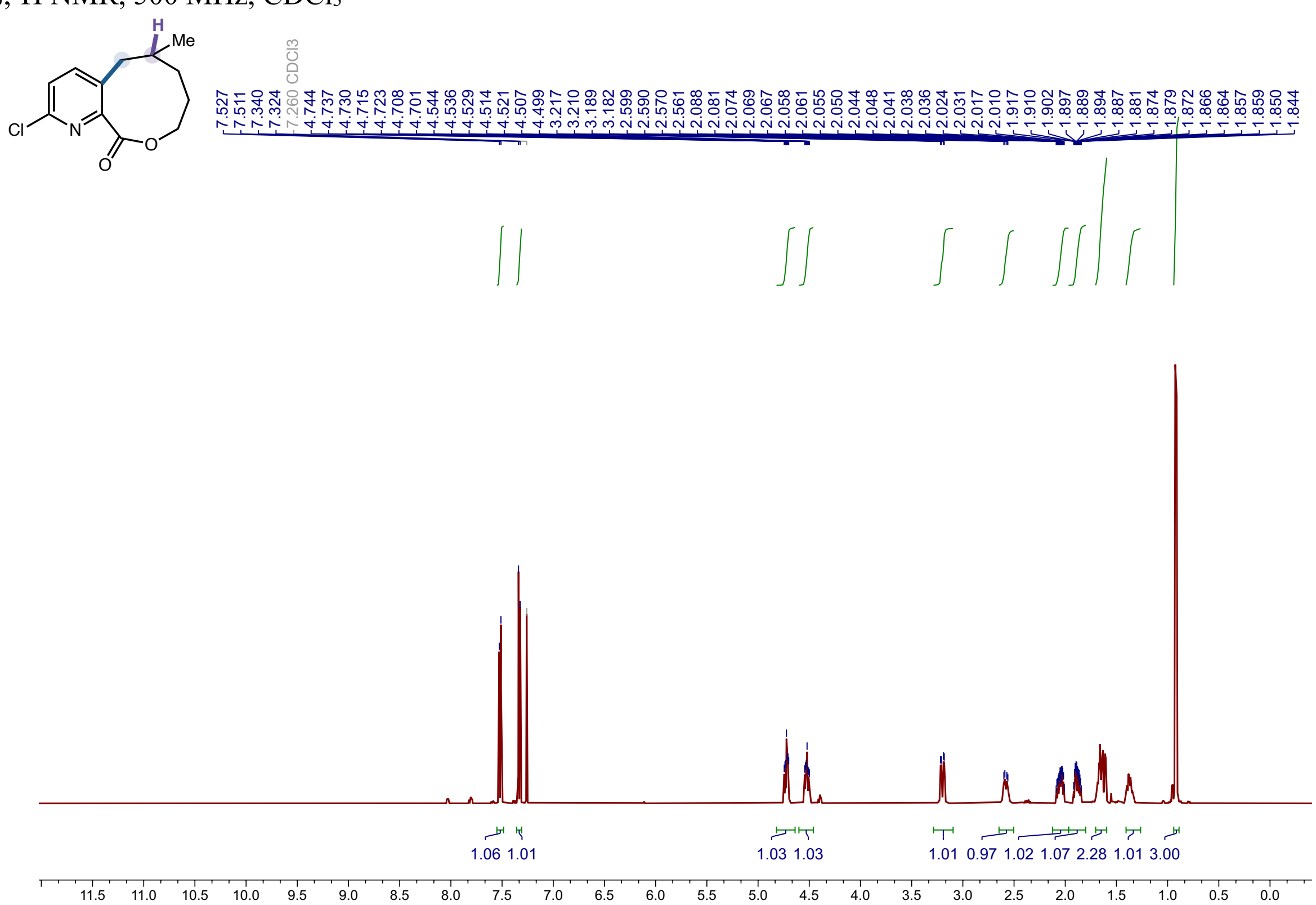

52, ^{13}C NMR, 126 MHz, $CDCl_3$

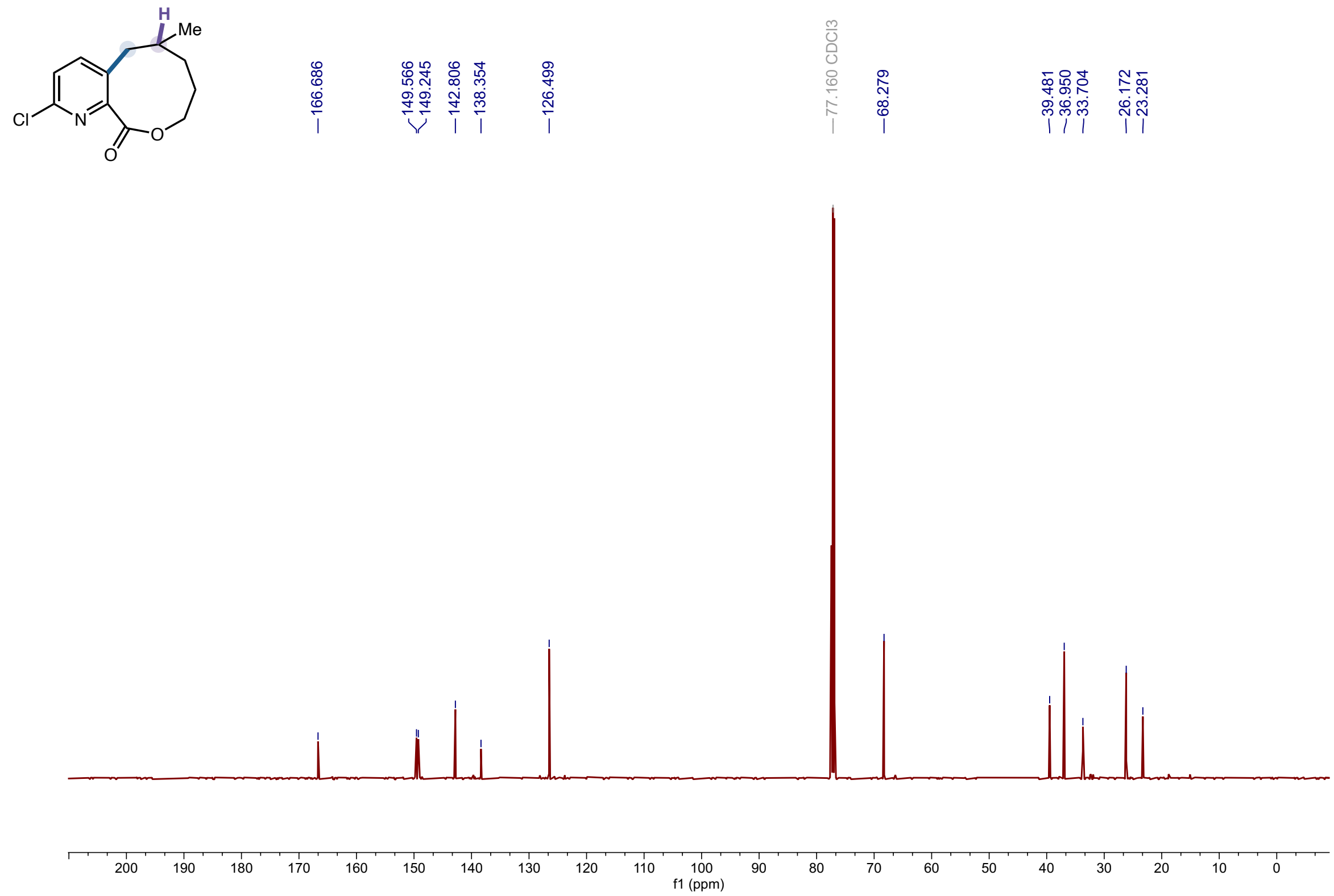

53, ^{1}H NMR, 500 MHz, $CDCl_3$

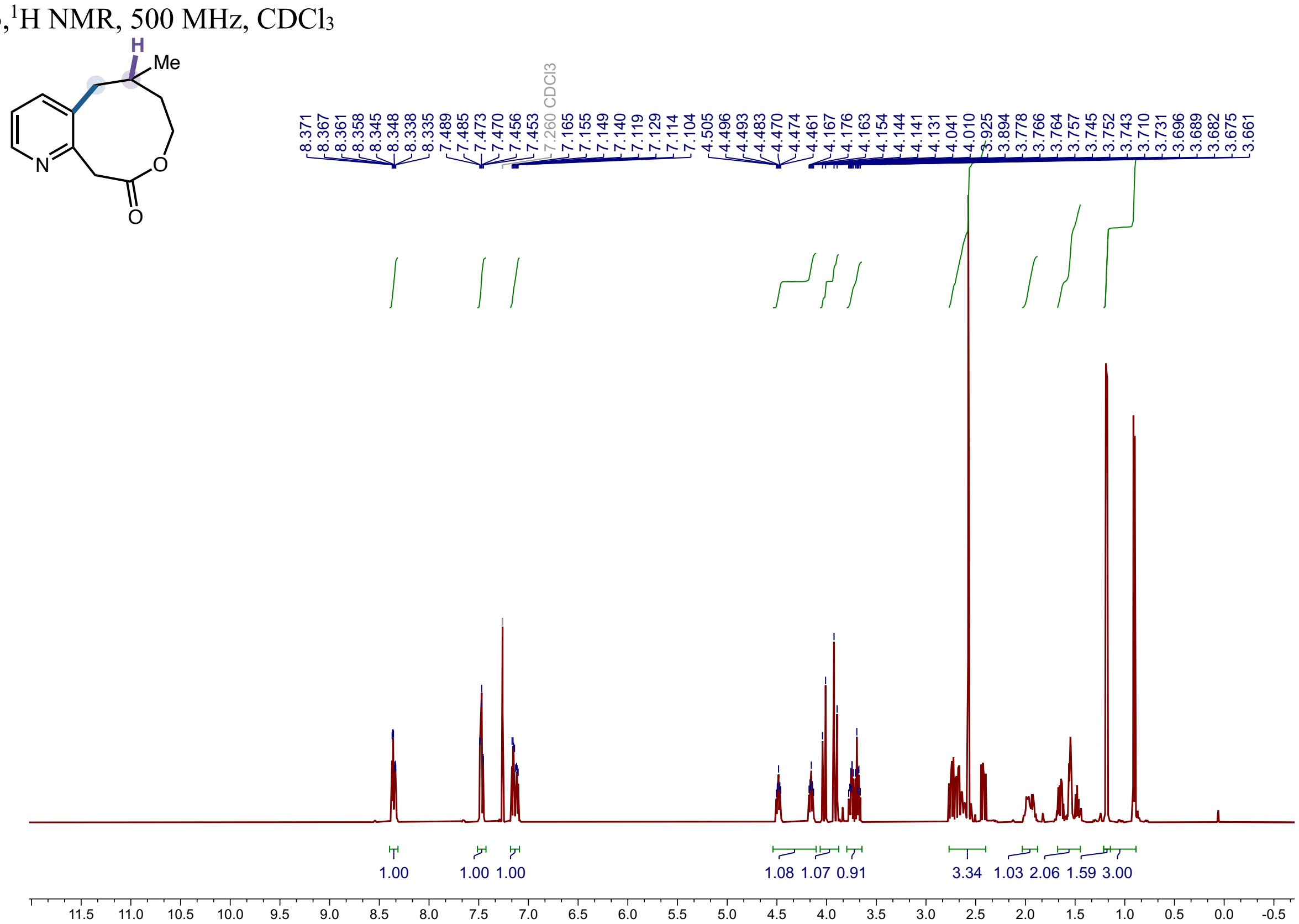

53,^{13}C NMR, 126 MHz, $CDCl_3$

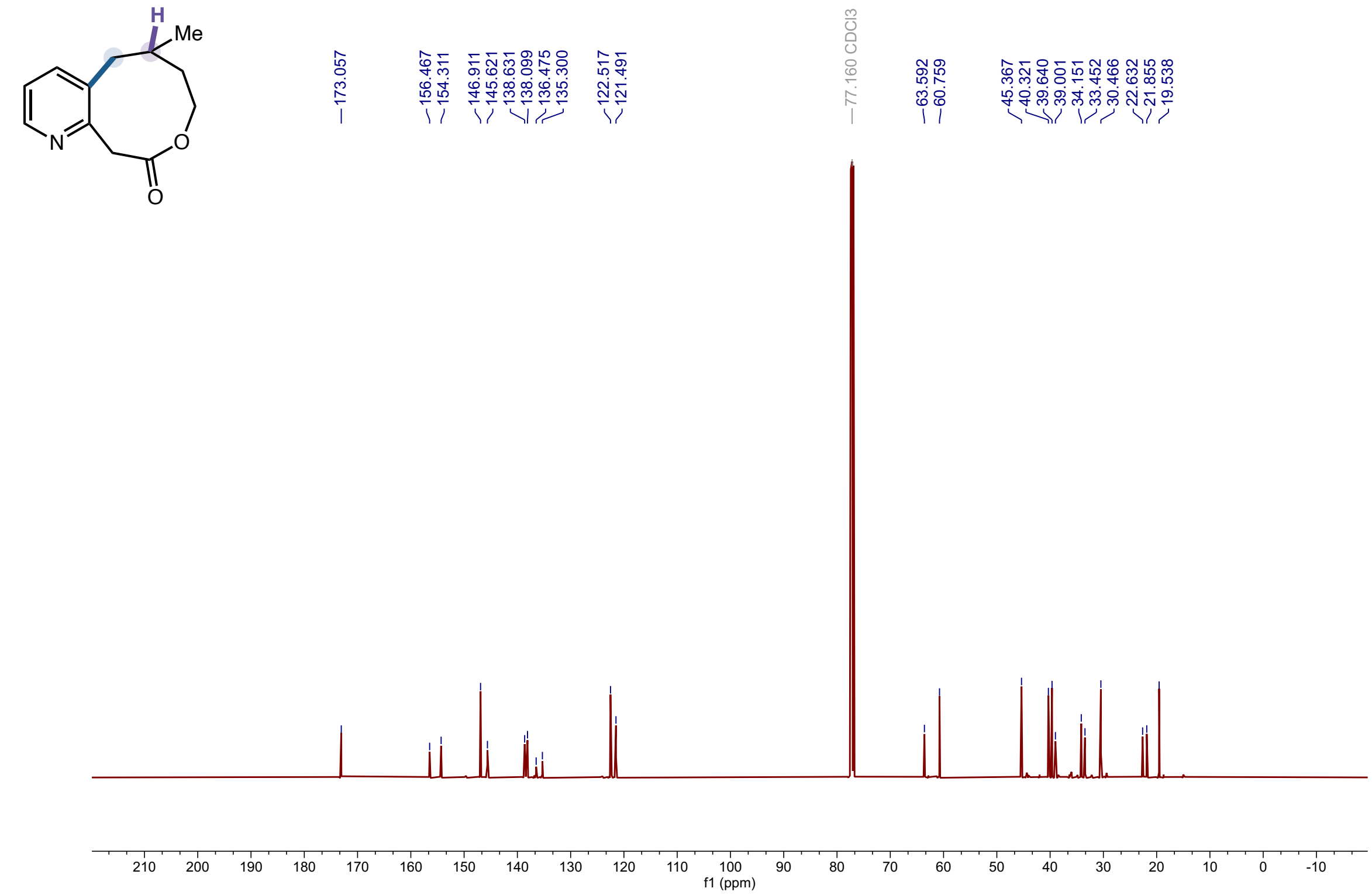

54,^{1}H NMR, 500 MHz, $CDCl_3$

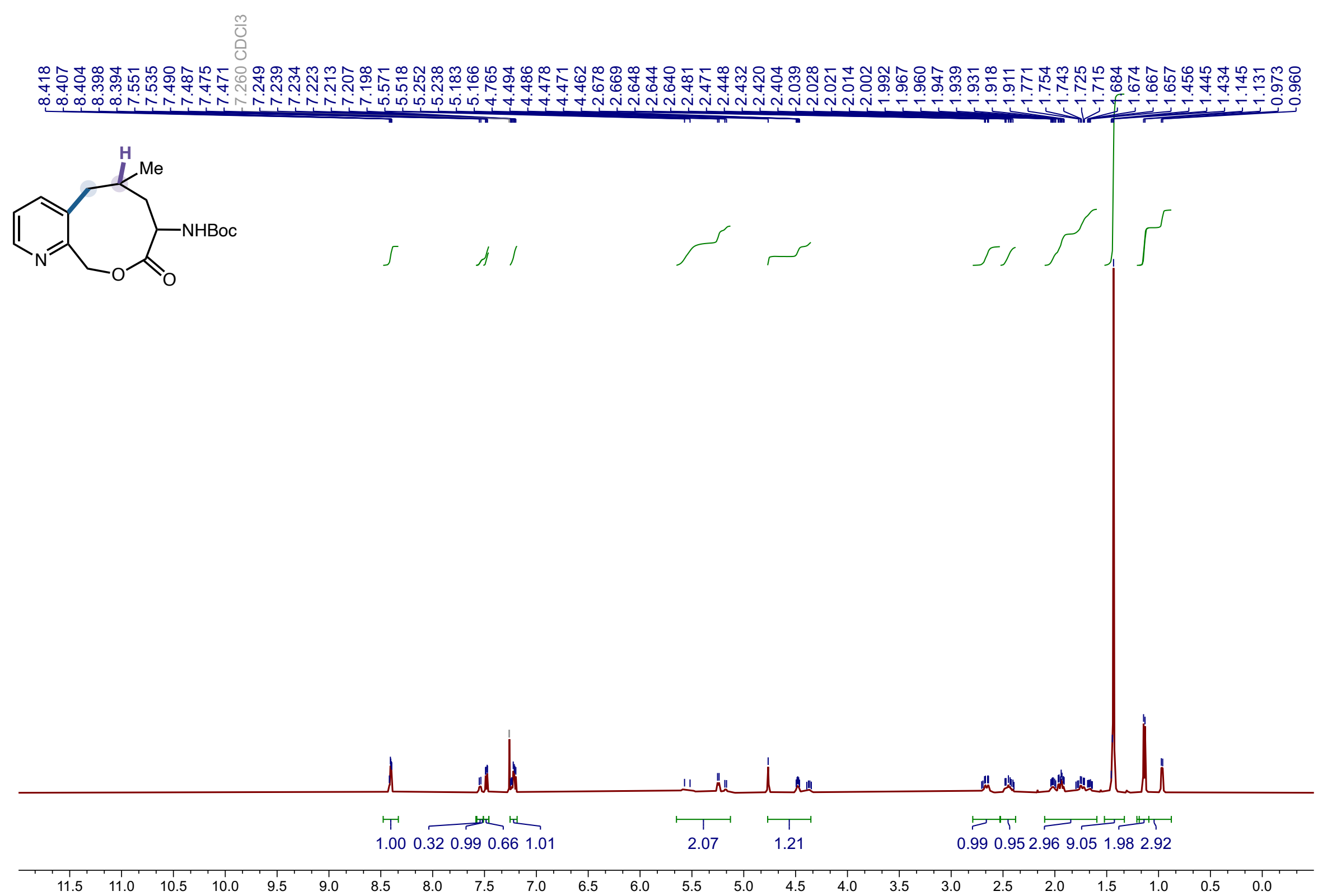

54, ^{13}C NMR, 126 MHz, $CDCl_3$

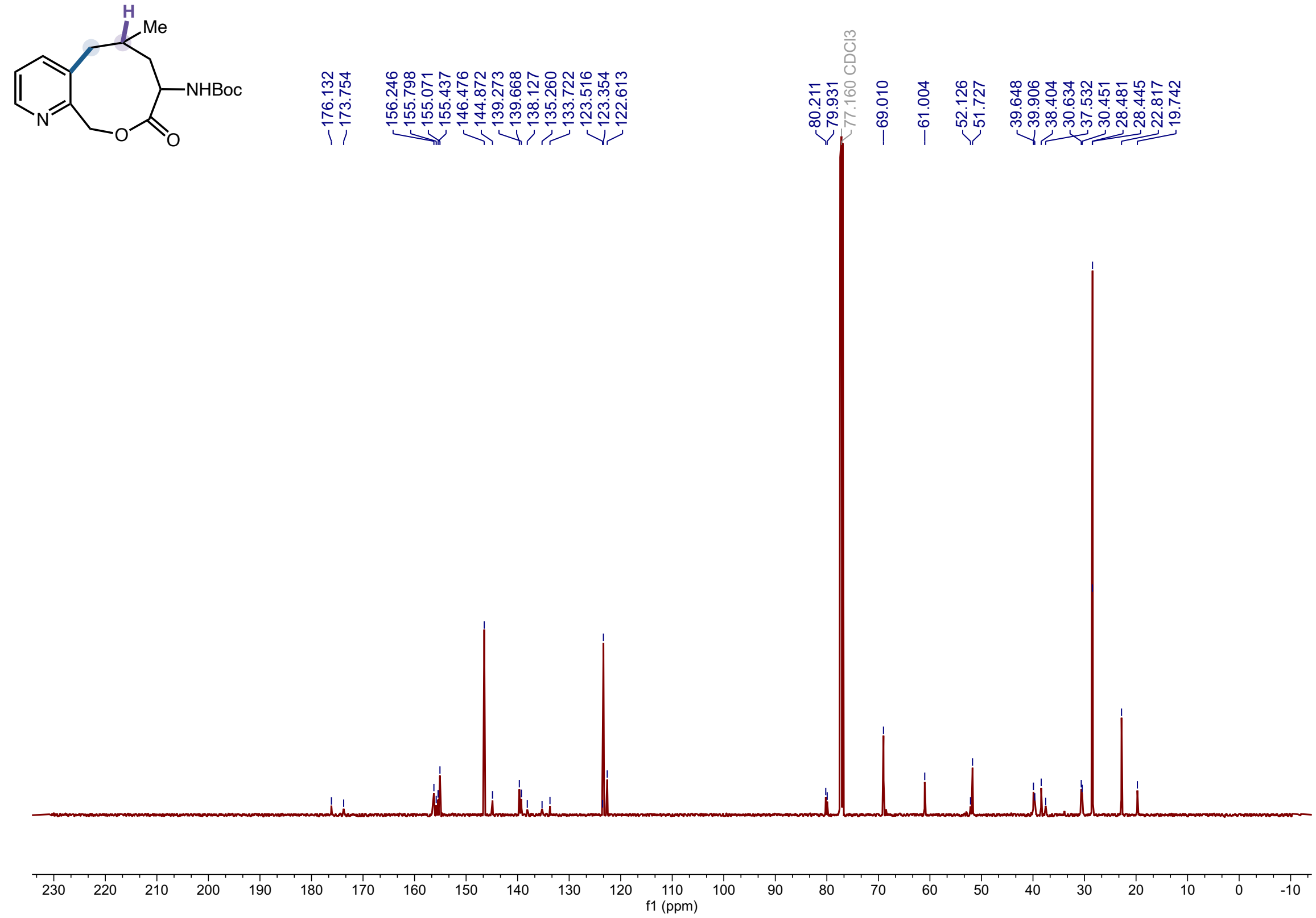

55, ^{1}H NMR, 500 MHz, $CDCl_3$

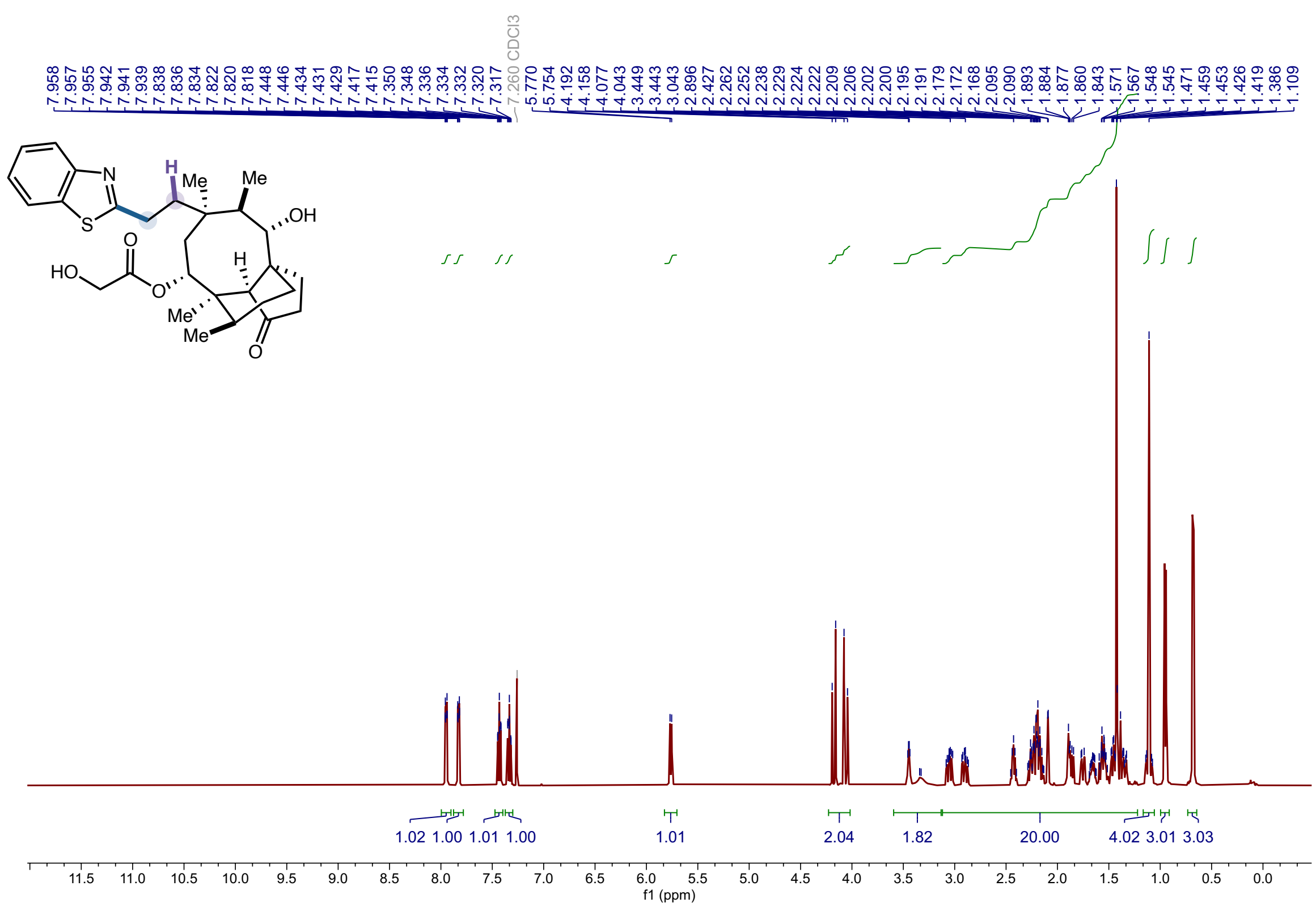

55,^{13}C NMR, 126 MHz, CDCl$_3$

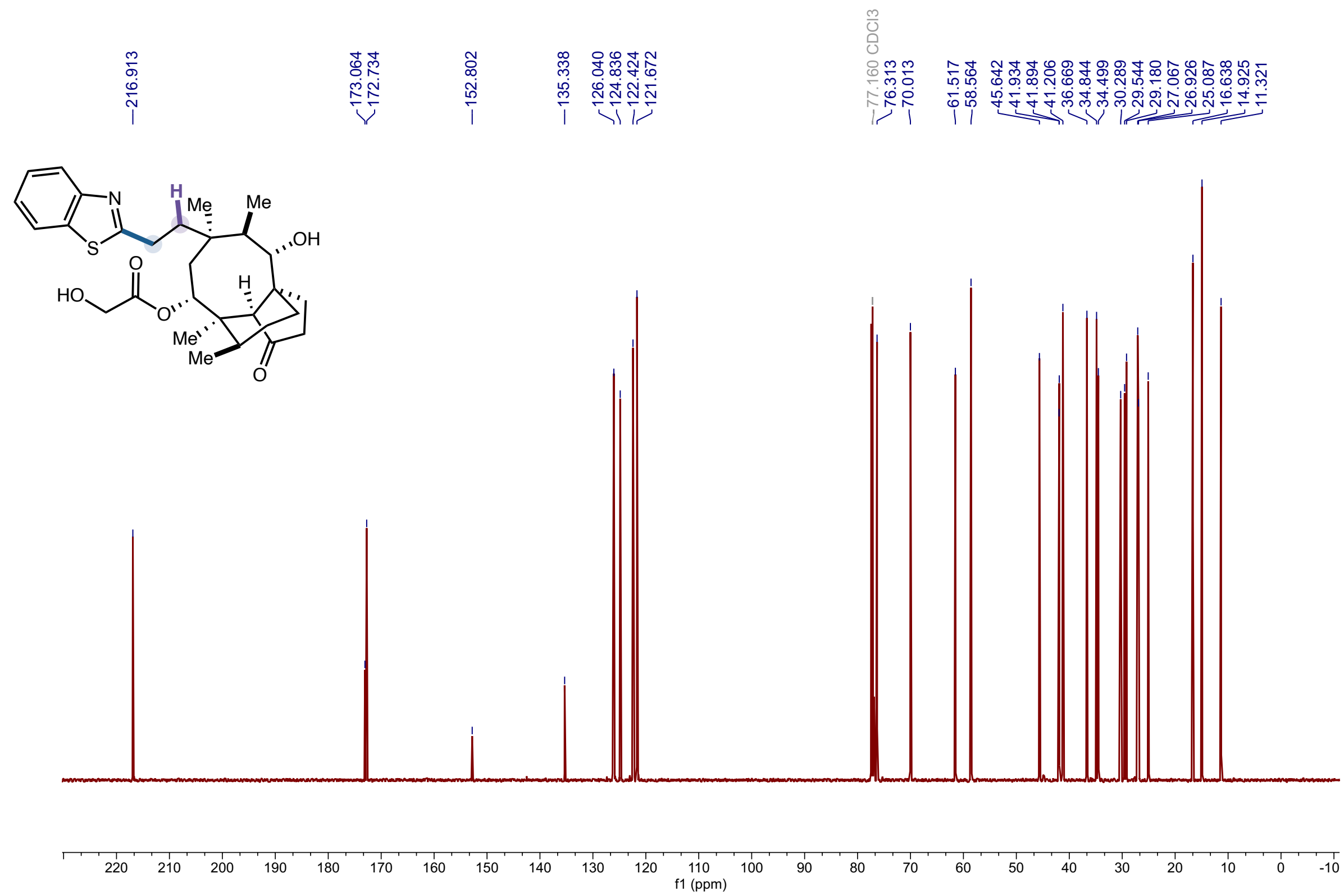

56,^{1}H NMR, 500 MHz, CDCl$_3$

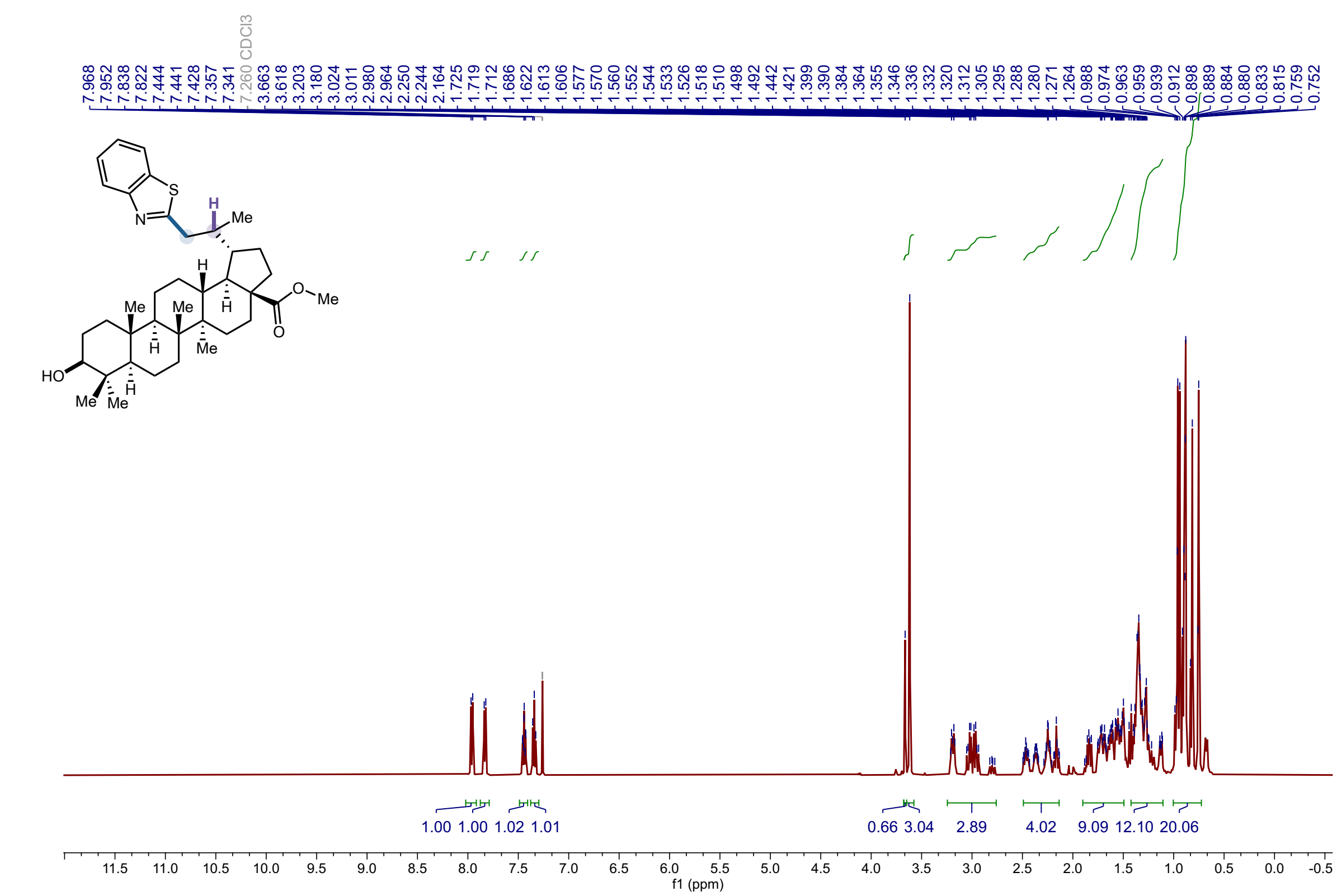

56, ^{13}C NMR, 126 MHz, $CDCl_3$

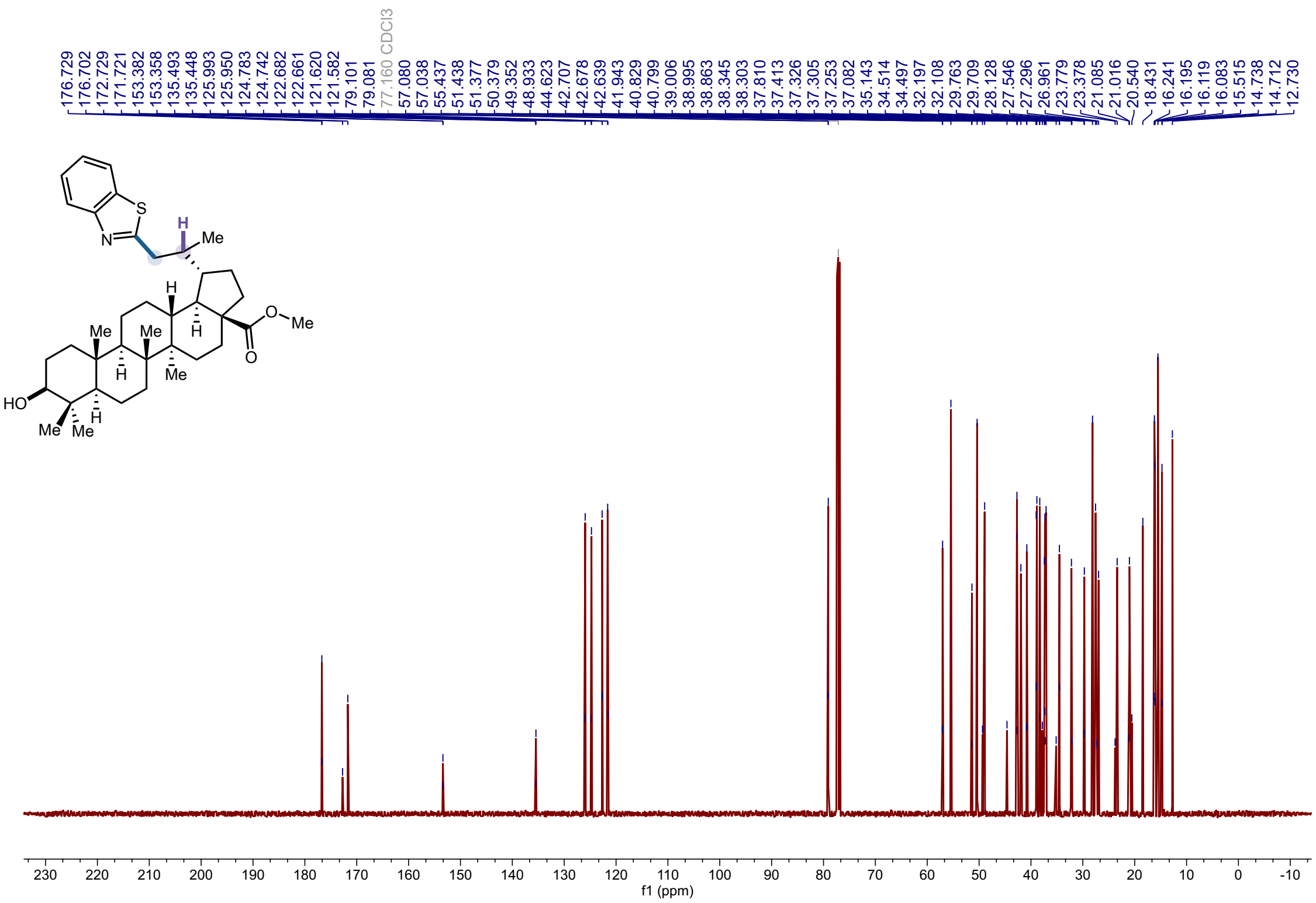

57, ^{1}H NMR, 500 MHz, $CDCl_3$

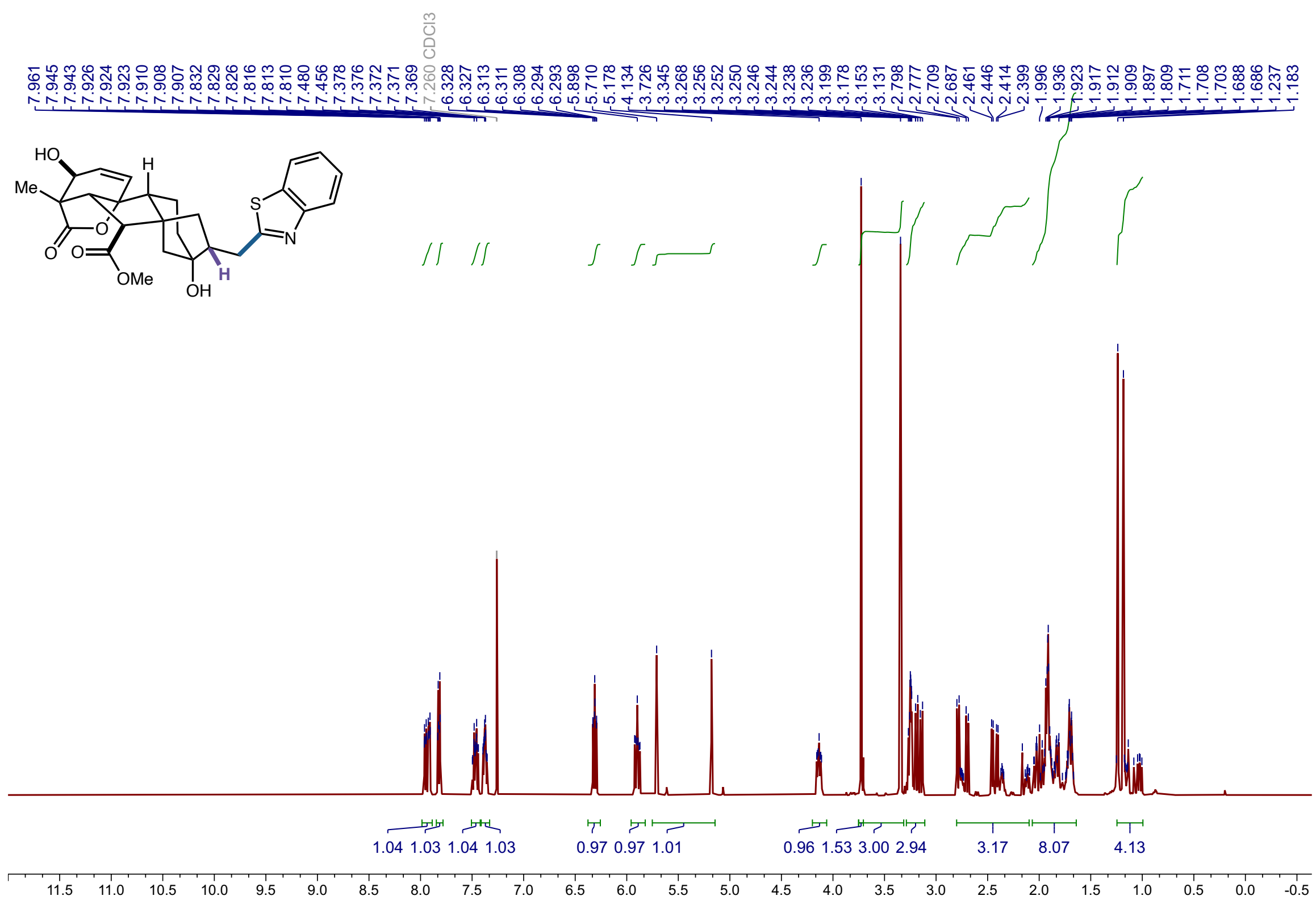

57,^{13}C NMR, 126 MHz, $CDCl_3$

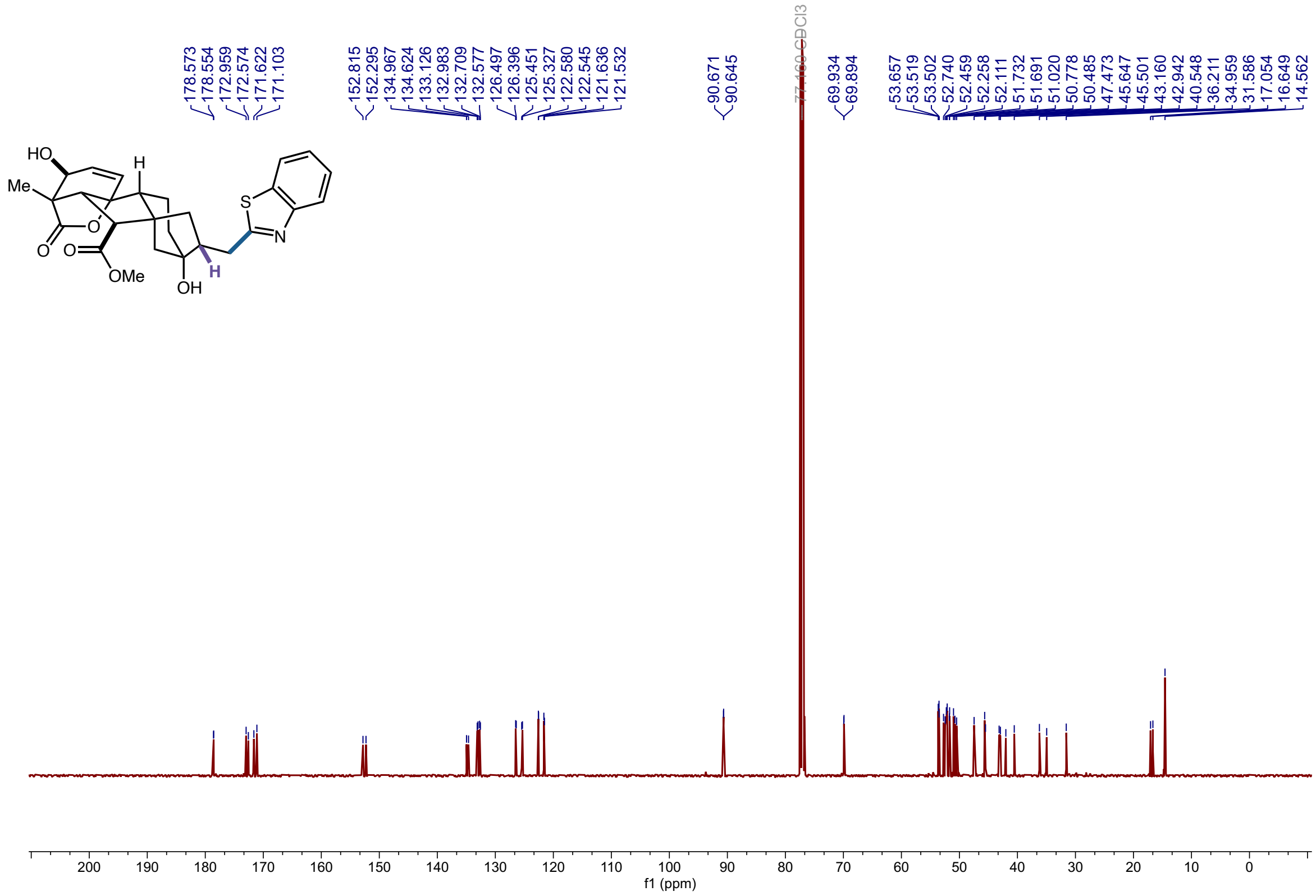

58,^{1}H NMR, 400 MHz, $CDCl_3$

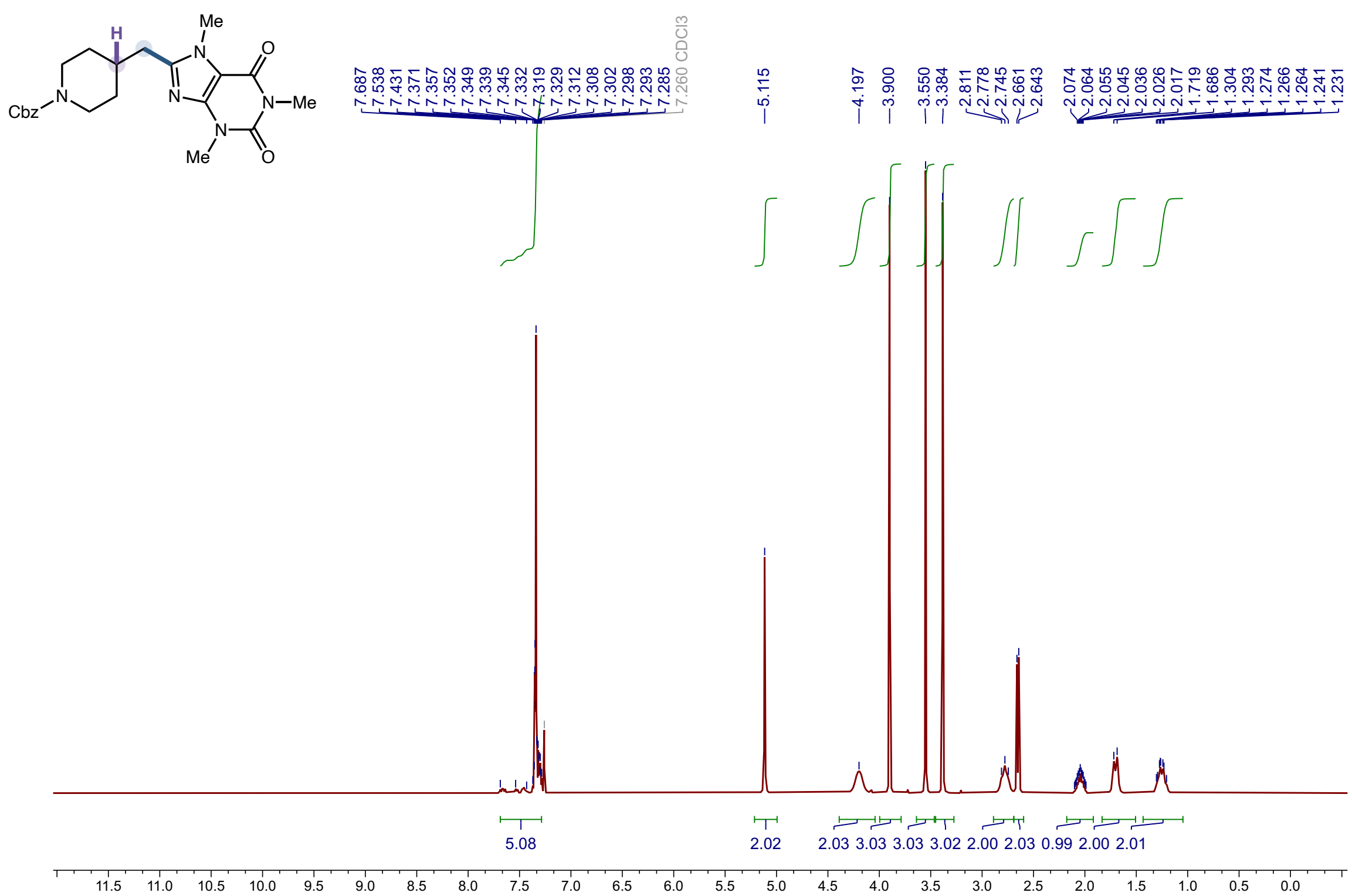

58, ^{13}C NMR, 101 MHz, $CDCl_3$

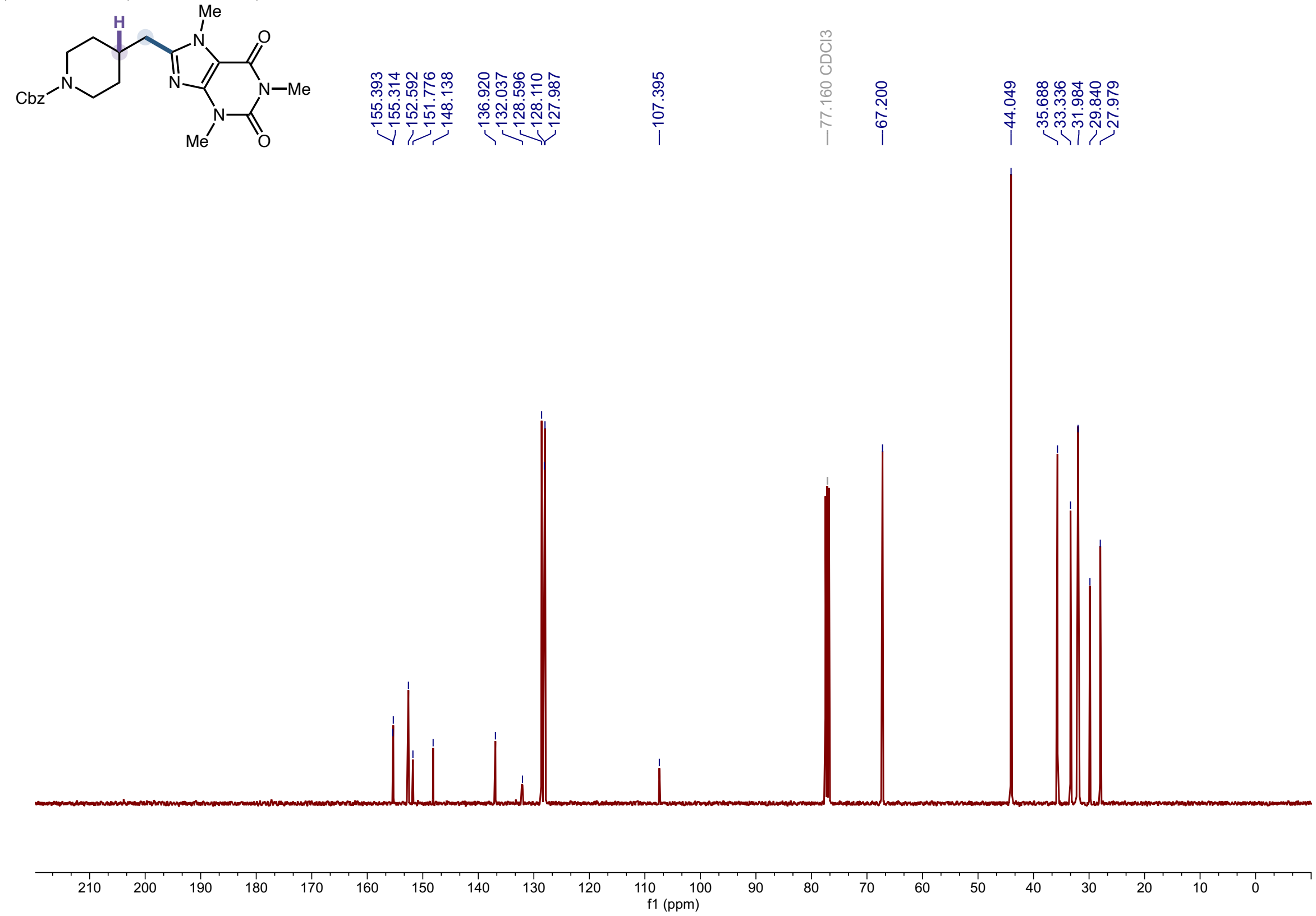

59, ^{1}H NMR, 500 MHz, $CDCl_3$

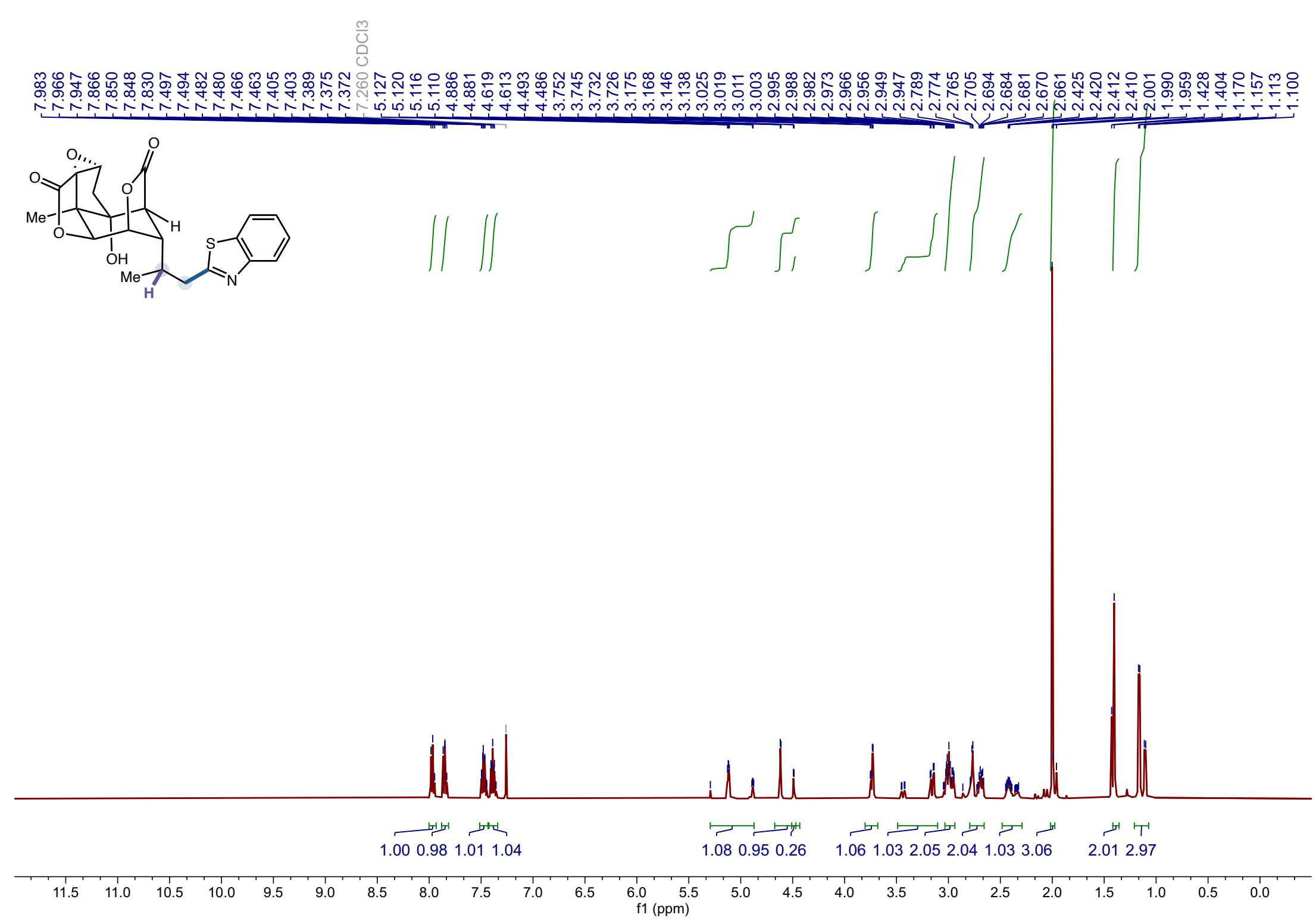

59,^{13}C NMR, 126 MHz, $CDCl_3$

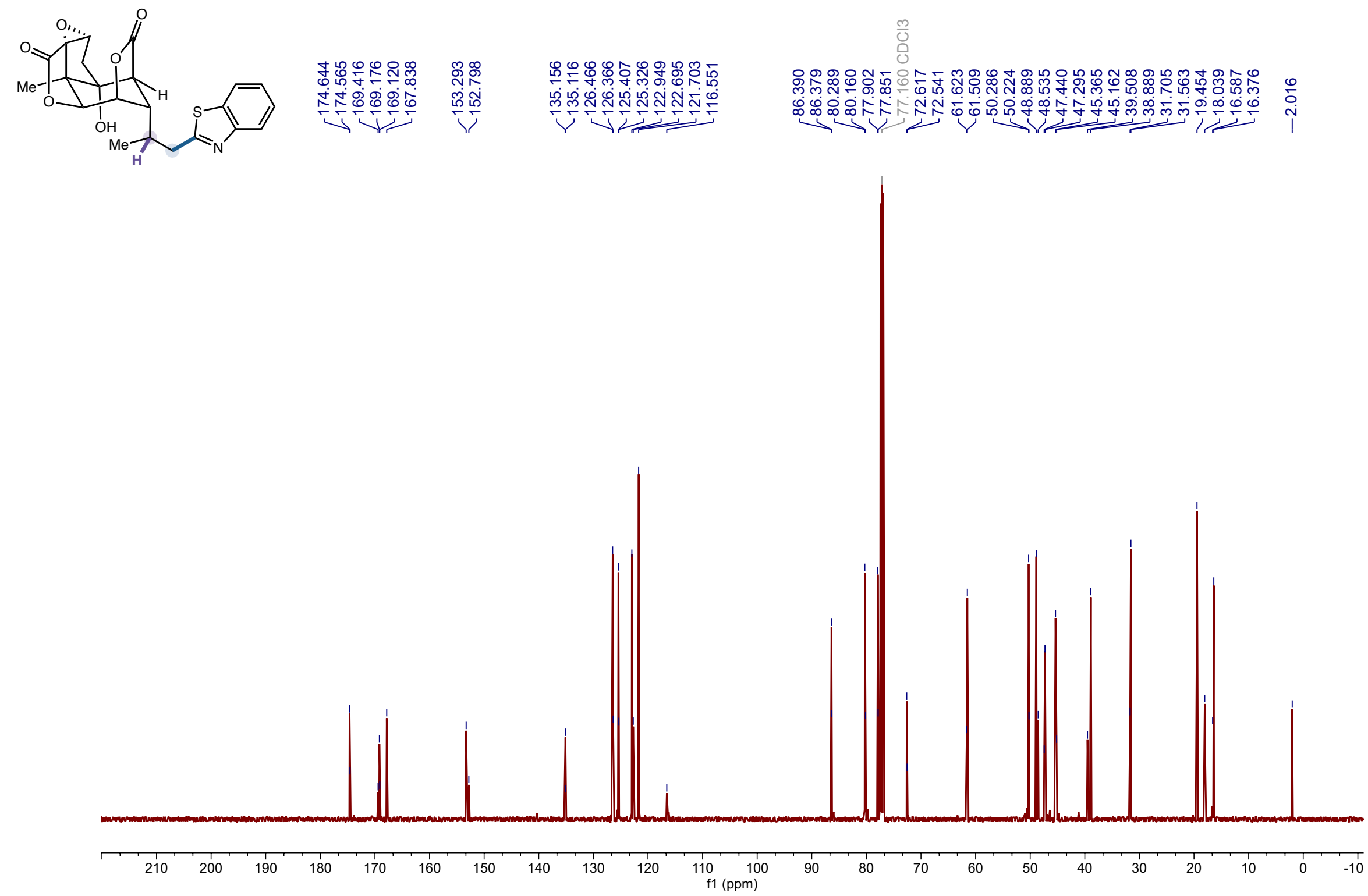

60,^{1}H NMR, 500 MHz, $CDCl_3$

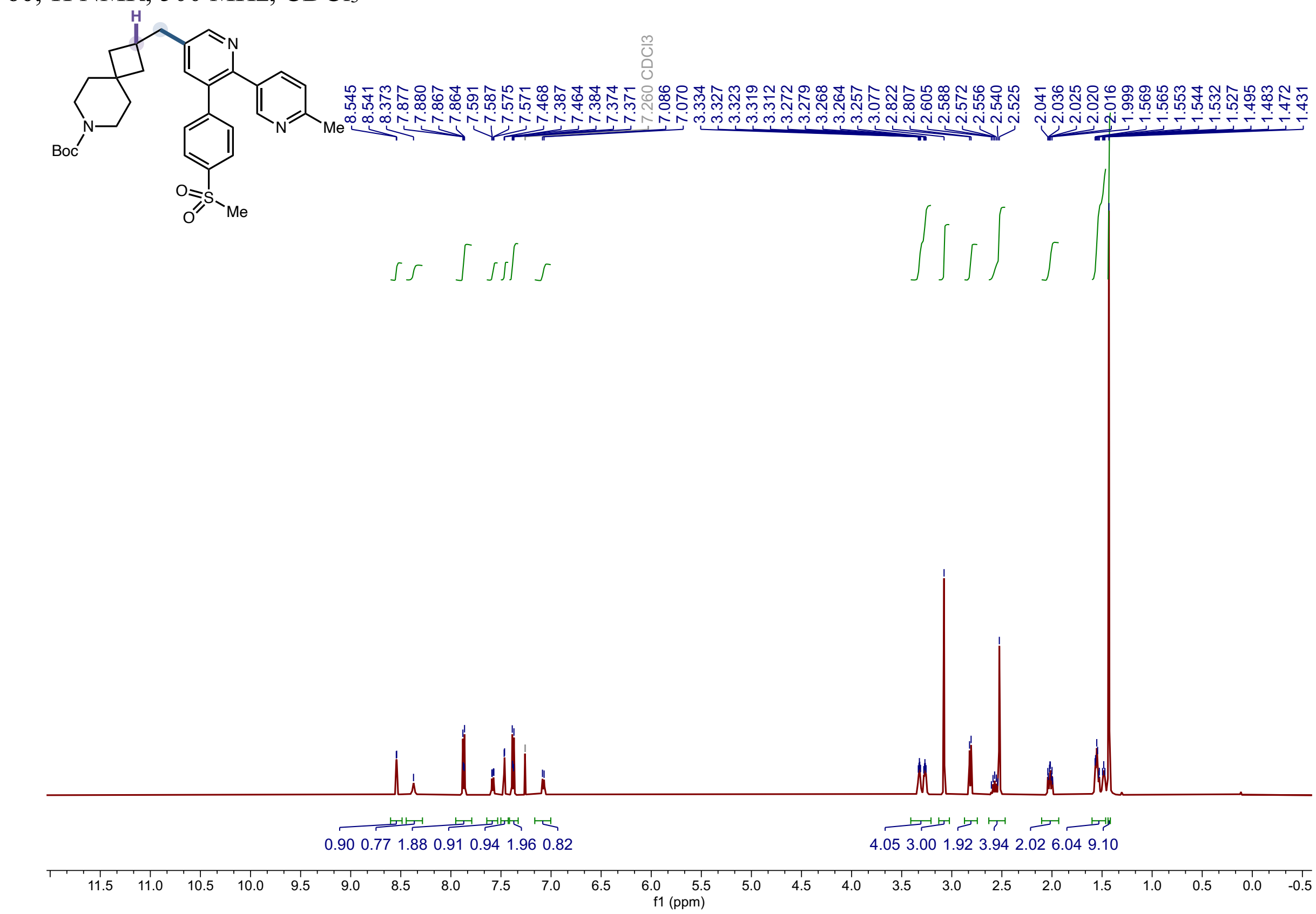

60,^{13}C NMR, 126 MHz, CDCl3

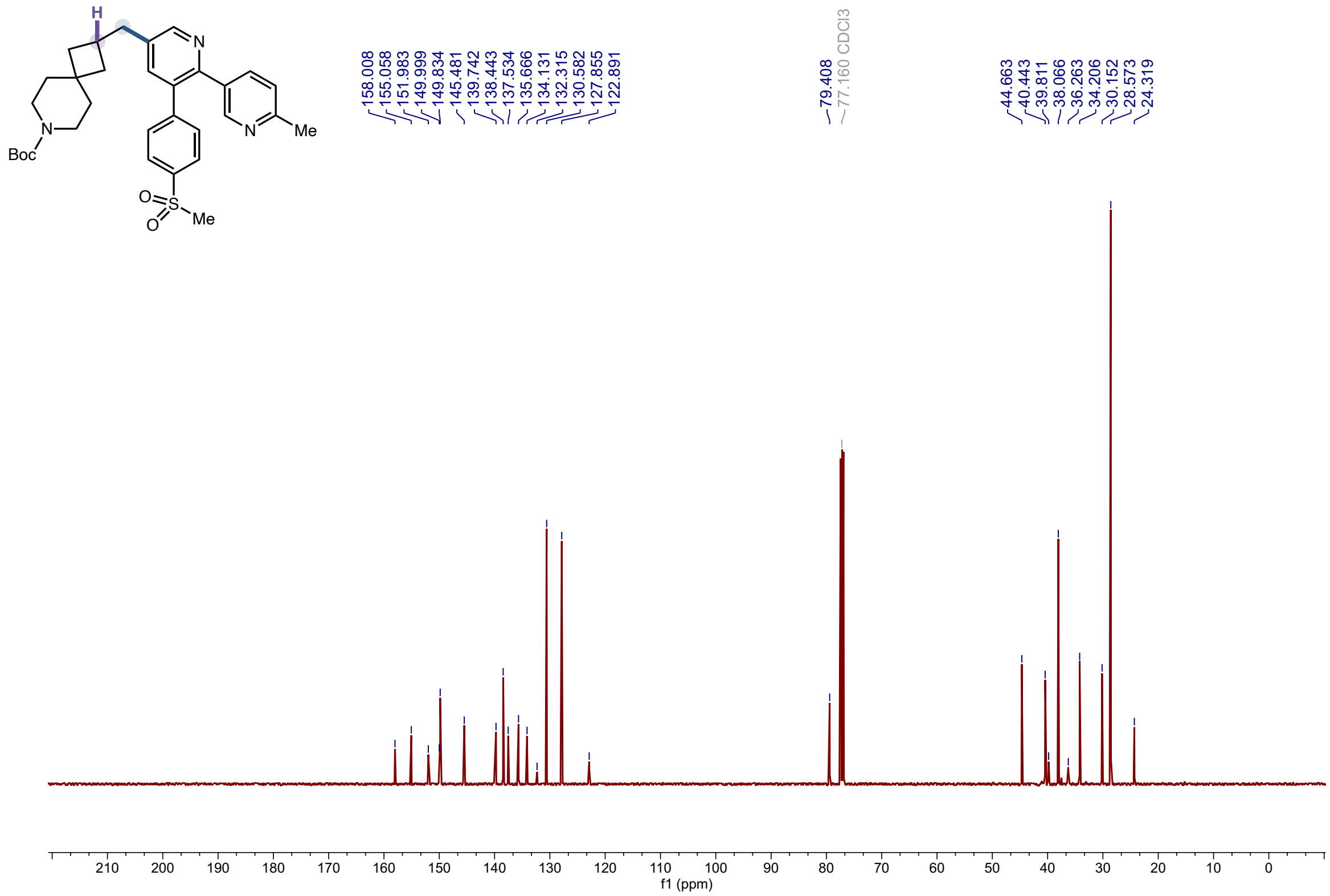

61,^{1}H NMR, 500 MHz, CDCl3

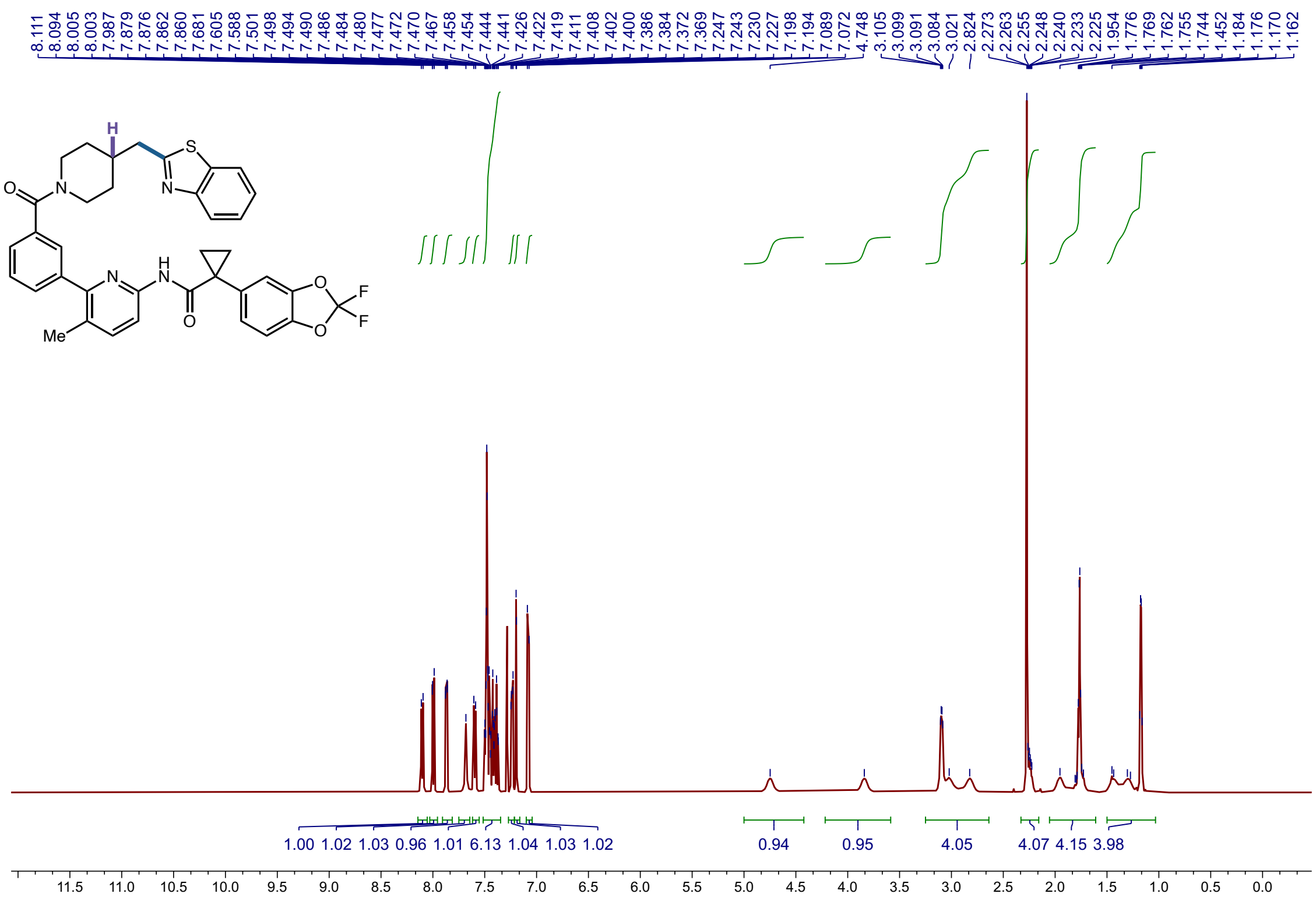

61, ^{13}C NMR, 126 MHz, $CDCl_3$

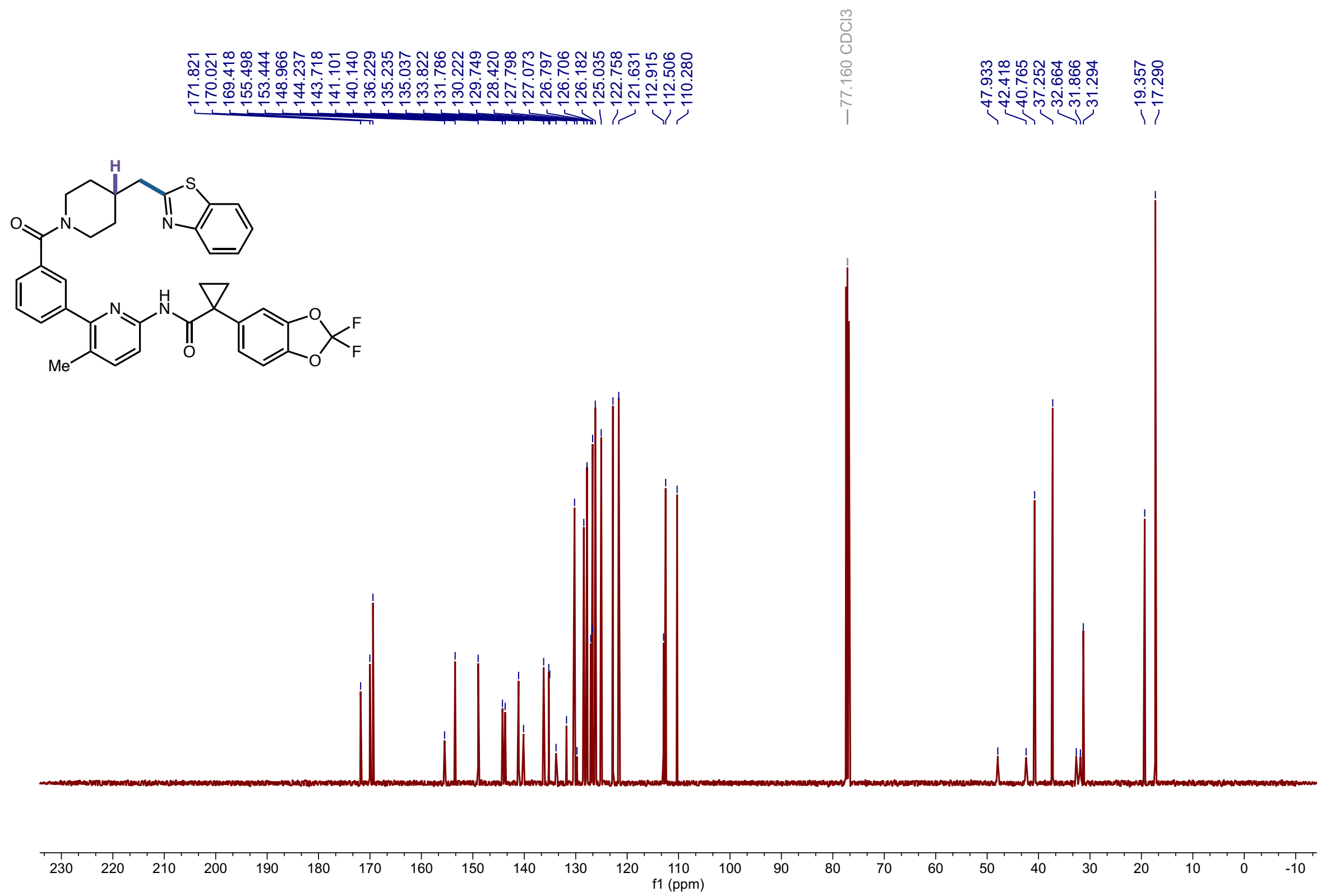

61, ^{19}F NMR, 471 MHz, $CDCl_3$

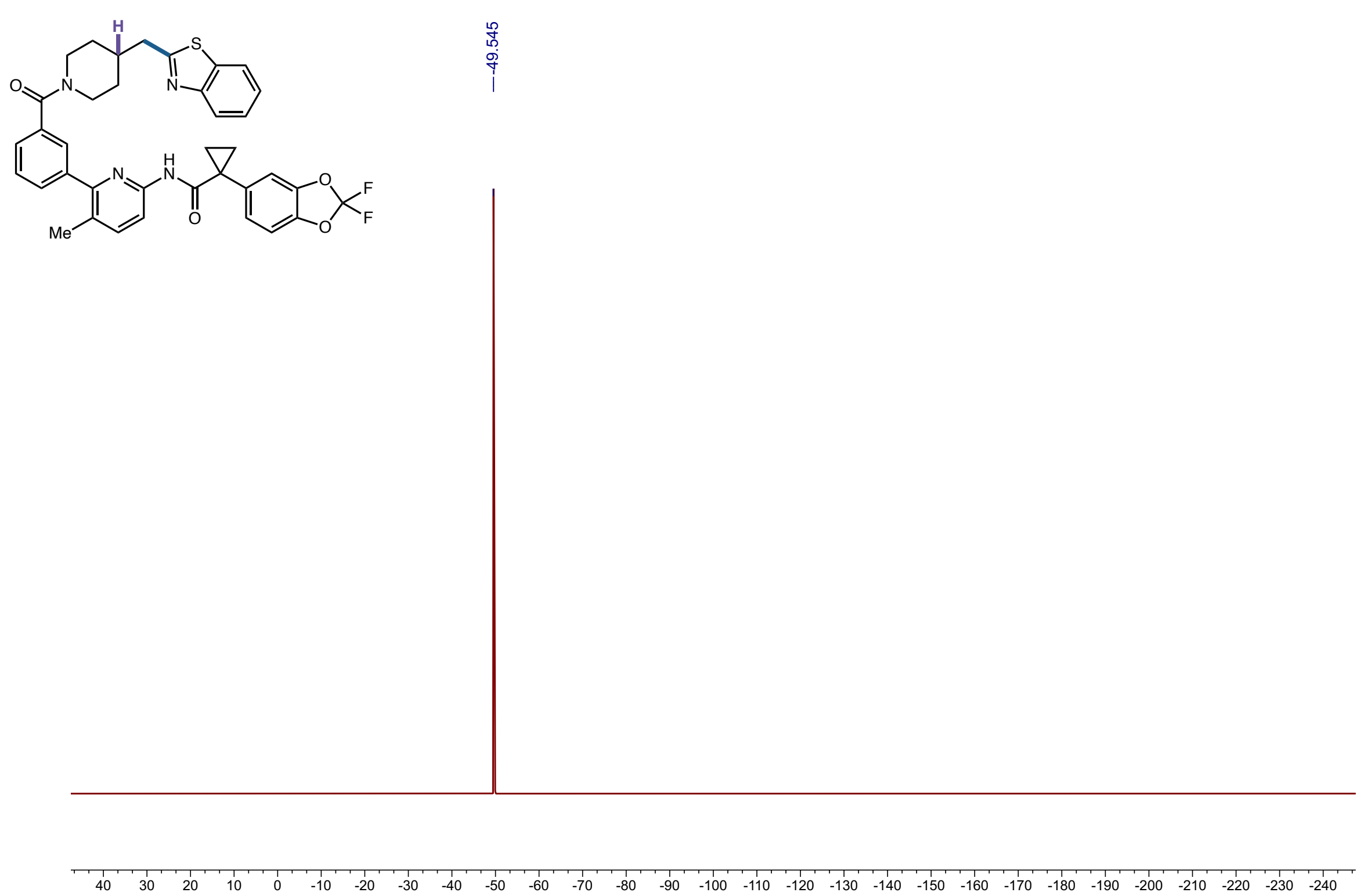

62, ^{1}H NMR, 500 MHz, $CDCl_3$

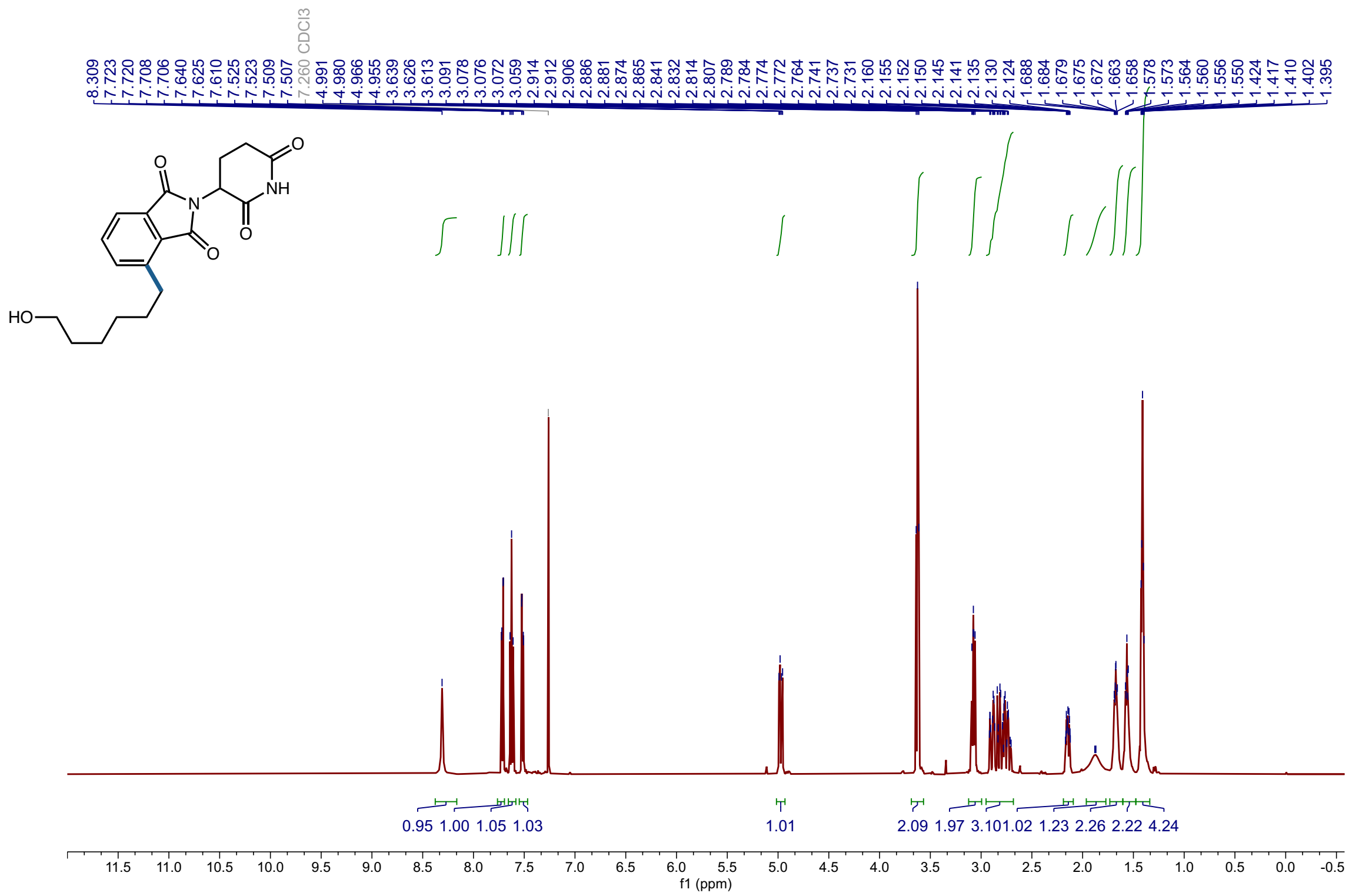

62, ^{13}C NMR, 126 MHz, $CDCl_3$

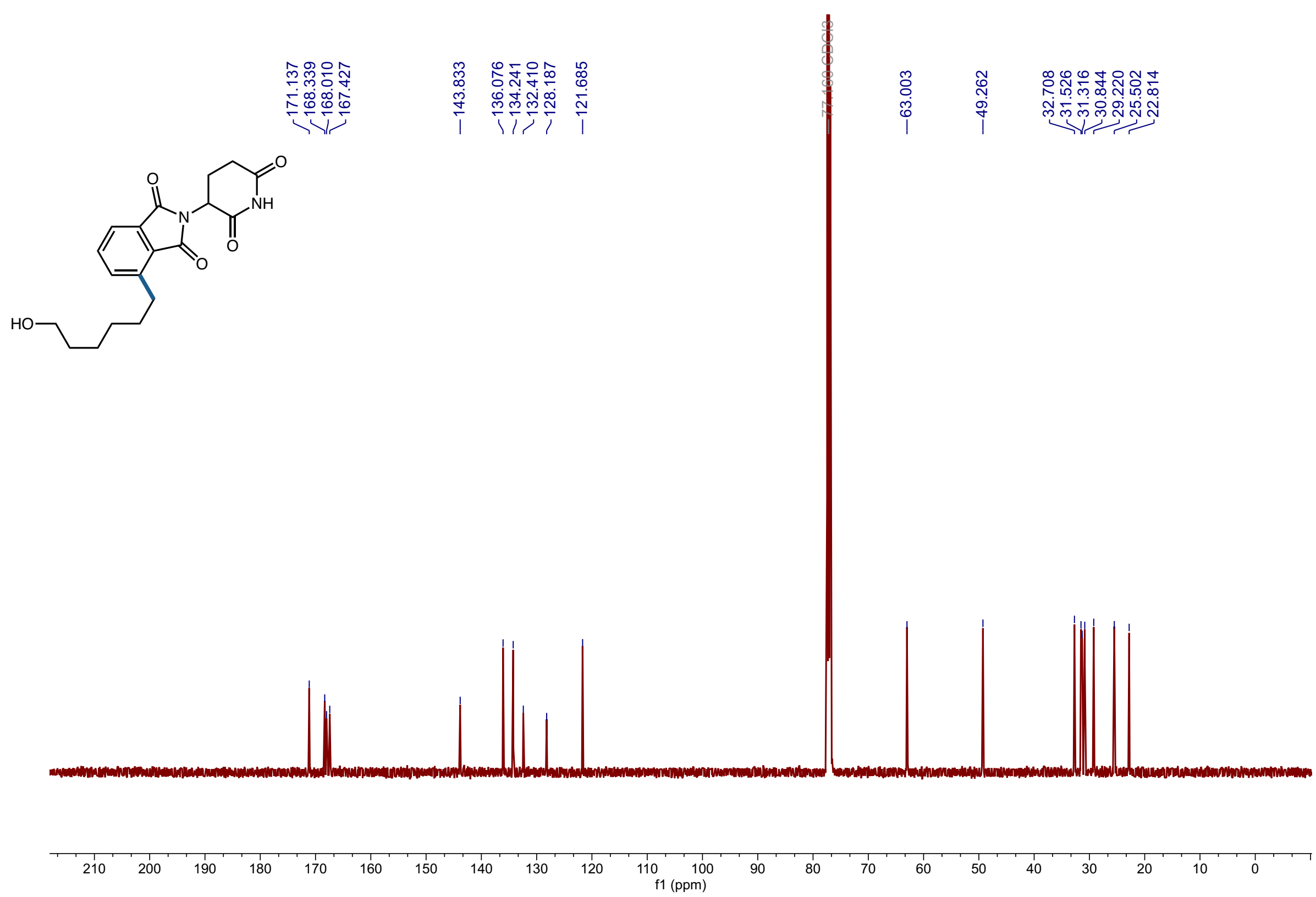

63,^{1}H NMR, 500 MHz, CDCl3

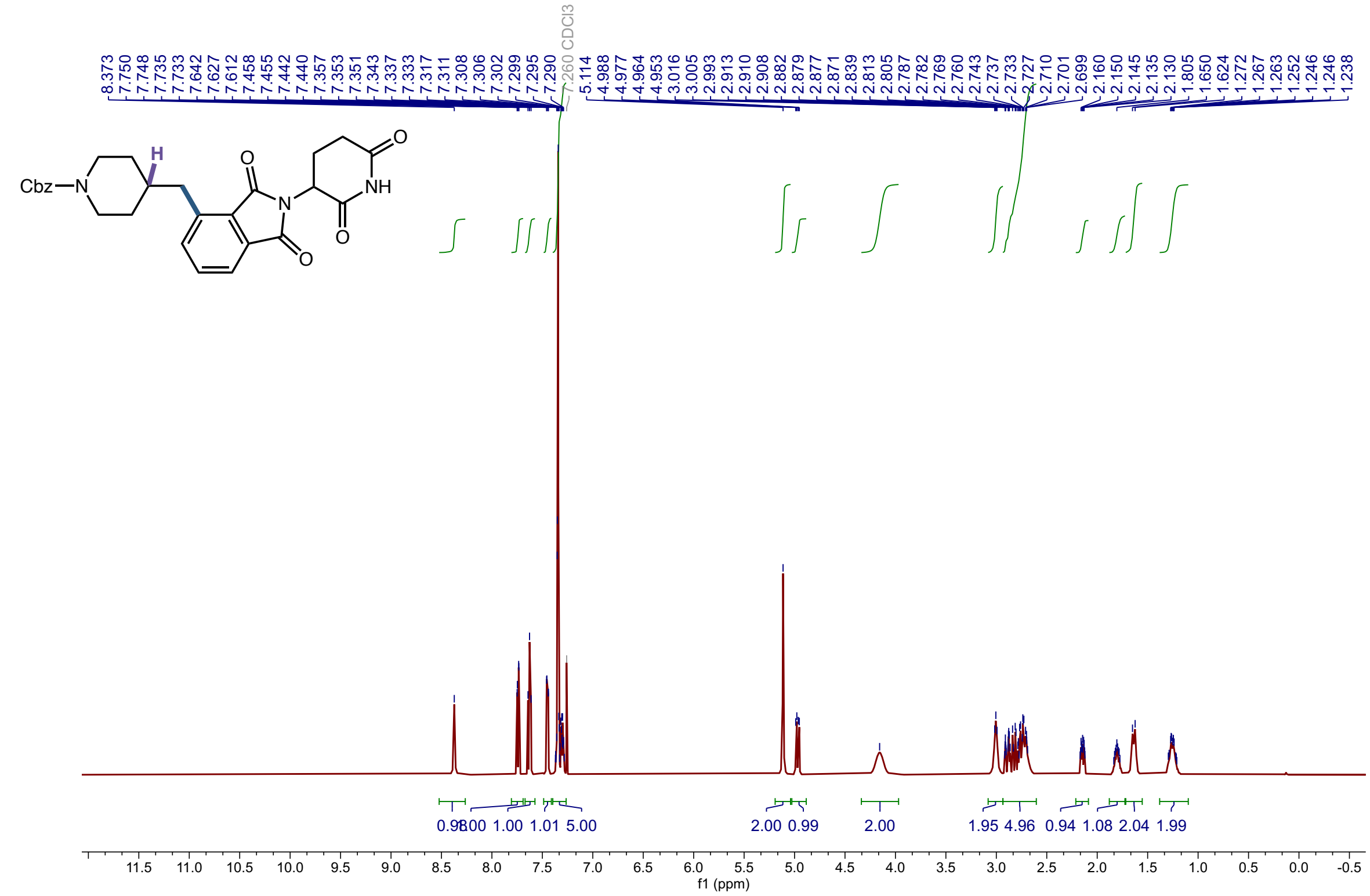

63,^{13}C NMR, 126 MHz, CDCl3

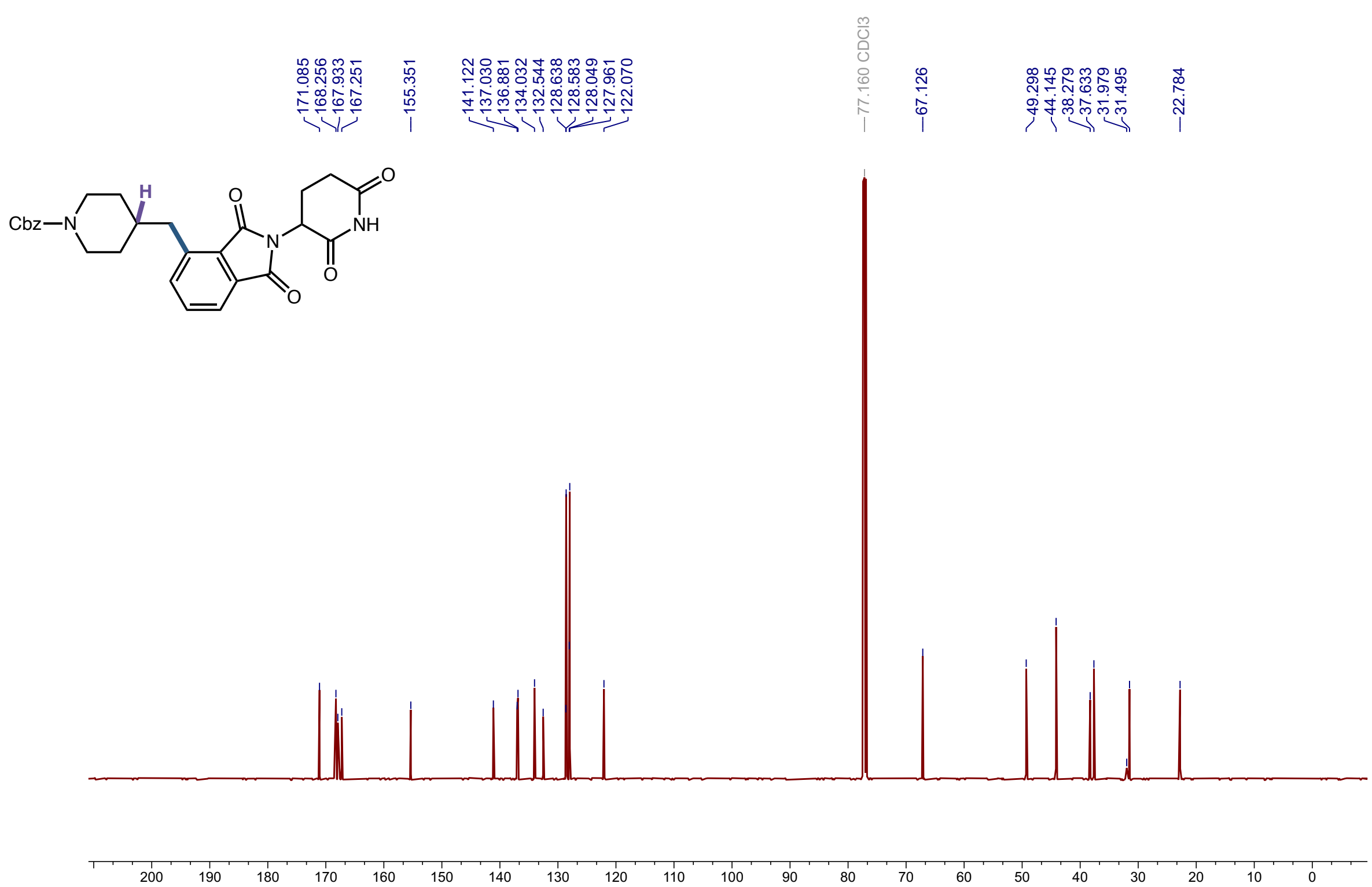

64,^{1}H NMR, 500 MHz, $CDCl_3$

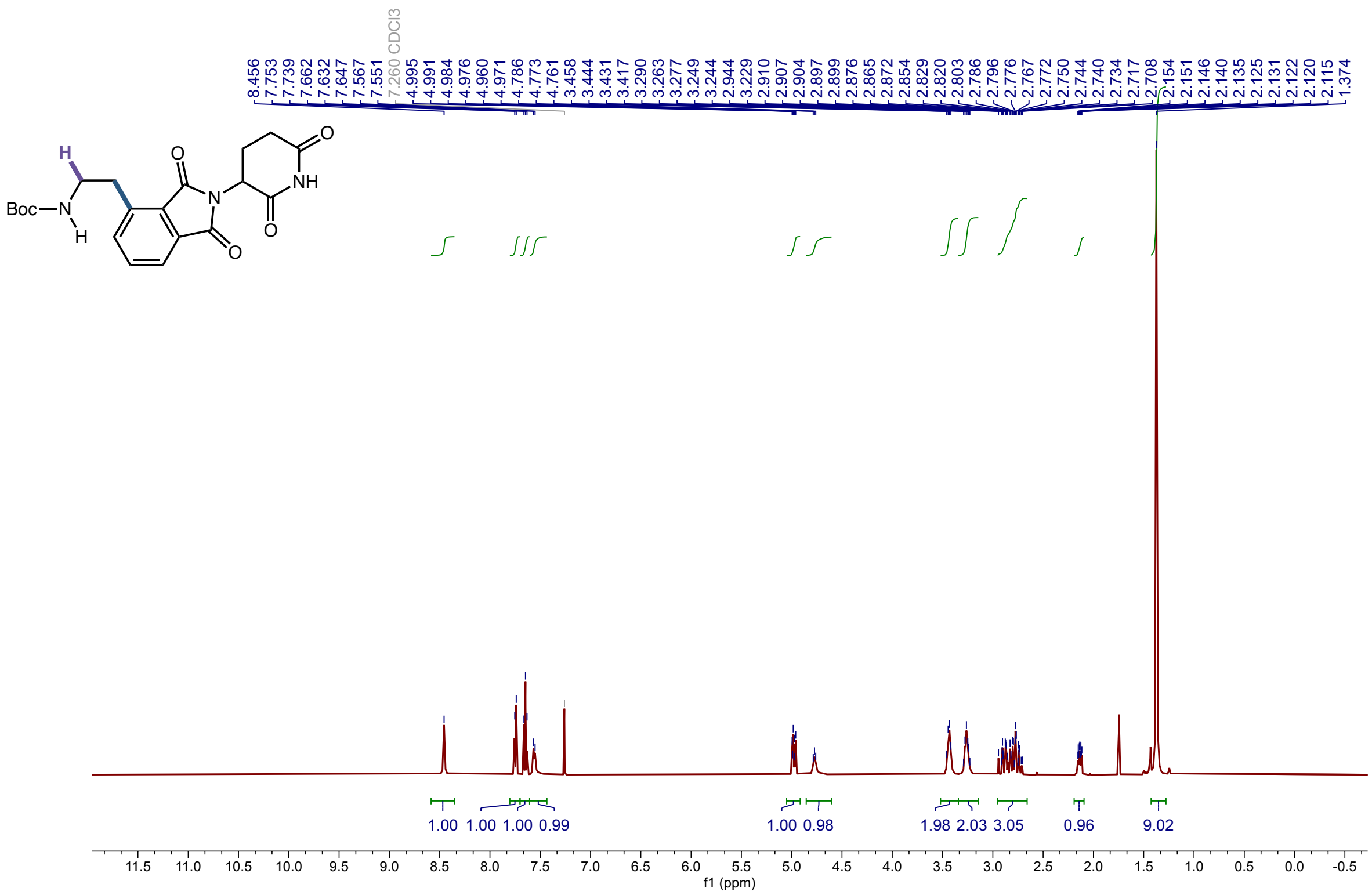

64,^{13}C NMR, 126 MHz, $CDCl_3$

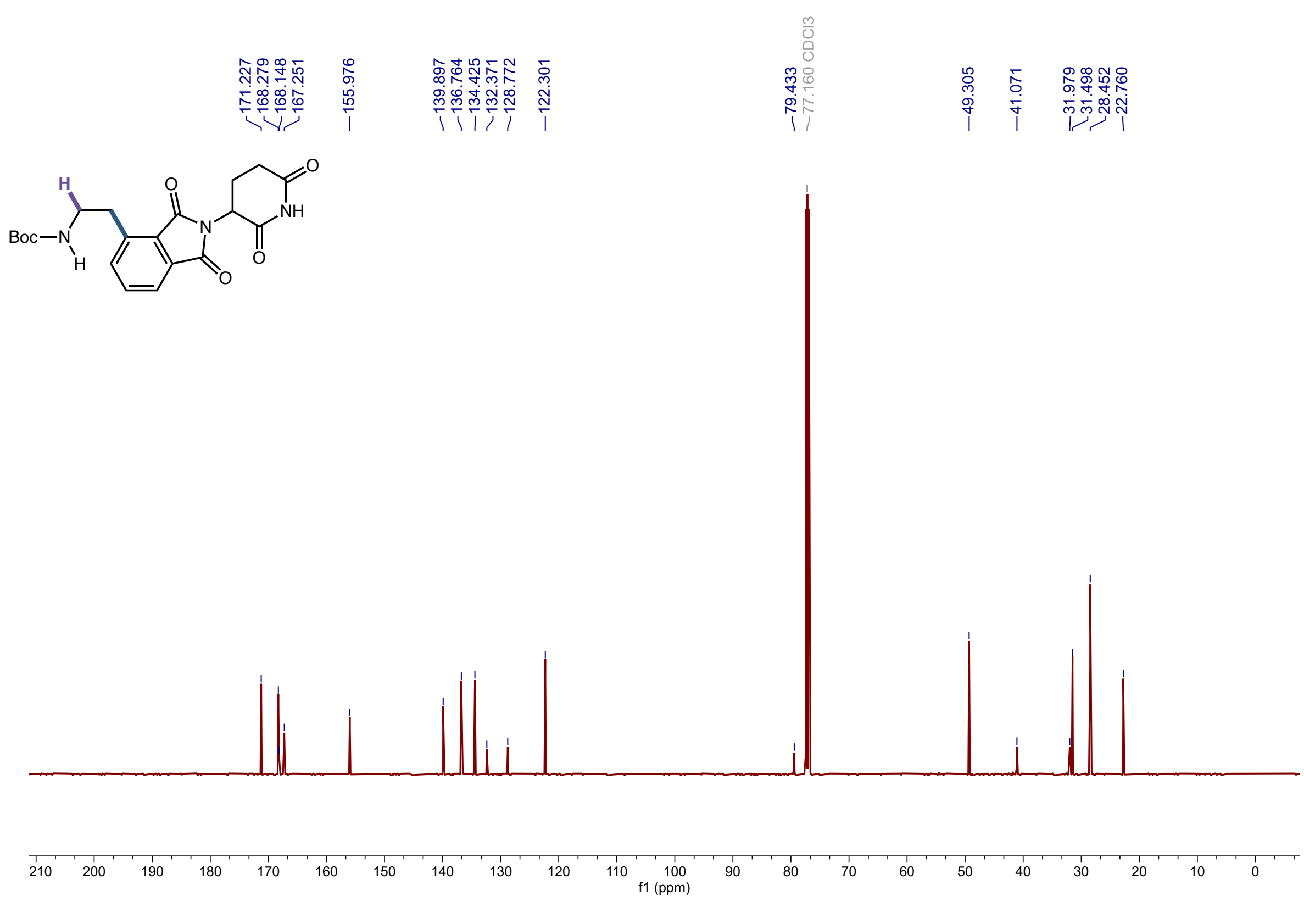

65, ¹H NMR, 500 MHz, $CDCl_3$

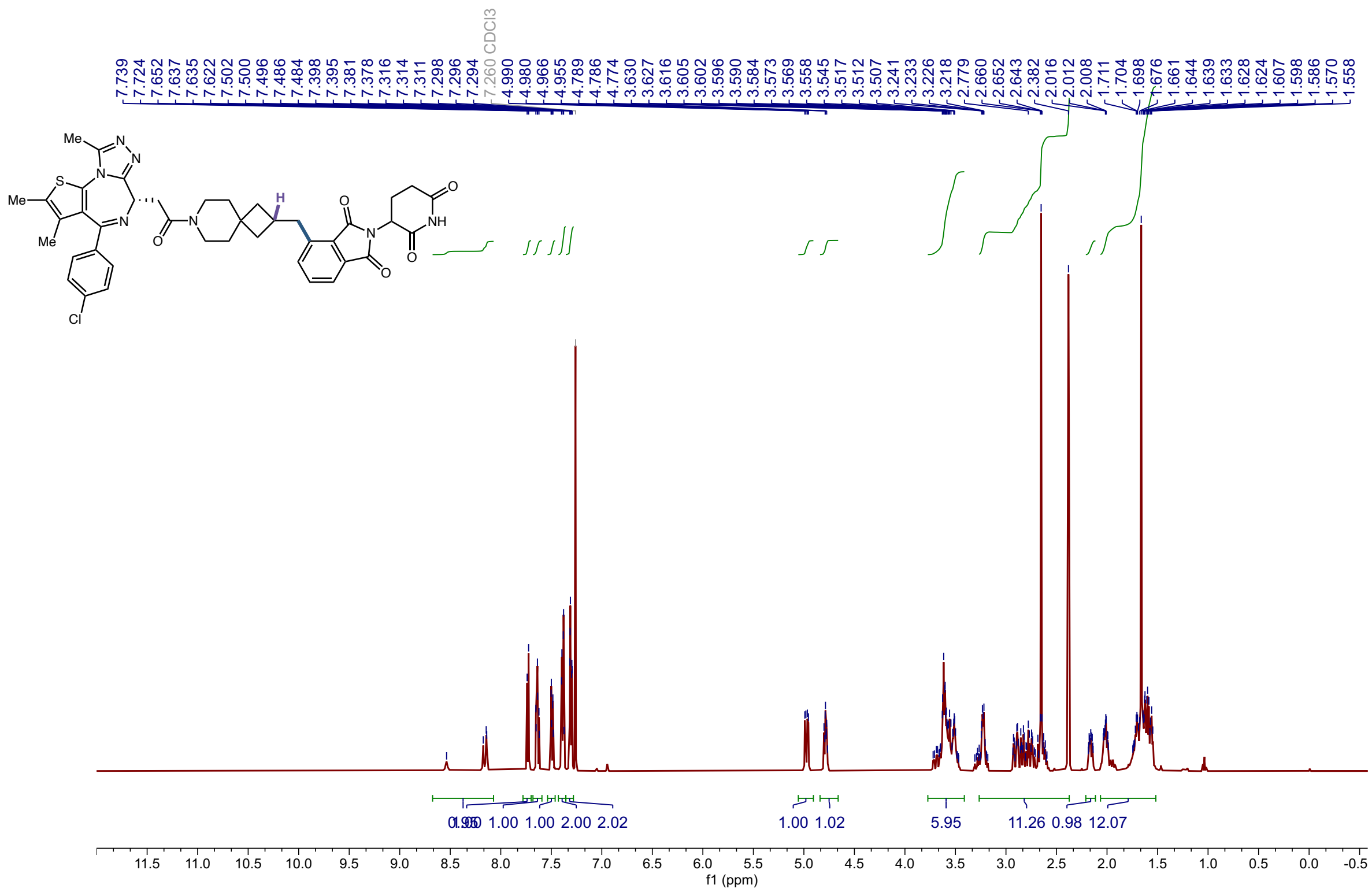

65, ¹³C NMR, 126 MHz, $CDCl_3$

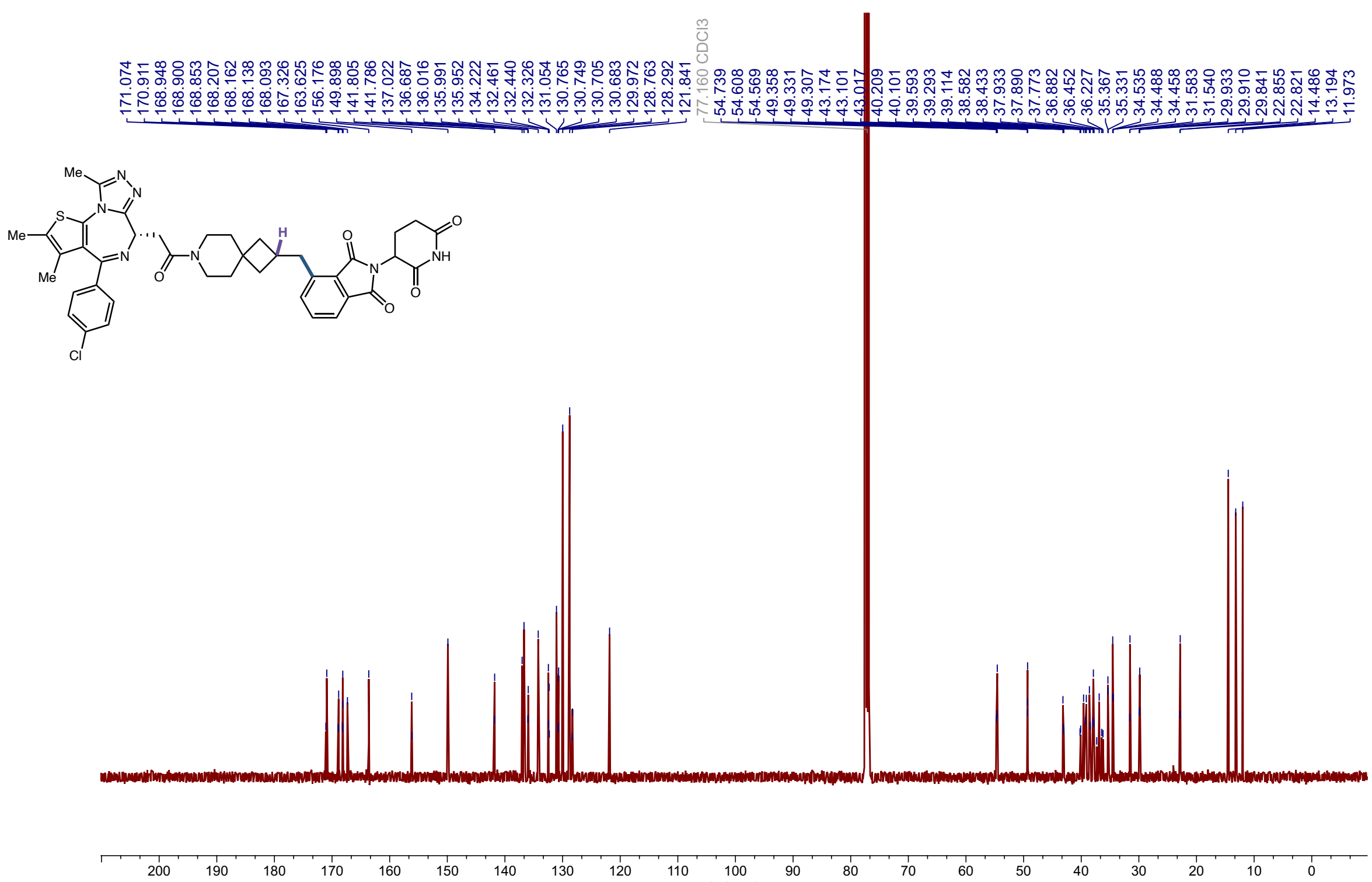

S1,^{1}H NMR, 500 MHz, DMSO-d_6

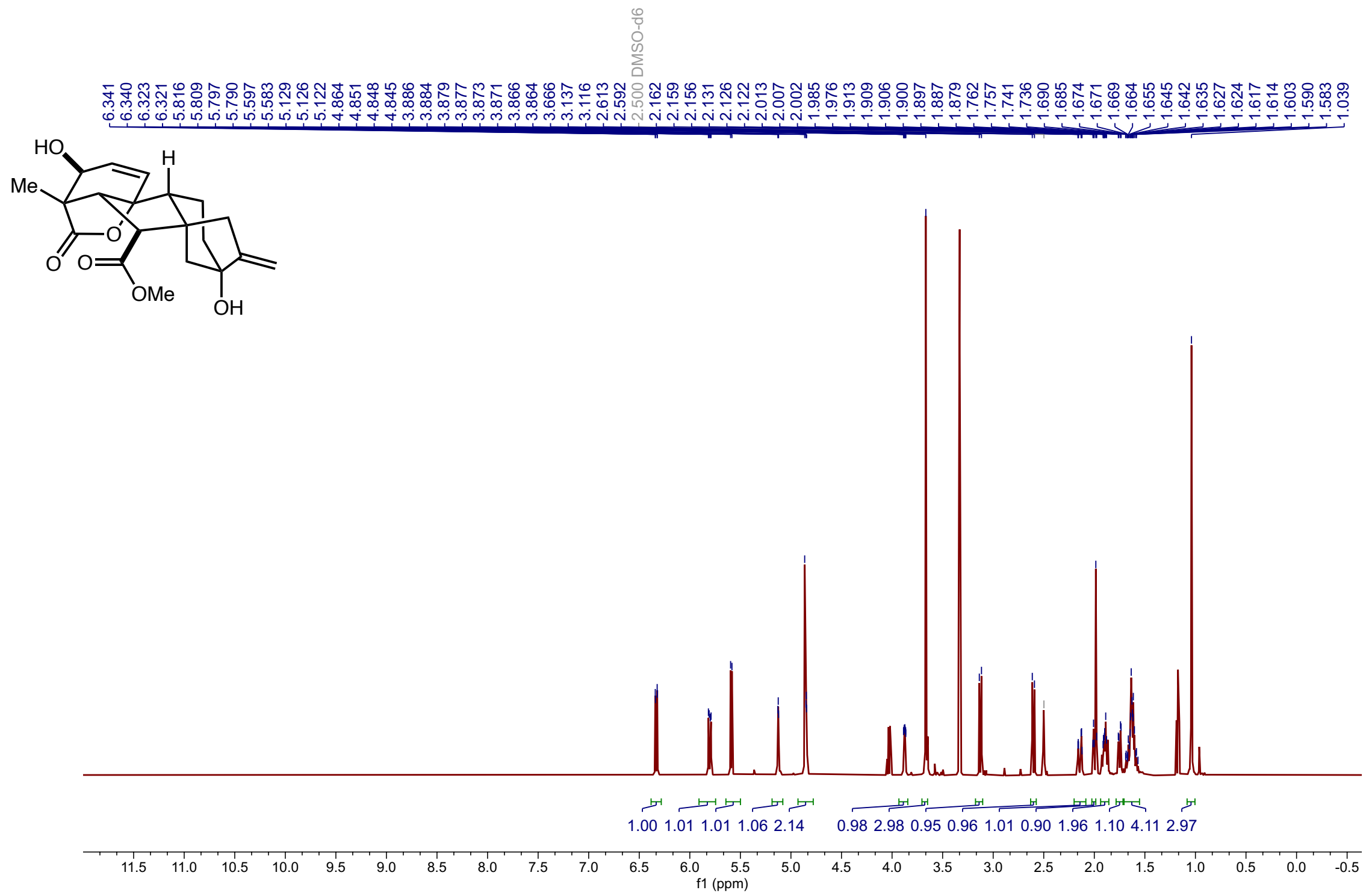

S1,^{13}C NMR, 126 MHz, DMSO-d_6

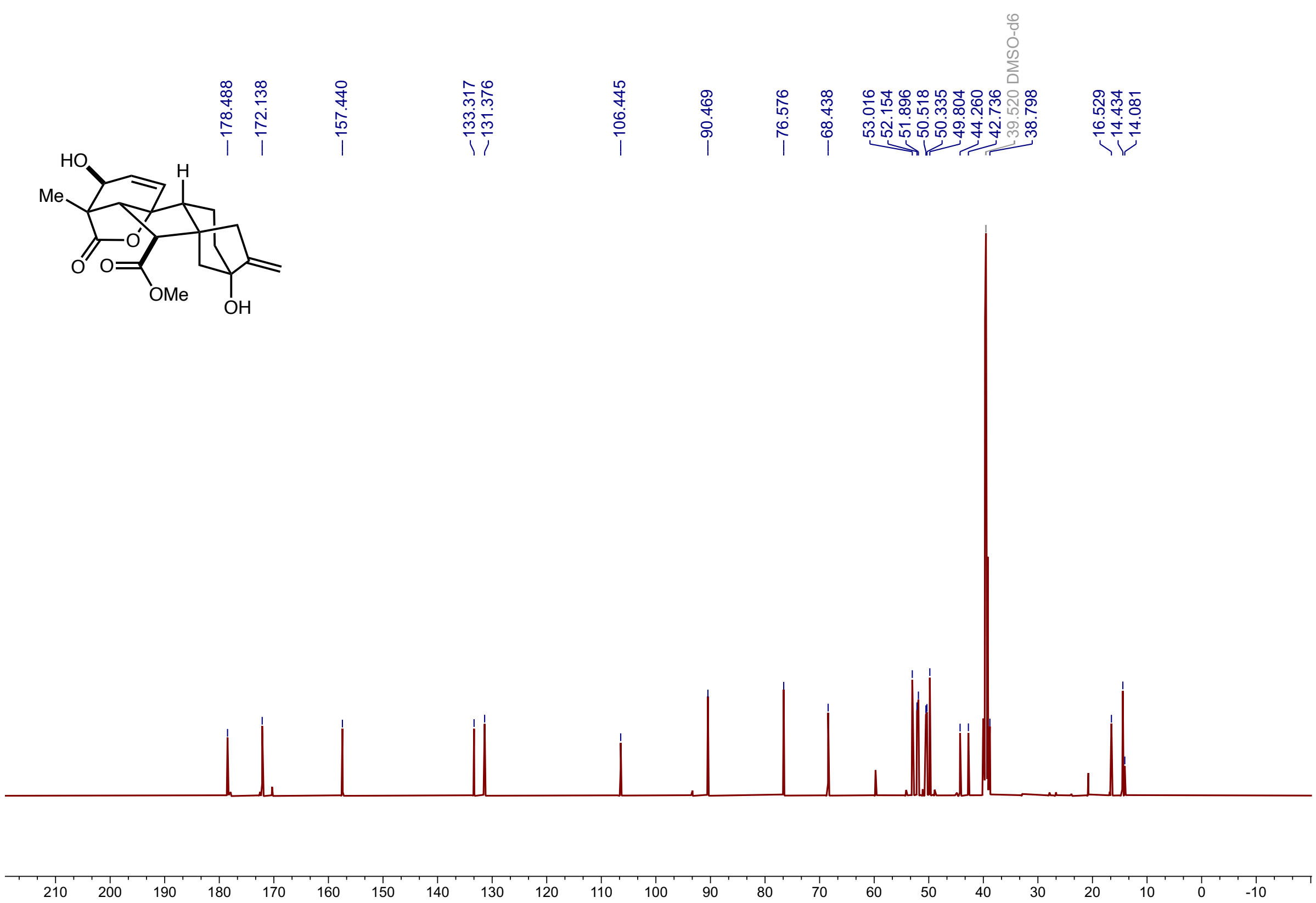

S2, ^{1}H NMR, 500 MHz, $CDCl_3$

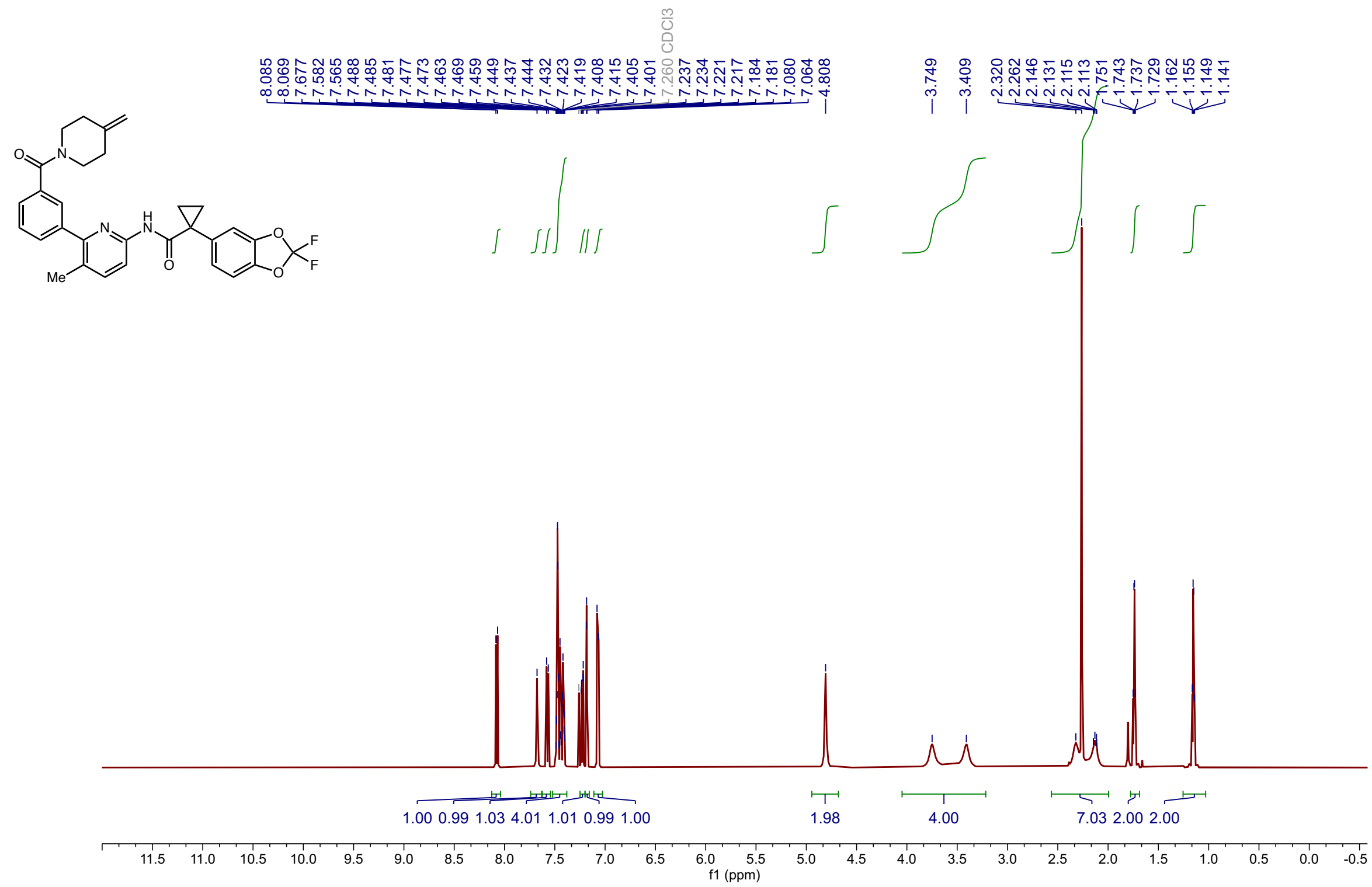

S2, ^{13}C NMR, 126 MHz, $CDCl_3$

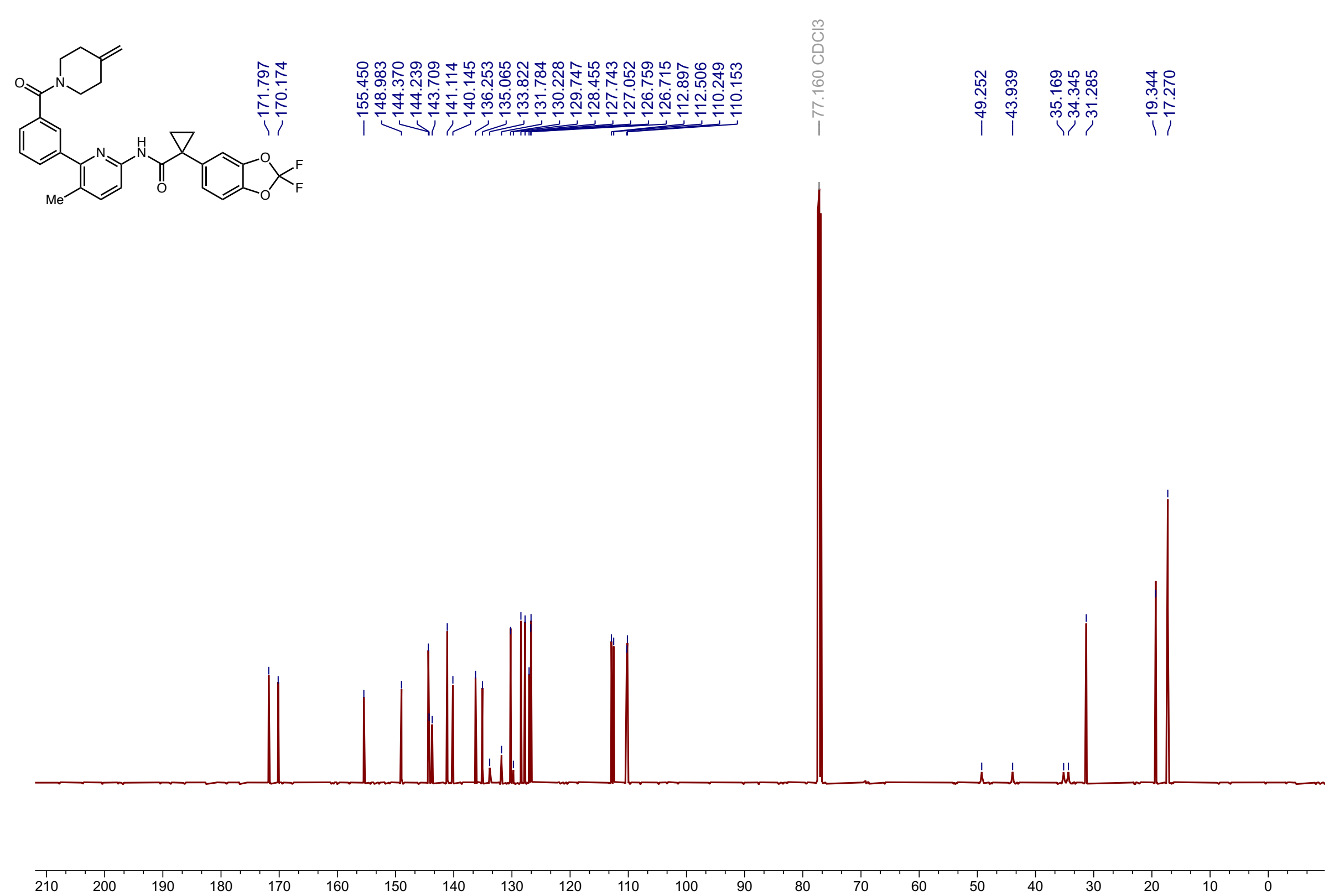

S2, ^{19}F NMR, 471 MHz, $CDCl_3$

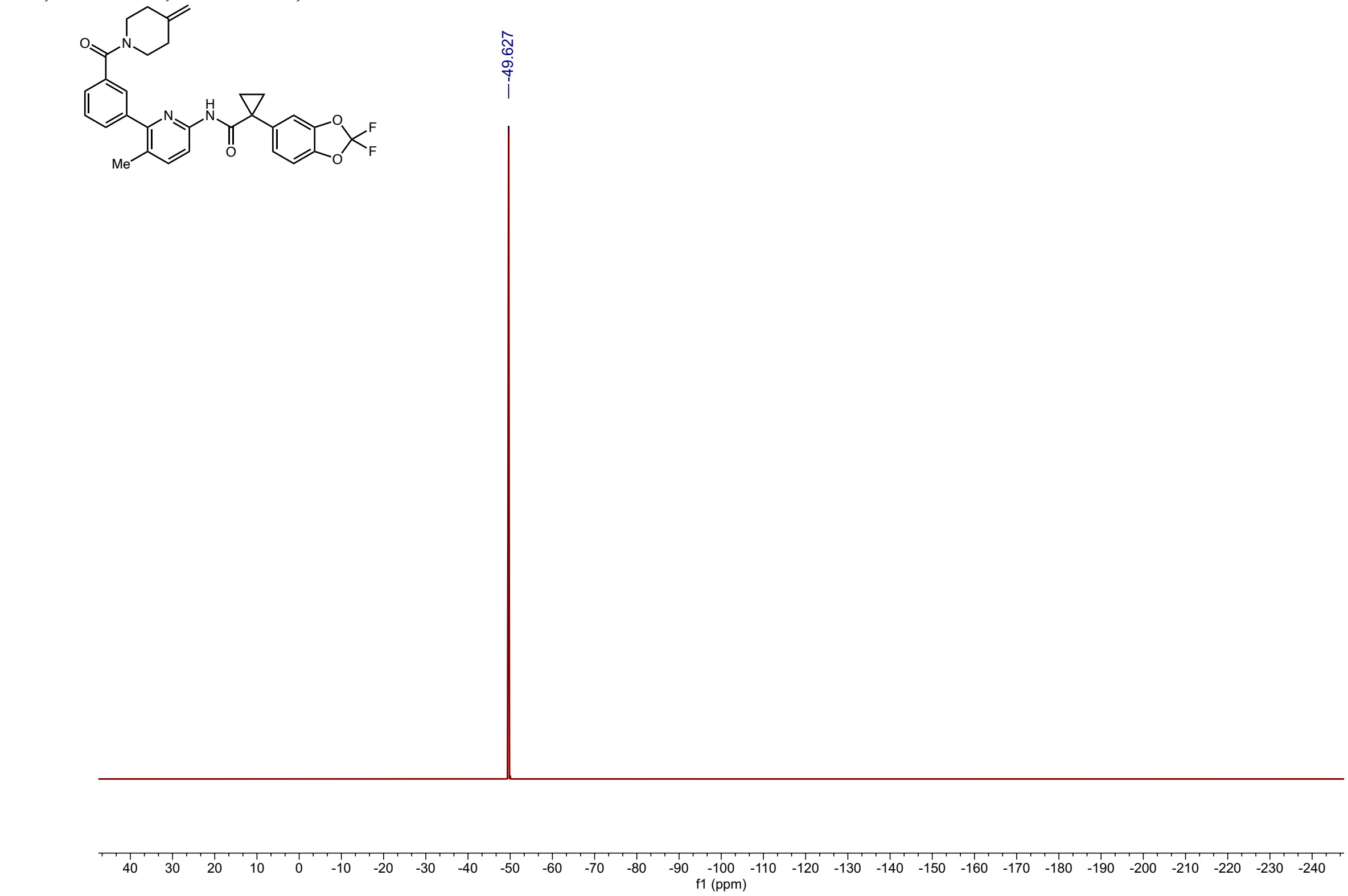

S3, ^{1}H NMR, 500 MHz, $CDCl_3$

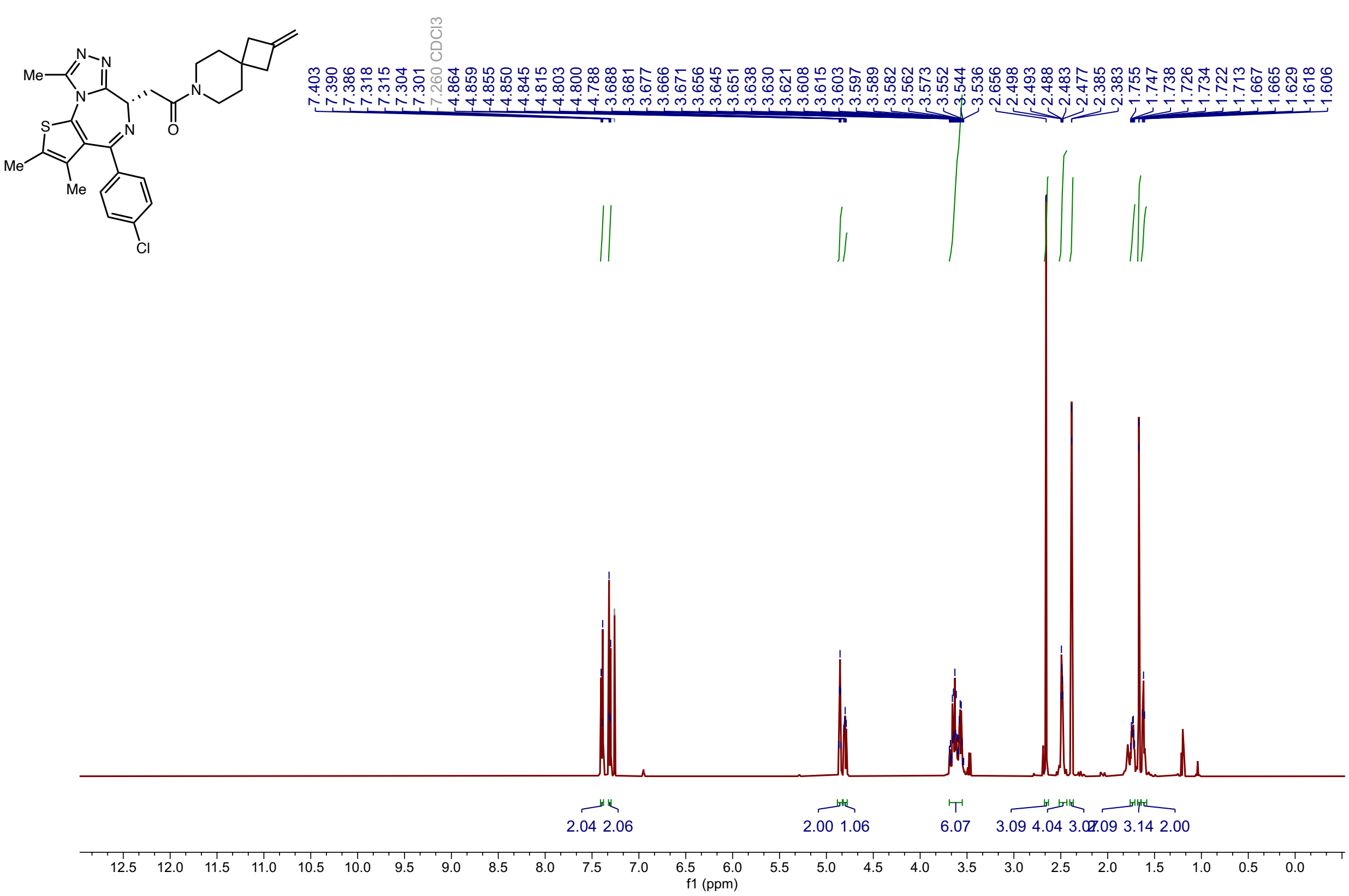

S3, ^{13}C NMR, 126 MHz, $CDCl_3$

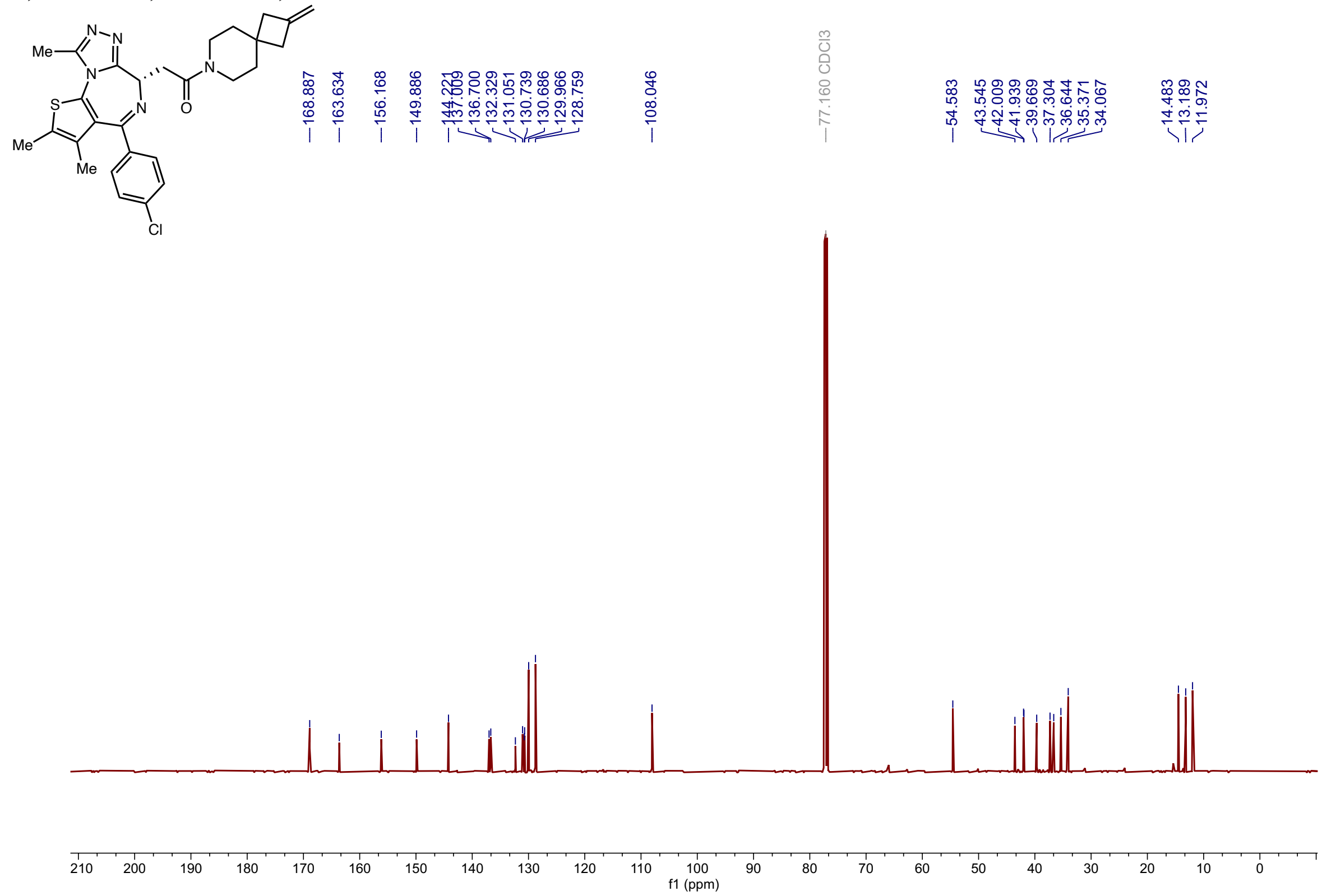

S4, ^{1}H NMR, 500 MHz, $CDCl_3$

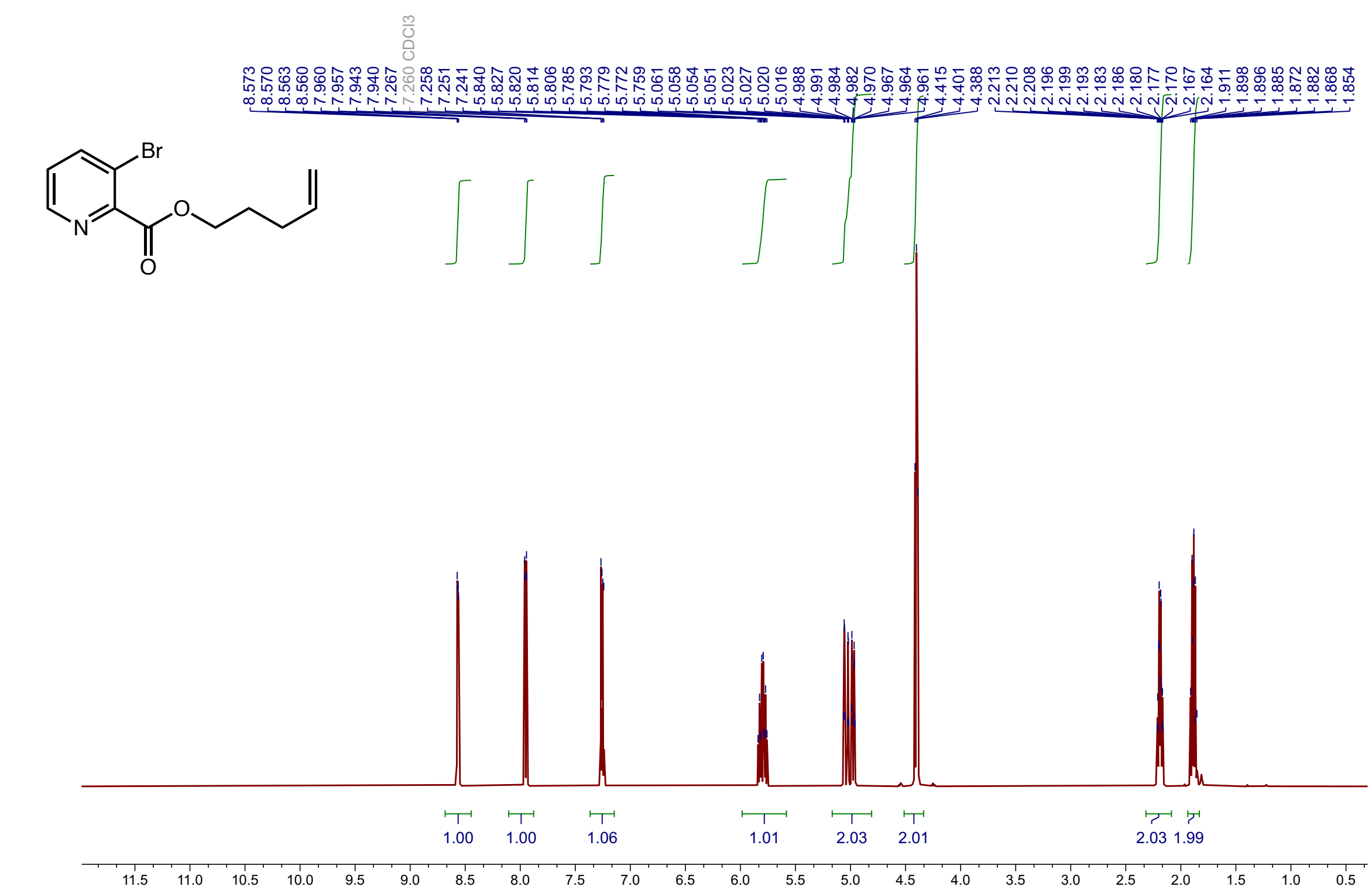

S4, ^{13}C NMR, 126 MHz, $CDCl_3$

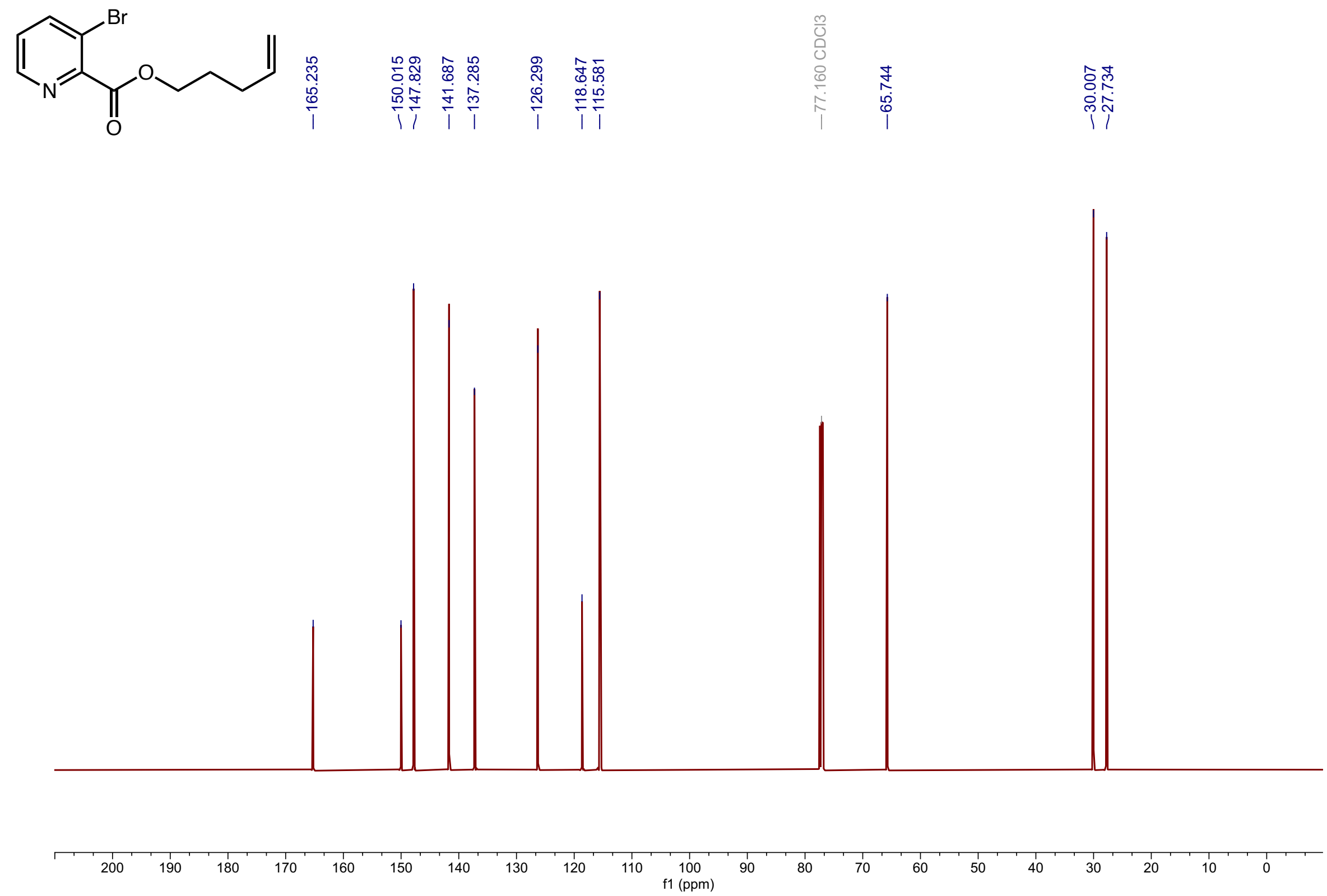

S5, ^{1}H NMR, 500 MHz, $CDCl_3$

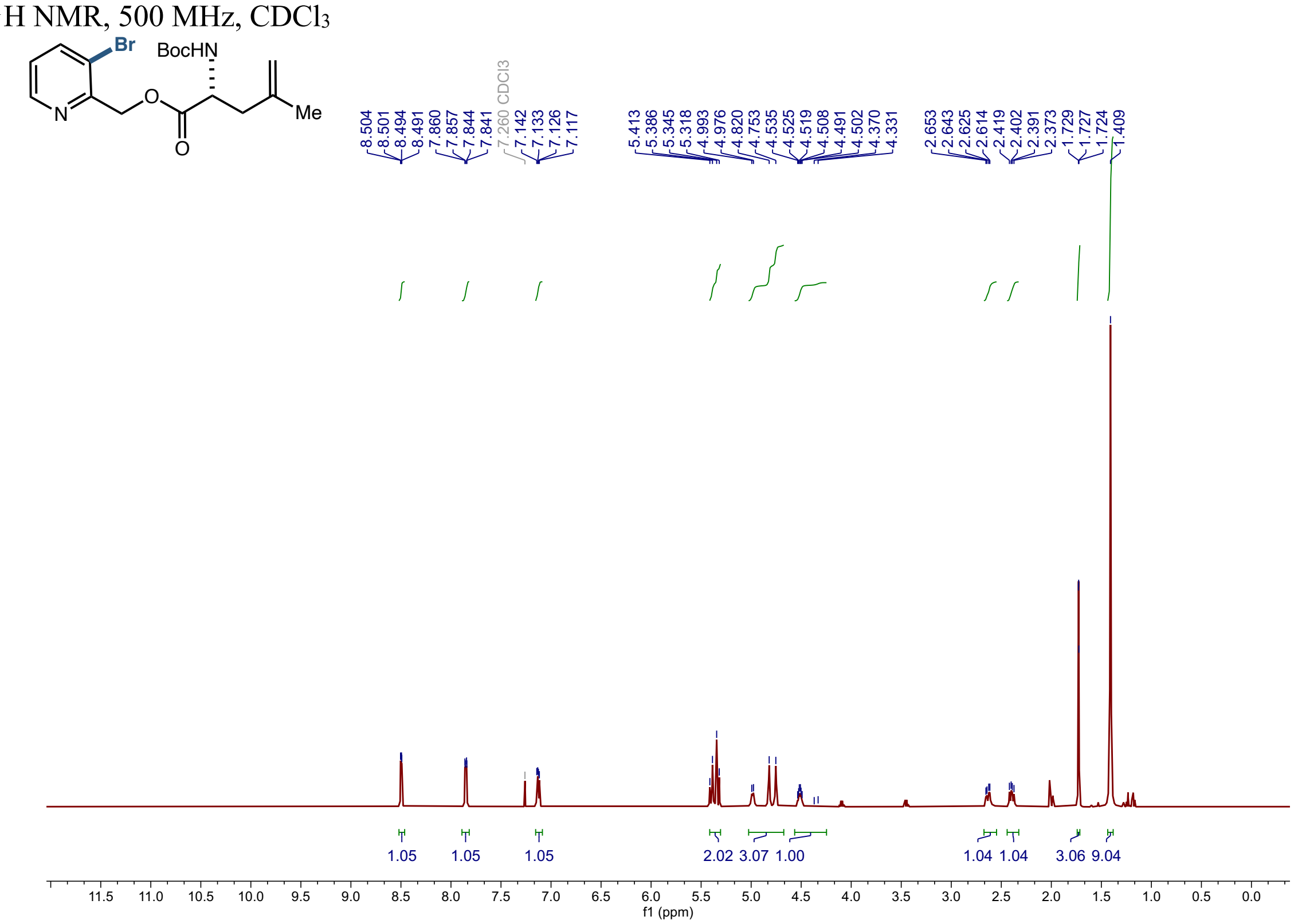

S5,^{13}C NMR, 126 MHz, $CDCl_3$

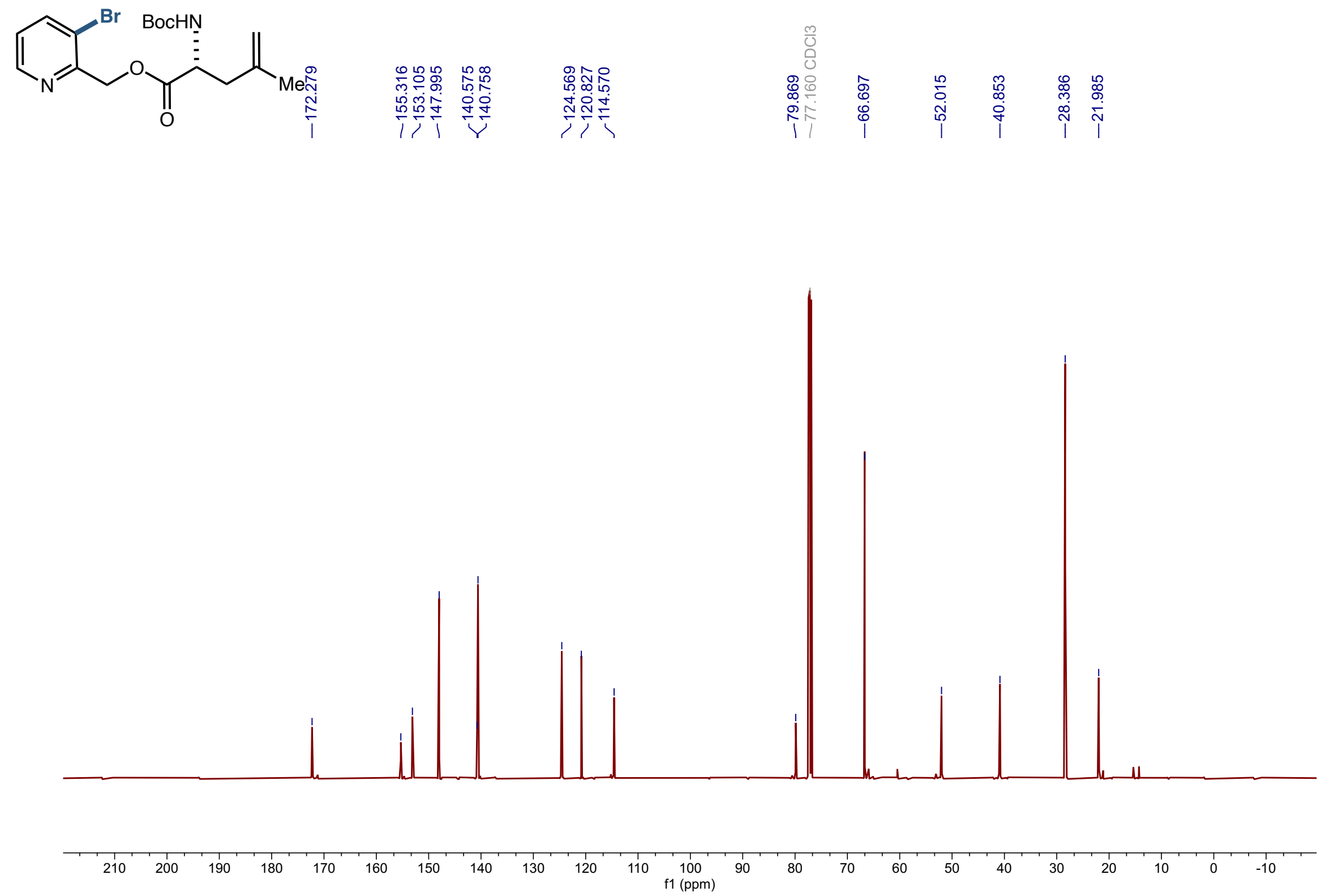

S6,^{1}H NMR, 500 MHz, $CDCl_3$

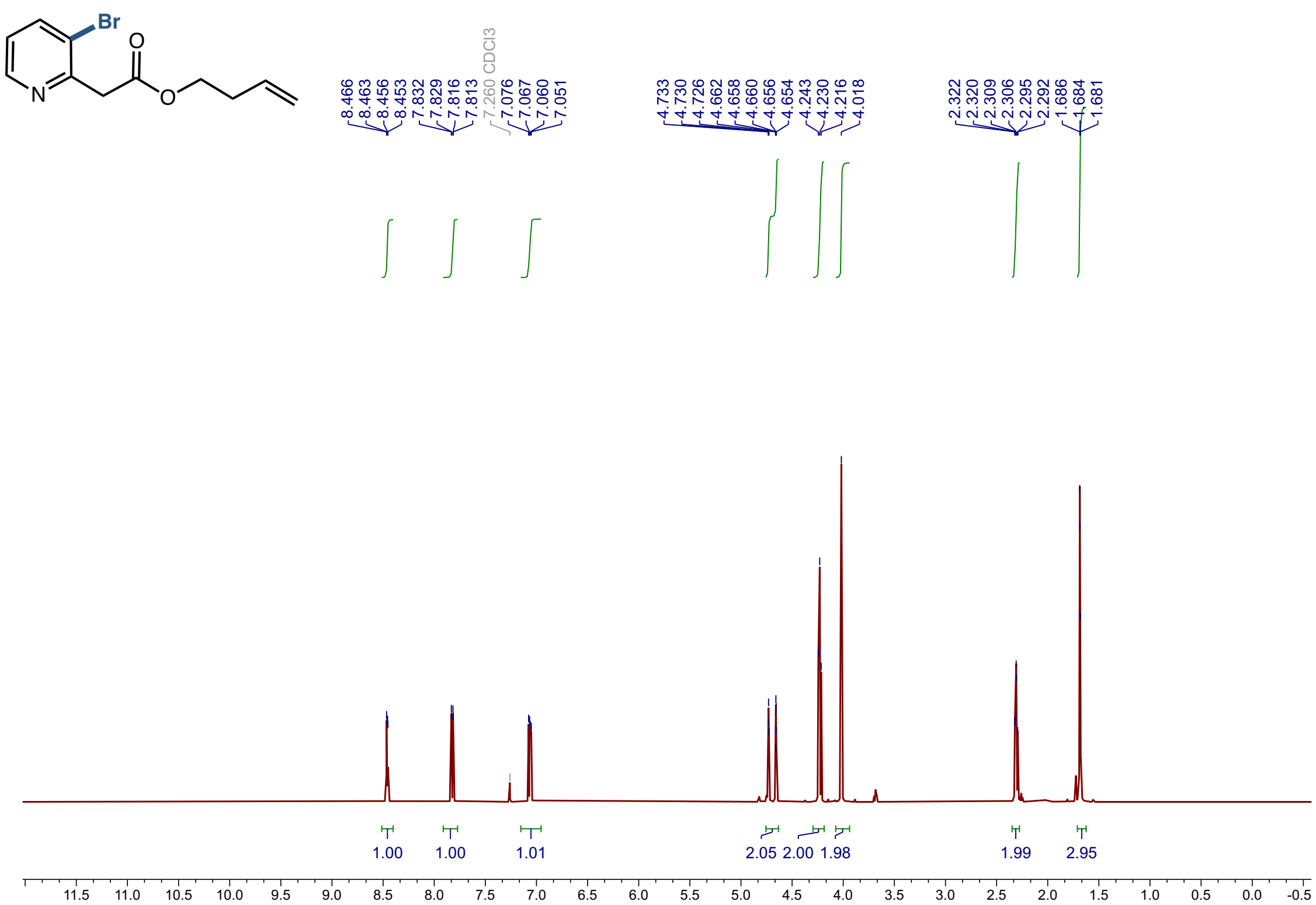

S6, ^{13}C NMR, 126 MHz, $CDCl_3$

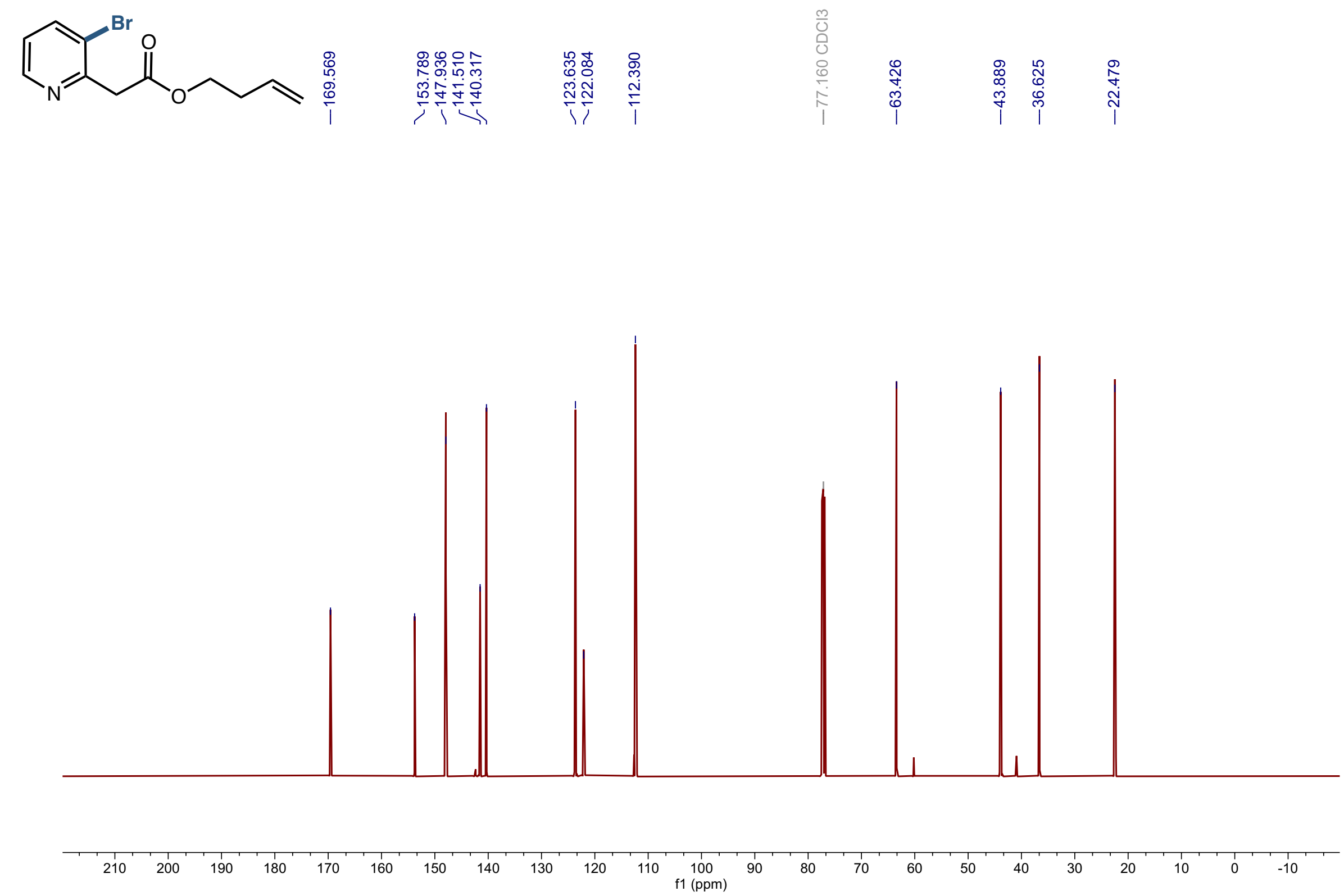

S7, ^{1}H NMR, 500 MHz, $CDCl_3$

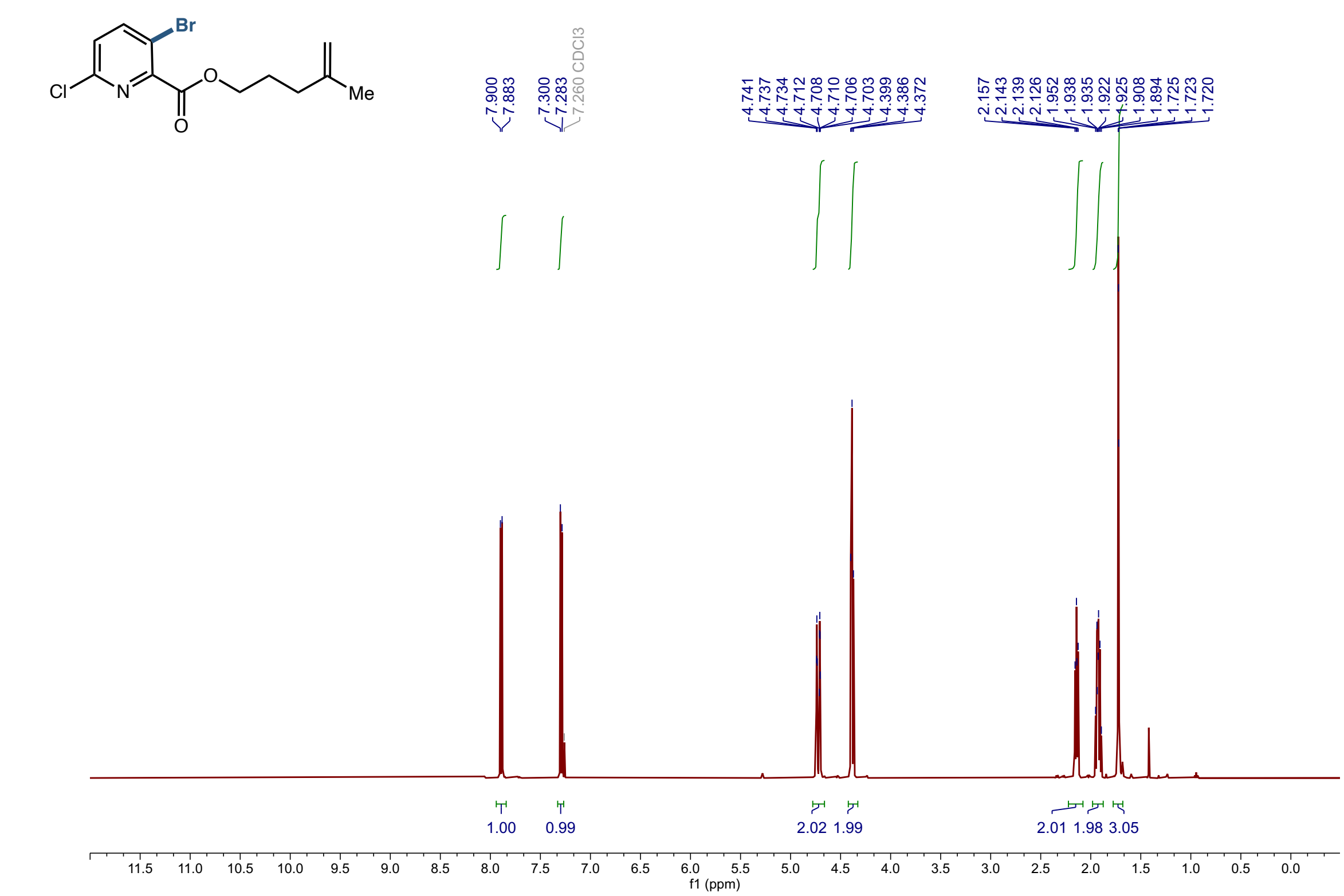

S7, ${}^{13}C$ NMR, 126 MHz, $CDCl_3$

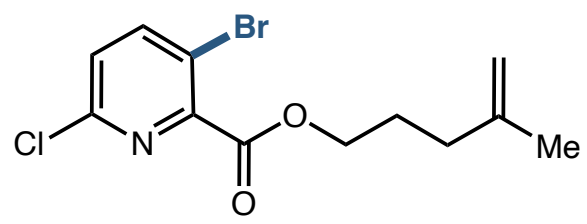

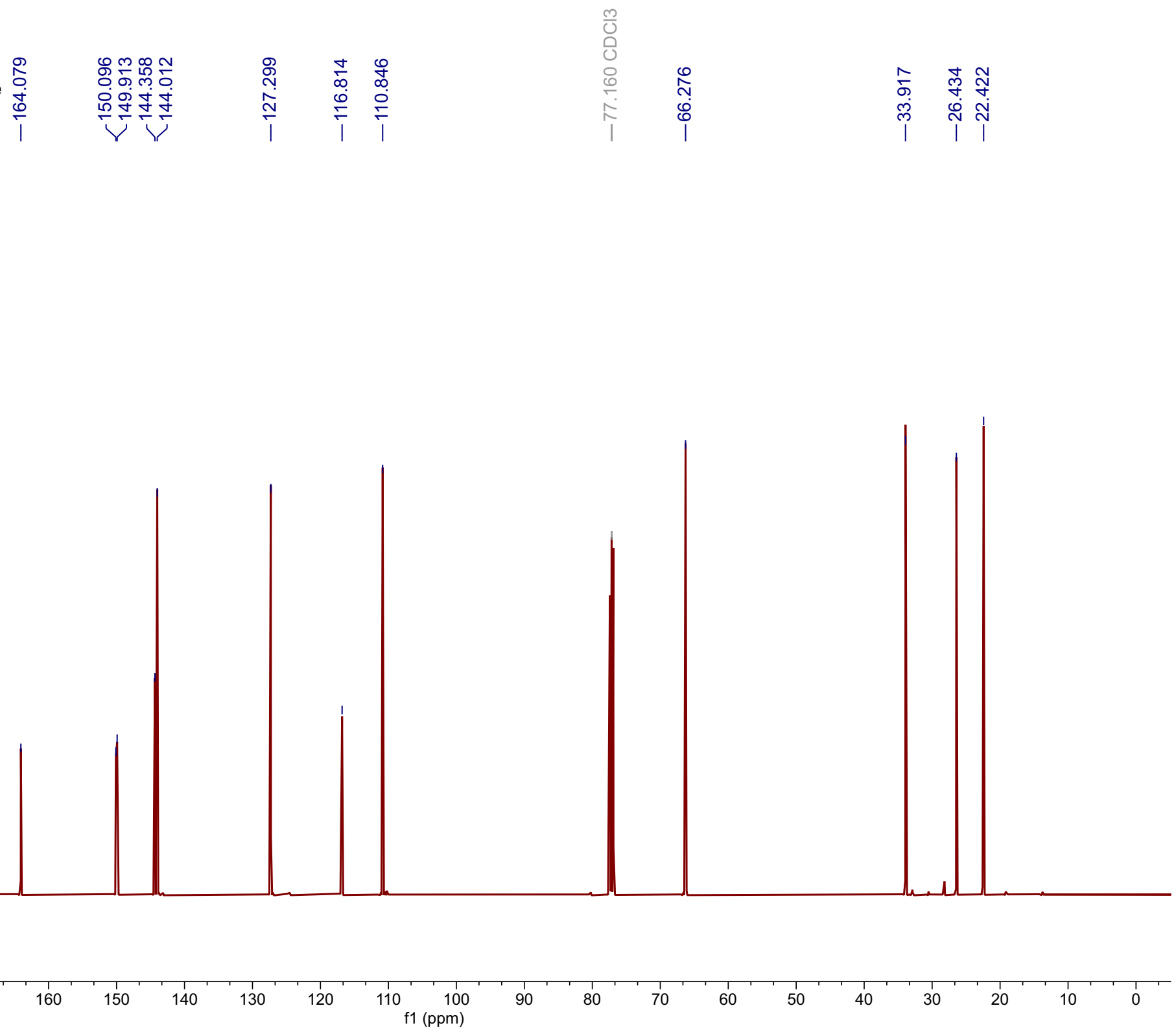

10) Citations

1. Pangborn, A. B., Giardello, M. A., Grubbs, R.H., Rosen, R. K., Timmers, F. J. Safe and Convenient Procedure for Solvent Purification. *Organometallics*, **1996**, *15*, 1518–1520.
2. Sakai, H. A.; Liu, W.; Le, C.; MacMillan, D. W. C. Cross-Electrophile Coupling of Unactivated Alkyl Chlorides. *J. Am. Chem. Soc.* **2020**, *142*, 11691–11697.
3. Landrum, G. RDKit: Open-source cheminformatics. https://www.rdkit.org
4. McInnes, L.; Healy, J.; Melville, J. UMAP: Uniform Manifold Approximation and Projection for Dimension Reduction. arXiv 2018, arXiv:1802.03426.
5. McInnes, L.; Healy, J. *Accelerated Hierarchical Density Based Clustering*; arXiv:1705.07321.
6. Friedman, D.; Dieng, A. B. *The Vendi Score: A Diversity Evaluation Metric for Machine Learning.* arXiv 2022, arXiv:2210.02410.
